# Artificial Metalloenzyme-Catalyzed Enantioselective Amidation via Nitrene Insertion in Unactivated C(*sp*^3^)–H Bonds

**DOI:** 10.1021/jacs.3c03969

**Published:** 2023-07-20

**Authors:** Kun Yu, Zhi Zou, Nico V. Igareta, Ryo Tachibana, Julia Bechter, Valentin Köhler, Dongping Chen, Thomas R. Ward

**Affiliations:** † Department of Chemistry, University of Basel, Mattenstrasse 24a, BPR 1096, Basel CH-4058, Switzerland

## Abstract

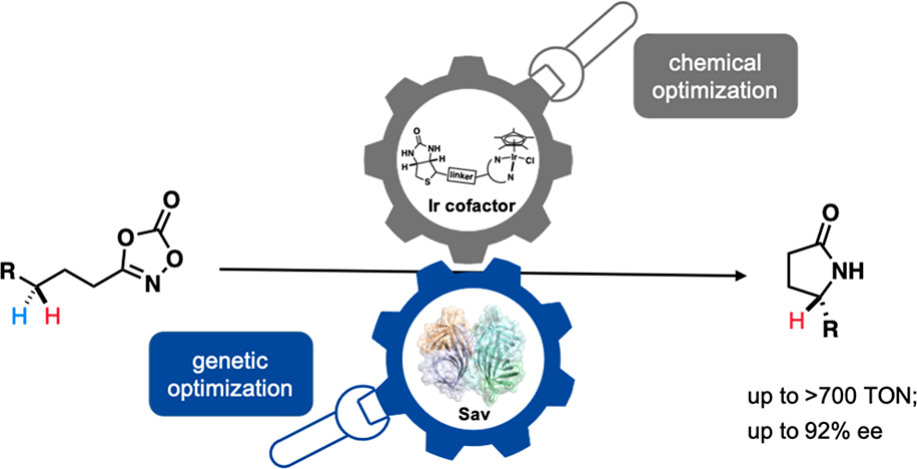

Enantioselective C–H amidation offers attractive means to assemble C–N bonds to synthesize high-added value, nitrogen-containing molecules. In recent decades, complementary enzymatic and homogeneous-catalytic strategies for C–H amidation have been reported. Herein, we report on an artificial metalloenzyme (ArM) resulting from anchoring a biotinylated Ir-complex within streptavidin (Sav). The resulting ArM catalyzes the enantioselective amidation of unactivated C(*sp*^3^)–H bonds. Chemogenetic optimization of the Ir cofactor and Sav led to significant improvement in both the activity and enantioselectivity. Up to >700 TON and 92% ee for the amidation of unactivated C(*sp*^3^)–H bonds was achieved. The single crystal X-ray analysis of the artificial nitrene insertase (ANIase) combined with quantum mechanics-molecular mechanics (QM-MM) calculations sheds light on critical second coordination sphere contacts leading to improved catalytic performance.

## 1 Introduction

Nitrogen-containing motifs are prevalent in natural products, functional materials, and pharmaceuticals.^1^ Various strategies for synthesizing nitrogen-containing compounds, such as nucleophilic substitution, condensation, reductive amination, and hydroamination, have been developed in the past decades.^2^ These strategies, however, mostly rely on functional group interconversion, often leading to significant waste generation. Methods for constructing C–N bonds enabled by transition metal catalysts were developed to provide direct access to these functionalities. Among these, the Pd-catalyzed Buchwald–Hartwig amination was one of the most representative examples and has been widely applied in academia and industry.^3,4^ However, this reaction only applies to substrates containing a (pseudo)halide, thus requiring pre-functionalized substrates.

**Scheme 1:**
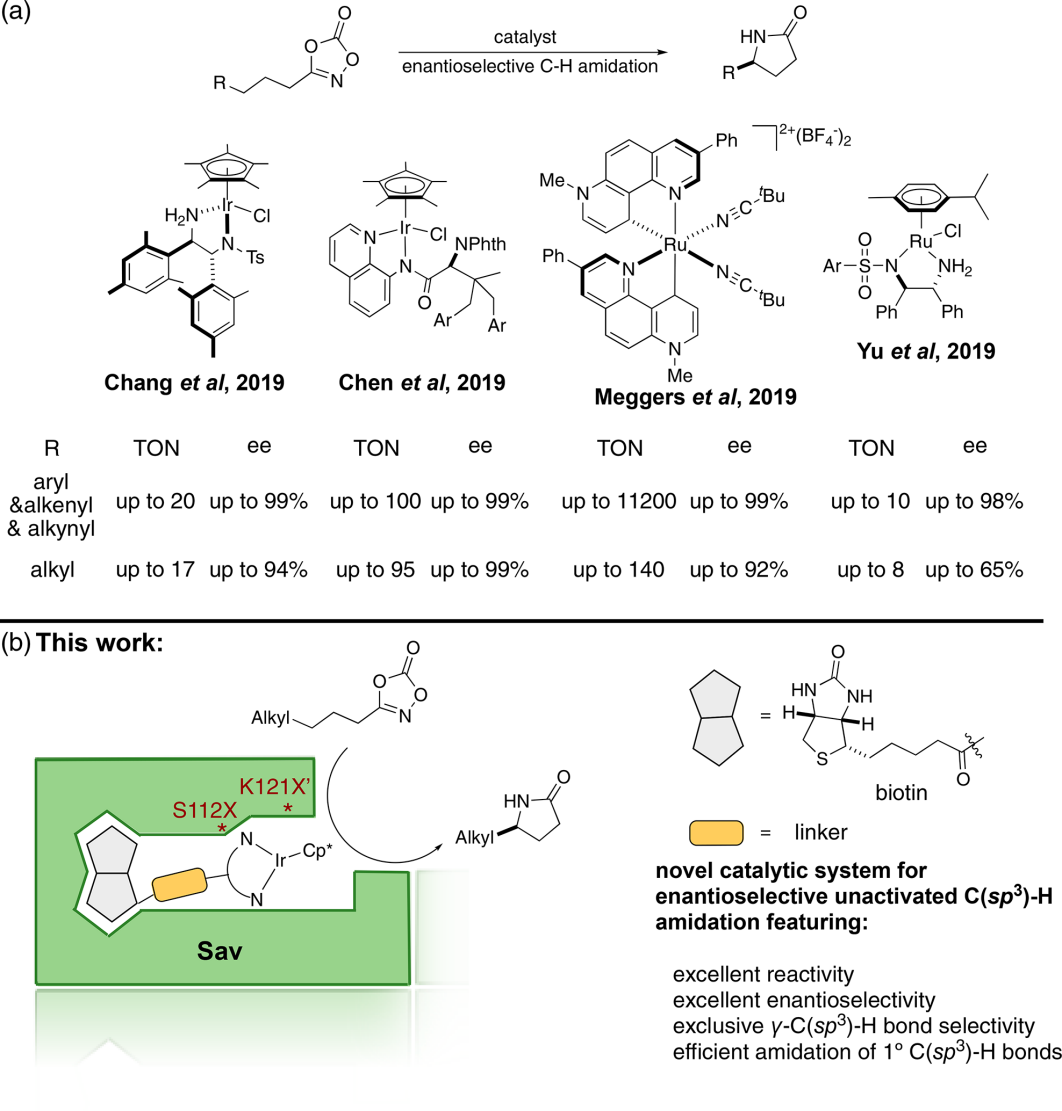
Selected Examples of Catalytic Enantioselective Amidation of Unactivated C(*sp*^3^)–H Bonds Relying on Dioxazolones-Containing Substrates (a) Reported homogeneous catalytic systems. (b) Artificial nitrene insertase (ANIase) based on anchoring a biotinylated Cp*Ir complex into streptavidin. Cp* = pentamethylcyclopentadiene.

Developing more direct and atom-economic strategies to construct C–N bonds has attracted increasing attention recently.^3^ Pioneering work was reported as early as 1983 by Breslow, relying on Fe(III)- or Rh(II)-catalyzed synthesis of oxathiazolidine using hypervalent ylides as sulfonylnitrene precursors.^5^ Capitalizing on this work, many research groups have exploited the potential of metal-nitrene chemistry to create C–N bonds, relying on either homogeneous catalysts or repurposed enzymes.^2,3,6−15^ Recently, Chang and co-workers reported an elegant Ir-catalyst for synthesizing γ-lactams, using dioxazolones as nitrene precursors.^16^ Since then, Chang,^17^ Yu,^18^ Chen,^19^ and Meggers^20^ reported enantioselective variations of this C–H amidation, see Scheme 1a. All four groups obtained excellent enantioselectivity with their catalytic systems. Except for the system reported by Meggers, high catalyst loadings were typically required for the enantioselective amidation of unactivated, purely aliphatic C(*sp*^3^)–H bonds, affording modest turnover numbers, Scheme 1a. More recently, another highly efficient catalytic system was reported by Chang and co-workers, and up to 47000 TON was obtained for the racemic amidation of C–H bonds.^21^

To complement homogeneous catalysis, repurposed enzymes have attracted increasing attention in the context of enantioselective nitrene insertion.^14,22,23^ Compared to homogeneous catalysis, enzyme catalysis displays some distinct advantages, including high specificity, mild (aqueous) reaction conditions, high turnover numbers, and compatibility with biological systems. During the preparation of this paper, Fasan and co-workers reported repurposed myoglobin-catalyzed stereoselective construction of β-, γ-, and δ-lactams using dioxazolones as substrates. Utilizing their engineered enzymatic catalysis system, they achieved high enantioselectivity and TON for both benzylic and allylic C–H amidation reactions.^24^

With the aim of combining the advantages of both homogeneous and enzymatic catalysis,^25,26^ artificial metalloenzymes (ArMs) have emerged as an attractive means to endow organometallic catalysts with an evolvable genotype. ArMs result from the incorporation of a catalytically competent metallocofactor into a genetically encoded protein. Since the first example of ArMs reported by Wilson and Whitesides in the late 1970s,^27^ several protein scaffolds have proven versatile for assembling and optimizing of such hybrid catalysts. These include the following: human carbonic anhydrase II,^28^ hemoproteins,^29−32^ proline oligopeptidase,^33^ lactococcal multiresistance regulator,^34^ nitrobindin,^35^ four-helix bundles,^36^ streptavidin,^37−40^ etc.^41−51^ Regarding the advantages of ArMs, the introduction of metallocofactors endows the host protein with new-to-nature catalytic activity, thus potentially contributing to expand the catalytic repertoire of (natural) enzymes. The presence of a well-defined second coordination sphere provided by the host protein may enable the achievement of high levels of selectivities. Importantly, the ArMs’ performance can be improved by combining both chemical optimization (i.e., modification of the cofactor and linker structures) and genetic optimization (directed evolution of the host protein).^52^ Based on our experience with Cp*Ir-pianostool cofactors,^41^ we set out to evaluate the potential of [Cp*Ir(amidoquinoline)Cl] and [Cp*Ir(aminosulfonamide)Cl]-derived cofactors, to engineer an ANIase based on the biotin–streptavidin technology, Scheme 1b.

## 2 Results and Discussion

Inspired by the seminal publication of Wilson and Whitesides,^27^ several groups have capitalized on the biotin-Sav technology to develop ArMs that catalyze a variety of transformations including: hydrogenation,^56^ transfer hydrogenation,^55^^57,58^ cross-coupling,^59^ olefin metathesis,^60,61^ C–H activation,^62,63^ hydroxylation, hydroamination, etc.^33,37−41,64−68^ The versatility of the biotin-Sav technology for the assembly of ArMs can be traced back to the high affinity of biotin for Sav (*K*_d_ < 10^–13^ M), as well as the remarkable stability of Sav against chaotropic agents, including organic solvents, temperature, pH, etc. With the aim of identifying the most promising biotinylated Cp*Ir cofactor, we tested both 8-amidoquinoline and aminosulfonamide bidentate ligands. In total, eight biotinylated cofactors **3**–**10** were tested in the presence of wild-type mature streptavidin (WT Sav).^69^ We selected dioxazolone **1** as the model substrate for the chemical optimization of ANIase activity, as shown in Table 1. To our delight, varying amounts of the γ-lactam **2** were detected by chiral GC analysis for all of the cofactors embedded within Sav WT. Notably, the biotin’s anchoring position and the bidentate ligand’s nature have a marked influence on the ANIase’s performance. The best performing ArM [Cp*Ir(Boc-AQ-biot)Cl] **10** · Sav WT included a bulky carbamate moiety (Table 1, entry 8). In all cases, perfect regioselectivity for the nitrene insertion was observed in favor of γ-lactam **2**. However, the enantioselectivity (ee) was modest, varying between 3% and 30%, as shown in Table 1. Importantly, the free cofactor afforded significantly lower TONs than the corresponding ANIase [Cp*Ir(Boc-AQ-biot)Cl] **10** · Sav WT, highlighting the beneficial influence of the protein scaffold (see Table 1, entries 8–9). Having identified a promising ANIase, we set out to further optimize the catalytic performance by genetic means in the presence of [Cp*Ir(Boc-AQ-biot)Cl] **10**.

**Table 1:**
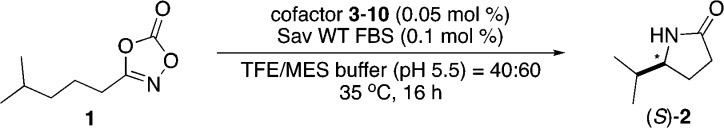
Selected Results for the Chemical Optimization of [Cp*Ir(biot-AQ)Cl] · Sav WT for the Enantioselective Synthesis of γ-Lactam 2^a^

entry	ANIase	TON^b^	ee (%)^b^
1	[Cp*Ir(H_2_NCH_2_CH_2_NTos-biot)Cl] **3** · Sav WT	4.1 ± 1.2	5 ± 3.1
2	[Cp*Ir(biot-AQ)Cl] **4** · Sav WT	6.5 ± 0.7	8 ± 3.2
3^c^	[Biot-Cp*Ir(AQ)Cl] **5** · Sav WT	6.5 ± 1.1	3 ± 1.4
4^c^	[Biot-Cp*Ir(AQ-OMe)Cl] **6** · Sav WT	6.2 ± 0.8	3 ± 2.3
5	[Cp*Ir(Ac-AQ-biot)Cl] **7** · Sav WT	8.8 ± 0.6	6 ± 2.3
6	[Cp*Ir(Phoc-AQ-biot)Cl] **8** · Sav WT	8.5 ± 0.5	4 ± 2.0
7	[Cp*Ir(Meoc-AQ-biot)Cl] **9** · Sav WT	21.4 ± 0.6	4 ± 0.4
8	[Cp*Ir(Boc-AQ-biot)Cl] **10** · Sav WT	47.6 ± 1.0	30 ± 0.8
9	[Cp*Ir(Boc-AQ-biot)Cl] **10**	14 ± 0.7	–

a Standard conditions: dioxazolone [**1**] = 15 mM, [Cp*Ir cofactor] = 7.5 μM, [Sav WT, FBS] = 15 μM, 200 μL of TFE, 300 μL of MES buffer (0.1 M, pH 5.5), 35 °C, 16 h under air.

b Determined by chiral GC using 1,3,5-trimethoxybezene as internal standard.

c The Biot-Cp*Ir cofactor was prepared *in situ* by incubating [Biot-Cp*IrCl_2_]_2_^54^ and corresponding 8-aminoquinoline derivative in TFE at 35 °C for 2 h.^54,55^ Sav WT FBS = Sav WT free biotin-binding sites. TFE = trifluoroethanol. MES = 2-(*N*-morpholino)ethanesulfonic acid (see Supporting Information for a detailed experimental procedure).
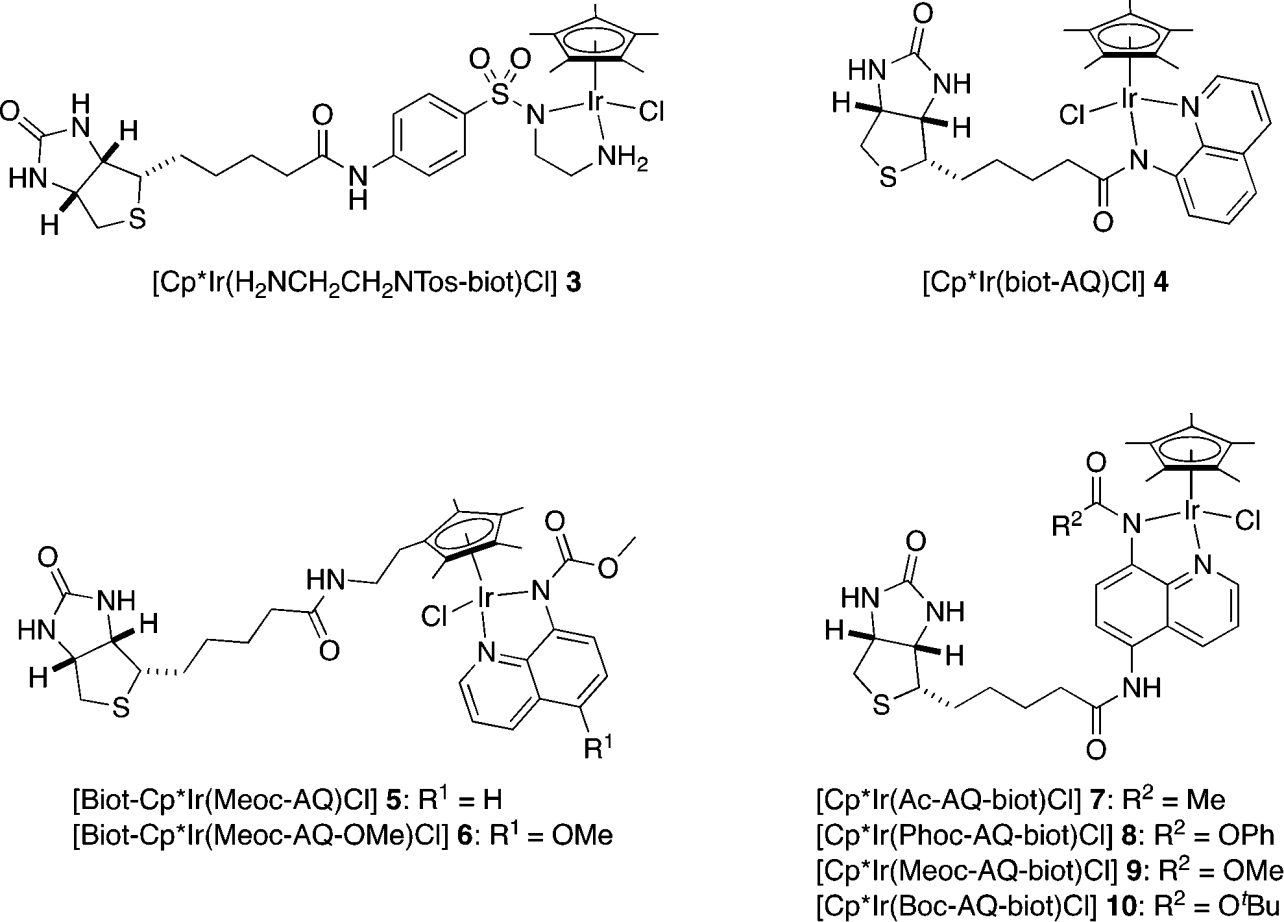

**Figure 1:**
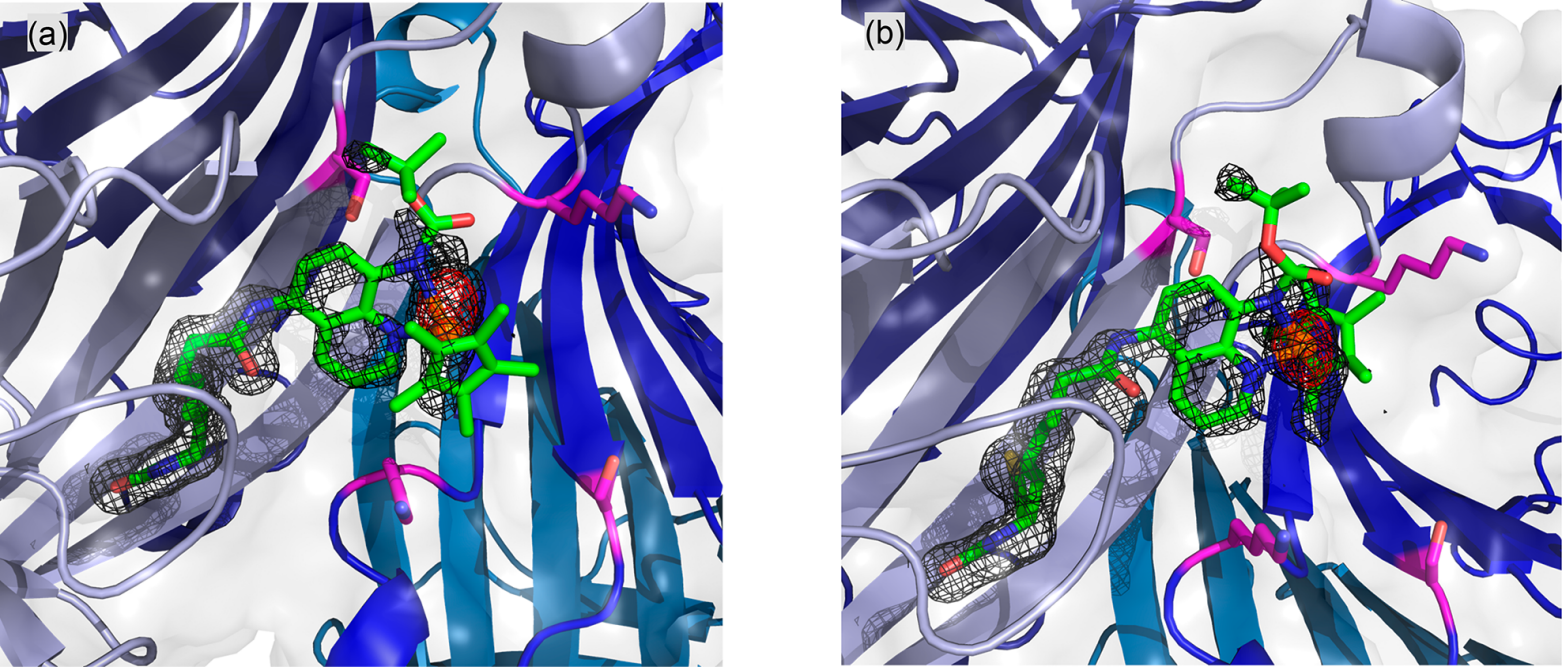
Crystal structure of [Cp*Ir(Boc-AQ-biot)Cl] **10** · Sav WT (PDB: 8BY1). The [Cp*Ir(Boc-AQ-biot)Cl] **10** is represented as sticks (with color-coded atoms; nitrogen = blue, oxygen = red, carbon = green, and sulfur = yellow) with the Ir as an orange sphere. The protein is represented as a cartoon and surface model. The monomers are color-coded in different shades of blue. The residues S112 and K121 are displayed as purple sticks (nitrogen = blue, oxygen = red, and carbon = purple). The 2F_o_−F_c_ difference map is displayed as a dark gray mesh (1σ), and the anomalous electron density is displayed as a red mesh (8σ). The occupancy of the Iridium was set to 50 (a) and 30% (b), respectively.

To identify the position of the cofactor upon incorporation in Sav WT, we determined the crystal structure of [Cp*Ir(Boc-AQ-biot)Cl] **10** · Sav WT (PDB: 8BY1), Figure 1. Both Ir_(*R*)_- and Ir_(*S*)_-configurations of [Cp*Ir(Boc-AQ-biot)Cl] **10** were observed, and the position of the metal center was slightly different, depending on its absolute configuration. The closest lying residues include S112 and K121 (Cβ^112^–Ir = 5.6 and 6.7 Å and Cβ^121^–Ir = 7.3 and 8.5 Å and 7.2 and 7.0 Å for the neighboring Sav monomer).

**Figure 2:**
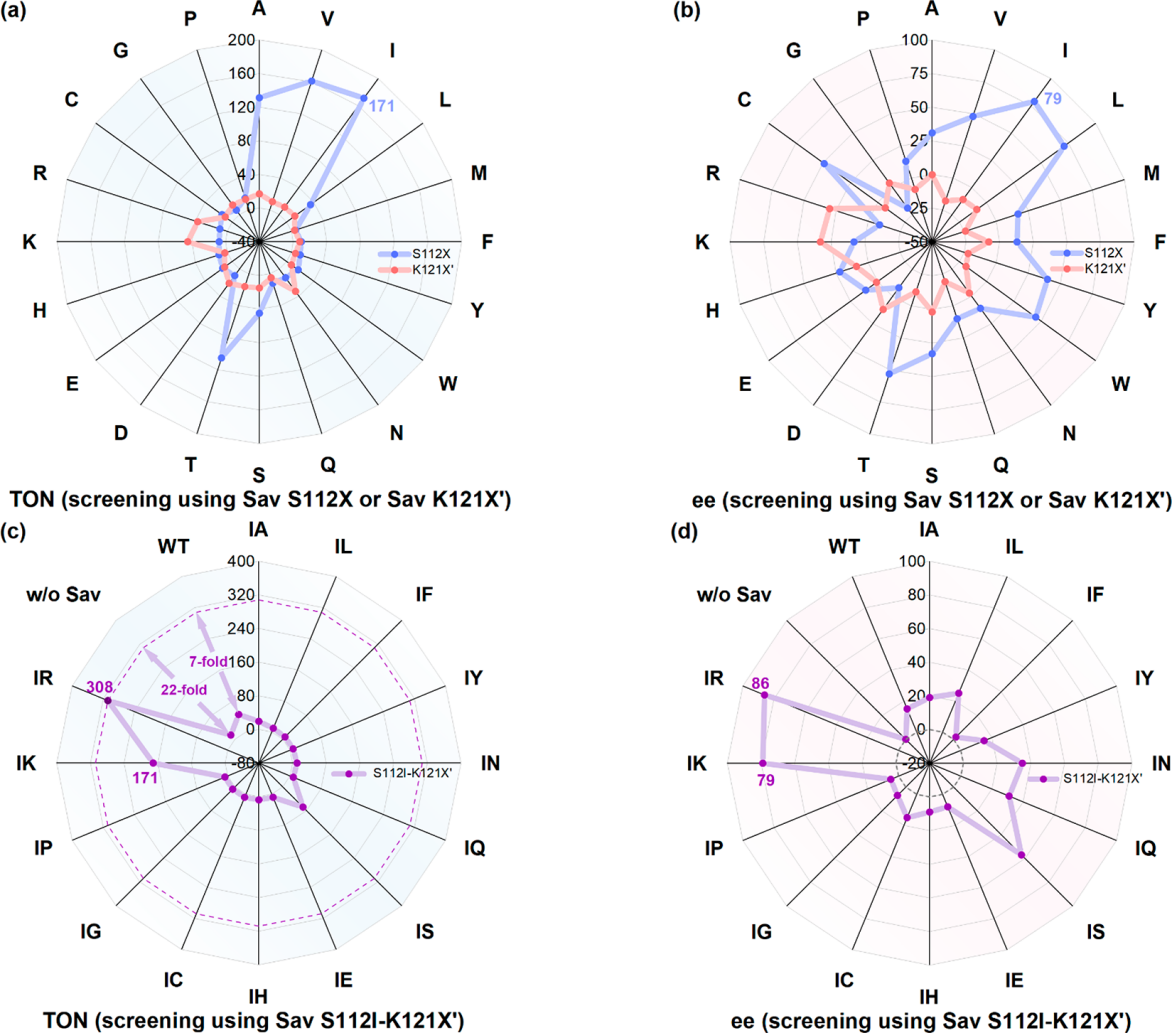
Results for genetic optimization using [Cp*Ir(Boc-AQ-biot)Cl] **10**: (a) TON and (b) ee for reactions using single mutants at positions S112X or K121X′; (c) TON and (d) ee for reactions using double mutants Sav S112I-K121X′. Results for reactions using single mutants Sav S112X, Sav K121X′ and double mutants Sav S112I-K121X′ are highlighted in blue, red, and violet, respectively. Double mutants Sav S112I-K121X′ are abbreviated as IX′ in (c) and (d).

Next, we screened single saturation mutagenesis libraries resulting from randomization at position S112X or K121X′ to afford 38 corresponding single mutant ANIases. The screening results are summarized in Figure 2a–b. The following trends are apparent:

i) Residues at position Sav S112X have a more pronounced influence on ANIase’s activity and enantioselectivity than residues at position Sav K121X′.
ii) Mutations at position Sav K121X′ have a modest influence on ANIase. Both lysine and arginine lead to slightly better performance, in terms of both activity and enantioselectivity, than other single mutants at K121X′.
iii) Introduction of a hydrophobic residue at position S112X has the most pronounced positive effect on both activity and selectivity. The best performing single mutant ANIase [Cp*Ir(Boc-AQ-biot)Cl] **10** · Sav S112I affords (*S*)-2 in TON 171 and 79% ee. The structurally related, but smaller, Sav S112V- or Sav S112L-ANIases lead to a pronounced decrease in ee or TON, respectively

Based on this initial screen, we selected [Cp*Ir(Boc-AQ-biot)Cl] **10** · Sav S112I and introduced a second mutation at position K121X′. Unfortunately, only 13 double mutant variants were obtained after expression and purification, which were used for the screening, Figure 2c–d. From this screening, it appears that combining the best single-point mutants at both S112 and K121 (i.e., S112I with K121R) leads to significantly improved ANIase performance. Indeed, [Cp*Ir(Boc-AQ-biot)Cl] **10** · Sav S112I-K121R clearly outperforms its parent single mutant (TON = 308, 86% ee compared to TON 171 and 79% ee). Compared to the wild-type ANIase, the [Cp*Ir(Boc-AQ-biot)Cl] **10** · Sav S112I-K121R double mutant displays 6-fold higher TONs; see Table S2 for a complete list of results.

**Figure 3:**
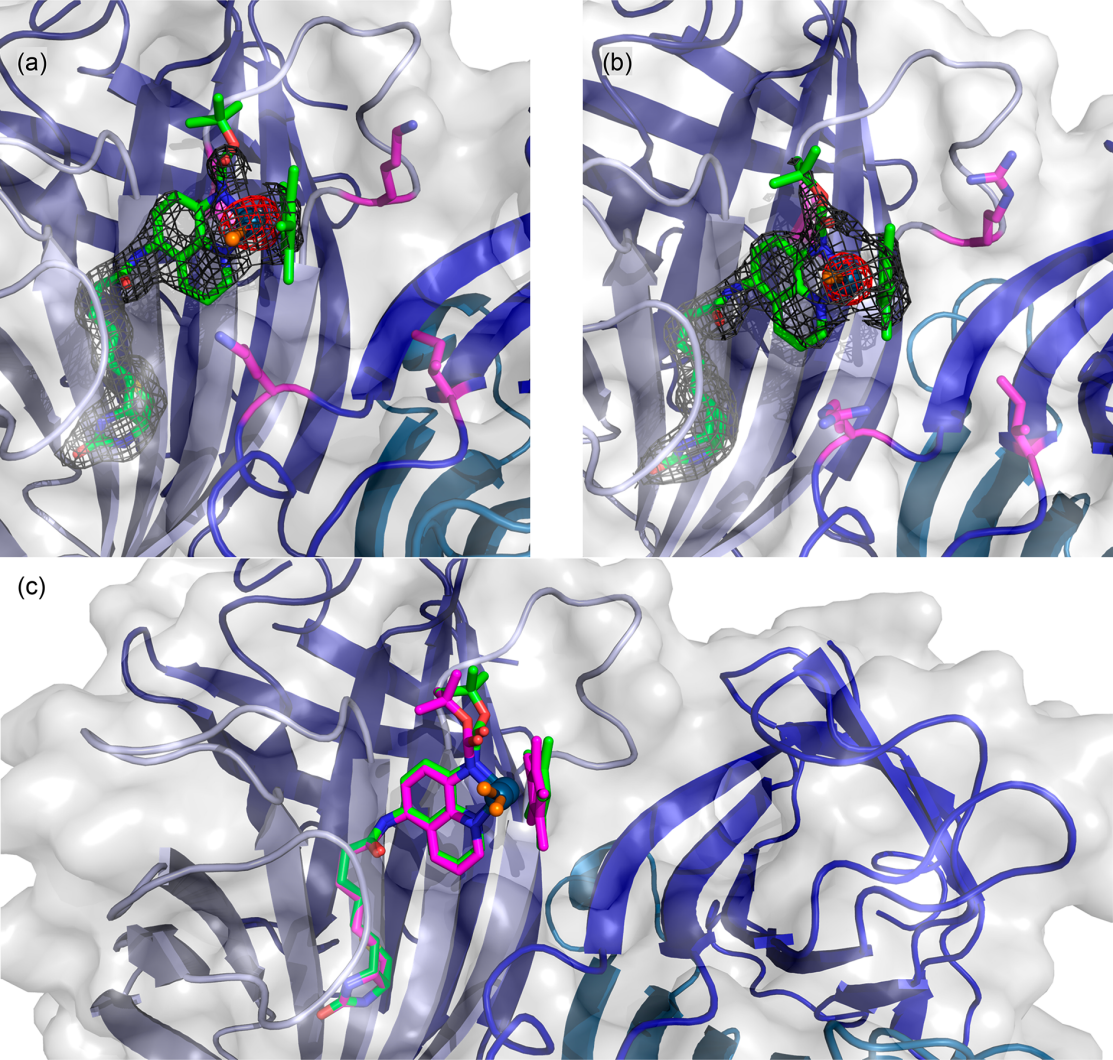
Structural characterization of the best-performing ANIases. (a) Crystal structure of [Cp*Ir(Boc-AQ-biot)Cl] **10** · Sav S112I (PDB: 8AQX). (b) Crystal structure of [Cp*Ir(Boc-AQ-biot)Cl] **10** · Sav S112I-K121R (PDB: 8BY0). The [Cp*Ir(Boc-AQ-biot)Cl] **10** is represented as green sticks (atoms are color-coded; nitrogen = blue, oxygen = red, carbon = green, chloride = orange, and sulfur = yellow) with the Ir as a dark blue sphere. The protein is represented as both a cartoon and transparent surface models. The monomers are color-coded in different shades of blue. The residues S112 and K121 are displayed as sticks (atoms are color-coded; nitrogen = blue, oxygen = red, and carbon = purple). The 2F_o_–F_c_ difference map is displayed as a dark gray mesh (1σ), and the anomalous electron density is displayed as a red mesh (8σ). The occupancy of the iridium was set to 70%. (c) Superposition of both crystal structures (PDB: 8AQX and 8BY0). The [Cp*Ir(Boc-AQ-biot)Cl] **10** of Sav S112I is represented as green sticks with the Ir as a dark blue sphere, whereas in the case of Sav S112I-K121R, it is represented as purple sticks with the Ir as a dark blue sphere.

To gain insight into the influence of the second sphere on ANIase performance, we determined the structures of ANIases [Cp*Ir(Boc-AQ-biot)Cl] **10** · Sav S112I and [Cp*Ir(Boc-AQ-biot)Cl] **10** · Sav S112I-K121R by crystallography. Based on the collected data sets, the absolute configurations of [Cp*Ir(Boc-AQ-biot)Cl] **10** · Sav S112I and [Cp*Ir(Boc-AQ-biot)Cl] **10** · Sav S112I-K121R could be determined unambiguously. In both cases, modeling of the cofactor into the residual electron density in the F_o_–F_c_ map projected the iridium in the position of the anomalous density peak, and the configuration could be identified as Ir_(*S*)_-[Cp*Ir(Boc-AQ-biot)Cl] **10**, Figure 3. The screening results reveal that the basicity of the residue at position K121 plays an important role in selectivity, next to steric bulkiness. The more basic arginine leads to higher enantioselectivity.

Upon decreasing the temperature to 10 °C, both the TON and the ee were positively affected: in the presence of [Cp*Ir(Boc-AQ-biot)Cl] **10** · Sav S112I-K121R, dioxazolone **1** was converted to γ-lactam (*S*)-**2**, in 363 TON and 89% ee, Table S3, entry 9. Next, we evaluated the substrate scope using a focused library of Sav mutants (including Sav S112I, Sav S112I-K121R, Sav S112V, and Sav S112V-K121R) combined with either [Cp*Ir(Boc-AQ-biot)Cl] **10** or [Cp*Ir(Meoc-AQ-biot)Cl] **9**, Table 2.

**Table 2:**
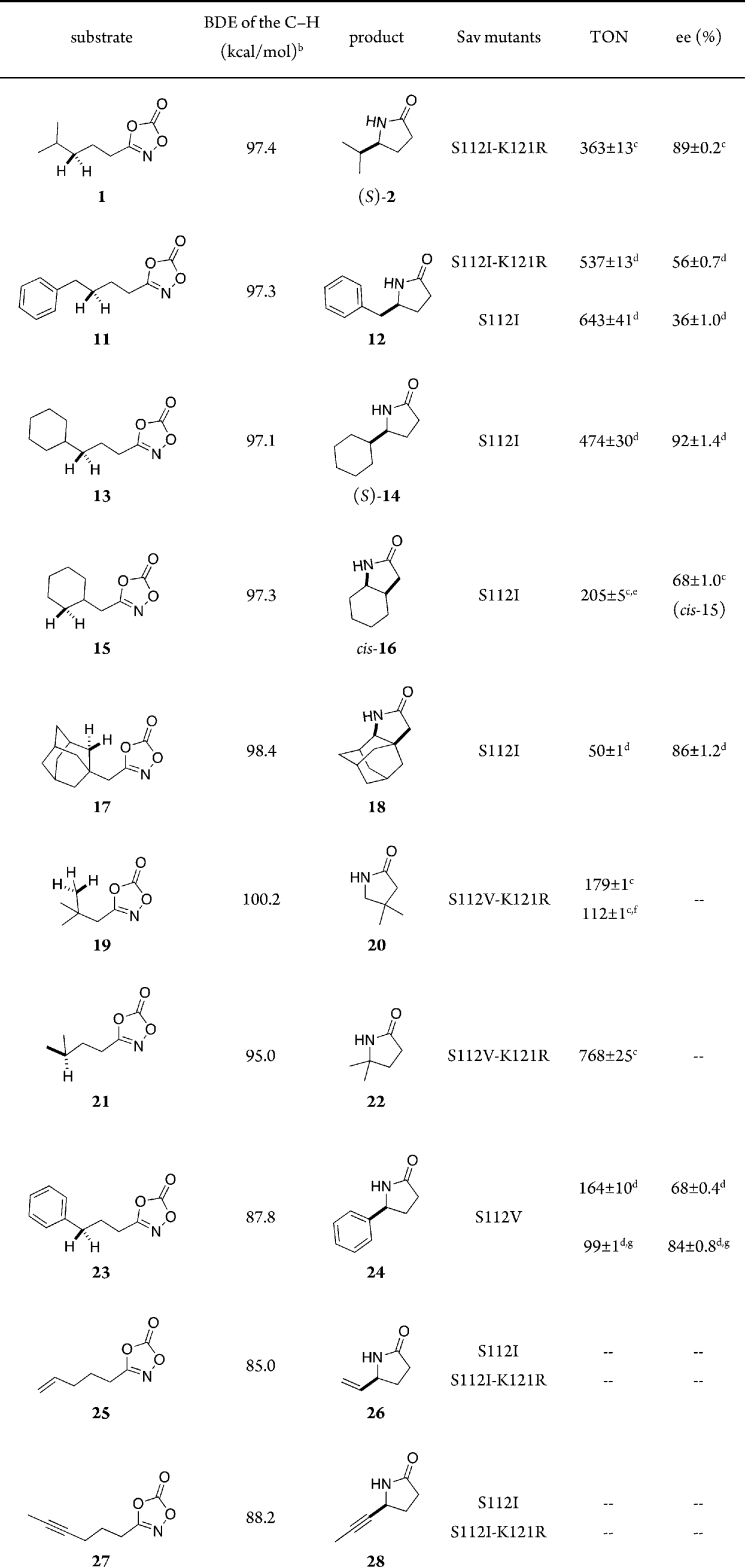
Substrate Scope for the Enantioselective C–H Amidation To Afford γ-Lactams Catalyzed by [Cp*Ir(Boc-AQ-biot)Cl] 10 · Sav Variants^a^

a Conditions: [dioxazolone] = 15 mM, [[Cp*Ir(Boc-AQ-biot)Cl] **10**] = 7.5 μM, [Sav FBS] = 15 μM, 200 μL of TFE, 300 μL of MES buffer (0.1 M, pH 5.5), 10 °C, 48 h under air.

b Bond dissociation energy (BDE) computed with the BDE estimator at https://bde.ml.nrel.gov.

c TON and ee were determined by chiral GC.

d TON and ee was determined by SFC.

e Total turnover number for both *cis*- and *trans*-**16**, *cis*-/*trans*-**16** = 86:14.

f [[Cp*Ir(Boc-AQ-biot)Cl] **10**] = 75 μM, [Sav FBS] = 150 μM were used.

g [Cp*Ir(Meoc-AQ-biot)Cl] **9** · Sav S112V was used.

Various alkyldioxazolones bearing unactivated C(*sp*^3^)–H bonds were tested in the presence of the ANIAse. Several noteworthy observations arose from the screening:

i) γ-Lactams were formed exclusively in excellent TON in the presence of the ANIase. Despite the presence of activated benzylic C–H bonds, no δ-lactam was observed for substrate **11**.

ii) The bond dissociation energy (BDE) of the reactive C–H bond does not correlate with the TON. The BDE values of the C–H bonds varied from roughly 85 to 100 kcal/mol. However, the substrate with the highest and lowest BDEs–i.e. substrates **19** and **23**– afforded comparable TON. Other substrates bearing moderate-BDE C–H bonds gave similar TONs, except for the poorly soluble substrate **17**.
iii) Among the evaluated substrates, substrate **13** afforded the corresponding lactam **14** with the highest ee (92% ee and 474 TONs) in the presence of ANIase [Cp*Ir(Boc-AQ-biot)Cl] **10** · Sav S112I. The same enantioselectivity was observed on a 100 mg preparative experiment using 0.5 mol % ANIase [Cp*Ir(Boc-AQ-biot)Cl] **10** · Sav S112I, in 83% yield. A second preparative experiment using 200 mg of substrate **1** afforded γ-lactam **2** in 55% yield (0.5 mol % ANIase, i.e. 110 TON) and 91% ee; see Supporting Information.
iv) To identify side-products, a semipreparative experiment using substrate **13** (45 μmol, 9.5 mg) was performed. Analysis of the crude of the reaction by ^1^H NMR revealed >90% yield for γ-lactam **14**. The only side-product identified was the corresponding hydroxamate, resulting from nitrene insertion into water (see SI, p. S25).
v) Strikingly, no conversion was observed for either allylic and propargylic C–H insertion (Table 2, substrate **25** and **27**).

**Figure 4:**
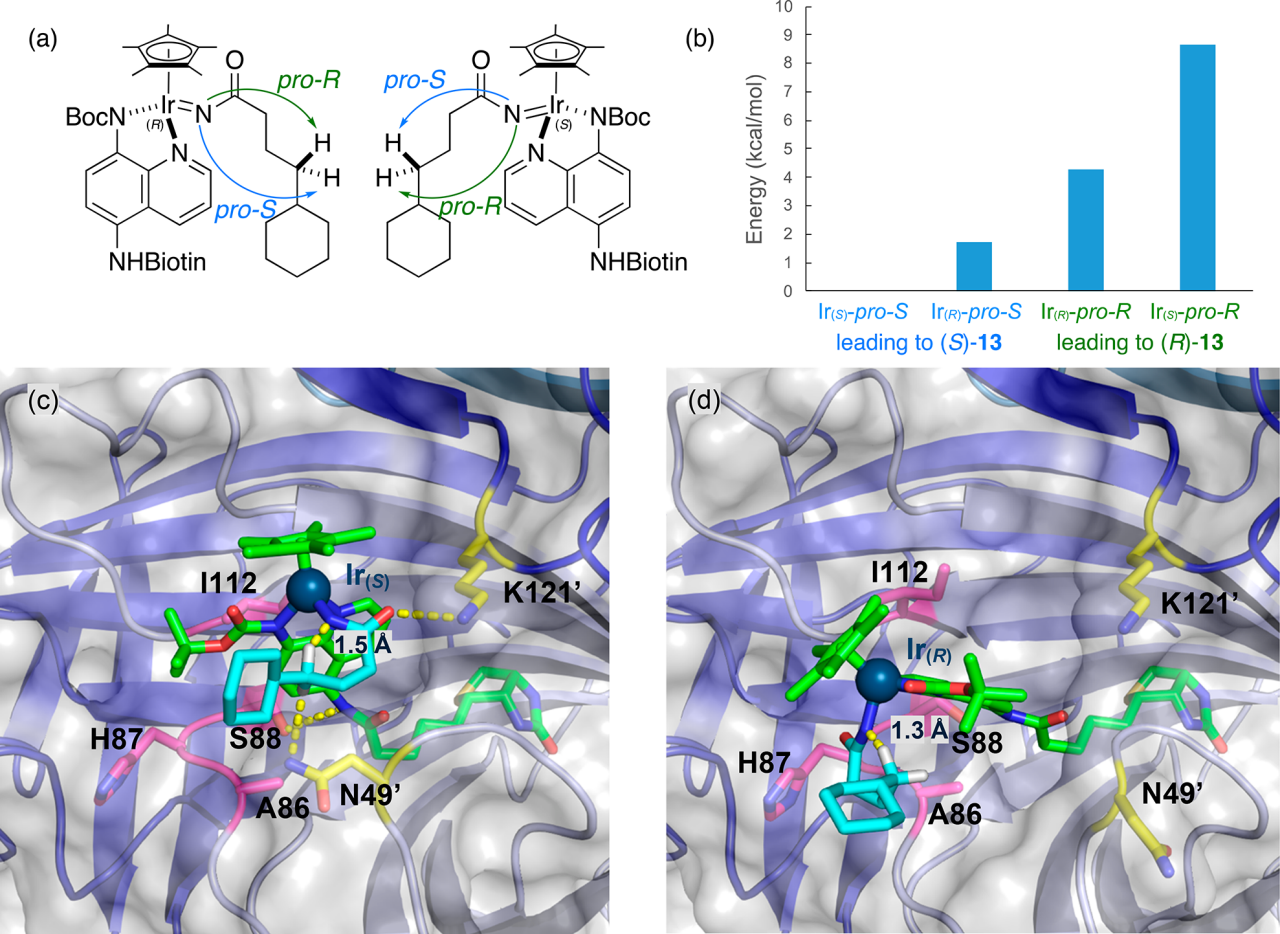
QM-MM Computation of the reaction paths leading to enantiopure **14**. (a) Four diastereotopic C–H amidation paths lead to both enantiomers of γ-lactam **14**. (b) Computed relative energy values for the four diastereotopic transition states. (c) Close-up of the lowest energy transition state with Ir_(*S*)_ interacting with the *pro*-*S* C–H bond leading to (*S*)-**14**. (d) Close-up view of the lowest lying transition state with Ir_(*R*)_ interacting with the *pro*-*R* C–H bond leading to (*R*)-**14**. The cofactor is displayed as color-coded sticks (nitrogen = blue, oxygen = red, and carbon = green) with Ir displayed as dark blue sphere. The nitrene moiety is represented by color-coded sticks (carbon = cyan). Close-lying residues that interact with the transition states are represented as color-coded sticks (for residues interacting with substrate and cofactor, carbon = yellow and magenta respectively). Critical interactions between the host protein and the four diastereotopic transition states are presented in Figure S1.

To study the origin of the good enantioselectivity, transition states for the conversion of substrate **13** in the presence of ANIase [Cp*Ir(Boc-AQ-biot)Cl] **10** · Sav S112I were modeled by QM-MM, including solvation. Computational details are collected in the Supporting Information. The four possible transition states resulting from the two pseudo-enantiomers at Ir for [Cp*Ir(Boc-AQ-biot)Cl] **10** · Sav S112I with the two prochiral C–H bonds of substrate **13** were computed, Figure 4. The two most stable transition states are Ir_(*S*)_-*pro*-*S* (Figure 4c) and Ir_(*R*)_-*pro*-*S* which both lead to (*S*)-**14**. The lowest-lying transition state that affords (*R*)-**14** (i.e., Ir_(*R*)_-*pro*-*R*, Figure 4d) lies 4.27 and 2.55 kcal/mol above both transition states that afford (*S*)-**14**, Figure 4b. In the Ir_(*S*)_-*pro*-*S* transition state, the conformation of the alkyl chain projects the *pro*-*S* C–H bond within 1.5 Å of the nitrene moiety (compared to 3.0 Å for the *pro-R* C–H bond), Figure 4c. Comparable C–H····N distances are observed for the Ir_(*R*)_-*pro*-*R* transition state, albeit for the opposite diastereotopic C–H groups, thus leading to the formation of (*R*)-**14**. Residue Sav K121 lies at 3.0 and 12.2 Å of the oxygen of the carbonyl of the substrate for both transition states displayed in Figure 4c–d. As observed in the mutation studies at this position, the presence of either a lysine or an arginine is critical for both high TON and ee, Figure 2. We hypothesize that residue Sav K121 (or K121R) forms a H-bond with the carbonyl moiety of the substrate in the lowest energy transition state leading to (*S*)-**14**. As this interaction is absent in the lowest energy transition state leading to (*R*)-**14**, we surmise that a H-bond between a cationic residue at position 121 and the carbonyl moiety of the substrate significantly contributes to favoring the formation of (*S*)-**14** in high yield. For the lowest-lying transition state (e.g., Ir_(*S*)_-*pro*-*S*) leading to (*S*)-**14**, close contacts between the protein and the nitrene-bound substrate include amino acids N49, A86, H87, S88, and I112. No such close contacts between the protein and the nitrene moiety are apparent in the Ir_(*R*)_-*pro*-*R* transition state, leading to (*R*)-**14**. Interestingly, any mutation at Sav G48 markedly reduces the TON, as shown in Table S13. We hypothesize that bulkier amino acids at this position prevent the γ-CH_2_ group of the nitrene from approaching the Ir=N moiety, thus preventing the C–H insertion that leads to the formation of γ-lactam **14**.

## 3 Conclusion

In summary, we have developed a versatile ANIase that is highly efficient for nitrene insertion into unactivated aliphatic primary, secondary, and tertiary C(*sp*^3^)–H bonds. Strikingly, and despite a lower BDE, allylic and propargylic C–H bonds are not subject to functionalization. By screening a small library of biotinylated Cp*Ir-cofactors in the presence of Sav WT, we identified [Cp*Ir(Boc-AQ-biot)Cl] **10** as the most efficient. Iterative saturation mutagenesis led to the identification of Sav S112I-K121R as a versatile host for the artificial nitrene insertase. Interestingly, the single crystal X-ray structure of [Cp*Ir(Boc-AQ-biot)Cl] **10** · Sav S112I-K121R revealed the preferential incorporation of the Ir_(*S*)_-cofactor. QM-MM calculations suggest that this pseudo-enantiomer leads to the most stable transition state to afford enantioenriched (*S*)-γ-lactam **14**.

The ANIase presented herein catalyzes the enantioselective intramolecular nitrene insertion into various C(*sp*^3^)–H bonds, with BDEs up to 100 kcal/mol. Accordingly, it complements Ir-based homogeneous catalysts which perform best with benzylic C–H bonds.

Current efforts aiming toward intermolecular C–H amidation will contribute to expand the potential applications of ANIase. We surmise that second coordination sphere interactions between the substrates and the host protein may increase the effective molarity of both substrates to afford enantioenriched high-added value amides.

## Supporting Information Available

General information, experimental section, figures, schemes, tables, and X-ray reports (PDF)

## References

[ref1] Ricci A.; Wiley I. Amino group chemistry: from synthesis to the life sciences; Wiley-VCH Verlag GmbH & Co. KGaA: 2008.

[ref2] van Vliet K. M.; de Bruin B. Dioxazolones: Stable Substrates for the Catalytic Transfer of Acyl Nitrenes. ACS Catal. 2020, 10, 4751–4769. 10.1021/acscatal.0c00961.

[ref3] Park Y.; Kim Y.; Chang S. Transition Metal-Catalyzed C-H Amination: Scope, Mechanism, and Applications. Chem. Rev. 2017, 117, 9247–9301. 10.1021/acs.chemrev.6b00644. 28051855

[ref4] Forero-Cortes P. A.; Haydl A. M. The 25th Anniversary of the Buchwald-Hartwig Amination: Development, Applications, and Outlook. Org. Process Res. Dev. 2019, 23, 1478–1483. 10.1021/acs.oprd.9b00161.

[ref5] Breslow R.; Gellman S. H. Intramolecular Nitrene C-H Insertions Mediated by Transition-Metal Complexes as Nitrogen Analogs of Cytochrome-P-450 Reactions. J. Am. Chem. Soc. 1983, 105, 6728–6729. 10.1021/ja00360a039.

[ref6] Du B. N.; Chan C. M.; Au C. M.; Yu W. Y. Transition Metal-Catalyzed Regioselective Direct C-H Amidation: Interplay between Inner- and Outer-Sphere Pathways for Nitrene Cross-Coupling Reactions. Acc. Chem. Res. 2022, 55, 2123–2137. 10.1021/acs.accounts.2c00283. 35853135

[ref7] Liu Y. E.; Shing K. P.; Lo V. K. Y.; Che C. M. Iron- and Ruthenium-Catalyzed C-N Bond Formation Reactions. Reactive Metal Imido/Nitrene Intermediates. ACS. Catal. 2023, 13, 1103–1124. 10.1021/acscatal.2c04830.

[ref8] Hong S. Y.; Hwang Y.; Lee M.; Chang S. Mechanism-Guided Development of Transition-Metal-Catalyzed C-N Bond-Forming Reactions Using Dioxazolones as the Versatile Amidating Source. Acc. Chem. Res. 2021, 54, 2683–2700. 10.1021/acs.accounts.1c00198. 33979133

[ref9] Ju M.; Schomaker J. M. Nitrene transfer catalysts for enantioselective C-N bond formation. Nat. Rev. Chem. 2021, 5, 580–594. 10.1038/s41570-021-00291-4. 37117585

[ref10] Dequirez G.; Pons V.; Dauban P. Nitrene chemistry in organic synthesis: still in its infancy?. Angew. Chem., Int. Ed. 2012, 51, 7384–7395. 10.1002/anie.201201945. 22730346

[ref11] Roizen J. L.; Harvey M. E.; Du Bois J. Metal-Catalyzed Nitrogen-Atom Transfer Methods for the Oxidation of Aliphatic C-H Bonds. Acc. Chem. Res. 2012, 45, 911–922. 10.1021/ar200318q. 22546004 PMC5483381

[ref12] Brandenberg O. F.; Fasan R.; Arnold F. H. Exploiting and engineering hemoproteins for abiological carbene and nitrene transfer reactions. Curr. Opin. in Biotechnol. 2017, 47, 102–111. 10.1016/j.copbio.2017.06.005. PMC5617781 28711855

[ref13] Ye Y.; Cao J.; Oblinsky D. G.; Verma D.; Prier C. K.; Scholes G. D.; Hyster T. K. Using enzymes to tame nitrogen-centered radicals for enantioselective hydroamination. Nat. Chem. 2023, 15, 206–212. 10.1038/s41557-022-01083-z. 36376390 PMC10859868

[ref14] Yang Y.; Arnold F. H. Navigating the Unnatural Reaction Space: Directed Evolution of Heme Proteins for Selective Carbene and Nitrene Transfer. Acc. Chem. Res. 2021, 54, 1209–1225. 10.1021/acs.accounts.0c00591. 33491448 PMC7931446

[ref15] Hayashi H.; Uchida T. Nitrene Transfer Reactions for Asymmetric C-H Amination: Recent Development. Eur. J. Org. Chem. 2020, 2020, 909–916. 10.1002/ejoc.201901562.

[ref16] Hong S. Y.; Park Y.; Hwang Y.; Kim Y. B.; Baik M. H.; Chang S. Selective formation of gamma-lactams via C-H amidation enabled by tailored iridium catalysts. Science 2018, 359, 1016–1021. 10.1126/science.aap7503. 29496875

[ref17] Park Y.; Chang S. Asymmetric formation of γ-lactams via C–H amidation enabled by chiral hydrogen-bond-donor catalysts. Nat. Catal. 2019, 2, 219–227. 10.1038/s41929-019-0230-x.

[ref18] Xing Q.; Chan C. M.; Yeung Y. W.; Yu W. Y. Ruthenium(II)-Catalyzed Enantioselective gamma-Lactams Formation by Intramolecular C-H Amidation of 1,4,2-Dioxazol-5-ones. J. Am. Chem. Soc. 2019, 141, 3849–3853. 10.1021/jacs.9b00535. 30785737

[ref19] Wang H.; Park Y.; Bai Z.; Chang S.; He G.; Chen G. Iridium-Catalyzed Enantioselective C(sp(3))-H Amidation Controlled by Attractive Noncovalent Interactions. J. Am. Chem. Soc. 2019, 141, 7194–7201. 10.1021/jacs.9b02811. 30978019

[ref20] Zhou Z.; Chen S.; Hong Y.; Winterling E.; Tan Y.; Hemming M.; Harms K.; Houk K. N.; Meggers E. Non-C2-Symmetric Chiral-at-Ruthenium Catalyst for Highly Efficient Enantioselective Intramolecular C(sp(3))-H Amidation. J. Am. Chem. Soc. 2019, 141, 19048–19057. 10.1021/jacs.9b09301. 31751132

[ref21] Kweon J.; Chang S. Highly Robust Iron Catalyst System for Intramolecular C(sp(3))-H Amidation Leading to gamma-Lactams. Angew. Chem., Int. Ed. 2021, 60, 2909–2914. 10.1002/anie.202013499. 33111492

[ref22] Singh R.; Bordeaux M.; Fasan R. P450-Catalyzed Intramolecular sp3 C-H Amination with Arylsulfonyl Azide Substrates. ACS Catal. 2014, 4, 546–552. 10.1021/cs400893n. 24634794 PMC3949735

[ref23] Steck V.; Kolev J. N.; Ren X. K.; Fasan R. Mechanism-Guided Design and Discovery of Efficient Cytochrome P450-Derived C-H Amination Biocatalysts. J. Am. Chem. Soc. 2020, 142, 10343–10357. 10.1021/jacs.9b12859. 32407077 PMC7372717

[ref24] Fasan R.; Roy S.; Vargas D.; Ma P.; Sengupta A.; Houk K. Stereoselective Construction of β-, γ-, and δ-Lactam Rings via Enzymatic C–H Amidation. Research Square 2023-01-19 (accessed 2023-02-16). 10.21203/rs.3.rs-2429100/v1. PMC10997382 38584987

[ref25] Jeschek M.; Panke S.; Ward T. R. Artificial Metalloenzymes on the Verge of New-to-Nature Metabolism. Trends Biotechnol. 2018, 36, 60–72. 10.1016/j.tibtech.2017.10.003. 29061328

[ref26] Perez-Rizquez C.; Rodriguez-Otero A.; Palomo J. M. Combining enzymes and organometallic complexes: novel artificial metalloenzymes and hybrid systems for C-H activation chemistry. Org. Biomol. Chem. 2019, 17, 7114–7123. 10.1039/C9OB01091B. 31294731

[ref27] Wilson M. E.; Whitesides G. M. Conversion of a Protein to a Homogeneous Asymmetric Hydrogenation Catalyst by Site-Specific Modification with a Diphosphinerhodium(I) Moiety. J. Am. Chem. Soc. 1978, 100, 306–307. 10.1021/ja00469a064.

[ref28] Monnard F. W.; Nogueira E. S.; Heinisch T.; Schirmer T.; Ward T. R. Human carbonic anhydrase II as host protein for the creation of artificial metalloenzymes: the asymmetric transfer hydrogenation of imines. Chem. Sci. 2013, 4, 3269–3274. 10.1039/c3sc51065d.

[ref29] Oohora K.; Onoda A.; Hayashi T. Hemoproteins Reconstituted with Artificial Metal Complexes as Biohybrid Catalysts. Acc. Chem. Res. 2019, 52, 945–954. 10.1021/acs.accounts.8b00676. 30933477

[ref30] Mirts E. N.; Petrik I. D.; Hosseinzadeh P.; Nilges M. J.; Lu Y. A designed heme-[4Fe-4S] metalloenzyme catalyzes sulfite reduction like the native enzyme. Science 2018, 361, 1098–1101. 10.1126/science.aat8474. 30213908 PMC6650743

[ref31] Natoli S. N.; Hartwig J. F. Noble-Metal Substitution in Hemoproteins: An Emerging Strategy for Abiological Catalysis. Acc. Chem. Res. 2019, 52, 326–335. 10.1021/acs.accounts.8b00586. 30693758 PMC11620731

[ref32] Shoji O.; Aiba Y.; Watanabe Y. Hoodwinking Cytochrome P450BM3 into Hydroxylating Non-Native Substrates by Exploiting Its Substrate Misrecognition. Acc. Chem. Res. 2019, 52, 925–934. 10.1021/acs.accounts.8b00651. 30888147

[ref33] Lewis J. C. Beyond the Second Coordination Sphere: Engineering Dirhodium Artificial Metalloenzymes To Enable Protein Control of Transition Metal Catalysis. Acc. Chem. Res. 2019, 52, 576–584. 10.1021/acs.accounts.8b00625. 30830755

[ref34] Roelfes G. LmrR: A Privileged Scaffold for Artificial Metalloenzymes. Acc. Chem. Res. 2019, 52, 545–556. 10.1021/acs.accounts.9b00004. 30794372 PMC6427492

[ref35] Grimm A. R.; Sauer D. F.; Polen T.; Zhu L. L.; Hayashi T.; Okuda J.; Schwaneberg U. A Whole Cell E. coli Display Platform for Artificial Metalloenzymes: Poly(phenylacetylene) Production with a Rhodium-Nitrobindin Metalloprotein. ACS Catal. 2018, 8, 2611–2614. 10.1021/acscatal.7b04369.

[ref36] Lombardi A.; Pirro F.; Maglio O.; Chino M.; DeGrado W. F. De Novo Design of Four-Helix Bundle Metalloproteins: One Scaffold, Diverse Reactivities. Acc. Chem. Res. 2019, 52, 1148–1159. 10.1021/acs.accounts.8b00674. 30973707 PMC7362765

[ref37] Ward T. R. Artificial Metalloenzymes Based on the Biotin-Avidin Technology: Enantioselective Catalysis and Beyond. Acc. Chem. Res. 2011, 44, 47–57. 10.1021/ar100099u. 20949947

[ref38] Heinisch T.; Ward T. R. Artificial Metalloenzymes Based on the Biotin-Streptavidin Technology: Challenges and Opportunities. Acc. Chem. Res. 2016, 49, 1711–1721. 10.1021/acs.accounts.6b00235. 27529561

[ref39] Liang A. D.; Serrano-Plana J.; Peterson R. L.; Ward T. R. Artificial Metalloenzymes Based on the Biotin-Streptavidin Technology: Enzymatic Cascades and Directed Evolution. Acc. Chem. Res. 2019, 52, 585–595. 10.1021/acs.accounts.8b00618. 30735358 PMC6427477

[ref40] Reetz M. T. Directed Evolution of Artificial Metalloenzymes: A Universal Means to Tune the Selectivity of Transition Metal Catalysts?. Acc. Chem. Res. 2019, 52, 336–344. 10.1021/acs.accounts.8b00582. 30689339

[ref41] Schwizer F.; Okamoto Y.; Heinisch T.; Gu Y. F.; Pellizzoni M. M.; Lebrun V.; Reuter R.; Kohler V.; Lewis J. C.; Ward T. R. Artificial Metalloenzymes: Reaction Scope and Optimization Strategies. Chem. Rev. 2018, 118, 142–231. 10.1021/acs.chemrev.7b00014. 28714313

[ref42] Chen K.; Arnold F. H. Engineering new catalytic activities in enzymes. Nat. Catal. 2020, 3, 203–213. 10.1038/s41929-019-0385-5.

[ref43] Pamies O.; Dieguez M.; Backvall J. E. Artificial Metalloenzymes in Asymmetric Catalysis: Key Developments and Future Directions. Adv. Synth. Catal. 2015, 357, 1567–1586. 10.1002/adsc.201500290.

[ref44] Upp D. M.; Lewis J. C. Selective C-H bond functionalization using repurposed or artificial metalloenzymes. Curr. Opin. Chem. Biol. 2017, 37, 48–55. 10.1016/j.cbpa.2016.12.027. 28135654 PMC7437473

[ref45] Davis H. J.; Ward T. R. Artificial Metalloenzymes: Challenges and Opportunities. ACS Cent. Sci. 2019, 5, 1120–1136. 10.1021/acscentsci.9b00397. 31404244 PMC6661864

[ref46] Lewis J. C. Artificial Metalloenzymes and Metallopeptide Catalysts for Organic Synthesis. ACS Catal. 2013, 3, 2954–2975. 10.1021/cs400806a.

[ref47] Churchfield L. A.; Tezcan F. A. Design and Construction of Functional Supramolecular Metalloprotein Assemblies. Acc. Chem. Res. 2019, 52, 345–355. 10.1021/acs.accounts.8b00617. 30698941

[ref48] Mirts E. N.; Bhagi-Damodaran A.; Lu Y. Understanding and Modulating Metalloenzymes with Unnatural Amino Acids, Non-Native Metal Ions, and Non-Native Metallocofactors. Acc. Chem. Res. 2019, 52, 935–944. 10.1021/acs.accounts.9b00011. 30912643 PMC6642817

[ref49] Mayer C.; Hilvert D. A Genetically Encodable Ligand for Transfer Hydrogenation. Eur. J. Org. Chem. 2013, 2013, 3427–3431. 10.1002/ejoc.201300340.

[ref50] Mayer C.; Gillingham D. G.; Ward T. R.; Hilvert D. An artificial metalloenzyme for olefin metathesis. Chem. Commun. 2011, 47, 12068–12070. 10.1039/c1cc15005g. 21991583

[ref51] Koebke K. J.; Pecoraro V. L. Noncoded Amino Acids in de Novo Metalloprotein Design: Controlling Coordination Number and Catalysis. Acc. Chem. Res. 2019, 52, 1160–1167. 10.1021/acs.accounts.9b00032. 30933479 PMC6533121

[ref52] Wang Y. J.; Xue P.; Cao M. F.; Yu T. H.; Lane S. T.; Zhao H. M. Directed Evolution: Methodologies and Applications. Chem. Rev. 2021, 121, 12384–12444. 10.1021/acs.chemrev.1c00260. 34297541

[ref53] Zimbron J. M.; Heinisch T.; Schmid M.; Hamels D.; Nogueira E. S.; Schirmer T.; Ward T. R. A Dual Anchoring Strategy for the Localization and Activation of Artificial Metalloenzymes Based on the Biotin-Streptavidin Technology. J. Am. Chem. Soc. 2013, 135, 5384–5388. 10.1021/ja309974s. 23496309

[ref54] Quinto T.; Schwizer F.; Zimbron J. M.; Morina A.; Kohler V.; Ward T. R. Expanding the Chemical Diversity in Artificial Imine Reductases Based on the Biotin- Streptavidin Technology. ChemCatChem. 2014, 6, 1010–1014. 10.1002/cctc.201300825.

[ref55] Facchetti G.; Rimoldi I. 8-Amino-5,6,7,8-tetrahydroquinoline in iridium(III) biotinylated Cp* complex as artificial imine reductase. New J. Chem. 2018, 42, 18773–18776. 10.1039/C8NJ04558E.

[ref56] Lin C. C.; Lin C. W.; Chan A. S. C. Catalytic hydrogenation of itaconic acid in a biotinylated Pyrphos-rhodium(I) system in a protein cavity. Tetrahedron: Asymmetry 1999, 10, 1887–1893. 10.1016/S0957-4166(99)00193-7.

[ref57] Letondor C.; Pordea A.; Humbert N.; Ivanova A.; Mazurek S.; Novic M.; Ward T. R. Artificial transfer hydrogenases based on the biotin-(strept)avidin technology: Fine tuning the selectivity by saturation mutagenesis of the host protein. J. Am. Chem. Soc. 2006, 128, 8320–8328. 10.1021/ja061580o. 16787096

[ref58] Santi N.; Morrill L. C.; Swiderek K.; Moliner V.; Luk L. Y. P. Transfer hydrogenations catalyzed by streptavidin-hosted secondary amine organocatalysts. Chem. Commun. 2021, 57, 1919–1922. 10.1039/D0CC08142F. PMC8330412 33496282

[ref59] Chatterjee A.; Mallin H.; Klehr J.; Vallapurackal J.; Finke A. D.; Vera L.; Marsh M.; Ward T. R. An enantioselective artificial Suzukiase based on the biotin-streptavidin technology. Chem. Sci. 2016, 7, 673–677. 10.1039/C5SC03116H. 29896353 PMC5953008

[ref60] Vornholt T.; Christoffel F.; Pellizzoni M. M.; Panke S.; Ward T. R.; Jeschek M. Systematic engineering of artificial metalloenzymes for new-to-nature reactions. Sci. Adv. 2021, 7, eabe4208 10.1126/sciadv.abe4208. 33523952 PMC10964965

[ref61] Lo C.; Ringenberg M. R.; Gnandt D.; Wilson Y.; Ward T. R. Artificial metalloenzymes for olefin metathesis based on the biotin-(strept)avidin technology. Chem. Commun. 2011, 47, 12065–12067. 10.1039/c1cc15004a. 21959544

[ref62] Hyster T. K.; Knorr L.; Ward T. R.; Rovis T. Biotinylated Rh(III) Complexes in Engineered Streptavidin for Accelerated Asymmetric C-H Activation. Science 2012, 338, 500–503. 10.1126/science.1226132. 23112327 PMC3820005

[ref63] Hassan I. S.; Ta A. N.; Danneman M. W.; Semakul N.; Burns M.; Basch C. H.; Dippon V. N.; McNaughton B. R.; Rovis T. Asymmetric delta-Lactam Synthesis with a Monomeric Streptavidin Artificial Metalloenzyme. J. Am. Chem. Soc. 2019, 141, 4815–4819. 10.1021/jacs.9b01596. 30865436 PMC6980373

[ref64] Roy A.; Vaughn M. D.; Tomlin J.; Booher G. J.; Kodis G.; Simmons C. R.; Allen J. P.; Ghirlanda G. Enhanced Photocatalytic Hydrogen Production by Hybrid Streptavidin-Diiron Catalysts. Chem.—Eur. J. 2020, 26, 6240–6246. 10.1002/chem.202000204. 32201996

[ref65] Santi N.; Morrill L. C.; Luk L. Y. P. Streptavidin-Hosted Organocatalytic Aldol Addition. Molecules 2020, 25, 2457–2467. 10.3390/molecules25102457. 32466220 PMC7287710

[ref66] Lechner H.; Emann V. R.; Breuning M.; Hocker B. An Artificial Cofactor Catalyzing the Baylis-Hillman Reaction with Designed Streptavidin as Protein Host. ChemBioChem. 2021, 22, 1573–1577. 10.1002/cbic.202000880. 33400831 PMC8247847

[ref67] Olshansky L.; Huerta-Lavorie R.; Nguyen A. I.; Vallapurackal J.; Furst A.; Tilley T. D.; Borovik A. S. Artificial Metalloproteins Containing Co4O4 Cubane Active Sites. J. Am. Chem. Soc. 2018, 140, 2739–2742. 10.1021/jacs.7b13052. 29401385 PMC5866047

[ref68] Mann S. I.; Heinisch T.; Ward T. R.; Borovik A. S. Peroxide Activation Regulated by Hydrogen Bonds within Artificial Cu Proteins. J. Am. Chem. Soc. 2017, 139, 17289–17292. 10.1021/jacs.7b10452. 29117678 PMC5747327

[ref69] Jeschek M.; Bahls M. O.; Schneider V.; Marliere P.; Ward T. R.; Panke S. Biotin-independent strains of Escherichia coli for enhanced streptavidin production. Metab. Eng. 2017, 40, 33–40. 10.1016/j.ymben.2016.12.013. 28062280

