# Supplementary material for: Artificial Metalloenzyme-Catalyzed Enantioselective Amidation via Nitrene Insertion in Unactivated C(sp3)–H Bonds
Source: J Am Chem Soc. 2023 Jul 20;145(30):16621–9. doi: 10.1021/jacs.3c03969 (PMC10401721; doi:10.1021/jacs.3c03969)
Supplement: Supplementary file 1 — ja3c03969_si_001.pdf [file ja3c03969_si_001.pdf]

Supporting Information

Artificial Metalloenzyme-Catalyzed Enantioselective Amidation via Nitrene Insertion in Unactivated C(sp^3)-H Bonds

Kun Yu[a], Zhi Zou[a], Nico V. Igareta[a], Ryo Tachibana[a], Julia Bechter[a], Valentin Köhler[a], Dongping Chen[a] and Thomas R. Ward*[a]

[a] Department of Chemistry, University of Basel, BPR 1096, Mattenstrasse 24a, 4058 Basel, Switzerland

Email: thomas.ward@unibas.ch

Table of contents

1. General aspects 2
- 1.1. Materials and methods 2
- 1.2. Instrumentation 2
- 1.3. Calculation Details 3

2. Synthesis of cofactors and substrates 5
- 2.1. Synthesis of biotinylated ligands 5
- 2.2. Synthesis of the corresponding Ir-cofactors 8
- 2.3. Synthesis of dioxazolones 11

3. HABA titration for determining the binding constant of [Cp*Ir(Boc-AQ-biot)Cl] 10 for Sav WT 13

4. Crystallographic characterization of [Cp*Ir(Boc-AQ-biot)Cl] 10 · Sav WT, Sav S112I and Sav S112I-K121R 15

5. Expression and purification of Sav mutants 17
- 5.1. General procedure for the expression and purification of Sav variants for catalysis 17
- 5.2. Sequence of selected Sav muntants 17

6. Catalysis with purified Sav variants 19
- 6.1. General procedure for catalysis with purified Sav variants 19
- 6.2. Assignment of the absolute configuration of the lactam **2** 22

7. Procedures for preparative scale reactions 23
- 7.1. Preparative experiment using dioxazolone **13** 23
- 7.2. Semi-preparative experiment using dioxazolone **13** 25
- 7.3. Preparative experiment using dioxazolone **1** 26

8. Supporting ables 28

9. Supporting Figures 40

10. NMR Spectra 52

11. Coordinates for calculation 63

1. General aspects

1.1. Materials and methods

All commercially-available chemicals were purchased from Sigma-Aldrich, Acros Organics, TCI Europe, Fluka, Fluorochem, Activate Scientific, Enamine and used without further purification. The most important chemicals are listed below: (*S*)-5-isopropylpyrrolidine-2-one (>97% ee) (CAS: 139564-41-3, Activate Scientific), internal standard 1,3,5-trimethoxybezne (CAS: 621-23-8, Sigma Aldrich), biphenyl (CAS: 92-52-4, Sigma Aldrich). Dioxazolones,[1] racemic products,[1] and $[Cp^{*biot}IrCl_2]_2$[2] were synthesized according to reported procedures. Dry solvents were purchased from Acros Organics and used directly without further purification. The water used for all biological and catalytic experiments was purified with a Milli-Q Advantage system. The Ir cofactors were purified by reversed-phase chromatography on a preparative HPLC. All enzymes used for PCRs and cloning were purchased from NEB, New England Biolabs.

1.2. Instrumentation

^1H and ^{13}C spectra were recorded on a Bruker 500 MHz at room temperature. Chemical shifts are reported in ppm (parts per million) relative to TMS (δ = 0.00 ppm for ^1H and ^{13}C). Signals are quoted as s (singlet), d (doublet), t (triplet), bs (broad singlet) and m (multiplet). Spectra were analyzed on MestReNova and calibrated relative to the residual solvent peak. Electron-Spray Ionization Mass Spectra (ESI-MS) were recorded on a Bruker FTMS 4.7T bioAPEX II. High-resolution mass spectra (HRMS) were measured on a Bruker maXis 4G QTOF ESI mass spectrometer. Supercritical fluid chromatography was carried out on an Acquity UPC2 system from Waters using CO_2 as the mobile phase with addition of a cosolvent. GC analysis of catalysis was performed on a GC-FID equipped with a Astec® CHIRALDEX™ G-TA Capillary GC Column (50 m 0.25 mm 0.12 μm) using He as carrier gas and 1,3,5-trimethoxybezene as internal standard. Circular Dichroism (CD) spectra were recorded on a Chirascan from Applied Photophysics at 25 °C using a quartz cell (1 cm path length). Flash chromatography was performed on a Biotage Isolera or a Buchi Pure chromatography system. Preparative HPLC separations were carried out with a Water Prep LC 4000 System equipped with a Agilent XDB-C18 column (21.2 × 150 mm, 5 μm). No unexpected or unusually high safety hazards were encountered.

1.3. Calculation Details

To the crystal structure of Sav S112I, acidic protons at pH 7.4 were assigned to each amino acid using the PROPKA server (https://server.poissonboltzmann.org/pdb2pqr). Next, both pseudoenantiomers of [Cp*Ir(Boc-AQ-biot)Cl] **10** were docked so that the coordinates of their biotin moiety were superimposable to the biotin observed in the X-ray structure (PDB: 8AQX). Finally, substrate **13** was added. All subsequent calculations were performed at the QM-MM level using gaussian09.[3] As a first step, the structure was optimized by fixing the length between either the pro-*R* or pro-*S* C–H bond and the nitrene atom of the substrate bond to Ir. These lengths were set in analogy to the transition state structure of a related homogeneous nitrene insertase reported by Park and Chang.[4] Using this pseudo transition-state structure as an initial structure, the true transition-state structures were computed without any bond-length constraints. During the structural optimization, only the substrate, catalyst (except for the bicyclic urea moiety of biotin), and other nearby atoms of Sav (within 8 Å) were allowed to relax, while the coordinates of other parts were frozen. Calculations were performed at the ONIOM(b3lyp:UFF) level. The basis set was LANL2DZ for Ir and 6-31G(d,p) for all other atoms. The high layer included only substrate and catalyst (except for the fused urea moiety of biotin). The atomic charges for the cofactor and the substrate were computed at the same level as the high layer. For the final result, the high layer energy was referred.

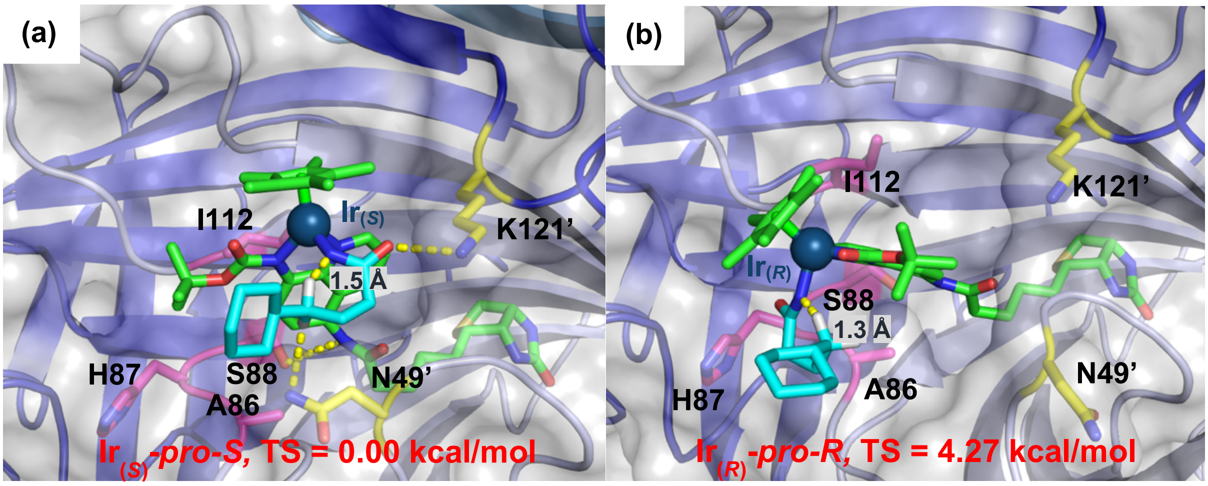

(a) Hydrophobic interactions with TS:
A86, H87, L110, I112, L124
H-bonding interactions:
N49’ with C–H of substrate
S88 with N–H of cofactor
K121’ with C=O of nitrene

(b) Hydrophobic interactions with TS:
A86, H87, I112
H-bonding interactions:
S88 with N–H of cofactor

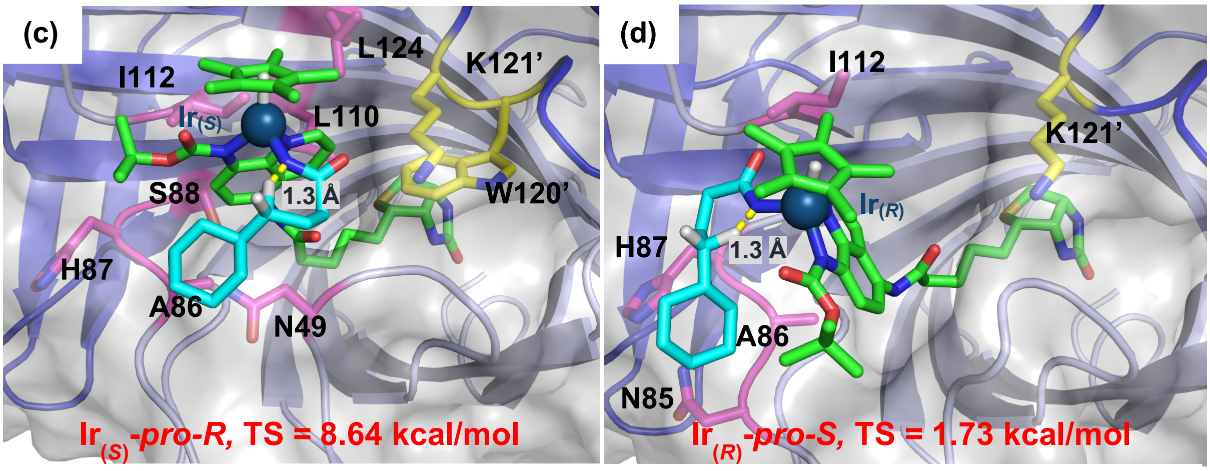

Hydrophobic interactions with TS:
W120’, N49, A86, H87, L110, I112, L124
H-bonding interactions:
S88 with N–H of cofactor
K121’ with C=O of nitrene
[illegible]

Hydrophobic interactions with TS:
N85, A86, H87, I112

Figure S1. QM-MM Computation of the reaction paths leading to enantiopure **14**. Close-up view of the transition state with (a) $Ir_{(S)}$ interacting with the *pro-S* C–H bond leading to (*S*)-**14**; (b) $Ir_{(R)}$ interacting with the *pro-R* C–H bond leading to (*R*)-**14**; (c) $Ir_{(S)}$ interacting with the *pro-R* C–H bond leading to (*R*)-**14**; (d) $Ir_{(R)}$ interacting with the *pro-S* C–H bond leading to (*S*)-**14**. The cofactor is displayed as color-coded sticks (nitrogen = blue, oxygen = red and carbon = green) with Ir displayed as dark blue sphere. The nitrene moiety is represented a color-coded sticks (carbon = cyan). Close-lying residues that interact with the transition states are represented as color-coded sticks (for residues interact with substrate and cofactor, carbon = yellow and magenta respectively).

2. Synthesis of cofactors and substrates

2.1. Synthesis of the biotinylated ligands

[Cp*Ir($H_2NCH_2CH_2NTos$-biot)Cl] **3** was synthesized according to a reported procedure.[5]

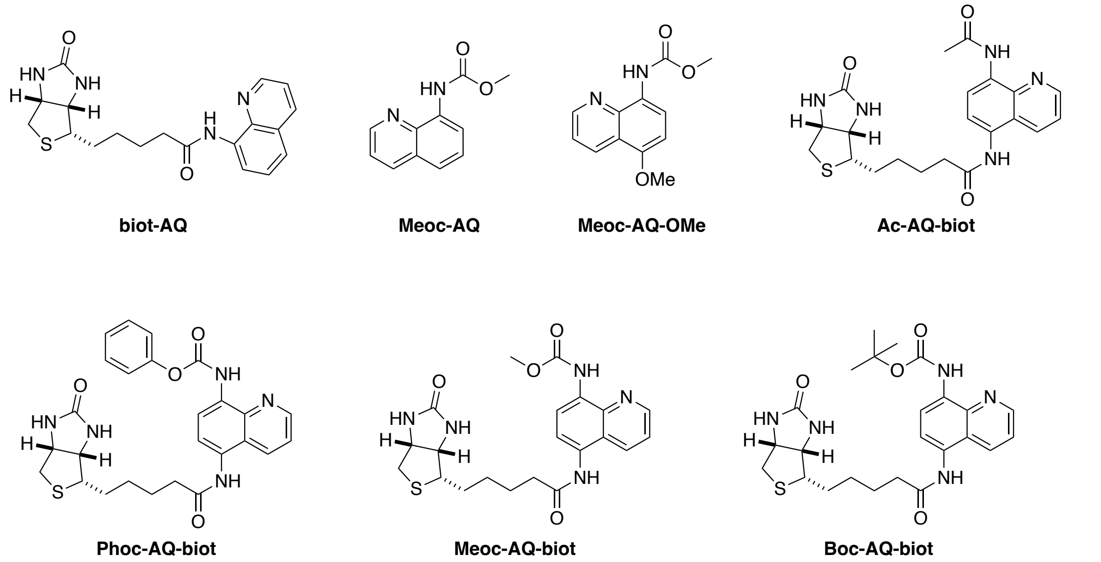

Figure S2: List of ligands used in this work.

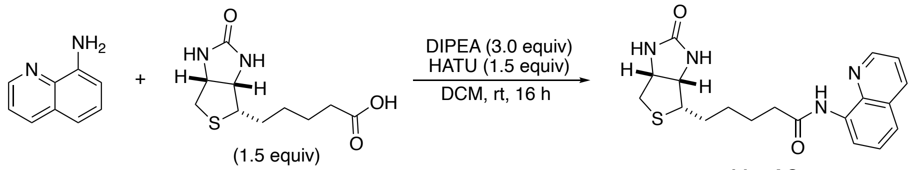

biot-AQ

Scheme S1: Synthesis of biotinylated ligand **biot-AQ**.[6]

Biotin (254 mg, 1.04 mmol), Hexafluorophosphate Azabenzotriazole Tetramethyl Uronium (HATU, 396 mg, 1.04 mmol) and *N,N*-Diisopropylethylamine (DIPEA, 344 µL, 2.08 mmol) were added to a solution of 8-aminoquinoline (100 mg, 0.70 mmol) in dry dichloromethane (DCM) (5 mL). The solution was stirred at room temperature for 16 h. Water was added, and the mixture was extracted with DCM (3 × 10 mL). The combined organic layers were washed with brine, dried over anhydrous $MgSO_4$ and concentrated in vacuo. The resulting residue was purified by silica gel flash chromatography (DCM/MeOH = 95:5) to afford the **biot-AQ** (213 mg, 83%) as a pale-yellow solid. R_f (DCM/MeOH = 90:10): 0.48; ^{1}H NMR (500 MHz, DMSO-*d*$_6$): 10.05 (s, 1H), 8.94 (dd, J = 4.2, 1.7 Hz, 1H), 8.63 (dd, J = 7.7, 1.3 Hz, 1H), 8.41 (dd, J = 8.3, 1.7 Hz, 1H), 7.71 – 7.62 (m, 2H), 7.58 (t, J = 7.9 Hz, 1H), 6.45 (s, 1H), 6.36 (s, 1H), 4.33 – 4.28 (m, 1H), 4.18 – 4.13 (m, 1H), 3.17 – 3.11 (m, 1H), 2.83 (dd, J = 12.4, 5.1 Hz, 1H), 2.62 – 2.56 (m, 3H), 1.76 – 1.61 (m, 3H), 1.59 – 1.49 (m, 1H), 1.48 – 1.35 (m, 2H); ^{13}C NMR (126 MHz, DMSO-*d*$_6$): 174.1, 171.3, 162.4, 162.4, 148.4, 137.6, 136.6, 134.1, 127.6, 126.7, 121.8, 121.5, 116.6, 60.7, 60.7, 58.9, 58.9, 55.1, 55.1, 36.2, 33.2, 27.9, 27.8, 27.8, 27.7, 24.9, 24.2;

HRMS (ESI positive mode, m/z): calculated for C19H22N4O2S $[M+H]^+$ 371.1536; found 371.1538.

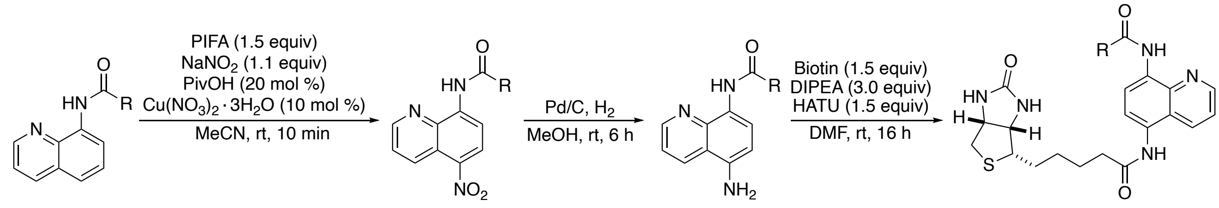

Scheme S2: Synthesis of biotinylated ligands.

Step 1. C-H nitration of 8-aminoquinoline derivatives:[7]

[Bis(trifluoroacetoxy)iodo]benzene (PIFA, 1.5 equiv), $NaNO_2$ (1.1 equiv), PivOH (20 mol %) and $Cu(NO_3)_2 \cdot 3H_2O$ (10 mol %) were added to a solution of amide **S1** (1.0 equiv) in MeCN (0.1 M). The mixture was stirred at room temperature for 10 min. After complete consumption of **S1** (monitored by TLC), the solvent was removed *in vacuo*. The residue was purified by silica gel flash chromatography (cyclohexane/ethylacetate (EA) = 4:1) to afford the corresponding nitration product **S2**.

Step 2. Hydrogenation of the nitro group:[7]

Pd/C (20% wt) was added to a solution of compound **S2** in MeOH (0.1 M). The mixture was stirred under hydrogen at room temperature for 6 h. The solution was filtered through a celite pad, and the solvent was removed *in vacuo*. The residue was purified by silica gel flash chromatography (DCM/MeOH = 20:1) to afford the crude amine product **S3**. A more thorough purification was carried out after the following step **S3**.

Step 3. Biotinylation of the amine:[6]

Biotin (1.5 equiv), HATU (1.5 equiv) and DIPEA (3.0 equiv) were added to a solution of the amine (1.0 equiv) in dry DMF (0.2 M). The solution was stirred at room temperature for 16 h. The solvent was removed *in vacuo*. The residue was resuspended in DCM. The solvent was filtered off and the resulting residue was washed with a large volume of DCM to afford the biotinylated ligand.

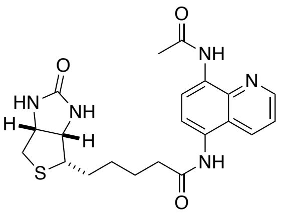

Ac-AQ-biot

White solid. **Ac-AQ-biot** was synthesized following the general procedures in 29% yield (3 steps).

^{1}H NMR (500 MHz, DMSO-d_6): 10.09 (s, 1H), 9.90 (s, 1H), 8.95 (dd, J = 4.2, 1.6 Hz, 1H), 8.56 (d, J = 8.4 Hz, 1H), 8.42 (dd, J = 8.5, 1.6 Hz, 1H), 7.67 (dd, J = 8.5, 4.1 Hz, 1H), 7.62 (d, J = 8.3 Hz, 1H), 6.46 (s, 1H), 6.37 (s, 1H), 4.39 – 4.26 (m, 1H), 4.20 – 4.14 (m, 1H), 3.23 – 3.03 (m, 1H), 2.85 (dd, J = 12.4, 5.1 Hz, 1H), 2.60 (d, J = 12.4 Hz, 1H), 2.46 (t, J = 7.5 Hz, 2H), 2.27 (s, 3H), 1.77 – 1.63 (m, 3H), 1.61 – 1.37 (m, 3H).

^{13}C NMR (126 MHz, DMSO-d_6): 173.8, 172.7, 169.4, 163.2, 149.8, 140.8, 138.9, 133.4, 131.1, 122.9, 122.2, 119.6, 116.8, 61.5, 59.7, 55.8, 51.7, 33.6, 28.5, 24.9, 24.8.

HRMS (ESI positive mode, m/z): calculated for $C_{21}H_{25}N_5O_3S$ $[M+H]^+$ 428.1751; found 428.1752.

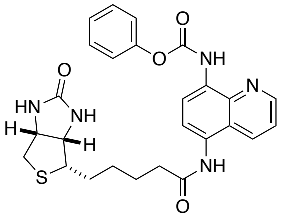

Phoc-AQ-biot

Pale-brown solid. **Phoc-AQ-biot** was synthesized following the general procedures in 10% yield (3 steps).

^{1}H NMR (500 MHz, DMSO-d_6): 9.94 (s, 1H), 9.70 (s, 1H), 8.97 (dd, J = 4.2, 1.6 Hz, 1H), 8.46 (dd, J = 8.6, 1.6 Hz, 1H), 8.20 (d, J = 8.3 Hz, 1H), 7.74 – 7.67 (m, 2H), 7.49 – 7.42 (m, 2H), 7.36 – 7.25 (m, 3H), 6.45 (s, 1H), 6.37 (s, 1H), 4.35 – 4.27 (m, 1H), 4.22 – 4.10 (m, 1H), 3.19 – 3.12 (m, 1H), 2.84 (dd, J = 12.4, 5.1 Hz, 1H), 2.60 (d, J = 12.4 Hz, 1H), 2.46 (t, J = 7.4 Hz, 2H), 1.75 – 1.64 (m, 3H), 1.59 – 1.38 (m, 4H).

^{13}C NMR (126 MHz, DMSO-d_6): 174.9, 172.5, 163.2, 151.8, 151.0, 149.6, 138.8, 133.0, 131.8, 129.9, 129.1, 126.1, 123.8, 123.0, 122.4, 122.3, 61.5, 59.7, 59.6, 55.9, 55.9, 55.4, 36.1, 28.8, 28.6, 25.7.

HRMS (ESI positive mode, m/z): calculated for $C_{26}H_{28}N_5O_4S$ $[M+H]^+$ 506.1857; found 506.1854.

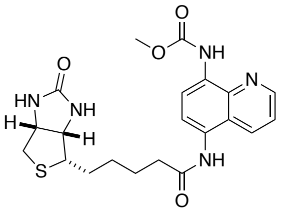

Meoc-AQ-biot

Pale-yellow solid. **Meoc-AQ-biot** was synthesized following the general procedures in 13% yield (3 steps).

^{1}H NMR (500 MHz, DMSO-d_6): 9.91 (s, 1H), 9.23 (s, 1H), 8.92 (dd, J = 4.2, 1.6 Hz, 1H), 8.42 (dd, J = 8.5, 1.7 Hz, 1H), 8.23 (d, J = 8.3 Hz, 1H), 7.73 – 7.59 (m, 2H), 6.46 (s, 1H), 6.38 (s,

1H), 4.33 (dd, J = 7.8, 5.1 Hz, 1H), 4.18 (s, 1H), 3.77 (s, 3H), 3.22 – 3.08 (m, 1H), 2.85 (dd, J = 12.4, 5.1 Hz, 1H), 2.60 (d, J = 12.4 Hz, 1H), 2.46 (t, J = 7.4 Hz, 2H), 1.83 – 1.62 (m, 3H), 1.60 – 1.31 (m, 3H).

^{13}C NMR (126 MHz, DMSO-*d*$_6$): 174.9, 172.5, 163.2, 153.9, 149.3, 138.3, 132.9, 132.3, 128.3, 123.9, 123.3, 122.2, 114.9, 61.5, 59.7, 55.9, 52.7, 36.1, 28.8, 28.6, 25.7.

HRMS (ESI positive mode, m/z): calculated for $C_{21}H_{25}N_5O_4S$ $[M+H]^+$ 444.1700; found 444.1704.

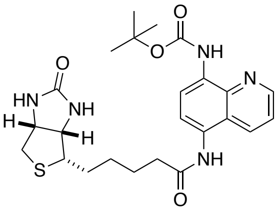

Boc-AQ-biot

Yellow solid. **Boc-AQ-biot** was synthesized following the general procedures in 14% yield (3 steps).

^{1}H NMR (500 MHz, DMSO-*d*$_6$): 9.89 (s, 1H), 8.96 (s, 1H), 8.90 (dd, J = 4.3, 1.6 Hz, 1H), 8.41 (dd, J = 8.6, 1.6 Hz, 1H), 8.22 (d, J = 8.3 Hz, 1H), 7.67 (dd, J = 8.6, 4.2 Hz, 1H), 7.62 (d, J = 8.4 Hz, 1H), 6.46 (s, 1H), 6.38 (s, 1H), 4.39 – 4.28 (m, 1H), 4.21 – 4.11 (m, 1H), 3.21 – 3.10 (m, 1H), 2.85 (dd, J = 12.4, 5.1 Hz, 1H), 2.60 (d, J = 12.4 Hz, 1H), 2.46 (t, J = 7.4 Hz, 2H), 1.76 – 1.61 (m, 3H), 1.53 (s, 9H), 1.50 – 1.35 (m, 2H).

^{13}C NMR (126 MHz, DMSO-*d*$_6$): 172.5, 163.2, 152.5, 149.2, 138.0, 133.0, 132.5, 127.9, 123.8, 123.3, 122.2, 114.2, 80.6, 61.5, 59.7, 55.9, 55.4, 36.0, 28.8, 28.6, 28.4, 25.7.

HRMS (ESI positive mode, m/z): calculated for $C_{24}H_{31}N_5O_4S$ $[M+H]^+$ 486.2170; found 486.2177.

2.2. Synthesis of the corresponding Ir-cofactors

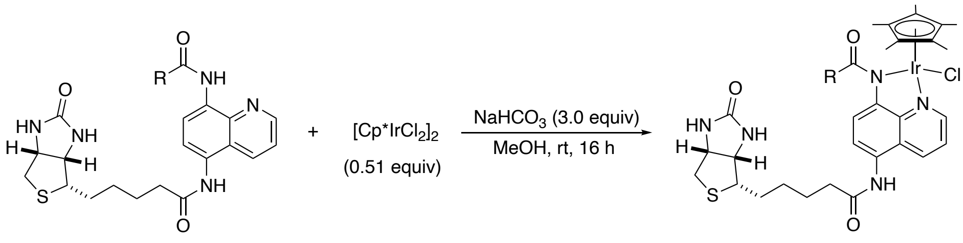

Scheme S3: Synthesis of Ir cofactors.

Preparation of [Cp*Ir(PG-AQ-biot)Cl]:[1]

$NaHCO_3$ (3.0 equiv) was added to a mixture of $[Cp^*IrCl_2]_2$ (0.51 equiv) and the biotinylated ligand **PG-AQ-biot** (1.0 equiv) in dry MeOH. The solution was stirred at room temperature for

16 h. The solvent was removed *in vacuo*. The resulting residue was purified by reverse-phase preparative HPLC (Solvents were composed as follows: (A) water/acetonitrile/TFA = 97:3:0.1; (B) acetonitrile/TFA = 99.9:0.1. Method: 0 min – 0% B; 4 min – 10% B; 30 min – 90% B; 31 min – 100% B; 36 min – 100%.) to afford the corresponding Ir cofactors.

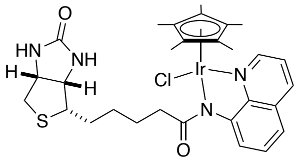

[Cp*Ir(biot-AQ)Cl] **4**

Orange solid. [Cp*Ir(biot-AQ)Cl] **4** was synthesized following the general procedure in 75% yield.

^{1}H NMR (500 MHz, DMSO-*d$_6$*): 8.90 (dd, J = 5.2, 1.3 Hz, 1H), 8.66 (dd, J = 8.4, 1.3 Hz, 1H), 7.82 (dd, J = 8.3, 5.1 Hz, 2H), 7.67 (t, J = 8.0 Hz, 1H), 7.51 (d, J = 8.0 Hz, 1H), 6.43 (s, 1H), 6.38 (s, 1H), 4.36 – 4.27 (m, 1H), 4.19 – 4.11 (m, 1H), 3.19 – 3.09 (m, 1H), 2.98 (brs, 1H), 2.83 (dd, J = 12.4, 5.1 Hz, 1H), 2.68 – 2.54 (m, 2H), 1.80 – 1.57 (m, 4H), 1.52 (s, 15H), 1.45 – 1.32 (m, 2H).

^{13}C NMR (126 MHz, DMSO-*d$_6$*): 177.1, 163.2, 158.9, 158.7, 158.4, 158.1, 153.9, 148.7, 146.8, 140.9, 130.3, 129.5, 124.6, 122.6, 120.7, 95.2, 61.5, 59.7, 55.9, 28.9, 28.7, 26.8, 8.7.

HRMS (ESI positive mode, m/z): calculated for $C_{29}H_{36}ClIrN_4O_2S$ $[M-Cl]^+$ 697.2182; found 697.2194.

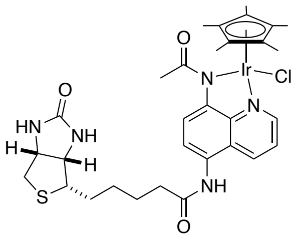

[Cp*Ir(Ac-AQ-biot)Cl] **7**

Orange solid. [Cp*Ir(Ac-AQ-biot)Cl] **7** was synthesized following the general procedure in 82% yield.

^{1}H NMR (500 MHz, DMSO-*d$_6$*): 10.05 (s, 1H), 8.92 (dd, J = 5.2, 1.3 Hz, 1H), 8.61 (dd, J = 8.6, 1.2 Hz, 1H), 7.89 (d, J = 8.5 Hz, 1H), 7.84 (dd, J = 8.6, 5.2 Hz, 1H), 7.75 (d, J = 8.6 Hz, 1H), 6.45 (s, 1H), 6.39 (s, 1H), 4.33 (dd, J = 7.8, 4.9 Hz, 1H), 4.17 (dd, J = 7.8, 4.4 Hz, 1H), 3.21 – 3.11 (m, 1H), 2.85 (dd, J = 12.5, 5.1 Hz, 1H), 2.60 (d, J = 12.4 Hz, 2H), 2.49 – 2.43 (m, 5H), 1.78 – 1.61 (m, 3H), 1.53 (s, 15H), 1.50 – 1.36 (m, 2H).

^{13}C NMR (126 MHz, DMSO-*d$_6$*): 174.4, 172.7, 163.2, 159.0, 158.7, 158.4, 158.1, 154.0, 146.5, 146.5, 136.9, 127.6, 125.1, 124.4, 123.9, 121.7, 95.3, 61.6, 59.7, 55.9, 36.0, 28.8, 28.6, 25.9, 25.7, 8.6.

HRMS (ESI positive mode, m/z): calculated for $C_{31}H_{39}ClIrN_5O_3S$ [M-Cl]$^+$ 754.2397; found 754.2402.

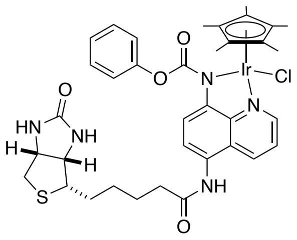

[Cp*Ir(Phoc-AQ-biot)Cl] **8**

Orange solid. [Cp*Ir(Phoc-AQ-biot)Cl] **8** was synthesized following the general procedure in 78% yield.

^{1}H NMR (500 MHz, DMSO-d_6): 10.06 (s, 1H), 9.00 (dd, J = 5.2, 1.3 Hz, 1H), 8.65 (dd, J = 8.5, 1.3 Hz, 1H), 8.62 (d, J = 8.7 Hz, 1H), 7.86 (dd, J = 8.6, 5.2 Hz, 1H), 7.78 (d, J = 8.7 Hz, 1H), 7.55 – 7.40 (m, 2H), 7.35 – 7.21 (m, 3H), 6.45 (s, 1H), 6.39 (s, 1H), 4.39 – 4.26 (m, 1H), 4.21 – 4.12 (m, 1H), 3.26 – 3.11 (m, 1H), 2.85 (dd, J = 12.4, 5.1 Hz, 1H), 2.60 (d, J = 12.4 Hz, 1H), 2.48 (d, J = 7.5 Hz, 2H), 1.75 – 1.64 (m, 3H), 1.56 (s, 15H), 1.50 – 1.36 (m, 2H).

^{13}C NMR (126 MHz, DMSO-d_6): 172.7, 163.2, 159.0, 158.7, 158.4, 158.1, 156.0, 154.0, 152.4, 146.0, 145.5, 137.1, 129.9, 127.7, 125.6, 125.3, 125.1, 124.0, 122.3, 121.8, 95.9, 61.6, 59.7, 55.9, 36.0, 28.8, 28.6, 25.7, 8.6.

HRMS (ESI positive mode, m/z): calculated for $C_{36}H_{41}ClIrN_5O_4S$ [M-Cl]$^+$ 832.2503; found 832.2507.

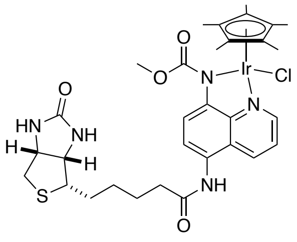

[Cp*Ir(Meoc-AQ-biot)Cl] **9**

Orange solid. [Cp*Ir(Meoc-AQ-biot)Cl] **9** was synthesized following the general procedure in 80% yield.

^{1}H NMR (500 MHz, DMSO-d_6): 10.00 (s, 1H), 8.95 (dd, J = 5.2, 1.2 Hz, 1H), 8.64 (d, J = 8.7 Hz, 1H), 8.58 (dd, J = 8.6, 1.3 Hz, 1H), 7.82 (dd, J = 8.6, 5.2 Hz, 1H), 7.73 (d, J = 8.7 Hz, 1H), 6.45 (s, 1H), 6.39 (s, 1H), 4.39 – 4.28 (m, 1H), 4.22 – 4.12 (m, 1H), 3.71 (s, 3H), 3.21 – 3.10 (m, 1H), 2.85 (dd, J = 12.4, 5.1 Hz, 1H), 2.61 (d, J = 12.3 Hz, 1H), 2.46 (t, J = 7.4 Hz, 2H), 1.73 – 1.63 (m, 3H), 1.54 (s, 15H), 1.49 – 1.36 (m, 2H).

^{13}C NMR (126 MHz, DMSO-d_6): 173.8, 172.6, 163.2, 158.9, 158.6, 158.4, 158.1, 157.9, 153.7, 146.7, 145.3, 137.1, 126.7, 125.4, 123.7, 120.7, 95.6, 61.6, 59.7, 55.9, 53.4, 51.7, 36.0, 33.6, 28.8, 28.6, 25.7, 24.9, 8.4.

HRMS (ESI positive mode, m/z): calculated for $C_{31}H_{39}ClIrN_5O_4S$ $[M\text{-}Cl]^+$ 770.2346; found 770.2349.

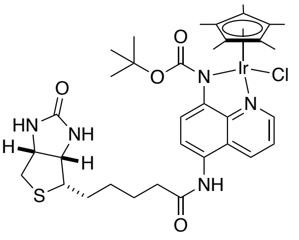

[Cp*Ir(Boc-AQ-biot)Cl] **10**

Orange solid. [Cp*Ir(Boc-AQ-biot)Cl] **10** was synthesized following the general procedure in 85% yield.

^{1}H NMR (500 MHz, DMSO-*d₆*): 9.98 (s, 1H), 8.93 (dd, J = 5.2, 1.2 Hz, 1H), 8.56 (d, J = 8.5 Hz, 1zH), 8.24 (d, J = 8.7 Hz, 1H), 7.80 (dd, J = 8.6, 5.1 Hz, 1H), 7.70 (d, J = 8.7 Hz, 1H), 6.45 (s, 1H), 6.39 (s, 1H), 4.39 – 4.26 (m, 1H), 4.25 – 4.11 (m, 1H), 2.85 (dd, J = 12.4, 5.1 Hz, 1H), 2.60 (d, J = 12.4 Hz, 1H), 2.46 (t, J = 7.4 Hz, 2H), 1.84 – 1.62 (m, 5H), 1.56 (s, 23H), 1.48 – 1.35 (m, 2H).

^{13}C NMR (126 MHz, DMSO-*d₆*): 172.7, 163.2, 159.0, 158.7, 158.4, 158.1, 156.9, 153.9, 146.3, 145.8, 136.9, 126.4, 125.3, 125.3, 123.6, 121.2, 95.4, 80.9, 69.0, 61.6, 59.7, 56.3, 55.9, 36.0, 32.6, 30.1, 28.8, 28.7, 28.6, 25.8, 8.6.

HRMS (ESI positive mode, m/z): calculated for $C_{34}H_{45}ClIrN_5O_4S$ $[M\text{-}Cl]^+$ 812.2816; found 812.2822.

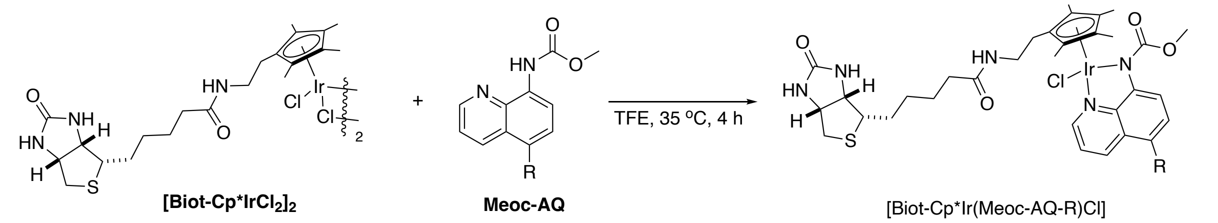

Scheme S4: *In-situ* preparation of Ir cofactors.

In-situ preparation of [Biot-Cp*Ir(Meoc-AQ)Cl] 5 and 6:

[Biot-Cp*Ir(Meoc-AQ)Cl] **5** and **6** were *in situ* prepared based on reported method:[8, 9] [Biot-Cp*IrCl₂]₂ (3.75×10^{-6} mmol) and the ligand **Meoc-AQ** (7.87×10^{-6} mmol) were dissolved in TFE (100 μL) and stirred for 2 h at 35 °C. The resulting solution was used for the enantioselective catalytic C-H amidation directly.

2.3. Synthesis of dioxazolones

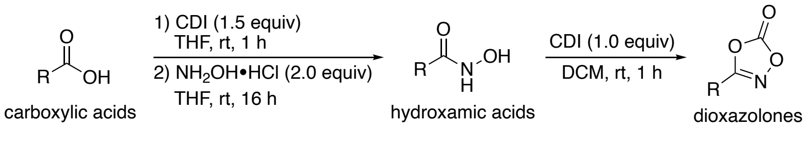

General procedure for the synthesis of dioxazolones according to reported procedures:[1]

Step 1. Synthesis of hydroxamic acids:

1,1'-Carbonyldiimidazole (CDI, 1.5 equiv) was added to a solution of the carboxylic acid (1.0 equiv) in dry THF (0.3 M). The mixture was stirred at room temperature for 1 h. $NH_2OH{\cdot}HCl$ (2.0 equiv) was added. The reaction mixture was stirred overnight at room temperature. $KHSO_4$ (5% aq.) was added and the mixture was extracted with EA. The organic layers were combined and washed with brine then dried over $MgSO_4$. The extract was filtered and concentrated *in vacuo*. The residue was purified by silica gel flash chromatography (DCM/MeOH = 20:1) to afford the hydroxamic acid.

Step 2. Synthesis of dioxazolones:

To the solution of hydroxamic acid (1.0 equiv) in dry DCM (0.1 M) was added CDI (1.0 equiv). The mixture was stirred at room temperature for 1 h. The reaction was quenched with HCl (1 N), extracted with DCM. The extract was dried over $MgSO_4$ and filtered. The solvent was removed *in vacuo*. The residue was filtered through a pad of silica and washed with DCM. The filtrate was concentrated *in vacuo* to afford the dioxazolones.

Dioxazolones **1,**[10, 11] **11,**[10, 11] **15,**[11] **17,**[10-12] **19,**[1, 13] **21,**[1] **23**[1], **25,**[1] **27**[1] are reported compounds and synthesized following the above procedure.

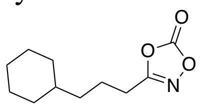

13

^{1}H NMR (500 MHz, $CDCl_3$): 2.60 (t, J = 7.5 Hz, 2H), 1.80 – 1.60 (m, 7H), 1.32 – 1.08 (m, 6H), 0.95 – 0.82 (m, 2H).

^{13}C NMR (126 MHz, $CDCl_3$): 166.7, 154.2, 37.1, 36.4, 33.1, 26.5, 26.2, 25.0, 22.0.

3. HABA titration for determining the binding constant of [Cp*Ir(Boc-AQ-biot)Cl] 10 for Sav WT

HABA titration was performed according to previously reported procedure.[6, 14, 15] To a quartz cuvette, a solution of streptavidin WT (Sav WT, tetrameric, initial concentration 8 μM, 2.4 mL, 0.0192 μmol, 1.0 equiv) in PBS buffer (20 mM, pH 7) was added. A solution of 2-(4'-hydroxyazobenzene)benzoic acid (HABA, 9.6 mM, 300 μL, 2.88 μmol, 150 equiv) in PBS buffer (20 mM, pH 7) was added and the mixture was incubated for 5 min to ensure full saturation of the biotin-binding sites. A blank (PBS buffer only) was measured at 506 nm and the absorbance of the HABA·Sav solution was determined. Aliquots of [Cp*Ir(Boc-AQ-biot)Cl] **10** (0.96 mM in DMSO) or biotin (0.96 mM in DMSO) were added to the HABA·Sav solution in 0.50 equiv. step (10 μL per step, up to 5.0 equiv). The CD spectrum (at 506 nm) was recorded 2 minutes after each addition and the molar ellipticity was plotted against the equivalents of [Cp*Ir(Boc-AQ-biot)Cl] **10** or biotin added. The decrease of CD signal ceased when the HABA was completely displaced by [Cp*Ir(Boc-AQ-biot)Cl] **10** or biotin. The measured data was fitted according to published method.[16, 17]

$$A = (Ka + Kb + Ca + X - Cp)$$

$$B = (Kb * (Ca - Cp) - Ka * (X - Cp) - Ka * Kb)$$

$$\theta = (\arccos\left(\frac{-2*(Ka+Kb+Ca+X-Cp)^3+9*(Ka+Kb+Ca+X-Cp)*(Kb*(Ca-Cp)+Ka*(X-Cp)+Ka*Kb)-27*(-Ka*Kb*Cp)}{2*sqrt\left(((Ka+Kb+Ca+X-Cp)^2-3*(Kb*(Ca-Cp)+Ka*(X-Cp)+Ka*Kb))^3\right)}\right))$$

$$Cbound = Ca * (2 * sqrt(A\hat{}2 - 3 * B) * \cos(\theta/3) - A)/(3 * Ka + \left(2 * sqrt(A^2 - 3 * B)\right) * \cos\left(\frac{\theta}{3}\right) - A))$$

$$Cfree = Ca - Cbound$$

$$Y = Cbound * Ebound + Baseline$$

Abbreviations:

$K_a = K_{HABA}$

$K_b = K_{cofactor}$

$C_a = [HABA]_{total}$

$X = [Cofactor]_{total}$

$C_p = [Sav_{mono}]_{total}$

C_{bound} = [HABA•Sav]

E_{bound} = molar ellipticity

Y = Calculated signal

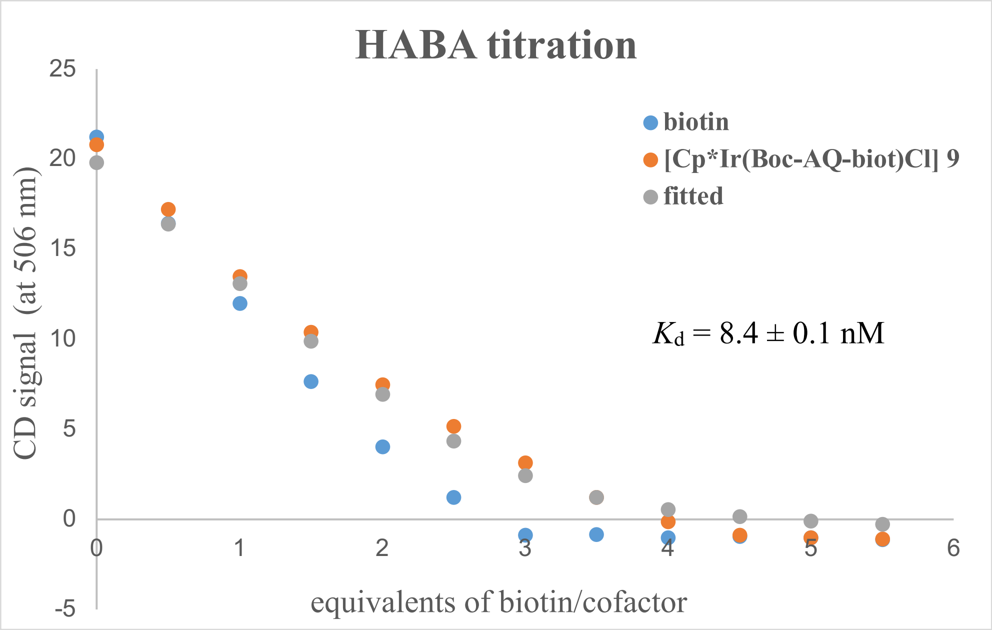

Figure S3. HABA titration experiments with biotin (blue), [Cp*Ir(Boc-AQ-biot)Cl] **10** (orange); fitted value based on the same K_d for all four binding sites (gray).

4. Crystallographic characterization of [Cp*Ir(Boc-AQ-biot)Cl] 10 · Sav WT, Sav S112I and Sav S112I-K121R

Lyophilized wild-type (WT) Sav was dissolved in ultrapure water (18.2 MΩ·cm, MilliQ, Millipore Corporation, Burlington, USA). For sitting drop vapor diffusion, Sav WT (2.5 µL of a 20 mg/mL stock solution) was mixed with the precipitation buffer (2.5 µL, 2.0 M $(NH_4)_2SO_4$, 100 mM Na_2SO_4, pH 4.0). The drop was equilibrated against a reservoir of the precipitation buffer (60 µL at 20 °C). Crystals of Sav WT grew within 4 days. For soaking, individual crystals were transferred into a sitting-drop containing the pH-adapted soaking buffer (4.5 µL, 2.3 M $(NH_4)_2SO_4$, 100 mM Na_2SO_4, pH 6.0) and a solution of [Cp*Ir(Boc-AQ-biot)Cl] **10** (10 mM, 0.5 µL in DMSO) was added to the drop. After soaking (20 h, 20 °C), the crystals were flash-frozen in liquid nitrogen.

Lyophilized Sav S112I was dissolved in 20 mM Tris-HCl buffer (20 mM, pH 7.2, protein concentration 2.0 mg/mL) and an 2 fold excess of [Cp*Ir(Boc-AQ-biot)Cl] **10** (in DMSO) was added to the solution. After 6 h of incubation, the buffer was exchanged to remove the unbound cofactor by ultracentrifugation (Amicon® ultra centrifugal filters, Merck; cut-off of 10 kDa; 14,000 g, 5 min RT; dilution buffer: water) and the protein was concentrated 10 times (to 20 mg/mL). For sitting drop vapor diffusion, the protein solution (2.5 µL of a 20 mg/mL stock solution) was mixed with the precipitation buffer (2.5 µL, 2.0 M NaCl, 100 mM Bis-Tris, pH 6.5, 25% w/v PEG 3350). The drop was equilibrated against a reservoir of the precipitation buffer (60 µL at 20 °C). Crystals of [Cp*Ir(Boc-AQ-biot)Cl] **10** ·SavS112I grew within 3-4 weeks. The crystals were directly flash-frozen in liquid nitrogen prior to data collection.

Lyophilized Sav S112I-K121R was dissolved in ultrapure water (18.2 MΩ·cm, MilliQ, Millipore Corporation, Burlington, USA). For sitting drop vapor diffusion, Sav S112I-K121R (2.5 µL of a 20 mg/mL stock solution) was mixed with the precipitation buffer (2.5 µL, 2.0 M $(NH_4)_2SO_4$, 100 mM Na_2SO_4, pH 4.0). The drop was equilibrated against a reservoir of the precipitation buffer (60 µL at 20 °C). Crystals of Sav S112I-K121R grew within 14 days. For soaking, individual crystals were transferred into a sitting-drop containing the pH-adapted soaking buffer (4.5 µL, 2.3 M $(NH_4)_2SO_4$, 100 mM Na_2SO_4, pH 6.0) and a solution of [Cp*Ir(Boc-AQ-biot)Cl] **10** (10 mM, 0.5 µL in DMSO) was added to the drop. After soaking (20 h, 20 °C), the crystals were flash-frozen in liquid nitrogen.

The data collection was carried out at the Swiss Light Source beam line PSI at a wavelength of 1.0 Å. XDS[18] and AIMLESS[19] were used for crystal indexing, integration and scaling, using the graphical interface CCP4i2[20] of the CCP4 suite. The structures were solved by molecular

replacement using PHASER MR[21] and the streptavidin structure PDB:2BC3. Refinement was carried out by REFMAC[22] and for structure modeling and electron-density visualization COOT[23] was used. Figures were generated with PyMOL (the PyMOL Molecular Graphics System, Version 1.821, Schrödinger, LLC). In the case of Sav WT and Sav S112I-K121R one monomer per asymmetric unit was found (space group $I4_122$), whereas for Sav S112I four monomers (corresponding to tetrameric structure) were obtained (space group C121).

Residual electron density in the F_o-F_c map was observed in the biotin-binding site of streptavidin for all structures. Furthermore, anomalous dispersion density was observed. Modeling of the cofactor [Cp*Ir(Boc-AQ-biot)Cl] **10** into the electron density projected the iridium in the position of the anomalous density peak.

Table S1. Data processing and crystal structure refinement statistics.

Sav	Sav WT	Sav S112I	Sav S112I-K121R
Cofactor	[Cp*Ir(Boc-AQ-biot)Cl] **10**	[Cp*Ir(Boc-AQ-biot)Cl] **10**	[Cp*Ir(Boc-AQ-biot)Cl] **10**
PDB Code	8BY1	8AQX	8BY0

Data Processing Statistics

Resolution Range (Å)	45.91-1.49 (1.51-1.49)	46.19-1.85 (1.89-1.85)	45.78-2.10 (2.16-2.10)
Cell Parameters<br>- a, b, c (Å)<br>- α, β, γ (°)	57.65, 57.65, 183.62<br>90.00, 90.00, 90.00	114.88, 88.83, 57.83<br>90.00, 97.29, 90.00	57.42, 57.42, 183.12<br>90.00, 90.00, 90.00
Space group	I4122	C121	I4122
Unique reflections	26068 (1214)	47474 (2675)	9422 (755)
Rmerge (%)	7.8 (228)	6.7 (56.3)	18.9 (234)
Multiplicity	25.3 (19.4)	7.1 (6.9)	15.5 (16.5)
Mean I/Sig(I)	23.7 (1.5)	15.9 (3.6)	10.5 (1.5)
Completeness (%)	99.9 (97.5)	96.8 (89.5)	99.8 (100)
CC (1/2)	1.00 (0.556)	0.999 (0.924)	0.998 (0.573)

Structure Refinement Statistics

R_{work}/R_{free}	0.19/0.20	0.18/0.21	0.21/0.25
Average B-factors (Å)	27.0	38.0	47.0

5. Expression and purification of Sav mutants

5.1. General procedure for the expression and purification of Sav variants for catalysis

The plasmids of the Sav mutants were produced according to previous work.[17] Plasmids were transformed into *E. coli* BL21 (DE3) chemically competent cells. After heat-shock at 42 °C, the cells were placed on ice for 2 min. Super Optimal Broth (SOC) medium (500 µL) was added into vials and the cells were incubated for 1 h with a shaker (300 rpm at 37 °C). The suspension (150 µL) was plated out on Lysogeny Broth (LB) agar plates supplemented with antibiotic (50 µg/mL kanamycin). The plates were incubated (37 °C, 16 h). Preculture was prepared by inoculating fresh single colony in 2.5 mL LB medium in 24-well plates and incubated (37 °C, 300 rpm, 16 h). The preculture (500 µL) was inoculated in the main culture (50 mL, ZYP-5052 medium containing 200 µg/mL kanamycin) and further incubated (25 °C, 200 rpm, 24 h). The cells were harvested by centrifugation (4 °C, 3500 g, 10 min) and frozen overnight at -20 °C. Cells were lysed for 2 h. The suspension was frozen again for 5 h and mixed with twice-concentrated IBB buffer (10 mL). The samples were centrifuged (4 °C, 4200 g, 25 min) and the clear supernatant was collected and subsequently loaded into iminobiotin sepharose beads column for affinity purification. The column was washed with IBB buffer (10 column volume) and then eluted with MES buffer (0.1 M, pH 5.5) three times to afford purified Sav variants. The concentration of purified Sav variants was determined by nanodrop and the solution was diluted with MES buffer (0.1 M, pH 5.5) to a defined concentration of 6.3 µM of the tetrameric Sav, corresponding to 25 µM FBS.

ZYP-5052 medium composition: salts: KH_2PO_4 (50 mM), Na_2HPO_4 (50 mM), $(NH_4)_2SO_4$ (5 mM); sugars: glucose monohydrate (2.77 mM), lactose (6.13 mM) with glycerol (0.5%); yeast triptone mix (5 g/L yeast extract, 10 g/L triptone); $MgSO_4$ (1 mM).

Lysis buffer composition: Lysozyme (1 mg/mL) and DNAse I in Tris(HCl) buffer (20 mM, pH 7.5).

IBB buffer composition: $NaHCO_3$ (50 mM, pH 10.8) and NaCl (500 mM).

5.2. Sequence of selected Sav muntants

Amino acid sequence of Sav S112I:

(M)ASMTGGQQMGRDQAGITGTWYNQLGSTFIVTAGADGALTGTYESAVGNAESRY
VLTGRYDSAPATDGSGTALGWTVAWKNNYRNAHSATTWSGQYVGGAEARINTQW
LLTIGTTEANAWKSTLVGHDTFTKVKPSAASIDAAKKAGVNNGNPLDAVQQ

Amino acid sequence of Sav S112I-K121R:

(M)ASMTGGQQMGRDQAGITGTWYNQLGSTFIVTAGADGALTGTYESAVGNAESRY
VLTGRYDSAPATDGSGTALGWTVAWKNNYRNAHSATTWSGQYVGGAEARINTQW
LLTIGTTEANAWRSTLVGHDTFTKVKPSAASIDAAKKAGVNNGNPLDAVQQ

Amino acid sequence of Sav S112V:

(M)ASMTGGQQMGRDQAGITGTWYNQLGSTFIVTAGADGALTGTYESAVGNAESRY
VLTGRYDSAPATDGSGTALGWTVAWKNNYRNAHSATTWSGQYVGGAEARINTQW
LLTVGTTEANAWKSTLVGHDTFTKVKPSAASIDAAKKAGVNNGNPLDAVQQ

Amino acid sequence of Sav S112V-K121R:

(M)ASMTGGQQMGRDQAGITGTWYNQLGSTFIVTAGADGALTGTYESAVGNAESRY
VLTGRYDSAPATDGSGTALGWTVAWKNNYRNAHSATTWSGQYVGGAEARINTQW
LLTVGTTEANAWRSTLVGHDTFTKVKPSAASIDAAKKAGVNNGNPLDAVQQ

6. Catalysis with purified Sav variants

6.1. General procedure for catalysis with purified Sav variants

To a 1.5 mL HPLC glass vial, a stock solution of purified Sav (tetrameric, initial concentration 6.3 μM, 300 μL) in MES buffer (0.1 M, pH 5.5) was added. Then, a stock solution of Ir cofactor (initial concentration 750 μM, 5 μL in TFE) was added and the mixture was incubated at room temperature for 5 min. The substrate (initial concentration 166.7 mM, 45 μL in TFE) and more TFE (150 μL) were added successively. The vial was sealed and incubated with a thermoshaker for 16 h (35 °C, at 750 rpm). A solution of internal standard (1,3,5-trimethoxybezene, initial concentration 1 mM, 200 μL) in EA was added and the mixture was extracted with EA (HPLC grade, 2 × 200 μL). The combined organic layers were dried over $MgSO_4$. The extract was filtered and analyzed by GC-FID with an Astec® CHIRALDEX™ G-TA Capillary GC Column.

GC methods:

Column: Astec® CHIRALDEX™ G-TA Capillary GC Column (50 m 0.25 mm 0.12 μm);
Inlet mode: Split; Split ratio: 10:1, 12.0 mL/min
Carrier gas: Helium (Constant flow, pressure: 27.5 psi; Flow: 1.2 ml/min; Average Velocity: 28 cm/sec)
Column oven temperature: 150 °C (isothermal mode for 27.5 min)

Table S2. Experiment data set of genetic optimization

Sav variants	yield (%)	TON	ee (%)
w/o Sav	0.7	13.9	--
WT	2.2	44.6	33
S112A	6.6	131.2	31
S112V	8.1	161.1	48
S112I	8.5	171.0	86
S112L	1.8	35.1	71
S112M	0.3	5.2	17
S112F	0.5	9.8	13
S112Y	0.6	11.0	40
S112W	0.8	16.9	45
S112N	0.6	12.9	11
S112Q	0.6	11.9	10
S112T	5.3	105.4	53

S112D	0.5	9.8	-8
S112E	0.7	13.8	11
S112H	0.5	10.4	22
S112K	0.4	7.7	8
S112R	0.5	9.2	-9
S112C	0.7	14.8	49
S112G	0.3	6.4	-19
S112P	0.7	14.6	13
K121A	0.8	16.8	0
K121V	0.5	9.6	-18
K121I	0.5	11.0	-11
K121L	0.6	11.7	-9
K121M	0.2	4.2	-24
K121F	0.4	7.6	-8
K121Y	0.3	5.5	-22
K121W	0.4	7.0	-19
K121N	1.7	33.4	3
K121Q	0.3	5.4	-19
K121S	0.8	15.2	2
K121T	0.8	16.4	-11
K121D	1.1	21.0	12
K121E	0.5	11.0	1
K121H	0.2	3.0	9
K121R	1.8	36.6	30
K121C	0.5	10.1	-7
K121G	0.7	14.1	-4
K121P	0.6	12.9	-9
S112I-K121A	0.4	8.2	19
S112I-K121L	0.5	9.4	25
S112-K121F	0.4	7.8	2
S112I-K121Y	0.4	8.0	15
S112I-K121N	0.5	10.7	35
S112I-K121Q	0.4	8.7	31

S112I-K121S	3.4	68.1	57
S112I-K121E	0.4	8.0	8
S112I-K121H	0.4	7.6	9
S112I-K121K	8.5	171.0	79
S112I-K121R	15.4	303.5	86
S112I-K121C	0.4	8.0	15
S112I-K121G	0.4	7.9	7
S112I-K121P	0.4	7.2	5

6.2. Assignment of the absolute configuration of the lactam **2**

To assign the absolute configuration of lactam **2**, commercial (*S*)- 5-isopropylpyrrolidine-2-one **2** (97% ee, CAS: 139564-41-3) was purchased from Activate Scientific and analyzed with GC using the GC method for catalysis.

(a)

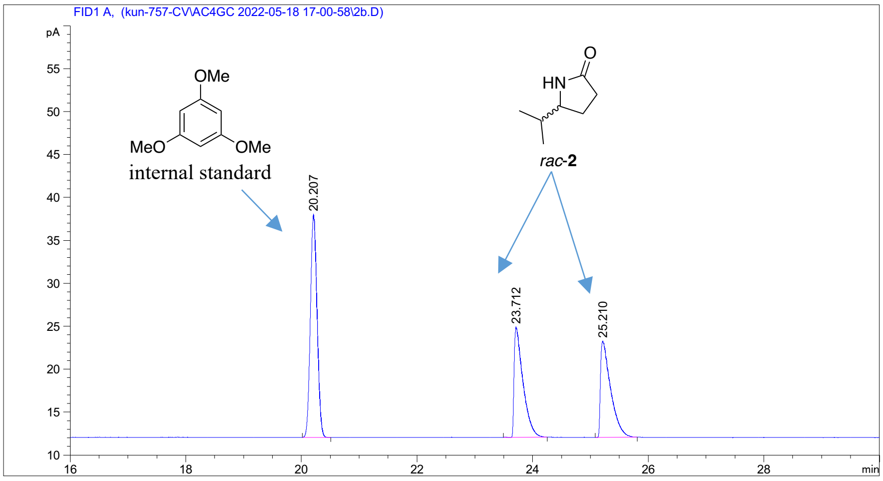

(b)

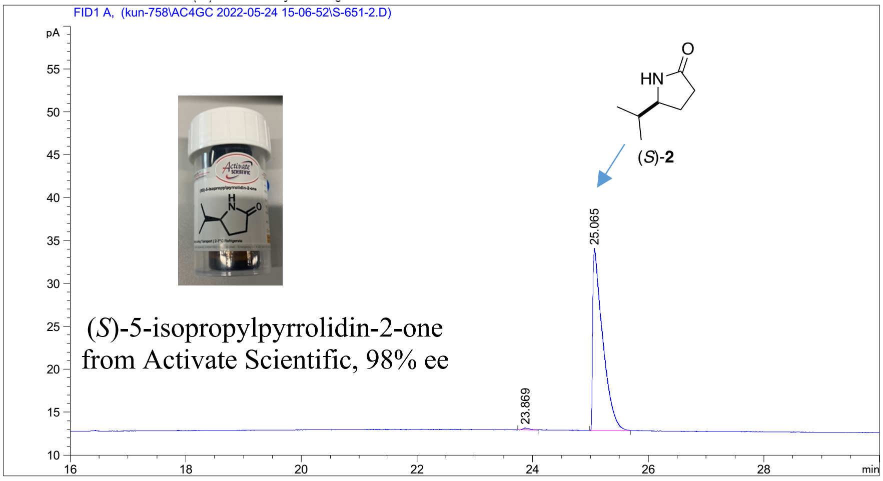

(c)

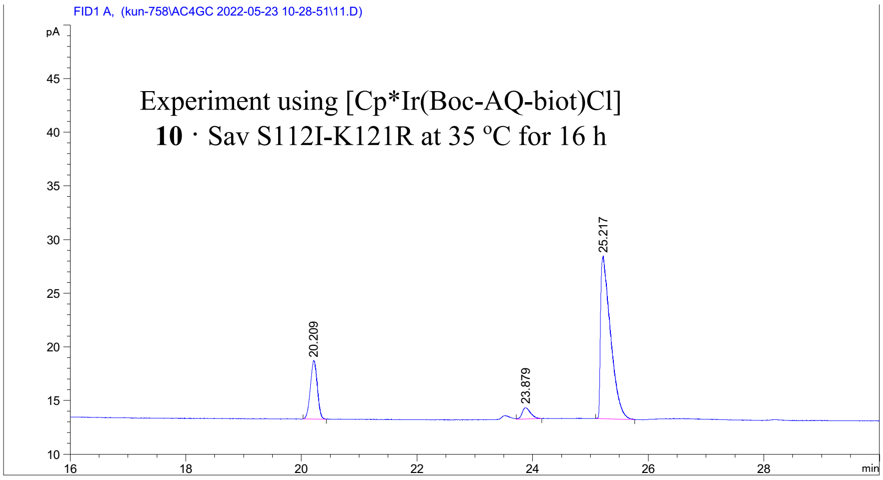

Figure S4. GC analysis for (a) racemic lactam *rac*-**2**; (b) commercial (*S*)-5-isopropylpyrrolidine-2-one **2**; (c) experiment using [Cp*Ir(Boc-AQ-biot)Cl] **10** · Sav S112I-K121R with 1,3,5-trimethoxybenzene as internal standard.

7. Procedures for preparative scale reactions

7.1. Preparative experiment using dioxazolone **13**

Lyophilized Sav S112I was added into MES buffer (19.0 mL, 0.1 M, pH 5.5) in a flask. The solution was stirred until the protein was completely dissolved. Then, a solution of [Cp*Ir(Boc-AQ-biot)Cl] **10** (0.5 mol %, 2.00 mg in 1.0 mL TFE) was added and the mixture was incubated at room temperature for 5 min. The substrate **13** (100 mg, 0.47 mmol) and TFE (11.6 mL) were added successively. The mixture was stirred for 48 h at 10 °C. The reaction mixture was extracted with EA (2 × 20 mL). The organic layers were combined and dried over $MgSO_4$. The extract was filtered and the solvent was removed *in vacuo*. The residue was purified with silica gel flash chromatography (DCM/MeOH = 20:1) to afford lactam (*S*)-**14** (66 mg, 83%, 93% ee) as a white solid. The specific rotation of the product was measured $[\alpha]^{32}_{D}$ = -0.5 (*c* 0.4, $CHCl_3$), which indicates the absolute configuration of the product is (*S*)-**14** based on the reported optical rotation.[24]

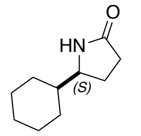

(*S*)-**14**

^{1}H NMR (500 MHz, $CDCl_3$): 6.40 (brs, 1H), 3.36 (q, J = 7.1 Hz, 1H), 2.39 – 2.22 (m, 2H), 2.21 – 2.11 (m, 1H), 1.87 – 1.60 (m, 6H), 1.35 – 1.07 (m, 4H), 1.04 – 0.83 (m, 2H).

^{13}C NMR (126 MHz, $CDCl_3$): 178.5, 59.6, 43.4, 30.3, 29.4, 28.7, 26.3, 25.8, 25.8, 25.0.

HRMS (ESI positive mode, m/z): calculated for $C_{10}H_{18}NO$ $[M+H]^+$ 168.1383; found 168.1382.

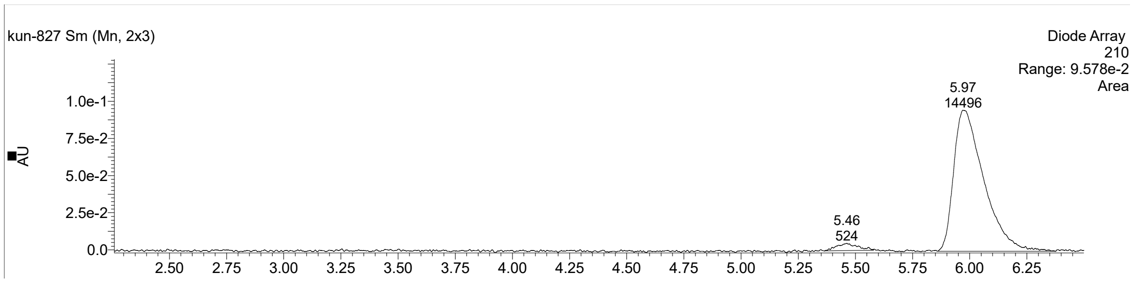

Figure S5. SFC trace of the purified lactam (*S*)-**14** (93% ee) obtained by the preparative experiment.

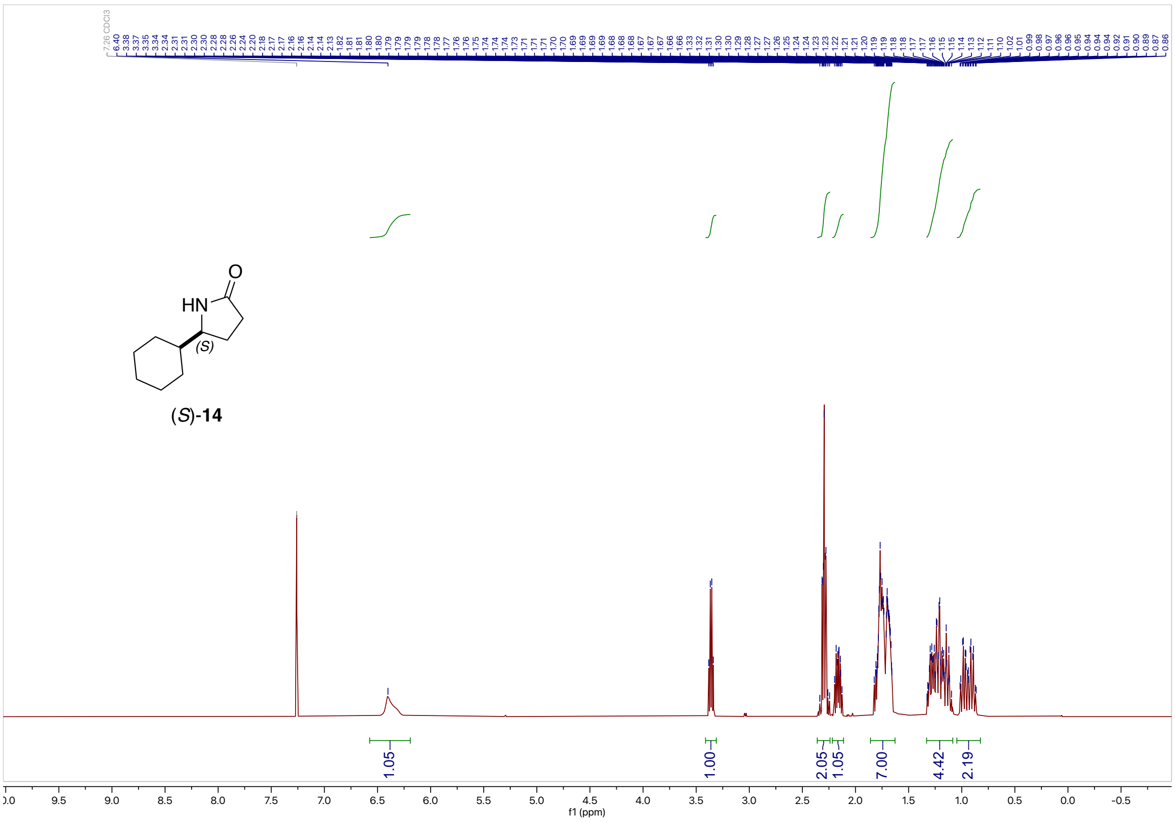

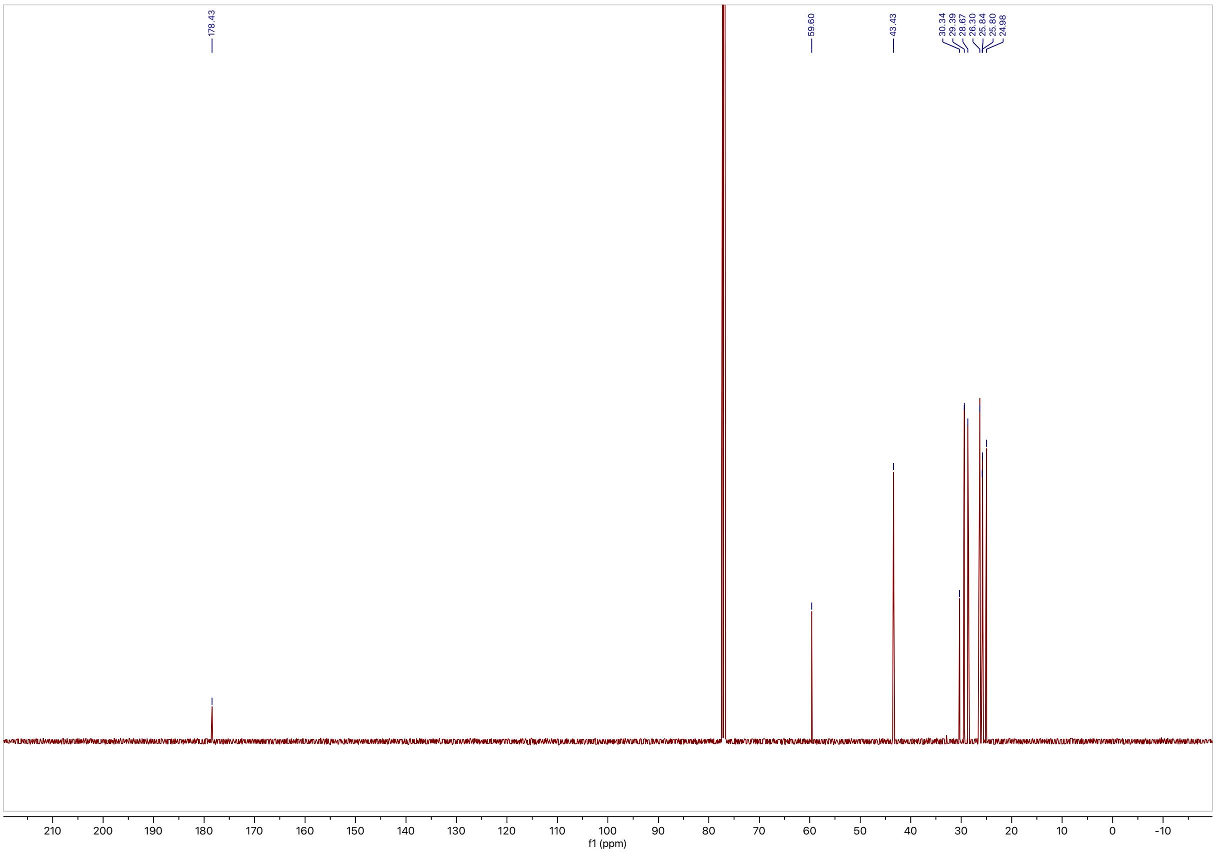

Figure S6. ^{1}H NMR and ^{13}C NMR of lactam (*S*)-**14** (93% ee) obtained by preparative experiment.

7.2. Semi-preparative experiment using dioxazolone **13**

Lyophilized Sav S112I (7.4 mg) was added into MES buffer (1.8 mL, 0.1 M, pH 5.5) in a flask. The solution was stirred until the protein was completely dissolved. Then, a solution of [Cp*Ir(Boc-AQ-biot)Cl] **10** (0.5 mol %, 0.2 mg in 200 μL TFE) was added and the mixture was incubated at room temperature for 5 min. The substrate **13** (9.5 mg, 0.045 mmol) and TFE (1.0 mL) were added successively. The mixture was stirred for 48 h at 10 °C. Biphenyl (6.9 mg, 45 mmol) was added to the reaction as internal standard. The reaction mixture was extracted with EA (2 × 2 mL). The organic layers were combined and dried over MgSO4. The extract was filtered, and the solvent was removed in vacuo. The lactam **14** was obtained in >90% yield determined by crude ^{1}H NMR of the reaction mixture.

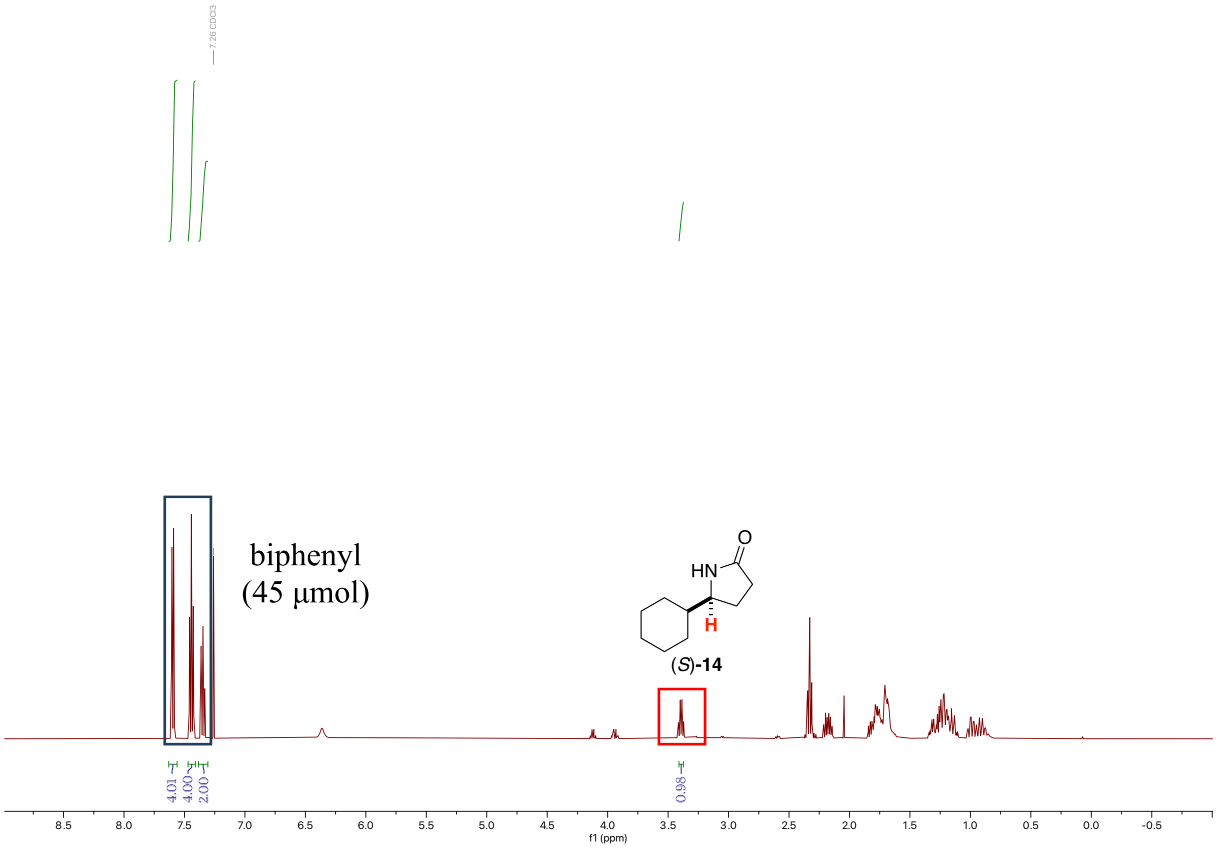

Figure S7. Crude ^{1}H NMR of the reaction mixture of the semi-preparative experiment.

7.3. Preparative experiment using dioxazolone **1** as substrate

A stock solution of Sav S112I-K121R in MES buffer (0.1 M, pH 5.5) was diluted with MES buffer (0.1 M, pH 5.5) to make a solution of Sav S112I-K121R (0.25 mM, 47 mL) in a flask. Then, a solution of [Cp*Ir(Boc-AQ-biot)Cl] **10** (0.5 mol %, 4.95 mg in 10.0 mL TFE) was added and the mixture was incubated at room temperature for 5 min. The substrate **1** (200 mg, 1.17 mmol) and TFE (21.0 mL) were added successively. The mixture was stirred for 48 h at 10 °C. The reaction mixture was extracted with EA (2 × 20 mL). The organic layers were combined and dried over $MgSO_4$. The extract was filtered and the solvent was removed *in vacuo*. The residue was purified with silica gel flash chromatography (DCM/MeOH = 20:1) to afford lactam (*S*)-**2** (80 mg, 55%, 91% ee) as a white solid.

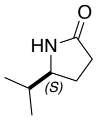

(*S*)-**2**

^{1}H NMR (500 MHz, $CDCl_3$) δ 6.67 (brs, 1H), 3.39 (q, *J* = 7.0 Hz, 1H), 2.33 (m, 2H), 2.20 – 2.10 (m, 1H), 1.76 (m, 1H), 1.64 (h, *J* = 6.7 Hz, 1H), 0.92 (dd, *J* = 22.3, 6.7 Hz, 6H).

^{13}C NMR (126 MHz, $CDCl_3$) δ 178.7, 60.7, 33.5, 30.6, 24.7, 18.8, 18.1.

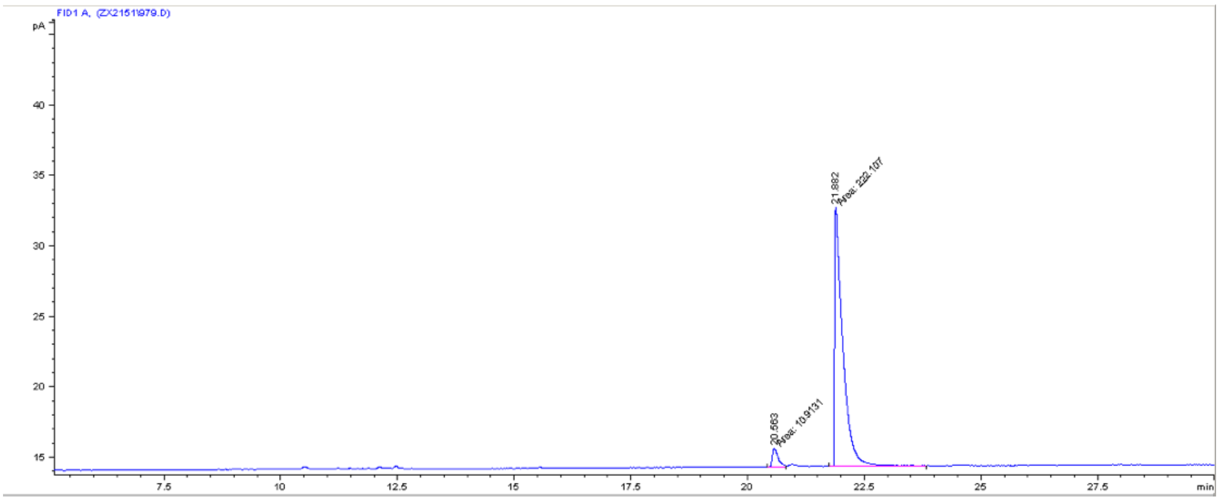

#	Time	Area	Height	Width	Area%	Symmetry
1	20.563	10.9	1.3	0.1408	4.683	0.482
2	21.882	222.1	18.4	0.2011	95.317	0.164

Figure S8. GC trace of the purified lactam (*S*)-**2** (91% ee) obtained by the preparative experiment.

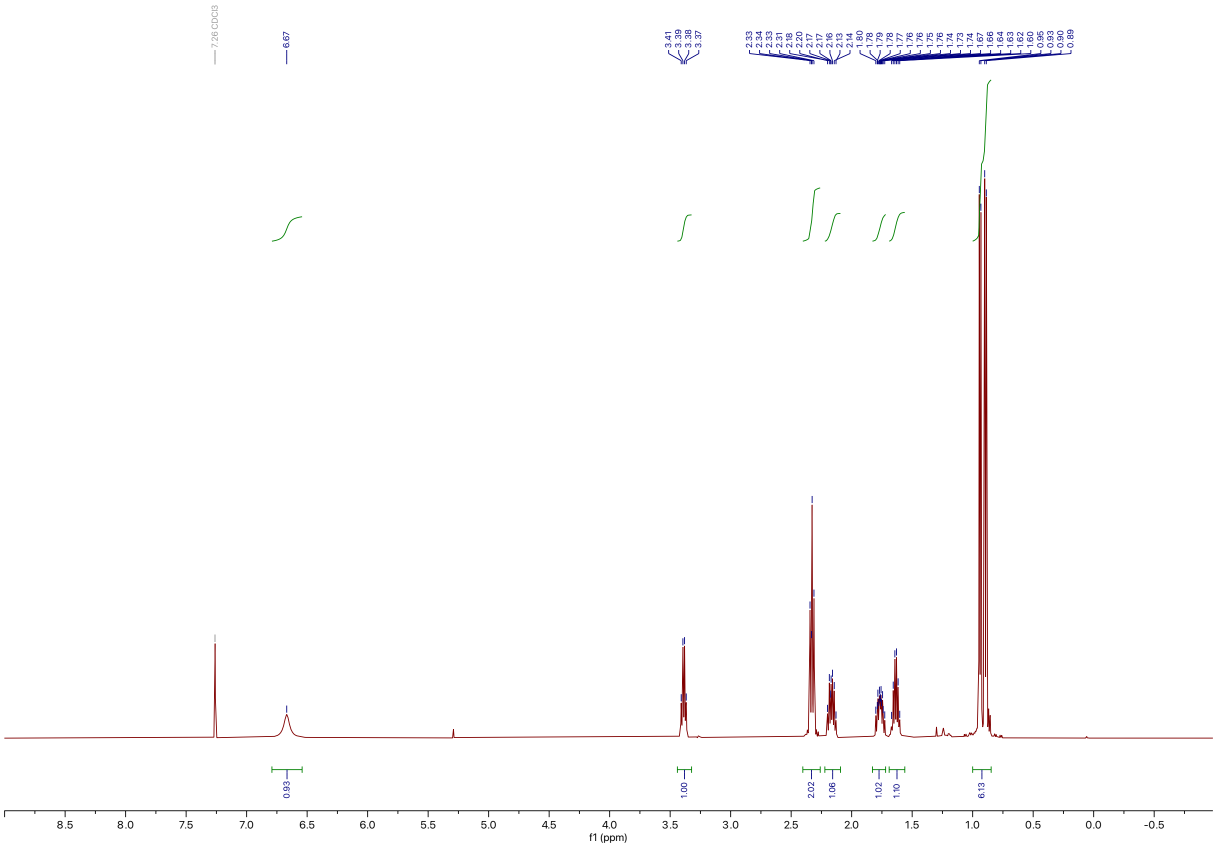

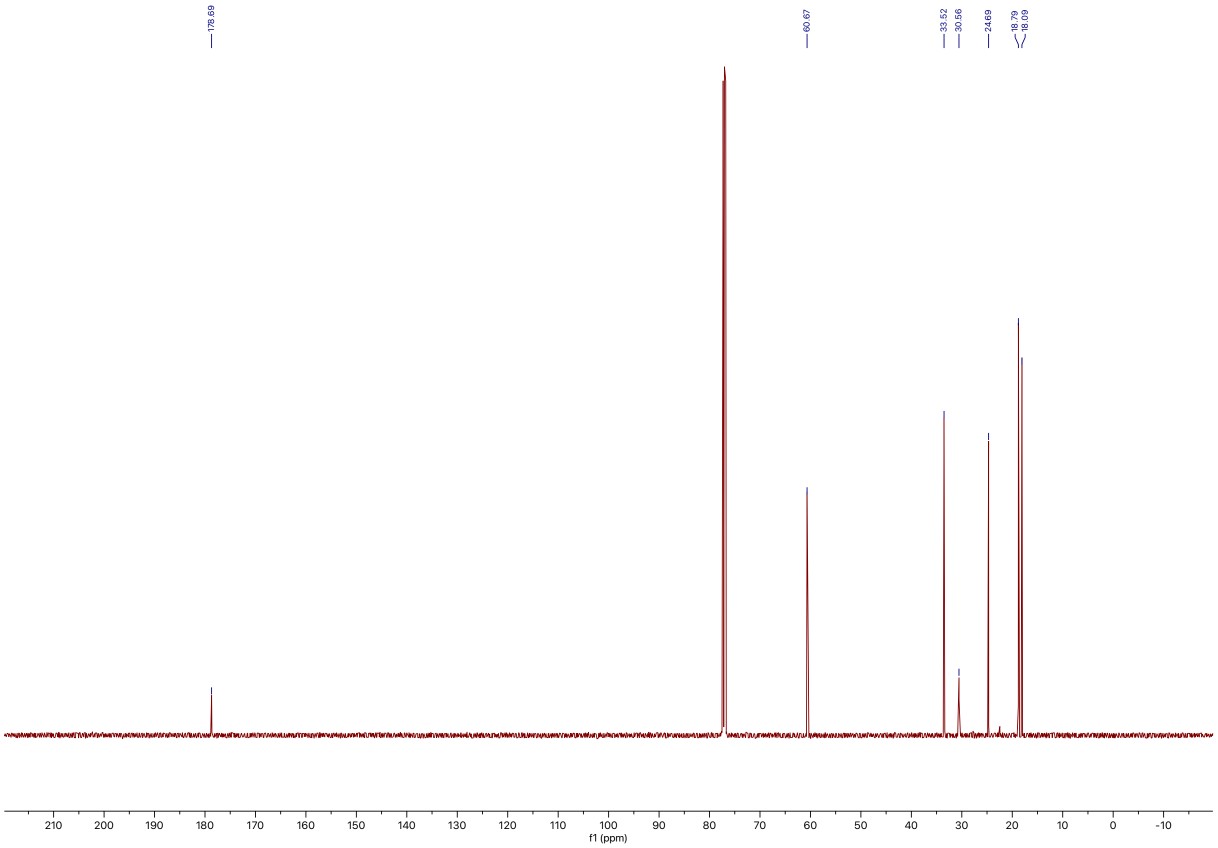

Figure S9. ^{1}H NMR and ^{13}C NMR of lactam (*S*)-**2** (91% ee) obtained by preparative experiment.

8. Supporting tables

Table S3. Optimization of reaction parameters for enantioselective C-H amidation of dioxazolone 1 using [Cp*Ir(Boc-AQ-biot)Cl] 10 · Sav S112I-K121R[a]

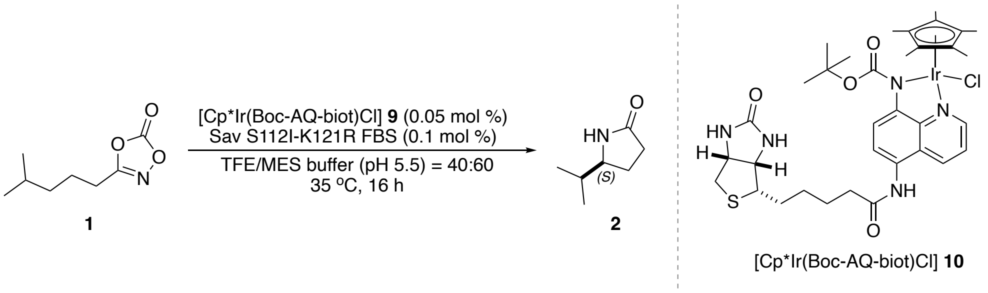

entry	variations of standard conditions	TON	ee (%)[b]
1	none[a]	304	86
2	HFIP as cosolvent	--	--
3	EtOH as cosolvent	97	74
4	MeOH as cosolvent	76	70
5	TFE/MES buffer = 20:80	244	74
6	MES buffer (pH 6.0)	160	84
7	[Sav FBS] = 7.5 μM	284	76
8	[Sav FBS] = 30 μM	281	76
9	10 °C, 48 h	363±13	89±0.2

[a]Standard conditions: dioxazolone [**1**] = 15 mM, [[Cp*Ir(Boc-AQ-biot)Cl] **10**] = 7.5 μM, [Sav FBS] = 15 μM, 200 μL of TFE, 300 μL of MES buffer (0.1 M, pH 5.5), 35 °C, 16 h. [b]ee was determined by chiral GC. Positive values indicate ee of (*S*)-**2**.

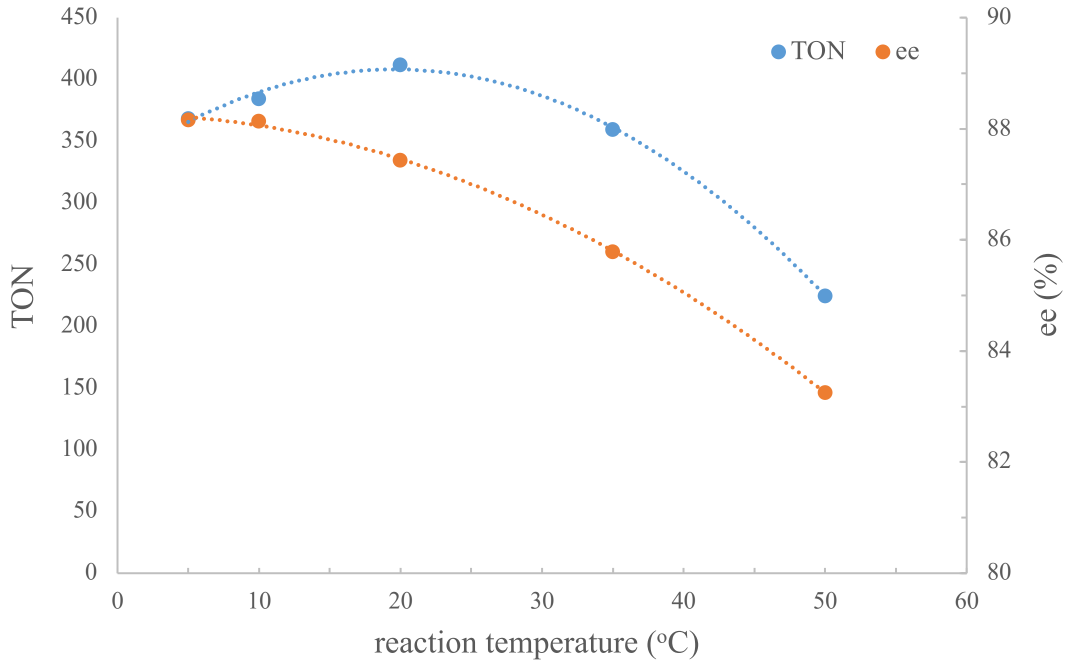

Figure S10. Effect of reaction temperature on TON (blue), and ee (orange).

Table S4. Optimization of ratio [[Cp*Ir(Boc-AQ-biot)Cl] 10]/[Sav S112I-K121R FBS] for enantioselective C-H amidation of dioxazolone 1[a]

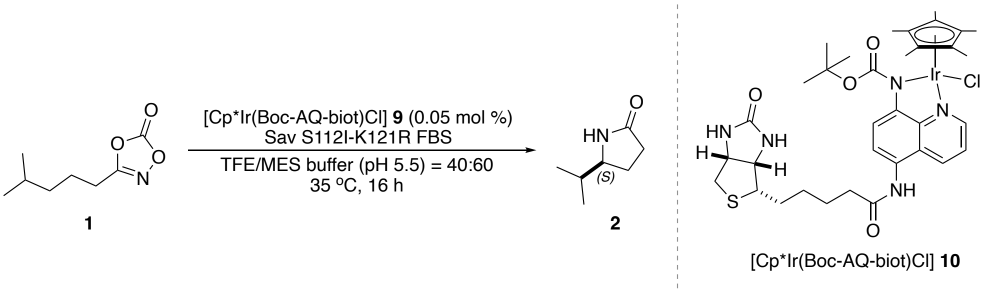

entry	[[Cp*Ir(Boc-AQ-biot)Cl] 10]:[Sav S112I-K121R FBS]	TON	ee (%)[b]
1	1:1	175	84
2	1:2	255	88
3	1:4	253	88
4	1:8	263	88

[a]Reaction conditions: dioxazolone [**1**] = 15 mM, [[Cp*Ir(Boc-AQ-biot)Cl] **10**] = 7.5 μM, 200 μL of TFE, 300 μL of MES buffer (0.1 M, pH 5.5), 35 °C, 16 h. For these experiments, the concentration of [Cp*Ir(Boc-AQ-biot)Cl] **10** remained unchanged. The concentration of Sav S112I-K121R FBS varied according to the ratio [[Cp*Ir(Boc-AQ-biot)Cl] **10**]:[Sav S112I-K121R FBS]. [b]ee was determined by chiral GC. Positive values indicate ee of (*S*)-**2**.

Table S5. Screening of purified Sav variants for the enantioselective C(sp^3)-H amidation of dioxazolone 1[a]

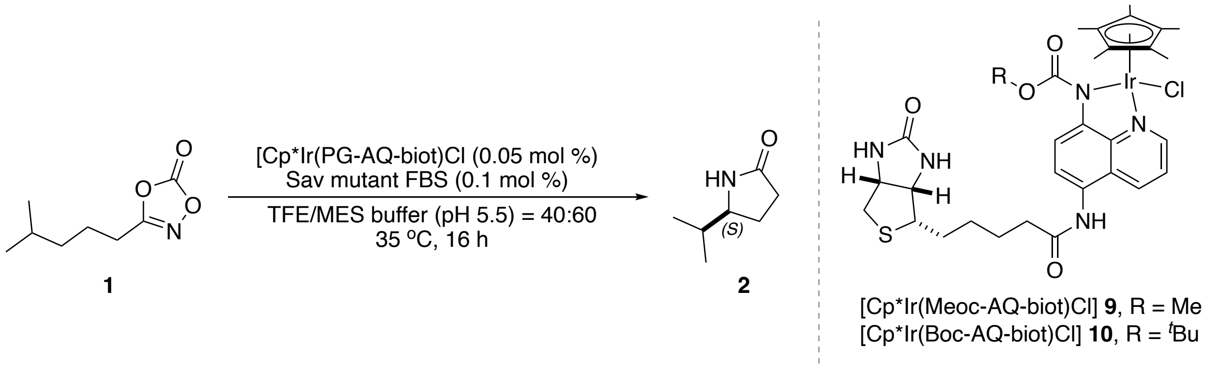

entry	Sav variants	[Cp*Ir(PG-AQ-biot)Cl]	TON	ee (%)[b]
1	WT	**10**	45	32
2	S112I	**10**	171	80
3	S112I-K121R	**10**	304	86
4	S112V	**10**	161	48
5	S112V-K121R	**10**	147	54
6	S112I	**9**	43	76
7	S112I-K121R	**9**	45	70
8	S112V	**9**	37	38
9	S112V-K121R	**9**	67	42

[a]Reaction condition: dioxazolone [**1**] = 15 mM, [[Cp*Ir(PG-AQ-biot)Cl]] = 7.5 μM, [Sav FBS] = 15 μM, 200 μL of TFE, 300 μL of MES buffer (0.1 M, pH 5.5), 35 °C, 16 h. [b]ee was determined by chiral GC. Positive values indicate ee of (*S*)-**2**.

Table S6. Screening of purified Sav variants for the enantioselective C(sp^3)-H amidation of dioxazolone 11.[a]

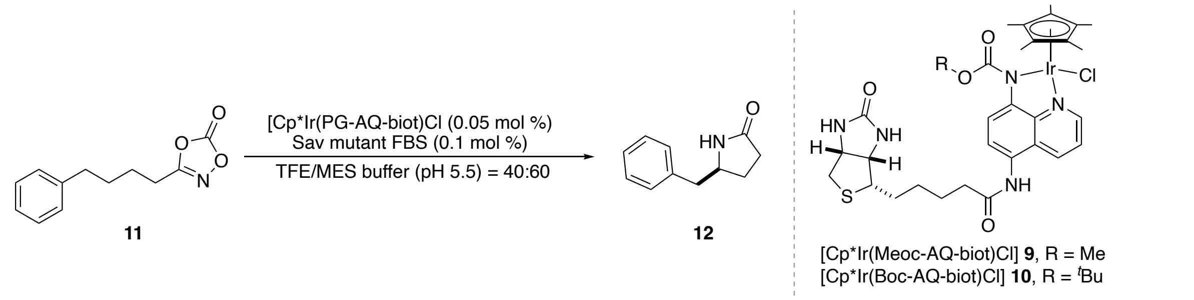

entry	Sav variants	[Cp*Ir(PG-AQ-biot)Cl]	temp. (°C)	TON	ee (%)[b]
1	WT	**10**	35 (16 h)	187	-12
2	S112I	**10**	35 (16 h)	539	-36
3	S112I-K121R	**10**	35 (16 h)	416	-58
4	S112V	**10**	35 (16 h)	580	2
5	S112V-K121R	**10**	35 (16 h)	602	2
6	WT	**9**	35 (16 h)	--	--
7	S112I	**9**	35 (16 h)	280	-16
8	S112I-K121R	**9**	35 (16 h)	265	-34
9	S112V	**9**	35 (16 h)	97	-2
10	S112V-K121R	**9**	35 (16 h)	78	14
11	S112I	**10**	10 (48 h)	1025	-36
12	S112I-K121R	**10**	10 (48 h)	537±13	-56±0.7
13	S112V	**10**	10 (48 h)	744	-12
14	S112V-K121R	**10**	10 (48 h)	692	-8

[a]Reaction condition: dioxazolone [**11**] = 15 mM, [[Cp*Ir(PG-AQ-biot)Cl]] = 7.5 μM, [Sav FBS] = 15 μM, 200 μL of TFE, 300 μL of MES buffer (0.1 M, pH 5.5).[b]ee was determined by SFC. Positive values indicate that the first peak corresponding to one of the enantiomers of lactam **12** on the SFC spectrum was bigger than the second peak.

Table S7. Screening of purified Sav variants for the enantioselective C(*sp*^{3})-H amidation of dioxazolone 13[a]

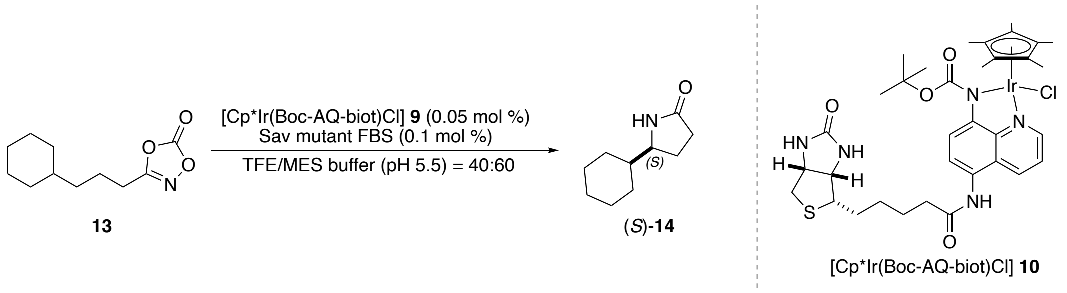

entry	Sav variants	temp. (°C)	TON	ee (%)[b]
1	WT	35 (16 h)	110	40
2	S112I	35 (16 h)	228	-92
3	S112I-K121R	35 (16 h)	504	-56
4	S112V	35 (16 h)	371	-10
5	S112V-K121R	35 (16 h)	548	-33
6	S112I	10 (48 h)	474±30	-92±1.4

Reaction condition: dioxazolone [**13**] = 15 mM, [[Cp*Ir(PG-AQ-biot)Cl]] = 7.5 μM, [Sav FBS] = 15 μM, 200 μL of TFE, 300 μL of MES buffer (0.1 M, pH 5.5). [b]ee was determined by SFC. Positive values indicate ee of (*R*)-**14**, negative values indicate ee of (*S*)-**14**.

Table S8. Screening of purified Sav variants for the enantioselective C(sp^3)-H amidation of dioxazolone 15[a]

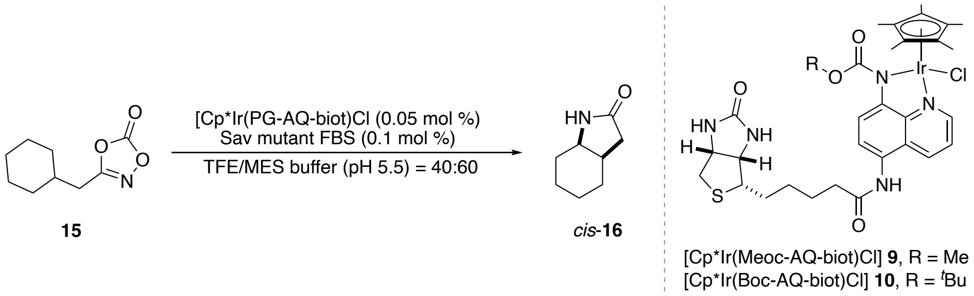

entry	Sav variants	[Cp*Ir(PG-AQ-biot)Cl]	temp. (°C)	TTON	dr (*cis-/trans-*)	ee (%)[b]
1	WT	**10**	35 (16 h)	47	85:15	-32
2	S112I	**10**	35 (16 h)	120	85:15	-66
3	S112I-K121R	**10**	35 (16 h)	142	86:14	-52
4	S112V	**10**	35 (16 h)	135	84:16	2
5	S112V-K121R	**10**	35 (16 h)	170	85:15	-8
6	WT	**9**	35 (16 h)	27	81:19	0
7	S112I	**9**	35 (16 h)	61	77:23	-58
8	S112I-K121R	**9**	35 (16 h)	105	81:19	-40
9	S112V	**9**	35 (16 h)	42	77:23	-36
10	S112V-K121R	**9**	35 (16 h)	54	81:19	0
11	S112I	**10**	10 (48 h)	205±5	86:14	-68±1.0
12	S112I-K121R	**10**	10 (48 h)	107	87:13	-50
13	S112V	**10**	10 (48 h)	157	85:15	-50
14	S112V-K121R	**10**	10 (48 h)	189	86:14	-6

[a]Reaction condition: dioxazolone [**15**] = 15 mM, [[Cp*Ir(PG-AQ-biot)Cl]] = 7.5 μM, [Sav FBS] = 15 μM, 200 μL of TFE, 300 μL of MES buffer (0.1 M, pH 5.5). [b]ee was determined by chiral GC. Negative values indicate that the first peak corresponding to one of the enantiomers of lactam *cis*-**16** on the GC spectrum was smaller than the second peak.

Table S9. Screening of purified Sav variants for the enantioselective C(sp^3)-H amidation of dioxazolone 17[a]

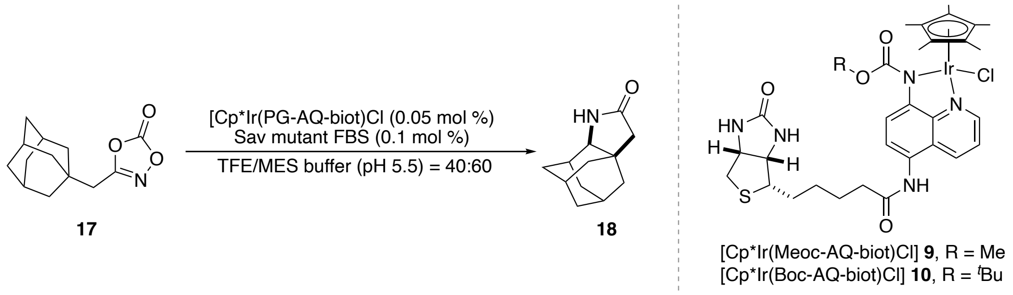

entry	Sav variants	[Cp*Ir(PG-AQ-biot)Cl]	temp. (°C)	TON	ee (%)
1	WT	**10**	35 (16 h)	38	40
2	S112I	**10**	35 (16 h)	82	66
3	S112I, K121R	**10**	35 (16 h)	82	62
4	S112V	**10**	35 (16 h)	105	62
5	S112V, K121R	**10**	35 (16 h)	116	38
6	WT	**9**	35 (16 h)	16	8
7	S112I	**9**	35 (16 h)	32	78
8	S112I, K121R	**9**	35 (16 h)	53	58
9	S112V	**9**	35 (16 h)	24	68
10	S112V, K121R	**9**	35 (16 h)	24	16
11	S112I	**10**	10 (48 h)	50±1	86±1.2
12	S112I, K121R	**10**	10 (48 h)	50	74
13	S112V	**10**	10 (48 h)	30	72
14	S112V, K121R	**10**	10 (48 h)	26	34

[a]Reaction condition: dioxazolone [**17**] = 15 mM, [[Cp*Ir(PG-AQ-biot)Cl]] = 7.5 μM, [Sav FBS] = 15 μM, 200 μL of TFE, 300 μL of MES buffer (0.1 M, pH 5.5). [b]ee was determined by SFC. Positive values indicate that the first peak corresponding to one of the enantiomers of lactam **18** on the SFC spectrum was bigger than the second peak.

Table S10. Screening of purified Sav variants for the enantioselective C(sp^3)-H amidation of dioxazolone 19[a]

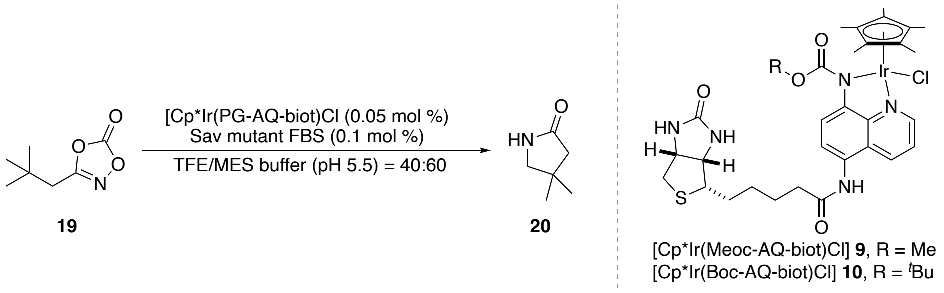

entry	Sav variants	[Cp*Ir(PG-AQ-biot)Cl]	temp. (°C)	TON
1	WT	**10**	35 (16 h)	18
2	S112I	**10**	35 (16 h)	73
3	S112I-K121R	**10**	35 (16 h)	50
4	S112V	**10**	35 (16 h)	122
5	S112V-K121R	**10**	35 (16 h)	132
6	S112I	**9**	35 (16 h)	51
7	S112I-K121R	**9**	35 (16 h)	147
8	S112V	**9**	35 (16 h)	57
9	S112V-K121R	**9**	35 (16 h)	44
10	S112V-K121R	**10**	10 (48 h)	179±1
11[b]	S112V-K121R	**10**	10 (48 h)	112

[a]Reaction condition: dioxazolone [**19**] = 15 mM, [[Cp*Ir(PG-AQ-biot)Cl]] = 7.5 μM, [Sav FBS] = 15 μM, 200 μL of TFE, 300 μL of MES buffer (0.1 M, pH 5.5). [b][[Cp*Ir(Boc-AQ-biot)Cl] **10**] = 75 μM, [Sav FBS] = 150 μM were used.

Table S11. Screening using purified Sav variants for the enantioselective C(sp^3)-H amidation of dioxazolone 21[a]

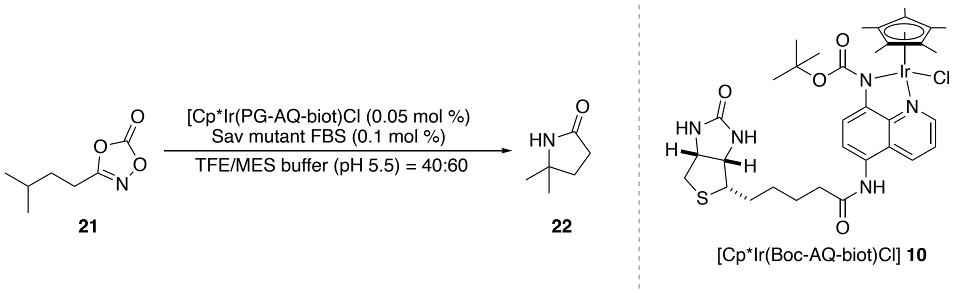

entry	Sav variants	temp. (°C)	TON
1	WT	35 (16 h)	90
2	S112I	35 (16 h)	212
3	S112I-K121R	35 (16 h)	419
4	S112V	35 (16 h)	147
5	S112V-K121R	35 (16 h)	768±25
6	S112V-K121R	10 (48 h)	757

[a]Reaction condition: dioxazolone [21] = 15 mM, [[Cp*Ir(Boc-AQ-biot)Cl] **10**] = 7.5 μM, [Sav FBS] = 15 μM, 200 μL of TFE, 300 μL of MES buffer (0.1 M, pH 5.5), 35 °C, 16 h.

Table S12. Screening of purified Sav variants for the enantioselective C(sp^3)-H amidation of dioxazolone 23[a]

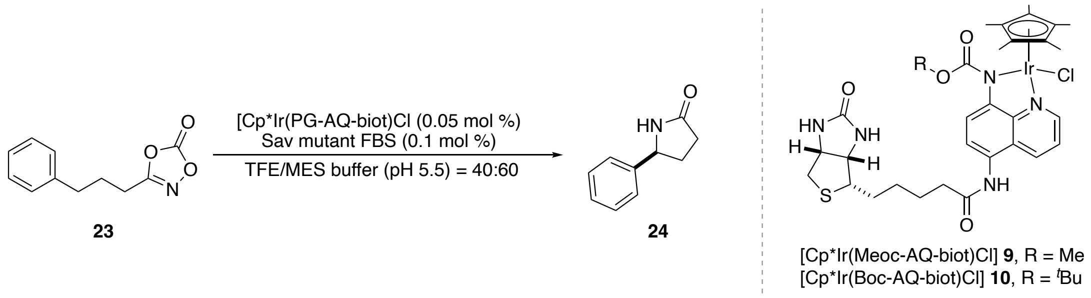

entry	Sav variants	[Cp*Ir(PG-AQ-biot)Cl]	temp. (°C)	TON	ee (%)[b]
1	WT	**10**	35 (16 h)	42	36
2	S112I	**10**	35 (16 h)	101	60
3	S112I-K121R	**10**	35 (16 h)	254	56
4	S112V	**10**	35 (16 h)	128	60
5	S112V-K121R	**10**	35 (16 h)	293	50
6	S112I	**9**	35 (16 h)	64	64
7	S112I-K121R	**9**	35 (16 h)	96	34
8	S112V	**9**	35 (16 h)	70	80
9	S112V-K121R	**9**	35 (16 h)	176	64
10	S112V	**10**	10 (48 h)	164±10	68±0.4
11	S112V	**9**	10 (48 h)	99±1	84±0.8

[a]Reaction condition: dioxazolone [**23**] = 15 mM, [[Cp*Ir(PG-AQ-biot)Cl]] = 7.5 μM, [Sav FBS] = 15 μM, 200 μL of TFE, 300 μL of MES buffer (0.1 M, pH 5.5). [b]ee was determined by SFC. Positive values indicate that the first peak corresponding to one of the enantiomers of lactam **24** on the SFC spectrum was bigger than the second peak.

Table S13. Screening of purified Sav S112V-K121R-G48X variants for the enantioselective C(sp^3)-H amidation of dioxazolone 20[a]

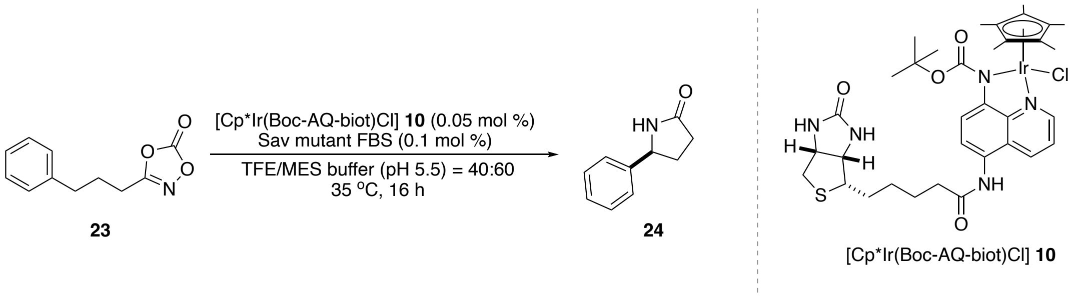

entry	Sav variants	TON	ee (%)[b]
1	S112V-K121R-G48A	55	5
2	S112V-K121R-G48L	26	-11
3	S112V-K121R-G48M	16	-9
4	S112V-K121R-G48F	29	-8
5	S112V-K121R-G48S	36	-16
6	S112V-K121R-G48T	29	-9
7	S112V-K121R-G48D	34	-11
8	S112V-K121R-G48E	32	-8
9	S112V-K121R-G48N	29	-3
10	S112V-K121R-G48Q	28	-10
11	S112V-K121R-G48H	11	-9
12	S112V-K121R-G48K	27	-4
13	S112V-K121R-G48R	27	-7
14	S112V-K121R-G48G	270	49
15	S112V-K121R-G48P	37	-14

[a]Reaction condition: dioxazolone [**23**] = 15 mM, [[Cp*Ir(BOC-AQ-biot)Cl] **10**] = 7.5 μM, [Sav FBS] = 15 μM, 200 μL of TFE, 300 μL of MES buffer (0.1 M, pH 5.5). [b]ee was determined by SFC. Positive values indicate that the first peak corresponding to one of the enantiomers of lactam **24** on the SFC spectrum was bigger than the second peak.

9. Supporting Figures

(a)

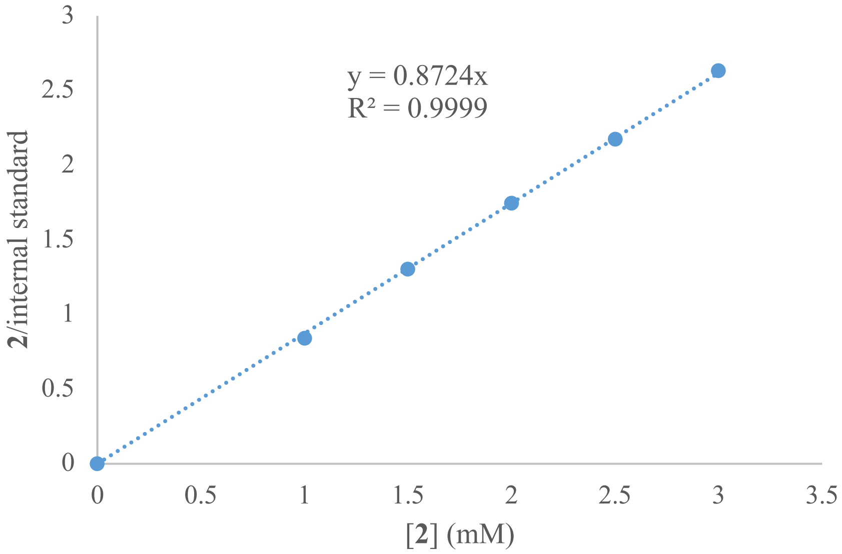

(b)

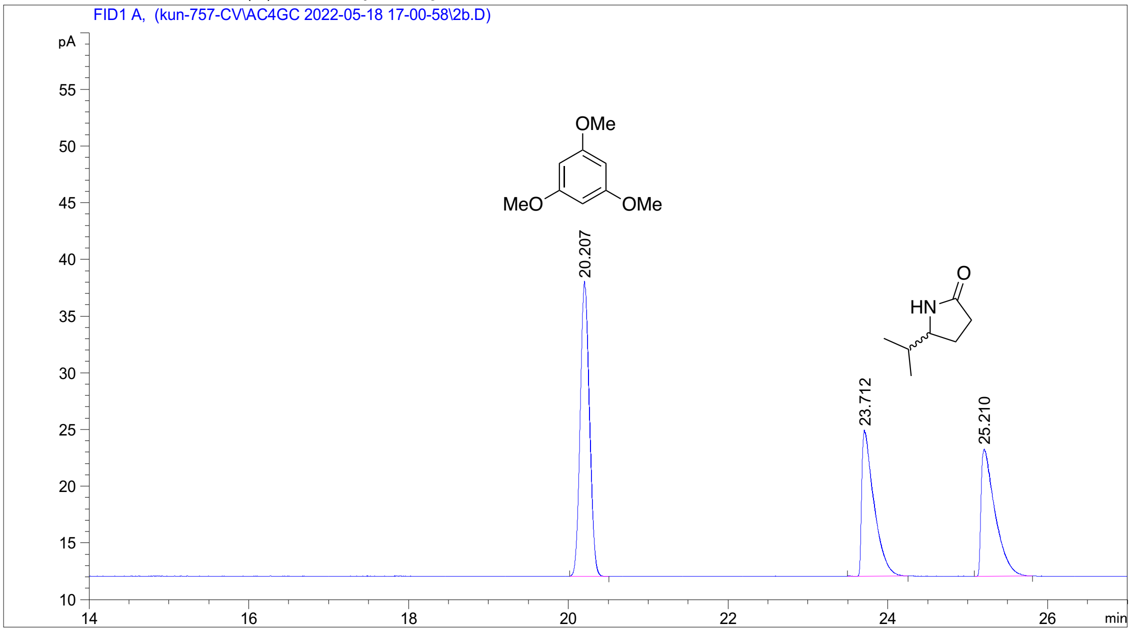

(c)

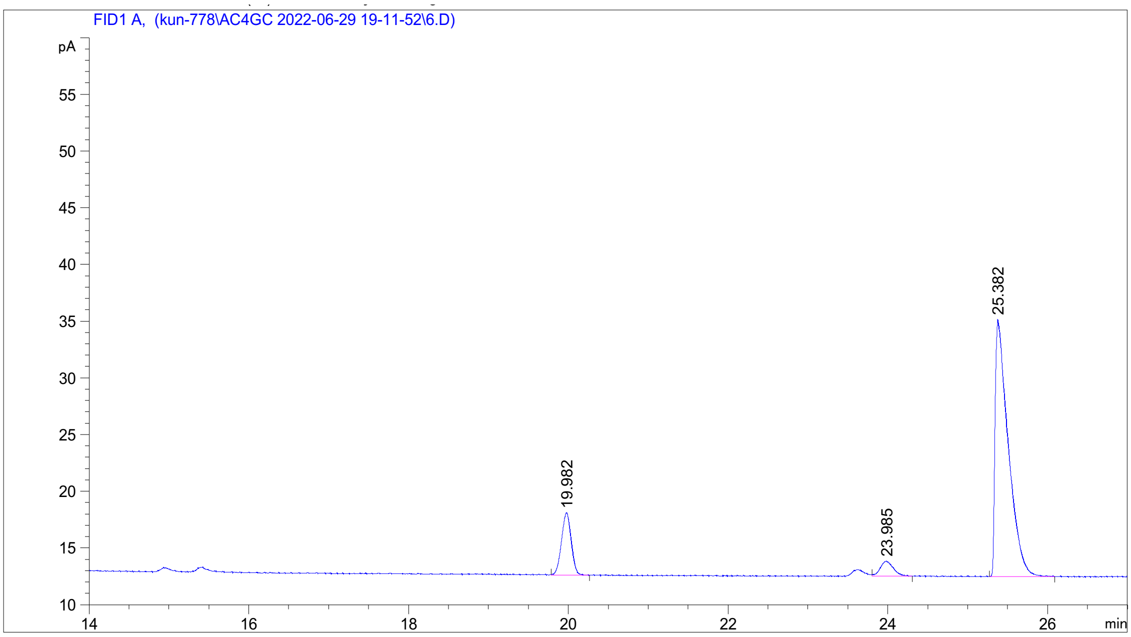

Peak #	RetTime [min]	Type	Width [min]	Area [pA*s]	Area %
9	19.982	BB	0.1352	46.12712	14.41805
10	23.985	VB	0.1542	15.08755	4.71595
11	25.382	BB	0.1592	258.71149	80.86600

Figure S11. (a) Calibration curve used to determine TON for lactam **2**. (b) GC-analysis of racemic lactam **2**. (c) GC-analysis resulting from a reaction catalyzed by [Cp*Ir(Boc-AQ-biot)Cl] **10** · Sav S112I-K121R. GC method: column: Astec® CHIRALDEX™ G-TA Capillary GC Column (50 m 0.25 mm 0.12 μm); carrier gas: He; flow: 1.2 mL/min; oven temperature: isothermal, 150 °C for 27.5 min. Internal standard: 1,3,5-trimethoxybezene (initial concentration: 1 mM).

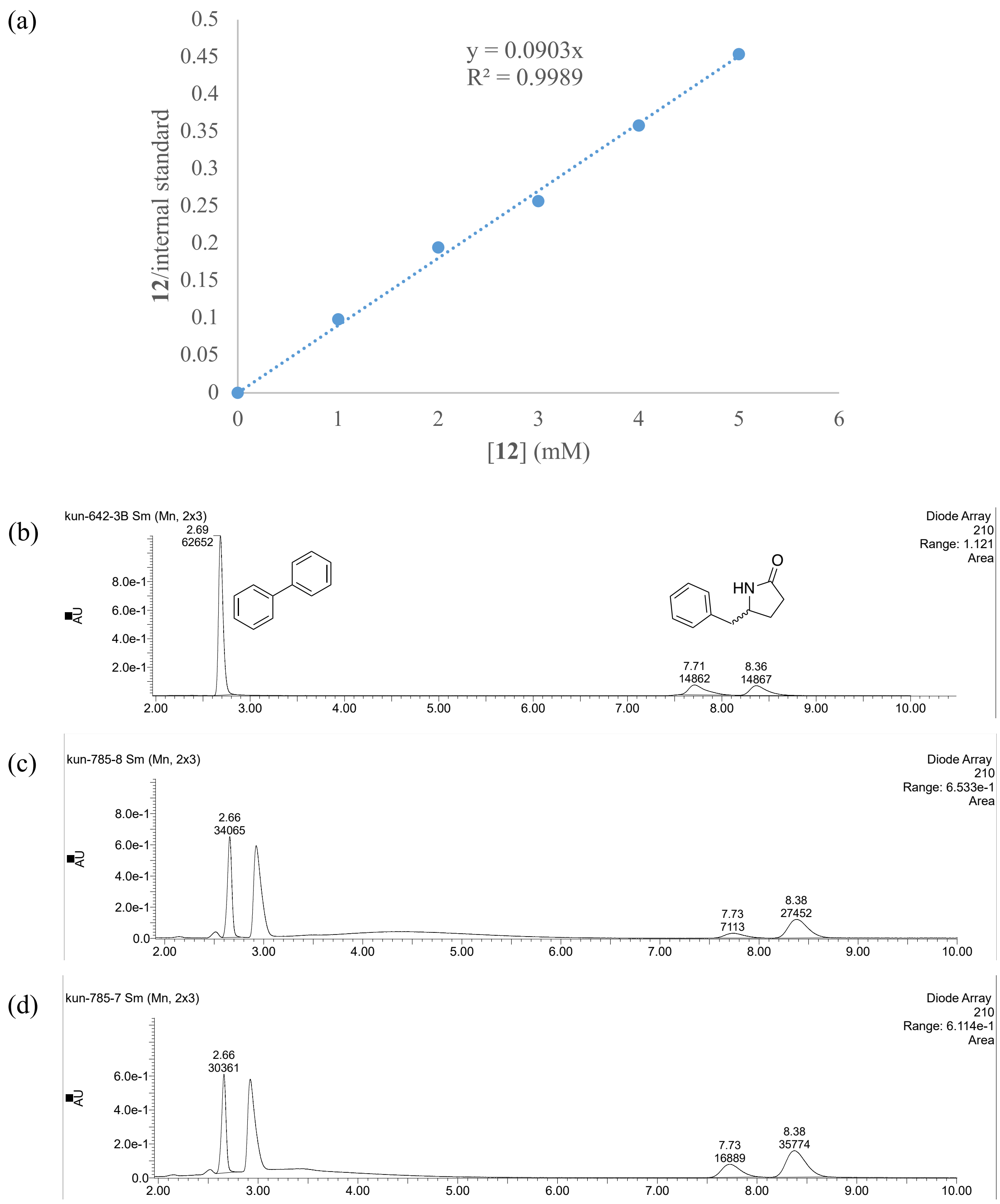

Figure S12. (a) Calibration curve used to determine TON for lactam **12**. (b) SFC-analysis of racemic lactam **12**. (c) SFC-analysis resulting from a reaction catalyzed by [Cp*Ir(Boc-AQ-biot)Cl] **10** · Sav S112I-K121R. (d) SFC-analysis resulting from a reaction catalyzed by [Cp*Ir(Boc-AQ-biot)Cl] **10** · Sav S112I. SFC method: Chiralpak IB©, 4.6 × 250 mm; isocratic, 10% ^{i}PrOH in CO_2, 2 mL/min, 210 nm. Internal standard: biphenyl (initial concentration: 2 mM).

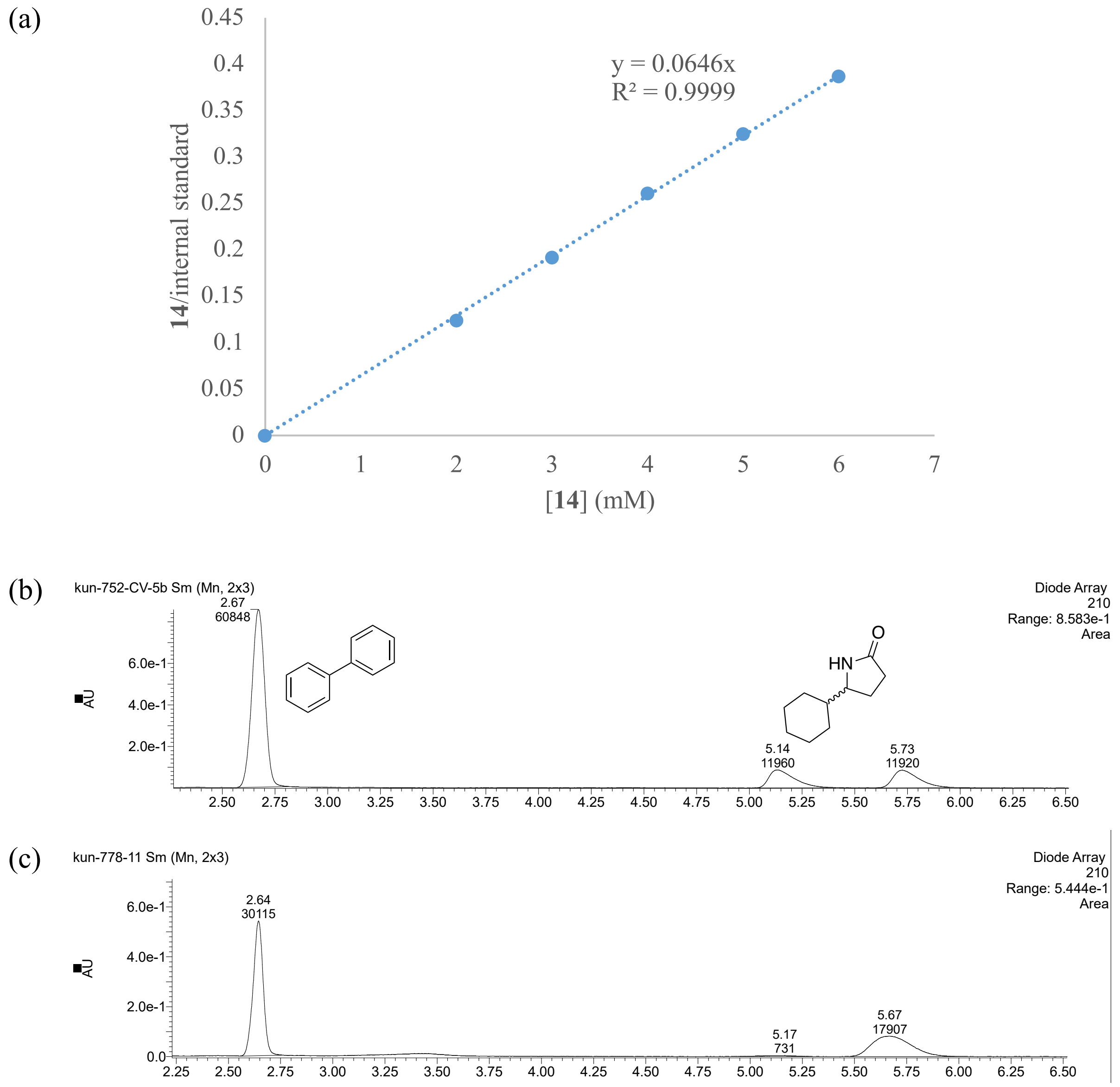

Figure S13. (a) Calibration curve used to determine TON for lactam **14**. (b) SFC-analysis of racemic lactam **14**. (c) SFC-analysis resulting from a reaction catalyzed by [Cp*Ir(Boc-AQ-biot)Cl] **10** · Sav S112I. SFC method: Chiralpak IB©, 4.6 × 250 mm; isocratic, 10% ^{i}PrOH in CO_2, 2 mL/min, 210 nm. Internal standard: biphenyl (initial concentration: 1 mM).

(a)

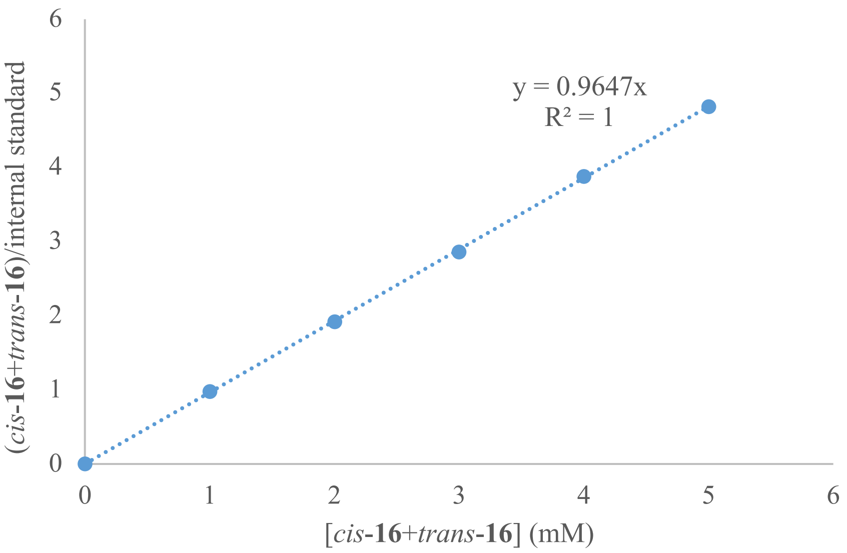

y = 0.9647x
R² = 1
(cis-16+trans-16)/internal standard
[cis-16+trans-16] (mM)
0
1
2
3
4
5
6

(b)

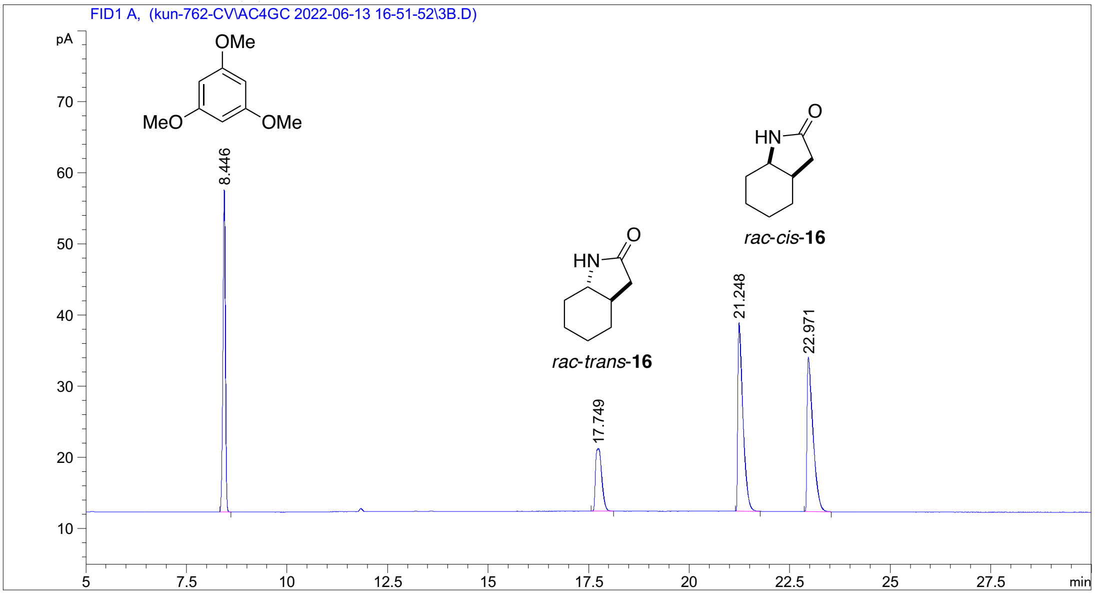

FID1 A, (kun-762-CV\AC4GC 2022-06-13 16-51-52\3B.D)
pA
OMe
MeO
OMe
HN
O
rac-trans-16
rac-cis-16
8.446
17.749
21.248
22.971
min

(c)

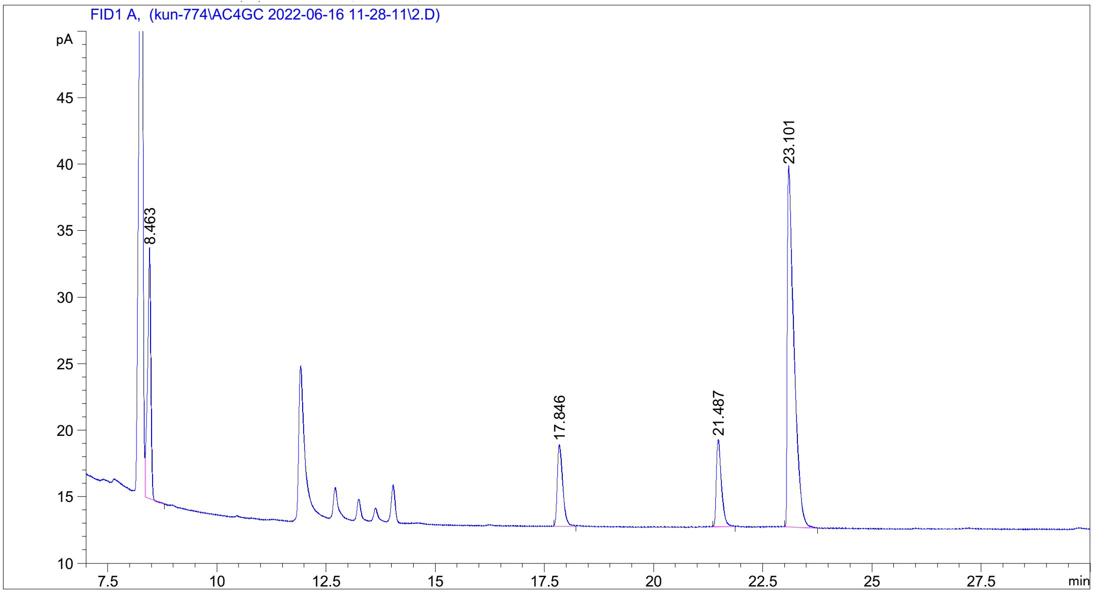

FID1 A, (kun-774\AC4GC 2022-06-16 11-28-11\2.D)
pA
8.463
17.846
21.487
23.101
min

Peak #	RetTime [min]	Type	Width [min]	Area [pA*s]	Area %
9	8.463	VB	0.0778	99.50069	20.20280
10	17.846	BV	0.1334	53.20022	10.80187
11	21.487	BB	0.1230	53.28671	10.81943
12	23.101	BB	0.1500	286.52182	58.17590

Figure S14. (a) Calibration curve used to determine TTON for lactam **16**. (b) GC-analysis of racemic lactam **16**. (c) GC-analysis resulting from a reaction catalyzed by [Cp*Ir(Boc-AQ-biot)Cl] **10** · Sav S112I. GC method: column: Astec® CHIRALDEX™ G-TA Capillary GC Column (50 m 0.25 mm 0.12 µm); carrier gas: He; flow: 1.2 mL/min; oven temperature: isothermal, 170 °C for 30 min. Internal standard: 1,3,5-trimethoxybezene (initial concentration: 1 mM).

(a)

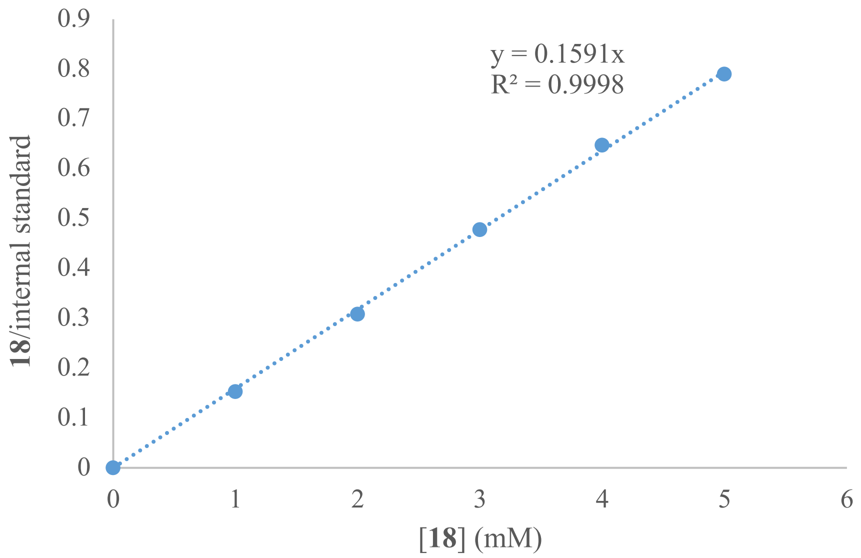

(b)

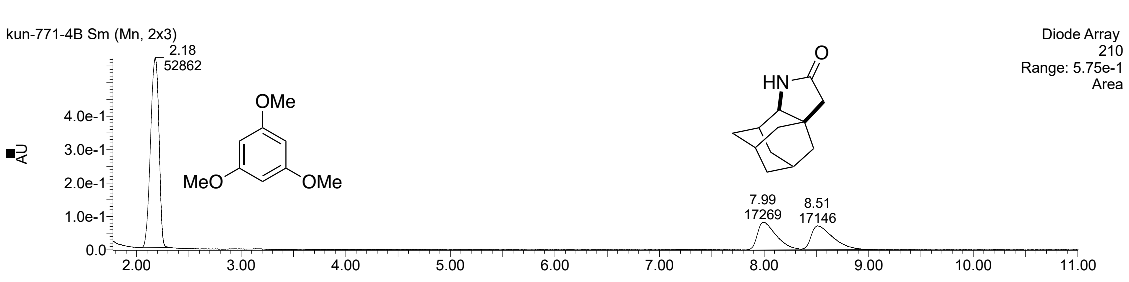

(c)

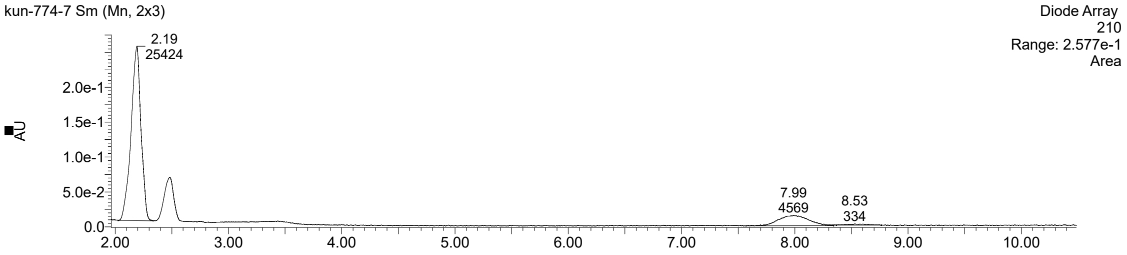

Figure S15. (a) Calibration curve used to determine TON for lactam **18**. (b) SFC-analysis of racemic lactam **18**. (c) SFC-analysis resulting from a reaction catalyzed by [Cp*Ir(Boc-AQ-biot)Cl] **10** · Sav S112I. SFC method: Chiralpak IB©, 4.6 × 250 mm; isocratic, 10% ^{i}PrOH in CO_2, 2 mL/min, 210 nm. Internal standard: 1,3,5-trimethoxybezene (initial concentration: 0.5 mM).

(a)

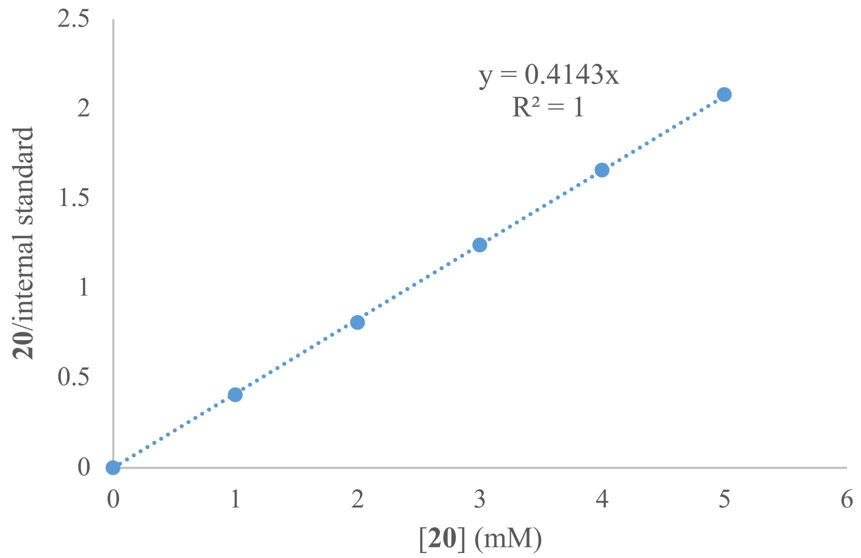
y = 0.4143x
R² = 1
20/internal standard
[20] (mM)
0
0.5
1
1.5
2
2.5
0
1
2
3
4
5
6

(b)

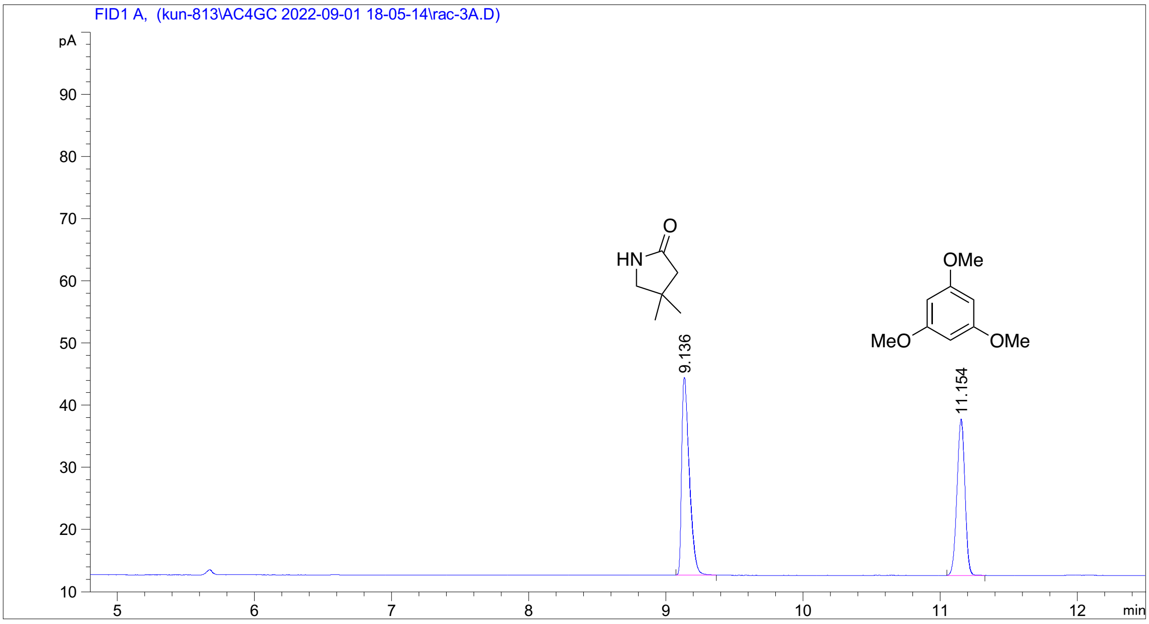
FID1 A, (kun-813\AC4GC 2022-09-01 18-05-14\rac-3A.D)
pA
9.136
11.154
HN
O
OMe
MeO
OMe
min

(c)

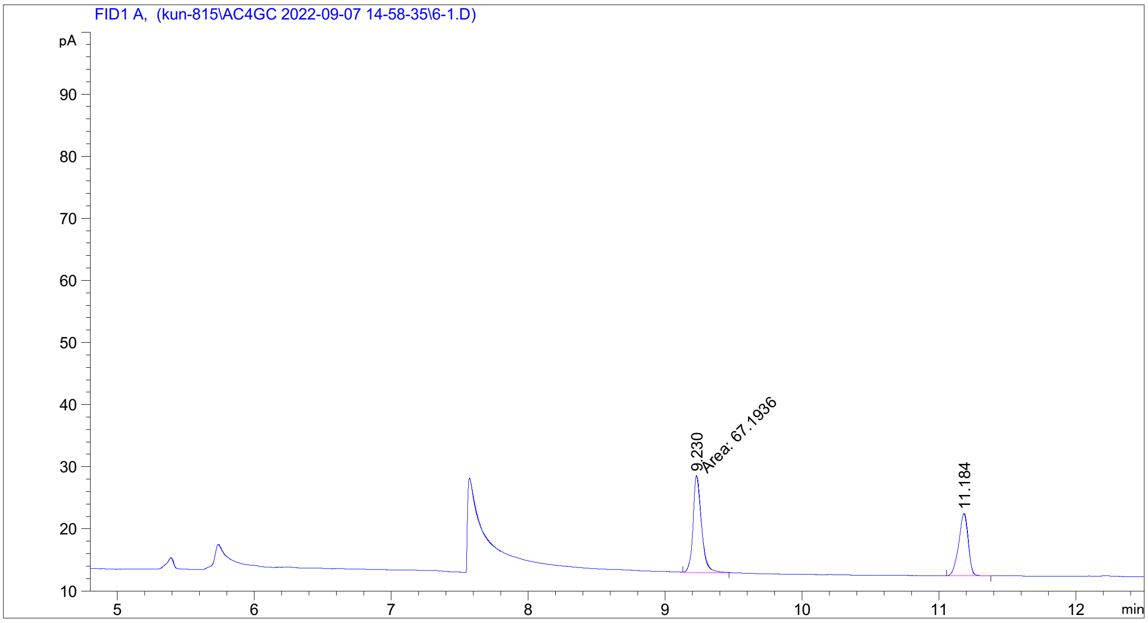

Peak #	RetTime [min]	Type	Width [min]	Area [pA*s]	Area %
9	9.230	MM	0.0717	67.19357	58.18556
10	11.184	BB	0.0742	48.28795	41.81444

(d)

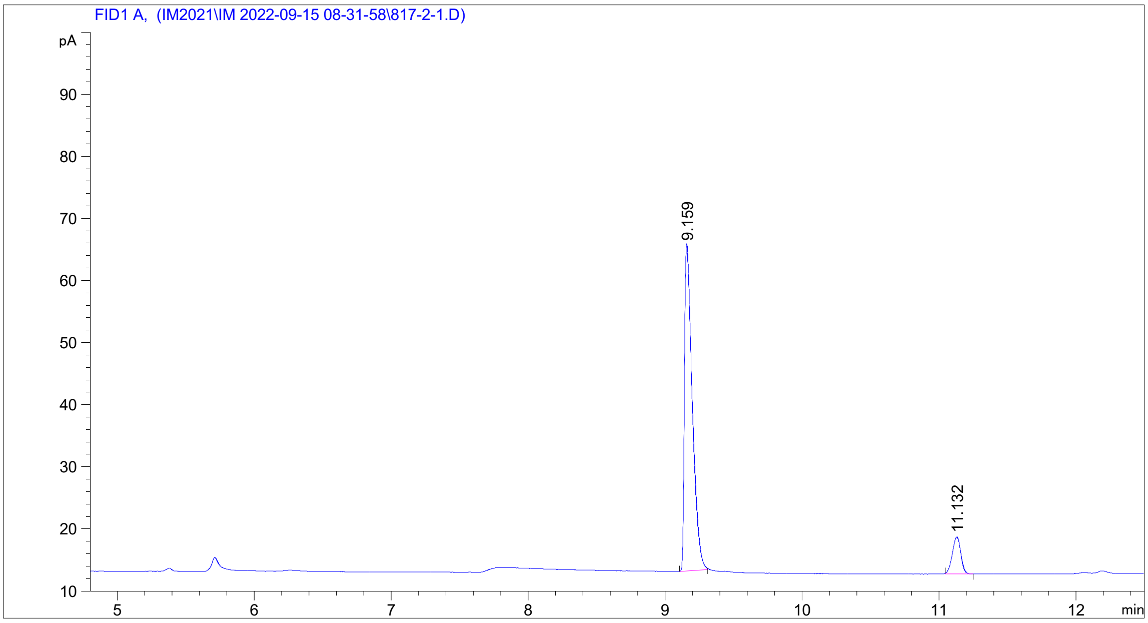

Peak #	RetTime [min]	Type	Width [min]	Area [pA*s]	Area %
9	9.159	BB	0.0613	208.53917	89.09055
10	11.132	BB	0.0670	25.53634	10.90945

Figure S16. (a) Calibration curve used to determine TON for lactam **20**. (b) GC-analysis of lactam **20**. (c) GC-analysis resulting from a reaction catalyzed by [Cp*Ir(Boc-AQ-biot)Cl] **10** · Sav S112V- K121R. GC method: column: Astec® CHIRALDEX™ G-TA Capillary GC Column (50 m 0.25 mm 0.12 μm); carrier gas: He; flow: 1.2 mL/min; oven temperature: isothermal, 160 ºC for 12.5 min. Internal standard: 1,3,5-trimethoxybezene (initial concentration: 1 mM).

(a)

(b)

(c)

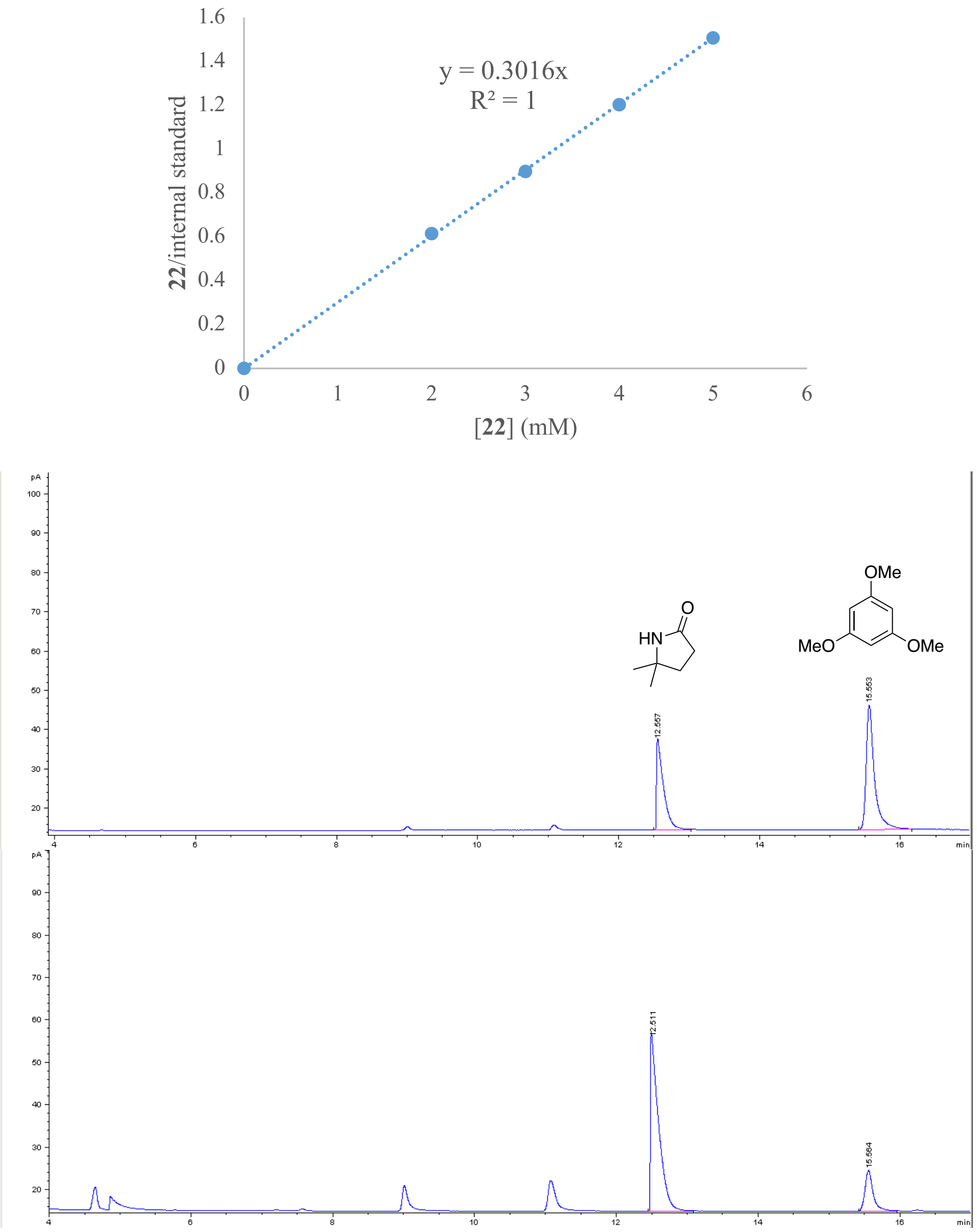

#	Time	Area	Height	Width	Area%	Symmetry
1	12.511	332.8	40.6	0.1117	81.279	0.206
2	15.564	76.6	9.6	0.1192	18.721	0.697

Figure S17. (a) Calibration curve used to determine TON for lactam **22**. (b) GC-analysis of lactam **22**. (c) GC-analysis resulting from a reaction catalyzed by [Cp*Ir(Boc-AQ-biot)Cl] **10** · Sav S112V- K121R. GC method: column: Astec® CHIRALDEX™ G-TA Capillary GC Column (50 m 0.25 mm 0.12 µm); carrier gas: He; flow: 1.2 mL/min; oven temperature: isothermal, 150 °C for 17 min. Internal standard: 1,3,5-trimethoxybezene (initial concentration: 2 mM).

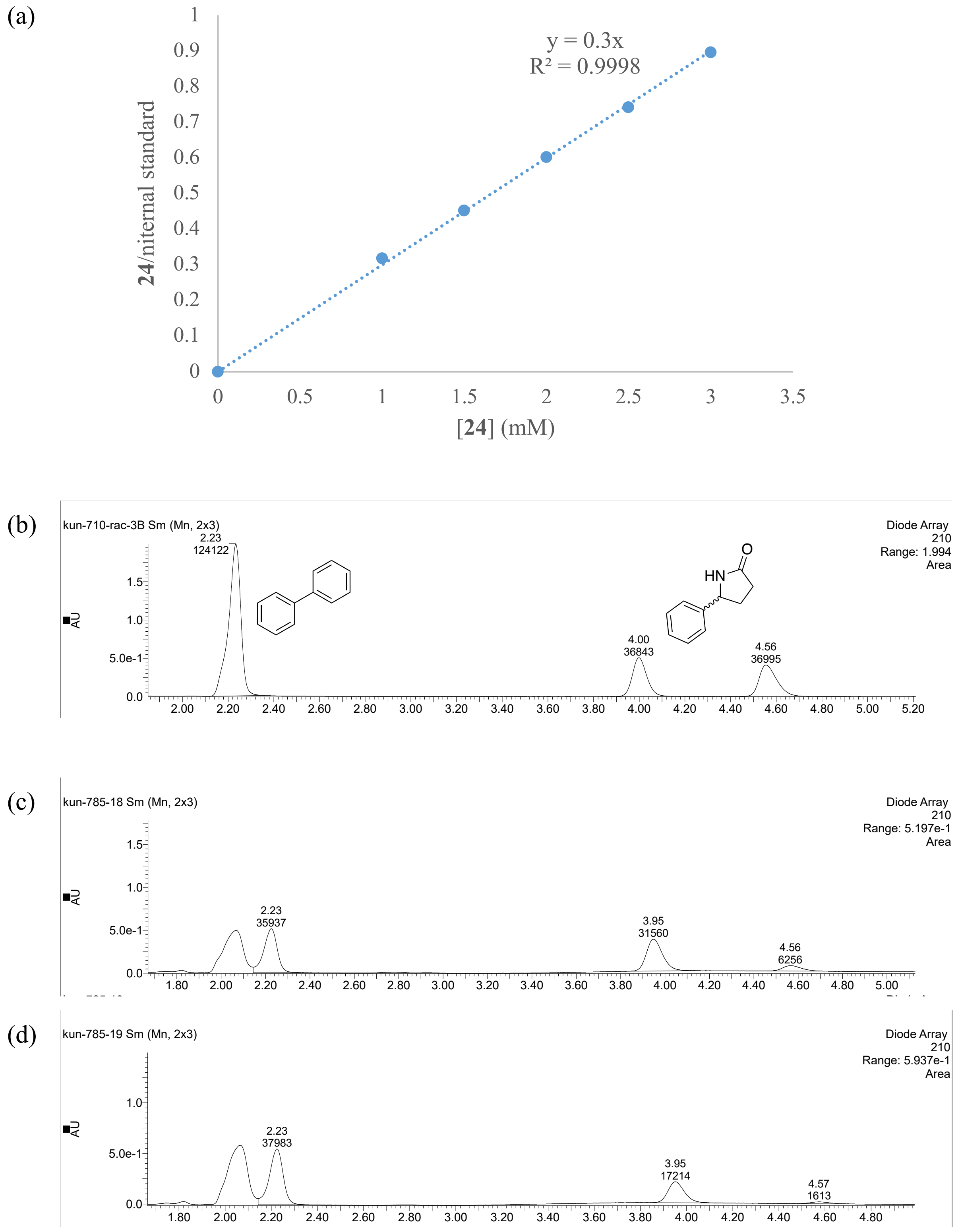

Figure S18. (a) Calibration curve used to determine TON for lactam **24**. (b) SFC-analysis of racemic lactam **24**. (c) SFC-analysis resulting from a reaction catalyzed by [Cp*Ir(Boc-AQ-biot)Cl] **10** · Sav S112V. (d) SFC-analysis resulting from a reaction catalyzed by [Cp*Ir(Meoc-AQ-biot)Cl] **9** · Sav S112V. SFC method: Chiralpak IA©, 4.6 × 250 mm; isocratic, 20% ^{i}PrOH in CO_2, 2 mL/min, 210 nm. Internal standard: biphenyl (initial concentration: 2 mM).

10. NMR Spectra

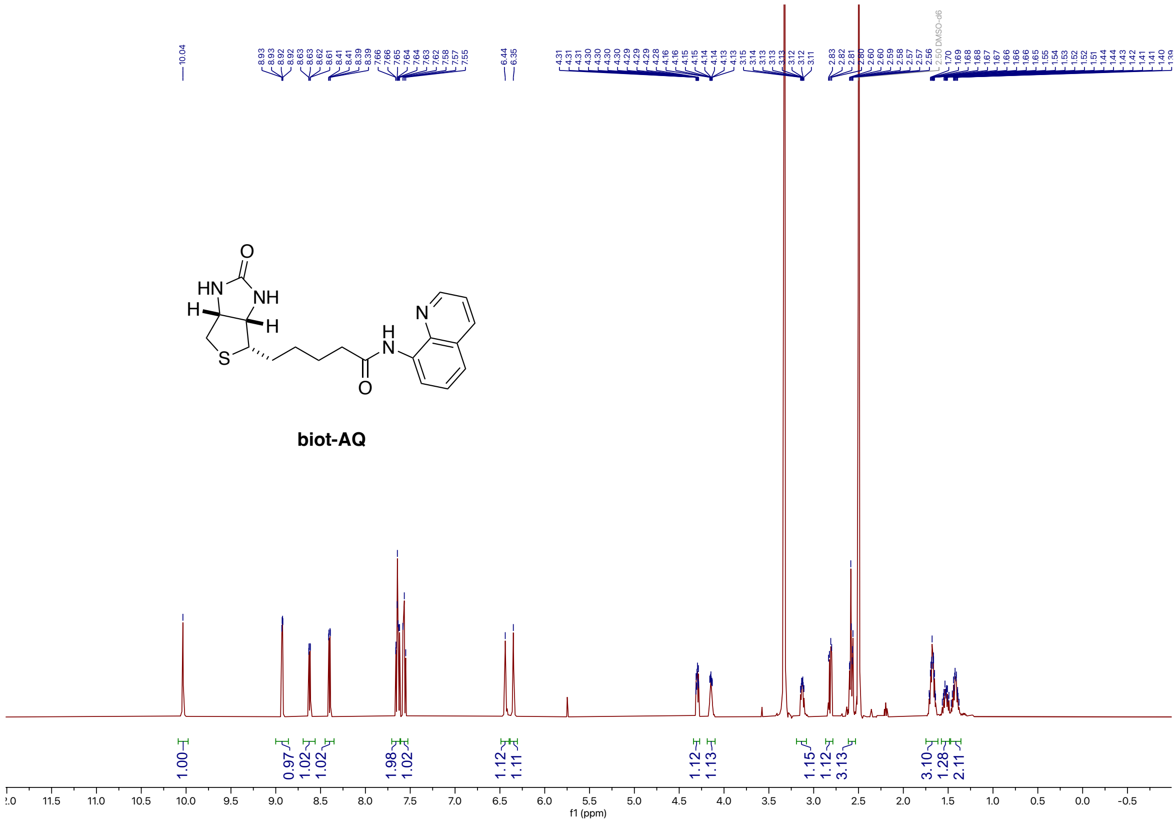

Figure S19. ^{1}H NMR of ligand **biot-AQ**.

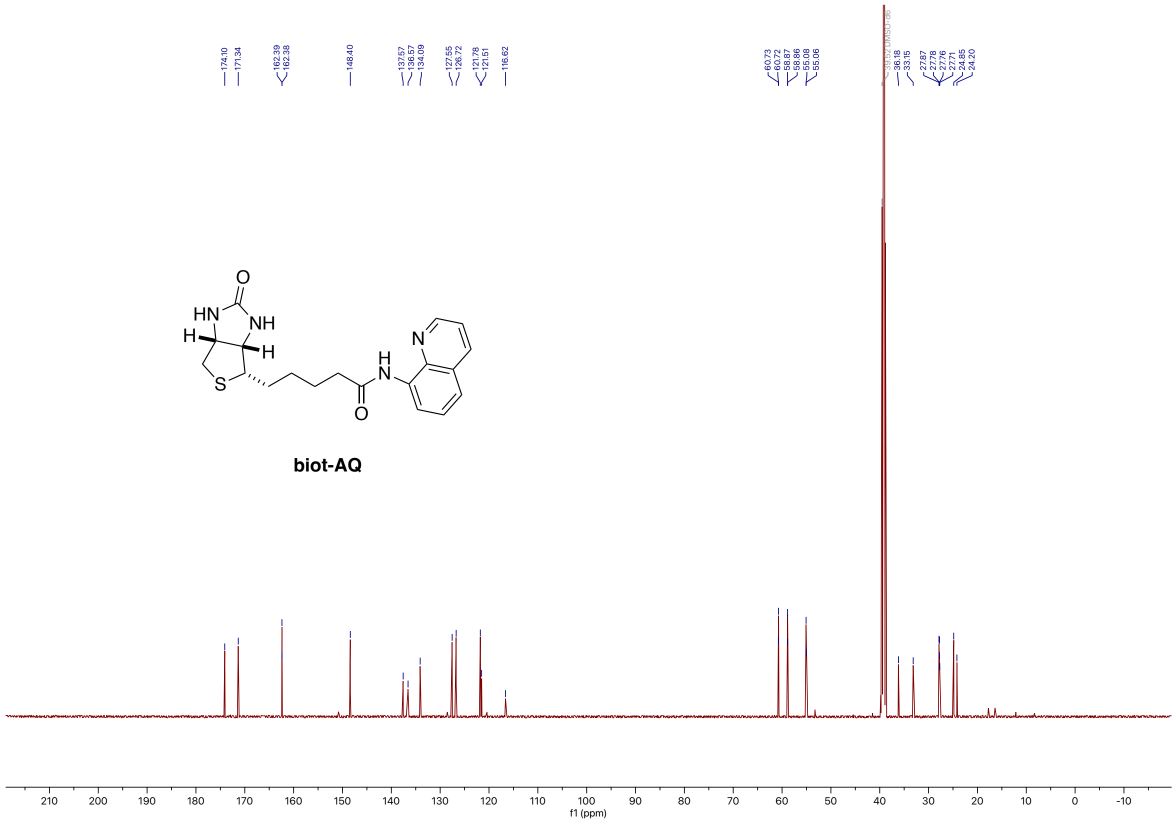

Figure S20. ^{13}C NMR of ligand **biot-AQ**.

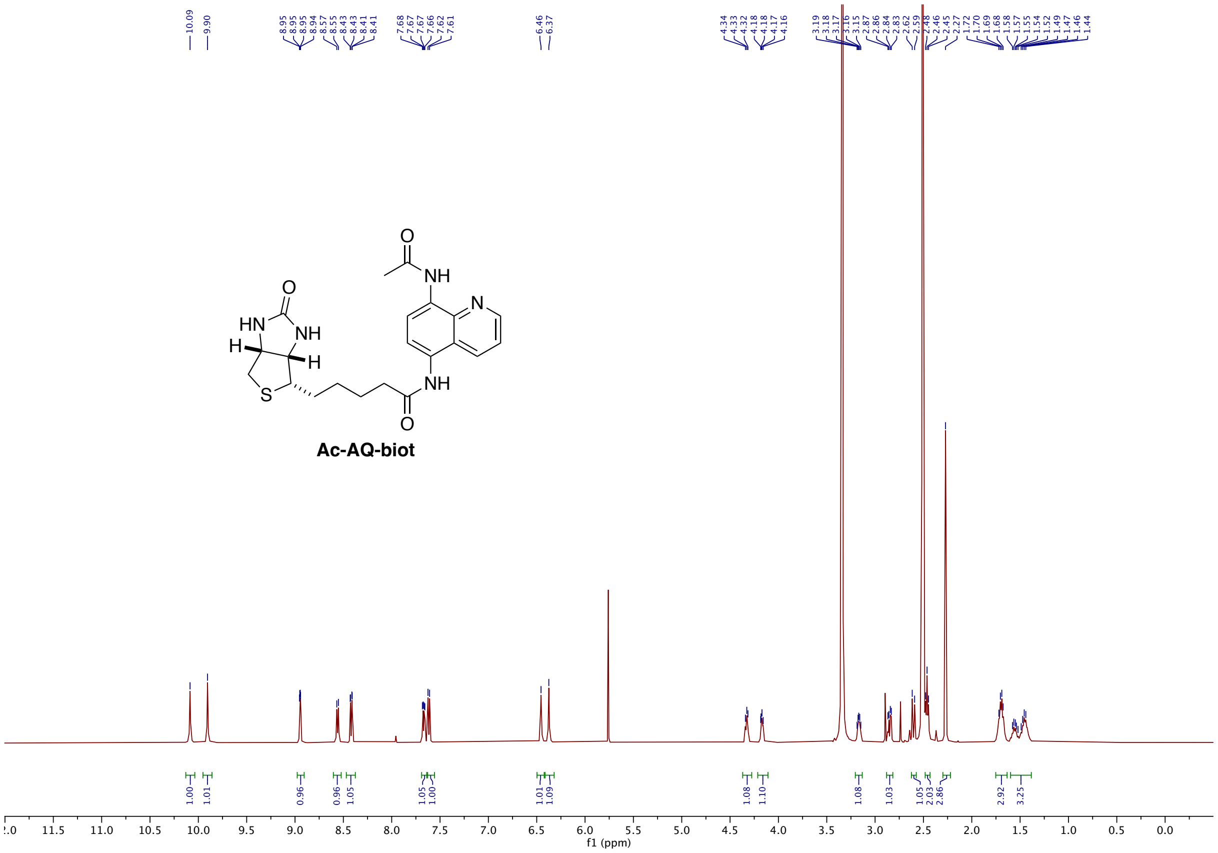

Figure S21. ^{1}H NMR of ligand **Ac-AQ-biot**.

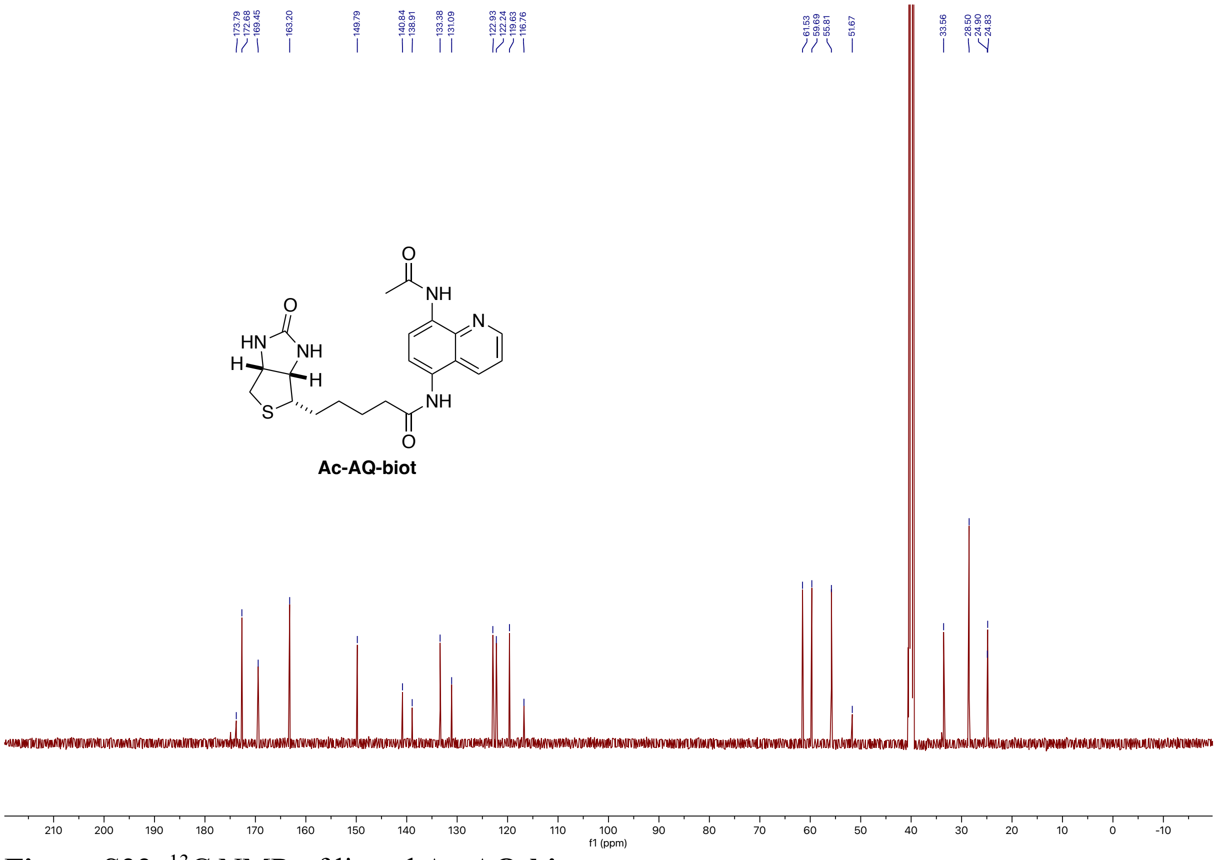

Figure S22. ^{13}C NMR of ligand **Ac-AQ-biot**.

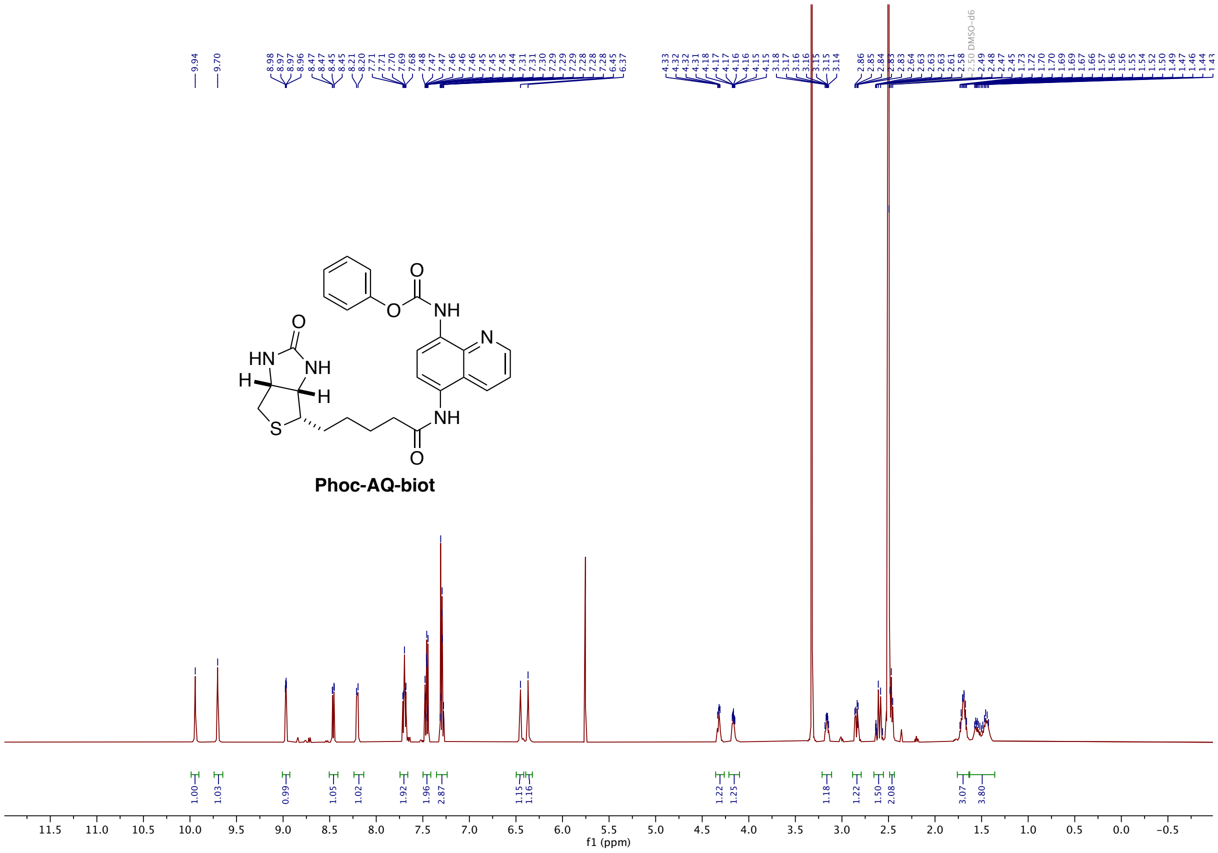

Figure S23. ^{1}H NMR of ligand **Phoc-AQ-biot**.

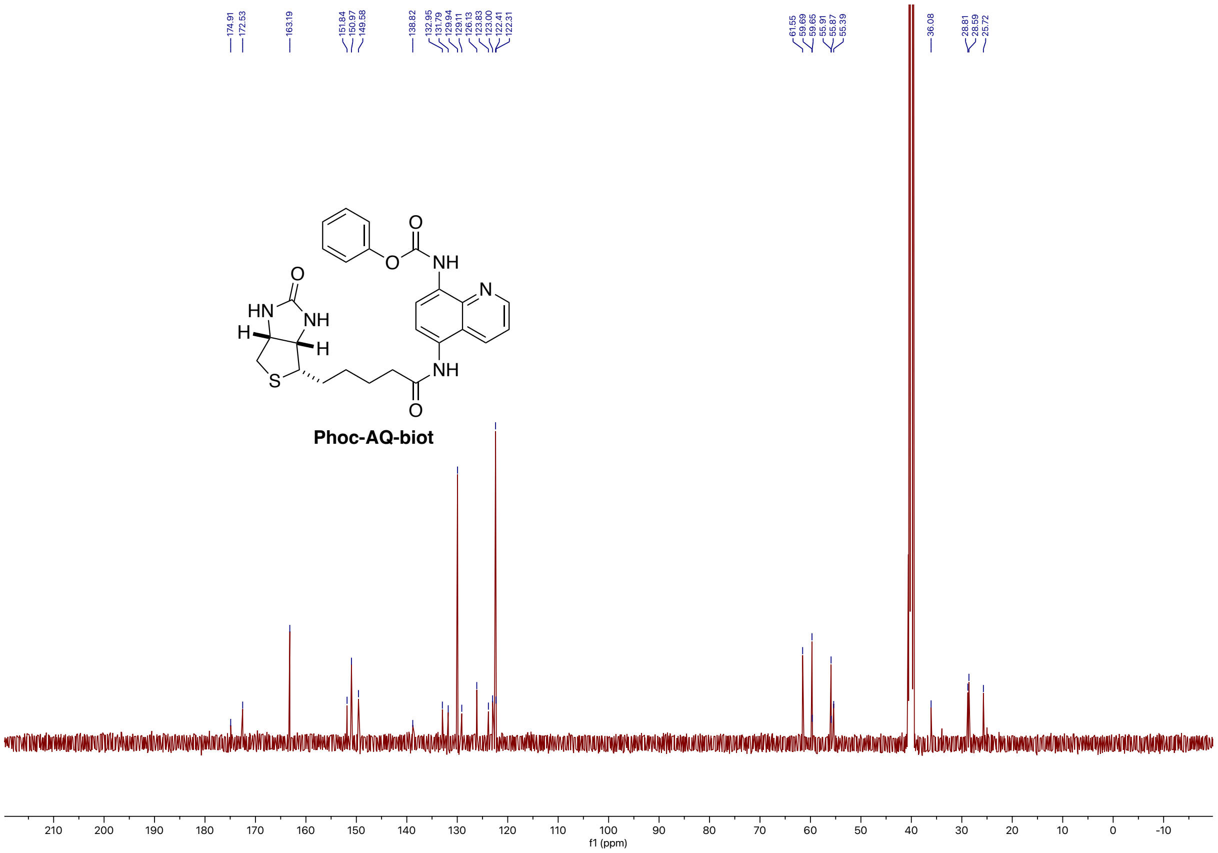

Figure S24. ^{13}C NMR of ligand **Phoc-AQ-biot**.

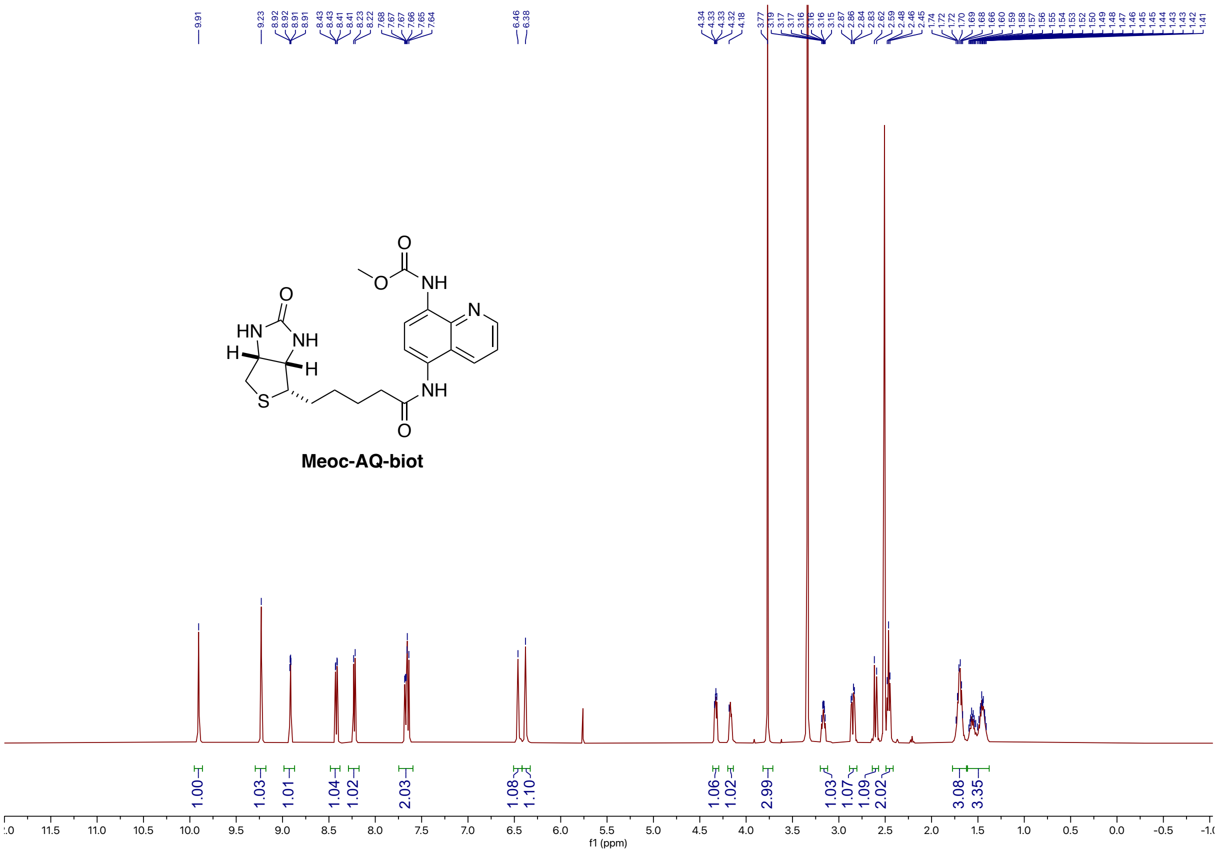

Figure S25. ^{1}H NMR of ligand **Meoc-AQ-biot**.

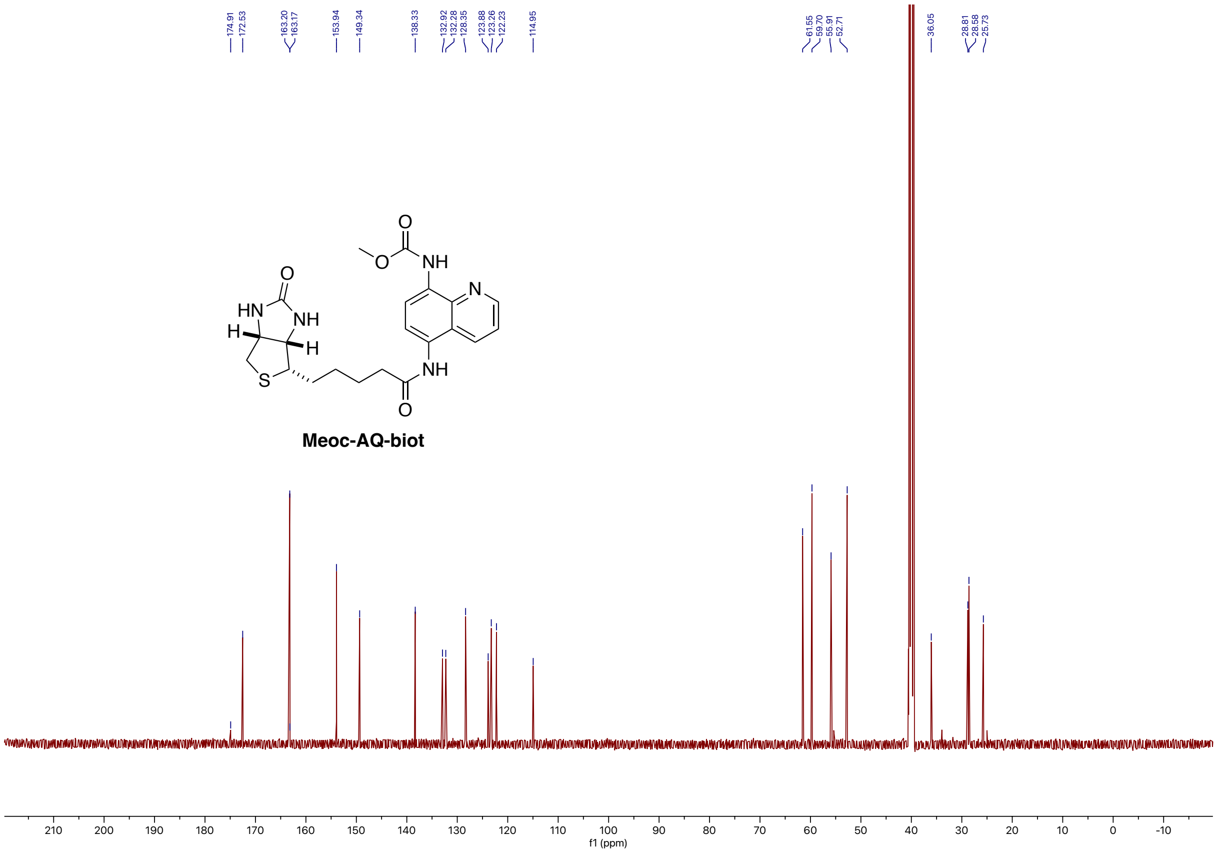

Figure S26. ^{13}C NMR of ligand **Meoc-AQ-biot**.

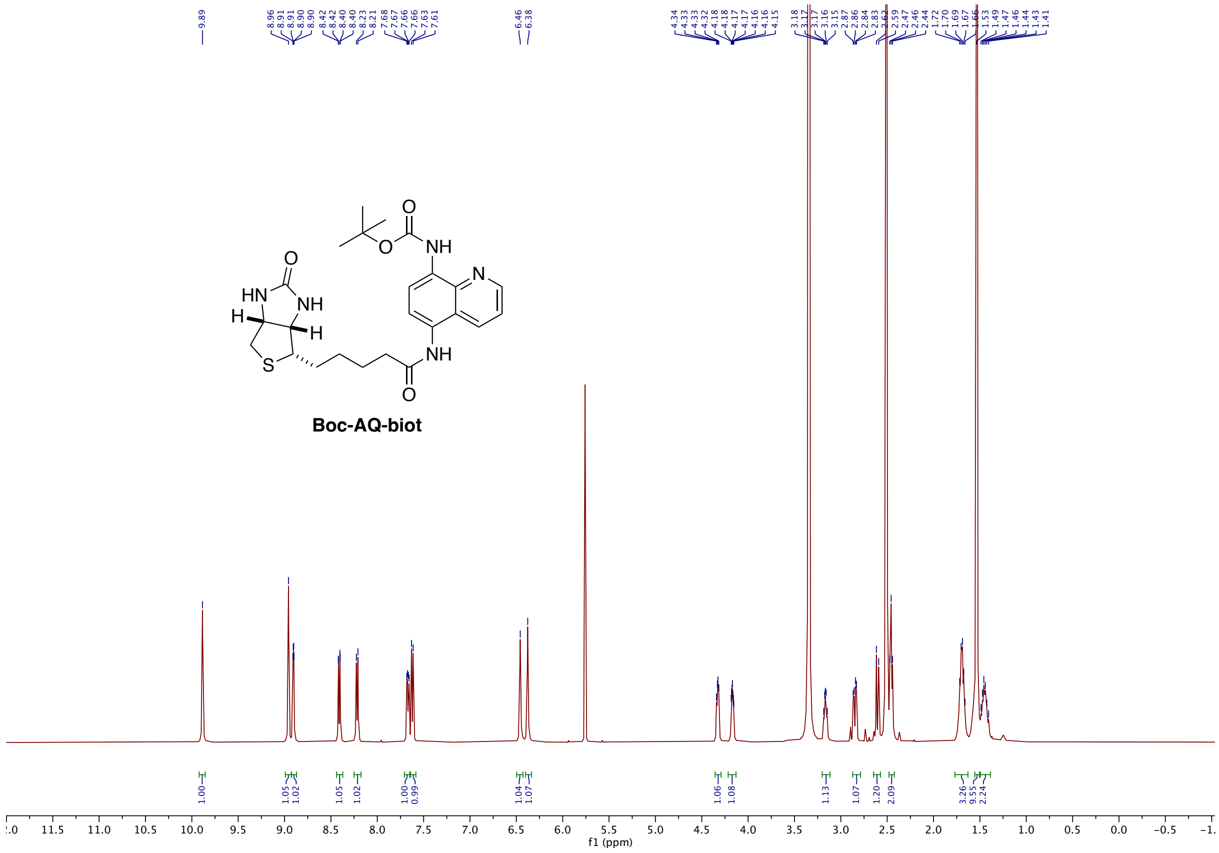

Figure S27. ^{1}H NMR of ligand **Boc-AQ-biot**.

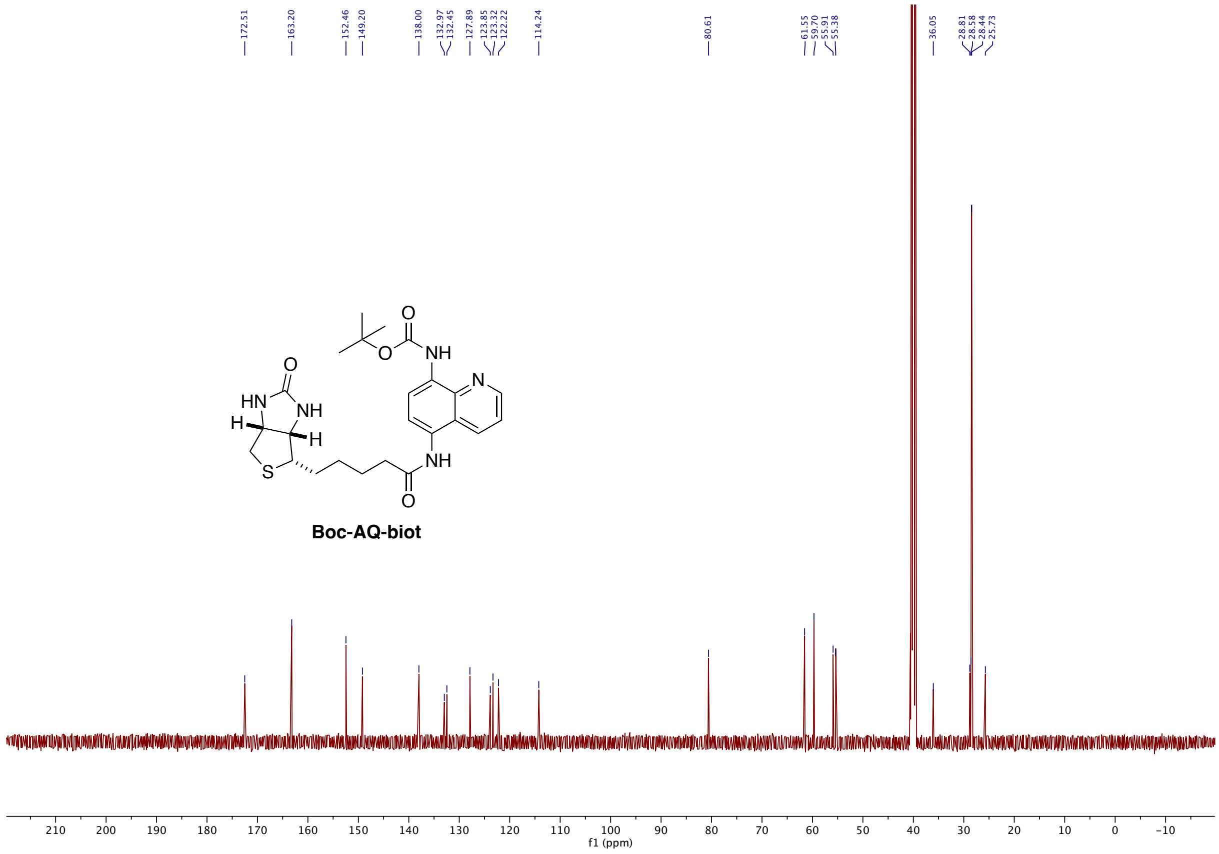

Figure S28. ^{13}C NMR of ligand **Boc-AQ-biot**.

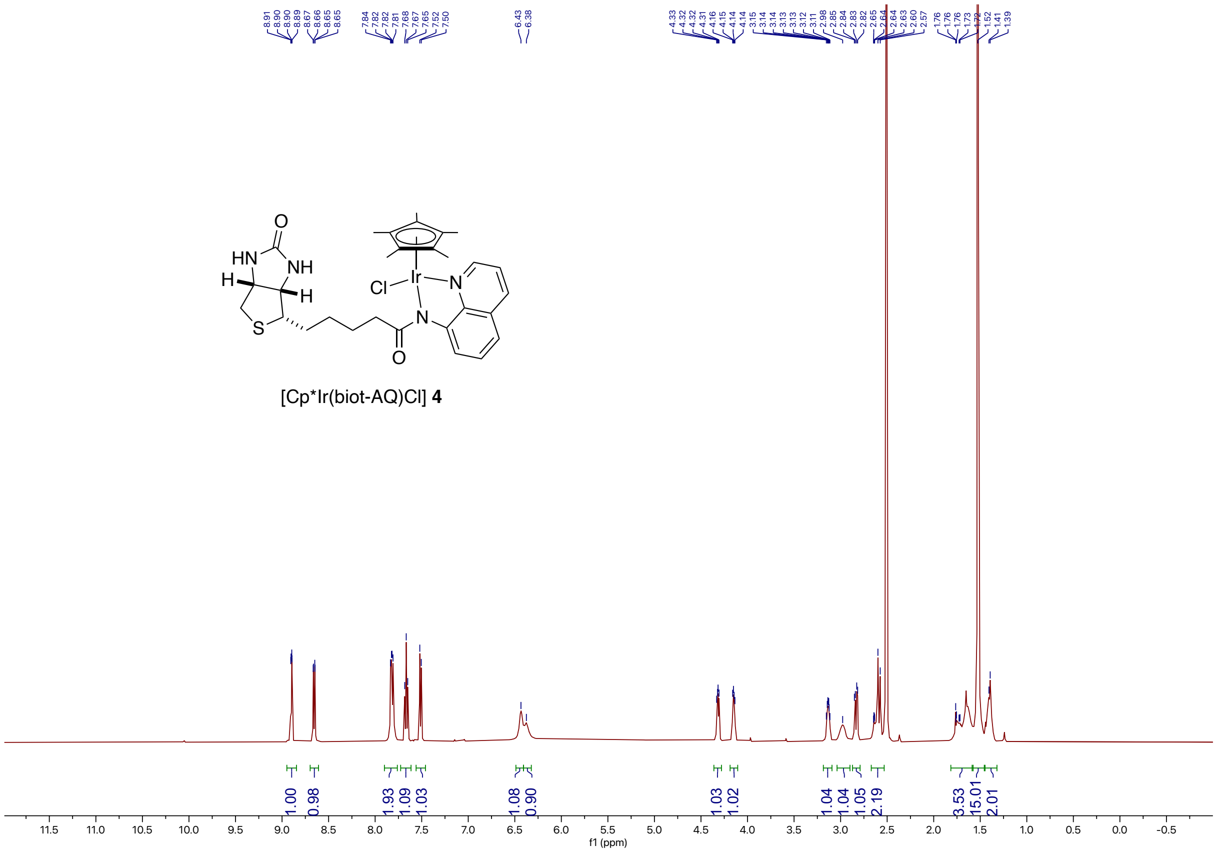

Figure S29. ^{1}H NMR of cofactor [Cp*Ir(biot-AQ)Cl] **4**.

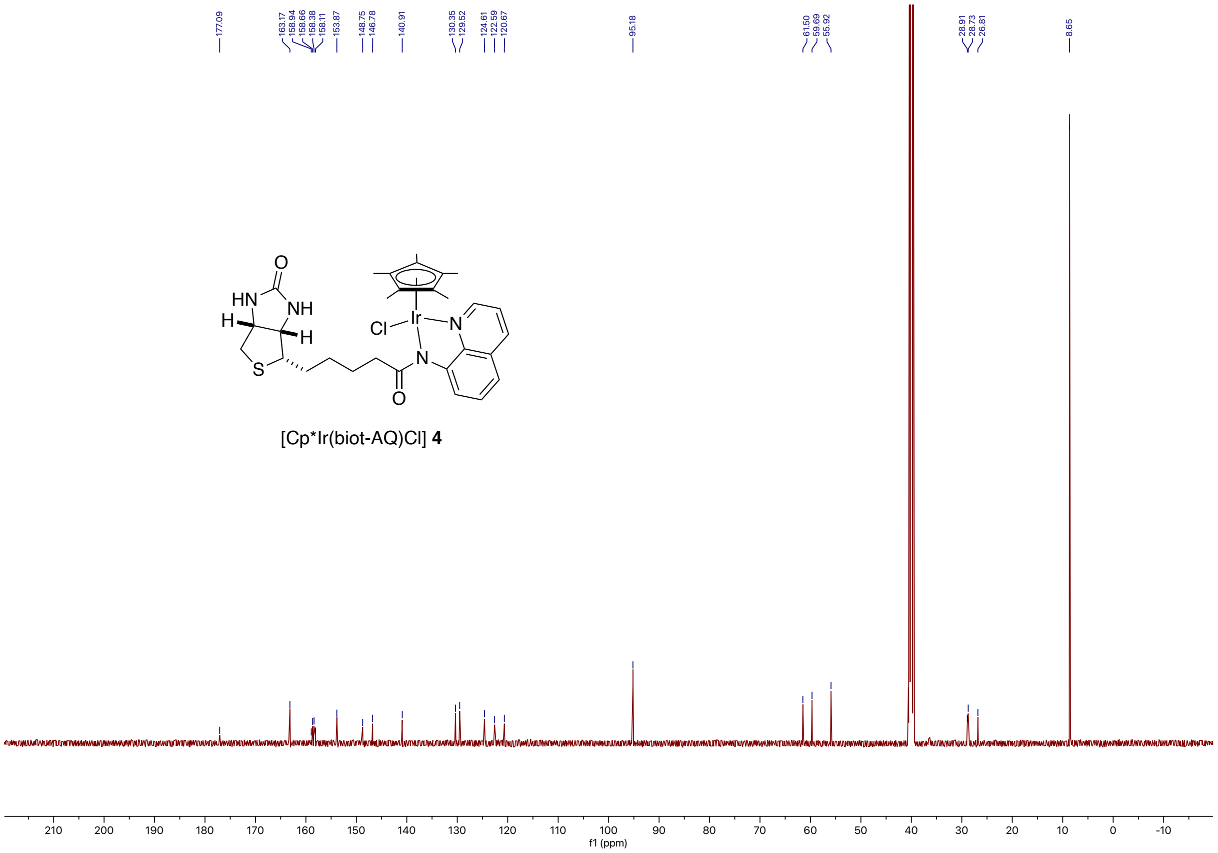

Figure S30. ^{13}C NMR of cofactor [Cp*Ir(biot-AQ)Cl] **4**.

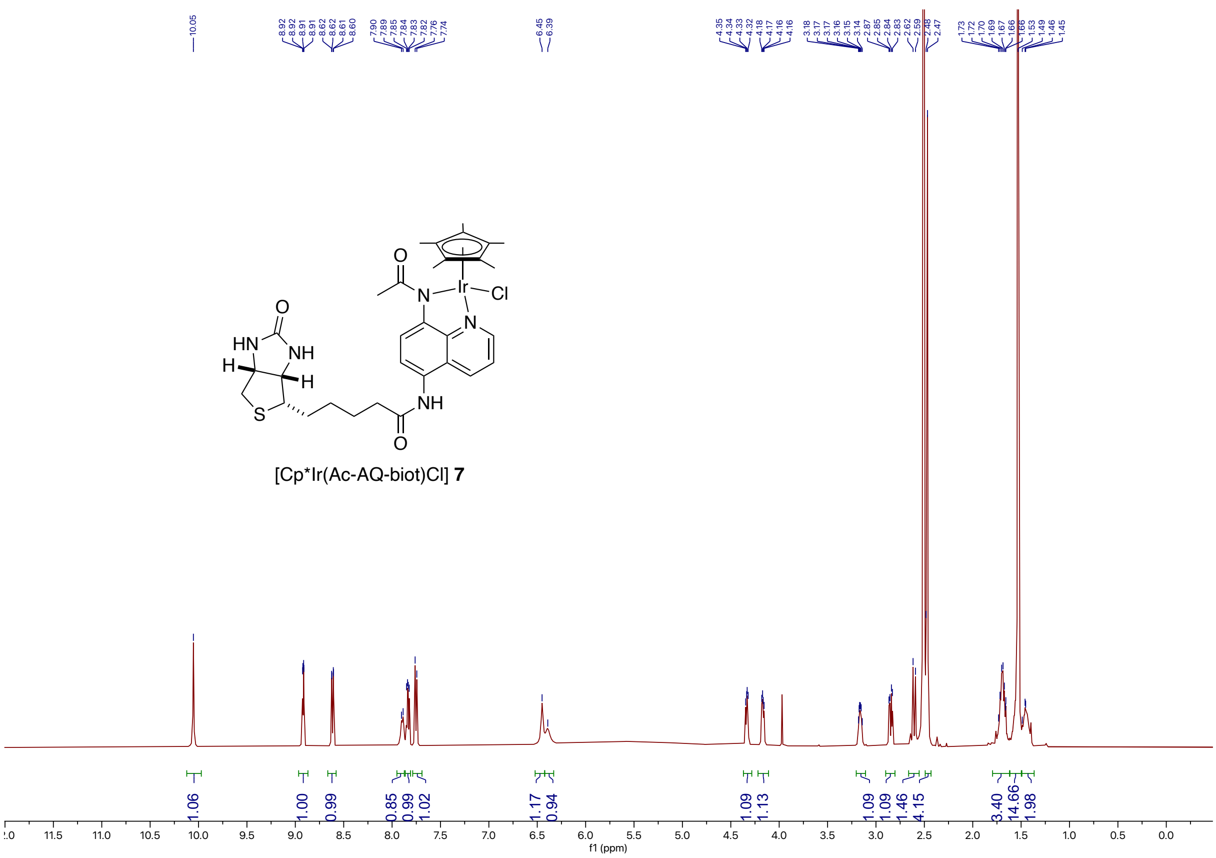

Figure S31. ^{1}H NMR of cofactor [Cp*Ir(Ac-AQ-biot)Cl] **7**.

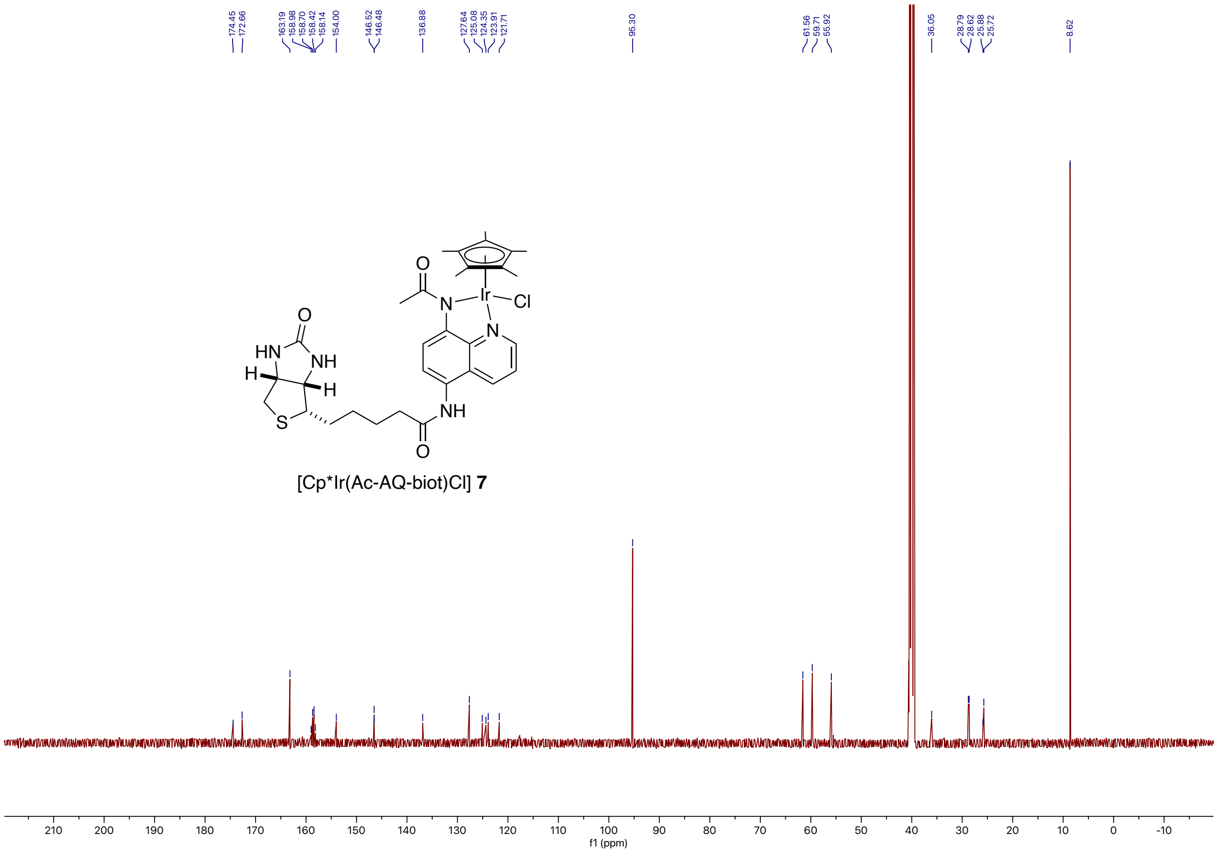

Figure S32. ^{13}C NMR of cofactor [Cp*Ir(Ac-AQ-biot)Cl] **7**.

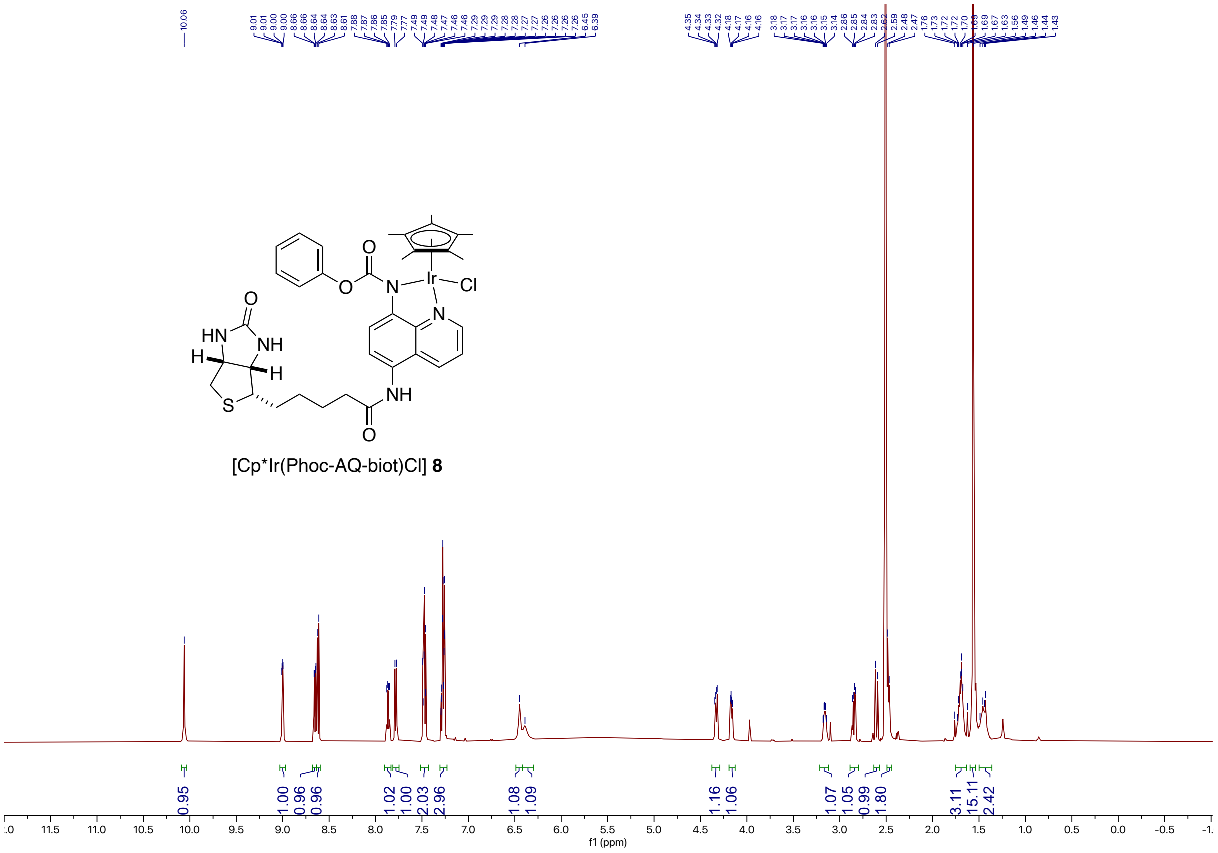

Figure S33. ^{1}H NMR of cofactor [Cp*Ir(Phoc-AQ-biot)Cl] **8**.

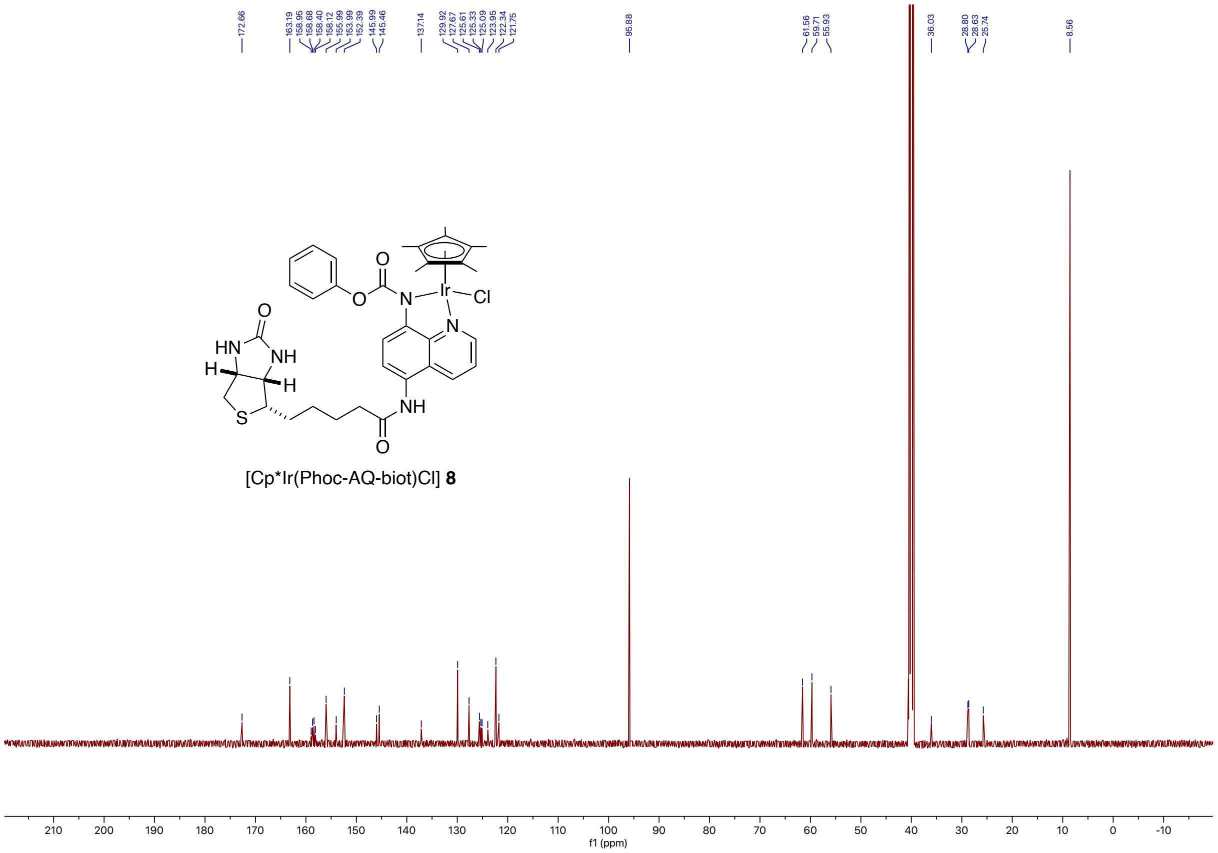

Figure S34. ^{13}C NMR of cofactor [Cp*Ir(Phoc-AQ-biot)Cl] **8**.

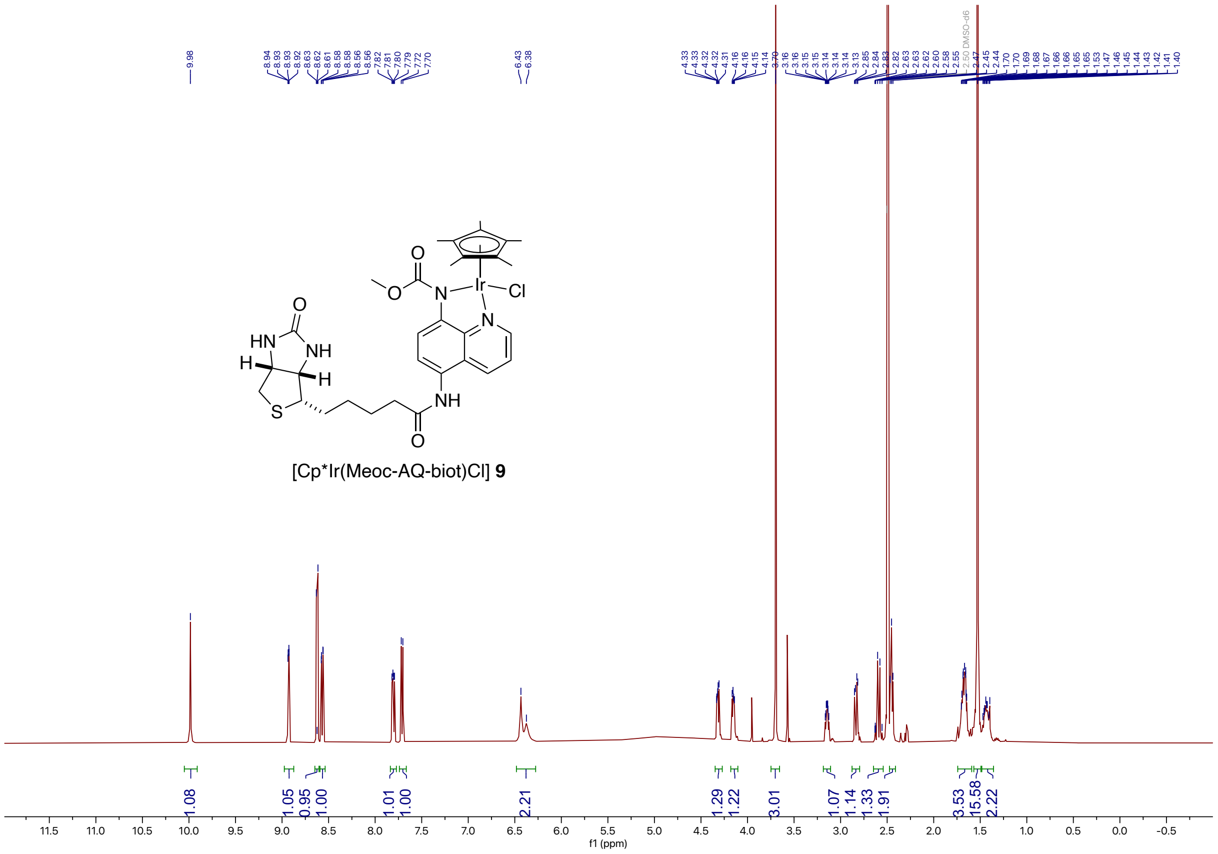

Figure S35. ^{1}H NMR of cofactor [Cp*Ir(Meoc-AQ-biot)Cl] **9**.

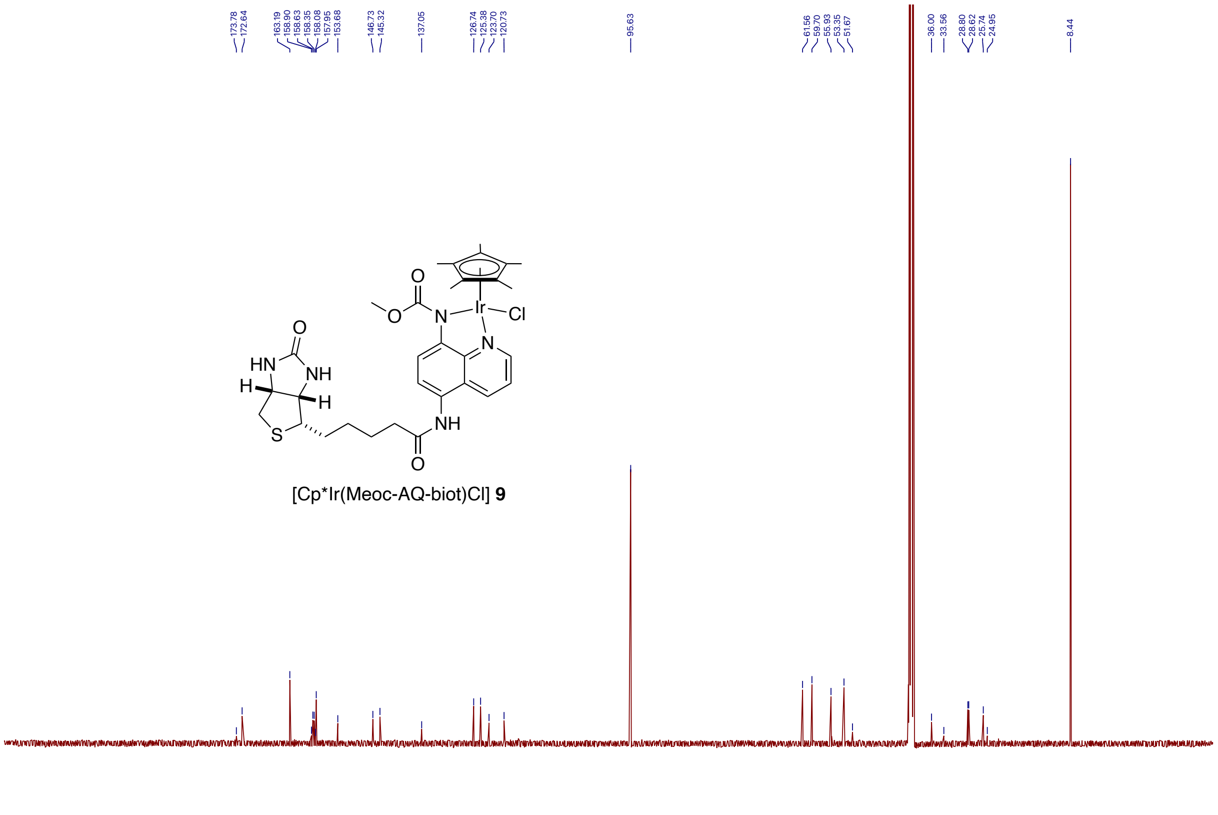

Figure S36. ^{13}C NMR of cofactor [Cp*Ir(Meoc-AQ-biot)Cl] **9**.

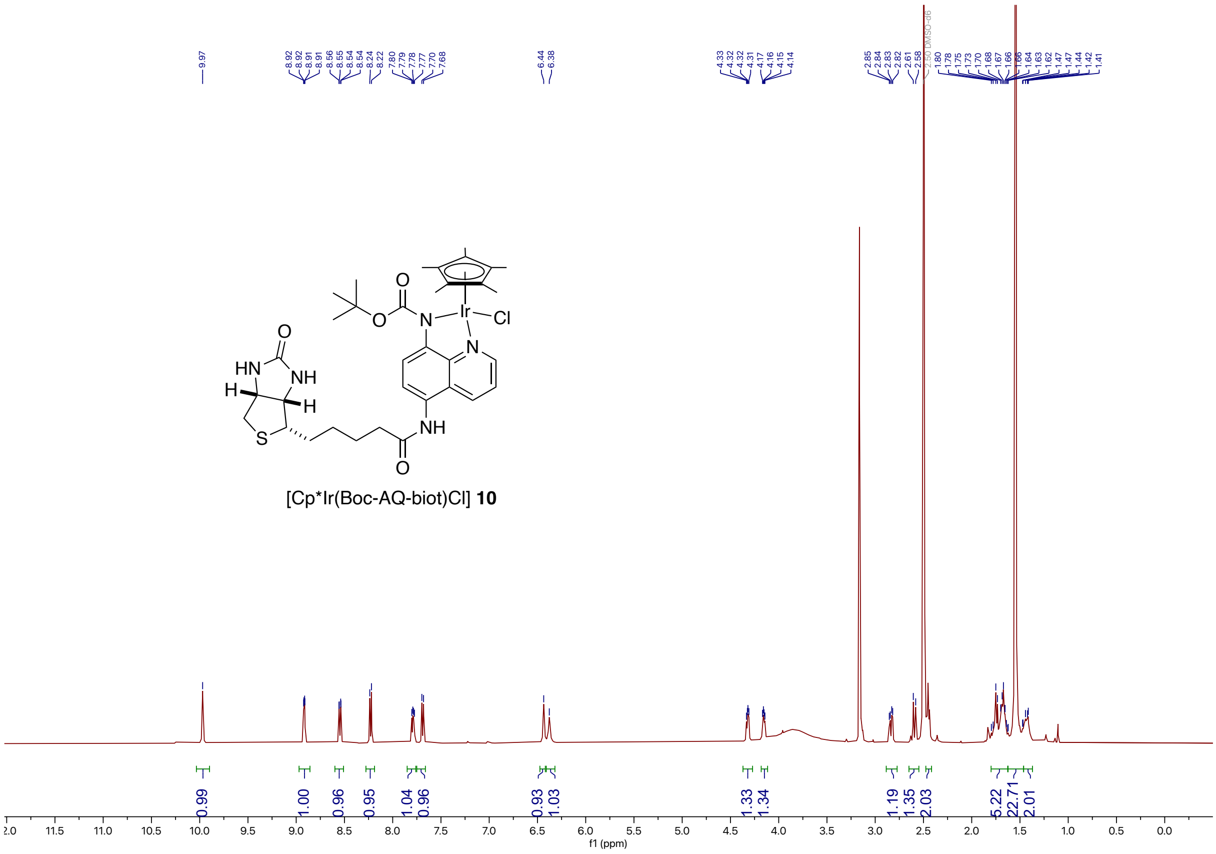

Figure S37. ^{1}H NMR of cofactor [Cp*Ir(Boc-AQ-biot)Cl] **10**.

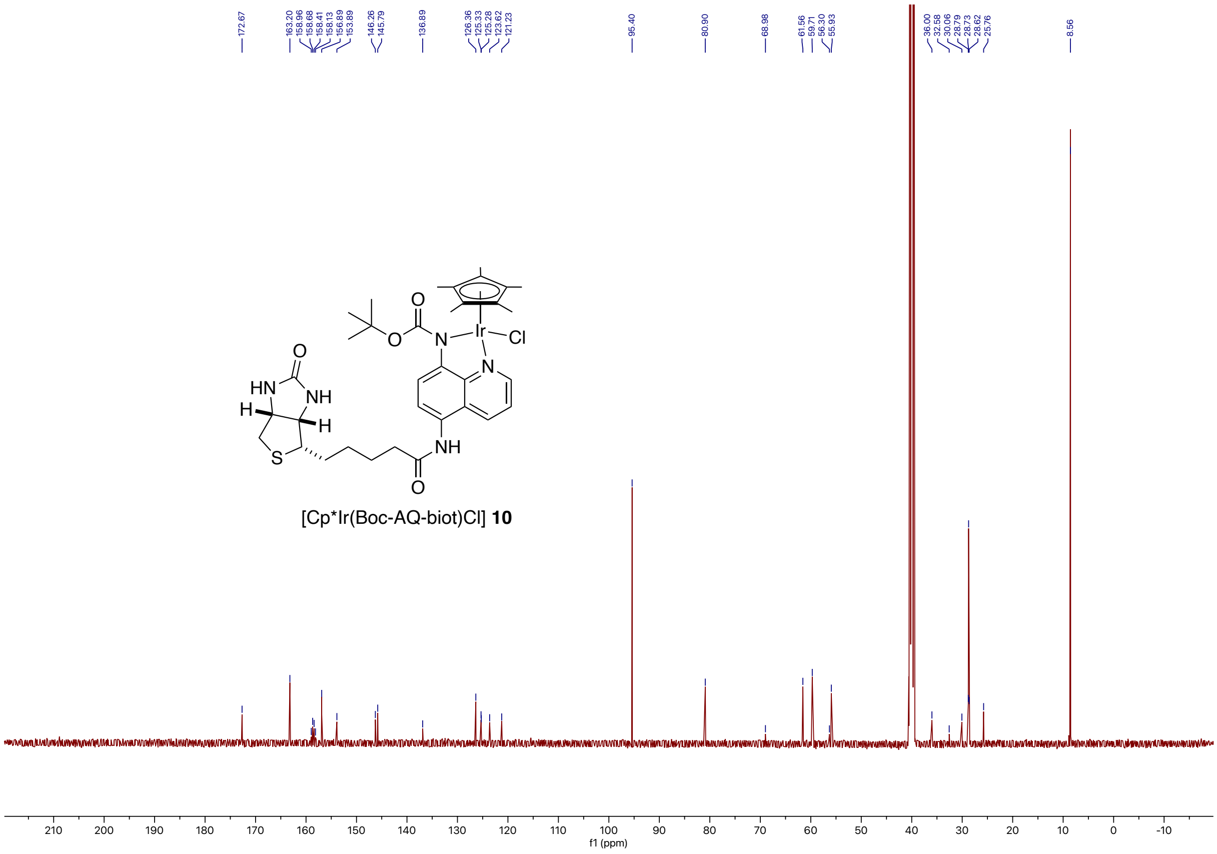

Figure S38. ^{13}C NMR of cofactor [Cp*Ir(Boc-AQ-biot)Cl] **10**.

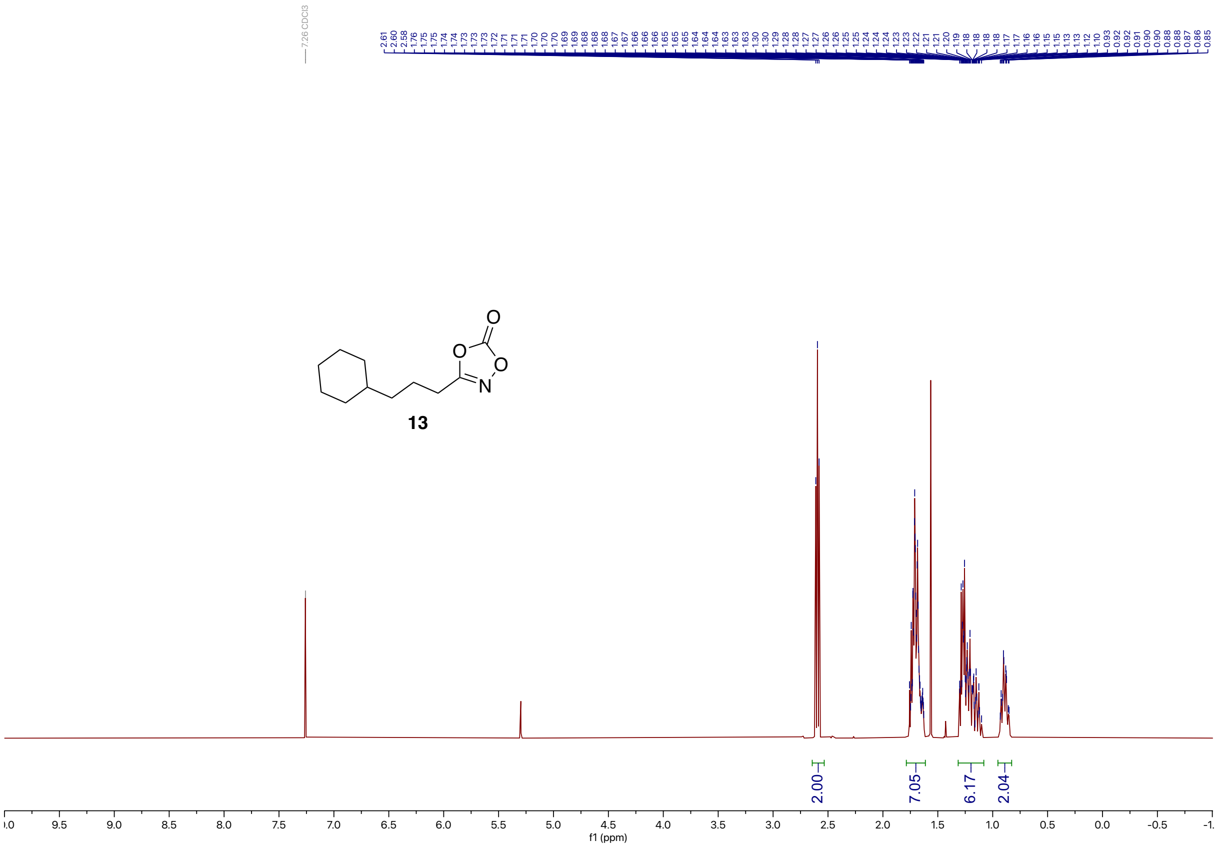

Figure S39. ^{1}H NMR of substrate **13**.

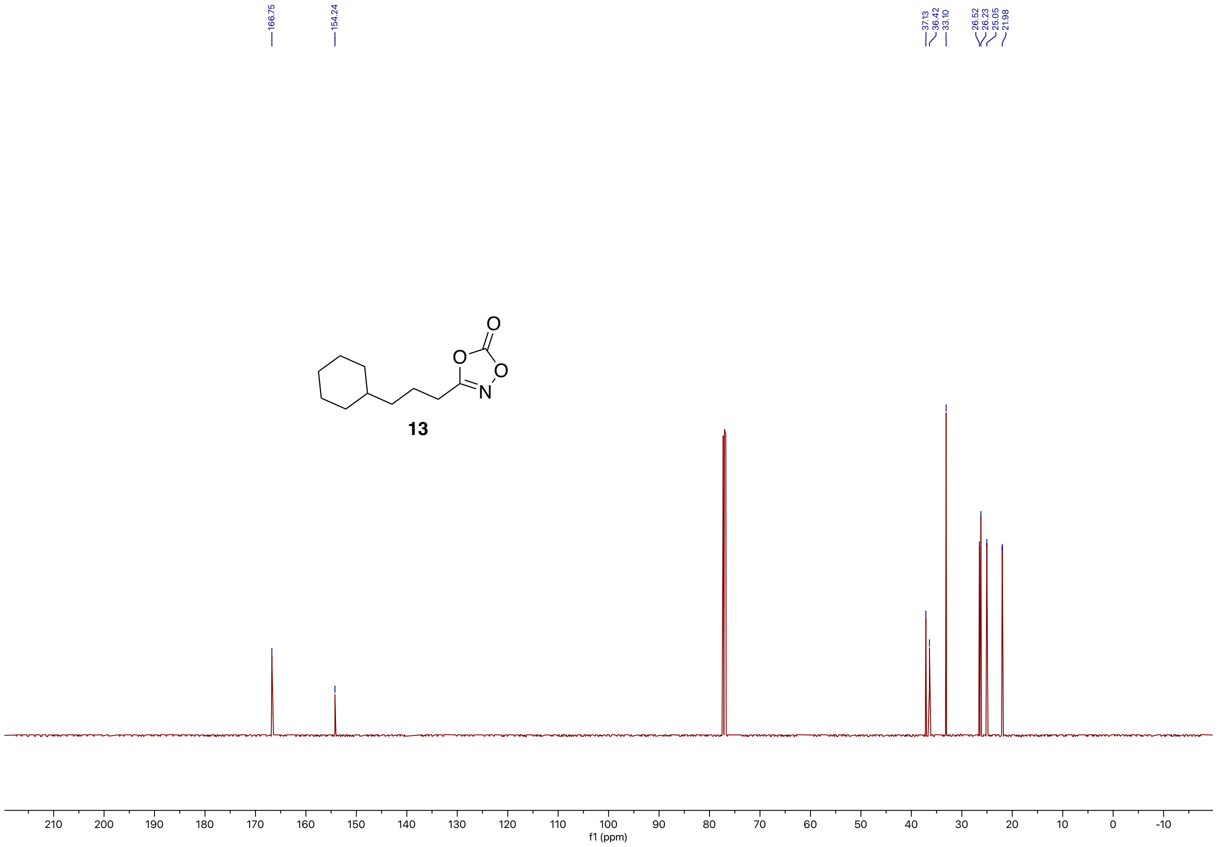

Figure S40. ^{13}C NMR of substrate **13**.

11. Coordinates for calculation

Ir$_{(S)}$-*pro-S* without Sav E(B3LYP) = -2026.462824

S	-9.226	5.594	-8.320
O	-10.251	10.072	-8.088
N	-9.098	8.562	-9.594
N	-8.397	8.661	-7.330
C	-10.684	3.487	-13.624
C	-10.528	4.832	-12.941
C	-9.504	4.771	-11.800
C	-9.467	6.109	-11.080
C	-8.436	6.180	-9.916
C	-8.039	7.607	-9.496
C	-7.945	6.200	-7.371
C	-7.540	7.645	-7.918
C	-9.342	9.192	-8.333
H	-9.926	8.045	-9.817
H	-8.950	8.200	-6.635
H	-11.365	3.595	-14.477
H	-11.154	2.785	-12.932
H	-11.520	5.138	-12.532
H	-10.218	5.606	-13.680
H	-9.790	3.972	-11.080
H	-8.496	4.572	-12.221
H	-10.504	6.283	-10.712
H	-9.209	6.895	-11.824
H	-7.611	5.618	-10.300
H	-7.260	7.871	-10.181
H	-8.328	6.338	-6.382
H	-7.105	5.558	-7.535
H	-6.506	7.850	-7.733
C	-1.450	0.277	-16.553
N	-4.641	0.920	-15.508
O	-8.580	3.579	-14.824
C	-0.898	1.584	-16.062
N	-9.085	1.653	-13.692
C	-1.839	-0.878	-15.751
O	-4.784	-2.575	-18.619
C	-1.749	-0.974	-14.257
O	-6.676	-3.002	-17.446
C	-2.218	-1.931	-16.651
N	-5.425	-1.136	-16.960
C	-2.649	-3.307	-16.243
C	-2.033	-1.458	-18.017
C	-2.090	-2.261	-19.283
C	-1.537	-0.112	-17.936
C	-1.181	0.745	-19.108
C	-6.382	-0.624	-16.077
C	-5.944	0.519	-15.339
C	-7.695	-1.042	-15.857
C	-6.792	1.221	-14.442
C	-8.556	-0.298	-15.038
C	-8.156	0.839	-14.373
C	-6.225	2.238	-13.641
C	-4.131	1.884	-14.746
C	-4.892	2.545	-13.768
C	-9.359	2.946	-14.130
C	-5.589	-2.284	-17.744
C	-6.982	-4.311	-18.097
C	-7.161	-4.130	-19.606
C	-5.896	-5.332	-17.755
C	-8.306	-4.706	-17.448
Ir	-3.606	-0.146	-16.969
H	-0.909	1.639	-14.969
H	-1.482	2.410	-16.469
H	0.137	1.715	-16.384
H	-9.858	1.166	-13.256
H	-2.131	-1.931	-13.893
H	-2.310	-0.182	-13.753
H	-0.714	-0.897	-13.917
H	-3.294	-3.761	-16.996
H	-3.203	-3.296	-15.298
H	-1.784	-3.962	-16.094
H	-2.457	-3.270	-19.101
H	-2.772	-1.816	-20.011
H	-1.097	-2.322	-19.738
H	-1.838	0.534	-19.956
H	-1.262	1.805	-18.868
H	-0.154	0.537	-19.427
H	-8.082	-1.928	-16.334
H	-9.582	-0.626	-14.944
H	-6.849	2.739	-12.915
H	-3.098	2.142	-14.912
H	-4.420	3.284	-13.135
H	-7.518	-5.076	-20.030
H	-7.915	-3.366	-19.815
H	-6.225	-3.864	-20.094
H	-6.225	-6.321	-18.094
H	-5.753	-5.397	-16.672
H	-4.951	-5.101	-18.243
H	-8.637	-5.670	-17.848
H	-9.082	-3.963	-17.648
H	-8.205	-4.824	-16.365
N	-4.126	1.266	-18.250
C	-4.279	2.616	-18.067
O	-3.439	3.263	-17.426
C	-5.483	3.302	-18.705
H	-5.227	4.344	-18.898
H	-6.261	3.306	-17.931
C	-5.992	2.602	-19.967
H	-5.285	2.754	-20.789
H	-6.942	3.052	-20.290
C	-6.176	1.119	-19.699
H	-5.168	0.869	-19.056
H	-6.978	0.929	-18.979
C	-6.193	0.124	-20.852
C	-7.488	0.291	-21.694
C	-4.954	0.180	-21.768
H	-6.226	-0.882	-20.410
C	-7.561	-0.764	-22.810
H	-7.506	1.295	-22.138
H	-8.370	0.214	-21.050
C	-5.030	-0.885	-22.873
H	-4.881	1.169	-22.239
H	-4.046	0.044	-21.169
C	-6.313	-0.741	-23.704
H	-8.464	-0.599	-23.411
H	-7.667	-1.761	-22.359
H	-4.150	-0.816	-23.523
H	-5.005	-1.885	-22.418
H	-6.373	-1.540	-24.452
H	-6.284	0.205	-24.262

Ir$_{(S)}$-*pro-R* without Sav

E(B3LYP) = -2026.449069

S	38.208	7.982	7.286
O	42.350	7.884	5.288
N	40.384	9.303	5.294
N	40.127	7.009	4.747
C	37.110	13.197	9.657
C	38.129	12.593	8.708
C	37.542	11.401	7.948
C	38.613	10.766	7.074
C	38.099	9.547	6.260
C	38.983	9.135	5.069
C	37.924	6.982	5.934
C	38.772	7.535	4.699
C	41.085	8.067	5.124
H	40.488	9.557	6.256
H	40.144	6.334	5.485
H	37.561	14.050	10.179
H	36.849	12.464	10.423
H	39.010	12.253	9.302
H	38.486	13.361	7.983
H	37.167	10.644	8.673
H	36.718	11.755	7.294
H	39.444	10.485	7.760
H	38.965	11.533	6.351
H	37.128	9.879	5.958
H	38.671	9.785	4.278
H	38.316	6.016	6.173
H	36.891	7.092	5.677
H	38.295	7.302	3.770
C	28.887	15.917	4.304
N	31.399	15.051	6.436
O	35.898	14.375	7.923
C	29.576	15.435	3.060
N	34.684	13.258	9.520
C	28.313	15.098	5.365
O	28.504	17.962	8.680
C	28.288	13.600	5.396
O	29.416	16.865	10.442
C	27.650	15.977	6.292
N	30.197	16.468	8.309
C	26.863	15.527	7.485
C	27.790	17.341	5.808
C	27.109	18.574	6.324
C	28.514	17.279	4.565
C	28.828	18.451	3.692
C	31.203	15.602	8.759
C	31.867	14.884	7.716
C	31.654	15.363	10.058
C	32.948	13.998	7.964
C	32.773	14.551	10.295
C	33.463	13.922	9.282
C	33.421	13.198	6.898
C	31.863	14.290	5.448
C	32.855	13.319	5.653
C	35.873	13.691	8.932
C	29.307	17.160	9.140
C	28.492	17.420	11.479
C	28.569	18.947	11.514
C	27.067	16.922	11.235
C	29.052	16.842	12.777
Ir	29.872	16.452	6.255
H	29.691	14.347	3.062
H	30.560	15.896	2.969
H	28.995	15.697	2.172
H	34.781	12.837	10.435
H	27.785	13.228	6.293
H	29.289	13.160	5.372
H	27.742	13.200	4.538
H	26.765	16.323	8.222
H	27.334	14.674	7.984
H	25.859	15.202	7.188
H	26.581	18.379	7.256
H	27.829	19.367	6.542
H	26.396	18.953	5.586
H	28.920	18.154	2.646
H	28.052	19.216	3.776
H	29.777	18.905	4.000
H	31.169	15.821	10.904
H	33.113	14.434	11.316
H	34.201	12.475	7.092
H	31.447	14.458	4.469
H	33.162	12.686	4.829
H	27.955	19.304	12.348
H	29.596	19.272	11.695
H	28.199	19.393	10.593
H	26.455	17.174	12.108
H	27.043	15.834	11.132
H	26.619	17.388	10.358
H	28.458	17.200	13.623
H	30.090	17.150	12.929
H	29.008	15.750	12.794
N	31.253	17.810	5.654
C	32.405	17.587	4.939
O	32.479	16.721	4.058
C	33.566	18.538	5.205
H	34.315	18.421	4.422
H	34.042	18.261	6.154
C	33.049	19.977	5.303

H	32.614	20.290	4.350
H	33.890	20.653	5.502
C	32.019	20.091	6.410
H	31.124	20.661	6.133
H	31.442	18.870	6.308
C	32.456	20.385	7.828
C	33.202	21.759	7.857
C	31.255	20.435	8.794
H	33.174	19.625	8.164
C	33.612	22.155	9.283
H	32.541	22.532	7.440
H	34.092	21.723	7.222
C	31.640	20.864	10.218
H	30.528	21.157	8.395
H	30.745	19.468	8.813
C	32.412	22.190	10.235
H	34.100	23.137	9.251
H	34.367	21.450	9.652
H	30.731	20.962	10.822
H	32.238	20.078	10.693
H	32.739	22.427	11.253
H	31.737	23.003	9.931

Ir$_{(R)}$-*pro-S* without Sav

E(B3LYP) = -2026.460082

S	38.208	7.982	7.286
O	42.350	7.884	5.288
N	40.384	9.303	5.294
N	40.127	7.009	4.747
C	37.296	13.282	9.698
C	38.225	12.640	8.679
C	37.568	11.462	7.940
C	38.616	10.775	7.066
C	38.099	9.547	6.260
C	38.983	9.135	5.069
C	37.924	6.982	5.934
C	38.772	7.535	4.699
C	41.085	8.067	5.124
H	40.488	9.557	6.256
H	40.144	6.334	5.485
H	37.896	13.668	10.533
H	36.633	12.526	10.137
H	39.120	12.258	9.224
H	38.591	13.397	7.947
H	37.198	10.721	8.681
H	36.722	11.826	7.317
H	39.433	10.472	7.761
H	39.003	11.524	6.342
H	37.128	9.879	5.958
H	38.671	9.785	4.278
H	38.316	6.016	6.173
H	36.891	7.092	5.677
H	38.295	7.302	3.770
C	30.007	18.525	7.296
N	31.665	17.042	9.814
O	35.829	14.325	8.054
C	30.876	17.867	6.264
N	36.299	15.473	9.979
C	28.906	17.890	8.022
O	31.506	21.652	9.998
C	28.476	16.461	7.905
O	33.687	21.410	9.402
C	28.241	18.904	8.778
N	32.359	19.557	9.635
C	27.008	18.754	9.610
C	28.911	20.173	8.549
C	28.464	21.499	9.081
C	29.955	19.941	7.588
C	30.792	20.996	6.940
C	33.425	18.647	9.677
C	33.016	17.284	9.794
C	34.799	18.890	9.663
C	33.952	16.219	9.938
C	35.729	17.833	9.729
C	35.345	16.514	9.850
C	33.440	14.932	10.221
C	31.209	15.819	10.085
C	32.082	14.741	10.313
C	36.419	14.387	9.119
C	32.466	20.944	9.717
C	34.035	22.857	9.505
C	33.192	23.675	8.524
C	33.869	23.334	10.947
C	35.509	22.868	9.094
Ir	30.465	18.730	9.451
H	31.058	16.820	6.517
H	31.842	18.371	6.189
H	30.392	17.905	5.283
H	36.922	15.520	10.774
H	28.086	16.111	8.864
H	29.302	15.809	7.615
H	27.689	16.365	7.151
H	27.107	19.301	10.553
H	26.799	17.716	9.858
H	26.154	19.186	9.077
H	27.907	21.377	10.014
H	29.315	22.146	9.289
H	27.800	21.998	8.365
H	30.852	21.887	7.566
H	31.806	20.649	6.734
H	30.331	21.282	5.987
H	35.167	19.900	9.592
H	36.787	18.071	9.689
H	34.123	14.112	10.409
H	30.136	15.717	10.177
H	31.673	13.777	10.586
H	33.564	24.704	8.505
H	33.271	23.270	7.511
H	32.145	23.696	8.827
H	34.216	24.369	11.030
H	34.472	22.724	11.626
H	32.826	23.296	11.264
H	35.887	23.894	9.114
H	35.637	22.472	8.084
H	36.111	22.273	9.787
N	29.754	18.532	11.300
C	29.047	17.515	11.856
O	28.553	16.633	11.129
C	28.792	17.516	13.360
H	28.764	16.469	13.681
H	27.775	17.902	13.520
C	29.814	18.341	14.142
H	30.774	17.818	14.158
H	29.478	18.458	15.181
C	29.963	19.691	13.473
H	30.108	19.337	12.271
H	28.998	20.208	13.394
C	31.080	20.651	13.823
C	30.757	21.309	15.200
C	32.495	20.048	13.802
H	31.053	21.459	13.077
C	31.843	22.311	15.622
H	30.664	20.531	15.967
H	29.781	21.808	15.148
C	33.545	21.091	14.210
H	32.560	19.197	14.491
H	32.712	19.655	12.801
C	33.241	21.680	15.594
H	31.610	22.690	16.623
H	31.819	23.176	14.946
H	34.545	20.644	14.191
H	33.552	21.903	13.473
H	33.998	22.423	15.867
H	33.301	20.885	16.351

Ir$_{(R)}$-*pro-R* without Sav

E(B3LYP) = -2026.456024

S	38.208	7.982	7.286
O	42.350	7.884	5.288
N	40.384	9.303	5.294
N	40.127	7.009	4.747
C	36.847	13.326	9.463
C	37.963	12.665	8.658
C	37.463	11.413	7.925
C	38.576	10.791	7.071
C	38.099	9.547	6.260
C	38.983	9.135	5.069
C	37.924	6.982	5.934
C	38.772	7.535	4.699
C	41.085	8.067	5.124
H	40.488	9.557	6.256
H	40.144	6.334	5.485
H	37.305	13.978	10.222
H	36.283	12.571	10.029
H	38.777	12.359	9.357
H	38.408	13.389	7.936
H	37.138	10.661	8.677
H	36.605	11.693	7.274
H	39.411	10.537	7.763
H	38.911	11.559	6.344
H	37.128	9.879	5.958
H	38.671	9.785	4.278
H	38.316	6.016	6.173
H	36.891	7.092	5.677
H	38.295	7.302	3.770
C	28.039	18.439	10.376
N	31.094	17.283	10.917
O	36.143	14.618	7.502
C	27.644	17.169	9.681
N	34.779	14.609	9.338
C	28.135	18.591	11.825
O	30.863	21.408	8.818
C	27.815	17.559	12.866
O	32.366	20.378	7.465
C	28.363	19.970	12.108
N	31.490	19.246	9.253
C	28.443	20.555	13.480
C	28.427	20.699	10.859
C	28.519	22.187	10.736
C	28.176	19.755	9.797
C	28.003	20.106	8.352
C	32.352	18.143	9.097
C	32.084	17.078	9.997
C	33.419	17.936	8.214
C	32.791	15.839	9.951
C	34.219	16.772	8.249
C	33.954	15.728	9.124
C	32.269	14.777	10.711
C	30.723	16.299	11.734
C	31.232	15.008	11.586
C	35.917	14.217	8.629
C	31.538	20.430	8.521
C	32.632	21.581	6.624
C	31.349	22.010	5.909
C	33.225	22.699	7.482
C	33.666	21.057	5.624
Ir	30.126	19.114	10.824
H	28.179	16.314	10.104
H	27.851	17.205	8.612
H	26.573	16.983	9.825
H	34.687	14.205	10.264
H	28.534	17.568	13.690
H	27.753	16.547	12.462
H	26.833	17.780	13.309
H	28.983	21.502	13.497
H	28.914	19.868	14.184
H	27.423	20.748	13.845
H	28.911	22.487	9.767
H	27.526	22.632	10.881
H	29.174	22.601	11.507
H	26.976	20.460	8.201
H	28.687	20.905	8.066
H	28.167	19.245	7.704
H	33.680	18.705	7.507
H	35.068	16.699	7.583
H	32.682	13.780	10.602
H	30.016	16.533	12.513
H	30.807	14.221	12.186
H	31.585	22.799	5.188
H	30.910	21.171	5.362
H	30.616	22.402	6.616
H	33.550	23.519	6.834
H	34.100	22.341	8.034

H 32.494 23.091 8.190
H 33.935 21.855 4.926
H 33.265 20.217 5.053
H 34.576 20.736 6.139
N 31.307 19.895 12.184
C 31.764 19.405 13.368
O 31.224 18.425 13.903
C 32.818 20.244 14.091
H 33.222 19.632 14.900
H 32.270 21.064 14.571
C 33.926 20.810 13.185
H 34.669 20.035 12.984
H 34.461 21.596 13.733
C 33.381 21.349 11.858
H 33.959 20.990 11.001
H 32.330 20.790 11.736
C 33.077 22.839 11.715
C 32.236 23.432 12.859
C 34.393 23.643 11.541
H 32.502 22.957 10.784
C 31.955 24.928 12.644
H 32.769 23.308 13.813
H 31.293 22.877 12.954
C 34.114 25.140 11.331
H 35.015 23.512 12.438
H 34.968 23.241 10.698
C 33.257 25.722 12.464
H 31.382 25.324 13.490
H 31.324 25.054 11.753
H 35.062 25.683 11.253
H 33.592 25.280 10.374
H 33.034 26.776 12.269
H 33.828 25.694 13.403

Ir$_{(S)}$-*pro-S* with Sav S112I

N -21.089 13.595 12.825
C -20.544 14.216 11.581
C -21.267 13.727 10.330
O -20.721 13.849 9.234
C -19.087 13.863 11.295
C -18.199 13.727 12.510
O -17.946 14.759 13.177
O -17.766 12.586 12.759
H -21.846 12.935 12.800
H -20.713 15.206 11.661
H -19.076 12.992 10.791
H -18.708 14.580 10.699
N -22.468 13.162 10.506
C -23.368 12.846 9.414
C -23.543 14.030 8.455
O -23.444 13.832 7.245
C -24.697 12.398 10.010
C -25.751 12.077 8.989
C -26.929 11.362 9.620
O -27.928 11.124 8.914
O -26.834 11.047 10.828
H -22.697 12.968 11.512
H -23.015 12.053 8.895
H -24.526 11.582 10.538
H -25.033 13.133 10.575
H -26.086 12.913 8.608
H -25.370 11.476 8.317
N -23.803 15.245 8.976
C -24.024 16.413 8.122
C -22.698 16.949 7.573
O -22.637 17.409 6.423
C -24.777 17.494 8.867
H -23.830 15.273 10.009
H -24.604 16.142 7.346
H -25.542 17.089 9.369
H -24.163 17.950 9.514
H -25.130 18.169 8.217
N -21.649 16.902 8.413
C -20.295 17.213 7.994
C -19.809 16.357 6.810
O -19.194 16.883 5.888
H -21.902 16.622 9.387
H -20.235 18.192 7.729
H -19.657 17.079 8.773
N -20.059 15.040 6.842
C -19.585 14.148 5.781
C -20.479 14.242 4.544
O -19.987 14.216 3.416
C -19.475 12.686 6.252
C -18.388 12.518 7.310
C -19.229 11.738 5.073
C -18.537 11.231 8.094
H -20.600 14.720 7.662
H -18.681 14.490 5.508
H -20.349 12.430 6.662
H -17.503 12.522 6.860
H -18.443 13.284 7.941
H -19.238 10.796 5.402
H -19.949 11.864 4.396
H -18.343 11.943 4.666
H -18.482 10.455 7.468
H -17.805 11.169 8.770
H -19.422 11.226 8.556
N -21.801 14.289 4.744
C -22.736 14.493 3.645
C -22.340 15.732 2.839
O -22.051 16.794 3.388
C -24.164 14.558 4.197
C -25.212 14.795 3.125
O -24.337 13.296 4.849
H -22.092 14.170 5.732
H -22.695 13.697 3.035
H -24.216 15.282 4.885
H -24.934 14.348 2.271
H -26.093 14.418 3.420
H -25.316 15.779 2.961
H -24.052 13.357 5.812
N -22.252 15.585 1.519
C -21.932 16.720 0.674
C -21.214 16.293 -0.603
O -21.116 15.096 -0.914
H -22.424 14.631 1.170
H -22.782 17.210 0.427
H -21.344 17.367 1.182
N -20.705 17.304 -1.314
C -19.953 17.133 -2.535
C -18.500 17.493 -2.250
O -18.185 18.586 -1.788
C -20.517 17.957 -3.696
C -19.634 17.848 -4.915
O -21.834 17.487 -4.002
H -20.907 18.251 -0.893
H -19.989 16.175 -2.810
H -20.585 18.932 -3.409
H -18.935 17.139 -4.774
H -20.183 17.605 -5.720
H -19.174 18.723 -5.089
H -21.872 16.485 -3.939
N -17.612 16.532 -2.537
C -16.197 16.644 -2.241
C -15.421 16.474 -3.528
O -15.907 15.807 -4.446
C -15.753 15.582 -1.242
C -16.402 15.570 0.094
C -17.600 14.998 0.434
C -15.873 16.144 1.283
C -16.804 15.877 2.306
C -14.691 16.829 1.588
N -17.834 15.176 1.758
C -16.579 16.263 3.623
C -14.495 17.247 2.882
C -15.422 16.963 3.883
H -18.038 15.690 -2.997
H -16.026 17.568 -1.901
H -15.906 14.670 -1.655
H -14.757 15.682 -1.089
H -18.203 14.524 -0.204
H -18.640 14.845 2.262
H -14.009 17.009 0.885
H -17.236 16.036 4.343
H -13.671 17.766 3.108
H -15.238 17.278 4.818
N -14.236 17.093 -3.572
C -13.306 16.974 -4.678
C -11.913 16.601 -4.150
O -11.432 17.082 -3.127
C -13.184 18.321 -5.437
C -14.503 18.934 -5.825
C -15.112 18.576 -7.018
C -15.173 19.826 -4.990
C -16.343 19.085 -7.380
C -16.411 20.353 -5.344
C -16.983 19.987 -6.545
O -18.157 20.454 -7.016
H -14.039 17.680 -2.720
H -13.613 16.233 -5.280
H -12.696 18.958 -4.845
H -12.654 18.153 -6.265
H -14.646 17.931 -7.635
H -14.759 20.091 -4.121
H -16.774 18.805 -8.240
H -16.877 20.991 -4.734
H -18.906 20.160 -6.417
N -11.196 15.776 -4.906
C -9.815 15.504 -4.544
C -8.881 16.370 -5.382
O -9.333 17.139 -6.237
C -9.479 14.005 -4.627
C -9.499 13.429 -6.014
H -11.677 15.376 -5.715
H -9.694 15.784 -3.589
H -8.563 13.874 -4.232
H -10.146 13.510 -4.058
N -9.410 12.096 -6.114
O -9.464 14.180 -6.991
H -9.342 11.547 -5.281
H -9.413 11.662 -7.008
N -7.572 16.158 -5.182
C -6.506 16.929 -5.819
C -6.409 16.650 -7.319
O -5.781 17.419 -8.053
C -5.182 16.693 -5.072
C -4.540 15.294 -5.234
C -5.291 14.094 -4.669
H -7.376 15.368 -4.511
H -6.720 17.910 -5.703
H -4.506 17.372 -5.390
H -5.335 16.841 -4.085
H -4.389 15.137 -6.230
H -3.616 15.327 -4.804
N -5.039 12.915 -5.249
O -6.037 14.183 -3.692
H -5.491 12.071 -4.917
H -4.387 12.885 -6.027
N -7.058 15.587 -7.804
C -7.124 15.291 -9.229
C -8.327 15.963 -9.876
O -8.480 15.862 -11.092
C -7.256 13.779 -9.432
C -6.088 12.919 -8.953
C -6.453 11.439 -9.060
C -4.836 13.252 -9.739
H -7.509 14.992 -7.075
H -6.289 15.642 -9.670
H -8.071 13.476 -8.940
H -7.370 13.611 -10.409
H -5.887 13.140 -8.003
H -6.658 11.215 -10.012
H -5.684 10.885 -8.745
H -7.254 11.253 -8.492
H -4.604 14.218 -9.610
H -4.075 12.685 -9.418
H -4.991 13.077 -10.712
N -9.227 16.493 -9.058
C -10.468 17.072 -9.553
C -11.602 16.056 -9.727
O -12.629 16.353 10.333
H -8.973 16.459 -8.049
H -10.777 17.803 -8.914
H -10.295 17.526 -10.448

N	-11.458	14.868	-9.132
C	-12.551	13.905	-9.097
C	-13.646	14.412	-8.178
O	-13.321	15.085	-7.212
C	-12.019	12.554	-8.631
O	-11.008	12.116	-9.532
H	-10.528	14.701	-8.711
H	-12.930	13.831	-10.022
H	-11.626	12.654	-7.730
H	-12.757	11.898	-8.634
H	-10.205	12.700	-9.436
N	-14.888	13.959	-8.414
C	-16.049	14.427	-7.665
C	-16.667	13.260	-6.894
O	-16.939	12.232	-7.492
C	-17.123	15.022	-8.586
C	-18.244	15.646	-7.778
O	-16.486	16.002	-9.414
H	-14.943	13.250	-9.176
H	-15.748	15.116	-7.003
H	-17.488	14.290	-9.183
H	-18.963	15.980	-8.392
H	-18.637	14.965	-7.155
H	-17.891	16.416	-7.240
H	-15.496	15.833	-9.460
N	-16.767	13.426	-5.579
C	-17.270	12.428	-4.639
C	-18.528	13.017	-4.002
O	-18.427	13.918	-3.163
C	-16.154	12.165	-3.629
C	-16.448	11.359	-2.398
C	-17.221	10.217	-2.459
C	-15.909	11.719	-1.177
C	-17.441	9.443	-1.329
C	-16.150	10.968	-0.041
C	-16.908	9.821	-0.116
H	-16.438	14.383	-5.254
H	-17.560	11.644	-5.171
H	-15.402	11.701	-4.124
H	-15.804	13.066	-3.325
H	-17.630	9.937	-3.326
H	-15.332	12.539	-1.113
H	-17.996	8.602	-1.407
H	-15.762	11.271	0.850
H	-17.072	9.266	0.698
N	-19.696	12.493	-4.384
C	-20.960	12.976	-3.844
C	-21.479	11.919	-2.885
O	-21.773	10.810	-3.308
C	-21.981	13.258	-4.962
C	-21.469	14.303	-5.953
C	-23.351	13.634	-4.393
C	-22.365	14.498	-7.146
H	-19.621	11.731	-5.083
H	-20.745	13.777	-3.297
H	-22.131	12.405	-5.462
H	-21.372	15.173	-5.471
H	-20.565	14.014	-6.270
H	-24.009	13.708	-5.139
H	-23.647	12.929	-3.754
H	-23.283	14.509	-3.920
H	-23.270	14.797	-6.841
H	-21.973	15.191	-7.751
H	-22.452	13.635	-7.644
N	-21.542	12.253	-1.609
C	-21.839	11.248	-0.606
C	-22.931	11.704	0.357
O	-23.051	12.879	0.710
C	-20.567	10.859	0.161
C	-20.023	11.999	1.005
C	-20.796	9.595	0.989
H	-21.369	13.247	-1.399
H	-22.172	10.417	-1.078
H	-19.860	10.633	-0.516
H	-19.259	12.436	0.527
H	-20.744	12.674	1.163
H	-19.703	11.643	1.884
H	-20.287	9.668	1.845
H	-21.772	9.503	1.182
H	-20.478	8.803	0.471
N	-23.682	10.704	0.811
C	-24.648	10.815	1.894
C	-24.264	9.837	3.011
O	-24.157	8.636	2.782
C	-26.056	10.472	1.395
C	-27.103	10.475	2.490
O	-26.368	11.455	0.416
H	-23.511	9.789	0.300
H	-24.612	11.742	2.267
H	-26.034	9.569	0.950
H	-27.980	10.802	2.126
H	-27.226	9.546	2.850
H	-26.815	11.079	3.238
H	-25.576	12.056	0.251
N	-24.035	10.373	4.205
C	-23.865	9.579	5.403
C	-25.227	9.371	6.078
O	-25.958	10.337	6.264
C	-22.910	10.290	6.289
H	-23.989	11.421	4.200
H	-23.497	8.681	5.157
H	-21.973	9.953	6.143
H	-22.924	11.279	6.102
H	-23.152	10.147	7.256
N	-25.578	8.119	6.368
C	-26.795	7.773	7.091
C	-26.532	7.634	8.588
O	-25.379	7.623	9.027
H	-24.894	7.398	6.027
H	-27.491	8.487	6.941
H	-27.161	6.902	6.737
N	-27.611	7.560	9.383
C	-27.503	7.551	10.840
C	-26.829	6.265	11.335
O	-26.222	6.238	12.402
C	-28.886	7.703	11.445
H	-28.515	7.510	8.874
H	-26.942	8.335	11.130
H	-28.904	7.283	12.353
H	-29.112	8.674	11.527
H	-29.561	7.254	10.860
N	-26.957	5.216	10.509
C	-26.616	3.828	10.788
C	-25.179	3.469	10.380
O	-24.856	2.285	10.326
C	-27.572	2.939	9.978
C	-27.406	3.024	8.460
O	-27.146	4.142	7.929
O	-27.537	1.963	7.809
H	-27.361	5.509	9.554
H	-26.673	3.698	11.792
H	-27.420	1.989	10.258
H	-28.508	3.205	10.209
N	-24.359	4.473	10.024
C	-22.983	4.242	9.602
C	-22.816	3.989	8.098
O	-21.713	3.718	7.665
H	-24.794	5.417	10.077
H	-22.411	5.046	9.863
H	-22.603	3.445	10.112
N	-23.874	4.093	7.285
C	-23.778	3.840	5.847
C	-23.241	5.063	5.113
O	-23.557	6.184	5.471
C	-25.116	3.488	5.267
H	-24.758	4.366	7.760
H	-23.145	3.072	5.697
H	-25.838	4.022	5.714
H	-25.128	3.687	4.283
H	-25.305	2.511	5.402
N	-22.441	4.835	4.063
C	-22.117	5.858	3.071
C	-22.697	5.383	1.740
O	-22.559	4.220	1.387
C	-20.590	6.037	2.953
C	-19.849	6.465	4.216
C	-18.353	6.570	3.960
C	-20.339	7.818	4.718
H	-22.069	3.857	4.017
H	-22.563	6.704	3.325
H	-20.208	5.162	2.661
H	-20.423	6.730	2.255
H	-20.015	5.790	4.937
H	-18.183	7.247	3.243
H	-17.888	6.851	4.800
H	-18.001	5.681	3.666
H	-21.316	7.762	4.926
H	-19.836	8.071	5.544
H	-20.188	8.510	4.011
N	-23.370	6.268	1.006
C	-23.889	5.930	-0.313
C	-23.694	7.137	-1.210
O	-23.661	8.273	-0.738
C	-25.383	5.571	-0.220
C	-25.650	4.367	0.651
O	-26.054	6.710	0.330
H	-23.490	7.200	1.449
H	-23.360	5.172	-0.680
H	-25.739	5.409	-1.152
H	-26.317	4.599	1.366
H	-26.018	3.615	0.096
H	-24.799	4.062	1.089
H	-25.452	7.196	0.971
N	-23.585	6.926	-2.507
C	-23.460	8.079	-3.382
C	-22.838	7.738	-4.722
O	-22.989	6.633	-5.220
H	-23.595	5.957	-2.816
H	-24.379	8.486	-3.534
H	-22.896	8.790	-2.923
N	-22.162	8.734	-5.306
C	-21.608	8.588	-6.637
C	-20.192	9.167	-6.660
O	-19.818	10.098	-5.929
C	-22.468	9.228	-7.745
C	-23.878	8.679	-7.843
O	-22.471	10.646	-7.582
H	-22.077	9.593	-4.732
H	-21.549	7.614	-6.860
H	-22.051	9.010	-8.649
H	-24.115	8.512	-8.804
H	-23.946	7.815	-7.336
H	-24.530	9.336	-7.456
H	-21.891	11.083	-8.278
N	-19.402	8.588	-7.543
C	-18.017	8.995	-7.722
C	-17.757	9.191	-9.209
O	-18.105	8.350	-10.037
C	-17.044	7.934	-7.192
C	-15.654	8.457	-6.931
C	-14.728	8.621	-7.951
C	-15.281	8.807	-5.652
C	-13.451	9.109	-7.698
C	-14.031	9.324	-5.380
C	-13.115	9.454	-6.405
O	-11.908	10.000	-6.179
H	-19.848	7.824	-8.096
H	-17.881	9.872	-7.263
H	-17.412	7.575	-6.337
H	-16.983	7.204	-7.868
H	-15.001	8.358	-8.967
H	-15.934	8.684	-4.897
H	-12.793	9.209	-8.440
H	-13.789	9.602	-4.451
H	-11.663	10.615	-6.934
N	-17.111	10.305	-9.529
C	-16.743	10.605	-10.901
C	-15.253	10.889	-10.942
O	-14.784	11.831	-10.315
C	-17.523	11.803	-11.417
C	-17.108	12.195	-12.816
C	-18.004	13.233	-13.475
O	-17.688	13.601	-14.620
O	-19.011	13.656	-12.857
H	-16.895	10.930	-8.726

H	-16.954	9.828	-11.488
H	-18.470	11.557	-11.443
H	-17.339	12.560	-10.826
H	-16.195	12.579	-12.776
H	-17.126	11.387	-13.389
N	-14.460	10.045	-11.767
C	-13.010	10.004	-11.716
C	-12.468	10.854	-12.823
O	-12.571	10.474	-14.021
C	-12.533	8.561	-11.834
O	-11.147	8.528	-12.001
H	-14.943	9.602	-12.584
H	-12.657	10.389	-10.736
H	-12.787	8.031	-10.895
H	-13.064	8.036	-12.663
H	-10.981	8.282	-12.950
N	-11.759	12.047	12.507
C	-11.177	12.916	-13.531
C	-10.028	12.241	-14.289
O	-9.533	12.816	-15.248
C	-10.719	14.214	-12.898
H	-11.489	12.087	-11.504
H	-11.898	13.159	-14.198
H	-10.639	14.922	-13.601
H	-11.384	14.507	-12.209
H	-9.829	14.081	-12.461
N	-9.577	10.905	-13.987
C	-8.433	10.278	-14.686
C	-8.711	8.866	-15.158
O	-9.103	7.983	-14.355
C	-7.152	10.331	-13.823
C	-6.661	11.773	-13.669
C	-7.305	9.686	-12.442
H	-9.929	10.457	-13.118
H	-8.217	10.852	-15.613
H	-6.361	9.779	-14.376
H	-6.741	12.226	-14.558
H	-7.192	12.284	-12.990
H	-5.699	11.758	-13.391
H	-6.356	9.776	-11.879
H	-8.092	10.184	-11.841
H	-7.539	8.608	-12.533
N	-8.237	8.442	-16.442
C	-7.463	7.200	-16.610
C	-8.162	6.213	-17.504
O	-7.673	5.956	-18.635
H	-8.258	9.143	-17.217

H	-6.475	7.449	-17.049
H	-7.284	6.709	-15.631
N	-9.236	5.506	-17.046
C	-10.109	4.696	-17.916
C	-11.538	4.744	-17.394
O	-12.136	3.685	-17.066
C	-9.561	3.251	-18.021
C	-10.282	2.420	-19.036
H	-9.485	5.622	-16.037
H	-10.128	5.143	-18.937
H	-8.499	3.279	-18.314
H	-9.645	2.748	-17.038
N	-10.058	1.098	-19.120
O	-11.075	2.940	-19.855
H	-9.411	0.617	-18.469
H	-10.601	0.566	-19.831
N	-12.132	5.953	-17.179
C	-13.454	6.104	-16.555
C	-14.344	7.030	-17.361
O	-13.843	7.743	-18.275
C	-13.256	6.645	-15.138
H	-11.598	6.821	-17.407
H	-13.975	5.124	-16.491
H	-12.720	7.619	-15.165
H	-14.230	6.790	-14.632
H	-12.664	5.921	-14.543
N	-15.683	7.088	-17.105
C	-16.592	8.060	-17.733
C	-17.880	8.218	-16.918
O	-18.548	7.213	-16.659
C	-16.919	7.635	-19.158
C	-17.690	8.660	-19.971
C	-18.040	8.254	-21.394
O	-18.835	9.002	-22.006
O	-17.542	7.189	-21.904
H	-16.097	6.362	-16.487
H	-16.054	9.031	-17.809
H	-15.982	7.386	-19.689
H	-17.471	6.824	-19.097
H	-18.546	8.849	-19.500
H	-17.141	9.488	-20.029
N	-18.208	9.463	-16.525
C	-19.433	9.776	-15.819
C	-19.369	9.201	-14.386
O	-18.282	9.003	-13.833
C	-20.638	9.301	-16.620
O	-21.839	9.884	-16.131

H	-17.488	10.182	-16.780
H	-19.512	10.770	-15.737
H	-20.519	9.564	-17.574
H	-20.709	8.310	-16.544
H	-22.430	9.166	-15.767
N	-20.535	8.989	-13.774
C	-20.639	8.667	-12.353
C	-20.733	7.159	-12.121
O	-21.370	6.419	-12.880
C	-21.892	9.328	-11.774
C	-21.805	10.836	-11.682
C	-23.164	11.427	-11.329
N	-23.040	12.859	-11.158
C	-24.016	13.666	-10.735
N	-23.730	14.912	-10.389
N	-25.249	13.199	-10.593
H	-21.368	9.072	-14.401
H	-19.816	9.012	-11.881
H	-22.663	9.094	-12.358
H	-22.036	8.967	-10.857
H	-21.163	11.094	-10.970
H	-21.526	11.210	-12.558
H	-23.785	11.230	-12.072
H	-23.466	11.013	-10.484
H	-22.149	13.289	-11.375
H	-22.806	15.274	-10.454
H	-24.469	15.514	-10.051
H	-25.445	12.242	-10.824
H	-25.977	13.793	-10.260
N	-20.161	6.716	-10.998
C	-20.180	5.321	-10.574
C	-20.797	5.258	-9.172
O	-20.590	6.156	-8.349
C	-18.750	4.735	-10.549
C	-18.168	4.637	-11.931
C	-17.534	5.742	-12.530
C	-18.221	3.468	-12.609
C	-16.944	5.590	-13.734
C	-17.628	3.356	-13.815
C	-16.959	4.394	-14.359
O	-16.232	4.226	-15.533
H	-19.688	7.472	-10.439
H	-20.769	4.807	-11.192
H	-18.199	5.306	-9.948
H	-18.808	3.809	-10.186
H	-17.499	6.702	-12.024
H	-18.714	2.605	-12.172

H	-16.450	6.432	-14.190
H	-17.669	2.421	-14.350
H	-16.242	3.264	-15.858
N	-21.525	4.170	-8.883
C	-22.064	3.942	-7.554
C	-20.911	3.707	-6.575
O	-19.974	3.007	-6.922
C	-23.024	2.744	-7.542
C	-23.479	2.454	-6.122
C	-24.234	2.978	-8.460
H	-21.664	3.522	-9.682
H	-22.549	4.766	-7.261
H	-22.519	1.947	-7.854
H	-22.721	2.062	-5.600
H	-23.779	3.303	-5.686
H	-24.240	1.804	-6.139
H	-24.889	2.239	-8.326
H	-24.652	3.853	-8.225
H	-23.923	2.992	-9.408
N	-21.014	4.298	-5.382
C	-20.141	3.991	-4.268
C	-20.972	3.572	-3.062
O	-22.099	4.019	-2.891
C	-19.242	5.171	-3.894
C	-19.885	6.365	-3.191
C	-19.890	6.259	-1.652
C	-19.154	7.627	-3.612
H	-21.787	5.006	-5.328
H	-19.546	3.225	-4.522
H	-18.520	4.824	-3.289
H	-18.821	5.513	-4.738
H	-20.842	6.460	-3.509
H	-18.950	6.193	-1.329
H	-20.324	7.071	-1.272
H	-20.400	5.445	-1.385
H	-19.223	7.741	-4.605
H	-19.564	8.420	-3.159
H	-18.190	7.560	-3.351
N	-20.399	2.673	-2.255
C	-20.975	2.373	-0.958
C	-19.827	2.264	0.034
O	-18.721	1.885	-0.366
C	-21.866	1.139	-1.070
C	-21.104	-0.128	-1.333
O	-22.583	0.974	0.151
H	-19.545	2.232	-2.630
H	-21.559	3.130	-0.681

H	-22.542	1.283	-1.820
H	-21.631	-0.733	-1.940
H	-20.223	0.078	-1.773
H	-20.926	-0.615	-0.471
H	-22.651	1.850	0.641
N	-20.110	2.548	1.311
C	-19.145	2.322	2.368
C	-19.735	2.498	3.753
O	-20.955	2.415	3.915
H	-21.060	2.934	1.465
H	-18.766	1.382	2.284
H	-18.364	2.963	2.254
N	-18.831	2.714	4.717
C	-19.148	2.779	6.136
C	-18.324	3.879	6.776
O	-17.203	4.124	6.339
C	-18.833	1.441	6.814
C	-19.549	0.239	6.229
C	-21.076	0.156	6.385
N	-21.434	0.079	7.788
C	-22.639	0.310	8.304
N	-23.674	0.552	7.527
N	-22.802	0.286	9.611
H	-17.850	2.836	4.348
H	-20.119	2.997	6.243
H	-17.848	1.282	6.741
H	-19.086	1.515	7.778
H	-19.347	0.182	5.226
H	-19.161	-0.616	6.639
H	-21.463	0.972	5.981
H	-21.381	-0.658	5.912
H	-20.696	-0.175	8.447
H	-23.581	0.548	6.531
H	-24.576	0.743	7.934
H	-22.028	0.075	10.215
H	-23.701	0.476	10.013
N	-18.858	4.495	7.847
C	-18.109	5.400	8.697
C	-18.510	5.111	10.153
O	-19.570	4.513	10.401
C	-18.365	6.870	8.320
C	-19.712	7.403	8.757
C	-20.893	7.024	8.131
C	-19.791	8.330	9.794
C	-22.124	7.487	8.574
C	-21.003	8.856	10.202
C	-22.172	8.421	9.607

O	-23.333	8.970	10.042
H	-19.867	4.258	8.005
H	-17.139	5.203	8.605
H	-17.654	7.427	8.746
H	-18.305	6.949	7.326
H	-20.858	6.404	7.344
H	-18.949	8.620	10.253
H	-22.970	7.155	8.160
H	-21.035	9.548	10.925
H	-24.109	8.454	9.676
N	-17.647	5.534	11.052
C	-17.861	5.517	12.499
C	-18.845	6.624	12.883
O	-18.481	7.806	12.931
C	-16.528	5.683	13.227
C	-16.584	5.601	14.752
O	-17.675	5.329	15.287
O	-15.527	5.881	15.370
H	-16.743	5.904	10.631
H	-18.299	4.646	12.725
H	-15.903	4.966	12.898
H	-16.146	6.578	12.974
N	-20.085	6.202	13.171
C	-21.183	7.115	13.489
C	-21.171	7.576	14.954
O	-21.955	8.435	15.319
C	-22.510	6.505	13.097
O	-22.677	5.210	13.637
H	-20.196	5.163	13.148
H	-21.082	7.939	12.908
H	-23.251	7.090	13.430
H	-22.561	6.446	12.098
H	-21.827	4.917	14.075
N	-20.255	7.066	15.781
C	-20.109	7.530	17.151
C	-18.629	7.716	17.485
O	-18.064	7.031	18.329
C	-20.755	6.534	18.072
H	-19.665	6.318	15.360
H	-20.570	8.415	17.249
H	-21.295	7.012	18.770
H	-21.361	5.927	17.552
H	-20.054	5.981	18.528
N	-17.933	8.659	16.843
C	-16.475	8.748	17.001
C	-16.009	9.324	18.347
O	-16.811	9.869	19.100
C	-16.084	9.682	15.853
C	-17.300	10.591	15.713
C	-18.506	9.709	15.993
H	-16.044	7.835	16.842
H	-15.279	10.218	16.083
H	-15.934	9.175	15.011
H	-17.245	11.341	16.373
H	-17.340	10.967	14.787
H	-19.213	10.214	16.477
H	-18.873	9.326	15.150
N	-14.696	9.223	18.611
C	-14.078	9.705	19.838
C	-14.177	11.225	19.917
O	-14.243	11.926	18.904
C	-12.638	9.244	19.897
H	-14.151	8.759	17.840
H	-14.563	9.312	20.625
H	-12.085	9.919	20.388
H	-12.582	8.365	20.373
H	-12.277	9.138	18.968
N	-14.159	11.736	21.148
C	-14.402	13.149	21.401
C	-13.105	13.868	21.791
O	-13.158	14.907	22.448
C	-15.480	13.271	22.489
C	-16.857	12.816	22.049
O	-14.990	12.459	23.558
H	-13.958	11.045	21.899
H	-14.751	13.573	20.558
H	-15.520	14.221	22.802
H	-16.796	12.345	21.164
H	-17.242	12.185	22.728
H	-17.467	13.607	21.957
H	-14.032	12.198	23.391
N	-11.948	13.347	21.350
C	-10.633	13.808	21.784
C	-9.909	14.603	20.684
O	-8.709	14.871	20.790
C	-9.805	12.616	22.271
C	-9.515	11.590	21.184
O	-10.282	11.548	20.184
O	-8.503	10.870	21.316
H	-12.077	12.568	20.656
H	-10.775	14.445	22.555
H	-8.938	12.964	22.617
H	-10.310	12.168	23.003
N	-10.636	15.008	19.630
C	-10.043	15.717	18.502
C	-9.565	14.767	17.395
O	-8.983	15.222	16.421
H	-11.647	14.772	19.694
H	-10.722	16.359	18.112
H	-9.255	16.266	18.824
N	-9.801	13.460	17.567
C	-9.556	12.429	16.563
C	-10.473	12.599	15.358
O	-11.591	13.076	15.474
C	-9.740	11.045	17.140
O	-8.583	10.656	17.853
H	-10.191	13.233	18.526
H	-8.604	12.523	16.244
H	-10.516	11.049	17.763
H	-9.893	10.399	16.400
H	-8.739	10.771	18.834
N	-10.022	12.134	14.188
C	-10.885	12.109	13.032
C	-11.869	10.943	13.086
O	-11.795	10.093	13.970
H	-9.043	11.810	14.196
H	-11.405	12.974	12.973
H	-10.329	12.028	12.191
N	-12.791	10.948	12.117
C	-13.823	9.936	11.967
C	-13.391	8.946	10.888
O	-13.275	9.366	9.728
C	-15.167	10.590	11.627
C	-16.275	9.588	11.439
O	-15.386	11.495	12.726
H	-12.699	11.768	11.458
H	-13.916	9.439	12.829
H	-15.069	11.128	10.792
H	-16.028	8.926	10.723
H	-16.442	9.089	12.295
H	-17.124	10.054	11.167
H	-16.326	11.847	12.702
N	-13.202	7.694	11.304
C	-12.748	6.613	10.426
C	-13.854	6.229	9.456
O	-15.033	6.230	9.801
C	-12.270	5.432	11.219
H	-13.407	7.546	12.322
H	-11.955	6.936	9.901
H	-13.054	4.911	11.567
H	-11.714	4.831	10.640
H	-11.714	5.740	11.995
N	-13.472	6.027	8.182
C	-14.443	5.680	7.164
C	-13.739	5.003	5.992
O	-12.511	5.016	5.864
C	-15.226	6.931	6.725
C	-14.436	8.146	6.231
C	-13.682	7.838	4.953
C	-15.382	9.318	6.010
H	-12.461	6.140	8.013
H	-15.103	5.033	7.551
H	-15.842	6.654	5.981
H	-15.778	7.232	7.508
H	-13.785	8.420	6.946
H	-14.328	7.571	4.237
H	-13.176	8.649	4.656
H	-13.038	7.087	5.114
H	-15.838	9.546	6.871
H	-14.863	10.110	5.687
H	-16.068	9.069	5.325
N	-14.552	4.420	5.134
C	-14.041	3.910	3.875
C	-15.181	3.700	2.908
O	-16.337	3.706	3.314
H	-15.540	4.359	5.423
H	-13.387	4.566	3.487
H	-13.568	3.039	4.033
N	-14.847	3.566	1.624
C	-15.839	3.314	0.589
C	-15.181	2.625	-0.583
O	-13.949	2.644	-0.731
C	-16.595	4.591	0.199
C	-15.836	5.573	-0.655
C	-15.718	5.633	-2.008
C	-15.041	6.651	-0.136
C	-14.468	7.310	-1.229
C	-14.743	7.080	1.163
N	-14.893	6.672	-2.358
C	-13.618	8.407	-1.071
C	-13.946	8.189	1.311
C	-13.369	8.811	0.202
H	-13.827	3.658	1.431
H	-16.524	2.689	0.969
H	-17.418	4.336	-0.310
H	-16.861	5.076	1.035
H	-16.162	5.015	-2.654
H	-14.643	6.924	-3.296
H	-15.098	6.595	1.956
H	-13.222	8.859	-1.872
H	-13.779	8.553	2.224
H	-12.745	9.589	0.366
N	-16.020	1.991	-1.409
C	-15.631	1.311	-2.618
C	-16.391	1.812	-3.851
O	-17.599	2.076	-3.786
C	-15.939	-0.190	-2.491
C	-15.479	-1.008	-3.679
O	-15.338	-0.709	-1.307
H	-17.030	2.038	-1.080
H	-14.641	1.467	-2.782
H	-16.951	-0.313	-2.418
H	-15.997	-0.747	-4.499
H	-14.502	-0.849	-3.848
H	-15.623	-1.985	-3.501
H	-15.432	-1.711	-1.274
N	-15.650	1.875	-4.969
C	-16.191	2.027	-6.301
C	-15.711	0.915	-7.097
O	-14.500	0.778	-7.364
C	-15.766	3.349	-6.977
C	-16.267	3.429	-8.426
C	-16.335	4.523	-6.202
H	-14.611	1.801	-4.778
H	-17.189	1.994	-6.243
H	-14.656	3.435	-6.992
H	-16.101	4.443	-8.845
H	-15.705	2.725	-9.073
H	-17.350	3.191	-8.471
H	-17.312	4.378	-6.035
H	-15.858	4.614	-5.326
H	-16.210	5.360	-6.737
N	-16.650	0.053	-7.641
C	-16.295	-0.752	-8.803
C	-16.422	0.093	-10.049
O	-17.407	0.877	-10.152
C	-17.164	-1.991	-8.803
H	-17.637	0.333	-7.467
H	-15.249	-1.111	-8.714
H	-16.762	-2.709	-9.375
H	-17.219	-2.321	-7.858
H	-18.089	-1.792	-9.132
N	-15.446	0.100	-11.007
C	-15.468	0.999	-12.179
C	-16.246	0.381	-13.310

O	-15.800	0.418	-14.489
C	-14.038	1.349	-12.620
C	-13.289	2.004	-11.509
C	-12.316	1.444	-10.785
C	-13.467	3.284	-11.026
C	-12.575	3.422	-10.006
C	-14.314	4.331	-11.393
N	-11.847	2.308	-9.859
C	-12.501	4.592	-9.265
C	-14.239	5.482	-10.683
C	-13.327	5.609	-9.600
H	-14.655	-0.567	-10.902
H	-15.984	1.946	-11.914
H	-13.514	0.422	-12.936
H	-14.056	2.043	-13.489
H	-11.953	0.434	-10.908
H	-11.076	2.153	-9.181
H	-15.013	4.219	-12.209
H	-11.815	4.667	-8.437
H	-14.897	6.308	-10.929
H	-13.292	6.528	-9.031
N	-17.585	-0.055	-13.076
C	-18.554	-0.370	-14.119
C	-19.799	0.495	-13.951
O	-20.357	0.616	-12.867
C	-18.925	-1.847	-13.989
C	-20.051	-2.304	-14.923
C	-20.407	-3.749	-14.737
C	-21.531	-4.179	-15.664
N	-22.788	-3.514	-15.277
H	-17.904	-0.192	-12.099
H	-18.090	-0.239	-15.114
H	-18.115	-2.395	-14.192
H	-19.216	-2.018	-13.048
H	-20.861	-1.733	-14.744
H	-19.756	-2.150	-15.873
H	-19.607	-4.314	-14.933
H	-20.703	-3.900	-13.795
H	-21.292	-3.926	-16.599
H	-21.641	-5.169	-15.600
H	-22.700	-2.523	-15.403
H	-23.540	-3.854	-15.845
H	-22.991	-3.707	-14.315
N	-20.192	1.155	-15.057
C	-21.486	1.821	-15.147
C	-22.050	1.517	-16.547
O	-21.551	0.653	-17.241
C	-21.348	3.310	-14.790
C	-20.500	4.111	-15.767
H	-19.494	1.144	-15.828
H	-22.093	1.388	-14.495
H	-22.266	3.711	-14.764
H	-20.933	3.376	-13.880
N	-20.126	5.319	-15.373
O	-20.096	3.607	-16.815
H	-20.399	5.657	-14.468
H	-19.571	5.896	-15.972
N	-23.041	2.289	-16.980
C	-23.624	2.133	-18.309
C	-22.709	2.585	-19.449
O	-22.965	2.255	-20.599
C	-24.932	2.911	-18.419
C	-26.019	2.293	-17.574
H	-23.362	3.008	-16.297
H	-23.794	1.139	-18.446
H	-24.772	3.840	-18.107
H	-25.222	2.904	-19.369
N	-26.846	3.134	-16.971
O	-26.074	1.073	-17.430
H	-26.735	4.122	-17.094
H	-27.585	2.785	-16.392
N	-21.638	3.310	-19.162
C	-20.765	3.807	-20.221
C	-19.501	2.981	-20.378
O	-18.914	2.959	-21.459
C	-20.325	5.239	-19.916
C	-21.504	6.160	-19.795
C	-22.451	5.951	-18.802
C	-21.727	7.176	-20.711
C	-23.573	6.751	-18.694
C	-22.840	7.998	-20.610
C	-23.771	7.765	-19.609
O	-24.889	8.529	-19.462
H	-21.479	3.488	-18.157
H	-21.278	3.769	-21.095
H	-19.835	5.236	-19.058
H	-19.753	5.553	-20.658
H	-22.320	5.198	-18.147
H	-21.076	7.321	-21.455
H	-24.234	6.598	-17.957
H	-22.971	8.752	-21.251
H	-24.704	9.274	-18.820
N	-18.997	2.410	-19.267
C	-17.653	1.868	-19.280
C	-17.463	0.830	-18.251
O	-17.437	1.150	-17.036
C	-16.676	3.063	-19.092
C	-15.199	2.756	-19.399
C	-14.846	2.882	-20.897
N	-15.089	4.235	-21.432
C	-14.401	5.353	-21.163
N	-13.326	5.342	-20.393
N	-14.761	6.491	-21.726
H	-19.628	2.400	-18.459
H	-17.473	1.446	-20.167
H	-17.001	3.921	-19.721
H	-16.761	3.438	-18.053
H	-14.573	3.459	-18.812
H	-14.940	1.735	-19.046
H	-13.785	2.588	-21.060
H	-15.459	2.149	-21.468
H	-15.901	4.381	-22.067
H	-12.935	4.454	-20.036
H	-12.885	6.238	-20.119
H	-15.573	6.531	-22.305
H	-14.239	7.332	-21.549
N	-17.071	-0.469	-18.666
C	-16.424	-1.389	-17.751
C	-14.922	-1.371	-17.956
O	-14.446	-1.584	-19.103
C	-17.025	-2.777	-17.972
C	-16.734	-3.672	-16.835
H	-17.062	-0.692	-19.686
H	-16.637	-1.076	-16.705
H	-18.129	-2.704	-18.060
H	-16.624	-3.232	-18.902
N	-17.123	-4.939	-16.883
O	-16.119	-3.257	-15.827
H	-17.623	-5.346	-17.694
H	-16.934	-5.516	-16.038
N	-14.085	-1.129	-16.907
C	-12.621	-1.145	-17.015
C	-12.028	-2.355	-16.307
O	-10.780	-2.423	-16.159
C	-12.094	0.179	-16.451
H	-14.513	-0.977	-15.966
H	-12.297	-1.210	-18.077
H	-12.348	0.285	-15.376
H	-10.997	0.249	-16.581
H	-12.556	1.026	-17.002
N	-12.866	-3.394	-15.772
C	-12.391	-4.580	-15.032
C	-11.510	-4.203	-13.866
O	-10.261	-4.315	-13.951
C	-11.751	-5.605	-15.989
C	-12.687	-5.972	-17.112
H	-13.821	-3.475	-16.177
H	-13.263	-5.079	-14.567
H	-10.809	-5.194	-16.403
H	-11.494	-6.533	-15.429
C	-12.426	-5.897	-18.408
N	-13.938	-6.441	-16.949
C	-14.391	-6.641	-18.202
N	-13.507	-6.323	-19.087
H	-14.454	-6.610	-16.057
H	-11.499	-5.57	-18.865
H	-15.374	-7.048	-18.443
N	-12.079	-3.540	-12.730
C	-11.256	-2.922	-11.660
C	-12.081	-2.406	-10.489
O	-13.337	-2.443	-10.537
C	-10.387	-1.785	-12.226
O	-11.184	-0.805	-12.830
H	-13.125	-3.534	-12.673
H	-10.585	-3.700	-11.254
H	-9.787	-1.313	-11.420
H	-9.648	-2.175	-12.957
H	-11.573	-1.226	-13.642
N	-11.447	-1.843	-9.332
C	-12.181	-1.484	-8.109
C	-11.321	-0.667	-7.169
O	-10.334	-1.219	-6.620
C	-12.635	-2.750	-7.370
H	-10.414	-1.926	-9.261
H	-13.062	-0.882	-8.388
H	-11.830	-3.516	-7.381
H	-12.824	-2.506	-6.296
H	-13.550	-3.175	-7.836
N	-11.623	0.623	-6.843
C	-10.941	1.324	-5.742
C	-11.629	1.137	-4.396
O	-12.853	1.322	-4.287
C	-10.691	2.794	-6.059
C	-9.850	3.462	-4.969
O	-10.029	2.883	-7.292
H	-12.327	1.140	-7.400
H	-9.934	0.909	-5.666
H	-11.665	3.296	-6.169
H	-10.407	3.521	-4.018
H	-8.914	2.893	-4.801
H	-9.594	4.498	-5.270
H	-9.061	3.023	-7.126
N	-10.827	0.959	-3.333
C	-11.277	1.256	-1.996
C	-10.423	2.384	-1.425
O	-9.208	2.379	-1.567
C	-11.216	0.050	-1.048
C	-12.019	-1.091	-1.615
O	-9.839	-0.337	-0.868
H	-9.883	0.598	-3.567
H	-12.226	1.571	-2.031
H	-11.586	0.321	-0.139
H	-11.424	-1.884	-1.784
H	-12.741	-1.363	-0.970
H	-12.447	-0.818	-2.481
H	-9.344	-0.267	-1.738
N	-11.121	3.321	-0.804
C	-10.555	4.388	-0.025
C	-10.783	4.090	1.443
O	-11.911	3.872	1.855
C	-11.171	5.740	-0.398
C	-10.909	6.247	-1.785
C	-10.041	7.263	-2.077
C	-11.582	5.941	-3.025
C	-11.013	6.765	-4.021
C	-12.533	4.999	-3.439
N	-10.092	7.550	-3.410
C	-11.407	6.712	-5.363
C	-12.946	4.962	-4.752
C	-12.397	5.857	-5.700
H	-12.173	3.211	-0.938
H	-9.566	4.401	-0.180
H	-12.170	5.678	-0.292
H	-10.827	6.438	0.241
H	-9.458	7.725	-1.410
H	-9.525	8.247	-3.874
H	-12.915	4.345	-2.774
H	-10.996	7.403	-6.083
H	-13.651	4.303	-5.019
H	-12.782	5.858	-6.709
N	-9.713	4.252	2.227
C	-9.732	4.127	3.676
C	-9.103	5.394	4.255
O	-8.025	5.820	3.836
C	-8.969	2.880	4.055

O -8.969 2.697 5.450
H -8.833 4.483 1.689
H -10.679 4.093 3.977
H -9.399 2.090 3.625
H -8.026 2.966 3.739
H -8.761 1.743 5.666
N -9.814 6.059 5.172
C -9.332 7.323 5.708
C -10.170 7.852 6.862
O -10.841 7.106 7.590
H -10.698 5.609 5.451
H -8.370 7.210 6.020
H -9.315 8.018 4.964
N -10.064 9.164 7.051
C -10.752 9.784 8.158
C -11.174 11.192 7.754
O -10.503 11.893 6.995
C -9.914 9.669 9.440
C -8.564 10.349 9.444
C -7.817 10.148 10.745
H -9.477 9.663 6.371
H -11.584 9.261 8.349
H -10.460 10.047 10.209
H -9.770 8.682 9.635
H -8.011 9.975 8.699
H -8.695 11.330 9.300
N -6.521 9.896 10.640
O -8.389 10.234 11.837
H -5.944 9.762 11.473
H -6.101 9.835 9.714
N -12.366 11.538 8.239
C -12.928 12.868 8.138
C -12.401 13.715 9.306
O -12.440 13.286 10.452
C -14.454 12.721 8.140
C -15.192 14.000 8.421
C -15.409 14.912 7.400
C -15.667 14.303 9.691
C -16.084 16.098 7.634
C -16.361 15.479 9.939
C -16.568 16.377 8.901
O -17.238 17.572 9.047
H -12.872 10.739 8.713
H -12.613 13.290 7.289
H -14.734 12.385 7.242
H -14.699 12.054 8.840
H -15.073 14.713 6.477
H -15.506 13.662 10.442
H -16.224 16.754 6.887
H -16.708 15.678 10.852
H -17.854 17.698 8.272
N -11.909 14.918 8.993
C -11.425 15.876 9.969
C -12.249 17.154 9.777
O -12.136 17.823 8.742
C -9.914 16.120 9.793
C -9.383 17.033 10.880
C -9.113 14.819 9.744
H -11.905 15.110 7.955
H -11.613 15.529 10.877
H -9.784 16.591 8.924
H -10.092 17.184 11.570
H -8.584 16.612 11.313
H -9.117 17.912 10.483
H -9.651 14.088 10.162
H -8.916 14.593 8.792
H -8.260 14.941 10.248
N -13.149 17.421 10.728
C -14.095 18.526 10.618
C -13.442 19.872 10.929
O -12.222 19.969 11.009
H -13.115 16.776 11.546
H -14.475 18.551 9.682
H -14.864 18.376 11.257
N -14.274 20.905 11.112
C -13.820 22.198 11.598
C -13.521 23.166 10.457
O -14.146 23.108 9.390
H -15.270 20.696 10.876
H -14.529 22.602 12.197
H -12.984 22.076 12.155
N -12.577 24.083 10.715
C -12.038 24.951 9.681
C -11.422 24.072 8.592
O -10.604 23.205 8.907
C -11.021 25.903 10.284
H -12.272 24.113 11.710
H -12.779 25.481 9.278
H -10.404 25.397 10.888
H -10.489 26.334 9.554
H -11.493 26.610 10.811
N -11.845 24.274 7.327
C -11.279 23.552 6.190
C -11.427 22.050 6.464
O -10.489 21.393 6.911
C -9.811 23.945 5.947
C -9.560 25.450 5.833
C -9.163 25.951 4.454
O -8.165 25.445 3.924
O -9.836 26.860 3.924
H -12.602 24.982 7.241
H -11.770 23.787 5.359
H -9.284 23.614 6.711
H -9.528 23.532 5.100
H -10.398 25.920 6.091
H -8.824 25.686 6.458
N -12.661 21.565 6.306
C -12.989 20.164 6.499
C -12.316 19.374 5.373
O -12.304 19.820 4.218
C -14.490 19.990 6.498
H -13.369 22.287 6.030
H -12.616 19.854 7.367
H -14.908 20.639 7.136
H -14.850 20.157 5.578
H -14.724 19.058 6.777
N -11.780 18.206 5.746
C -11.045 17.359 4.825
C -11.449 15.909 5.052
O -11.850 15.523 6.150
C -9.540 17.531 5.040
C -8.802 17.948 3.654
C -7.544 18.767 3.868
N -7.665 20.113 3.330
C -8.287 21.115 3.941
N -9.597 21.079 4.103
N -7.593 22.149 4.383
H -11.940 17.965 6.749
H -11.280 17.613 3.886
H -9.370 18.264 5.657
H -9.139 16.686 5.305
H -8.578 17.102 3.184
H -9.461 18.475 3.128
H -7.369 18.817 4.850
H -6.788 18.296 3.415
H -7.248 20.300 2.430
H -10.140 20.303 3.795
H -10.065 21.856 4.551
H -6.595 22.166 4.274
H -8.056 22.916 4.827
N -11.349 15.117 3.988
C -11.280 13.673 4.144
C -9.921 13.240 3.594
O -9.630 13.465 2.425
C -12.460 12.977 3.463
C -13.815 13.457 4.009
C -12.306 11.458 3.590
C -14.975 12.827 3.301
H -11.327 15.602 3.078
H -11.258 13.494 5.117
H -12.432 13.197 2.486
H -13.854 13.235 4.977
H -13.857 14.445 3.896
H -13.031 11.010 3.074
H -11.418 11.187 3.226
H -12.368 11.201 4.551
H -14.938 11.832 3.408
H -15.833 13.167 3.687
H -14.941 13.054 2.326
N -9.069 12.680 4.457
C -7.742 12.251 4.051
C -7.727 10.737 3.899
O -8.191 10.049 4.803
C -6.689 12.617 5.095
C -6.504 14.111 5.230
H -9.437 12.583 5.424
H -7.566 12.638 3.145
H -6.978 12.249 5.974
H -5.821 12.211 4.822
N -6.435 14.560 6.465
O -6.465 14.847 4.241
H -6.496 13.943 7.249
H -6.318 15.550 6.635
N -7.328 10.253 2.716
C -7.484 8.833 2.424
C -6.209 8.255 1.829
O -5.396 8.964 1.214
C -8.609 8.491 1.422
C -9.955 9.047 1.810
O -8.266 8.964 0.126
H -6.924 10.938 2.072
H -7.680 8.348 3.286
H -8.709 7.475 1.381
H -10.582 9.020 1.025
H -10.351 8.505 2.558
H -9.862 10.000 2.116
H -7.873 9.891 0.178
N -6.120 6.917 1.974
C -5.271 6.079 1.164
C -6.175 5.146 0.383
O -7.293 4.872 0.823
C -4.282 5.377 2.073
C -3.282 6.409 2.632
C -2.009 5.885 3.247
H -6.737 6.546 2.742
H -4.789 6.655 0.511
H -4.756 4.977 2.834
H -3.771 4.715 1.559
H -3.039 7.045 1.873
H -3.776 6.969 3.328
N -1.986 5.899 4.569
O -1.042 5.535 2.562
H -1.158 5.545 5.078
H -2.781 6.259 5.082
N -5.714 4.718 -0.793
C -6.573 3.876 -1.619
C -5.781 2.741 -2.247
O -4.554 2.801 -2.422
C -7.349 4.709 -2.662
C -6.522 5.561 -3.577
C -6.414 6.918 -3.512
C -5.777 5.156 -4.734
C -5.212 6.319 -5.302
C -5.542 3.917 -5.329
N -5.597 7.378 -4.525
C -4.404 6.274 -6.429
C -4.723 3.874 -6.409
C -4.156 5.055 -6.964
H -4.773 5.013 -1.043
H -7.269 3.467 -1.018
H -7.879 4.081 -3.237
H -7.978 5.319 -2.176
H -6.858 7.500 -2.834
H -5.331 8.330 -4.666
H -5.985 3.021 -4.926
H -3.965 7.174 -6.838
H -4.480 2.920 -6.851
H -3.498 4.973 -7.820
N -6.529 1.679 -2.566
C -6.051 0.497 -3.233
C -6.975 0.294 -4.436
O -8.205 0.168 -4.275
C -6.149 -0.731 -2.298
C -5.159 -0.765 -1.137
C -5.466 -1.958 -0.218

C	-3.759	-0.814	-1.650
H	-7.546	1.795	-2.269
H	-5.135	0.653	-3.571
H	-7.066	-0.740	-1.913
H	-5.995	-1.540	-2.852
H	-5.237	0.078	-0.611
H	-5.390	-2.803	-0.743
H	-4.812	-1.968	0.535
H	-6.393	-1.865	0.140
H	-3.565	-0.004	-2.213
H	-3.107	-0.837	-0.885
H	-3.621	-1.636	-2.213
N	-6.441	0.282	-5.703
C	-7.177	0.162	-6.981
C	-6.853	-1.165	-7.624
O	-5.920	-1.253	-8.464
C	-6.771	1.345	-7.887
C	-7.579	1.578	-9.186
C	-7.055	2.851	-9.835
C	-7.482	0.461	-10.221
H	-5.406	0.380	-5.773
H	-8.265	0.206	-6.821
H	-6.860	2.263	-7.269
H	-5.706	1.247	-8.151
H	-8.649	1.707	-8.948
H	-5.994	2.732	-10.138
H	-7.661	3.090	-10.721
H	-7.122	3.693	-9.119
H	-7.989	-0.454	-9.858
H	-7.998	0.757	-11.158
H	-6.419	0.246	-10.451
N	-7.523	-2.290	-7.268
C	-7.185	-3.595	-7.851
C	-7.693	-3.729	-9.260
O	-8.922	-3.631	-9.491
C	-7.698	-4.751	-7.005
C	-7.189	-6.061	-7.591
O	-7.247	-4.633	-5.692
H	-8.298	-2.216	-6.582
H	-6.094	-3.693	-7.865
H	-8.776	-4.754	-6.976
H	-7.849	-6.410	-8.414
H	-6.142	-5.991	-7.955
H	-7.187	-6.796	-6.805
H	-7.841	-4.016	-5.164
N	-6.847	-4.086	-10.266
C	-7.273	-4.303	-11.660
C	-7.402	-5.785	-11.911
O	-6.402	-6.518	-11.721
C	-6.248	-3.659	-12.630
C	-6.146	-2.135	-12.369
C	-6.633	-3.922	-14.096
C	-4.824	-1.542	-12.847
H	-5.856	-4.297	-10.024
H	-8.256	-3.826	-11.845
H	-5.254	-4.126	-12.445
H	-6.994	-1.600	-12.848
H	-6.204	-1.928	-11.282
H	-5.923	-3.434	-14.792
H	-6.607	-5.011	-14.316
H	-7.658	-3.549	-14.304
H	-4.691	-1.691	-13.937
H	-4.807	-0.453	-12.633
H	-3.980	-2.016	-12.303
N	-8.582	-6.333	-12.324
C	-8.743	-7.754	-12.667
C	-7.681	-8.241	-13.622
O	-7.635	-7.776	-14.795
H	-9.434	-5.745	-12.305
H	-8.735	-8.359	-11.736
H	-9.728	-7.914	-13.152
N	-6.694	-9.074	-13.180
C	-5.697	-9.726	-14.050
C	-5.700	-11.220	-13.860
O	-5.958	-11.702	-12.728
C	-4.266	-9.217	-13.773
C	-4.108	-7.736	-14.082
O	-3.892	-9.441	-12.442
H	-6.680	-9.296	-12.164
H	-5.939	-9.533	-15.117
H	-3.557	-9.748	-14.444
H	-4.389	-7.530	-15.136
H	-4.753	-7.135	-13.415
H	-3.054	-7.434	-13.913
H	-3.797	-10.428	-12.339
N	-4.947	-12.007	-14.775
C	-4.747	-13.468	-14.636
C	-3.882	-13.844	-13.418
O	-3.217	-12.945	-12.836
C	-4.176	-14.020	-15.969
C	-5.176	-13.866	-17.124
O	-2.964	-13.384	-16.290
H	-4.746	-11.548	-15.690
H	-5.733	-13.949	-14.462
H	-3.980	-15.083	-15.902
H	-4.880	-14.509	-17.980
H	-6.194	-14.183	-16.812
H	-5.216	-12.822	-17.491
H	-2.725	-13.735	-17.192
N	-3.742	-15.120	-13.046
C	-2.896	-15.529	-11.943
C	-1.457	-15.100	-12.216
O	-0.763	-14.619	-11.308
C	-3.058	-17.041	-11.733
C	-4.441	-17.403	-11.199
C	-5.237	-18.374	-12.058
O	-6.147	-17.912	-12.775
O	-4.964	-19.583	-11.998
H	-4.300	-15.794	-13.624
H	-3.212	-15.106	-11.092
H	-2.951	-17.476	-12.599
H	-2.401	-17.323	-11.070
H	-4.326	-17.822	-10.306
H	-4.972	-16.568	-11.127
N	-1.034	-15.231	-13.481
C	0.339	-14.923	-13.853
C	0.629	-13.436	-13.633
O	1.774	-13.059	-13.390
C	0.579	-15.311	-15.288
H	-1.754	-15.562	-14.146
H	0.963	-15.457	-13.275
H	-0.178	-14.990	-15.861
H	1.433	-14.899	-15.615
H	0.648	-16.308	-15.367
N	-0.317	-12.507	-13.974
C	-0.126	-11.053	-13.868
C	-0.797	-10.486	-12.612
O	-0.732	-9.245	-12.414
C	-0.644	-10.400	-15.170
C	0.175	-10.786	-16.365
H	-1.263	-12.857	-14.244
H	0.954	-10.806	-13.762
H	-1.694	-10.701	-15.361
H	-0.626	-9.297	-15.065
N	0.053	-11.991	-16.939
O	0.947	-9.955	-16.899
H	-0.629	-12.680	-16.575
H	0.611	-12.201	-17.790
N	-1.129	-11.348	-11.512
C	-1.646	-10.913	-10.195
C	-0.659	-10.045	-9.440
O	-1.090	-9.215	-8.598
C	-2.050	-12.141	-9.327
H	-1.135	-12.362	-11.721
H	-2.549	-10.290	-10.365
H	-1.495	-12.216	-8.495
H	-3.012	-12.026	-9.065
H	-1.957	-12.989	-9.856
N	0.689	-10.214	-9.590
C	1.656	-9.395	-8.861
C	1.573	-7.932	-9.269
O	1.734	-7.032	-8.408
C	3.072	-9.948	-9.047
C	3.573	-9.793	-10.448
C	3.340	-10.641	-11.490
C	4.286	-8.664	-10.991
C	4.487	-8.931	-12.364
C	4.836	-7.501	-10.450
N	3.890	-10.131	-12.637
C	5.185	-8.065	-13.202
C	5.511	-6.634	-11.280
C	5.698	-6.923	-12.631
H	0.943	-10.923	-10.289
H	1.440	-9.465	-7.877
H	3.690	-9.454	-8.453
H	3.068	-10.915	-8.838
H	2.843	-11.505	-11.428
H	3.861	-10.569	-13.539
H	4.743	-7.296	-9.475
H	5.302	-8.276	-14.173
H	5.872	-5.781	-10.903
H	6.220	-6.281	-13.197
N	1.249	-7.594	-10.558
C	1.017	-6.215	-11.046
C	-0.452	-5.957	-11.309
O	-0.789	-5.147	-12.219
C	1.883	-5.947	-12.294
C	1.592	-6.923	-13.446
C	2.285	-6.519	-14.750
C	2.052	-7.612	-15.794
N	2.742	-7.336	-17.058
H	1.097	-8.390	-11.207
H	1.311	-5.485	-10.262
H	1.720	-4.901	-12.636
H	2.944	-6.047	-12.000
H	1.952	-7.934	-13.164
H	0.498	-6.986	-13.630
H	1.852	-5.563	-15.112
H	3.374	-6.389	-14.575
H	2.403	-8.591	-15.381
H	0.952	-7.693	-15.975
H	2.326	-6.505	-17.535
H	3.763	-7.172	-16.904
H	2.626	-8.164	-17.694
N	-1.431	-6.446	-10.395
C	-2.834	-5.995	-10.407
C	-2.999	-4.744	-9.590
O	-3.533	-3.727	-10.110
C	-3.770	-7.090	-9.876
O	-3.531	-7.339	-8.515
H	-1.081	-6.962	-9.560
H	-3.150	-5.778	-11.451
H	-4.819	-6.759	-9.976
H	-3.642	-8.006	-10.491
H	-3.319	-8.305	-8.418
N	-2.595	-4.703	-8.287
C	-3.030	-3.647	-7.356
C	-2.178	-2.433	-7.482
O	-0.931	-2.536	-7.417
C	-3.031	-4.143	-5.899
C	-3.738	-3.173	-4.946
O	-3.648	-5.404	-5.819
H	-2.057	-5.513	-7.904
H	-4.055	-3.357	-7.612
H	-1.978	-4.265	-5.566
H	-4.535	-2.592	-5.455
H	-4.186	-3.725	-4.237
H	-3.093	-2.541	-4.509
H	-4.612	-5.281	-6.038
N	-2.780	-1.149	-7.625
C	-2.069	0.091	-7.325
C	-2.317	0.533	-5.894
O	-3.175	0.006	-5.157
C	-2.476	1.184	-8.341
C	-2.026	0.882	-9.789
C	-2.923	1.617	-10.788
C	-0.578	1.312	-10.021
H	-3.815	-1.112	-7.623
H	-0.980	-0.069	-7.405
H	-3.574	1.267	-8.315
H	-2.081	2.177	-8.036
H	-2.113	-0.205	-10.001
H	-2.931	2.706	-10.573
H	-2.565	1.446	-11.822
H	-3.959	1.225	-10.711

H 0.094 0.786 -9.315
H -0.258 1.055 -11.050
H -0.459 2.404 -9.863
N -1.575 1.592 -5.380
C -1.799 2.276 -4.093
C -1.528 3.769 -4.285
O -0.648 4.130 -5.069
C -0.920 1.699 -2.978
C 0.558 1.856 -3.262
C -1.302 2.210 -1.583
H -0.915 2.033 -6.044
H -2.761 2.171 -3.843
H -1.106 0.714 -2.923
H 1.023 0.979 -3.126
H 0.691 2.156 -4.209
H 0.951 2.537 -2.642
H -0.480 2.541 -1.123
H -1.962 2.952 -1.678
H -1.705 1.460 -1.063
N -2.363 4.577 -3.643
C -2.197 6.021 -3.654
C -2.877 6.677 -2.471
O -3.269 6.020 -1.520
H -3.136 4.093 -3.140
H -1.210 6.245 -3.640
H -2.580 6.397 -4.512
N -2.940 8.013 -2.531
C -3.470 8.826 -1.457
C -4.245 9.979 -2.099
O -3.762 10.558 -3.078
C -2.362 9.235 -0.482
C -1.203 9.803 -1.172
H -2.569 8.412 -3.426
H -4.110 8.284 -0.924
H -2.735 9.912 0.157
H -2.076 8.423 0.033
C -0.147 9.319 -1.850
N -1.149 11.177 -1.288
C -0.029 11.516 -1.929
N 0.566 10.411 -2.326
H -1.832 11.816 -0.949
H 0.090 8.364 -1.993
H 0.292 12.443 -2.078
N -5.486 10.171 -1.648
C -6.361 11.244 -2.086
C -6.759 12.084 -0.868
O -7.119 11.541 0.181
C -7.623 10.719 -2.795
C -7.458 10.204 -4.215
O -6.346 10.347 -4.754
O -8.455 9.619 -4.758
H -5.782 9.456 -0.926
H -5.832 11.840 -2.687
H -8.002 9.973 -2.226
H -8.299 11.471 -2.804
N -6.698 13.423 -1.025
C -7.250 14.347 -0.042
C -8.439 15.100 -0.638
O -8.336 15.700 -1.696
C -6.266 15.418 0.472
C -6.848 16.212 1.621
O -5.077 14.784 0.924
H -6.226 13.726 -1.901
H -7.576 13.820 0.748
H -6.028 16.038 -0.303
H -6.547 15.822 2.496
H -6.542 17.166 1.570
H -7.851 16.189 1.582
H -5.294 14.038 1.566
N -9.579 14.993 0.061
C -10.847 15.541 -0.400
C -11.192 16.802 0.374
O -11.103 16.831 1.612
C -11.964 14.524 -0.243
C -11.806 13.323 -1.140
C -11.066 12.221 -0.729
C -12.348 13.309 -2.402
C -10.888 11.148 -1.587
C -12.213 12.204 -3.242
C -11.477 11.133 -2.821
H -9.472 14.481 0.968
H -10.718 15.813 -1.359
H -11.961 14.205 0.691
H -12.816 14.963 -0.475
H -10.667 12.199 0.178
H -12.857 14.111 -2.737
H -10.308 10.371 -1.283
H -12.660 12.214 -4.148
H -11.366 10.329 -3.417
N -11.617 17.831 -0.362
C -12.201 18.992 0.312
C -13.441 19.428 -0.455
O -13.697 18.947 -1.548
C -11.249 20.184 0.482
C -9.889 19.825 1.043
O -11.078 20.860 -0.759
H -11.504 17.740 -1.369
H -12.475 18.720 1.232
H -11.661 20.841 1.151
H -9.670 18.868 0.828
H -9.184 20.417 0.644
H -9.887 19.943 2.040
H -10.647 20.253 -1.438
N -14.210 20.355 0.133
C -15.445 20.800 -0.483
C -15.187 21.871 -1.553
O -16.066 22.150 -2.353
C -16.426 21.175 0.633
C -16.920 19.968 1.415
C -18.287 20.118 2.021
C -18.790 18.821 2.609
N -19.727 19.058 3.735
H -13.849 20.707 1.035
H -15.898 20.014 -0.927
H -15.962 21.788 1.262
H -17.209 21.625 0.220
H -16.921 19.161 0.794
H -16.251 19.767 2.154
H -18.250 20.802 2.749
H -18.929 20.416 1.315
H -19.261 18.296 1.894
H -18.008 18.285 2.940
H -20.668 19.049 3.394
H -19.614 18.339 4.421
H -19.532 19.947 4.149
N -13.969 22.394 -1.652
C -13.607 23.346 -2.703
C -13.011 22.608 -3.918
O -11.976 21.976 -3.781
C -12.562 24.329 -2.133
C -12.156 25.396 -3.148
C -12.995 24.976 -0.824
H -13.297 22.072 -0.923
H -14.441 23.811 -3.010
H -11.738 23.804 -1.918
H -11.255 25.756 -2.908
H -12.120 24.989 -4.060
H -12.826 26.137 -3.138
H -12.194 25.359 -0.365
H -13.653 25.703 -1.017
H -13.419 24.285 -0.240
N -13.619 22.719 -5.106
C -13.059 22.160 -6.334
C -11.811 22.937 -6.733
O -11.861 24.166 -6.781
C -14.080 22.247 -7.474
C -13.678 21.598 -8.793
C -14.776 21.710 -9.850
C -14.320 21.329 -11.245
N -13.525 20.076 -11.238
H -14.524 23.237 -5.075
H -12.806 21.204 -6.171
H -14.933 21.814 -7.160
H -14.267 23.222 -7.649
H -12.852 22.047 -9.140
H -13.482 20.628 -8.635
H -15.534 21.108 -9.585
H -15.104 22.658 -9.869
H -15.127 21.204 -11.829
H -13.761 22.073 -11.621
H -13.641 19.602 -12.111
H -12.558 20.295 -11.105
H -13.838 19.486 -10.493
N -10.672 22.267 -7.046
C -9.382 22.952 -7.190
C -9.116 23.502 -8.603
O -10.027 23.439 -9.416
C -8.400 21.829 -6.826
C -9.060 20.585 -7.412
C -10.556 20.819 -7.296
O -8.127 24.008 -9.080
H -9.296 23.700 -6.501
H -7.513 21.979 -7.247
H -8.308 21.739 -5.842
H -8.788 20.473 -8.369
H -8.783 19.775 -6.893
H -11.027 20.568 -8.142
H -10.945 20.296 -6.534
S -9.226 5.594 -8.320
O -10.251 10.072 -8.088
N -9.098 8.562 -9.594
N -8.397 8.661 -7.330
C -10.684 3.487 -13.624
C -10.528 4.832 -12.941
C -9.504 4.771 -11.800
C -9.467 6.109 -11.080
C -8.436 6.180 -9.916
C -8.039 7.607 -9.496
C -7.945 6.200 -7.371
C -7.540 7.645 -7.918
C -9.342 9.192 -8.333
H -9.926 8.045 -9.817
H -8.950 8.200 -6.635
H -11.365 3.595 -14.477
H -11.154 2.785 -12.932
H -11.520 5.138 -12.532
H -10.218 5.606 -13.680
H -9.790 3.972 -11.080
H -8.496 4.572 -12.221
H -10.504 6.283 -10.712
H -9.209 6.895 -11.824
H -7.611 5.618 -10.300
H -7.260 7.871 -10.181
H -8.328 6.338 -6.382
H -7.105 5.558 -7.535
H -6.506 7.850 -7.733
C -1.450 0.277 -16.553
N -4.641 0.920 -15.508
O -8.580 3.579 -14.824
C -0.898 1.584 -16.062
N -9.085 1.653 -13.692
C -1.839 -0.878 -15.751
O -4.784 -2.575 -18.619
C -1.749 -0.974 -14.257
O -6.676 -3.002 -17.446
C -2.218 -1.931 -16.651
N -5.425 -1.136 -16.960
C -2.649 -3.307 -16.243
C -2.033 -1.458 -18.017
C -2.090 -2.261 -19.283
C -1.537 -0.112 -17.936
C -1.181 0.745 -19.108
C -6.382 -0.624 -16.077
C -5.944 0.519 -15.339
C -7.695 -1.042 -15.857
C -6.792 1.221 -14.442
C -8.556 -0.298 -15.038
C -8.156 0.839 -14.373
C -6.225 2.238 -13.641
C -4.131 1.884 -14.746
C -4.892 2.545 -13.768
C -9.359 2.946 -14.130
C -5.589 -2.284 -17.744
C -6.982 -4.311 -18.097
C -7.161 -4.130 -19.606

C	-5.896	-5.332	-17.755
C	-8.306	-4.706	-17.448
Ir	-3.606	-0.146	-16.969
H	-0.909	1.639	-14.969
H	-1.482	2.410	-16.469
H	0.137	1.715	-16.384
H	-9.858	1.166	-13.256
H	-2.131	-1.931	-13.893
H	-2.310	-0.182	-13.753
H	-0.714	-0.897	-13.917
H	-3.294	-3.761	-16.996
H	-3.203	-3.296	-15.298
H	-1.784	-3.962	-16.094
H	-2.457	-3.270	-19.101
H	-2.772	-1.816	-20.011
H	-1.097	-2.322	-19.738
H	-1.838	0.534	-19.956
H	-1.262	1.805	-18.868
H	-0.154	0.537	-19.427
H	-8.082	-1.928	-16.334
H	-9.582	-0.626	-14.944
H	-6.849	2.739	-12.915
H	-3.098	2.142	-14.912
H	-4.420	3.284	-13.135
H	-7.518	-5.076	-20.030
H	-7.915	-3.366	-19.815
H	-6.225	-3.864	-20.094
H	-6.225	-6.321	-18.094
H	-5.753	-5.397	-16.672
H	-4.951	-5.101	-18.243
H	-8.637	-5.670	-17.848
H	-9.082	-3.963	-17.648
H	-8.205	-4.824	-16.365
N	-4.126	1.266	-18.250
C	-4.279	2.616	-18.067
O	-3.439	3.263	-17.426
C	-5.483	3.302	-18.705
H	-5.227	4.344	-18.898
H	-6.261	3.306	-17.931
C	-5.992	2.602	-19.967
H	-5.285	2.754	-20.789
H	-6.942	3.052	-20.290
C	-6.176	1.119	-19.699
H	-5.168	0.869	-19.056
H	-6.978	0.929	-18.979
C	-6.193	0.124	-20.852
C	-7.488	0.291	-21.694
C	-4.954	0.180	-21.768
H	-6.226	-0.882	-20.410
C	-7.561	-0.764	-22.810
H	-7.506	1.295	-22.138
H	-8.370	0.214	-21.050
C	-5.030	-0.885	-22.873
H	-4.881	1.169	-22.239
H	-4.046	0.044	-21.169
C	-6.313	-0.741	-23.704
H	-8.464	-0.599	-23.411
H	-7.667	-1.761	-22.359
H	-4.150	-0.816	-23.523
H	-5.005	-1.885	-22.418
H	-6.373	-1.540	-24.452
H	-6.284	0.205	-24.262
N	21.312	-12.705	14.247
C	20.541	-13.023	13.001
C	21.338	-12.771	11.724
O	20.821	-13.019	10.641
C	19.292	-12.163	12.819
C	18.250	-12.300	13.910
O	18.256	-13.330	14.655
O	17.483	-11.343	14.049
H	22.258	-12.362	14.194
H	20.336	-14.011	13.043
H	19.584	-11.203	12.775
H	18.872	-12.413	11.942
N	22.569	-12.263	11.852
C	23.469	-12.067	10.727
C	23.599	-13.355	9.908
O	23.444	-13.323	8.689
C	24.816	-11.587	11.276
C	25.707	-10.980	10.231
C	27.098	-10.643	10.744
O	27.892	-10.142	9.946
O	27.388	-10.916	11.932
H	22.825	-12.020	12.840
H	23.122	-11.333	10.134
H	24.628	-10.900	11.955
H	25.276	-12.369	11.655
H	25.818	-11.626	9.492
H	25.302	-10.136	9.916
N	23.852	-14.489	10.591
C	23.982	-15.796	9.960
C	22.660	-16.267	9.357
O	22.631	-16.921	8.302
C	24.452	-16.789	10.998
H	23.947	-14.340	11.625
H	24.667	-15.734	9.219
H	25.154	-17.385	10.605
H	24.837	-16.302	11.784
H	23.680	-17.349	11.305
N	21.567	-16.001	10.081
C	20.243	-16.431	9.683
C	19.777	-15.722	8.411
O	19.106	-16.321	7.562
H	21.759	-15.456	10.953
H	20.242	-17.428	9.520
H	19.584	-16.240	10.424
N	20.080	-14.421	8.298
C	19.612	-13.655	7.133
C	20.534	-13.857	5.918
O	20.088	-13.843	4.761
C	19.491	-12.159	7.500
C	18.369	-11.939	8.515
C	19.318	-11.270	6.271
C	18.474	-10.625	9.301
H	20.639	-14.028	9.059
H	18.721	-14.044	6.882
H	20.349	-11.879	7.920
H	17.494	-11.948	8.034
H	18.380	-12.695	9.170
H	19.329	-10.312	6.551
H	20.066	-11.439	5.632
H	18.448	-11.480	5.830
H	18.447	-9.864	8.659
H	17.707	-10.567	9.933
H	19.334	-10.620	9.803
N	21.847	-13.928	6.153
C	22.796	-14.075	5.063
C	22.496	-15.345	4.257
O	22.356	-16.422	4.807
C	24.234	-14.084	5.597
C	25.250	-14.420	4.528
O	24.493	-12.772	6.105
H	22.114	-13.870	7.150
H	22.707	-13.287	4.445
H	24.296	-14.741	6.363
H	25.816	-15.195	4.822
H	24.783	-14.665	3.674
H	25.845	-13.629	4.358
H	24.174	-12.695	7.056
N	22.443	-15.218	2.937
C	22.183	-16.363	2.082
C	21.221	-16.036	0.946
O	21.069	-14.870	0.561
H	22.599	-14.256	2.582
H	23.056	-16.697	1.690
H	21.795	-17.117	2.637
N	20.599	-17.093	0.421
C	19.779	-17.020	-0.768
C	18.350	-17.317	-0.351
O	18.057	-18.304	0.337
C	20.263	-17.988	-1.863
C	19.409	-17.913	-3.108
O	21.619	-17.695	-2.215
H	20.761	-17.985	0.953
H	19.815	-16.094	-1.130
H	20.228	-18.942	-1.498
H	18.698	-17.214	-2.997
H	19.976	-17.674	-3.902
H	18.970	-18.800	-3.279
H	21.785	-16.704	-2.173
N	17.450	-16.438	-0.805
C	16.039	-16.525	-0.505
C	15.238	-16.547	-1.800
O	15.580	-15.869	-2.761
C	15.676	-15.318	0.344
C	16.348	-15.295	1.680
C	17.523	-14.685	1.992
C	15.802	-15.775	2.905
C	16.729	-15.477	3.913
C	14.648	-16.476	3.244
N	17.748	-14.772	3.336
C	16.522	-15.839	5.239
C	14.447	-16.849	4.548
C	15.373	-16.525	5.538
H	17.867	-15.679	-1.400
H	15.872	-17.395	-0.034
H	15.946	-14.489	-0.136
H	14.695	-15.318	0.508
H	18.132	-14.240	1.337
H	18.531	-14.383	3.825
H	13.967	-16.706	2.542
H	17.198	-15.599	5.943
H	13.624	-17.360	4.791
H	15.190	-16.801	6.483
N	14.140	-17.287	-1.772
C	13.271	-17.453	-2.912
C	11.854	-17.058	-2.535
O	11.356	-17.416	-1.478
C	13.290	-18.923	-3.365
C	14.671	-19.456	-3.619
C	15.342	-19.138	-4.785
C	15.306	-20.297	-2.713
C	16.636	-19.574	-5.020
C	16.594	-20.769	-2.945
C	17.241	-20.437	-4.118
O	18.514	-20.851	-4.380
H	13.959	-17.745	-0.842
H	13.581	-16.840	-3.645
H	12.863	-19.469	-2.650
H	12.764	-18.991	-4.207
H	14.882	-18.577	-5.484
H	14.829	-20.568	-1.877
H	17.133	-19.268	-5.833
H	17.046	-21.344	-2.264
H	19.134	-20.484	-3.686
N	11.175	-16.334	-3.435
C	9.811	-15.969	-3.126
C	8.896	-16.924	-3.871
O	9.351	-17.836	-4.557
C	9.519	-14.488	-3.373
C	9.481	-14.104	-4.833
H	11.678	-16.091	-4.282
H	9.670	-16.128	-2.153
H	8.631	-14.272	-2.961
H	10.231	-13.949	-2.918
N	9.364	-12.805	-5.095
O	9.563	-14.959	-5.702
H	9.312	-12.144	-4.346
H	9.327	-12.487	-6.043
N	7.603	-16.717	-3.667
C	6.559	-17.527	-4.259
C	6.611	-17.427	-5.790
O	6.195	-18.356	-6.440
C	5.217	-17.137	-3.612
C	4.644	-15.757	-3.989
C	5.260	-14.483	-3.414
H	7.399	-15.901	-3.030
H	6.723	-18.486	-4.016
H	4.527	-17.831	-3.861
H	5.327	-17.156	-2.608
H	4.665	-15.681	-5.017
H	3.641	-15.761	-3.747
N	4.889	-13.362	-4.025
O	6.033	-14.467	-2.446
H	5.247	-12.459	-3.692

H	4.259	-13.409	-4.813
N	7.144	-16.335	-6.368
C	7.231	-16.164	-7.824
C	8.450	-16.868	-8.440
O	8.593	-16.859	-9.661
C	7.335	-14.666	-8.147
C	6.128	-13.805	-7.775
C	6.447	-12.329	-7.989
C	4.908	-14.226	-8.573
H	7.489	-15.621	-5.690
H	6.396	-16.557	-8.243
H	8.123	-14.304	-7.655
H	7.475	-14.577	-9.130
H	5.901	-13.958	-6.815
H	6.675	-12.174	-8.950
H	5.650	-11.777	-7.742
H	7.224	-12.070	-7.415
H	4.693	-15.185	-8.380
H	4.126	-13.654	-8.319
H	5.092	-14.119	-9.552
N	9.387	-17.343	-7.598
C	10.630	-17.957	-8.052
C	11.764	-16.942	-8.221
O	12.847	-17.271	-8.690
H	9.139	-17.230	-6.587
H	10.919	-18.664	-7.386
H	10.472	-18.424	-8.937
N	11.541	-15.695	-7.806
C	12.626	-14.729	-7.803
C	13.645	-15.131	-6.731
O	13.251	-15.716	-5.733
C	12.097	-13.311	-7.577
O	11.115	-12.963	-8.544
H	10.581	-15.495	-7.504
H	13.087	-14.766	-8.688
H	11.689	-13.260	-6.670
H	12.857	-12.670	-7.646
H	10.298	-13.520	-8.401
N	14.886	-14.696	-6.920
C	16.030	-15.049	-6.088
C	16.692	-13.798	-5.515
O	17.163	-12.949	-6.259
C	17.077	-15.840	-6.890
C	18.215	-16.340	-6.026
O	16.412	-16.945	-7.502
H	14.979	-14.048	-7.756
H	15.707	-15.614	-5.321
H	17.448	-15.244	-7.622
H	18.529	-17.235	-6.354
H	18.982	-15.693	-6.059
H	17.908	-16.433	-5.074
H	15.455	-16.709	-7.711
N	16.781	-13.747	-4.185
C	17.378	-12.670	-3.402
C	18.650	-13.213	-2.722
O	18.540	-14.101	-1.869
C	16.258	-12.221	-2.451
C	16.586	-11.387	-1.251
C	17.388	-10.271	-1.351
C	15.947	-11.634	-0.043
C	17.598	-9.449	-0.252
C	16.232	-10.876	1.074
C	17.031	-9.762	0.966
H	16.355	-14.608	-3.714
H	17.678	-11.969	-4.040
H	15.585	-11.704	-3.009
H	15.792	-13.061	-2.124
H	17.824	-10.043	-2.218
H	15.271	-12.371	0.014
H	18.171	-8.624	-0.355
H	15.849	-11.149	1.976
H	17.201	-9.179	1.761
N	19.828	-12.707	-3.115
C	21.096	-13.069	-2.498
C	21.606	-11.906	-1.639
O	21.900	-10.819	-2.109
C	22.130	-13.501	-3.562
C	21.591	-14.659	-4.413
C	23.470	-13.850	-2.919
C	22.446	-14.998	-5.616
H	19.750	-12.025	-3.907
H	20.903	-13.822	-1.869
H	22.291	-12.725	-4.165
H	21.514	-15.468	-3.829
H	20.675	-14.410	-4.730
H	24.137	-14.054	-3.633
H	23.789	-13.076	-2.376
H	23.357	-14.647	-2.331
H	23.360	-15.260	-5.308
H	22.030	-15.757	-6.114
H	22.509	-14.199	-6.213
N	21.716	-12.136	-0.340
C	21.943	-11.022	0.560
C	23.036	-11.368	1.552
O	23.147	-12.517	1.984
C	20.651	-10.602	1.280
C	20.193	-11.657	2.270
C	20.836	-9.252	1.953
H	21.632	-13.114	-0.047
H	22.254	-10.236	0.017
H	19.933	-10.518	0.589
H	19.930	-11.219	3.131
H	19.407	-12.150	1.897
H	20.938	-12.302	2.443
H	21.626	-8.789	1.554
H	20.016	-8.697	1.813
H	20.986	-9.389	2.933
N	23.824	-10.346	1.895
C	24.747	-10.401	3.018
C	24.285	-9.417	4.080
O	24.171	-8.231	3.818
C	26.202	-10.094	2.609
C	27.187	-10.234	3.756
O	26.566	-10.995	1.561
H	23.717	-9.503	1.283
H	24.724	-11.322	3.408
H	26.247	-9.147	2.249
H	27.665	-11.113	3.691
H	27.861	-9.492	3.720
H	26.700	-10.191	4.632
H	25.994	-10.838	0.748
N	24.048	-9.923	5.291
C	23.777	-9.056	6.419
C	25.098	-8.689	7.108
O	25.801	-9.565	7.612
C	22.834	-9.776	7.333
H	24.076	-10.955	5.345
H	23.348	-8.212	6.094
H	22.844	-10.761	7.135
H	23.104	-9.636	8.289
H	21.899	-9.431	7.208
N	25.445	-7.403	7.110
C	26.624	-6.926	7.825
C	26.358	-6.817	9.323
O	25.205	-6.778	9.753
H	24.814	-6.781	6.567
H	27.391	-7.561	7.670
H	26.890	-6.021	7.469
N	27.431	-6.733	10.123
C	27.295	-6.723	11.579
C	26.664	-5.412	12.095
O	26.143	-5.362	13.198
C	28.656	-6.990	12.197
H	28.337	-6.677	9.629
H	26.699	-7.489	11.858
H	28.859	-7.969	12.153
H	29.358	-6.484	11.696
H	28.655	-6.695	13.153
N	26.730	-4.361	11.279
C	26.342	-2.991	11.573
C	24.899	-2.669	11.144
O	24.484	-1.525	11.230
C	27.219	-2.081	10.703
C	26.983	-2.238	9.188
O	27.057	-3.401	8.655
O	26.705	-1.182	8.524
H	27.125	-4.630	10.312
H	26.376	-2.875	12.580
H	27.026	-1.135	10.950
H	28.173	-2.294	10.892
N	24.211	-3.626	10.529
C	22.839	-3.452	10.086
C	22.685	-3.291	8.571
O	21.560	-3.055	8.134
H	24.747	-4.517	10.391
H	22.282	-4.251	10.388
H	22.436	-2.635	10.544
N	23.779	-3.441	7.786
C	23.746	-3.322	6.323
C	23.229	-4.587	5.632
O	23.520	-5.710	6.057
C	25.128	-2.970	5.805
H	24.652	-3.650	8.321
H	23.136	-2.551	6.077
H	25.360	-3.566	5.035
H	25.142	-2.017	5.501
H	25.803	-3.095	6.534
N	22.412	-4.394	4.584
C	22.056	-5.455	3.643
C	22.596	-5.049	2.282
O	22.387	-3.913	1.838
C	20.532	-5.685	3.571
C	19.783	-6.063	4.844
C	18.285	-6.121	4.549
C	20.225	-7.409	5.421
H	22.054	-3.415	4.501
H	22.502	-6.295	3.926
H	20.119	-4.836	3.219
H	20.374	-6.417	2.898
H	19.953	-5.376	5.542
H	18.113	-6.805	3.842
H	17.792	-6.368	5.382
H	17.976	-5.225	4.230
H	21.199	-7.371	5.640
H	19.701	-7.601	6.250
H	20.061	-8.128	4.746
N	23.312	-5.995	1.647
C	23.914	-5.820	0.335
C	23.778	-7.150	-0.404
O	23.771	-8.196	0.224
C	25.389	-5.393	0.459
C	25.611	-4.251	1.426
O	26.114	-6.531	0.919
H	23.398	-6.889	2.200
H	23.403	-5.132	-0.165
H	25.733	-5.140	-0.457
H	25.880	-3.424	0.925
H	24.767	-4.061	1.935
H	26.338	-4.486	2.077
H	25.632	-6.971	1.686
N	23.568	-7.107	-1.715
C	23.464	-8.318	-2.490
C	22.810	-8.058	-3.834
O	22.985	-6.993	-4.413
H	23.490	-6.153	-2.112
H	24.385	-8.708	-2.640
H	22.920	-9.003	-1.983
N	22.107	-9.071	-4.336
C	21.562	-9.009	-5.671
C	20.190	-9.656	-5.660
O	19.888	-10.532	-4.852
C	22.465	-9.698	-6.718
C	23.840	-9.081	-6.795
O	22.583	-11.081	-6.370
H	21.995	-9.880	-3.698
H	21.456	-8.050	-5.935
H	22.017	-9.633	-7.627
H	24.467	-9.693	-7.285
H	23.799	-8.203	-7.282
H	24.200	-8.920	-5.871
H	21.785	-11.377	-5.834
N	19.399	-9.236	-6.629
C	18.036	-9.687	-6.781
C	17.885	-10.088	-8.234
O	18.299	-9.343	-9.119

C	17.020	-8.574	-6.484
C	15.634	-9.081	-6.157
C	14.722	-9.376	-7.155
C	15.245	-9.300	-4.845
C	13.455	-9.847	-6.856
C	13.988	-9.802	-4.530
C	13.094	-10.065	-5.536
O	11.869	-10.570	-5.197
H	19.849	-8.545	-7.282
H	17.899	-10.492	-6.219
H	17.354	-8.049	-5.706
H	16.957	-7.989	-7.289
H	14.981	-9.247	-8.118
H	15.886	-9.093	-4.101
H	12.799	-10.032	-7.592
H	13.743	-9.968	-3.573
H	11.575	-11.223	-5.894
N	17.318	-11.264	-8.450
C	16.942	-11.657	-9.793
C	15.449	-11.920	-9.796
O	15.012	-12.812	-9.078
C	17.723	-12.904	-10.190
C	17.275	-13.478	-11.520
C	17.881	-14.833	-11.849
O	17.095	-15.769	-12.174
O	19.131	-14.950	-11.769
H	17.174	-11.849	-7.613
H	17.156	-10.928	-10.436
H	18.666	-12.654	-10.275
H	17.571	-13.586	-9.504
H	16.294	-13.592	-11.494
H	17.546	-12.854	-12.237
N	14.689	-11.114	-10.558
C	13.235	-11.262	-10.650
C	12.877	-12.240	-11.770
O	13.359	-12.118	-12.893
C	12.516	-9.930	-10.862
O	11.089	-10.102	-10.978
H	15.224	-10.384	-11.072
H	12.906	-11.649	-9.787
H	12.696	-9.333	-10.086
H	12.848	-9.508	-11.701
H	10.815	-9.892	-11.912
N	11.983	-13.175	-11.444
C	11.446	-14.127	-12.403
C	10.356	-13.459	-13.255
O	9.941	-14.030	-14.270
C	10.928	-15.340	-11.648
H	11.703	-13.161	-10.434
H	12.181	-14.429	-13.015
H	11.452	-15.459	-10.805
H	9.963	-15.208	-11.422
H	11.026	-16.156	-12.218
N	9.861	-12.274	-12.832
C	8.775	-11.592	-13.540
C	9.167	-10.141	-13.870
O	10.141	-9.567	-13.343
C	7.419	-11.638	-12.785
C	6.976	-13.063	-12.454
C	7.408	-10.762	-11.527
H	10.315	-11.904	-11.978
H	8.616	-12.070	-14.408
H	6.724	-11.250	-13.393
H	7.676	-13.707	-12.760
H	6.850	-13.150	-11.467
H	6.114	-13.258	-12.919
H	6.464	-10.625	-11.236
H	7.924	-11.222	-10.808
H	7.829	-9.882	-11.740
N	8.397	-9.577	-14.809
C	8.500	-8.178	-15.179
C	9.614	-7.900	-16.189
O	10.141	-8.821	-16.827
H	7.715	-10.240	-15.250
H	7.616	-7.865	-15.573
H	8.665	-7.618	-14.347
N	9.943	-6.600	-16.297
C	10.965	-6.054	-17.184
C	12.307	-6.117	-16.437
O	12.918	-5.101	-16.076
C	10.499	-4.680	-17.699
C	11.507	-3.936	-18.558
H	9.367	-5.980	-15.652
H	11.042	-6.665	-17.963
H	9.661	-4.819	-18.239
H	10.276	-4.109	-16.901
N	11.205	-2.674	-18.831
O	12.546	-4.474	-18.952
H	10.366	-2.262	-18.478
H	11.825	-2.123	-19.398
N	12.754	-7.363	-16.221
C	13.926	-7.683	-15.424
C	14.701	-8.785	-16.136
O	14.104	-9.756	-16.599
C	13.494	-8.135	-14.041
H	12.165	-8.103	-16.692
H	14.508	-6.876	-15.358
H	13.270	-7.337	-13.481
H	12.689	-8.724	-14.116
H	14.237	-8.645	-13.607
N	16.027	-8.655	-16.194
C	16.858	-9.700	-16.784
C	18.194	-9.808	-16.042
O	19.030	-8.894	-16.056
C	17.058	-9.435	-18.280
C	18.048	-10.373	-18.960
C	18.590	-9.861	-20.286
O	17.940	-8.972	-20.896
O	19.685	-10.316	-20.681
H	16.400	-7.778	-15.798
H	16.380	-10.582	-16.737
H	16.186	-9.551	-18.723
H	17.402	-8.518	-18.379
H	18.819	-10.499	-18.354
H	17.588	-11.230	-19.140
N	18.410	-10.978	-15.445
C	19.632	-11.187	-14.707
C	19.484	-10.480	-13.353
O	18.365	-10.296	-12.856
C	20.828	-10.705	-15.547
O	22.060	-11.253	-15.100
H	17.663	-11.674	-15.554
H	19.738	-12.159	-14.527
H	20.678	-10.974	-16.498
H	20.877	-9.707	-15.487
H	22.756	-10.538	-15.090
N	20.636	-10.110	-12.780
C	20.756	-9.686	-11.401
C	20.812	-8.161	-11.364
O	21.337	-7.506	-12.264
C	21.975	-10.357	-10.735
C	22.124	-11.843	-11.070
C	22.842	-12.727	-10.060
N	22.185	-12.674	-8.750
C	21.018	-13.246	-8.405
N	20.524	-14.258	-9.109
N	20.386	-12.827	-7.302
H	21.460	-10.156	-13.438
H	19.952	-9.987	-10.891
H	22.793	-9.888	-11.045
H	21.875	-10.272	-9.751
H	21.193	-12.239	-11.222
H	22.617	-11.929	-11.961
H	22.826	-13.659	-10.397
H	23.775	-12.400	-9.972
H	22.663	-12.146	-8.014
H	21.023	-14.622	-9.893
H	19.642	-14.662	-8.851
H	20.796	-12.112	-6.739
H	19.511	-13.232	-7.047
N	20.282	-7.616	-10.270
C	20.257	-6.190	-10.017
C	20.789	-5.983	-8.617
O	20.588	-6.859	-7.767
C	18.826	-5.671	-10.079
C	18.217	-5.762	-11.448
C	17.633	-6.934	-11.901
C	18.236	-4.661	-12.293
C	17.089	-7.021	-13.171
C	17.687	-4.734	-13.564
C	17.134	-5.923	-14.007
O	16.615	-6.025	-15.274
H	19.880	-8.322	-9.600
H	20.872	-5.742	-10.656
H	18.271	-6.211	-9.450
H	18.827	-4.715	-9.798
H	17.604	-7.738	-11.299
H	18.648	-3.809	-11.983
H	16.664	-7.873	-13.480
H	17.691	-3.930	-14.160
H	15.738	-5.548	-15.309
N	21.454	-4.845	-8.406
C	21.988	-4.515	-7.101
C	20.838	-4.273	-6.120
O	19.868	-3.611	-6.447
C	22.939	-3.310	-7.182
C	23.334	-2.794	-5.812
C	24.201	-3.645	-7.976
H	21.548	-4.238	-9.242
H	22.516	-5.297	-6.764
H	22.449	-2.567	-7.646
H	22.859	-3.319	-5.104
H	24.323	-2.890	-5.688
H	23.083	-1.828	-5.731
H	24.850	-2.890	-7.892
H	24.605	-4.482	-7.609
H	23.958	-3.778	-8.936
N	20.977	-4.789	-4.897
C	20.118	-4.339	-3.821
C	20.946	-3.842	-2.662
O	22.073	-4.273	-2.427
C	19.146	-5.429	-3.405
C	19.644	-6.594	-2.575
C	19.765	-6.267	-1.074
C	18.695	-7.764	-2.796
H	21.713	-5.498	-4.799
H	19.549	-3.580	-4.159
H	18.398	-4.988	-2.882
H	18.740	-5.814	-4.248
H	20.552	-6.883	-2.913
H	18.867	-6.007	-0.725
H	20.096	-7.074	-0.591
H	20.407	-5.513	-0.956
H	18.686	-8.008	-3.767
H	19.001	-8.550	-2.258
H	17.772	-7.504	-2.510
N	20.341	-2.889	-1.971
C	20.857	-2.388	-0.727
C	19.714	-2.219	0.272
O	18.587	-1.870	-0.092
C	21.642	-1.106	-1.001
C	20.804	0.102	-1.336
O	22.329	-0.844	0.216
H	19.456	-2.537	-2.413
H	21.505	-3.053	-0.346
H	22.319	-1.284	-1.720
H	20.611	0.631	-0.504
H	21.291	0.691	-1.988
H	19.932	-0.184	-1.746
H	22.396	-1.681	0.773
N	20.010	-2.441	1.542
C	19.005	-2.219	2.575
C	19.594	-2.147	3.974
O	20.804	-2.021	4.152
H	20.966	-2.769	1.729
H	18.508	-1.355	2.378
H	18.318	-2.967	2.540
N	18.713	-2.301	4.954
C	19.078	-2.232	6.364
C	18.240	-3.226	7.150
O	17.121	-3.539	6.772
C	18.834	-0.819	6.911
C	19.567	0.327	6.213
C	21.047	0.445	6.477
N	21.297	0.678	7.889

C	22.467	0.470	8.479
N	23.464	-0.020	7.771
N	22.595	0.639	9.783
H	17.733	-2.477	4.629
H	20.045	-2.476	6.461
H	17.847	-0.630	6.854
H	19.104	-0.810	7.880
H	19.432	0.235	5.207
H	19.128	1.206	6.485
H	21.483	-0.404	6.201
H	21.399	1.209	5.949
H	20.537	1.017	8.456
H	23.381	-0.224	6.801
H	24.356	-0.199	8.226
H	21.806	0.945	10.324
H	23.469	0.466	10.234
N	18.757	-3.661	8.303
C	18.011	-4.521	9.190
C	18.351	-4.087	10.616
O	19.399	-3.441	10.849
C	18.291	-5.988	8.877
C	19.633	-6.501	9.371
C	20.820	-6.226	8.706
C	19.700	-7.286	10.509
C	22.051	-6.673	9.191
C	20.915	-7.775	10.977
C	22.091	-7.451	10.336
O	23.274	-7.956	10.836
H	19.723	-3.328	8.492
H	17.040	-4.371	9.048
H	17.574	-6.543	9.299
H	18.263	-6.110	7.884
H	20.800	-5.695	7.853
H	18.861	-7.508	11.007
H	22.895	-6.429	8.714
H	20.931	-8.368	11.790
H	24.038	-7.458	10.430
N	17.468	-4.468	11.519
C	17.669	-4.287	12.950
C	18.713	-5.291	13.437
O	18.413	-6.482	13.561
C	16.379	-4.458	13.741
C	16.463	-4.271	15.273
O	17.567	-3.963	15.814
O	15.397	-4.453	15.943
H	16.606	-4.915	11.121
H	18.055	-3.375	13.086
H	15.702	-3.794	13.390
H	16.021	-5.386	13.567
N	19.931	-4.793	13.730
C	21.003	-5.707	14.110
C	21.031	-5.992	15.625
O	21.861	-6.793	16.064
C	22.313	-5.201	13.596
O	22.501	-3.851	13.967
H	20.020	-3.780	13.664
H	20.848	-6.589	13.640
H	23.055	-5.744	13.981
H	22.329	-5.262	12.601
H	22.967	-3.362	13.230
N	20.126	-5.392	16.428
C	20.017	-5.724	17.856
C	18.554	-5.933	18.221
O	17.971	-5.159	18.983
C	20.614	-4.635	18.713
H	19.530	-4.691	15.955
H	20.518	-6.575	18.025
H	21.298	-5.025	19.332
H	21.053	-3.946	18.132
H	19.894	-4.196	19.255
N	17.914	-6.980	17.682
C	16.461	-7.128	17.793
C	16.010	-7.480	19.209
O	16.842	-7.717	20.082
C	16.140	-8.277	16.840
C	17.402	-9.095	16.819
C	18.549	-8.113	16.984
H	15.992	-6.289	17.469
H	15.373	-8.820	17.181
H	15.925	-7.935	15.926
H	17.399	-9.765	17.569
H	17.484	-9.591	15.947
H	19.280	-8.504	17.537
H	18.908	-7.828	16.098
N	14.690	-7.495	19.404
C	14.093	-7.801	20.690
C	14.282	-9.288	20.935
O	14.490	-10.035	19.985
C	12.635	-7.400	20.711
H	14.130	-7.266	18.551
H	14.572	-7.296	21.401
H	12.168	-7.858	21.469
H	12.558	-6.409	20.830
H	12.199	-7.663	19.849
N	14.279	-9.700	22.207
C	14.367	-11.116	22.534
C	13.084	-11.504	23.257
O	13.096	-11.792	24.451
C	15.596	-11.443	23.395
C	16.917	-11.359	22.655
O	15.527	-10.519	24.482
H	14.210	-8.947	22.910
H	14.423	-11.638	21.689
H	15.502	-12.373	23.758
H	16.971	-10.496	22.147
H	17.678	-11.402	23.307
H	16.996	-12.124	22.010
H	15.521	-9.573	24.141
N	11.974	-11.443	22.523
C	10.664	-11.789	23.044
C	9.946	-12.693	22.041
O	8.755	-12.953	22.203
C	9.882	-10.513	23.391
C	9.719	-9.540	22.224
O	10.055	-9.929	21.084
O	9.292	-8.386	22.463
H	12.132	-11.123	21.538
H	10.802	-12.330	23.875
H	8.975	-10.785	23.705
H	10.367	-10.046	24.127
N	10.668	-13.185	21.015
C	10.065	-13.989	19.952
C	9.534	-13.158	18.769
O	8.814	-13.677	17.908
H	11.678	-12.943	21.050
H	10.750	-14.653	19.602
H	9.298	-14.535	20.337
N	9.876	-11.868	18.767
C	9.580	-10.941	17.682
C	10.457	-11.219	16.475
O	11.646	-11.493	16.629
C	9.878	-9.521	18.098
O	8.869	-9.011	18.905
H	10.393	-11.571	19.640
H	8.620	-11.078	17.408
H	10.744	-9.507	18.600
H	9.965	-8.955	17.276
H	9.050	-9.230	19.867
N	9.904	-10.975	15.281
C	10.712	-10.909	14.082
C	11.687	-9.737	14.092
O	11.607	-8.797	14.897
H	8.876	-10.844	15.300
H	11.236	-11.772	13.976
H	10.108	-10.828	13.269
N	12.620	-9.814	13.141
C	13.639	-8.801	12.935
C	13.239	-7.925	11.744
O	13.179	-8.422	10.625
C	14.987	-9.477	12.669
C	16.120	-8.502	12.436
O	15.227	-10.309	13.806
H	12.549	-10.681	12.550
H	13.687	-8.219	13.746
H	14.907	-10.064	11.856
H	16.079	-7.759	13.110
H	17.003	-8.972	12.525
H	16.053	-8.109	11.515
H	16.164	-10.675	13.783
N	13.026	-6.638	12.000
C	12.616	-5.711	10.955
C	13.744	-5.445	9.950
O	14.908	-5.261	10.299
C	12.117	-4.449	11.608
H	13.176	-6.359	12.989
H	11.833	-6.108	10.454
H	11.943	-3.749	10.911
H	11.265	-4.634	12.102
H	12.801	-4.104	12.254
N	13.378	-5.342	8.659
C	14.353	-5.079	7.619
C	13.651	-4.480	6.405
O	12.419	-4.460	6.335
C	15.145	-6.355	7.269
C	14.366	-7.621	6.909
C	13.501	-7.448	5.705
C	15.361	-8.777	6.679
H	12.364	-5.464	8.490
H	15.019	-4.413	7.957
H	15.737	-6.135	6.487
H	15.725	-6.574	8.059
H	13.799	-7.882	7.706
H	14.060	-7.204	4.906
H	13.009	-8.301	5.504
H	12.828	-6.718	5.861
H	15.883	-8.926	7.515
H	14.851	-9.601	6.444
H	15.975	-8.532	5.932
N	14.462	-3.955	5.500
C	13.963	-3.499	4.218
C	15.084	-3.373	3.212
O	16.257	-3.273	3.590
H	15.459	-3.901	5.783
H	13.274	-4.150	3.873
H	13.512	-2.602	4.332
N	14.725	-3.404	1.924
C	15.742	-3.181	0.911
C	15.095	-2.607	-0.329
O	13.888	-2.596	-0.439
C	16.524	-4.468	0.620
C	15.726	-5.539	-0.047
C	15.594	-5.704	-1.386
C	14.983	-6.605	0.580
C	14.431	-7.363	-0.466
C	14.684	-6.959	1.907
N	14.832	-6.797	-1.643
C	13.595	-8.447	-0.233
C	13.888	-8.053	2.147
C	13.334	-8.770	1.083
H	13.741	-3.587	1.738
H	16.384	-2.499	1.266
H	17.289	-4.247	0.020
H	16.861	-4.838	1.482
H	15.999	-5.107	-2.081
H	14.594	-7.145	-2.558
H	15.048	-6.419	2.667
H	13.202	-8.965	-0.993
H	13.705	-8.335	3.081
H	12.728	-9.544	1.286
N	15.937	-2.086	-1.218
C	15.524	-1.456	-2.452
C	16.316	-2.090	-3.604
O	17.499	-2.392	-3.451
C	15.834	0.046	-2.455
C	15.435	0.739	-3.749
O	15.227	0.668	-1.317
H	16.952	-2.177	-0.936
H	14.556	-1.639	-2.606
H	16.845	0.176	-2.386
H	14.752	1.448	-3.558
H	16.239	1.163	-4.170
H	15.044	0.071	-4.386
H	15.345	1.665	-1.359
N	15.619	-2.266	-4.724
C	16.213	-2.565	-6.020

C	15.736	-1.490	-7.010
O	14.548	-1.274	-7.170
C	15.837	-3.969	-6.525
C	16.391	-4.216	-7.929
C	16.278	-5.094	-5.601
H	14.575	-2.168	-4.590
H	17.200	-2.487	-5.942
H	14.844	-4.013	-6.595
H	17.149	-3.589	-8.100
H	16.713	-5.158	-7.995
H	15.668	-4.060	-8.601
H	17.235	-5.315	-5.783
H	16.175	-4.800	-4.650
H	15.709	-5.900	-5.767
N	16.662	-0.819	-7.693
C	16.344	-0.040	-8.883
C	16.572	-0.928	-10.100
O	17.598	-1.602	-10.180
C	17.199	1.206	-8.959
H	17.627	-0.900	-7.304
H	15.383	0.221	-8.854
H	18.142	0.957	-9.183
H	16.844	1.817	-9.667
H	17.185	1.681	-8.077
N	15.580	-0.992	-10.989
C	15.543	-1.997	-12.039
C	16.355	-1.585	-13.275
O	15.834	-1.624	-14.388
C	14.105	-2.304	-12.397
C	13.412	-2.903	-11.217
C	12.471	-2.302	-10.439
C	13.607	-4.227	-10.686
C	12.766	-4.338	-9.559
C	14.419	-5.310	-11.013
N	12.051	-3.160	-9.472
C	12.706	-5.498	-8.792
C	14.366	-6.455	-10.241
C	13.510	-6.555	-9.154
H	14.842	-0.265	-10.863
H	15.962	-2.843	-11.676
H	13.639	-1.472	-12.621
H	14.081	-2.964	-13.119
H	12.138	-1.366	-10.563
H	11.335	-2.980	-8.792
H	15.038	-5.267	-11.801
H	12.089	-5.550	-7.999
H	14.962	-7.222	-10.478
H	13.476	-7.402	-8.630
N	17.606	-1.233	-13.028
C	18.596	-0.920	-14.056
C	19.858	-1.725	-13.746
O	20.343	-1.728	-12.609
C	18.906	0.576	-14.094
C	19.995	0.967	-15.100
C	20.571	2.324	-14.857
C	21.773	2.634	-15.733
N	22.881	1.667	-15.509
H	17.840	-1.195	-11.995
H	18.246	-1.202	-14.943
H	18.072	1.065	-14.337
H	19.209	0.859	-13.185
H	20.730	0.274	-15.052
H	19.594	0.930	-16.027
H	19.869	3.021	-15.036
H	20.859	2.398	-13.897
H	21.493	2.597	-16.695
H	22.097	3.561	-15.525
H	23.507	1.693	-16.288
H	23.374	1.914	-14.675
H	22.504	0.746	-15.414
N	20.314	-2.500	-14.741
C	21.611	-3.159	-14.692
C	22.290	-2.887	-16.039
O	21.890	-1.961	-16.741
C	21.476	-4.635	-14.305
C	20.647	-5.439	-15.286
H	19.657	-2.585	-15.547
H	22.153	-2.703	-14.000
H	22.391	-5.033	-14.261
H	21.044	-4.685	-13.405
N	20.228	-6.634	-14.910
O	20.423	-4.997	-16.406
H	20.468	-6.965	-13.988
H	19.685	-7.201	-15.518
N	23.300	-3.685	-16.403
C	24.035	-3.478	-17.649
C	23.184	-3.860	-18.862
O	23.424	-3.399	-19.972
C	25.352	-4.260	-17.643
C	26.374	-3.681	-16.686
H	23.509	-4.451	-15.734
H	24.224	-2.492	-17.724
H	25.157	-5.199	-17.373
H	25.731	-4.242	-18.565
N	27.453	-4.412	-16.460
O	26.207	-2.582	-16.163
H	27.564	-5.298	-16.917
H	28.161	-4.086	-15.833
N	22.184	-4.706	-18.632
C	21.404	-5.325	-19.684
C	20.109	-4.566	-19.979
O	19.575	-4.684	-21.080
C	21.058	-6.754	-19.251
C	22.213	-7.727	-19.205
C	23.511	-7.340	-19.526
C	21.994	-9.055	-18.862
C	24.558	-8.246	-19.509
C	23.030	-9.973	-18.842
C	24.313	-9.567	-19.170
O	25.351	-10.463	-19.150
H	22.009	-4.886	-17.610
H	21.957	-5.332	-20.534
H	20.648	-6.711	-18.335
H	20.372	-7.115	-19.892
H	23.692	-6.385	-19.776
H	21.068	-9.353	-18.623
H	25.487	-7.951	-19.739
H	22.854	-10.926	-18.593
H	24.995	-11.376	-18.959
N	19.544	-3.870	-18.977
C	18.178	-3.382	-19.091
C	17.939	-2.233	-18.131
O	18.580	-2.130	-17.088
C	17.132	-4.427	-18.696
C	17.046	-5.641	-19.597
C	15.678	-6.266	-19.493
N	15.656	-7.527	-20.213
C	14.573	-8.264	-20.402
N	14.684	-9.457	-20.962
N	13.393	-7.823	-19.992
H	20.149	-3.723	-18.157
H	18.027	-3.056	-20.031
H	17.344	-4.742	-17.768
H	16.234	-3.980	-18.692
H	17.198	-5.367	-20.539
H	17.721	-6.312	-19.314
H	15.479	-6.428	-18.537
H	15.020	-5.645	-19.895
H	16.528	-7.867	-20.596
H	15.566	-9.832	-21.231
H	13.851	-10.010	-21.123
H	13.327	-6.940	-19.522
H	12.569	-8.366	-20.149
N	16.948	-1.418	-18.463
C	16.515	-0.368	-17.556
C	15.000	-0.222	-17.675
O	14.500	0.216	-18.695
C	17.266	0.935	-17.808
C	16.939	1.989	-16.772
H	16.526	-1.590	-19.391
H	16.704	-0.677	-16.627
H	18.242	0.748	-17.775
H	17.009	1.280	-18.705
N	17.609	3.129	-16.854
O	16.099	1.752	-15.885
H	18.287	3.260	-17.581
H	17.440	3.861	-16.194
N	14.277	-0.603	-16.617
C	12.827	-0.520	-16.586
C	12.342	0.798	-15.955
O	11.148	0.979	-15.783
C	12.292	-1.712	-15.840
H	14.848	-0.964	-15.820
H	12.473	-0.562	-17.532
H	12.605	-2.558	-16.277
H	12.616	-1.696	-14.891
H	11.289	-1.695	-15.842
N	13.241	1.698	-15.592
C	12.875	3.009	-15.042
C	11.889	2.886	-13.871
O	10.894	3.603	-13.795
C	12.243	3.911	-16.109
C	13.120	4.047	-17.331
H	14.234	1.402	-15.727
H	13.713	3.456	-14.700
H	11.369	3.521	-16.394
H	12.099	4.821	-15.726
C	12.914	3.769	-18.638
N	14.398	4.554	-17.235
C	14.947	4.590	-18.464
N	14.071	4.106	-19.321
H	14.850	4.847	-16.401
H	12.086	3.392	-19.044
H	15.855	4.927	-18.673
N	12.265	2.045	-12.912
C	11.384	1.749	-11.798
C	12.268	1.303	-10.642
O	13.445	0.947	-10.858
C	10.348	0.736	-12.211
O	10.914	-0.452	-12.684
H	13.207	1.637	-13.030
H	10.925	2.586	-11.527
H	9.762	0.527	-11.419
H	9.773	1.135	-12.935
H	11.413	-0.279	-13.535
N	11.659	1.268	-9.451
C	12.339	0.782	-8.252
C	11.302	0.104	-7.370
O	10.191	0.618	-7.236
C	13.024	1.911	-7.519
H	10.680	1.610	-9.456
H	13.023	0.111	-8.519
H	12.338	2.539	-7.147
H	13.574	1.544	-6.768
H	13.621	2.412	-8.149
N	11.740	-0.967	-6.704
C	10.924	-1.608	-5.690
C	11.620	-1.527	-4.341
O	12.842	-1.737	-4.243
C	10.664	-3.063	-6.053
C	9.699	-3.717	-5.078
O	10.117	-3.102	-7.383
H	12.689	-1.286	-6.971
H	10.049	-1.121	-5.624
H	11.543	-3.571	-6.061
H	10.199	-4.339	-4.469
H	9.244	-3.014	-4.526
H	9.006	-4.238	-5.581
H	9.499	-2.324	-7.527
N	10.814	-1.285	-3.311
C	11.228	-1.458	-1.932
C	10.352	-2.508	-1.277
O	9.126	-2.483	-1.431
C	11.139	-0.184	-1.077
C	11.960	0.960	-1.641
O	9.755	0.213	-0.924
H	9.857	-0.960	-3.585
H	12.176	-1.782	-1.918
H	11.511	-0.379	-0.146
H	12.875	0.960	-1.230
H	12.048	0.861	-2.636
H	11.512	1.835	-1.439
H	9.249	0.017	-1.767
N	11.015	-3.348	-0.486
C	10.426	-4.371	0.371

C	10.630	-3.970	1.827
O	11.766	-3.708	2.239
C	11.098	-5.734	0.129
C	10.843	-6.386	-1.197
C	9.980	-7.413	-1.447
C	11.519	-6.132	-2.453
C	10.989	-7.034	-3.403
C	12.531	-5.260	-2.877
N	10.067	-7.815	-2.758
C	11.437	-7.084	-4.723
C	12.975	-5.313	-4.183
C	12.455	-6.234	-5.087
H	12.075	-3.204	-0.533
H	9.443	-4.389	0.197
H	12.092	-5.618	0.215
H	10.786	-6.373	0.839
H	9.364	-7.822	-0.768
H	9.543	-8.557	-3.175
H	12.932	-4.600	-2.237
H	11.021	-7.724	-5.376
H	13.685	-4.676	-4.479
H	12.828	-6.275	-6.012
N	9.551	-4.050	2.596
C	9.584	-3.768	4.009
C	9.013	-5.009	4.693
O	7.954	-5.492	4.302
C	8.787	-2.522	4.305
O	8.787	-2.246	5.696
H	8.682	-4.336	2.084
H	10.526	-3.672	4.295
H	9.193	-1.749	3.826
H	7.846	-2.654	4.006
H	8.583	-1.279	5.843
N	9.741	-5.543	5.674
C	9.238	-6.741	6.310
C	10.095	-7.178	7.487
O	10.729	-6.366	8.186
H	10.603	-5.067	5.908
H	8.289	-6.581	6.633
H	9.199	-7.494	5.631
N	9.933	-8.458	7.820
C	10.625	-8.966	8.994
C	11.056	-10.411	8.753
O	10.381	-11.169	8.059
C	9.781	-8.755	10.251
C	8.492	-9.531	10.279
C	7.748	-9.318	11.579
H	9.322	-9.006	7.218
H	11.454	-8.420	9.130
H	10.331	-9.022	11.051
H	9.563	-7.774	10.327
H	7.912	-9.228	9.528
H	8.693	-10.503	10.184
N	6.444	-9.061	11.465
O	8.343	-9.390	12.661
H	5.870	-8.919	12.297
H	6.033	-9.008	10.539
N	12.232	-10.712	9.321
C	12.850	-12.024	9.295
C	12.407	-12.813	10.531
O	12.414	-12.301	11.658
C	14.367	-11.852	9.205
C	15.147	-13.064	9.645
C	15.332	-14.151	8.800
C	15.635	-13.143	10.944
C	16.025	-15.284	9.223
C	16.296	-14.277	11.388
C	16.541	-15.327	10.512
O	17.221	-16.460	10.957
H	12.680	-9.882	9.804
H	12.527	-12.516	8.486
H	14.602	-11.655	8.255
H	14.628	-11.083	9.785
H	14.963	-14.125	7.869
H	15.509	-12.372	11.565
H	16.150	-16.057	8.604
H	16.599	-14.343	12.341
H	18.053	-16.562	10.421
N	11.951	-14.042	10.271
C	11.549	-14.994	11.295
C	12.459	-16.211	11.141
O	12.415	-16.897	10.111
C	10.063	-15.372	11.105
C	9.559	-16.237	12.259
C	9.177	-14.140	10.928
H	11.909	-14.263	9.240
H	11.723	-14.594	12.184
H	9.994	-15.924	10.277
H	8.856	-15.738	12.765
H	9.172	-17.082	11.895
H	10.321	-16.452	12.869
H	8.490	-14.329	10.228
H	8.728	-13.932	11.795
H	9.744	-13.368	10.645
N	13.359	-16.420	12.097
C	14.341	-17.496	12.006
C	13.768	-18.862	12.422
O	12.636	-18.952	12.897
H	13.303	-15.763	12.900
H	14.682	-17.563	11.054
H	15.134	-17.281	12.597
N	14.567	-19.929	12.249
C	14.085	-21.285	12.490
C	14.402	-22.240	11.340
O	15.051	-21.874	10.344
H	15.527	-19.703	11.937
H	14.503	-21.644	13.342
H	13.081	-21.264	12.635
N	13.917	-23.484	11.487
C	14.262	-24.549	10.550
C	13.616	-24.259	9.197
O	14.230	-24.568	8.168
C	13.862	-25.906	11.086
H	13.294	-23.611	12.303
H	15.255	-24.568	10.432
H	14.547	-26.589	10.826
H	13.795	-25.870	12.084
H	12.973	-26.171	10.707
N	12.399	-23.664	9.228
C	11.778	-23.063	8.052
C	11.709	-21.536	8.248
O	10.676	-20.948	8.549
C	10.426	-23.709	7.729
C	10.535	-25.186	7.339
C	10.041	-26.136	8.411
O	10.855	-26.923	8.946
O	8.835	-26.077	8.717
H	11.952	-23.678	10.171
H	12.318	-23.264	7.239
H	9.869	-23.657	8.534
H	10.039	-23.231	6.967
H	9.983	-25.328	6.535
H	11.486	-25.388	7.173
N	12.869	-20.917	8.092
C	13.027	-19.485	8.201
C	12.294	-18.820	7.043
O	12.101	-19.390	5.951
C	14.504	-19.155	8.231
H	13.672	-21.566	7.880
H	12.618	-19.171	9.058
H	14.848	-19.227	9.167
H	15.005	-19.795	7.646
H	14.650	-18.222	7.898
N	11.849	-17.597	7.308
C	10.987	-16.877	6.387
C	11.341	-15.392	6.486
O	11.600	-14.902	7.592
C	9.536	-17.103	6.836
C	8.448	-16.879	5.808
C	7.144	-17.450	6.339
N	7.046	-18.871	6.047
C	6.692	-19.374	4.873
N	6.225	-18.588	3.923
N	6.776	-20.670	4.652
H	12.168	-17.207	8.220
H	11.167	-17.184	5.459
H	9.463	-18.055	7.167
H	9.358	-16.490	7.619
H	8.324	-15.912	5.657
H	8.675	-17.352	4.973
H	7.123	-17.310	7.321
H	6.396	-16.967	5.901
H	7.260	-19.521	6.791
H	6.145	-17.602	4.057
H	5.941	-18.985	3.039
H	7.127	-21.278	5.373
H	6.494	-21.057	3.775
N	11.244	-14.682	5.351
C	11.125	-13.212	5.365
C	9.770	-12.831	4.765
O	9.537	-13.103	3.597
C	12.292	-12.542	4.630
C	13.646	-13.007	5.182
C	12.163	-11.016	4.666
C	14.821	-12.581	4.365
H	11.260	-15.244	4.484
H	11.098	-12.947	6.323
H	12.247	-12.808	3.668
H	13.739	-12.650	6.114
H	13.626	-14.009	5.234
H	12.887	-10.612	4.112
H	11.274	-10.753	4.301
H	12.246	-10.702	5.608
H	14.858	-11.581	4.316
H	15.668	-12.919	4.783
H	14.744	-12.952	3.437
N	8.895	-12.250	5.597
C	7.547	-11.814	5.240
C	7.560	-10.319	4.908
O	8.021	-9.531	5.741
C	6.583	-12.019	6.394
C	6.391	-13.489	6.704
H	9.282	-12.129	6.580
H	7.290	-12.296	4.401
H	6.951	-11.572	7.201
H	5.703	-11.630	6.148
N	6.446	-13.831	7.980
O	6.308	-14.301	5.786
H	6.602	-13.135	8.683
H	6.333	-14.790	8.250
N	7.231	-9.971	3.660
C	7.369	-8.588	3.202
C	6.105	-8.065	2.517
O	5.275	-8.830	2.024
C	8.527	-8.405	2.215
C	9.855	-8.923	2.701
O	8.190	-9.026	0.970
H	6.883	-10.741	3.069
H	7.556	-8.002	4.007
H	8.664	-7.408	2.046
H	9.972	-9.885	2.434
H	10.605	-8.385	2.303
H	9.909	-8.858	3.703
H	7.765	-9.924	1.130
N	5.996	-6.723	2.514
C	5.155	-5.947	1.645
C	6.072	-5.075	0.809
O	7.189	-4.759	1.233
C	4.180	-5.128	2.507
C	3.284	-6.080	3.310
C	1.979	-5.550	3.850
H	6.625	-6.272	3.253
H	4.678	-6.565	1.028
H	4.693	-4.583	3.139
H	3.608	-4.589	1.919
H	3.083	-6.891	2.717
H	3.838	-6.440	4.094
N	1.921	-5.454	5.160
O	1.017	-5.320	3.112
H	1.068	-5.087	5.621
H	2.713	-5.740	5.725
N	5.666	-4.808	-0.423
C	6.561	-4.076	-1.318
C	5.777	-2.972	-2.034
O	4.547	-3.042	-2.171

C	7.303	-5.030	-2.271
C	6.458	-5.923	-3.134
C	6.327	-7.278	-2.990
C	5.710	-5.576	-4.322
C	5.154	-6.771	-4.823
C	5.447	-4.388	-5.026
N	5.505	-7.776	-3.962
C	4.332	-6.808	-5.956
C	4.642	-4.428	-6.149
C	4.093	-5.625	-6.619
H	4.735	-5.136	-0.676
H	7.260	-3.633	-0.758
H	7.877	-4.481	-2.888
H	7.900	-5.626	-1.724
H	6.770	-7.822	-2.278
H	5.202	-8.730	-4.037
H	5.838	-3.520	-4.717
H	3.936	-7.679	-6.264
H	4.450	-3.575	-6.637
H	3.529	-5.616	-7.436
N	6.522	-1.941	-2.457
C	6.023	-0.846	-3.267
C	6.933	-0.796	-4.480
O	8.126	-0.608	-4.292
C	6.115	0.501	-2.527
C	5.242	0.632	-1.279
C	5.559	1.892	-0.484
C	3.778	0.529	-1.603
H	7.530	-2.002	-2.138
H	5.107	-1.057	-3.566
H	7.060	0.627	-2.246
H	5.840	1.213	-3.161
H	5.389	-0.174	-0.693
H	5.403	2.696	-1.058
H	4.968	1.937	0.320
H	6.516	1.871	-0.194
H	3.581	-0.360	-2.027
H	3.232	0.619	-0.765
H	3.516	1.258	-2.242
N	6.333	-0.780	-5.658
C	7.051	-0.634	-6.918
C	6.611	0.672	-7.575
O	5.432	0.844	-7.875
C	6.701	-1.835	-7.792
C	7.529	-2.073	-9.057
C	6.983	-3.280	-9.823
C	7.663	-0.871	-9.943
H	5.288	-0.881	-5.611
H	8.027	-0.577	-6.728
H	6.786	-2.662	-7.225
H	5.744	-1.738	-8.079
H	8.476	-2.248	-8.800
H	5.927	-3.113	-10.105
H	7.531	-3.425	-10.646
H	7.032	-4.091	-9.240
H	8.110	-0.123	-9.445
H	8.215	-1.097	-10.751
H	6.659	-0.541	-10.266
N	7.550	1.606	-7.694
C	7.249	2.896	-8.290
C	7.915	2.977	-9.660
O	9.085	2.637	-9.803
C	7.751	4.085	-7.467
C	7.424	5.412	-8.108
O	7.156	4.065	-6.173
H	8.482	1.338	-7.336
H	6.260	2.970	-8.417
H	8.765	4.006	-7.355
H	7.710	5.411	-9.072
H	6.436	5.584	-8.065
H	7.903	6.155	-7.630
H	7.735	3.556	-5.525
N	7.102	3.329	-10.653
C	7.567	3.600	-12.002
C	7.662	5.113	-12.197
O	6.755	5.878	-11.814
C	6.665	2.926	-13.077
C	6.451	1.408	-12.816
C	7.291	3.089	-14.473
C	5.218	1.089	-11.976
H	6.148	3.686	-10.430
H	8.500	3.250	-12.058
H	5.678	3.442	-13.091
H	6.296	0.882	-13.777
H	7.348	0.961	-12.343
H	6.640	2.641	-15.253
H	7.401	4.157	-14.734
H	8.294	2.613	-14.516
H	4.326	1.621	-12.367
H	5.013	-0.002	-12.018
H	5.393	1.358	-10.916
N	8.750	5.553	-12.853
C	8.853	6.939	-13.277
C	7.700	7.296	-14.218
O	7.398	6.552	-15.144
H	9.473	4.837	-13.019
H	8.822	7.543	-12.475
H	9.723	7.085	-13.757
N	6.997	8.386	-13.909
C	5.838	8.826	-14.666
C	5.920	10.347	-14.824
O	6.612	11.029	-14.056
C	4.482	8.481	-13.997
C	4.283	7.009	-13.702
O	4.350	9.254	-12.802
H	7.358	8.899	-13.068
H	5.868	8.417	-15.574
H	3.733	8.739	-14.633
H	4.588	6.389	-14.570
H	4.882	6.734	-12.827
H	3.216	6.815	-13.466
H	4.041	10.186	-13.020
N	5.107	10.869	-15.752
C	4.792	12.292	-15.747
C	3.986	12.630	-14.498
O	3.364	11.771	-13.852
C	4.005	12.699	-16.998
C	4.720	12.387	-18.296
O	2.782	12.002	-17.002
H	4.583	10.229	-16.386
H	5.648	12.811	-15.719
H	3.772	13.677	-16.937
H	5.393	11.506	-18.231
H	4.009	12.240	-19.136
H	5.304	13.264	-18.524
H	2.189	12.488	-17.640
N	3.926	13.919	-14.200
C	3.141	14.386	-13.059
C	1.648	14.070	-13.244
O	1.003	13.686	-12.283
C	3.442	15.873	-12.810
C	4.939	16.135	-12.611
C	5.326	17.591	-12.435
O	6.153	18.090	-13.220
O	4.794	18.218	-11.515
H	4.462	14.545	-14.821
H	3.469	13.944	-12.216
H	3.158	16.372	-13.597
H	2.985	16.138	-11.991
H	5.226	15.647	-11.796
H	5.417	15.793	-13.410
N	1.102	14.143	-14.474
C	-0.296	13.802	-14.747
C	-0.603	12.325	-14.454
O	-1.740	11.985	-14.131
C	-0.625	13.999	-16.198
H	1.766	14.464	-15.213
H	-0.890	14.372	-14.160
H	-1.450	14.655	-16.251
H	0.134	14.562	-16.633
H	-0.800	13.078	-16.797
N	0.356	11.409	-14.755
C	0.224	9.960	-14.548
C	0.776	9.526	-13.186
O	0.752	8.299	-12.896
C	0.929	9.237	-15.719
C	0.220	9.441	-17.026
H	1.300	11.857	-14.893
H	-0.851	9.669	-14.544
H	1.972	9.603	-15.799
H	0.961	8.146	-15.523
N	0.850	9.799	-18.083
O	-0.979	9.092	-17.146
H	1.868	10.016	-18.076
H	0.319	9.902	-18.971
N	1.115	10.442	-12.224
C	1.676	10.044	-10.930
C	0.691	9.230	-10.074
O	1.137	8.397	-9.274
C	2.201	11.246	-10.163
H	1.015	11.420	-12.533
H	2.498	9.474	-11.095
H	1.478	11.612	-9.577
H	2.977	10.969	-9.596
H	2.496	11.952	-10.806
N	-0.655	9.483	-10.107
C	-1.657	8.677	-9.367
C	-1.565	7.198	-9.702
O	-1.565	6.342	-8.780
C	-3.087	9.199	-9.627
C	-3.507	9.042	-11.065
C	-3.293	9.922	-12.054
C	-4.179	7.971	-11.627
C	-4.306	8.258	-12.957
C	-4.703	6.792	-11.088
N	-3.763	9.454	-13.236
C	-4.974	7.392	-13.823
C	-5.352	5.948	-11.926
C	-5.497	6.254	-13.305
H	-0.954	10.334	-10.634
H	-1.459	8.779	-8.276
H	-3.807	8.664	-8.970
H	-3.140	10.272	-9.345
H	-2.836	10.903	-11.936
H	-3.759	9.965	-14.149
H	-4.606	6.571	-10.031
H	-5.096	7.649	-14.863
H	-5.769	5.027	-11.539
H	-6.047	5.568	-13.938
N	-1.333	6.775	-11.052
C	-1.065	5.376	-11.452
C	0.417	5.130	-11.611
O	0.815	4.309	-12.484
C	-1.867	5.006	-12.723
C	-1.573	5.933	-13.914
C	-2.290	5.488	-15.192
C	-2.129	6.571	-16.265
N	-2.821	6.223	-17.511
H	-1.188	7.551	-11.730
H	-1.392	4.691	-10.640
H	-1.653	3.951	-13.005
H	-2.943	5.060	-12.482
H	-1.916	6.958	-13.672
H	-0.479	5.972	-14.108
H	-1.844	4.537	-15.546
H	-3.368	5.320	-14.983
H	-2.528	7.539	-15.871
H	-1.039	6.714	-16.463
H	-2.773	7.042	-18.166
H	-2.358	5.407	-17.973
H	-3.825	5.991	-17.334
N	1.310	5.546	-10.575
C	2.747	5.221	-10.591
C	3.063	3.999	-9.769
O	3.847	3.149	-10.254
C	3.635	6.431	-10.222
O	3.492	6.889	-8.914
H	0.896	6.014	-9.742
H	3.043	4.969	-11.634
H	4.690	6.161	-10.299
H	3.447	7.258	-10.943
H	2.646	7.423	-8.893
N	2.297	3.624	-8.617
C	2.909	2.781	-7.564
C	2.169	1.483	-7.393

O	1.068	1.486	-6.806
C	3.019	3.542	-6.211
C	3.701	2.721	-5.140
O	3.804	4.711	-6.450
H	1.394	4.105	-8.414
H	3.947	2.548	-7.832
H	2.096	3.832	-5.923
H	3.556	3.141	-4.240
H	3.327	1.790	-5.129
H	4.688	2.672	-5.319
H	3.494	5.178	-7.285
N	2.654	0.304	-7.873
C	2.113	-1.001	-7.452
C	2.439	-1.353	-5.986
O	3.518	-1.008	-5.428
C	2.549	-2.114	-8.431
C	2.073	-1.881	-9.890
C	2.963	-2.642	-10.869
C	0.622	-2.307	-10.099
H	3.404	0.350	-8.588
H	1.014	-0.947	-7.520
H	3.650	-2.168	-8.413
H	2.175	-3.098	-8.069
H	2.149	-0.807	-10.162
H	3.032	-3.711	-10.577
H	2.563	-2.557	-11.899
H	3.975	-2.191	-10.861
H	-0.049	-1.621	-9.550
H	0.337	-2.263	-11.169
H	0.450	-3.335	-9.719
N	1.556	-2.117	-5.288
C	1.754	-2.667	-3.970
C	1.519	-4.173	-4.027
O	0.678	-4.656	-4.806
C	0.849	-1.987	-2.921
C	-0.644	-2.175	-3.180
C	1.240	-2.372	-1.503
H	0.695	-2.399	-5.795
H	2.705	-2.517	-3.698
H	1.027	-0.996	-2.962
H	-0.801	-2.265	-4.162
H	-0.960	-3.000	-2.713
H	-1.144	-1.382	-2.833
H	2.216	-2.197	-1.370
H	0.709	-1.827	-0.854
H	1.050	-3.343	-1.360
N	2.344	-4.894	-3.280
C	2.186	-6.337	-3.208
C	2.856	-6.889	-1.967
O	3.227	-6.146	-1.067
H	3.070	-4.365	-2.780
H	1.205	-6.568	-3.191
H	2.594	-6.761	-4.026
N	2.877	-8.217	-1.887
C	3.458	-8.916	-0.749
C	4.220	-10.120	-1.290
O	3.794	-10.713	-2.272
C	2.399	-9.301	0.272
C	1.215	-9.942	-0.341
C	0.828	-11.234	-0.520
N	0.197	-9.146	-0.819
C	-0.769	-9.928	-1.304
N	-0.399	-11.201	-1.161
H	2.446	-8.706	-2.701
H	4.106	-8.312	-0.298
H	2.802	-9.938	0.937
H	2.101	-8.474	0.760
H	0.182	-8.152	-0.808
H	1.325	-12.046	-0.245
H	-1.621	-9.615	-1.705
N	5.407	-10.363	-0.725
C	6.282	-11.466	-1.078
C	6.666	-12.198	0.217
O	7.044	-11.577	1.212
C	7.544	-11.007	-1.804
C	7.429	-10.614	-3.277
O	6.359	-10.873	-3.874
O	8.428	-10.020	-3.823
H	5.660	-9.656	0.025
H	5.755	-12.110	-1.624
H	7.920	-10.209	-1.297
H	8.232	-11.751	-1.733
N	6.605	-13.541	0.202
C	7.177	-14.326	1.300
C	8.389	-15.079	0.755
O	8.272	-15.777	-0.237
C	6.191	-15.330	1.909
C	6.714	-15.956	3.186
O	4.985	-14.661	2.238
H	6.140	-13.948	-0.619
H	7.485	-13.702	2.009
H	5.984	-16.049	1.214
H	7.152	-16.836	2.982
H	7.388	-15.349	3.615
H	5.959	-16.109	3.828
H	4.743	-13.989	1.526
N	9.545	-14.928	1.409
C	10.784	-15.532	0.991
C	11.119	-16.710	1.907
O	10.943	-16.599	3.120
C	11.921	-14.526	1.048
C	11.795	-13.405	0.037
C	11.089	-12.254	0.351
C	12.315	-13.543	-1.237
C	10.978	-11.238	-0.586
C	12.185	-12.532	-2.177
C	11.506	-11.385	-1.841
H	9.461	-14.320	2.267
H	10.641	-15.917	0.076
H	11.929	-14.117	1.948
H	12.767	-15.003	0.861
H	10.665	-12.152	1.239
H	12.796	-14.388	-1.487
H	10.496	-10.383	-0.326
H	12.588	-12.648	-3.097
H	11.396	-10.648	-2.520
N	11.559	-17.830	1.320
C	12.089	-18.912	2.129
C	13.421	-19.371	1.541
O	13.803	-18.979	0.441
C	11.093	-20.078	2.240
C	9.751	-19.682	2.802
O	10.899	-20.648	0.951
H	11.489	-17.846	0.296
H	12.256	-18.572	3.056
H	11.491	-20.791	2.850
H	9.801	-19.614	3.804
H	9.470	-18.792	2.432
H	9.057	-20.367	2.559
H	10.809	-19.926	0.254
N	14.072	-20.316	2.237
C	15.362	-20.865	1.823
C	15.304	-21.905	0.696
O	16.344	-22.165	0.106
C	16.044	-21.528	3.024
C	16.331	-20.592	4.177
C	17.199	-19.420	3.780
C	18.613	-19.524	4.307
N	19.410	-18.342	3.896
H	13.572	-20.624	3.103
H	15.960	-20.092	1.513
H	15.447	-22.262	3.351
H	16.909	-21.921	2.709
H	15.458	-20.242	4.540
H	16.794	-21.106	4.910
H	17.235	-19.368	2.778
H	16.788	-18.576	4.135
H	18.587	-19.574	5.305
H	19.039	-20.352	3.942
H	20.350	-18.443	4.223
H	19.411	-18.271	2.898
H	19.008	-17.515	4.289
N	14.125	-22.463	0.372
C	13.987	-23.567	-0.572
C	13.218	-23.114	-1.820
O	12.283	-22.318	-1.733
C	13.289	-24.753	0.129
C	13.942	-25.058	1.478
C	11.789	-24.529	0.300
H	13.307	-22.028	0.865
H	14.905	-23.850	-0.864
H	13.416	-25.559	-0.442
H	13.365	-24.703	2.212
H	14.044	-26.046	1.580
H	14.840	-24.621	1.516
H	11.509	-24.857	1.201
H	11.591	-23.553	0.219
H	11.300	-25.034	-0.410
N	13.619	-23.598	-3.007
C	12.934	-23.192	-4.231
C	11.497	-23.711	-4.181
O	11.254	-24.803	-3.675
C	13.708	-23.607	-5.491
C	14.906	-22.712	-5.791
C	15.116	-22.350	-7.249
C	16.201	-23.170	-7.915
N	16.266	-22.904	-9.369
H	14.412	-24.247	-2.972
H	12.913	-22.196	-4.279
H	14.036	-24.537	-5.361
H	13.084	-23.567	-6.265
H	14.807	-21.850	-5.253
H	15.747	-23.171	-5.445
H	14.257	-22.497	-7.744
H	15.367	-21.381	-7.308
H	17.085	-22.942	-7.497
H	16.013	-24.144	-7.763
H	15.756	-22.069	-9.581
H	17.220	-22.788	-9.648
H	15.865	-23.673	-9.869
N	10.490	-22.937	-4.652
C	9.091	-23.262	-4.391
C	8.489	-24.128	-5.509
O	8.174	-25.269	-5.209
C	8.444	-21.868	-4.306
C	9.346	-20.951	-5.150
C	10.615	-21.724	-5.475
O	8.261	-23.885	-6.665
H	8.986	-23.721	-3.498
H	7.519	-21.877	-4.685
H	8.415	-21.542	-3.362
H	8.860	-20.685	-5.991
H	9.557	-20.116	-4.628
H	10.655	-21.955	-6.445
H	11.428	-21.202	-5.219
C	1.772	-1.504	-17.365
N	4.540	-2.055	-15.656
O	8.273	-4.627	-14.257
S	9.132	-6.347	-7.786
C	1.061	-2.787	-17.002
N	8.756	-2.597	-13.057
O	10.053	-10.817	-7.212
C	2.148	-0.431	-16.493
N	8.940	-9.398	-8.834
O	5.973	0.205	-19.597
C	2.000	-0.371	-14.984
N	8.229	-9.311	-6.572
O	6.820	1.737	-17.975
C	2.717	0.554	-17.250
N	5.663	-0.191	-17.245
C	3.280	1.846	-16.696
C	2.709	0.130	-18.615
C	3.137	0.923	-19.831
C	2.121	-1.130	-18.666
C	1.947	-1.957	-19.919
C	6.429	-0.571	-16.113
C	5.807	-1.564	-15.245
C	7.822	-0.183	-15.885
C	6.521	-2.157	-14.116
C	8.561	-0.813	-14.787
C	7.931	-1.801	-13.898
C	5.845	-3.199	-13.335
C	3.883	-3.103	-14.938
C	4.520	-3.667	-13.745
C	9.163	-3.872	-13.510

C	10.507	-4.414	-13.142
C	10.443	-5.740	-12.369
C	9.429	-5.689	-11.218
C	9.377	-7.034	-10.496
C	8.335	-7.033	-9.337
C	7.904	-8.416	-8.814
C	7.834	-6.850	-6.801
C	7.398	-8.322	-7.240
C	9.166	-9.937	-7.527
C	6.180	0.597	-18.290
C	7.497	2.595	-18.808
C	8.636	1.867	-19.515
C	6.547	3.228	-19.818
C	8.085	3.690	-17.931
Ir	3.933	-1.255	-17.452
H	0.733	-2.805	-15.945
H	1.716	-3.651	-17.207
H	0.170	-2.919	-17.639
H	9.389	-2.122	-12.383
H	9.781	-8.919	-9.090
H	1.338	0.463	-14.678
H	2.993	-0.186	-14.516
H	1.612	-1.312	-14.551
H	8.789	-8.811	-5.911
H	3.774	2.463	-17.472
H	4.058	1.622	-15.932
H	2.485	2.454	-16.217
H	3.807	1.764	-19.570
H	3.696	0.276	-20.543
H	2.250	1.332	-20.357
H	2.938	-2.097	-20.409
H	1.270	-1.440	-20.632
H	1.546	-2.966	-19.700
H	8.327	0.527	-16.522
H	9.608	-0.631	-14.719
H	6.357	-3.702	-12.531
H	2.939	-3.495	-15.265
H	4.042	-4.447	-13.181
H	11.095	-4.553	-14.071
H	11.049	-3.668	-12.529
H	11.455	-5.963	-11.956
H	10.195	-6.573	-13.059
H	9.717	-4.890	-10.497
H	8.416	-5.470	-11.617
H	10.409	-7.229	-10.123
H	9.111	-7.833	-11.225
H	7.524	-6.484	-9.767
H	7.121	-8.713	-9.481
H	8.210	-6.923	-5.802
H	7.010	-6.203	-7.017
H	6.359	-8.490	-7.045
H	9.234	2.585	-20.116
H	9.304	1.386	-18.770
H	8.246	1.090	-20.202
H	7.068	4.027	-20.388
H	5.685	3.684	-19.291
H	6.181	2.480	-20.546
H	8.652	4.433	-18.534
H	8.768	3.257	-17.171
H	7.280	4.233	-17.392
N	4.950	-2.683	-18.507
C	4.997	-4.029	-18.078
O	3.893	-4.612	-17.470
C	6.256	-4.809	-18.222
H	6.561	-5.137	-17.207
H	7.042	-4.130	-18.616
C	6.089	-6.012	-19.168
H	5.924	-5.633	-20.203
H	5.201	-6.612	-18.876
C	7.340	-6.902	-19.125
H	8.225	-6.322	-19.466
H	7.524	-7.201	-18.072
C	7.200	-8.177	-19.984
C	7.291	-7.851	-21.492
C	8.298	-9.187	-19.598
H	6.212	-8.651	-19.779
C	7.210	-9.127	-22.341
H	8.247	-7.328	-21.712
H	6.458	-7.182	-21.795
C	8.217	-10.457	-20.454
H	9.304	-8.726	-19.719
H	8.187	-9.479	-18.531
C	8.311	-10.116	-21.943
H	7.323	-8.865	-23.416
H	6.213	-9.606	-22.213
H	9.050	-11.143	-20.178
H	7.262	-10.992	-20.250
H	8.204	-11.048	-22.543
H	9.309	-9.680	-22.170
N	20.539	13.140	-12.828
C	19.876	13.661	-11.598
C	20.731	13.425	-10.362
O	20.256	13.634	-9.254
C	18.547	12.969	-11.279
C	17.669	12.703	-12.481
O	17.255	13.682	-13.140
O	17.440	11.521	-12.762
H	21.437	12.694	-12.810
H	19.790	14.656	-11.723
H	18.758	12.096	-10.834
H	18.046	13.551	-10.636
N	21.964	12.946	-10.538
C	22.833	12.735	-9.393
C	22.892	14.043	-8.624
O	22.749	14.074	-7.405
C	24.218	12.291	-9.863
C	25.272	12.329	-8.789
C	26.576	11.703	-9.251
O	27.449	11.508	-8.406
O	26.706	11.423	-10.468
H	22.226	12.751	-11.512
H	22.464	12.019	-8.811
H	24.139	11.361	-10.181
H	24.496	12.902	-10.584
H	25.460	13.265	-8.564
H	24.957	11.811	-8.018
N	23.048	15.121	-9.401
C	23.213	16.466	-8.879
C	21.930	16.972	-8.214
O	21.967	17.537	-7.111
C	23.648	17.357	-10.033
H	23.040	14.903	-10.429
H	23.948	16.463	-8.187
H	24.118	18.163	-9.675
H	24.269	16.850	-10.632
H	22.845	17.646	-10.555
N	20.782	16.741	-8.878
C	19.505	17.204	-8.371
C	19.066	16.446	-7.114
O	18.391	17.019	-6.271
H	20.900	16.213	-9.763
H	19.562	18.192	-8.153
H	18.795	17.093	-9.085
N	19.406	15.150	-7.001
C	19.001	14.367	-5.826
C	19.899	14.646	-4.608
O	19.404	14.891	-3.510
C	18.914	12.870	-6.186
C	17.783	12.653	-7.195
C	18.738	12.017	-4.935
C	17.811	11.320	-7.871
H	19.956	14.772	-7.783
H	18.080	14.687	-5.579
H	19.767	12.610	-6.617
H	16.912	12.764	-6.713
H	17.849	13.375	-7.889
H	18.729	11.051	-5.190
H	19.493	12.188	-4.306
H	17.875	12.252	-4.491
H	17.735	10.590	-7.189
H	17.046	11.248	-8.514
H	18.672	11.211	-8.372
N	21.223	14.673	-4.825
C	22.192	14.899	-3.767
C	21.884	16.189	-3.030
O	21.730	17.241	-3.635
C	23.635	14.935	-4.306
C	24.631	15.195	-3.197
O	23.942	13.698	-4.958
H	21.489	14.517	-5.826
H	22.136	14.136	-3.112
H	23.710	15.686	-4.995
H	24.309	15.947	-2.615
H	24.741	14.372	-2.633
H	25.522	15.445	-3.587
H	23.569	13.693	-5.892
N	21.846	16.109	-1.706
C	21.570	17.267	-0.892
C	20.663	16.973	0.284
O	20.495	15.815	0.696
H	22.029	15.160	-1.323
H	22.447	17.656	-0.546
H	21.144	17.993	-1.468
N	20.106	18.059	0.813
C	19.261	18.015	1.984
C	17.810	18.234	1.557
O	17.473	19.169	0.842
C	19.776	18.986	3.053
C	18.876	18.998	4.264
O	21.092	18.558	3.458
H	20.337	18.946	0.303
H	19.328	17.109	2.402
H	19.859	19.913	2.648
H	19.418	18.864	5.099
H	18.393	19.876	4.329
H	18.197	18.260	4.198
H	21.125	17.556	3.518
N	16.942	17.323	1.994
C	15.521	17.390	1.725
C	14.713	17.379	3.006
O	15.130	16.855	4.026
C	15.163	16.188	0.885
C	15.801	16.141	-0.449
C	17.006	15.613	-0.770
C	15.228	16.624	-1.665
C	16.136	16.327	-2.680
C	14.014	17.223	-1.981
N	17.195	15.692	-2.108
C	15.879	16.607	-4.015
C	13.753	17.511	-3.295
C	14.689	17.230	-4.293
H	17.384	16.551	2.553
H	15.336	18.264	1.254
H	15.433	15.355	1.375
H	14.169	16.174	0.742
H	17.656	15.225	-0.116
H	17.994	15.337	-2.611
H	13.347	17.439	-1.270
H	16.536	16.363	-4.727
H	12.881	17.928	-3.544
H	14.478	17.493	-5.239
N	13.532	17.996	2.972
C	12.672	18.073	4.134
C	11.267	17.612	3.770
O	10.781	17.959	2.703
C	12.583	19.534	4.609
C	13.926	20.178	4.841
C	14.613	20.785	3.788
C	14.556	20.109	6.083
C	15.868	21.356	3.983
C	15.818	20.661	6.292
C	16.465	21.301	5.240
O	17.729	21.847	5.357
H	13.290	18.409	2.046
H	13.028	17.453	4.839
H	12.098	20.054	3.913
H	12.074	19.548	5.465
H	14.199	20.810	2.877
H	14.091	19.652	6.843
H	16.340	21.805	3.224
H	16.253	20.598	7.185
H	18.219	21.715	4.499
N	10.575	16.917	4.668
C	9.219	16.488	4.350
C	8.231	17.382	5.069

O	8.613	18.365	5.710
C	8.929	14.991	4.523
C	8.893	14.467	5.931
H	11.054	16.722	5.550
H	9.087	16.630	3.368
H	8.036	14.798	4.082
H	9.636	14.479	4.005
N	8.898	13.144	6.034
O	8.829	15.224	6.902
H	8.931	12.560	5.230
H	8.869	12.717	6.949
N	6.955	17.046	4.867
C	5.854	17.829	5.388
C	5.827	17.744	6.921
O	5.287	18.646	7.543
C	4.580	17.405	4.638
C	3.922	16.094	5.105
C	4.520	14.789	4.608
H	6.828	16.175	4.305
H	6.003	18.791	5.144
H	3.895	18.139	4.728
H	4.801	17.303	3.658
H	3.935	16.085	6.124
H	2.941	16.123	4.830
N	3.900	13.689	5.016
O	5.497	14.742	3.863
H	4.233	12.767	4.715
H	3.096	13.769	5.629
N	6.381	16.680	7.541
C	6.359	16.523	9.002
C	7.495	17.277	9.709
O	7.572	17.191	10.931
C	6.521	15.038	9.384
C	5.365	14.103	9.023
C	5.742	12.642	9.292
C	4.098	14.504	9.776
H	6.818	15.992	6.898
H	5.478	16.884	9.348
H	7.337	14.697	8.924
H	6.645	14.994	10.373
H	5.147	14.211	8.053
H	5.959	12.532	10.261
H	4.973	12.054	9.049
H	6.539	12.401	8.741
H	3.848	15.441	9.531
H	3.354	13.884	9.528
H	4.266	14.449	10.760
N	8.470	17.818	8.968
C	9.678	18.345	9.586
C	10.888	17.396	9.558
O	11.988	17.792	9.953
H	8.289	17.826	7.951
H	9.941	19.220	9.124
H	9.480	18.591	10.558
N	10.747	16.166	9.049
C	11.903	15.281	8.959
C	12.925	15.792	7.942
O	12.559	16.502	7.005
C	11.417	13.877	8.643
O	10.557	13.444	9.690
H	9.801	15.919	8.745
H	12.352	15.258	9.861
H	10.910	13.890	7.797
H	12.192	13.267	8.593
H	9.688	13.930	9.627
N	14.191	15.385	8.129
C	15.332	15.735	7.301
C	15.998	14.498	6.720
O	16.404	13.573	7.433
C	16.468	16.431	8.086
C	17.323	17.310	7.212
O	15.939	17.208	9.149
H	14.288	14.749	8.982
H	15.010	16.310	6.536
H	17.077	15.708	8.482
H	17.883	16.748	6.595
H	16.746	17.918	6.659
H	17.935	17.873	7.777
H	14.931	17.207	9.124
N	16.154	14.529	5.402
C	16.708	13.459	4.590
C	17.981	14.035	3.971
O	17.883	14.966	3.176
C	15.584	13.093	3.595
C	15.940	12.182	2.471
C	16.611	10.993	2.713
C	15.459	12.429	1.187
C	16.907	10.124	1.668
C	15.767	11.574	0.144
C	16.493	10.419	0.383
H	15.822	15.447	4.968
H	16.992	12.740	5.204
H	14.850	12.664	4.127
H	15.246	13.954	3.207
H	16.884	10.753	3.638
H	14.885	13.230	1.023
H	17.426	9.281	1.860
H	15.460	11.799	-0.797
H	16.718	9.800	-0.369
N	19.162	13.534	4.345
C	20.423	13.988	3.770
C	21.005	12.858	2.937
O	21.210	11.755	3.421
C	21.363	14.448	4.898
C	20.654	15.490	5.766
C	22.684	14.933	4.314
C	21.418	15.889	7.023
H	19.100	12.795	5.079
H	20.194	14.723	3.137
H	21.569	13.662	5.466
H	20.495	16.309	5.215
H	19.766	15.118	6.041
H	23.307	15.175	5.056
H	23.091	14.208	3.762
H	22.520	15.735	3.742
H	22.299	16.276	6.760
H	20.887	16.567	7.526
H	21.558	15.081	7.589
N	21.142	13.068	1.639
C	21.478	11.972	0.741
C	22.572	12.371	-0.249
O	22.606	13.529	-0.677
C	20.193	11.489	0.018
C	19.624	12.529	-0.939
C	20.409	10.151	-0.674
H	20.994	14.041	1.328
H	21.830	11.206	1.295
H	19.498	11.326	0.722
H	19.934	12.332	-1.868
H	18.625	12.499	-0.908
H	19.940	13.439	-0.668
H	21.077	10.264	-1.409
H	20.749	9.486	-0.010
H	19.541	9.832	-1.055
N	23.380	11.375	-0.642
C	24.276	11.422	-1.790
C	23.873	10.368	-2.804
O	23.718	9.187	-2.472
C	25.744	11.228	-1.371
C	26.710	11.301	-2.532
O	26.060	12.267	-0.441
H	23.305	10.518	-0.019
H	24.197	12.325	-2.220
H	25.836	10.337	-0.900
H	26.378	10.735	-3.293
H	26.798	12.250	-2.848
H	27.615	10.968	-2.249
H	25.384	12.287	0.305
N	23.668	10.810	-4.047
C	23.449	9.902	-5.162
C	24.788	9.673	-5.847
O	25.420	10.640	-6.279
C	22.432	10.457	-6.126
H	23.680	11.846	-4.142
H	23.115	9.031	-4.813
H	22.624	10.127	-7.053
H	21.512	10.162	-5.859
H	22.468	11.458	-6.121
N	25.208	8.406	-5.905
C	26.356	7.996	-6.687
C	26.035	7.821	-8.166
O	24.874	7.700	-8.583
H	24.632	7.742	-5.336
H	27.097	8.684	-6.589
H	26.718	7.119	-6.323
N	27.106	7.763	-8.971
C	26.981	7.693	-10.421
C	26.401	6.341	-10.860
O	25.830	6.221	-11.947
C	28.342	7.951	-11.048
H	28.018	7.772	-8.471
H	26.357	8.420	-10.731
H	28.269	7.890	-12.044
H	28.661	8.863	-10.793
H	28.996	7.268	-10.721
N	26.552	5.347	-9.975
C	26.238	3.939	-10.163
C	24.778	3.593	-9.808
O	24.355	2.471	-10.071
C	27.161	3.133	-9.236
C	26.910	3.312	-7.726
O	27.022	4.480	-7.178
O	26.583	2.265	-7.077
H	26.957	5.706	-9.042
H	26.350	3.731	-11.149
H	27.049	2.161	-9.455
H	28.106	3.405	-9.428
N	24.040	4.532	-9.192
C	22.671	4.307	-8.721
C	22.511	4.180	-7.196
O	21.394	3.915	-6.729
H	24.536	5.448	-9.077
H	22.075	5.071	-9.050
H	22.303	3.459	-9.159
N	23.577	4.373	-6.411
C	23.508	4.283	-4.949
C	22.942	5.546	-4.304
O	23.146	6.664	-4.780
C	24.868	3.947	-4.365
H	24.457	4.591	-6.926
H	22.911	3.501	-4.703
H	24.971	2.954	-4.303
H	25.587	4.319	-4.952
H	24.950	4.346	-3.451
N	22.172	5.349	-3.218
C	21.795	6.428	-2.317
C	22.356	6.058	-0.958
O	22.252	4.919	-0.523
C	20.276	6.618	-2.181
C	19.513	6.897	-3.475
C	18.004	6.873	-3.260
C	19.946	8.228	-4.096
H	21.874	4.358	-3.086
H	22.224	7.266	-2.622
H	19.901	5.785	-1.786
H	20.122	7.388	-1.569
H	19.761	6.202	-4.156
H	17.751	7.571	-2.591
H	17.541	7.059	-4.127
H	17.731	5.972	-2.923
H	20.923	8.194	-4.301
H	19.430	8.382	-4.938
H	19.763	8.969	-3.450
N	22.975	7.027	-0.303
C	23.590	6.780	0.980
C	23.397	8.068	1.752
O	23.395	9.139	1.161
C	25.056	6.332	0.863
C	25.276	5.161	-0.069
O	25.801	7.445	0.384
H	22.977	7.948	-0.780
H	23.099	6.060	1.441
H	25.396	6.088	1.785
H	26.111	4.667	0.191
H	24.496	4.530	-0.019

H	25.372	5.487	-1.014
H	25.296	7.924	-0.344
N	23.154	7.989	3.056
C	23.069	9.200	3.838
C	22.437	8.929	5.188
O	22.671	7.875	5.772
H	23.039	7.040	3.429
H	23.993	9.582	3.974
H	22.518	9.887	3.345
N	21.684	9.902	5.675
C	21.142	9.871	7.017
C	19.750	10.472	7.009
O	19.396	11.322	6.192
C	22.012	10.619	8.042
C	23.436	10.111	8.142
O	22.009	11.978	7.637
H	21.515	10.692	5.008
H	21.074	8.916	7.316
H	21.576	10.549	8.949
H	24.072	10.885	8.213
H	23.538	9.532	8.954
H	23.667	9.573	7.326
H	21.852	12.054	6.643
N	18.952	9.964	7.913
C	17.575	10.360	8.023
C	17.315	10.764	9.462
O	17.701	10.076	10.409
C	16.633	9.223	7.635
C	15.236	9.693	7.349
C	14.321	9.911	8.353
C	14.847	9.957	6.039
C	13.031	10.351	8.073
C	13.581	10.427	5.752
C	12.667	10.622	6.762
O	11.414	11.040	6.440
H	19.398	9.254	8.541
H	17.419	11.159	7.443
H	16.993	8.786	6.818
H	16.600	8.576	8.389
H	14.579	9.752	9.312
H	15.499	9.804	5.297
H	12.370	10.474	8.815
H	13.325	10.628	4.799
H	11.465	11.923	5.971
N	16.640	11.889	9.611
C	16.287	12.378	10.927
C	14.788	12.603	10.957
O	14.306	13.505	10.266
C	17.056	13.659	11.223
C	16.700	14.201	12.587
C	17.778	15.090	13.186
O	17.654	15.403	14.385
O	18.741	15.456	12.440
H	16.393	12.375	8.729
H	16.539	11.707	11.620
H	18.005	13.444	11.223
H	16.800	14.322	10.559
H	15.883	14.746	12.505
H	16.571	13.442	13.205
N	14.073	11.773	11.738
C	12.625	11.852	11.784
C	12.183	12.801	12.893
O	12.716	12.742	13.994
C	11.979	10.515	11.969
O	10.598	10.655	12.289
H	14.638	11.099	12.280
H	12.301	12.230	10.911
H	12.053	9.985	11.126
H	12.425	10.025	12.716
H	10.441	10.335	13.221
N	11.148	13.602	12.608
C	10.594	14.537	13.581
C	9.506	13.880	14.442
O	9.033	14.510	15.377
C	10.060	15.750	12.853
H	10.780	13.497	11.639
H	11.334	14.850	14.192
H	10.724	16.054	12.168
H	9.199	15.519	12.396
H	9.898	16.492	13.505
N	9.118	12.614	14.161
C	8.046	11.940	14.887
C	8.467	10.506	15.188
O	9.405	9.969	14.567
C	6.688	11.896	14.143
C	6.047	13.271	14.044
C	6.803	11.217	12.769
H	9.655	12.173	13.384
H	7.913	12.413	15.758
H	6.071	11.322	14.682
H	6.092	13.594	13.098
H	5.090	13.217	14.331
H	6.535	13.913	14.636
H	6.432	10.294	12.834
H	6.285	11.748	12.103
H	7.764	11.182	12.506
N	7.696	9.892	16.096
C	7.857	8.485	16.417
C	9.028	8.255	17.372
O	9.593	9.200	17.947
H	6.985	10.509	16.539
H	7.011	8.135	16.845
H	8.015	7.959	15.570
N	9.393	6.975	17.493
C	10.443	6.524	18.399
C	11.761	6.562	17.633
O	12.238	5.548	17.117
C	10.098	5.151	18.994
C	11.212	4.581	19.847
H	8.854	6.320	16.875
H	10.512	7.193	19.131
H	9.282	5.252	19.558
H	9.917	4.522	18.241
N	11.074	3.317	20.217
O	12.222	5.244	20.091
H	10.273	2.791	19.930
H	11.774	2.881	20.787
N	12.320	7.773	17.586
C	13.478	8.116	16.790
C	14.169	9.324	17.424
O	13.482	10.292	17.745
C	13.021	8.444	15.386
H	11.824	8.480	18.203
H	14.119	7.350	16.790
H	12.451	7.700	15.033
H	12.488	9.291	15.392
H	13.816	8.560	14.789
N	15.505	9.261	17.544
C	16.335	10.377	17.993
C	17.590	10.485	17.135
O	18.258	9.489	16.888
C	16.744	10.210	19.459
C	17.910	11.091	19.864
C	18.196	11.149	21.359
O	19.080	11.984	21.753
O	17.537	10.367	22.123
H	15.906	8.325	17.283
H	15.812	11.233	17.932
H	15.973	10.459	20.014
H	17.014	9.276	19.589
H	18.729	10.746	19.422
H	17.719	12.019	19.566
N	17.894	11.716	16.693
C	19.088	11.987	15.922
C	18.956	11.319	14.534
O	17.839	11.116	14.026
C	20.352	11.560	16.714
O	21.501	12.275	16.278
H	17.196	12.445	16.956
H	19.159	12.971	15.770
H	20.196	11.748	17.677
H	20.497	10.588	16.569
H	21.412	12.483	15.305
N	20.116	11.033	13.926
C	20.214	10.563	12.566
C	20.357	9.050	12.569
O	21.056	8.481	13.421
C	21.415	11.227	11.884
C	21.249	12.726	11.691
C	22.512	13.363	11.157
N	23.199	14.220	12.115
C	23.692	13.837	13.290
N	23.363	12.671	13.817
N	24.484	14.651	13.963
H	20.956	11.187	14.544
H	19.376	10.803	12.070
H	22.216	11.075	12.449
H	21.534	10.812	10.990
H	20.511	12.896	11.041
H	21.031	13.150	12.568
H	23.135	12.628	10.869
H	22.266	13.908	10.346
H	23.313	15.201	11.865
H	22.753	12.043	13.333
H	23.730	12.405	14.715
H	24.730	15.546	13.581
H	24.846	14.380	14.857
N	19.732	8.445	11.559
C	19.818	7.030	11.250
C	20.437	6.857	9.881
O	20.175	7.667	8.984
C	18.409	6.417	11.249
C	17.751	6.528	12.585
C	17.160	7.698	13.027
C	17.791	5.459	13.456
C	16.643	7.793	14.301
C	17.214	5.526	14.702
C	16.664	6.703	15.146
O	16.136	6.714	16.414
H	19.144	9.115	10.979
H	20.417	6.593	11.925
H	17.865	6.904	10.580
H	18.494	5.459	11.011
H	17.107	8.489	12.412
H	18.251	4.611	13.172
H	16.245	8.665	14.619
H	17.196	4.710	15.293
H	15.238	6.277	16.401
N	21.188	5.762	9.712
C	21.765	5.403	8.447
C	20.630	5.081	7.468
O	19.685	4.374	7.841
C	22.758	4.236	8.580
C	23.164	3.707	7.212
C	23.999	4.664	9.371
H	21.310	5.198	10.589
H	22.263	6.188	8.082
H	22.291	3.492	9.053
H	23.890	3.028	7.318
H	22.374	3.283	6.770
H	23.496	4.463	6.646
H	24.674	3.928	9.340
H	24.377	5.490	8.959
H	23.735	4.843	10.317
N	20.737	5.600	6.234
C	19.881	5.105	5.154
C	20.718	4.670	3.975
O	21.843	5.140	3.738
C	18.856	6.155	4.738
C	19.330	7.347	3.915
C	19.396	7.068	2.398
C	18.385	8.486	4.212
H	21.438	6.337	6.126
H	19.366	4.313	5.497
H	18.142	5.688	4.201
H	18.435	6.519	5.575
H	20.251	7.623	4.231
H	18.486	6.820	2.074
H	19.713	7.890	1.931
H	20.032	6.317	2.235
H	18.408	8.701	5.192
H	18.656	9.298	3.691
H	17.450	8.229	3.956
N	20.160	3.710	3.256
C	20.732	3.301	2.001

C 19.590 3.034 1.037
O 18.535 2.521 1.446
C 21.618 2.078 2.213
C 20.850 0.868 2.655
O 22.290 1.778 0.996
H 19.304 3.296 3.669
H 21.285 4.046 1.633
H 22.320 2.295 2.921
H 20.152 0.627 1.971
H 21.468 0.083 2.772
H 20.389 1.046 3.532
H 22.339 2.594 0.408
N 19.844 3.301 -0.234
C 18.883 2.957 -1.275
C 19.507 3.019 -2.667
O 20.725 2.943 -2.806
H 20.752 3.758 -0.419
H 18.529 2.025 -1.113
H 18.100 3.595 -1.235
N 18.637 3.020 -3.681
C 18.998 2.955 -5.085
C 18.108 3.925 -5.859
O 16.953 4.181 -5.492
C 18.792 1.548 -5.664
C 19.488 0.389 -4.962
C 21.005 0.458 -5.143
N 21.360 0.209 -6.540
C 22.545 0.530 -7.071
N 23.525 0.971 -6.316
N 22.742 0.380 -8.357
H 17.627 3.075 -3.358
H 19.948 3.239 -5.195
H 17.800 1.357 -5.665
H 19.106 1.560 -6.624
H 19.293 0.424 -3.987
H 19.172 -0.472 -5.348
H 21.303 1.363 -4.882
H 21.412 -0.237 -4.570
H 20.681 -0.225 -7.133
H 23.415 1.058 -5.324
H 24.407 1.228 -6.733
H 22.017 0.006 -8.944
H 23.621 0.635 -8.771
N 18.628 4.436 -6.979
C 17.847 5.259 -7.892
C 18.239 4.888 -9.315
O 19.279 4.258 -9.530
C 18.020 6.756 -7.620
C 19.337 7.344 -8.112
C 20.539 7.090 -7.459
C 19.391 8.124 -9.266
C 21.754 7.593 -7.937
C 20.583 8.667 -9.733
C 21.774 8.390 -9.080
O 22.981 8.899 -9.585
H 19.629 4.195 -7.137
H 16.882 5.042 -7.767
H 17.277 7.245 -8.073
H 17.969 6.905 -6.634
H 20.542 6.534 -6.623
H 18.544 8.298 -9.777
H 22.606 7.380 -7.458
H 20.581 9.259 -10.541
H 23.732 8.401 -9.166
N 17.369 5.264 -10.259
C 17.572 5.029 -11.688
C 18.564 6.066 -12.209
O 18.220 7.236 -12.296
C 16.268 5.166 -12.465
C 16.409 4.942 -13.975
O 17.544 4.754 -14.449
O 15.397 4.974 -14.651
H 16.517 5.748 -9.879
H 18.004 4.135 -11.784
H 15.620 4.494 -12.110
H 15.910 6.087 -12.318
N 19.802 5.648 -12.503
C 20.835 6.614 -12.855
C 20.798 6.966 -14.347
O 21.588 7.791 -14.782
C 22.208 6.143 -12.416
O 22.424 4.768 -12.740
H 19.946 4.633 -12.458
H 20.664 7.462 -12.337
H 22.906 6.688 -12.876
H 22.293 6.251 -11.427
H 22.945 4.337 -12.007
N 19.887 6.381 -15.129
C 19.705 6.752 -16.539
C 18.213 6.838 -16.850
O 17.635 5.933 -17.466
C 20.391 5.747 -17.441
H 19.320 5.646 -14.658
H 20.117 7.650 -16.690
H 20.616 4.923 -16.920
H 19.783 5.503 -18.198
H 21.233 6.144 -17.810
N 17.512 7.866 -16.327
C 16.055 7.932 -16.467
C 15.584 8.175 -17.896
O 16.396 8.336 -18.806
C 15.658 9.079 -15.534
C 16.895 9.919 -15.381
C 18.064 8.965 -15.516
H 15.624 7.089 -16.089
H 14.909 9.620 -15.933
H 15.356 8.725 -14.643
H 16.930 10.626 -16.093
H 16.905 10.368 -14.482
H 18.832 9.398 -15.984
H 18.362 8.629 -14.622
N 14.268 8.143 -18.094
C 13.687 8.488 -19.383
C 13.718 10.010 -19.578
O 13.865 10.776 -18.626
C 12.293 7.916 -19.495
H 13.709 7.858 -17.269
H 14.232 8.066 -20.112
H 11.682 8.383 -18.853
H 11.948 8.042 -20.427
H 12.309 6.938 -19.281
N 13.596 10.449 -20.841
C 13.764 11.857 -21.193
C 12.470 12.442 -21.752
O 12.498 13.434 -22.476
C 14.916 12.034 -22.191
C 16.249 12.195 -21.485
O 14.897 10.904 -23.076
H 13.374 9.707 -21.533
H 14.008 12.368 -20.359
H 14.753 12.869 -22.740
H 16.217 12.986 -20.870
H 16.454 11.372 -20.948
H 16.980 12.334 -22.159
H 15.228 10.084 -22.600
N 11.340 11.835 -21.384
C 10.034 12.224 -21.879
C 9.320 13.071 -20.828
O 8.095 13.182 -20.884
C 9.207 10.987 -22.240
C 8.962 10.015 -21.091
O 9.502 10.231 -19.981
O 8.213 9.053 -21.308
H 11.485 11.050 -20.703
H 10.176 12.812 -22.679
H 8.318 11.300 -22.583
H 9.686 10.498 -22.972
N 10.085 13.620 -19.865
C 9.543 14.374 -18.747
C 9.090 13.504 -17.562
O 8.499 14.017 -16.620
H 11.111 13.451 -20.002
H 10.242 15.037 -18.414
H 8.749 14.928 -19.064
N 9.380 12.201 -17.600
C 9.072 11.297 -16.497
C 9.964 11.596 -15.301
O 11.138 11.949 -15.444
C 9.241 9.866 -16.900
O 8.197 9.458 -17.764
H 9.843 11.890 -18.482
H 8.119 11.455 -16.221
H 10.110 9.752 -17.377
H 9.224 9.285 -16.087
H 8.450 9.657 -18.711
N 9.445 11.302 -14.099
C 10.263 11.336 -12.904
C 11.311 10.213 -12.859
O 11.333 9.283 -13.674
H 8.441 11.059 -14.112
H 10.743 12.229 -12.842
H 9.671 11.260 -12.081
N 12.189 10.307 -11.849
C 13.280 9.368 -11.661
C 12.935 8.467 -10.475
O 12.899 8.961 -9.357
C 14.575 10.123 -11.352
C 15.751 9.222 -11.058
O 14.768 10.942 -12.517
H 12.012 11.119 -11.212
H 13.359 8.796 -12.474
H 14.428 10.722 -10.568
H 15.641 8.345 -11.535
H 16.603 9.655 -11.365
H 15.814 9.048 -10.071
H 15.741 11.173 -12.623
N 12.713 7.185 -10.737
C 12.364 6.233 -9.678
C 13.511 6.071 -8.692
O 14.662 5.957 -9.087
C 12.004 4.919 -10.299
H 12.806 6.917 -11.734
H 11.560 6.583 -9.184
H 12.588 4.740 -11.096
H 12.127 4.177 -9.635
H 11.045 4.929 -10.599
N 13.159 5.929 -7.408
C 14.119 5.673 -6.367
C 13.432 5.060 -5.152
O 12.210 4.985 -5.085
C 14.842 6.980 -6.033
C 13.977 8.213 -5.780
C 13.245 8.154 -4.466
C 14.852 9.466 -5.817
H 12.130 6.022 -7.241
H 14.804 5.016 -6.702
H 15.387 6.824 -5.208
H 15.451 7.195 -6.796
H 13.310 8.299 -6.535
H 13.902 8.086 -3.711
H 12.692 8.982 -4.344
H 12.642 7.354 -4.445
H 15.286 9.540 -6.715
H 14.285 10.271 -5.651
H 15.555 9.400 -5.109
N 14.252 4.627 -4.202
C 13.770 4.166 -2.922
C 14.921 4.041 -1.949
O 16.080 3.986 -2.367
H 15.263 4.650 -4.465
H 13.095 4.817 -2.558
H 13.324 3.271 -3.028
N 14.580 3.986 -0.671
C 15.584 3.829 0.360
C 14.936 3.223 1.599
O 13.705 3.192 1.711
C 16.288 5.158 0.712
C 15.417 6.179 1.382
C 15.330 6.422 2.720
C 14.641 7.216 0.754
C 14.048 7.973 1.773
C 14.360 7.559 -0.571
N 14.466 7.451 2.954
C 13.192 9.062 1.528
C 13.538 8.638 -0.821

C	12.937	9.357	0.212
H	13.565	4.063	-0.483
H	16.270	3.178	0.031
H	17.046	4.960	1.330
H	16.632	5.566	-0.130
H	15.827	5.918	3.427
H	14.182	7.776	3.865
H	14.747	7.033	-1.328
H	12.802	9.578	2.284
H	13.370	8.908	-1.771
H	12.310	10.102	-0.017
N	15.813	2.760	2.498
C	15.452	2.118	3.747
C	16.209	2.775	4.890
O	17.395	3.046	4.736
C	15.859	0.648	3.785
C	15.459	-0.042	5.080
O	15.252	0.002	2.657
H	16.821	2.911	2.206
H	14.471	2.244	3.904
H	16.865	0.576	3.685
H	14.939	-0.875	4.875
H	16.278	-0.287	5.603
H	14.889	0.573	5.632
H	15.382	-0.994	2.713
N	15.485	2.952	5.994
C	16.023	3.218	7.321
C	15.598	2.099	8.264
O	14.401	1.804	8.364
C	15.552	4.585	7.846
C	15.995	4.744	9.297
C	16.025	5.722	6.942
H	14.438	2.880	5.817
H	17.021	3.212	7.264
H	14.563	4.587	7.844
H	15.190	4.778	9.888
H	16.568	3.967	9.557
H	16.516	5.591	9.395
H	16.259	6.513	7.506
H	16.829	5.422	6.431
H	15.291	5.964	6.309
N	16.574	1.471	8.954
C	16.280	0.692	10.160
C	16.447	1.611	11.377
O	17.444	2.313	11.462
C	17.172	-0.510	10.273
H	17.523	1.589	8.561
H	15.335	0.382	10.123
H	17.498	-0.783	9.364
H	17.964	-0.298	10.851
H	16.669	-1.276	10.680
N	15.461	1.616	12.273
C	15.326	2.634	13.309
C	16.140	2.251	14.542
O	15.586	2.237	15.658
C	13.850	2.858	13.636
C	13.131	3.443	12.462
C	12.266	2.801	11.630
C	13.307	4.768	11.938
C	12.461	4.873	10.809
C	14.032	5.896	12.339
N	11.834	3.654	10.658
C	12.335	6.069	10.084
C	13.908	7.069	11.621
C	13.076	7.150	10.507
H	14.787	0.818	12.168
H	15.707	3.491	12.949
H	13.432	1.994	13.840
H	13.778	3.497	14.377
H	11.988	1.844	11.719
H	11.167	3.441	9.942
H	14.634	5.856	13.139
H	11.725	6.117	9.291
H	14.428	7.872	11.910
H	13.018	8.014	10.005
N	17.403	1.914	14.281
C	18.420	1.686	15.312
C	19.585	2.635	15.046
O	20.109	2.732	13.944
C	18.909	0.223	15.340
C	20.077	-0.055	16.295
C	20.704	-1.431	16.129
C	21.886	-1.681	17.046
N	23.040	-0.837	16.663
H	17.615	1.824	13.253
H	18.029	1.896	16.203
H	18.140	-0.356	15.613
H	19.198	-0.024	14.414
H	20.788	0.647	16.145
H	19.745	0.040	17.245
H	20.004	-2.128	16.316
H	21.012	-1.532	15.178
H	21.618	-1.470	17.987
H	22.147	-2.646	16.986
H	23.052	-0.716	15.669
H	22.963	0.058	17.105
H	23.890	-1.278	16.954
N	20.039	3.278	16.133
C	21.238	4.071	16.108
C	21.826	4.035	17.527
O	21.501	3.147	18.311
C	20.937	5.484	15.597
C	20.121	6.344	16.542
H	19.444	3.145	16.980
H	21.897	3.617	15.520
H	21.813	5.947	15.422
H	20.437	5.403	14.728
N	19.790	7.558	16.103
O	19.709	5.916	17.629
H	20.077	7.850	15.190
H	19.257	8.171	16.683
N	22.629	5.048	17.859
C	23.349	5.043	19.126
C	22.556	5.701	20.235
O	23.107	5.929	21.316
C	24.722	5.690	18.979
C	25.633	4.843	18.115
H	22.690	5.806	17.162
H	23.483	4.075	19.388
H	24.611	6.575	18.549
H	25.128	5.779	19.878
N	26.686	5.453	17.600
O	25.397	3.645	17.928
H	26.852	6.423	17.791
H	27.325	4.949	17.015
N	21.269	5.941	19.988
C	20.372	6.502	20.960
C	19.199	5.588	21.258
O	18.754	5.548	22.410
C	19.902	7.865	20.471
C	21.026	8.857	20.460
C	21.453	9.471	21.631
C	21.739	9.094	19.297
C	22.513	10.367	21.622
C	22.790	9.986	19.265
C	23.162	10.633	20.427
O	24.232	11.455	20.328
H	20.974	5.680	19.012
H	20.882	6.646	21.827
H	19.557	7.765	19.550
H	19.199	8.192	21.085
H	20.991	9.266	22.495
H	21.484	8.605	18.459
H	22.804	10.815	22.465
H	23.279	10.164	18.412
H	23.930	12.379	20.084
N	18.657	4.927	20.217
C	17.361	4.279	20.313
C	17.400	3.025	19.450
O	18.173	2.911	18.500
C	16.203	5.102	19.727
C	16.086	6.549	20.154
C	15.157	6.772	21.320
N	15.057	8.213	21.498
C	13.953	8.928	21.305
N	14.021	10.250	21.342
N	12.802	8.320	21.051
H	19.244	4.928	19.361
H	17.196	4.012	21.258
H	16.291	5.079	18.720
H	15.341	4.633	19.970
H	17.000	6.895	20.409
H	15.752	7.099	19.377
H	14.280	6.407	21.096
H	15.554	6.369	22.117
H	15.889	8.710	21.790
H	14.879	10.729	21.490
H	13.175	10.793	21.214
H	12.776	7.318	20.987
H	11.968	8.851	20.923
N	16.546	2.085	19.788
C	16.219	1.020	18.844
C	14.722	0.782	18.912
O	14.247	0.166	19.866
C	17.054	-0.244	19.073
C	16.853	-1.298	18.009
H	16.144	2.157	20.733
H	16.422	1.363	17.931
H	18.019	0.018	19.088
H	16.798	-0.630	19.960
N	17.566	-2.421	18.113
O	16.061	-1.065	17.095
H	18.197	-2.523	18.889
H	17.475	-3.140	17.437
N	13.988	1.161	17.842
C	12.556	0.960	17.827
C	12.158	-0.370	17.188
O	10.961	-0.591	17.009
C	11.880	2.105	17.112
H	14.532	1.586	17.079
H	12.210	0.968	18.782
H	11.741	2.867	17.747
H	12.451	2.414	16.349
H	10.991	1.808	16.757
N	13.127	-1.217	16.813
C	12.852	-2.546	16.291
C	11.904	-2.477	15.086
O	10.908	-3.209	14.985
C	12.277	-3.463	17.377
C	13.203	-3.593	18.548
H	14.092	-0.834	16.930
H	13.716	-2.958	15.981
H	11.412	-3.085	17.699
H	12.126	-4.371	16.990
C	13.010	-3.292	19.859
N	14.521	-3.988	18.425
C	15.089	-3.964	19.646
N	14.193	-3.541	20.527
H	14.978	-4.244	17.587
H	12.166	-2.954	20.268
H	16.026	-4.225	19.843
N	12.231	-1.590	14.168
C	11.351	-1.334	13.045
C	12.210	-0.804	11.895
O	13.376	-0.432	12.101
C	10.238	-0.389	13.446
O	10.787	0.837	13.890
H	13.135	-1.111	14.313
H	10.951	-2.195	12.751
H	9.656	-0.217	12.661
H	9.717	-0.795	14.186
H	11.265	0.695	14.756
N	11.640	-0.821	10.684
C	12.319	-0.307	9.498
C	11.261	0.308	8.575
O	10.148	-0.201	8.472
C	13.108	-1.400	8.805
H	10.683	-1.227	10.666
H	12.955	0.409	9.771
H	13.417	-2.071	9.479
H	12.530	-1.854	8.126
H	13.905	-1.002	8.347
N	11.608	1.406	7.907
C	10.751	2.048	6.922
C	11.433	2.007	5.557

O	12.649	2.197	5.436
C	10.484	3.516	7.289
C	9.619	4.240	6.277
O	9.832	3.527	8.553
H	12.561	1.771	8.156
H	9.892	1.537	6.859
H	11.371	3.997	7.379
H	8.977	4.851	6.748
H	10.195	4.786	5.662
H	9.098	3.576	5.733
H	9.235	2.721	8.652
N	10.657	1.706	4.511
C	11.130	1.869	3.148
C	10.248	2.914	2.476
O	9.034	2.927	2.660
C	11.096	0.568	2.334
C	11.978	-0.495	2.913
O	9.716	0.182	2.236
H	9.714	1.355	4.759
H	12.064	2.212	3.169
H	11.441	0.752	1.403
H	12.082	-0.365	3.905
H	11.583	-1.407	2.751
H	12.890	-0.464	2.489
H	9.250	0.328	3.114
N	10.887	3.819	1.722
C	10.229	4.802	0.896
C	10.488	4.458	-0.566
O	11.631	4.203	-0.953
C	10.800	6.215	1.150
C	10.557	6.852	2.477
C	9.652	7.838	2.722
C	11.213	6.612	3.734
C	10.670	7.531	4.663
C	12.228	5.755	4.160
N	9.725	8.252	4.021
C	11.082	7.611	5.997
C	12.675	5.871	5.468
C	12.102	6.768	6.364
H	11.940	3.742	1.792
H	9.244	4.733	1.049
H	11.803	6.176	1.016
H	10.423	6.837	0.445
H	9.015	8.212	2.040
H	9.163	8.978	4.431
H	12.624	5.079	3.551
H	10.652	8.246	6.627
H	13.433	5.287	5.767
H	12.448	6.797	7.308
N	9.406	4.456	-1.338
C	9.431	4.205	-2.755
C	8.821	5.427	-3.434
O	7.705	5.866	-3.116
C	8.686	2.919	-3.025
O	8.657	2.634	-4.410
H	8.514	4.659	-0.807
H	10.376	4.135	-3.053
H	9.142	2.169	-2.552
H	7.748	3.008	-2.696
H	8.527	1.652	-4.547
N	9.531	5.974	-4.426
C	9.012	7.165	-5.031
C	9.756	7.627	-6.294
O	10.361	6.830	-6.997
H	10.390	5.500	-4.675
H	8.025	7.031	-5.273
H	9.024	7.935	-4.354
N	9.539	8.913	-6.585
C	10.191	9.517	-7.736
C	10.611	10.947	-7.418
O	9.910	11.698	-6.752
C	9.343	9.342	-8.999
C	7.972	9.977	-9.028
C	7.253	9.646	-10.320
H	8.901	9.407	-5.951
H	11.023	8.991	-7.936
H	9.875	9.707	-9.791
H	9.229	8.341	-9.168
H	7.437	9.628	-8.268
H	8.070	10.964	-8.963
N	5.976	9.316	-10.201
O	7.830	9.700	-11.413
H	5.417	9.092	-11.028
H	5.554	9.287	-9.276
N	11.797	11.251	-7.964
C	12.353	12.588	-8.014
C	11.816	13.287	-9.257
O	11.912	12.730	-10.337
C	13.868	12.464	-8.067
C	14.562	13.741	-8.464
C	14.781	14.742	-7.547
C	14.935	13.943	-9.781
C	15.397	15.934	-7.914
C	15.587	15.107	-10.157
C	15.813	16.102	-9.222
O	16.478	17.266	-9.615
H	12.297	10.408	-8.361
H	12.042	13.096	-7.213
H	14.194	12.199	-7.163
H	14.104	11.761	-8.734
H	14.495	14.620	-6.591
H	14.737	13.248	-10.466
H	15.537	16.656	-7.239
H	15.892	15.229	-11.103
H	17.333	17.328	-9.112
N	11.285	14.498	-9.060
C	10.818	15.382	-10.112
C	11.625	16.684	-10.058
O	11.529	17.408	-9.067
C	9.321	15.673	-9.923
C	8.793	16.524	-11.076
C	8.513	14.386	-9.728
H	11.233	14.763	-8.034
H	10.996	14.951	-10.994
H	9.218	16.214	-9.095
H	9.185	16.198	-11.936
H	7.798	16.450	-11.113
H	9.052	17.477	-10.930
H	9.034	13.617	-10.090
H	8.344	14.254	-8.753
H	7.645	14.470	-10.215
N	12.399	16.965	-11.115
C	13.312	18.105	-11.153
C	12.604	19.434	-11.440
O	11.384	19.505	-11.449
H	12.290	16.295	-11.911
H	13.798	18.176	-10.265
H	14.017	17.948	-11.866
N	13.395	20.497	-11.649
C	12.873	21.784	-12.078
C	12.859	22.802	-10.942
O	13.501	22.600	-9.909
H	14.407	20.314	-11.476
H	13.438	22.143	-12.836
H	11.932	21.668	-12.428
N	12.088	23.878	-11.158
C	12.076	25.053	-10.296
C	11.968	24.643	-8.830
O	12.865	24.920	-8.037
C	10.921	25.947	-10.693
H	11.486	23.795	-12.017
H	12.936	25.560	-10.424
H	10.493	26.330	-9.873
H	11.254	26.694	-11.270
H	10.241	25.416	-11.201
N	10.851	23.974	-8.523
C	10.506	23.487	-7.199
C	10.661	21.955	-7.183
O	9.674	21.236	-7.282
C	9.053	23.866	-6.875
C	8.729	25.351	-6.980
C	9.236	26.197	-5.826
O	10.189	25.773	-5.157
O	8.648	27.263	-5.579
H	10.217	23.826	-9.363
H	11.113	23.884	-6.515
H	8.472	23.388	-7.516
H	8.872	23.586	-5.945
H	9.146	25.699	-7.809
H	7.743	25.451	-7.011
N	11.900	21.458	-7.072
C	12.138	20.023	-7.150
C	11.453	19.329	-5.968
O	11.315	19.883	-4.876
C	13.614	19.717	-7.206
H	12.640	22.162	-6.933
H	11.741	19.669	-7.998
H	13.754	18.733	-7.339
H	14.037	20.213	-7.967
H	14.054	19.996	-6.350
N	11.039	18.081	-6.195
C	10.280	17.360	-5.187
C	10.651	15.887	-5.295
O	10.997	15.409	-6.370
C	8.771	17.588	-5.368
C	8.195	18.670	-4.475
C	6.779	19.084	-4.823
N	6.226	20.006	-3.842
C	6.645	21.248	-3.632
N	6.055	21.984	-2.708
N	7.652	21.751	-4.331
H	11.298	17.693	-7.114
H	10.538	17.690	-4.285
H	8.611	17.847	-6.317
H	8.304	16.732	-5.164
H	8.199	18.348	-3.517
H	8.780	19.492	-4.528
H	6.795	19.523	-5.718
H	6.216	18.261	-4.857
H	5.455	19.673	-3.269
H	5.314	21.632	-2.145
H	6.363	22.940	-2.564
H	8.111	21.171	-5.014
H	7.952	22.688	-4.186
N	10.589	15.183	-4.149
C	10.602	13.722	-4.142
C	9.281	13.283	-3.499
O	9.002	13.605	-2.357
C	11.833	13.139	-3.415
C	13.154	13.568	-4.072
C	11.716	11.616	-3.319
C	14.401	13.193	-3.297
H	10.531	15.763	-3.293
H	10.562	13.436	-5.087
H	11.829	13.488	-2.477
H	13.200	13.153	-4.983
H	13.134	14.564	-4.185
H	12.470	11.262	-2.771
H	10.848	11.379	-2.889
H	11.755	11.225	-4.236
H	14.440	12.200	-3.193
H	15.207	13.508	-3.795
H	14.372	13.624	-2.397
N	8.449	12.609	-4.294
C	7.128	12.206	-3.879
C	7.178	10.702	-3.609
O	7.622	9.941	-4.465
C	6.097	12.468	-4.974
C	5.906	13.928	-5.295
H	8.845	12.404	-5.243
H	6.933	12.646	-3.009
H	6.394	11.992	-5.805
H	5.219	12.087	-4.674
N	5.938	14.231	-6.583
O	5.807	14.756	-4.389
H	6.073	13.528	-7.277
H	5.824	15.190	-6.873
N	6.765	10.297	-2.416
C	7.012	8.928	-1.981
C	5.773	8.352	-1.296
O	4.914	9.080	-0.810
C	8.170	8.764	-0.979
C	9.465	9.430	-1.409
O	7.795	9.286	0.293
H	6.275	11.003	-1.850

H	7.214	8.365	-2.789
H	8.372	7.768	-0.872
H	10.000	9.690	-0.602
H	10.004	8.797	-1.969
H	9.264	10.252	-1.947
H	7.392	10.203	0.194
N	5.763	7.016	-1.286
C	4.935	6.279	-0.355
C	5.875	5.440	0.461
O	6.989	5.119	0.021
C	3.934	5.443	-1.157
C	2.947	6.364	-1.879
C	1.752	5.703	-2.509
H	6.386	6.574	-1.986
H	4.493	6.930	0.249
H	4.419	4.922	-1.834
H	3.424	4.871	-0.540
H	2.620	7.057	-1.210
H	3.459	6.867	-2.598
N	1.763	5.567	-3.812
O	0.815	5.307	-1.828
H	0.995	5.115	-4.313
H	2.564	5.922	-4.345
N	5.445	5.098	1.667
C	6.330	4.379	2.530
C	5.610	3.227	3.248
O	4.389	3.287	3.407
C	7.039	5.304	3.515
C	6.216	6.190	4.363
C	6.102	7.536	4.217
C	5.491	5.835	5.548
C	4.927	7.026	6.037
C	5.279	4.646	6.254
N	5.249	8.019	5.148
C	4.088	7.061	7.150
C	4.443	4.674	7.353
C	3.877	5.861	7.811
H	4.489	5.381	1.897
H	7.059	3.961	1.968
H	7.604	4.732	4.136
H	7.678	5.895	2.992
H	6.579	8.080	3.526
H	4.895	8.962	5.194
H	5.718	3.798	5.973
H	3.665	7.912	7.450
H	4.243	3.816	7.829
H	3.308	5.848	8.631
N	6.423	2.222	3.619
C	5.970	1.120	4.450
C	6.860	1.038	5.684
O	8.085	0.914	5.552
C	6.072	-0.186	3.657
C	5.161	-0.359	2.432
C	5.516	-1.634	1.644
C	3.717	-0.458	2.824
H	7.396	2.310	3.257
H	5.034	1.302	4.747
H	7.019	-0.274	3.334
H	5.869	-0.941	4.284
H	5.267	0.434	1.832
H	5.406	-2.426	2.240
H	4.906	-1.711	0.859
H	6.461	-1.571	1.334
H	3.432	0.376	3.307
H	3.144	-0.570	2.006
H	3.577	-1.247	3.429
N	6.241	0.960	6.859
C	6.971	0.906	8.125
C	6.591	-0.406	8.796
O	5.411	-0.581	9.107
C	6.576	2.101	8.993
C	7.328	2.287	10.322
C	6.879	3.581	11.010
C	7.127	1.107	11.224
H	5.201	0.940	6.800
H	7.942	0.895	7.933
H	6.720	2.933	8.456
H	5.605	2.012	9.216
H	8.306	2.335	10.136
H	5.898	3.533	11.192
H	7.376	3.686	11.869
H	7.073	4.356	10.412
H	7.468	0.272	10.782
H	7.624	1.242	12.087
H	6.151	0.990	11.428
N	7.550	-1.333	8.836
C	7.328	-2.612	9.509
C	7.959	-2.613	10.903
O	9.147	-2.290	11.083
C	7.900	-3.789	8.709
C	7.582	-5.145	9.326
O	7.323	-3.740	7.403
H	8.431	-1.075	8.367
H	6.337	-2.751	9.613
H	8.902	-3.670	8.621
H	6.788	-5.063	9.934
H	7.375	-5.807	8.602
H	8.368	-5.470	9.855
H	7.883	-3.170	6.792
N	7.193	-3.132	11.861
C	7.665	-3.311	13.227
C	7.812	-4.807	13.479
O	6.983	-5.594	13.016
C	6.731	-2.648	14.262
C	6.524	-1.152	14.011
C	7.250	-2.891	15.672
C	5.142	-0.814	13.501
H	6.233	-3.398	11.549
H	8.581	-2.926	13.262
H	5.836	-3.098	14.199
H	6.684	-0.664	14.865
H	7.197	-0.853	13.338
H	6.591	-2.541	16.335
H	7.378	-3.870	15.816
H	8.123	-2.421	15.792
H	4.460	-1.105	14.173
H	5.072	0.172	13.359
H	4.982	-1.288	12.636
N	8.926	-5.205	14.113
C	9.121	-6.588	14.516
C	7.980	-7.041	15.436
O	7.583	-6.288	16.314
H	9.616	-4.450	14.282
H	9.141	-7.179	13.706
H	9.992	-6.680	15.006
N	7.343	-8.171	15.108
C	6.203	-8.657	15.887
C	6.342	-10.171	16.043
O	7.085	-10.821	15.320
C	4.817	-8.348	15.265
C	4.554	-6.878	15.005
O	4.655	-9.093	14.056
H	7.719	-8.657	14.276
H	6.235	-8.242	16.791
H	4.095	-8.640	15.923
H	5.396	-6.350	15.144
H	4.238	-6.747	14.062
H	3.850	-6.539	15.635
H	4.325	-10.022	14.258
N	5.603	-10.721	17.019
C	5.266	-12.137	17.051
C	4.367	-12.481	15.862
O	3.750	-11.607	15.229
C	4.543	-12.485	18.365
C	5.306	-12.022	19.590
O	3.285	-11.819	18.311
H	5.293	-10.041	17.750
H	6.109	-12.671	16.971
H	4.380	-13.479	18.393
H	4.708	-12.048	20.397
H	6.095	-12.622	19.750
H	5.632	-11.083	19.456
H	3.188	-11.316	17.444
N	4.249	-13.785	15.595
C	3.492	-14.266	14.446
C	2.024	-13.918	14.666
O	1.348	-13.507	13.717
C	3.847	-15.745	14.180
C	5.352	-15.891	13.892
C	5.950	-17.243	13.535
O	6.728	-17.767	14.355
O	5.725	-17.725	12.402
H	4.736	-14.410	16.265
H	3.819	-13.825	13.615
H	3.653	-16.245	14.984
H	3.368	-16.028	13.391
H	5.572	-15.258	13.128
H	5.849	-15.553	14.710
N	1.582	-13.949	15.940
C	0.216	-13.599	16.313
C	-0.142	-12.140	15.981
O	-1.305	-11.838	15.687
C	0.038	-13.839	17.792
H	2.307	-14.245	16.630
H	-0.426	-14.202	15.810
H	-0.198	-12.981	18.250
H	-0.696	-14.504	17.943
H	0.888	-14.196	18.184
N	0.831	-11.227	16.097
C	0.580	-9.801	15.897
C	0.994	-9.310	14.500
O	0.978	-8.113	14.273
C	1.257	-8.959	16.977
C	0.620	-9.182	18.333
H	1.763	-11.617	16.336
H	-0.420	-9.660	15.969
H	2.212	-9.220	17.026
H	1.165	-8.002	16.735
N	1.405	-9.117	19.394
O	-0.570	-9.459	18.410
H	2.384	-8.918	19.269
H	1.039	-9.261	20.308
N	1.368	-10.206	13.585
C	1.988	-9.821	12.316
C	0.997	-9.075	11.414
O	1.408	-8.304	10.545
C	2.558	-11.037	11.616
H	1.182	-11.197	13.850
H	2.766	-9.209	12.508
H	1.830	-11.704	11.451
H	2.961	-10.765	10.741
H	3.264	-11.456	12.188
N	-0.312	-9.284	11.615
C	-1.342	-8.541	10.886
C	-1.278	-7.045	11.189
O	-1.745	-6.222	10.398
C	-2.759	-9.119	11.157
C	-3.242	-8.878	12.553
C	-3.005	-9.649	13.661
C	-3.929	-7.711	13.027
C	-4.142	-7.892	14.418
C	-4.488	-6.594	12.403
N	-3.564	-9.073	14.772
C	-4.832	-6.968	15.191
C	-5.146	-5.661	13.170
C	-5.333	-5.859	14.544
H	-0.527	-10.011	12.326
H	-1.164	-8.659	9.900
H	-3.396	-8.684	10.538
H	-2.729	-10.098	11.012
H	-2.496	-10.510	13.658
H	-3.548	-9.459	15.696
H	-4.411	-6.471	11.412
H	-4.955	-7.109	16.174
H	-5.495	-4.833	12.741
H	-5.845	-5.174	15.065
N	-0.766	-6.653	12.348
C	-0.701	-5.237	12.678
C	0.760	-4.764	12.692
O	1.109	-3.775	13.314
C	-1.478	-4.955	13.960
C	-0.941	-5.613	15.223
C	-1.711	-5.197	16.476
C	-1.484	-6.153	17.630
N	-2.391	-5.913	18.782

H -0.434 -7.407 12.966
H -1.197 -4.713 11.981
H -1.489 -3.960 14.107
H -2.427 -5.266 13.825
H -1.004 -6.610 15.126
H 0.023 -5.359 15.341
H -1.407 -4.287 16.748
H -2.684 -5.179 16.260
H -1.615 -7.100 17.299
H -0.526 -6.067 17.942
H -1.871 -5.978 19.634
H -2.793 -5.002 18.703
H -3.117 -6.600 18.782
N 1.582 -5.375 11.851
C 3.010 -5.077 11.811
C 3.359 -3.933 10.851
O 4.462 -3.424 10.974
C 3.805 -6.308 11.437
O 3.533 -6.747 10.097
H 1.129 -6.076 11.229
H 3.300 -4.795 12.741
H 4.783 -6.109 11.506
H 3.575 -7.057 12.061
H 2.728 -7.333 10.105
N 2.488 -3.545 9.912
C 2.959 -2.679 8.826
C 2.076 -1.450 8.671
O 0.890 -1.565 8.368
C 3.109 -3.399 7.480
C 3.820 -2.549 6.423
O 3.896 -4.573 7.658
H 1.536 -3.884 10.013
H 3.895 -2.374 9.048
H 2.183 -3.680 7.152
H 4.781 -2.823 6.364
H 3.382 -2.684 5.534
H 3.764 -1.583 6.677
H 3.611 -5.068 8.488
N 2.674 -0.268 8.875
C 1.993 0.972 8.550
C 2.348 1.400 7.131
O 3.477 1.165 6.682
C 2.444 2.055 9.540
C 2.011 1.914 10.995
C 2.819 2.865 11.879
C 0.539 2.218 11.137
H 3.625 -0.325 9.269
H 1.003 0.821 8.595
H 3.448 2.078 9.526
H 2.094 2.933 9.206
H 2.168 0.975 11.295
H 2.665 3.805 11.581
H 2.526 2.761 12.829
H 3.790 2.643 11.802
H 0.004 1.581 10.577
H 0.262 2.123 12.096
H 0.355 3.156 10.834
N 1.449 2.194 6.541
C 1.633 2.756 5.217
C 1.362 4.236 5.295
O 0.454 4.724 5.984
C 0.758 2.081 4.154
C -0.744 2.181 4.443
C 1.069 2.620 2.779
H 0.596 2.372 7.126
H 2.585 2.624 4.950
H 0.982 1.100 4.146
H -1.102 1.271 4.645
H -0.888 2.781 5.229
H -1.208 2.557 3.643
H 0.684 2.009 2.084
H 0.670 3.533 2.678
H 2.062 2.677 2.657
N 2.150 4.981 4.529
C 1.910 6.405 4.439
C 2.556 7.023 3.210
O 2.951 6.320 2.279
H 2.899 4.481 4.039
H 0.911 6.579 4.411
H 2.270 6.864 5.268
N 2.558 8.365 3.172
C 3.141 9.056 2.037
C 3.863 10.305 2.538
O 3.381 10.948 3.455
C 2.080 9.417 0.998
C 0.845 9.987 1.583
C 0.366 11.249 1.681
N -0.137 9.164 2.084
C -1.169 9.915 2.502
N -0.879 11.191 2.276
H 2.129 8.825 3.982
H 3.815 8.458 1.606
H 2.472 10.087 0.355
H 1.840 8.588 0.480
H -0.098 8.171 2.134
H 0.829 12.083 1.378
H -2.004 9.568 2.907
N 5.022 10.601 1.970
C 5.802 11.773 2.344
C 6.161 12.528 1.058
O 6.552 11.895 0.084
C 7.081 11.376 3.081
C 6.953 10.887 4.530
O 5.864 11.000 5.131
O 7.941 10.310 5.034
H 5.329 9.923 1.234
H 5.210 12.372 2.874
H 7.533 10.643 2.545
H 7.703 12.177 3.078
N 6.081 13.866 1.081
C 6.605 14.657 -0.018
C 7.783 15.497 0.500
O 7.618 16.148 1.522
C 5.540 15.596 -0.606
C 6.063 16.316 -1.825
O 4.402 14.841 -1.010
H 5.629 14.260 1.919
H 6.951 14.044 -0.725
H 5.248 16.255 0.119
H 6.993 16.001 -2.042
H 5.468 16.136 -2.615
H 6.088 17.306 -1.659
H 4.050 14.294 -0.241
N 8.917 15.436 -0.206
C 10.157 16.082 0.215
C 10.445 17.271 -0.697
O 10.318 17.144 -1.891
C 11.319 15.104 0.168
C 11.224 13.946 1.131
C 10.591 12.754 0.772
C 11.739 14.054 2.418
C 10.461 11.747 1.720
C 11.612 13.026 3.354
C 11.003 11.858 2.973
H 8.836 14.881 -1.091
H 10.000 16.451 1.131
H 11.369 14.727 -0.752
H 12.152 15.604 0.382
H 10.242 12.627 -0.139
H 12.219 14.899 2.685
H 9.942 10.903 1.461
H 11.965 13.157 4.287
H 10.954 11.082 3.611
N 10.805 18.414 -0.110
C 11.215 19.572 -0.889
C 12.601 20.012 -0.416
O 13.062 19.549 0.615
C 10.178 20.705 -0.785
C 8.810 20.304 -1.287
O 10.002 21.069 0.577
H 10.767 18.400 0.926
H 11.282 19.308 -1.849
H 10.530 21.517 -1.294
H 8.404 21.054 -1.819
H 8.882 19.493 -1.875
H 8.207 20.092 -0.513
H 10.228 20.296 1.184
N 13.245 20.907 -1.191
C 14.576 21.403 -0.874
C 14.494 22.509 0.169
O 15.515 22.908 0.699
C 15.267 22.002 -2.100
C 15.573 21.055 -3.249
C 16.505 19.940 -2.880
C 17.914 20.391 -2.565
N 18.763 19.232 -2.191
H 12.703 21.205 -2.030
H 15.127 20.649 -0.502
H 14.681 22.745 -2.454
H 16.137 22.416 -1.794
H 14.701 20.663 -3.582
H 15.977 21.590 -4.007
H 16.139 19.459 -2.072
H 16.549 19.280 -3.643
H 18.303 20.838 -3.370
H 17.888 21.039 -1.803
H 19.542 19.550 -1.650
H 18.224 18.584 -1.651
H 19.095 18.781 -3.020
N 13.292 22.996 0.468
C 13.104 24.051 1.457
C 12.173 23.548 2.559
O 11.342 22.687 2.282
C 12.539 25.327 0.794
C 13.274 25.669 -0.504
C 11.038 25.240 0.548
H 12.506 22.561 -0.060
H 13.990 24.256 1.872
H 12.693 26.088 1.423
H 12.624 26.045 -1.161
H 13.985 26.343 -0.312
H 13.688 24.841 -0.877
H 10.803 25.793 -0.251
H 10.785 24.287 0.386
H 10.552 25.585 1.351
N 12.334 24.067 3.790
C 11.347 23.958 4.868
C 11.930 23.178 6.050
O 12.912 23.670 6.629
C 10.041 23.285 4.421
C 9.072 22.912 5.537
C 8.809 24.046 6.501
C 7.561 23.841 7.331
N 7.183 25.081 8.052
O 11.610 22.130 6.554
H 13.253 24.568 3.913
H 11.104 24.881 5.181
H 9.570 23.912 3.793
H 10.281 22.447 3.920
H 8.199 22.625 5.122
H 9.451 22.129 6.046
H 9.593 24.132 7.122
H 8.709 24.898 5.981
H 6.809 23.568 6.728
H 7.727 23.110 7.998
H 6.927 24.855 8.993
H 7.959 25.712 8.060
H 6.407 25.512 7.591
N -20.523 -14.089 -11.796
C -20.099 -14.864 -10.596
C -20.756 -14.264 -9.360
O -20.115 -14.092 -8.325
C -18.579 -14.848 -10.376
C -17.751 -14.280 -11.519
O -17.641 -14.950 -12.573
O -17.234 -13.164 -11.345
H -21.136 -13.307 -11.782
H -20.460 -15.785 -10.713
H -18.397 -14.305 -9.549
H -18.285 -15.795 -10.209
N -22.035 -13.911 -9.484
C -22.861 -13.554 -8.338
C -22.938 -14.710 -7.330
O -22.835 -14.514 -6.114
C -24.262 -13.238 -8.858

C	-25.229	-12.811	-7.790
C	-26.536	-12.325	-8.387
O	-27.438	-11.943	-7.608
O	-26.644	-12.323	-9.631
H	-22.388	-13.912	-10.466
H	-22.480	-12.743	-7.880
H	-24.177	-12.499	-9.506
H	-24.614	-14.056	-9.280
H	-25.435	-13.583	-7.223
H	-24.841	-12.064	-7.289
N	-23.211	-15.905	-7.872
C	-23.262	-17.157	-7.129
C	-21.866	-17.554	-6.647
O	-21.736	-18.080	-5.535
C	-23.850	-18.238	-8.008
H	-23.391	-15.856	-8.913
H	-23.854	-17.035	-6.324
H	-23.160	-18.558	-8.659
H	-24.150	-19.008	-7.443
H	-24.634	-17.875	-8.513
N	-20.847	-17.326	-7.503
C	-19.454	-17.591	-7.169
C	-19.028	-16.825	-5.920
O	-18.397	-17.394	-5.031
H	-21.154	-16.942	-8.425
H	-19.327	-18.579	-7.010
H	-18.864	-17.320	-7.941
N	-19.398	-15.531	-5.844
C	-19.015	-14.710	-4.700
C	-19.969	-14.893	-3.513
O	-19.543	-14.884	-2.385
C	-18.931	-13.220	-5.092
C	-17.789	-12.977	-6.074
C	-18.813	-12.386	-3.831
C	-17.817	-11.599	-6.725
H	-19.956	-15.193	-6.641
H	-18.115	-15.049	-4.396
H	-19.786	-12.975	-5.542
H	-16.923	-13.084	-5.590
H	-17.839	-13.666	-6.795
H	-18.819	-11.414	-4.068
H	-19.583	-12.582	-3.225
H	-17.958	-12.605	-3.362
H	-17.753	-10.902	-6.014
H	-17.043	-11.519	-7.349
H	-18.671	-11.493	-7.228
N	-21.272	-15.056	-3.753
C	-22.233	-15.102	-2.670
C	-21.907	-16.329	-1.812
O	-21.599	-17.394	-2.358
C	-23.691	-15.133	-3.170
C	-24.656	-15.478	-2.059
O	-24.113	-13.871	-3.704
H	-21.521	-15.142	-4.755
H	-22.126	-14.292	-2.103
H	-23.767	-15.836	-3.920
H	-25.589	-15.554	-2.424
H	-24.400	-16.353	-1.639
H	-24.640	-14.763	-1.354
H	-25.116	-13.833	-3.757
N	-21.893	-16.129	-0.493
C	-21.692	-17.218	0.446
C	-20.758	-16.851	1.579
O	-20.638	-15.683	1.951
H	-22.036	-15.139	-0.206
H	-22.590	-17.501	0.830
H	-21.317	-18.023	-0.050
N	-20.122	-17.886	2.137
C	-19.365	-17.738	3.366
C	-17.894	-18.008	3.046
O	-17.559	-18.993	2.374
C	-19.936	-18.602	4.509
C	-19.027	-18.593	5.714
O	-21.207	-18.124	4.977
H	-20.219	-18.781	1.620
H	-19.445	-16.799	3.686
H	-20.069	-19.563	4.160
H	-19.275	-17.836	6.328
H	-19.111	-19.459	6.217
H	-18.072	-18.476	5.427
H	-21.190	-17.123	5.069
N	-17.029	-17.092	3.523
C	-15.612	-17.097	3.215
C	-14.819	-17.016	4.500
O	-15.242	-16.365	5.454
C	-15.259	-15.907	2.309
C	-15.837	-15.972	0.942
C	-17.050	-15.498	0.560
C	-15.247	-16.576	-0.219
C	-16.180	-16.438	-1.262
C	-14.042	-17.242	-0.471
N	-17.246	-15.751	-0.764
C	-15.904	-16.812	-2.567
C	-13.790	-17.657	-1.754
C	-14.720	-17.471	-2.778
H	-17.482	-16.374	4.139
H	-15.396	-17.972	2.776
H	-15.594	-15.071	2.740
H	-14.266	-15.864	2.216
H	-17.702	-15.035	1.160
H	-18.055	-15.475	-1.297
H	-13.388	-17.408	0.259
H	-16.542	-16.609	-3.308
H	-12.917	-18.104	-1.960
H	-14.510	-17.833	-3.691
N	-13.618	-17.610	4.480
C	-12.750	-17.622	5.643
C	-11.348	-17.174	5.249
O	-10.856	-17.612	4.217
C	-12.721	-19.056	6.216
C	-14.058	-19.785	6.296
C	-14.898	-19.607	7.387
C	-14.460	-20.694	5.320
C	-16.110	-20.276	7.503
C	-15.682	-21.365	5.404
C	-16.506	-21.169	6.511
O	-17.733	-21.801	6.656
H	-13.373	-18.051	3.571
H	-13.108	-16.968	6.316
H	-12.103	-19.605	5.643
H	-12.339	-19.009	7.145
H	-14.622	-18.973	8.122
H	-13.862	-20.872	4.538
H	-16.699	-20.117	8.296
H	-15.964	-21.985	4.674
H	-18.302	-21.584	5.867
N	-10.673	-16.384	6.098
C	-9.315	-15.945	5.790
C	-8.300	-16.776	6.572
O	-8.662	-17.711	7.295
C	-9.115	-14.418	5.884
C	-9.051	-13.779	7.263
H	-11.185	-16.127	6.954
H	-9.156	-16.129	4.816
H	-8.254	-14.189	5.388
H	-9.873	-13.976	5.364
N	-9.135	-12.454	7.300
O	-8.943	-14.443	8.288
H	-9.241	-11.935	6.451
H	-9.094	-11.974	8.176
N	-7.014	-16.449	6.378
C	-5.923	-17.197	6.993
C	-5.956	-17.053	8.523
O	-5.413	-17.900	9.217
C	-4.590	-16.805	6.326
C	-4.085	-15.368	6.569
C	-4.810	-14.218	5.884
H	-6.870	-15.626	5.759
H	-6.050	-18.171	6.779
H	-3.871	-17.438	6.649
H	-4.682	-16.929	5.326
H	-4.101	-15.200	7.577
H	-3.101	-15.335	6.294
N	-4.435	-13.004	6.267
O	-5.692	-14.382	5.034
H	-4.864	-12.175	5.845
H	-3.722	-12.905	6.982
N	-6.577	-15.985	9.061
C	-6.547	-15.684	10.485
C	-7.770	-16.265	11.202
O	-7.970	-15.969	12.377
C	-6.557	-14.166	10.699
C	-5.437	-13.334	10.063
C	-5.662	-11.842	10.358
C	-4.056	-13.805	10.527
H	-7.080	-15.395	8.359
H	-5.722	-16.100	10.887
H	-7.427	-13.813	10.338
H	-6.526	-13.998	11.689
H	-5.441	-13.476	9.079
H	-5.662	-11.700	11.346
H	-4.928	-11.311	9.939
H	-6.540	-11.563	9.976
H	-3.929	-14.762	10.265
H	-3.353	-13.243	10.095
H	-3.994	-13.717	11.520
N	-8.647	-16.953	10.466
C	-9.887	-17.468	11.012
C	-10.999	-16.425	11.152
O	-11.898	-16.567	11.976
H	-8.358	-17.083	9.470
H	-10.227	-18.231	10.423
H	-9.708	-17.879	11.931
N	-10.937	-15.338	10.385
C	-12.064	-14.428	10.292
C	-13.076	-14.997	9.292
O	-12.697	-15.725	8.357
C	-11.602	-13.012	9.889
O	-10.743	-12.414	10.866
H	-10.044	-15.208	9.881
H	-12.511	-14.383	11.183
H	-11.109	-13.071	9.023
H	-12.409	-12.434	9.777
H	-10.676	-11.436	10.686
N	-14.353	-14.621	9.496
C	-15.490	-15.038	8.683
C	-16.151	-13.867	7.941
O	-16.634	-12.928	8.579
C	-16.594	-15.632	9.578
C	-17.690	-16.291	8.779
O	-15.958	-16.545	10.471
H	-14.460	-13.971	10.328
H	-15.166	-15.702	7.991
H	-17.010	-14.888	10.120
H	-18.032	-15.661	8.073
H	-17.343	-17.120	8.331
H	-18.453	-16.545	9.381
H	-15.339	-16.054	11.095
N	-16.299	-14.032	6.621
C	-16.879	-13.043	5.719
C	-18.113	-13.659	5.064
O	-17.977	-14.558	4.247
C	-15.753	-12.676	4.743
C	-16.080	-11.877	3.515
C	-16.755	-10.667	3.609
C	-15.563	-12.268	2.289
C	-16.995	-9.901	2.475
C	-15.814	-11.521	1.158
C	-16.530	-10.336	1.249
H	-15.948	-14.971	6.279
H	-17.200	-12.285	6.281
H	-15.058	-12.161	5.267
H	-15.327	-13.542	4.440
H	-17.070	-10.341	4.494
H	-15.004	-13.094	2.229
H	-17.505	-9.039	2.556
H	-15.475	-11.842	0.256
H	-16.710	-9.795	0.425
N	-19.299	-13.135	5.384
C	-20.551	-13.610	4.819
C	-21.102	-12.524	3.905
O	-21.283	-11.397	4.357
C	-21.542	-13.918	5.954
C	-20.926	-14.842	7.018
C	-22.846	-14.479	5.390

C	-21.598	-14.711	8.386
H	-19.245	-12.353	6.078
H	-20.323	-14.384	4.235
H	-21.780	-13.056	6.400
H	-21.011	-15.781	6.709
H	-19.965	-14.609	7.116
H	-23.507	-14.588	6.128
H	-23.208	-13.850	4.706
H	-22.669	-15.366	4.967
H	-22.563	-14.945	8.295
H	-21.154	-15.332	9.025
H	-21.504	-13.771	8.703
N	-21.308	-12.853	2.638
C	-21.570	-11.830	1.642
C	-22.674	-12.297	0.705
O	-22.689	-13.440	0.282
C	-20.256	-11.449	0.901
C	-19.719	-12.521	-0.021
C	-20.370	-10.149	0.130
H	-21.266	-13.865	2.431
H	-21.892	-11.005	2.113
H	-19.555	-11.284	1.610
H	-20.231	-12.516	-0.882
H	-18.751	-12.348	-0.213
H	-19.812	-13.419	0.413
H	-20.225	-9.379	0.752
H	-19.679	-10.129	-0.593
H	-21.281	-10.082	-0.278
N	-23.540	-11.357	0.327
C	-24.375	-11.506	-0.845
C	-23.921	-10.535	-1.938
O	-23.714	-9.354	-1.673
C	-25.857	-11.281	-0.516
C	-26.744	-11.501	-1.725
O	-26.235	-12.157	0.548
H	-23.559	-10.517	0.946
H	-24.277	-12.441	-1.200
H	-25.981	-10.321	-0.199
H	-26.659	-10.728	-2.358
H	-26.475	-12.343	-2.199
H	-27.702	-11.583	-1.436
H	-25.517	-12.185	1.253
N	-23.754	-11.063	-3.149
C	-23.489	-10.265	-4.335
C	-24.786	-10.037	-5.104
O	-25.300	-10.975	-5.713
C	-22.529	-10.992	-5.208
H	-23.830	-12.109	-3.172
H	-23.124	-9.374	-4.054
H	-21.601	-10.948	-4.822
H	-22.794	-11.958	-5.293
H	-22.512	-10.584	-6.127
N	-25.261	-8.790	-5.136
C	-26.428	-8.444	-5.931
C	-26.135	-8.364	-7.429
O	-24.990	-8.194	-7.884
H	-24.735	-8.111	-4.557
H	-27.159	-9.133	-5.775
H	-26.796	-7.549	-5.619
N	-27.216	-8.456	-8.222
C	-27.117	-8.401	-9.677
C	-26.557	-7.051	-10.138
O	-25.960	-6.950	-11.208
C	-28.482	-8.648	-10.296
H	-28.114	-8.568	-7.710
H	-26.492	-9.126	-9.990
H	-29.116	-7.930	-10.007
H	-28.405	-8.642	-11.293
H	-28.831	-9.536	-9.996
N	-26.803	-6.025	-9.317
C	-26.473	-4.641	-9.592
C	-25.025	-4.304	-9.221
O	-24.628	-3.155	-9.360
C	-27.377	-3.731	-8.750
C	-27.270	-3.909	-7.228
O	-26.925	-5.033	-6.756
O	-27.520	-2.906	-6.505
H	-27.277	-6.327	-8.415
H	-26.557	-4.501	-10.591
H	-27.148	-2.778	-8.969
H	-28.328	-3.906	-9.019
N	-24.256	-5.270	-8.708
C	-22.867	-5.020	-8.328
C	-22.699	-4.719	-6.839
O	-21.595	-4.424	-6.388
H	-24.723	-6.190	-8.607
H	-22.304	-5.829	-8.568
H	-22.509	-4.238	-8.866
N	-23.770	-4.832	-6.050
C	-23.672	-4.602	-4.620
C	-23.053	-5.820	-3.938
O	-23.213	-6.949	-4.386
C	-25.008	-4.292	-4.022
H	-24.645	-5.090	-6.538
H	-23.080	-3.809	-4.454
H	-24.925	-3.547	-3.354
H	-25.653	-4.012	-4.739
H	-25.376	-5.102	-3.556
N	-22.286	-5.527	-2.902
C	-21.937	-6.502	-1.871
C	-22.525	-6.027	-0.567
O	-22.373	-4.864	-0.167
C	-20.406	-6.622	-1.680
C	-19.563	-7.045	-2.870
C	-18.103	-7.039	-2.481
C	-19.959	-8.403	-3.454
H	-21.947	-4.536	-2.880
H	-22.347	-7.377	-2.112
H	-20.077	-5.722	-1.375
H	-20.253	-7.288	-0.944
H	-19.712	-6.404	-3.620
H	-17.950	-7.677	-1.725
H	-17.542	-7.317	-3.265
H	-17.831	-6.118	-2.194
H	-20.909	-8.367	-3.759
H	-19.364	-8.615	-4.228
H	-19.858	-9.107	-2.751
N	-23.134	-6.976	0.160
C	-23.746	-6.697	1.443
C	-23.447	-7.887	2.361
O	-23.306	-9.001	1.883
C	-25.259	-6.453	1.255
C	-25.576	-5.232	0.417
O	-25.789	-7.624	0.637
H	-23.123	-7.921	-0.282
H	-23.325	-5.889	1.837
H	-25.685	-6.347	2.165
H	-26.279	-4.677	0.870
H	-24.750	-4.675	0.295
H	-25.914	-5.513	-0.485
H	-25.103	-8.045	0.033
N	-23.283	-7.660	3.663
C	-23.105	-8.790	4.557
C	-22.551	-8.405	5.911
O	-22.848	-7.331	6.466
H	-23.294	-6.681	3.952
H	-23.999	-9.263	4.682
H	-22.481	-9.467	4.119
N	-21.796	-9.375	6.452
C	-21.235	-9.294	7.773
C	-19.830	-9.878	7.762
O	-19.528	-10.853	7.072
C	-22.083	-10.045	8.828
C	-23.534	-9.600	8.844
O	-21.972	-11.424	8.483
H	-21.657	-10.198	5.811
H	-21.169	-8.330	8.045
H	-21.670	-9.903	9.731
H	-23.898	-9.587	7.909
H	-24.080	-10.234	9.398
H	-23.605	-8.679	9.235
H	-21.799	-11.529	7.497
N	-19.022	-9.279	8.621
C	-17.669	-9.720	8.870
C	-17.512	-10.003	10.370
O	-17.835	-9.166	11.200
C	-16.672	-8.642	8.428
C	-15.263	-9.126	8.197
C	-14.327	-9.164	9.214
C	-14.873	-9.520	6.942
C	-13.048	-9.648	8.994
C	-13.607	-10.001	6.700
C	-12.681	-10.034	7.723
O	-11.468	-10.564	7.393
H	-19.452	-8.452	9.108
H	-17.511	-10.577	8.381
H	-17.010	-8.239	7.575
H	-16.648	-7.934	9.137
H	-14.574	-8.834	10.130
H	-15.526	-9.457	6.179
H	-12.399	-9.718	9.753
H	-13.354	-10.327	5.786
H	-11.145	-11.146	8.139
N	-16.871	-11.130	10.675
C	-16.390	-11.422	12.019
C	-14.868	-11.589	12.041
O	-14.388	-12.569	11.471
C	-17.054	-12.720	12.464
C	-16.449	-13.303	13.731
C	-17.529	-13.886	14.622
O	-17.450	-13.665	15.849
O	-18.498	-14.476	14.069
H	-16.745	-11.784	9.869
H	-16.651	-10.676	12.644
H	-17.999	-12.527	12.648
H	-16.937	-13.381	11.748
H	-15.848	-14.028	13.480
H	-16.013	-12.585	14.225
N	-14.166	-10.681	12.756
C	-12.703	-10.616	12.850
C	-12.208	-11.500	13.998
O	-12.687	-11.373	15.125
C	-12.200	-9.192	13.046
O	-10.779	-9.151	13.264
H	-14.793	-9.992	13.264
H	-12.321	-10.977	11.997
H	-12.408	-8.655	12.233
H	-12.650	-8.792	13.842
H	-10.604	-8.908	14.214
N	-11.209	-12.350	13.719
C	-10.606	-13.194	14.744
C	-9.501	-12.455	15.514
O	-8.993	-12.993	16.493
C	-10.095	-14.459	14.125
H	-10.911	-12.350	12.724
H	-11.320	-13.468	15.403
H	-10.848	-15.108	13.991
H	-9.676	-14.263	13.234
H	-9.406	-14.882	14.720
N	-9.150	-11.216	15.137
C	-8.148	-10.462	15.891
C	-8.593	-9.012	16.079
O	-9.572	-8.546	15.477
C	-6.745	-10.548	15.243
C	-6.052	-11.869	15.555
C	-6.776	-10.294	13.736
H	-9.632	-10.861	14.301
H	-8.061	-10.879	16.801
H	-6.185	-9.822	15.642
H	-5.404	-12.085	14.825
H	-5.563	-11.792	16.423
H	-6.735	-12.596	15.620
H	-6.428	-9.376	13.553
H	-6.203	-10.971	13.279
H	-7.718	-10.371	13.411
N	-7.866	-8.327	16.975
C	-7.978	-6.889	17.173
C	-9.139	-6.483	18.082
O	-9.737	-7.316	18.772
H	-7.204	-8.929	17.525
H	-7.110	-6.534	17.569
H	-8.093	-6.433	16.270
N	-9.444	-5.173	18.082
C	-10.519	-4.611	18.897
C	-11.867	-4.845	18.188

O	-12.504	-3.915	17.686
C	-10.199	-3.151	19.252
C	-11.150	-2.520	20.258
H	-8.846	-4.599	17.449
H	-10.550	-5.128	19.750
H	-9.270	-3.118	19.630
H	-10.226	-2.611	18.406
N	-10.829	-1.309	20.678
O	-12.170	-3.097	20.640
H	-10.007	-0.853	20.334
H	-11.411	-0.837	21.346
N	-12.280	-6.119	18.126
C	-13.472	-6.539	17.402
C	-14.161	-7.665	18.176
O	-13.478	-8.532	18.718
C	-13.106	-7.008	16.010
H	-11.663	-6.787	18.649
H	-14.104	-5.767	17.335
H	-13.161	-8.007	15.964
H	-13.738	-6.613	15.342
H	-12.172	-6.720	15.790
N	-15.498	-7.667	18.211
C	-16.218	-8.825	18.735
C	-17.620	-8.925	18.135
O	-18.416	-7.982	18.164
C	-16.273	-8.855	20.274
C	-17.330	-7.961	20.916
C	-17.395	-8.035	22.436
O	-18.105	-8.921	22.949
O	-16.713	-7.221	23.106
H	-15.951	-6.818	17.850
H	-15.704	-9.667	18.525
H	-16.466	-9.787	20.546
H	-15.389	-8.560	20.608
H	-17.127	-7.023	20.674
H	-18.217	-8.233	20.570
N	-17.905	-10.146	17.680
C	-19.122	-10.477	16.974
C	-19.017	-9.892	15.561
O	-17.921	-9.676	15.021
C	-20.371	-10.034	17.760
O	-21.515	-10.828	17.419
H	-17.151	-10.855	17.885
H	-19.184	-11.466	16.886
H	-20.190	-10.136	18.733
H	-20.566	-9.083	17.542
H	-21.301	-11.377	16.615
N	-20.176	-9.708	14.944
C	-20.254	-9.455	13.523
C	-20.358	-7.949	13.329
O	-20.876	-7.213	14.189
C	-21.434	-10.209	12.898
C	-21.607	-11.656	13.367
C	-22.556	-12.556	12.524
N	-21.258	-12.892	11.922
C	-20.752	-14.128	11.868
N	-19.535	-14.327	11.409
N	-21.456	-15.164	12.299
H	-21.011	-9.762	15.567
H	-19.421	-9.775	13.082
H	-22.274	-9.715	13.119
H	-21.306	-10.223	11.907
H	-20.702	-12.115	13.384
H	-21.963	-11.661	14.317
H	-22.810	-13.220	13.058
H	-22.996	-12.009	11.978
H	-20.717	-12.144	11.527
H	-18.958	-13.568	11.113
H	-19.167	-15.267	11.354
H	-22.371	-15.007	12.687
H	-21.088	-16.086	12.242
N	-19.851	-7.517	12.166
C	-19.951	-6.130	11.752
C	-20.541	-6.079	10.345
O	-20.324	-6.985	9.538
C	-18.576	-5.457	11.830
C	-17.972	-5.415	13.215
C	-17.362	-6.527	13.781
C	-18.003	-4.251	13.955
C	-16.795	-6.474	15.045
C	-17.492	-4.203	15.244
C	-16.875	-5.316	15.785
O	-16.340	-5.184	17.065
H	-19.389	-8.258	11.601
H	-20.579	-5.655	12.368
H	-17.951	-5.957	11.230
H	-18.671	-4.517	11.503
H	-17.329	-7.388	13.266
H	-18.401	-3.422	13.558
H	-16.327	-7.281	15.417
H	-17.571	-3.362	15.783
H	-15.516	-4.627	17.010
N	-21.303	-5.014	10.099
C	-21.855	-4.737	8.791
C	-20.709	-4.454	7.809
O	-19.796	-3.696	8.145
C	-22.842	-3.558	8.831
C	-23.248	-3.143	7.409
C	-24.082	-3.887	9.694
H	-21.461	-4.402	10.933
H	-22.336	-5.552	8.466
H	-22.373	-2.778	9.232
H	-22.659	-2.398	7.105
H	-23.145	-3.926	6.799
H	-24.198	-2.842	7.415
H	-24.899	-3.754	9.140
H	-24.019	-4.835	9.994
H	-24.094	-3.276	10.480
N	-20.790	-5.081	6.634
C	-19.950	-4.691	5.503
C	-20.810	-4.319	4.303
O	-21.948	-4.761	4.120
C	-18.968	-5.811	5.154
C	-19.498	-6.982	4.352
C	-19.448	-6.745	2.840
C	-18.731	-8.223	4.743
H	-21.484	-5.848	6.595
H	-19.409	-3.887	5.768
H	-18.217	-5.399	4.630
H	-18.604	-6.172	6.015
H	-20.455	-7.163	4.620
H	-18.501	-6.589	2.562
H	-19.809	-7.548	2.369
H	-20.001	-5.945	2.613
H	-18.850	-8.403	5.722
H	-19.069	-9.010	4.221
H	-17.756	-8.096	4.550
N	-20.251	-3.406	3.512
C	-20.798	-3.075	2.222
C	-19.632	-2.882	1.249
O	-18.549	-2.435	1.634
C	-21.722	-1.862	2.343
C	-21.025	-0.559	2.687
O	-22.332	-1.736	1.071
H	-19.394	-2.962	3.911
H	-21.352	-3.836	1.892
H	-22.434	-2.059	3.026
H	-20.314	-0.361	2.007
H	-21.689	0.193	2.692
H	-20.601	-0.627	3.594
H	-22.402	-2.634	0.617
N	-19.893	-3.202	0.001
C	-18.935	-3.009	-1.074
C	-19.567	-3.116	-2.461
O	-20.795	-3.021	-2.625
H	-20.847	-3.608	-0.155
H	-18.501	-2.096	-0.980
H	-18.195	-3.699	-0.996
N	-18.697	-3.305	-3.448
C	-19.040	-3.335	-4.850
C	-18.169	-4.382	-5.517
O	-17.016	-4.645	-5.134
C	-18.853	-1.953	-5.499
C	-19.641	-0.795	-4.901
C	-21.122	-0.800	-5.198
N	-21.374	-0.727	-6.643
C	-22.520	-1.067	-7.222
N	-23.571	-1.314	-6.458
N	-22.593	-1.177	-8.546
H	-17.701	-3.435	-3.103
H	-19.996	-3.601	-4.951
H	-17.874	-1.719	-5.451
H	-19.110	-2.032	-6.470
H	-19.522	-0.802	-3.895
H	-19.255	0.077	-5.245
H	-21.512	-1.643	-4.844
H	-21.535	-0.011	-4.757
H	-20.626	-0.396	-7.230
H	-23.542	-1.235	-5.469
H	-24.445	-1.594	-6.896
H	-21.781	-0.981	-9.101
H	-23.446	-1.449	-8.981
N	-18.715	-4.974	-6.578
C	-17.933	-5.867	-7.395
C	-18.298	-5.632	-8.855
O	-19.352	-5.078	-9.134
C	-18.114	-7.316	-6.936
C	-19.422	-7.923	-7.395
C	-20.629	-7.552	-6.848
C	-19.425	-8.849	-8.423
C	-21.823	-8.117	-7.294
C	-20.603	-9.424	-8.872
C	-21.794	-9.060	-8.298
O	-22.911	-9.681	-8.781
H	-19.706	-4.741	-6.746
H	-16.964	-5.647	-7.280
H	-17.370	-7.857	-7.311
H	-18.094	-7.334	-5.943
H	-20.662	-6.867	-6.116
H	-18.558	-9.111	-8.853
H	-22.692	-7.834	-6.885
H	-20.580	-10.100	-9.611
H	-23.725	-9.185	-8.476
N	-17.402	-6.052	-9.745
C	-17.608	-6.012	-11.180
C	-18.549	-7.156	-11.566
O	-18.146	-8.307	-11.584
C	-16.266	-6.064	-11.918
C	-16.441	-5.772	-13.369
O	-17.538	-5.325	-13.809
O	-15.458	-5.862	-14.149
H	-16.515	-6.421	-9.308
H	-18.085	-5.162	-11.394
H	-15.573	-5.307	-11.488
H	-15.798	-7.066	-11.791
N	-19.796	-6.814	-11.866
C	-20.824	-7.788	-12.223
C	-20.795	-8.184	-13.699
O	-21.638	-8.985	-14.119
C	-22.163	-7.249	-11.868
O	-22.370	-6.001	-12.508
H	-19.982	-5.784	-11.826
H	-20.662	-8.628	-11.676
H	-22.874	-7.878	-12.168
H	-22.223	-7.110	-10.883
H	-21.533	-5.725	-12.980
N	-19.834	-7.684	-14.490
C	-19.613	-8.249	-15.823
C	-18.118	-8.397	-16.108
O	-17.541	-7.606	-16.905
C	-20.330	-7.420	-16.880
H	-19.297	-6.907	-14.094
H	-20.030	-9.167	-15.857
H	-20.700	-6.591	-16.461
H	-19.685	-7.168	-17.601
H	-21.077	-7.955	-17.274
N	-17.371	-9.344	-15.479
C	-15.898	-9.367	-15.505
C	-15.308	-9.622	-16.876
O	-16.033	-9.676	-17.906
C	-15.517	-10.496	-14.566
C	-16.763	-11.343	-14.521
C	-17.930	-10.387	-14.635
H	-15.503	-8.411	-15.107
H	-14.643	-11.107	-14.880

H	-15.313	-10.082	-13.552
H	-16.774	-12.009	-15.268
H	-16.786	-11.830	-13.645
H	-18.721	-10.831	-15.048
H	-18.176	-10.009	-13.746
N	-13.887	-9.670	-17.030
C	-13.288	-10.143	-18.281
C	-13.542	-11.626	-18.493
O	-13.486	-12.424	-17.523
C	-11.791	-9.837	-18.278
H	-13.320	-9.773	-16.158
H	-13.746	-9.590	-19.130
H	-11.294	-10.412	-17.471
H	-11.331	-10.118	-19.250
H	-11.619	-8.754	-18.107
N	-13.571	-12.148	-19.822
C	-13.674	-13.596	-20.147
C	-12.403	-14.032	-20.865
O	-12.420	-14.366	-22.045
C	-14.888	-13.855	-21.063
C	-16.234	-13.672	-20.389
O	-14.785	-12.969	-22.184
H	-13.214	-11.500	-20.561
H	-13.761	-14.122	-19.311
H	-14.84	-14.808	-21.404
H	-16.919	-13.385	-21.064
H	-16.527	-14.536	-19.972
H	-16.166	-12.971	-19.675
H	-14.896	-12.014	-21.886
N	-11.228	-13.625	-20.288
C	-9.903	-14.101	-20.691
C	-9.171	-14.801	-19.547
O	-7.914	-14.911	-19.561
C	-9.084	-12.950	-21.320
C	-8.763	-11.823	-20.369
O	-9.238	-11.796	-19.201
O	-8.035	-10.865	-20.744
H	-11.271	-13.007	-19.446
H	-10.086	-14.876	-21.427
H	-8.131	-13.360	-21.719
H	-9.650	-12.536	-22.182
N	-9.896	-15.423	-18.565
C	-9.301	-16.029	-17.388
C	-9.010	-15.033	-16.281
O	-8.535	-15.476	-15.208
H	-10.931	-15.313	-18.692
H	-9.915	-16.765	-17.037
H	-8.427	-16.489	-17.646
N	-9.171	-13.676	-16.430
C	-8.720	-12.692	-15.429
C	-9.738	-12.522	-14.341
O	-10.901	-12.135	-14.627
C	-8.425	-11.319	-16.052
O	-7.200	-11.344	-16.738
H	-9.662	-13.320	-17.278
H	-7.778	-13.050	-14.973
H	-9.259	-10.995	-16.710
H	-8.346	-10.555	-15.250
H	-7.437	-11.565	-17.687
N	-9.284	-12.458	-12.993
C	-10.167	-12.379	-11.820
C	-11.145	-11.233	-11.827
O	-10.794	-10.078	-12.198
H	-8.273	-12.619	-12.802
H	-10.758	-13.311	-11.711
H	-9.561	-12.302	-10.897
N	-12.236	-11.356	-10.924
C	-13.327	-10.370	-10.783
C	-12.971	-9.373	-9.756
O	-12.952	-9.728	-8.543
C	-14.642	-11.041	-10.375
C	-15.797	-10.096	-10.226
O	-14.895	-11.978	-11.410
H	-12.210	-12.233	-10.359
H	-13.496	-9.881	-11.766
H	-14.498	-11.539	-9.518
H	-16.123	-9.795	-11.130
H	-16.559	-10.545	-9.746
H	-15.521	-9.284	-9.699
H	-15.804	-12.398	-11.297
N	-12.802	-8.068	-10.114
C	-12.439	-7.024	-9.158
C	-13.558	-6.757	-8.157
O	-14.721	-6.679	-8.564
C	-12.069	-5.749	-9.912
H	-12.941	-7.820	-11.118
H	-11.526	-7.363	-8.624
H	-12.960	-5.332	-10.429
H	-11.665	-4.998	-9.204
H	-11.283	-5.976	-10.662
N	-13.236	-6.522	-6.870
C	-14.197	-6.193	-5.841
C	-13.525	-5.499	-4.656
O	-12.297	-5.405	-4.603
C	-14.919	-7.463	-5.370
C	-14.037	-8.653	-4.981
C	-13.186	-8.375	-3.774
C	-14.917	-9.871	-4.743
H	-12.200	-6.604	-6.686
H	-14.884	-5.562	-6.223
H	-15.464	-7.223	-4.569
H	-15.517	-7.764	-6.108
H	-13.447	-8.884	-5.769
H	-13.767	-8.160	-2.985
H	-12.626	-9.179	-3.556
H	-12.580	-7.596	-3.953
H	-15.422	-10.087	-5.579
H	-14.346	-10.652	-4.489
H	-15.564	-9.678	-4.005
N	-14.347	-4.908	-3.802
C	-13.879	-4.298	-2.570
C	-15.017	-4.134	-1.576
O	-16.197	-4.104	-1.966
H	-15.352	-4.920	-4.089
H	-13.160	-4.872	-2.160
H	-13.481	-3.393	-2.771
N	-14.665	-4.013	-0.288
C	-15.668	-3.772	0.723
C	-15.019	-3.047	1.882
O	-13.793	-2.997	1.975
C	-16.400	-5.071	1.092
C	-15.559	-6.078	1.840
C	-15.416	-6.133	3.188
C	-14.754	-7.147	1.319
C	-14.177	-7.810	2.430
C	-14.450	-7.631	0.064
N	-14.640	-7.187	3.547
C	-13.328	-8.902	2.308
C	-13.619	-8.727	-0.060
C	-13.082	-9.371	1.051
H	-13.655	-4.104	-0.104
H	-16.361	-3.164	0.336
H	-17.176	-4.850	1.675
H	-16.711	-5.514	0.256
H	-15.830	-5.479	3.833
H	-14.436	-7.469	4.492
H	-14.825	-7.198	-0.763
H	-12.917	-9.319	3.126
H	-13.399	-9.063	-0.980
H	-12.515	-10.180	0.918
N	-15.891	-2.472	2.710
C	-15.566	-1.744	3.906
C	-16.318	-2.327	5.097
O	-17.523	-2.588	5.003
C	-15.965	-0.273	3.796
C	-15.506	0.555	4.976
O	-15.396	0.287	2.612
H	-16.905	-2.610	2.387
H	-14.580	-1.832	4.088
H	-16.982	-0.208	3.722
H	-15.064	-0.034	5.660
H	-14.849	1.252	4.674
H	-16.292	1.013	5.402
H	-15.509	1.287	2.606
N	-15.589	-2.442	6.204
C	-16.171	-2.549	7.541
C	-15.713	-1.390	8.408
O	-14.512	-1.140	8.549
C	-15.728	-3.861	8.194
C	-16.132	-3.916	9.665
C	-16.243	-5.033	7.408
H	-14.554	-2.449	6.035
H	-17.169	-2.497	7.450
H	-14.730	-3.897	8.169
H	-15.390	-3.550	10.227
H	-16.956	-3.370	9.804
H	-16.310	-4.865	9.925
H	-16.922	-5.533	7.950
H	-16.671	-4.711	6.560
H	-15.485	-5.650	7.181
N	-16.676	-0.687	9.014
C	-16.379	0.198	10.122
C	-16.583	-0.615	11.408
O	-17.572	-1.332	11.527
C	-17.264	1.405	10.095
H	-17.626	-0.833	8.625
H	-15.430	0.479	10.078
H	-17.746	1.461	9.216
H	-17.943	1.354	10.832
H	-16.718	2.238	10.216
N	-15.633	-0.486	12.341
C	-15.488	-1.371	13.480
C	-16.308	-0.841	14.660
O	-15.804	-0.642	15.765
C	-14.014	-1.565	13.820
C	-13.296	-2.295	12.722
C	-12.424	-1.740	11.843
C	-13.428	-3.668	12.345
C	-12.605	-3.871	11.217
C	-14.170	-4.758	12.814
N	-11.984	-2.677	10.954
C	-12.481	-5.113	10.598
C	-14.070	-5.972	12.178
C	-13.221	-6.161	11.092
H	-14.988	0.331	12.168
H	-15.868	-2.265	13.232
H	-13.585	-0.686	13.918
H	-13.938	-2.112	14.634
H	-12.141	-0.777	11.843
H	-11.315	-2.524	10.224
H	-14.772	-4.654	13.612
H	-11.862	-5.227	9.810
H	-14.627	-6.740	12.507
H	-13.151	-7.060	10.674
N	-17.588	-0.634	14.377
C	-18.579	-0.286	15.370
C	-19.760	-1.232	15.202
O	-20.226	-1.505	14.098
C	-19.011	1.168	15.197
C	-20.074	1.639	16.174
C	-20.510	3.066	15.982
C	-21.719	3.428	16.825
N	-22.871	2.520	16.564
H	-17.819	-0.748	13.355
H	-18.195	-0.413	16.281
H	-18.203	1.750	15.309
H	-19.368	1.279	14.267
H	-20.884	1.033	16.087
H	-19.719	1.528	17.118
H	-19.757	3.677	16.235
H	-20.743	3.213	15.019
H	-21.469	3.370	17.796
H	-21.992	4.372	16.618
H	-23.212	2.161	17.431
H	-23.596	3.030	16.103
H	-22.571	1.764	15.983
N	-20.209	-1.809	16.327
C	-21.461	-2.548	16.350
C	-22.129	-2.251	17.707
O	-21.788	-1.274	18.390
C	-21.238	-4.043	16.039
C	-20.386	-4.806	17.053
H	-19.596	-1.680	17.151
H	-22.052	-2.164	15.654
H	-22.138	-4.488	15.984

H	-20.793	-4.110	15.139
N	-20.037	-6.041	16.717
O	-20.019	-4.280	18.110
H	-20.326	-6.421	15.836
H	-19.485	-6.594	17.340
N	-23.062	-3.110	18.119
C	-23.750	-2.934	19.399
C	-22.862	-3.204	20.619
O	-23.129	-2.697	21.704
C	-25.028	-3.763	19.414
C	-26.049	-3.111	18.516
H	-23.254	-3.893	17.469
H	-24.034	-1.960	19.453
H	-24.825	-4.661	19.069
H	-25.377	-3.786	20.333
N	-26.745	-3.903	17.721
O	-26.170	-1.888	18.518
H	-26.582	-4.895	17.741
H	-27.431	-3.525	17.101
N	-21.778	-3.952	20.445
C	-20.949	-4.370	21.556
C	-19.711	-3.484	21.693
O	-19.271	-3.226	22.815
C	-20.656	-5.859	21.369
C	-21.916	-6.673	21.183
C	-22.891	-6.706	22.172
C	-22.155	-7.385	20.013
C	-24.061	-7.433	22.012
C	-23.317	-8.116	19.837
C	-24.277	-8.137	20.839
O	-25.441	-8.850	20.676
H	-21.583	-4.206	19.453
H	-21.484	-4.287	22.408
H	-20.092	-5.968	20.562
H	-20.188	-6.190	22.177
H	-22.747	-6.193	23.025
H	-21.471	-7.367	19.281
H	-24.749	-7.448	22.742
H	-23.467	-8.629	18.991
H	-25.277	-9.606	20.045
N	-19.145	-2.988	20.580
C	-17.820	-2.376	20.628
C	-17.661	-1.258	19.600
O	-18.375	-1.222	18.605
C	-16.775	-3.469	20.389
C	-16.389	-4.190	21.670
C	-14.909	-4.475	21.767
N	-14.611	-5.887	21.641
C	-13.439	-6.420	21.949
N	-13.292	-7.735	21.988
N	-12.429	-5.626	22.257
H	-19.712	-3.076	19.726
H	-17.675	-1.986	21.551
H	-17.157	-4.133	19.764
H	-15.964	-3.047	20.015
H	-16.654	-3.626	22.459
H	-16.881	-5.064	21.718
H	-14.446	-3.971	21.038
H	-14.587	-4.145	22.656
H	-15.334	-6.497	21.301
H	-14.049	-8.346	21.769
H	-12.399	-8.129	22.243
H	-12.541	-4.631	22.238
H	-11.540	-6.018	22.509
N	-16.779	-0.307	19.886
C	-16.424	0.700	18.895
C	-14.914	0.908	18.926
O	-14.361	1.426	19.897
C	-17.212	2.006	19.032
C	-17.004	2.911	17.831
H	-16.382	-0.345	20.838
H	-16.650	0.329	17.996
H	-18.176	1.785	19.100
H	-16.899	2.478	19.845
N	-17.741	4.011	17.789
O	-16.170	2.616	16.959
H	-18.388	4.210	18.527
H	-17.654	4.646	17.021
N	-14.236	0.493	17.844
C	-12.793	0.611	17.793
C	-12.339	1.874	17.076
O	-11.136	2.046	16.880
C	-12.223	-0.616	17.150
H	-14.819	0.101	17.091
H	-12.432	0.634	18.741
H	-11.221	-0.586	17.173
H	-12.532	-1.438	17.638
H	-12.522	-0.677	16.193
N	-13.290	2.726	16.664
C	-13.001	4.014	16.055
C	-12.038	3.864	14.869
O	-11.032	4.584	14.764
C	-12.485	5.003	17.106
C	-13.447	5.187	18.241
H	-14.266	2.374	16.824
H	-13.861	4.408	15.706
H	-11.620	4.664	17.476
H	-12.335	5.891	16.671
C	-13.282	5.043	19.576
N	-14.774	5.515	18.050
C	-15.382	5.563	19.238
N	-14.505	5.264	20.166
H	-15.209	5.687	17.174
H	-12.433	4.820	20.049
H	-16.341	5.790	19.382
N	-12.375	2.910	13.997
C	-11.510	2.540	12.879
C	-12.344	1.860	11.792
O	-13.489	1.446	12.029
C	-10.352	1.687	13.354
O	-10.808	0.443	13.834
H	-13.295	2.461	14.182
H	-11.125	3.378	12.491
H	-9.727	1.532	12.592
H	-9.877	2.164	14.090
H	-11.736	0.273	13.501
N	-11.752	1.731	10.594
C	-12.427	1.121	9.455
C	-11.413	0.426	8.547
O	-10.293	0.910	8.418
C	-13.188	2.187	8.698
H	-10.780	2.102	10.556
H	-13.077	0.432	9.791
H	-13.064	3.074	9.145
H	-12.849	2.242	7.758
H	-14.163	1.961	8.683
N	-11.769	-0.743	8.001
C	-10.920	-1.479	7.079
C	-11.589	-1.521	5.712
O	-12.795	-1.767	5.621
C	-10.643	-2.908	7.579
C	-9.768	-3.725	6.656
O	-9.994	-2.721	8.840
H	-12.716	-1.078	8.300
H	-10.048	-0.997	6.989
H	-11.516	-3.373	7.735
H	-9.915	-3.444	5.703
H	-8.801	-3.590	6.888
H	-9.990	-4.701	6.744
H	-9.403	-1.907	8.813
N	-10.807	-1.286	4.658
C	-11.228	-1.623	3.304
C	-10.336	-2.723	2.759
O	-9.111	-2.742	2.923
C	-11.205	-0.413	2.351
C	-12.148	0.678	2.778
O	-9.863	0.073	2.245
H	-9.893	-0.851	4.883
H	-12.170	-1.963	3.338
H	-11.509	-0.718	1.427
H	-12.198	0.723	3.782
H	-11.831	1.568	2.431
H	-13.070	0.503	2.418
H	-9.378	-0.047	3.118
N	-10.996	-3.673	2.085
C	-10.389	-4.732	1.334
C	-10.598	-4.461	-0.145
O	-11.720	-4.276	-0.570
C	-10.983	-6.098	1.676
C	-10.674	-6.619	3.054
C	-9.784	-7.609	3.372
C	-11.355	-6.305	4.270
C	-10.809	-7.138	5.279
C	-12.398	-5.443	4.615
N	-9.835	-7.876	4.706
C	-11.222	-7.073	6.606
C	-12.828	-5.396	5.926
C	-12.248	-6.214	6.898
H	-12.055	-3.560	2.164
H	-9.403	-4.718	1.505
H	-11.981	-6.046	1.601
H	-10.638	-6.774	1.023
H	-9.185	-8.068	2.715
H	-9.234	-8.528	5.190
H	-12.832	-4.863	3.922
H	-10.780	-7.638	7.305
H	-13.561	-4.771	6.177
H	-12.599	-6.164	7.839
N	-9.497	-4.528	-0.895
C	-9.486	-4.368	-2.319
C	-8.891	-5.629	-2.905
O	-7.788	-6.061	-2.544
C	-8.677	-3.166	-2.712
O	-8.884	-2.912	-4.089
H	-8.621	-4.713	-0.327
H	-10.425	-4.294	-2.641
H	-8.976	-2.379	-2.193
H	-7.716	-3.349	-2.563
H	-8.661	-1.958	-4.290
N	-9.614	-6.269	-3.823
C	-9.038	-7.460	-4.383
C	-9.838	-8.070	-5.530
O	-10.485	-7.369	-6.311
H	-10.510	-5.870	-4.055
H	-8.090	-7.260	-4.717
H	-8.929	-8.167	-3.651
N	-9.585	-9.360	-5.743
C	-10.247	-10.043	-6.836
C	-10.626	-11.467	-6.427
O	-9.899	-12.137	-5.705
C	-9.434	-9.981	-8.129
C	-8.148	-10.763	-8.203
C	-7.379	-10.462	-9.438
H	-8.919	-9.795	-5.098
H	-11.099	-9.552	-7.047
H	-10.035	-10.290	-8.896
H	-9.208	-8.910	-8.335
H	-7.571	-10.521	-7.422
H	-8.355	-11.741	-8.173
N	-6.095	-10.095	-9.344
O	-7.899	-10.544	-10.566
H	-5.549	-9.986	-10.223
H	-5.704	-10.055	-8.392
N	-11.784	-11.874	-6.952
C	-12.289	-13.241	-6.859
C	-11.739	-14.023	-8.045
O	-11.807	-13.561	-9.196
C	-13.809	-13.147	-6.873
C	-14.471	-14.447	-7.255
C	-14.634	-15.451	-6.312
C	-14.860	-14.690	-8.561
C	-15.204	-16.665	-6.650
C	-15.485	-15.884	-8.903
C	-15.640	-16.873	-7.947
O	-16.253	-18.078	-8.228
H	-12.312	-11.109	-7.448
H	-11.936	-13.651	-6.024
H	-14.116	-12.895	-5.963
H	-14.075	-12.456	-7.535
H	-14.333	-15.295	-5.367
H	-14.693	-14.005	-9.264
H	-15.301	-17.387	-5.964
H	-15.819	-16.025	-9.833
H	-17.155	-18.090	-7.802
N	-11.227	-15.231	-7.759

C	-10.680	-16.140	-8.742
C	-11.447	-17.454	-8.563
O	-11.327	-18.102	-7.531
C	-9.162	-16.343	-8.534
C	-8.546	-17.207	-9.617
C	-8.398	-15.031	-8.427
H	-11.262	-15.459	-6.726
H	-10.881	-15.795	-9.645
H	-9.041	-16.827	-7.666
H	-7.853	-17.809	-9.215
H	-9.256	-17.765	-10.049
H	-8.116	-16.625	-10.309
H	-8.723	-14.525	-7.628
H	-7.422	-15.224	-8.329
H	-8.553	-14.491	-9.253
N	-12.230	-17.828	-9.565
C	-13.104	-18.987	-9.454
C	-12.349	-20.273	-9.771
O	-11.170	-20.241	-10.087
H	-12.167	-17.237	-10.415
H	-13.474	-19.042	-8.518
H	-13.877	-18.890	-10.092
N	-13.066	-21.395	-9.747
C	-12.486	-22.686	-10.070
C	-12.643	-23.621	-8.883
O	-13.390	-23.316	-7.951
H	-14.065	-21.263	-9.481
H	-12.951	-23.077	-10.866
H	-11.513	-22.576	-10.280
N	-11.920	-24.741	-8.906
C	-12.222	-25.822	-7.977
C	-11.897	-25.364	-6.565
O	-12.623	-25.705	-5.621
C	-11.455	-27.063	-8.346
H	-11.169	-24.769	-9.605
H	-13.198	-26.033	-8.030
H	-10.966	-27.416	-7.545
H	-12.086	-27.769	-8.679
H	-10.791	-26.854	-9.067
N	-10.806	-24.584	-6.464
C	-10.340	-24.030	-5.209
C	-10.443	-22.506	-5.295
O	-9.437	-21.823	-5.464
C	-8.908	-24.486	-4.922
C	-8.736	-26.001	-4.964
C	-8.070	-26.586	-3.733
O	-7.051	-26.024	-3.291
O	-8.572	-27.607	-3.224
H	-10.327	-24.416	-7.382
H	-10.905	-24.356	-4.456
H	-8.327	-24.103	-5.613
H	-8.671	-24.185	-4.019
H	-9.633	-26.409	-5.040
H	-8.171	-26.226	-5.743
N	-11.680	-22.018	-5.152
C	-12.022	-20.619	-5.337
C	-11.412	-19.778	-4.213
O	-11.351	-20.174	-3.046
C	-13.516	-20.498	-5.400
H	-12.391	-22.750	-4.891
H	-11.645	-20.300	-6.211
H	-13.851	-20.825	-6.288
H	-13.937	-21.045	-4.674
H	-13.790	-19.540	-5.285
N	-10.991	-18.551	-4.562
C	-10.290	-17.703	-3.618
C	-10.720	-16.255	-3.853
O	-11.036	-15.884	-4.999
C	-8.771	-17.820	-3.861
C	-8.033	-18.809	-2.966
C	-6.579	-18.966	-3.376
N	-5.782	-19.599	-2.337
C	-5.971	-20.815	-1.841
N	-5.171	-21.258	-0.888
N	-6.939	-21.596	-2.292
H	-11.209	-18.283	-5.536
H	-10.544	-17.962	-2.688
H	-8.633	-18.101	-4.813
H	-8.368	-16.915	-3.723
H	-8.059	-18.483	-2.023
H	-8.472	-19.703	-3.029
H	-6.550	-19.524	-4.203
H	-6.213	-18.058	-3.567
H	-5.006	-19.060	-1.952
H	-4.439	-20.701	-0.510
H	-5.305	-22.196	-0.524
H	-7.551	-21.243	-3.011
H	-7.065	-22.511	-1.930
N	-10.692	-15.469	-2.790
C	-10.669	-14.008	-2.925
C	-9.337	-13.536	-2.362
O	-9.036	-13.741	-1.194
C	-11.872	-13.348	-2.242
C	-13.191	-13.860	-2.849
C	-11.782	-11.824	-2.317
C	-14.409	-13.403	-2.116
H	-10.688	-15.955	-1.877
H	-10.652	-13.821	-3.897
H	-11.863	-13.602	-1.280
H	-13.233	-13.549	-3.799
H	-13.157	-14.861	-2.845
H	-12.543	-11.423	-1.811
H	-10.919	-11.524	-1.917
H	-11.826	-11.536	-3.271
H	-14.454	-12.402	-2.120
H	-15.231	-13.768	-2.559
H	-14.377	-13.725	-1.167
N	-8.527	-12.927	-3.217
C	-7.221	-12.452	-2.829
C	-7.306	-10.937	-2.640
O	-7.677	-10.249	-3.593
C	-6.198	-12.760	-3.918
C	-5.910	-14.239	-4.092
H	-8.916	-12.825	-4.181
H	-7.008	-12.840	-1.935
H	-6.542	-12.401	-4.787
H	-5.340	-12.296	-3.685
N	-5.829	-14.660	-5.334
O	-5.789	-14.971	-3.113
H	-5.946	-14.032	-6.104
H	-5.646	-15.634	-5.527
N	-6.902	-10.440	-1.468
C	-7.120	-9.044	-1.108
C	-5.854	-8.415	-0.546
O	-4.954	-9.087	-0.026
C	-8.249	-8.787	-0.109
C	-9.550	-9.368	-0.607
O	-7.918	-9.255	1.216
H	-6.427	-11.126	-0.849
H	-7.384	-8.537	-1.939
H	-8.419	-7.772	-0.046
H	-9.442	-10.349	-0.794
H	-10.269	-9.247	0.083
H	-9.834	-8.907	-1.453
H	-7.505	-10.168	1.165
N	-5.820	-7.091	-0.721
C	-5.044	-6.246	0.157
C	-5.991	-5.356	0.915
O	-7.066	-5.032	0.428
C	-4.086	-5.437	-0.729
C	-3.079	-6.408	-1.351
C	-1.855	-5.838	-2.025
H	-6.382	-6.745	-1.518
H	-4.571	-6.831	0.816
H	-4.594	-5.017	-1.449
H	-3.596	-4.801	-0.173
H	-2.771	-7.052	-0.615
H	-3.583	-6.986	-2.031
N	-1.819	-5.963	-3.330
O	-0.912	-5.389	-1.385
H	-1.024	-5.588	-3.874
H	-2.580	-6.433	-3.814
N	-5.590	-4.938	2.108
C	-6.456	-4.128	2.915
C	-5.721	-2.925	3.519
O	-4.498	-2.973	3.715
C	-7.155	-4.966	3.994
C	-6.302	-5.787	4.895
C	-6.136	-7.143	4.832
C	-5.597	-5.347	6.071
C	-4.990	-6.483	6.639
C	-5.365	-4.101	6.659
N	-5.321	-7.550	5.854
C	-4.168	-6.415	7.765
C	-4.602	-4.041	7.804
C	-3.999	-5.173	8.335
H	-4.642	-5.240	2.383
H	-7.190	-3.758	2.326
H	-7.695	-4.342	4.579
H	-7.802	-5.596	3.538
H	-6.545	-7.740	4.150
H	-5.010	-8.496	6.006
H	-5.743	-3.268	6.258
H	-3.732	-7.235	8.130
H	-4.484	-3.157	8.260
H	-3.426	-5.083	9.153
N	-6.525	-1.907	3.818
C	-6.147	-0.735	4.589
C	-7.077	-0.568	5.786
O	-8.289	-0.457	5.614
C	-6.248	0.509	3.717
C	-5.324	0.602	2.501
C	-5.757	1.755	1.580
C	-3.879	0.813	2.943
H	-7.508	-2.030	3.427
H	-5.211	-0.861	4.932
H	-7.190	0.571	3.376
H	-6.057	1.307	4.294
H	-5.366	-0.254	1.990
H	-5.715	2.612	2.088
H	-5.141	1.795	0.796
H	-6.691	1.590	1.268
H	-3.590	0.044	3.513
H	-3.290	0.872	2.136
H	-3.813	1.662	3.468
N	-6.452	-0.432	6.958
C	-7.124	-0.201	8.226
C	-6.721	1.158	8.771
O	-5.555	1.356	9.136
C	-6.736	-1.339	9.182
C	-7.412	-1.353	10.556
C	-6.902	-2.536	11.363
C	-7.206	-0.079	11.302
H	-5.400	-0.508	6.879
H	-8.113	-0.201	8.072
H	-6.960	-2.202	8.732
H	-5.752	-1.285	9.335
H	-8.396	-1.435	10.431
H	-5.911	-2.459	11.481
H	-7.343	-2.545	12.261
H	-7.113	-3.387	10.880
H	-7.587	0.696	10.785
H	-7.661	-0.120	12.198
H	-6.224	0.086	11.449
N	-7.686	2.092	8.762
C	-7.488	3.447	9.243
C	-8.124	3.575	10.627
O	-9.272	3.181	10.870
C	-8.035	4.529	8.299
C	-7.745	5.929	8.793
O	-7.433	4.389	7.003
H	-8.598	1.748	8.372
H	-6.509	3.619	9.332
H	-9.042	4.400	8.196
H	-7.809	5.961	9.794
H	-6.820	6.206	8.517
H	-8.407	6.575	8.404
H	-7.983	3.776	6.428
N	-7.341	4.165	11.533
C	-7.781	4.473	12.890
C	-7.950	5.982	12.968
O	-7.091	6.728	12.472
C	-6.744	3.949	13.903
C	-6.571	2.431	13.811
C	-7.052	4.402	15.318

C	-5.207	2.003	13.273
H	-6.379	4.388	11.183
H	-8.685	4.079	12.987
H	-5.864	4.374	13.689
H	-6.693	2.040	14.719
H	-7.275	2.070	13.204
H	-6.304	4.138	15.926
H	-7.157	5.395	15.338
H	-7.899	3.973	15.631
H	-4.496	2.351	13.878
H	-5.170	1.007	13.242
H	-5.088	2.376	12.357
N	-9.084	6.433	13.521
C	-9.277	7.842	13.821
C	-8.143	8.377	14.699
O	-7.871	7.806	15.735
H	-9.792	5.699	13.709
H	-9.306	8.371	12.965
H	-10.152	7.970	14.302
N	-7.425	9.407	14.243
C	-6.283	9.921	14.990
C	-6.404	11.442	15.067
O	-7.193	12.042	14.345
C	-4.900	9.573	14.394
C	-4.663	8.101	14.123
O	-4.719	10.314	13.190
H	-7.744	9.794	13.339
H	-6.315	9.556	15.917
H	-4.180	9.853	15.057
H	-3.680	7.902	14.140
H	-5.119	7.545	14.825
H	-5.030	7.854	13.223
H	-4.363	11.234	13.391
N	-5.586	12.070	15.917
C	-5.335	13.497	15.805
C	-4.490	13.749	14.555
O	-3.804	12.855	14.037
C	-4.645	14.029	17.075
C	-5.339	13.583	18.344
O	-3.304	13.536	17.079
H	-5.162	11.462	16.640
H	-6.205	13.972	15.694
H	-4.615	15.041	17.031
H	-6.297	13.356	18.151
H	-4.882	12.771	18.719
H	-5.311	14.317	19.028
H	-2.694	14.180	17.556
N	-4.476	15.007	14.122
C	-3.688	15.426	12.974
C	-2.203	15.151	13.244
O	-1.488	14.751	12.332
C	-4.002	16.892	12.643
C	-5.477	17.122	12.300
C	-5.974	18.562	12.303
O	-6.793	18.908	13.190
O	-5.592	19.315	11.400
H	-5.076	15.659	14.673
H	-3.973	14.920	12.158
H	-3.797	17.428	13.435
H	-3.475	17.143	11.859
H	-5.638	16.755	11.385
H	-6.028	16.617	12.964
N	-1.768	15.290	14.511
C	-0.388	15.063	14.927
C	0.063	13.617	14.677
O	1.206	13.409	14.269
C	-0.241	15.406	16.395
H	-2.525	15.579	15.181
H	0.217	15.679	14.399
H	0.712	15.286	16.677
H	-0.513	16.357	16.548
H	-0.824	14.805	16.942
N	-0.829	12.658	14.980
C	-0.608	11.218	14.840
C	-1.121	10.631	13.506
O	-1.062	9.427	13.322
C	-1.267	10.461	15.995
C	-0.603	10.770	17.319
H	-1.746	13.053	15.345
H	0.396	11.065	14.854
H	-2.220	10.733	16.044
H	-1.188	9.488	15.817
N	-1.322	10.545	18.400
O	0.552	11.185	17.352
H	-2.254	10.182	18.324
H	-0.941	10.734	19.310
N	-1.518	11.454	12.536
C	-2.084	10.963	11.278
C	-1.088	10.086	10.503
O	-1.468	9.094	9.874
C	-2.560	12.133	10.426
H	-1.391	12.465	12.746
H	-2.901	10.407	11.485
H	-2.659	11.836	9.477
H	-3.442	12.456	10.766
H	-1.892	12.875	10.476
N	0.200	10.421	10.580
C	1.247	9.642	9.944
C	1.192	8.175	10.365
O	1.567	7.322	9.576
C	2.635	10.254	10.201
C	3.113	10.087	11.608
C	2.830	10.908	12.669
C	3.850	8.970	12.146
C	4.037	9.232	13.526
C	4.442	7.824	11.584
N	3.402	10.415	13.811
C	4.734	8.353	14.359
C	5.152	6.969	12.404
C	5.307	7.241	13.768
H	0.385	11.287	11.136
H	1.096	9.675	8.947
H	3.293	9.809	9.606
H	2.592	11.228	10.013
H	2.286	11.744	12.613
H	3.366	10.846	14.715
H	4.349	7.632	10.610
H	4.810	8.531	15.340
H	5.560	6.147	12.015
H	5.847	6.612	14.329
N	0.689	7.857	11.569
C	0.604	6.474	11.995
C	-0.850	5.967	12.116
O	-1.133	5.065	12.902
C	1.398	6.310	13.290
C	0.885	7.090	14.482
C	1.584	6.683	15.752
C	1.060	7.474	16.924
N	2.089	7.549	17.980
H	0.382	8.663	12.135
H	1.083	5.887	11.321
H	1.403	5.332	13.530
H	2.347	6.596	13.110
H	1.039	8.068	14.322
H	-0.099	6.926	14.580
H	1.418	5.717	15.919
H	2.558	6.857	15.660
H	0.827	8.395	16.620
H	0.246	7.024	17.286
H	2.802	6.867	17.806
H	2.501	8.462	17.985
H	1.672	7.370	18.873
N	-1.765	6.455	11.255
C	-3.182	6.108	11.339
C	-3.549	4.885	10.493
O	-4.654	4.384	10.648
C	-4.004	7.289	10.903
O	-3.752	7.534	9.522
H	-1.370	7.093	10.534
H	-3.393	5.888	12.306
H	-4.964	7.088	11.017
H	-3.738	8.089	11.418
H	-2.921	8.079	9.431
N	-2.695	4.439	9.547
C	-3.141	3.446	8.559
C	-2.239	2.223	8.505
O	-1.055	2.314	8.203
C	-3.266	4.042	7.144
C	-3.996	3.139	6.162
O	-4.037	5.226	7.274
H	-1.755	4.839	9.583
H	-4.074	3.145	8.815
H	-2.344	4.289	6.814
H	-3.442	2.325	5.972
H	-4.876	2.854	6.550
H	-4.160	3.631	5.304
H	-3.721	5.773	8.061
N	-2.821	1.046	8.787
C	-2.128	-0.222	8.605
C	-2.478	-0.803	7.229
O	-3.572	-0.587	6.731
C	-2.559	-1.217	9.683
C	-2.202	-0.879	11.121
C	-2.948	-1.800	12.102
C	-0.709	-1.051	11.304
H	-3.795	1.126	9.141
H	-1.142	-0.061	8.634
H	-3.555	-1.305	9.633
H	-2.138	-2.099	9.467
H	-2.455	0.069	11.313
H	-2.693	-2.747	11.919
H	-2.695	-1.556	13.035
H	-3.930	-1.683	11.976
H	-0.218	-0.439	10.681
H	-0.456	-0.832	12.248
H	-0.451	-1.999	11.105
N	-1.562	-1.618	6.700
C	-1.720	-2.296	5.433
C	-1.406	-3.774	5.625
O	-0.507	-4.144	6.361
C	-0.849	-1.638	4.340
C	0.634	-1.747	4.627
C	-1.190	-2.219	2.982
H	-0.700	-1.722	7.302
H	-2.675	-2.220	5.147
H	-1.077	-0.660	4.323
H	1.012	-0.836	4.802
H	0.781	-2.325	5.431
H	1.101	-2.152	3.839
H	-0.554	-2.962	2.768
H	-2.126	-2.574	2.996
H	-1.114	-1.505	2.286
N	-2.168	-4.622	4.941
C	-1.886	-6.042	4.947
C	-2.587	-6.736	3.791
O	-3.023	-6.113	2.827
H	-2.952	-4.187	4.424
H	-0.892	-6.191	4.869
H	-2.197	-6.447	5.815
N	-2.596	-8.065	3.864
C	-3.177	-8.862	2.787
C	-3.918	-10.039	3.402
O	-3.437	-10.618	4.365
C	-2.093	-9.316	1.813
C	-0.903	-9.933	2.459
C	-0.548	-11.238	2.663
N	0.113	-9.169	3.002
C	1.052	-9.987	3.478
N	0.673	-11.236	3.290
H	-2.173	-8.464	4.713
H	-3.834	-8.298	2.293
H	-2.491	-9.988	1.172
H	-1.785	-8.519	1.274
H	0.140	-8.179	3.035
H	-1.080	-12.036	2.401
H	1.906	-9.693	3.904
N	-5.106	-10.346	2.882
C	-5.880	-11.470	3.394
C	-6.276	-12.318	2.184
O	-6.655	-11.771	1.140
C	-7.162	-11.021	4.095
C	-7.081	-10.410	5.481
O	-5.950	-10.390	6.079
O	-8.142	-9.897	5.950
H	-5.418	-9.735	2.110

H -5.273 -12.023 3.948
H -7.627 -10.348 3.478
H -7.785 -11.832 4.142
N -6.182 -13.650 2.313
C -6.720 -14.526 1.285
C -7.907 -15.302 1.858
O -7.778 -15.932 2.912
C -5.707 -15.542 0.764
C -6.264 -16.422 -0.335
O -4.562 -14.856 0.267
H -5.715 -13.974 3.169
H -7.050 -13.968 0.523
H -5.410 -16.142 1.536
H -5.925 -17.361 -0.229
H -7.267 -16.431 -0.293
H -5.979 -16.073 -1.232
H -4.609 -14.769 -0.735
N -9.026 -15.281 1.133
C -10.259 -15.933 1.572
C -10.503 -17.191 0.738
O -10.375 -17.207 -0.497
C -11.442 -14.968 1.497
C -11.310 -13.750 2.361
C -10.593 -12.636 1.934
C -11.904 -13.696 3.595
C -10.498 -11.532 2.755
C -11.810 -12.585 4.415
C -11.068 -11.508 3.997
H -8.945 -14.763 0.232
H -10.111 -16.237 2.514
H -11.528 -14.669 0.554
H -12.254 -15.462 1.784
H -10.156 -12.637 1.046
H -12.435 -14.497 3.925
H -9.982 -10.714 2.415
H -12.284 -12.578 5.307
H -10.946 -10.714 4.594
N -10.900 -18.283 1.425
C -11.261 -19.511 0.727
C -12.652 -19.981 1.220
O -13.159 -19.485 2.215
C -10.180 -20.589 0.918
C -8.804 -20.178 0.440
O -10.013 -20.893 2.300
H -10.921 -18.166 2.447
H -11.331 -19.323 -0.249
H -10.478 -21.443 0.435
H -8.163 -20.946 0.534
H -8.843 -19.903 -0.525
H -8.466 -19.405 0.985
H -10.635 -21.636 2.576
N -13.274 -20.902 0.477
C -14.636 -21.355 0.739
C -14.642 -22.496 1.759
O -15.699 -22.853 2.270
C -15.323 -21.816 -0.550
C -15.666 -20.726 -1.562
C -17.146 -20.658 -1.898
C -17.920 -19.979 -0.783
N -19.291 -20.524 -0.592
H -12.694 -21.272 -0.314
H -15.171 -20.589 1.112
H -14.719 -22.483 -1.004
H -16.177 -22.287 -0.297
H -15.377 -19.835 -1.189
H -15.147 -20.896 -2.409
H -17.263 -20.134 -2.733
H -17.494 -21.581 -2.010
H -17.403 -20.080 0.080
H -17.986 -18.991 -0.987
H -19.420 -20.767 0.369
H -19.963 -19.832 -0.855
H -19.407 -21.337 -1.162
N -13.451 -23.028 2.050
C -13.211 -24.006 3.102
C -12.180 -23.410 4.064
O -11.284 -22.713 3.609
C -12.721 -25.333 2.481
C -13.763 -25.940 1.536
C -11.371 -25.178 1.773
H -12.673 -22.665 1.428
H -14.056 -24.142 3.607
H -12.590 -25.986 3.224
H -13.371 -26.022 0.622
H -14.026 -26.842 1.872
H -14.565 -25.346 1.506
H -10.971 -26.083 1.635
H -11.515 -24.732 0.892
H -10.767 -24.622 2.341
N -12.279 -23.683 5.372
C -11.373 -23.072 6.349
C -9.958 -23.634 6.174
O -9.026 -22.912 6.577
C -11.794 -23.313 7.805
C -13.283 -23.541 8.006
C -13.770 -23.245 9.394
C -15.258 -23.490 9.524
N -15.783 -22.946 10.800
O -9.552 -24.661 5.712
H -13.032 -24.346 5.623
H -11.347 -22.083 6.194
H -11.303 -24.118 8.139
H -11.522 -22.513 8.342
H -13.789 -22.961 7.346
H -13.494 -24.507 7.779
H -13.295 -23.836 10.043
H -13.588 -22.288 9.610
H -15.731 -23.049 8.757
H -15.432 -24.478 9.490
H -15.212 -23.263 11.558
H -15.769 -21.946 10.767
H -16.723 -23.261 10.936

Ir(*S*)-*pro-R* with Sav S112I

N 52.818 -12.396 12.098
C 52.905 -11.169 11.250
C 52.919 -9.893 12.086
O 52.623 -8.824 11.554
C 51.707 -10.963 10.328
C 51.102 -12.223 9.754
O 51.779 -12.882 8.929
O 49.953 -12.514 10.138
H 52.775 -12.346 13.100
H 53.786 -11.223 10.764
H 50.998 -10.476 10.851
H 52.002 -10.373 9.568
N 53.236 -10.020 13.380
C 53.502 -8.890 14.250
C 54.491 -7.902 13.619
O 54.220 -6.702 13.633
C 54.010 -9.428 15.582
C 54.372 -8.362 16.577
C 54.579 -8.947 17.960
O 54.985 -8.196 18.868
O 54.329 -10.164 18.116
H 53.274 -11.017 13.707
H 52.640 -8.404 14.457
H 53.291 -9.979 15.974
H 54.819 -9.962 15.399
H 55.216 -7.949 16.304
H 53.635 -7.721 16.638
N 55.624 -8.387 13.075
C 56.634 -7.502 12.493
C 56.199 -7.009 11.109
O 56.467 -5.856 10.742
C 57.981 -8.188 12.423
H 55.710 -9.417 13.107
H 56.750 -6.703 13.094
H 58.166 -8.659 13.287
H 57.982 -8.856 11.677
H 58.698 -7.509 12.258
N 55.545 -7.901 10.344
C 54.923 -7.544 9.082
C 53.898 -6.402 9.208
O 53.882 -5.502 8.374
H 55.529 -8.866 10.743
H 55.637 -7.265 8.416
H 54.455 -8.356 8.690
N 53.029 -6.446 10.227
C 51.989 -5.426 10.388
C 52.565 -4.145 10.992
O 52.186 -3.043 10.594
C 50.802 -5.929 11.230
C 50.040 -7.043 10.517
C 49.856 -4.782 11.600
C 49.166 -7.842 11.460
H 53.155 -7.244 10.872
H 51.679 -5.192 9.462
H 51.167 -6.300 12.083
H 49.470 -6.637 9.812
H 50.701 -7.656 10.097
H 49.142 -5.127 12.205
H 50.373 -4.067 12.064
H 49.444 -4.415 10.770
H 48.498 -7.234 11.886
H 48.691 -8.555 10.947
H 49.736 -8.260 12.165
N 53.436 -4.278 11.998
C 54.129 -3.130 12.569
C 54.811 -2.322 11.463
O 55.483 -2.865 10.588
C 55.097 -3.610 13.657
C 55.887 -2.483 14.296
O 54.251 -4.275 14.599
H 53.570 -5.253 12.327
H 53.455 -2.538 13.019
H 55.725 -4.281 13.263
H 55.326 -1.652 14.336
H 56.157 -2.740 15.227
H 56.712 -2.295 13.757
H 54.164 -5.250 14.365
N 54.581 -1.011 11.451
C 55.228 -0.162 10.468
C 54.389 1.069 10.141
O 53.380 1.353 10.805
H 53.929 -0.672 12.173
H 56.129 0.136 10.817
H 55.388 -0.687 9.619
N 54.826 1.773 9.092
C 54.168 2.951 8.580
C 53.554 2.601 7.230
O 54.230 2.144 6.313
C 55.109 4.154 8.472
C 54.417 5.325 7.819
O 55.552 4.516 9.784
H 55.710 1.380 8.668
H 53.431 3.210 9.200
H 55.925 3.889 7.923
H 53.435 5.137 7.722
H 54.534 6.152 8.377
H 54.805 5.493 6.908
H 54.797 4.437 10.443
N 52.237 2.826 7.132
C 51.454 2.462 5.967
C 50.778 3.706 5.433
O 50.520 4.634 6.205
C 50.394 1.423 6.313
C 50.851 0.121 6.862
C 51.169 -0.170 8.163
C 51.022 -1.082 6.123
C 51.442 -2.062 7.043
C 50.832 -1.433 4.781
N 51.514 -1.477 8.270
C 51.662 -3.382 6.662
C 51.094 -2.727 4.402
C 51.497 -3.686 5.330
H 51.826 3.291 7.978
H 52.083 2.131 5.264
H 49.760 1.826 6.992
H 49.857 1.222 5.478
H 51.147 0.489 8.913
H 51.782 -1.946 9.119
H 50.514 -0.761 4.118
H 51.929 -4.072 7.336

H	50.994	-2.984	3.441
H	51.670	-4.623	5.014
N	50.518	3.702	4.121
C	49.794	4.758	3.441
C	48.658	4.155	2.603
O	48.779	3.118	1.955
C	50.734	5.535	2.482
C	52.018	5.999	3.115
C	52.065	7.214	3.782
C	53.170	5.215	3.102
C	53.214	7.646	4.413
C	54.338	5.640	3.727
C	54.355	6.861	4.370
O	55.431	7.398	4.979
H	50.892	2.852	3.623
H	49.381	5.361	4.127
H	50.952	4.931	1.719
H	50.234	6.332	2.151
H	51.243	7.795	3.806
H	53.157	4.332	2.637
H	53.226	8.521	4.901
H	55.154	5.066	3.708
H	55.697	6.832	5.763
N	47.532	4.859	2.540
C	46.474	4.424	1.644
C	46.528	5.230	0.351
O	47.371	6.121	0.199
C	45.092	4.462	2.318
C	44.592	5.838	2.657
H	47.484	5.683	3.143
H	46.660	3.469	1.401
H	44.435	4.020	1.697
H	45.148	3.918	3.163
N	43.493	5.908	3.419
O	45.111	6.825	2.133
H	43.060	5.063	3.732
H	43.116	6.793	3.669
N	45.554	4.962	-0.531
C	45.460	5.559	-1.861
C	45.114	7.047	-1.803
O	45.287	7.763	-2.794
C	44.483	4.743	-2.726
C	42.984	4.847	-2.354
C	42.543	4.298	-1.003
H	44.846	4.267	-0.171
H	46.364	5.472	-2.306
H	44.576	5.039	-3.686
H	44.739	3.768	-2.672
H	42.722	5.831	-2.401
H	42.452	4.372	-3.083
N	41.440	4.843	-0.477
O	43.123	3.361	-0.450
H	41.080	4.518	0.413
H	40.974	5.587	-0.987
N	44.668	7.544	-0.645
C	44.414	8.965	-0.447
C	45.660	9.683	0.052
O	45.621	10.903	0.201
C	43.308	9.146	0.597
C	41.930	8.594	0.238
C	40.999	8.691	1.446
C	41.372	9.322	-0.968
H	44.519	6.827	0.099
H	44.146	9.370	-1.330
H	43.603	8.689	1.435
H	43.204	10.124	0.764
H	42.020	7.639	-0.032
H	40.910	9.648	1.720
H	40.101	8.328	1.202
H	41.381	8.160	2.202
H	41.986	9.200	-1.750
H	40.469	8.954	-1.197
H	41.288	10.299	-0.764
N	46.672	8.920	0.444
C	47.876	9.487	1.035
C	47.785	9.698	2.550
O	48.630	10.360	3.148
H	46.533	7.900	0.295
H	48.668	8.878	0.836
H	48.081	10.381	0.592
N	46.796	9.075	3.198
C	46.729	9.077	4.653
C	47.850	8.223	5.214
O	48.216	7.254	4.566
C	45.365	8.560	5.097
O	44.351	9.401	4.559
H	46.104	8.606	2.589
H	46.866	10.018	4.970
H	45.238	7.643	4.753
H	45.314	8.587	6.083
H	44.309	9.276	3.570
N	48.262	8.512	6.459
C	49.386	7.830	7.092
C	48.897	7.069	8.325
O	48.239	7.661	9.164
C	50.479	8.814	7.531
C	51.702	8.075	8.036
O	50.808	9.628	6.399
H	47.709	9.262	6.926
H	49.766	7.167	6.444
H	50.109	9.415	8.258
H	52.384	8.730	8.371
H	51.445	7.459	8.786
H	52.107	7.534	7.294
H	50.055	9.621	5.732
N	49.149	5.764	8.335
C	48.728	4.832	9.377
C	50.002	4.269	10.006
O	50.679	3.443	9.386
C	47.872	3.763	8.699
C	47.483	2.534	9.467
C	47.073	2.612	10.782
C	47.483	1.294	8.854
C	46.658	1.480	11.469
C	47.099	0.158	9.543
C	46.675	0.249	10.850
H	49.705	5.441	7.488
H	48.274	5.363	10.079
H	47.018	4.212	8.390
H	48.370	3.459	7.871
H	47.071	3.493	11.253
H	47.765	1.217	7.893
H	46.346	1.570	12.426
H	47.134	-0.745	9.075
H	46.382	-0.566	11.347
N	50.306	4.699	11.234
C	51.496	4.232	11.931
C	51.040	3.280	13.024
O	50.340	3.697	13.935
C	52.303	5.404	12.520
C	52.753	6.388	11.440
C	53.478	4.911	13.368
C	53.410	7.627	11.984
H	49.633	5.379	11.634
H	52.011	3.690	11.277
H	51.709	5.895	13.158
H	53.392	5.917	10.833
H	51.948	6.654	10.908
H	53.912	5.690	13.814
H	53.142	4.274	14.058
H	54.139	4.451	12.780
H	54.222	7.373	12.510
H	53.678	8.224	11.228
H	52.769	8.113	12.579
N	51.398	2.015	12.898
C	50.845	1.010	13.786
C	51.927	0.111	14.378
O	52.934	-0.214	13.746
C	49.783	0.174	13.059
C	50.370	-0.675	11.944
C	48.978	-0.665	14.052
H	52.074	1.815	12.147
H	50.384	1.482	14.553
H	49.134	0.810	12.631
H	50.212	-0.228	11.063
H	51.354	-0.785	12.088
H	49.933	-1.575	11.942
H	48.756	-1.544	13.632
H	49.525	-0.811	14.875
H	48.138	-0.178	14.285
N	51.637	-0.324	15.602
C	52.376	-1.355	16.314
C	51.425	-2.508	16.658
O	50.411	-2.306	17.320
C	52.966	-0.793	17.613
C	53.672	-1.834	18.457
O	53.882	0.218	17.214
H	50.794	0.161	16.027
H	53.093	-1.712	15.716
H	52.228	-0.366	18.149
H	54.458	-1.421	18.926
H	53.041	-2.205	19.144
H	53.997	-2.585	17.875
H	53.848	0.355	16.216
N	51.754	-3.701	16.176
C	51.083	-4.921	16.573
C	51.803	-5.531	17.783
O	53.021	-5.663	17.753
C	51.082	-5.840	15.408
H	52.542	-3.679	15.484
H	50.142	-4.710	16.842
H	50.227	-5.747	14.885
H	51.853	-5.634	14.795
H	51.165	-6.796	15.712
N	51.059	-5.826	18.848
C	51.582	-6.494	20.033
C	51.378	-8.005	19.956
O	50.669	-8.501	19.076
H	50.054	-5.533	18.758
H	52.567	-6.297	20.124
H	51.116	-6.139	20.854
N	52.030	-8.746	20.865
C	52.022	-10.207	20.826
C	50.621	-10.758	21.119
O	50.269	-11.855	20.692
C	53.032	-10.740	21.825
H	52.533	-8.194	21.587
H	52.296	-10.510	19.906
H	52.757	-11.653	22.127
H	53.934	-10.792	21.396
H	53.077	-10.130	22.616
N	49.845	-9.947	21.853
C	48.563	-10.268	22.464
C	47.367	-9.939	21.557
O	46.240	-9.923	22.046
C	48.430	-9.428	23.743
C	48.324	-7.919	23.519
O	49.009	-7.381	22.603
O	47.549	-7.282	24.268
H	50.283	-8.968	21.959
H	48.543	-11.270	22.617
H	47.608	-9.733	24.227
H	49.233	-9.607	24.313
N	47.621	-9.605	20.280
C	46.563	-9.256	19.341
C	46.193	-7.768	19.328
O	45.273	-7.395	18.626
H	48.632	-9.619	20.030
H	46.844	-9.531	18.399
H	45.727	-9.799	19.558
N	46.898	-6.901	20.066
C	46.575	-5.475	20.110
C	47.160	-4.746	18.906
O	48.247	-5.067	18.460
C	47.110	-4.835	21.358
H	47.684	-7.327	20.597
H	45.574	-5.370	20.094
H	47.998	-5.236	21.598
H	47.228	-3.849	21.216
H	46.473	-4.979	22.120
N	46.434	-3.741	18.397
C	46.984	-2.748	17.477
C	46.916	-1.399	18.190
O	45.907	-1.074	18.801
C	46.165	-2.702	16.172

C	46.093	-3.993	15.360
C	45.229	-3.809	14.122
C	47.477	-4.444	14.909
H	45.438	-3.732	18.718
H	47.934	-2.964	17.302
H	45.231	-2.446	16.414
H	46.569	-2.001	15.589
H	45.703	-4.717	15.932
H	45.618	-3.089	13.546
H	45.196	-4.666	13.606
H	44.302	-3.550	14.396
H	48.053	-4.605	15.711
H	47.397	-5.290	14.381
H	47.890	-3.733	14.339
N	47.993	-0.616	18.141
C	47.994	0.721	18.721
C	48.772	1.629	17.789
O	49.658	1.177	17.065
C	48.651	0.696	20.112
C	47.919	-0.183	21.098
O	49.983	0.201	19.938
H	48.814	-1.035	17.663
H	47.055	1.047	18.773
H	48.705	1.641	20.466
H	48.549	-0.860	21.492
H	47.537	0.375	21.841
H	47.169	-0.668	20.638
H	50.015	-0.460	19.181
N	48.480	2.915	17.801
C	49.261	3.804	16.958
C	48.546	5.105	16.650
O	47.756	5.589	17.446
H	47.717	3.206	18.407
H	50.146	4.009	17.415
H	49.484	3.331	16.086
N	48.874	5.673	15.484
C	48.353	6.972	15.107
C	47.919	6.935	13.641
O	48.444	6.204	12.786
C	49.339	8.133	15.347
C	49.787	8.291	16.787
O	50.454	7.996	14.466
H	49.516	5.111	14.896
H	47.547	7.174	15.665
H	48.867	9.011	15.134
H	49.761	9.259	17.050
H	49.179	7.772	17.394
H	50.724	7.949	16.894
H	50.400	8.671	13.721
N	46.934	7.767	13.363
C	46.378	7.884	12.025
C	46.301	9.360	11.659
O	45.825	10.189	12.434
C	44.967	7.287	11.945
C	44.518	6.967	10.541
C	44.021	7.943	9.689
C	44.616	5.678	10.066
C	43.616	7.636	8.395
C	44.251	5.354	8.776
C	43.734	6.334	7.953
O	43.416	6.059	6.676
H	46.592	8.327	14.174
H	46.997	7.435	11.382
H	44.956	6.445	12.479
H	44.329	7.946	12.336
H	43.941	8.966	10.038
H	44.963	4.954	10.672
H	43.249	8.346	7.800
H	44.359	4.419	8.438
H	43.708	6.812	6.080
N	46.751	9.668	10.449
C	46.693	11.025	9.937
C	45.981	10.998	8.597
O	46.452	10.366	7.659
C	48.090	11.602	9.781
C	48.073	12.985	9.174
C	49.412	13.707	9.205
O	49.449	14.842	8.697
O	50.399	13.148	9.742
H	47.140	8.867	9.912
H	46.192	11.608	10.572
H	48.489	11.670	10.672
H	48.593	11.016	9.180
H	47.804	12.907	8.223
H	47.428	13.543	9.679
N	44.801	11.758	8.514
C	44.007	11.699	7.330
C	44.487	12.730	6.389
O	44.390	13.942	6.721
C	42.542	11.861	7.702
O	41.742	11.667	6.578
H	44.710	12.588	9.147
H	44.207	10.739	6.857
H	42.275	11.094	8.458
H	42.386	12.866	8.160
H	41.024	12.350	6.631
N	44.631	12.408	5.107
C	44.898	13.418	4.081
C	43.620	14.105	3.588
O	43.715	15.049	2.816
C	45.653	12.787	2.929
H	44.539	11.394	4.898
H	45.508	14.124	4.473
H	46.123	13.498	2.404
H	46.328	12.138	3.283
H	45.013	12.304	2.330
N	42.319	13.760	4.090
C	41.074	14.378	3.581
C	40.127	14.805	4.684
O	39.728	13.980	5.546
C	40.361	13.455	2.563
C	41.180	13.310	1.274
C	40.022	12.068	3.116
H	42.256	12.909	4.677
H	41.331	15.312	3.038
H	39.410	13.956	2.282
H	41.542	14.210	1.028
H	41.941	12.668	1.386
H	40.580	12.985	0.541
H	39.541	11.457	2.328
H	40.931	11.526	3.448
H	39.310	12.141	3.959
N	39.440	16.058	4.586
C	37.986	16.164	4.807
C	37.646	17.093	5.942
O	37.039	18.167	5.691
H	39.960	16.860	4.164
H	37.510	16.542	3.879
H	37.544	15.172	5.032
N	37.868	16.715	7.235
C	37.694	17.613	8.393
C	38.602	17.167	9.530
O	38.119	16.623	10.560
C	36.204	17.669	8.807
C	35.930	18.677	9.880
H	38.206	15.738	7.393
H	38.019	18.642	8.112
H	35.584	17.936	7.931
H	35.882	16.674	9.174
N	34.707	18.768	10.428
O	36.816	19.472	10.273
H	33.931	18.157	10.114
H	34.573	19.476	11.177
N	40.012	17.138	9.318
C	40.906	16.298	10.124
C	42.318	16.839	10.087
O	42.836	17.128	8.977
C	40.868	14.866	9.599
H	40.368	17.497	8.403
H	40.555	16.292	11.177
H	41.091	14.847	8.510
H	41.604	14.233	10.139
H	39.863	14.444	9.776
N	42.984	17.121	11.242
C	44.294	17.786	11.275
C	45.268	17.046	12.154
O	44.914	16.802	13.311
C	44.111	19.225	11.709
C	45.355	20.094	11.646
C	45.192	21.525	12.135
O	46.244	22.189	12.269
O	44.028	21.989	12.399
H	42.523	16.936	12.156
H	44.727	17.828	10.298
H	43.300	19.696	11.109
H	43.827	19.176	12.649
H	46.059	19.669	12.208
H	45.655	20.140	10.698
N	46.461	16.693	11.641
C	47.503	16.055	12.419
C	47.080	14.610	12.768
O	46.272	14.000	12.060
C	47.849	16.906	13.634
O	49.075	16.488	14.218
H	46.561	16.925	10.623
H	48.331	15.995	11.861
H	47.936	17.857	13.351
H	47.124	16.815	14.311
H	48.902	16.141	15.138
N	47.670	14.053	13.826
C	47.549	12.633	14.147
C	46.443	12.378	15.171
O	46.229	13.154	16.110
C	48.873	12.129	14.726
C	49.999	12.061	13.717
C	51.325	11.787	14.415
N	52.372	11.637	13.427
C	53.629	11.278	13.694
N	54.438	10.941	12.700
N	54.042	11.188	14.951
H	48.227	14.722	14.407
H	47.326	12.127	13.303
H	49.146	12.747	15.458
H	48.724	11.214	15.089
H	49.831	11.323	13.074
H	50.076	12.929	13.241
H	51.525	12.556	15.003
H	51.229	10.951	14.933
H	52.140	11.817	12.457
H	54.140	10.967	11.752
H	55.384	10.651	12.909
H	53.408	11.411	15.697
H	54.975	10.903	15.156
N	45.791	11.220	15.033
C	44.735	10.772	15.933
C	45.135	9.401	16.491
O	45.743	8.586	15.790
C	43.386	10.665	15.186
C	42.885	12.020	14.760
C	43.296	12.609	13.546
C	42.005	12.685	15.541
C	42.752	13.788	13.166
C	41.492	13.864	15.134
C	41.830	14.394	13.940
O	41.188	15.536	13.475
H	46.111	10.652	14.206
H	44.674	11.409	16.696
H	43.514	10.048	14.415
H	42.717	10.288	15.820
H	44.024	12.115	12.910
H	41.683	12.258	16.485
H	43.044	14.245	12.230
H	40.789	14.389	15.757
H	40.430	15.829	14.084
N	44.755	9.129	17.747
C	44.974	7.824	18.346
C	44.115	6.785	17.621
O	42.969	7.072	17.320
C	44.640	7.838	19.845
C	44.763	6.437	20.420
C	45.537	8.819	20.616
H	44.300	9.922	18.239
H	45.933	7.571	18.216
H	43.689	8.110	19.942
H	44.008	5.871	20.088

H	45.632	6.032	20.134
H	44.731	6.480	21.419
H	45.376	8.704	21.593
H	46.491	8.621	20.401
H	45.310	9.750	20.339
N	44.695	5.610	17.364
C	43.961	4.448	16.906
C	44.208	3.277	17.849
O	45.268	3.171	18.452
C	44.336	4.052	15.477
C	45.701	3.404	15.250
C	45.691	1.865	15.362
C	46.209	3.811	13.879
H	45.732	5.607	17.525
H	42.980	4.658	16.915
H	43.642	3.405	15.148
H	44.302	4.880	14.912
H	46.358	3.771	15.927
H	45.067	1.495	14.679
H	46.613	1.523	15.202
H	45.387	1.609	16.276
H	46.295	4.808	13.834
H	47.104	3.394	13.714
H	45.566	3.502	13.176
N	43.186	2.427	17.987
C	43.371	1.154	18.659
C	42.614	0.105	17.860
O	41.610	0.442	17.224
C	42.960	1.288	20.123
C	41.485	1.489	20.315
O	43.335	0.101	20.818
H	42.292	2.750	17.587
H	44.338	0.921	18.648
H	43.461	2.073	20.539
H	41.314	2.110	21.088
H	41.075	1.888	19.487
H	41.033	0.610	20.504
H	44.083	-0.369	20.336
N	43.070	-1.151	17.937
C	42.339	-2.259	17.355
C	42.908	-3.609	17.742
O	43.613	-3.712	18.749
H	43.972	-1.251	18.440
H	41.366	-2.211	17.646
H	42.346	-2.172	16.342
N	42.556	-4.613	16.929
C	42.869	-6.013	17.174
C	43.241	-6.673	15.861
O	42.712	-6.287	14.822
C	41.660	-6.730	17.784
C	41.144	-6.133	19.079
C	42.040	-6.214	20.325
N	42.267	-7.599	20.690
C	43.223	-8.050	21.499
N	44.023	-7.218	22.133
N	43.366	-9.348	21.677
H	42.021	-4.291	16.078
H	43.650	-6.067	17.797
H	40.918	-6.709	17.114
H	41.920	-7.679	17.961
H	40.927	-5.142	18.930
H	40.253	-6.577	19.323
H	42.899	-5.777	20.105
H	41.574	-5.742	21.059
H	41.638	-8.298	20.292
H	43.917	-6.228	22.036
H	44.754	-7.576	22.727
H	42.746	-9.993	21.222
H	44.094	-9.701	22.269
N	44.104	-7.704	15.920
C	44.380	-8.573	14.793
C	44.472	-10.013	15.326
O	44.679	-10.219	16.533
C	45.669	-8.157	14.062
C	46.946	-8.517	14.790
C	47.359	-7.840	15.931
C	47.765	-9.531	14.299
C	48.509	-8.213	16.612
C	48.951	-9.869	14.926
C	49.314	-9.224	16.093
O	50.488	-9.591	16.666
H	44.556	-7.816	16.859
H	43.616	-8.533	14.158
H	45.678	-8.607	13.171
H	45.648	-7.166	13.939
H	46.818	-7.067	16.270
H	47.486	-10.026	13.474
H	48.760	-7.763	17.468
H	49.543	-10.576	14.537
H	50.553	-9.196	17.584
N	44.303	-10.946	14.413
C	44.489	-12.380	14.630
C	45.985	-12.694	14.714
O	46.681	-12.738	13.692
C	43.819	-13.170	13.507
C	43.860	-14.692	13.643
O	44.353	-15.177	14.678
O	43.446	-15.356	12.661
H	44.008	-10.563	13.465
H	44.094	-12.600	15.522
H	42.855	-12.886	13.460
H	44.267	-12.920	12.642
N	46.443	-12.928	15.953
C	47.855	-13.174	16.245
C	48.274	-14.629	15.987
O	49.450	-14.939	16.070
C	48.191	-12.728	17.651
O	47.310	-13.283	18.606
H	45.701	-12.919	16.689
H	48.408	-12.584	15.635
H	49.125	-13.013	17.871
H	48.131	-11.729	17.700
H	46.571	-13.768	18.139
N	47.341	-15.510	15.615
C	47.674	-16.877	15.248
C	46.910	-17.280	13.987
O	46.062	-18.164	13.999
C	47.344	-17.783	16.400
H	46.371	-15.133	15.612
H	48.657	-16.935	15.056
H	48.085	-18.444	16.541
H	47.225	-17.245	17.238
H	46.495	-18.283	16.212
N	47.182	-16.656	12.837
C	46.347	-16.885	11.649
C	46.567	-18.242	10.962
O	47.527	-18.944	11.266
C	46.779	-15.741	10.729
C	48.241	-15.524	11.104
C	48.319	-15.760	12.603
H	45.359	-16.765	11.881
H	46.704	-16.001	9.772
H	46.252	-14.917	10.906
H	48.821	-16.172	10.609
H	48.517	-14.590	10.874
H	49.177	-16.199	12.851
H	48.211	-14.907	13.106
N	45.680	-18.572	10.009
C	45.725	-19.819	9.259
C	46.976	-19.865	8.387
O	47.517	-18.837	7.972
C	44.469	-19.958	8.427
H	44.943	-17.838	9.854
H	45.758	-20.588	9.905
H	44.673	-20.464	7.587
H	43.771	-20.453	8.947
H	44.119	-19.051	8.187
N	47.419	-21.086	8.086
C	48.684	-21.300	7.398
C	48.452	-21.741	5.948
O	49.325	-22.375	5.357
C	49.502	-22.331	8.191
C	49.987	-21.832	9.538
O	48.611	-23.438	8.347
H	46.789	-21.859	8.383
H	49.194	-20.433	7.382
H	50.282	-22.624	7.637
H	49.542	-20.960	9.761
H	49.767	-22.502	10.251
H	50.981	-21.695	9.515
H	47.802	-23.324	7.759
N	47.303	-21.368	5.360
C	46.861	-21.858	4.059
C	46.978	-20.781	2.967
O	46.443	-20.941	1.867
C	45.437	-22.407	4.173
C	44.406	-21.355	4.560
O	44.807	-20.319	5.157
O	43.219	-21.551	4.225
H	46.745	-20.683	5.930
H	47.481	-22.609	3.792
H	45.182	-22.790	3.289
H	45.436	-23.122	4.867
N	47.700	-19.685	3.251
C	47.831	-18.575	2.315
C	46.742	-17.510	2.507
O	46.689	-16.558	1.742
H	48.151	-19.703	4.189
H	48.738	-18.140	2.431
H	47.782	-18.925	1.366
N	45.878	-17.695	3.513
C	44.876	-16.723	3.941
C	45.526	-15.471	4.516
O	46.602	-15.523	5.092
C	43.938	-17.316	4.966
O	42.944	-18.093	4.327
H	45.988	-18.637	3.986
H	44.340	-16.450	3.131
H	44.454	-17.899	5.585
H	43.496	-16.581	5.470
H	43.176	-19.062	4.404
N	44.828	-14.334	4.424
C	45.295	-13.137	5.081
C	45.004	-13.164	6.579
O	44.335	-14.066	7.077
H	43.965	-14.397	3.864
H	46.292	-13.036	4.943
H	44.847	-12.326	4.675
N	45.539	-12.150	7.268
C	45.388	-11.968	8.702
C	44.297	-10.930	8.956
O	44.499	-9.770	8.571
C	46.722	-11.549	9.331
C	46.624	-11.325	10.816
O	47.614	-12.619	8.965
H	46.091	-11.482	6.665
H	45.098	-12.833	9.109
H	47.042	-10.711	8.895
H	45.921	-10.635	11.019
H	46.378	-12.181	11.282
H	47.505	-11.003	11.179
H	48.475	-12.542	9.476
N	43.222	-11.378	9.603
C	42.056	-10.544	9.902
C	42.403	-9.527	10.979
O	43.156	-9.813	11.906
C	40.873	-11.381	10.291
H	43.281	-12.387	9.883
H	41.789	-10.053	9.068
H	40.972	-11.700	11.237
H	40.030	-10.842	10.218
H	40.801	-12.178	9.686
N	41.949	-8.278	10.772
C	42.239	-7.220	11.719
C	41.218	-6.097	11.562
O	40.455	-6.030	10.593
C	43.683	-6.719	11.534
C	44.114	-6.243	10.144
C	43.345	-5.010	9.715
C	45.609	-5.953	10.142
H	41.398	-8.157	9.909
H	42.164	-7.585	12.649
H	43.818	-5.951	12.167
H	44.298	-7.468	11.797
H	43.954	-6.984	9.485

H	43.508	-4.267	10.366
H	43.648	-4.723	8.805
H	42.365	-5.216	9.687
H	46.111	-6.786	10.378
H	45.888	-5.642	9.233
H	45.812	-5.240	10.814
N	41.232	-5.210	12.538
C	40.458	-3.988	12.424
C	40.962	-2.969	13.418
O	41.708	-3.317	14.326
H	41.815	-5.451	13.353
H	40.543	-3.621	11.493
H	39.490	-4.185	12.605
N	40.591	-1.706	13.206
C	40.967	-0.628	14.109
C	39.965	0.497	13.996
O	39.204	0.584	13.020
C	42.417	-0.178	13.888
C	42.670	0.656	12.659
C	42.581	2.002	12.492
C	43.037	0.118	11.379
C	43.143	1.193	10.490
C	43.244	-1.186	10.913
N	42.860	2.330	11.190
C	43.475	1.013	9.145
C	43.618	-1.353	9.602
C	43.692	-0.263	8.732
H	40.017	-1.563	12.349
H	40.925	-0.985	15.045
H	42.709	0.367	14.676
H	42.999	-0.990	13.815
H	42.347	2.658	13.208
H	42.857	3.259	10.812
H	43.124	-1.969	11.515
H	43.543	1.802	8.532
H	43.839	-2.266	9.268
H	43.916	-0.443	7.764
N	39.957	1.352	15.024
C	39.129	2.527	15.114
C	39.938	3.805	15.359
O	40.902	3.805	16.137
C	38.157	2.388	16.297
C	37.178	3.536	16.418
O	37.431	1.166	16.186
H	40.640	1.074	15.791
H	38.625	2.644	14.240
H	38.697	2.363	17.165
H	37.668	4.385	16.636
H	36.684	3.659	15.553
H	36.515	3.347	17.147
H	36.710	1.119	16.887
N	39.474	4.886	14.712
C	39.870	6.246	15.002
C	38.668	6.995	15.304
O	37.782	7.178	14.447
C	40.602	6.922	13.821
C	40.905	8.399	14.111
C	41.910	6.200	13.554
H	38.776	4.642	13.954
H	40.470	6.237	15.802
H	39.974	6.876	12.904
H	41.558	8.829	13.323
H	39.972	8.998	14.106
H	41.407	8.500	15.095
H	42.415	6.079	14.411
H	41.723	5.303	13.149
H	42.460	6.744	12.918
N	38.557	7.570	16.559
C	37.672	8.709	16.759
C	38.371	9.967	16.312
O	39.596	10.111	16.581
C	37.231	8.718	18.208
H	39.399	7.451	17.158
H	36.757	8.593	16.154
H	36.394	9.256	18.328
H	37.051	7.771	18.483
H	37.949	9.097	18.795
N	37.730	10.891	15.534
C	38.402	12.067	14.951
C	38.370	13.228	15.912
O	38.073	14.384	15.505
C	37.759	12.438	13.605
C	37.849	11.296	12.648
C	36.842	10.497	12.282
C	38.972	10.867	11.971
C	38.570	9.804	11.220
C	40.289	11.325	11.929
N	37.260	9.577	11.385
C	39.460	9.113	10.410
C	41.163	10.664	11.131
C	40.742	9.542	10.366
H	36.717	10.755	15.349
H	39.472	11.833	14.762
H	36.694	12.714	13.766
H	38.272	13.317	13.155
H	35.827	10.570	12.646
H	36.688	8.863	10.892
H	40.598	12.176	12.517
H	39.130	8.254	9.850
H	42.197	10.985	11.087
H	41.454	9.020	9.741
N	38.818	13.051	17.191
C	39.121	14.126	18.132
C	40.581	14.036	18.565
O	41.073	12.983	18.953
C	38.207	13.987	19.349
C	38.512	14.967	20.487
C	37.616	14.773	21.674
C	37.941	15.746	22.794
N	39.262	15.434	23.368
H	39.036	12.094	17.523
H	38.891	15.106	17.674
H	37.265	14.140	19.055
H	38.299	13.059	19.708
H	39.471	14.839	20.766
H	38.405	15.904	20.135
H	36.667	14.918	21.399
H	37.726	13.844	22.025
H	37.946	16.673	22.424
H	37.241	15.669	23.501
H	39.974	15.574	22.676
H	39.441	16.033	24.151
H	39.282	14.480	23.673
N	41.291	15.172	18.428
C	42.614	15.339	19.017
C	42.666	16.759	19.609
O	41.649	17.411	19.742
C	43.705	15.003	17.987
C	43.755	15.951	16.798
H	40.811	15.907	17.870
H	42.685	14.710	19.779
H	44.592	15.030	18.452
H	43.538	14.075	17.647
N	44.481	15.561	15.761
O	43.062	16.968	16.770
H	44.956	14.678	15.787
H	44.557	16.143	14.951
N	43.867	17.255	19.886
C	44.049	18.608	20.402
C	43.777	19.709	19.375
O	43.628	20.864	19.750
C	45.467	18.797	20.932
C	45.703	17.996	22.188
H	44.659	16.602	19.705
H	43.374	18.735	21.153
H	46.106	18.495	20.234
H	45.600	19.760	21.136
N	46.902	17.451	22.323
O	44.792	17.833	22.999
H	47.598	17.586	21.615
H	47.118	16.903	23.133
N	43.687	19.383	18.094
C	43.481	20.406	17.074
C	42.040	20.485	16.602
O	41.607	21.535	16.129
C	44.336	20.106	15.843
C	45.796	20.061	16.189
C	46.270	19.113	17.085
C	46.686	21.006	15.701
C	47.599	19.075	17.461
C	48.027	20.975	16.057
C	48.472	20.018	16.956
O	49.773	19.941	17.353
H	43.772	18.375	17.886
H	43.732	21.304	17.474
H	44.067	19.225	15.486
H	44.190	20.825	15.181
H	45.631	18.438	17.471
H	46.359	21.719	15.083
H	47.927	18.370	18.092
H	48.667	21.636	15.670
H	50.267	19.304	16.760
N	41.331	19.340	16.597
C	40.068	19.276	15.888
C	39.186	18.182	16.363
O	39.542	16.988	16.216
C	40.393	19.110	14.378
C	39.215	19.354	13.418
C	38.658	20.799	13.397
N	39.647	21.825	13.771
C	40.699	22.245	13.046
N	40.937	21.781	11.830
N	41.511	23.162	13.545
H	41.754	18.564	17.117
H	39.526	20.232	16.034
H	41.227	19.783	14.088
H	40.793	18.092	14.199
H	39.557	19.090	12.394
H	38.390	18.647	13.654
H	38.223	21.024	12.397
H	37.806	20.847	14.112
H	39.565	22.243	14.721
H	40.345	21.060	11.393
H	41.854	22.005	11.386
H	41.493	23.395	14.554
H	42.385	23.416	13.040
N	37.835	18.492	16.676
C	36.814	17.471	16.867
C	35.756	17.604	15.796
O	35.095	18.672	15.704
C	36.266	17.647	18.282
C	35.431	16.519	18.758
H	37.548	19.493	16.738
H	37.261	16.457	16.792
H	37.135	17.703	18.961
H	35.679	18.586	18.361
N	35.005	16.532	20.025
O	35.236	15.514	18.043
H	35.217	17.324	20.602
H	34.430	15.797	20.378
N	35.581	16.616	14.873
C	34.581	16.688	13.801
C	33.316	15.933	14.173
O	32.420	15.781	13.304
C	35.222	16.156	12.515
H	36.143	15.741	14.974
H	34.281	17.743	13.611
H	35.493	15.084	12.614
H	34.533	16.286	11.658
H	36.147	16.731	12.298
N	33.138	15.375	15.487
C	31.953	14.618	15.930
C	31.730	13.391	15.083
O	30.881	13.407	14.156
C	30.723	15.540	16.047
C	31.019	16.728	16.927
H	33.730	15.763	16.250
H	32.149	14.220	16.944
H	30.410	15.883	15.040
H	29.869	14.978	16.487
C	30.949	18.003	16.579
N	31.418	16.652	18.210
C	31.570	17.932	18.597
N	31.297	18.756	17.640
H	31.590	15.797	18.782

H	30.651	18.396	15.616
H	31.870	18.238	19.599
N	32.661	12.304	15.163
C	32.690	11.188	14.183
C	33.674	10.084	14.555
O	34.473	10.257	15.510
C	33.008	11.705	12.768
O	34.233	12.384	12.748
H	33.296	12.299	15.995
H	31.685	10.734	14.166
H	33.056	10.860	12.051
H	32.202	12.370	12.394
H	34.103	13.209	13.288
N	33.730	8.917	13.837
C	34.684	7.829	14.122
C	34.755	6.836	12.968
O	33.708	6.229	12.625
C	34.287	7.082	15.416
H	32.994	8.741	13.125
H	35.684	8.279	14.243
H	33.288	7.397	15.779
H	34.233	5.990	15.215
H	35.023	7.234	16.241
N	35.928	6.544	12.332
C	36.055	5.421	11.383
C	36.413	4.100	12.055
O	37.327	4.056	12.896
C	37.025	5.748	10.243
C	37.089	4.627	9.198
O	36.618	6.946	9.637
H	36.761	7.137	12.499
H	35.090	5.303	10.885
H	38.023	5.922	10.679
H	37.526	3.706	9.621
H	36.068	4.402	8.829
H	37.723	4.940	8.344
H	36.118	6.730	8.808
N	35.822	2.996	11.571
C	36.395	1.688	11.772
C	36.767	1.097	10.416
O	35.998	1.178	9.468
C	35.460	0.716	12.505
C	35.046	1.301	13.830
O	34.306	0.461	11.679
H	34.940	3.176	11.055
H	37.232	1.776	12.313
H	35.944	-0.168	12.648
H	34.049	1.426	13.858
H	35.315	0.688	14.580
H	35.487	2.193	13.969
H	34.012	1.307	11.226
N	37.962	0.529	10.390
C	38.476	-0.258	9.302
C	38.453	-1.718	9.706
O	39.006	-2.077	10.733
C	39.898	0.170	8.926
C	40.066	1.552	8.366
C	40.303	1.819	7.046
C	40.191	2.818	9.049
C	40.433	3.799	8.063
C	40.032	3.261	10.368
N	40.498	3.158	6.870
C	40.577	5.158	8.366
C	40.202	4.592	10.678
C	40.512	5.529	9.664
H	38.527	0.713	11.275
H	37.861	-0.152	8.519
H	40.478	0.113	9.746
H	40.256	-0.472	8.238
H	40.329	1.133	6.321
H	40.666	3.606	5.979
H	39.791	2.604	11.094
H	40.826	5.869	7.594
H	40.116	4.882	11.632
H	40.705	6.557	9.935
N	37.946	-2.551	8.792
C	37.927	-3.999	8.925
C	38.548	-4.585	7.657
O	38.188	-4.212	6.539
C	36.495	-4.439	9.121
O	36.416	-5.835	9.273
H	37.552	-2.053	7.947
H	38.505	-4.253	9.692
H	36.128	-4.002	9.939
H	35.959	-4.168	8.324
H	35.552	-6.079	9.715
N	39.552	-5.453	7.820
C	40.261	-5.989	6.668
C	41.249	-7.090	7.021
O	41.120	-7.798	8.030
H	39.766	-5.696	8.798
H	39.586	-6.351	5.997
H	40.758	-5.234	6.199
N	42.214	-7.260	6.122
C	43.178	-8.320	6.300
C	44.521	-7.869	5.737
O	44.614	-7.132	4.754
C	42.623	-9.644	5.754
C	42.310	-9.702	4.276
C	41.746	-11.042	3.855
H	42.206	-6.601	5.334
H	43.299	-8.479	7.281
H	43.294	-10.377	5.964
H	41.773	-9.864	6.265
H	41.639	-8.993	4.059
H	43.150	-9.534	3.760
N	40.735	-11.006	3.000
O	42.220	-12.103	4.277
H	40.308	-11.869	2.657
H	40.384	-10.104	2.686
N	45.557	-8.288	6.462
C	46.941	-8.134	6.065
C	47.325	-9.311	5.156
O	47.067	-10.462	5.486
C	47.779	-8.063	7.346
C	49.250	-8.284	7.128
C	50.050	-7.236	6.699
C	49.841	-9.523	7.343
C	51.407	-7.415	6.489
C	51.203	-9.714	7.155
C	51.985	-8.651	6.726
O	53.341	-8.741	6.504
H	45.272	-8.751	7.369
H	47.034	-7.295	5.532
H	47.651	-7.159	7.750
H	47.444	-8.763	7.974
H	49.643	-6.335	6.537
H	49.275	-10.292	7.640
H	51.972	-6.650	6.167
H	51.616	-10.605	7.326
H	53.789	-7.934	6.884
N	47.941	-9.001	4.011
C	48.430	-9.982	3.060
C	49.932	-9.726	2.897
O	50.336	-8.685	2.362
C	47.669	-9.877	1.725
C	48.098	-10.972	0.769
C	46.152	-9.892	1.914
H	48.041	-7.961	3.859
H	48.318	-10.886	3.449
H	47.915	-9.007	1.305
H	48.690	-11.623	1.246
H	47.291	-11.452	0.422
H	48.599	-10.572	0.001
H	45.937	-10.296	2.802
H	45.809	-8.955	1.876
H	45.737	-10.435	1.186
N	50.746	-10.626	3.458
C	52.194	-10.448	3.500
C	52.849	-10.766	2.157
O	52.166	-10.905	1.148
H	50.259	-11.456	3.857
H	52.408	-9.494	3.755
H	52.586	-11.051	4.210
N	54.182	-10.889	2.164
C	54.929	-11.373	1.014
C	55.445	-10.230	0.144
O	55.742	-9.135	0.638
H	54.631	-10.608	3.064
H	55.714	-11.928	1.330
H	54.337	-11.973	0.454
N	55.582	-10.518	-1.159
C	55.875	-9.496	-2.151
C	54.755	-8.455	-2.110
O	53.583	-8.827	-2.196
C	56.009	-10.131	-3.523
H	55.460	-11.526	-1.388
H	56.733	-9.047	-1.916
H	55.257	-10.774	-3.671
H	55.980	-9.421	-4.227
H	56.879	-10.621	-3.583
N	55.119	-7.168	-1.941
C	54.151	-6.074	-1.961
C	53.085	-6.368	-0.898
O	52.007	-6.873	-1.206
C	53.530	-5.897	-3.358
C	54.541	-5.767	-4.499
C	54.621	-4.401	-5.159
O	53.579	-3.930	-5.635
O	55.726	-3.821	-5.218
H	56.140	-7.031	-1.797
H	54.603	-5.215	-1.748
H	52.977	-6.692	-3.541
H	52.992	-5.072	-3.343
H	55.442	-5.975	-4.133
H	54.294	-6.424	-5.203
N	53.471	-6.157	0.363
C	52.592	-6.360	1.501
C	51.505	-5.283	1.440
O	51.792	-4.122	1.120
C	53.394	-6.287	2.779
H	54.463	-5.833	0.455
H	52.157	-7.251	1.427
H	54.190	-6.892	2.716
H	53.707	-5.348	2.931
H	52.826	-6.572	3.553
N	50.276	-5.704	1.764
C	49.115	-4.837	1.696
C	48.247	-5.070	2.925
O	48.247	-6.154	3.509
C	48.319	-5.123	0.421
C	48.120	-3.768	-0.453
C	47.982	-4.029	-1.940
N	49.083	-3.461	-2.703
C	50.280	-4.021	-2.829
N	51.078	-4.119	-1.781
N	50.673	-4.478	-4.005
H	50.234	-6.702	2.066
H	49.417	-3.883	1.694
H	48.812	-5.734	-0.153
H	47.422	-5.423	0.645
H	47.299	-3.325	-0.110
H	48.918	-3.200	-0.283
H	47.957	-5.017	-2.081
H	47.122	-3.623	-2.247
H	48.927	-2.579	-3.170
H	50.798	-3.799	-0.880
H	51.996	-4.529	-1.890
H	50.058	-4.419	-4.796
H	51.580	-4.884	-4.112
N	47.519	-4.027	3.314
C	46.357	-4.213	4.168
C	45.146	-3.739	3.365
O	45.086	-2.582	2.966
C	46.521	-3.487	5.505
C	47.766	-3.956	6.275
C	45.246	-3.650	6.339
C	47.967	-3.203	7.554
H	47.842	-3.111	2.968
H	46.248	-5.189	4.290
H	46.630	-2.509	5.319
H	47.661	-4.925	6.471
H	48.557	-3.823	5.686
H	45.326	-3.107	7.171
H	44.463	-3.336	5.807

H	45.128	-4.611	6.575
H	47.173	-3.330	8.151
H	48.786	-3.540	8.021
H	48.078	-2.227	7.359
N	44.216	-4.654	3.075
C	43.034	-4.322	2.298
C	41.838	-4.199	3.230
O	41.632	-5.092	4.047
C	42.709	-5.410	1.277
C	43.765	-5.527	0.201
H	44.414	-5.603	3.449
H	43.185	-3.419	1.894
H	42.643	-6.281	1.756
H	41.838	-5.188	0.847
N	44.128	-6.755	-0.100
O	44.269	-4.527	-0.317
H	43.721	-7.546	0.355
H	44.834	-6.912	-0.806
N	41.157	-3.047	3.190
C	40.134	-2.774	4.193
C	38.860	-2.253	3.545
O	38.877	-1.667	2.451
C	40.525	-1.723	5.256
C	41.817	-2.034	5.968
O	40.620	-0.438	4.658
H	41.410	-2.411	2.429
H	39.918	-3.634	4.672
H	39.793	-1.696	5.969
H	42.151	-1.219	6.452
H	41.676	-2.771	6.636
H	42.516	-2.326	5.307
H	41.100	-0.492	3.773
N	37.768	-2.427	4.317
C	36.548	-1.676	4.158
C	36.349	-0.869	5.425
O	36.854	-1.258	6.480
C	35.424	-2.646	3.851
C	35.630	-3.235	2.441
C	34.454	-3.922	1.794
H	37.899	-3.171	5.051
H	36.666	-1.037	3.404
H	35.443	-3.390	4.491
H	34.565	-2.170	3.853
H	35.939	-2.477	1.833
H	36.406	-3.895	2.494
N	34.511	-5.243	1.803
O	33.546	-3.292	1.240
H	33.741	-5.799	1.393
H	35.311	-5.709	2.211
N	35.674	0.275	5.302
C	35.517	1.127	6.476
C	34.109	1.694	6.552
O	33.381	1.808	5.553
C	36.603	2.222	6.531
C	36.709	3.111	5.328
C	37.703	3.065	4.396
C	35.876	4.222	4.970
C	36.404	4.782	3.786
C	34.741	4.786	5.551
N	37.505	4.045	3.444
C	35.817	5.873	3.159
C	34.156	5.834	4.921
C	34.695	6.381	3.723
H	35.305	0.483	4.377
H	35.660	0.554	7.291
H	36.419	2.811	7.321
H	37.494	1.779	6.653
H	38.465	2.421	4.396
H	38.075	4.190	2.637
H	34.332	4.384	6.463
H	36.227	6.277	2.244
H	33.249	6.256	5.327
H	34.188	7.210	3.245
N	33.733	2.030	7.791
C	32.483	2.650	8.141
C	32.847	3.894	8.955
O	33.525	3.792	9.997
C	31.630	1.699	9.011
C	31.037	0.489	8.292
C	30.341	-0.435	9.303
C	30.101	0.931	7.218
H	34.473	1.786	8.519
H	32.017	2.945	7.320
H	32.213	1.360	9.742
H	30.877	2.229	9.382
H	31.767	-0.016	7.840
H	29.611	0.070	9.759
H	29.959	-1.220	8.820
H	31.009	-0.745	9.977
H	30.586	1.499	6.544
H	29.711	0.134	6.745
H	29.349	1.471	7.610
N	32.415	5.130	8.534
C	32.687	6.432	9.180
C	31.399	7.006	9.728
O	30.684	7.753	9.012
C	33.322	7.370	8.129
C	33.924	8.701	8.641
C	34.610	9.387	7.469
C	32.905	9.681	9.214
H	31.844	5.153	7.664
H	33.394	6.318	10.020
H	34.132	6.799	7.630
H	32.577	7.598	7.346
H	34.672	8.498	9.431
H	33.887	9.590	6.651
H	35.046	10.340	7.804
H	35.417	8.739	7.076
H	32.497	9.308	10.174
H	33.384	10.657	9.435
H	32.083	9.844	8.487
N	31.012	6.756	11.004
C	29.744	7.279	11.543
C	29.882	8.703	11.984
O	30.738	9.004	12.850
C	29.244	6.415	12.688
C	27.929	6.915	13.241
O	29.119	5.070	12.217
H	31.637	6.193	11.615
H	28.969	7.222	10.765
H	29.921	6.426	13.450
H	28.090	7.479	14.056
H	27.344	7.497	12.498
H	27.344	6.139	13.494
H	29.995	4.584	12.308
N	29.030	9.657	11.518
C	29.067	11.064	11.948
C	27.990	11.285	12.980
O	26.799	11.032	12.673
C	28.879	11.995	10.722
C	30.002	11.746	9.682
C	28.871	13.469	11.152
C	29.639	12.237	8.283
H	28.254	9.359	10.889
H	30.048	11.305	12.404
H	27.895	11.763	10.253
H	30.950	12.220	10.013
H	30.194	10.658	9.587
H	28.756	14.143	10.282
H	28.018	13.669	11.836
H	29.812	13.724	11.685
H	29.467	13.330	8.275
H	30.471	12.007	7.584
H	28.727	11.714	7.926
N	28.294	11.694	14.246
C	27.283	11.962	15.279
C	26.204	12.895	14.791
O	26.496	14.091	14.515
H	29.291	11.742	14.525
H	26.843	11.004	15.624
H	27.764	12.437	16.159
N	24.962	12.409	14.495
C	23.806	13.249	14.131
C	22.622	12.980	15.025
O	22.382	11.805	15.402
C	23.364	13.027	12.670
C	24.436	13.442	11.674
O	23.022	11.689	12.428
H	24.830	11.379	14.527
H	24.070	14.322	14.251
H	22.485	13.673	12.465
H	24.715	14.505	11.829
H	25.337	12.813	11.796
H	24.054	13.306	10.641
H	22.188	11.511	12.943
N	21.571	13.942	15.031
C	20.216	13.730	15.607
C	19.484	12.448	15.151
O	19.857	11.848	14.139
C	19.342	14.983	15.429
C	19.912	16.312	15.897
O	19.004	15.092	14.069
H	21.974	14.901	15.066
H	20.272	13.636	16.669
H	18.466	14.910	15.935
H	19.142	17.109	15.810
H	20.135	16.184	16.940
H	20.810	16.659	15.351
H	19.800	15.486	13.625
N	18.419	12.046	15.851
C	17.622	10.895	15.477
C	17.045	11.104	14.080
O	17.028	10.171	13.263
C	16.554	10.665	16.556
C	17.160	10.194	17.875
C	16.861	11.073	19.080
O	17.757	11.843	19.480
O	15.750	10.977	19.625
H	18.216	12.638	16.691
H	18.188	10.068	15.482
H	16.109	11.517	16.721
H	15.954	9.965	16.238
H	16.802	9.289	18.072
H	18.146	10.164	17.768
N	16.621	12.344	13.798
C	15.987	12.653	12.525
C	16.975	12.446	11.374
O	16.565	12.153	10.252
C	15.469	14.066	12.541
H	16.783	13.037	14.548
H	15.207	12.034	12.388
H	16.166	14.682	12.915
H	15.242	14.358	11.609
H	14.645	14.123	13.110
N	18.270	12.621	11.653
C	19.317	12.678	10.615
C	20.195	11.459	10.724
O	21.140	11.305	9.907
C	20.117	13.989	10.744
C	19.345	15.169	10.253
H	18.482	12.769	12.657
H	18.872	12.631	9.594
H	20.398	14.161	11.798
H	21.053	13.918	10.150
N	18.324	15.670	10.955
O	19.661	15.737	9.181
H	18.071	15.283	11.883
H	17.845	16.522	10.606
N	19.685	10.334	11.434
C	20.446	9.121	11.658
C	20.575	8.313	10.358
O	21.528	7.506	10.194
C	19.854	8.303	12.768
H	18.777	10.541	11.913
H	21.480	9.409	11.954
H	19.486	7.443	12.405
H	20.556	8.091	13.453
H	19.111	8.811	13.212
N	19.633	8.465	9.420
C	19.701	7.704	8.173
C	20.872	8.150	7.310
O	21.499	7.307	6.622
C	18.377	7.810	7.411
C	18.121	9.186	6.885
C	17.558	10.223	7.570

C	18.531	9.714	5.608
C	18.135	11.070	5.582
C	19.119	9.168	4.466
N	17.560	11.350	6.791
C	18.336	11.889	4.473
C	19.336	9.980	3.375
C	18.932	11.314	3.373
H	18.890	9.137	9.647
H	19.826	6.731	8.412
H	18.403	7.195	6.636
H	17.635	7.583	8.025
H	17.198	10.170	8.499
H	17.196	12.245	7.063
H	19.381	8.203	4.437
H	18.055	12.848	4.487
H	19.792	9.601	2.570
H	19.081	11.865	2.549
N	21.274	9.461	7.315
C	22.457	9.990	6.601
C	23.595	10.296	7.555
O	24.364	11.269	7.314
C	22.059	11.219	5.758
C	21.447	12.350	6.601
C	21.295	13.654	5.814
C	20.626	14.698	6.712
N	20.382	15.963	6.010
H	20.727	10.098	7.926
H	22.854	9.216	5.909
H	22.953	11.592	5.212
H	21.317	10.891	5.007
H	20.441	12.041	6.951
H	22.078	12.545	7.495
H	22.298	14.017	5.508
H	20.676	13.476	4.908
H	19.659	14.288	7.095
H	21.290	14.876	7.594
H	21.282	16.417	5.735
H	19.798	15.814	5.155
H	19.872	16.613	6.657
N	23.875	9.439	8.578
C	25.067	9.564	9.439
C	26.211	8.764	8.879
O	27.322	9.325	8.678
C	24.757	9.107	10.871
O	24.526	7.723	10.913
H	23.276	8.591	8.680
H	25.384	10.629	9.505
H	25.623	9.323	11.522
H	23.887	9.683	11.255
H	23.644	7.577	11.347
N	26.055	7.443	8.570
C	27.189	6.553	8.268
C	27.588	6.655	6.834
O	26.721	6.533	5.935
C	26.862	5.087	8.616
C	28.099	4.178	8.589
O	26.267	5.019	9.888
H	25.101	7.022	8.646
H	28.051	6.862	8.877
H	26.122	4.702	7.882
H	28.927	4.710	8.786
H	27.981	3.482	9.302
H	28.209	3.721	7.702
H	26.953	5.296	10.556
N	28.961	6.808	6.483
C	29.486	6.504	5.152
C	30.040	5.089	5.102
O	30.216	4.419	6.154
C	30.557	7.555	4.775
C	29.985	8.980	4.594
C	31.078	10.027	4.822
C	29.406	9.171	3.192
H	29.642	6.796	7.261
H	28.675	6.543	4.407
H	31.309	7.567	5.582
H	31.099	7.260	3.851
H	29.183	9.171	5.338
H	31.938	9.843	4.144
H	30.678	11.045	4.644
H	31.428	9.972	5.874
H	28.600	8.433	3.008
H	28.969	10.184	3.088
H	30.189	9.030	2.418
N	30.443	4.592	3.908
C	31.175	3.331	3.689
C	32.161	3.536	2.539
O	31.857	4.282	1.605
C	30.227	2.163	3.396
C	29.413	2.376	2.138
C	30.927	0.798	3.412
H	30.345	5.230	3.100
H	31.705	3.127	4.512
H	29.577	2.099	4.159
H	28.445	2.201	2.328
H	29.521	3.320	1.821
H	29.727	1.750	1.422
H	30.693	0.304	2.577
H	31.914	0.940	3.459
H	30.618	0.285	4.210
N	33.342	2.952	2.701
C	34.363	2.981	1.667
C	35.353	1.846	1.819
O	35.129	0.903	2.561
H	33.470	2.479	3.620
H	33.922	2.922	0.758
H	34.856	3.864	1.707
N	36.431	1.933	1.030
C	37.445	0.902	0.964
C	38.799	1.603	0.828
O	38.904	2.568	0.063
C	37.116	-0.119	-0.130
C	36.804	0.523	-1.407
H	36.470	2.816	0.467
H	37.446	0.392	1.816
H	37.906	-0.726	-0.246
H	36.328	-0.662	0.170
C	35.736	1.139	-1.946
N	37.836	0.662	-2.313
C	37.371	1.253	-3.415
N	36.120	1.599	-3.198
H	38.776	0.369	-2.171
H	34.837	1.252	-1.537
H	37.887	1.403	-4.250
N	39.744	1.218	1.687
C	41.107	1.719	1.686
C	42.066	0.538	1.503
O	41.916	-0.502	2.152
C	41.454	2.480	2.978
C	40.885	3.880	3.134
O	40.277	4.366	2.163
O	41.027	4.461	4.262
H	39.404	0.499	2.385
H	41.214	2.304	0.884
H	41.135	1.917	3.756
H	42.462	2.536	3.035
N	43.065	0.716	0.613
C	44.175	-0.221	0.492
C	45.477	0.447	0.931
O	45.832	1.510	0.446
C	44.418	-0.763	-0.932
C	45.453	-1.867	-0.944
O	43.202	-1.285	-1.449
H	42.966	1.573	0.032
H	44.003	-1.003	1.097
H	44.718	0.010	-1.526
H	45.001	-2.763	-0.910
H	46.004	-1.813	-1.781
H	46.060	-1.779	-0.149
H	42.785	-1.929	-0.796
N	46.138	-0.196	1.905
C	47.336	0.338	2.538
C	48.570	-0.395	2.038
O	48.593	-1.635	1.984
C	47.248	0.217	4.049
C	46.172	1.083	4.653
C	44.870	0.616	4.778
C	46.442	2.370	5.050
C	43.883	1.444	5.287
C	45.458	3.182	5.614
C	44.183	2.705	5.722
H	45.713	-1.116	2.167
H	47.424	1.294	2.241
H	47.040	-0.721	4.272
H	48.112	0.500	4.432
H	44.644	-0.310	4.505
H	47.370	2.745	4.936
H	42.928	1.097	5.330
H	45.704	4.109	5.931
H	43.459	3.280	6.123
N	49.604	0.380	1.705
C	50.904	-0.242	1.452
C	51.984	0.593	2.125
O	51.719	1.689	2.596
C	51.247	-0.437	-0.032
C	50.143	-1.072	-0.851
O	51.611	0.806	-0.622
H	49.417	1.379	1.647
H	50.905	-1.152	1.861
H	52.047	-1.073	-0.104
H	49.251	-0.886	-0.429
H	50.146	-0.697	-1.782
H	50.279	-2.065	-0.900
H	50.838	1.451	-0.596
N	53.214	0.061	2.160
C	54.304	0.746	2.827
C	54.929	1.822	1.926
O	55.660	2.669	2.415
C	55.260	-0.313	3.387
C	54.663	-1.091	4.549
C	55.662	-1.626	5.537
C	54.991	-2.212	6.757
N	55.813	-3.285	7.369
H	53.304	-0.851	1.682
H	53.954	1.197	3.660
H	55.477	-0.952	2.659
H	56.081	0.147	3.704
H	54.007	-0.486	5.038
H	54.122	-1.866	4.173
H	56.205	-2.342	5.099
H	56.263	-0.883	5.831
H	54.844	-1.484	7.433
H	54.100	-2.591	6.492
H	56.378	-2.898	8.099
H	55.213	-3.989	7.750
H	56.403	-3.692	6.672
N	54.572	1.870	0.647
C	55.040	2.919	-0.260
C	54.037	4.089	-0.295
O	52.904	3.889	-0.703
C	55.180	2.316	-1.674
C	55.711	3.329	-2.686
C	56.014	1.042	-1.706
H	53.934	1.102	0.345
H	55.909	3.275	0.091
H	54.265	2.050	-1.982
H	55.441	3.051	-3.607
H	55.330	4.230	-2.484
H	56.708	3.366	-2.627
H	55.833	0.551	-2.558
H	56.983	1.281	-1.654
H	55.767	0.467	-0.927
N	54.449	5.307	0.077
C	53.608	6.496	-0.043
C	53.416	6.846	-1.513
O	54.403	6.919	-2.244
C	54.261	7.686	0.667
C	53.444	8.971	0.724
C	54.169	10.084	1.481
C	53.524	11.447	1.327
N	52.051	11.377	1.491
H	55.420	5.331	0.459
H	52.712	6.303	0.363
H	54.471	7.407	1.612
H	55.130	7.888	0.198
H	53.263	9.285	-0.210

H	52.573	8.786	1.183
H	54.186	9.845	2.456
H	55.113	10.136	1.143
H	53.904	12.068	2.018
H	53.738	11.808	0.415
H	51.714	12.246	1.855
H	51.624	11.200	0.604
H	51.820	10.638	2.125
N	52.168	7.090	-1.990
C	51.890	7.182	-3.428
C	52.088	8.590	-4.018
O	52.571	9.446	-3.291
C	50.417	6.749	-3.480
C	49.833	7.344	-2.203
C	50.955	7.307	-1.180
O	51.843	9.027	-5.118
H	52.450	6.504	-3.944
H	49.961	7.128	-4.277
H	50.334	5.760	-3.469
H	49.532	8.284	-2.371
H	49.052	6.797	-1.898
H	51.015	8.171	-0.679
H	50.825	6.556	-0.529
S	38.208	7.982	7.286
O	42.350	7.884	5.288
N	40.384	9.303	5.294
N	40.127	7.009	4.747
C	37.110	13.197	9.657
C	38.129	12.593	8.708
C	37.542	11.401	7.948
C	38.613	10.766	7.074
C	38.099	9.547	6.260
C	38.983	9.135	5.069
C	37.924	6.982	5.934
C	38.772	7.535	4.699
C	41.085	8.067	5.124
H	40.488	9.557	6.256
H	40.144	6.334	5.485
H	37.561	14.050	10.179
H	36.849	12.464	10.423
H	39.010	12.253	9.302
H	38.486	13.361	7.983
H	37.167	10.644	8.673
H	36.718	11.755	7.294
H	39.444	10.485	7.760
H	38.965	11.533	6.351
H	37.128	9.879	5.958
H	38.671	9.785	4.278
H	38.316	6.016	6.173
H	36.891	7.092	5.677
H	38.295	7.302	3.770
C	28.887	15.917	4.304
N	31.399	15.051	6.436
O	35.898	14.375	7.923
C	29.576	15.435	3.060
N	34.684	13.258	9.520
C	28.313	15.098	5.365
O	28.504	17.962	8.680
C	28.288	13.600	5.396
O	29.416	16.865	10.442
C	27.650	15.977	6.292
N	30.197	16.468	8.309
C	26.863	15.527	7.485
C	27.790	17.341	5.808
C	27.109	18.574	6.324
C	28.514	17.279	4.565
C	28.828	18.451	3.692
C	31.203	15.602	8.759
C	31.867	14.884	7.716
C	31.654	15.363	10.058
C	32.948	13.998	7.964
C	32.773	14.551	10.295
C	33.463	13.922	9.282
C	33.421	13.198	6.898
C	31.863	14.290	5.448
C	32.855	13.319	5.653
C	35.873	13.691	8.932
C	29.307	17.160	9.140
C	28.492	17.420	11.479
C	28.569	18.947	11.514
C	27.067	16.922	11.235
C	29.052	16.842	12.777
Ir	29.872	16.452	6.255
H	29.691	14.347	3.062
H	30.560	15.896	2.969
H	28.995	15.697	2.172
H	34.781	12.837	10.435
H	27.785	13.228	6.293
H	29.289	13.160	5.372
H	27.742	13.200	4.538
H	26.765	16.323	8.222
H	27.334	14.674	7.984
H	25.859	15.202	7.188
H	26.581	18.379	7.256
H	27.829	19.367	6.542
H	26.396	18.953	5.586
H	28.920	18.154	2.646
H	28.052	19.216	3.776
H	29.777	18.905	4.000
H	31.169	15.821	10.904
H	33.113	14.434	11.316
H	34.201	12.475	7.092
H	31.447	14.458	4.469
H	33.162	12.686	4.829
H	27.955	19.304	12.348
H	29.596	19.272	11.695
H	28.199	19.393	10.593
H	26.455	17.174	12.108
H	27.043	15.834	11.132
H	26.619	17.388	10.358
H	28.458	17.200	13.623
H	30.090	17.150	12.929
H	29.008	15.750	12.794
N	31.253	17.810	5.654
C	32.405	17.587	4.939
O	32.479	16.721	4.058
C	33.566	18.538	5.205
H	34.315	18.421	4.422
H	34.042	18.261	6.154
C	33.049	19.977	5.303
H	32.614	20.290	4.350
H	33.890	20.653	5.502
C	32.019	20.091	6.410
H	31.124	20.661	6.133
H	31.442	18.870	6.308
C	32.456	20.385	7.828
C	33.202	21.759	7.857
C	31.255	20.435	8.794
H	33.174	19.625	8.164
C	33.612	22.155	9.283
H	32.541	22.532	7.440
H	34.092	21.723	7.222
C	31.640	20.864	10.218
H	30.528	21.157	8.395
H	30.745	19.468	8.813
C	32.412	22.190	10.235
H	34.100	23.137	9.251
H	34.367	21.450	9.652
H	30.731	20.962	10.822
H	32.238	20.078	10.693
H	32.739	22.427	11.253
H	31.737	23.003	9.931
N	5.895	-16.409	-4.446
C	6.072	-15.132	-3.679
C	5.712	-13.892	-4.493
O	5.793	-12.789	-3.964
C	7.514	-14.873	-3.249
C	8.108	-15.913	-2.322
O	7.335	-16.676	-1.661
O	9.339	-15.996	-2.319
H	5.569	-16.397	-5.399
H	5.431	-15.182	-2.900
H	8.078	-14.825	-4.079
H	7.542	-13.981	-2.789
N	5.345	-14.072	-5.767
C	4.885	-12.989	-6.622
C	3.762	-12.202	-5.939
O	3.828	-10.976	-5.871
C	4.443	-13.595	-7.957
C	4.312	-12.585	-9.060
C	3.729	-13.160	-10.341
O	3.587	-12.393	-11.295
O	3.389	-14.366	-10.364
H	5.420	-15.066	-6.092
H	5.647	-12.366	-6.827
H	5.126	-14.252	-8.222
H	3.563	-14.011	-7.815
H	3.705	-11.865	-8.762
H	5.208	-12.235	-9.281
N	2.751	-12.917	-5.408
C	1.622	-12.318	-4.708
C	2.053	-11.659	-3.399
O	1.514	-10.616	-2.996
C	0.602	-13.396	-4.424
H	2.855	-13.952	-5.547
H	1.209	-11.611	-5.300
H	-0.319	-13.049	-4.609
H	0.777	-14.191	-5.008
H	0.661	-13.675	-3.464
N	2.977	-12.322	-2.694
C	3.450	-11.867	-1.404
C	4.236	-10.561	-1.519
O	4.150	-9.691	-0.644
H	3.321	-13.193	-3.161
H	2.666	-11.723	-0.783
H	4.044	-12.571	-0.990
N	5.056	-10.439	-2.573
C	5.892	-9.238	-2.719
C	5.104	-8.074	-3.344
O	5.341	-6.896	-3.037
C	7.151	-9.571	-3.552
C	8.070	-10.525	-2.787
C	7.896	-8.319	-4.007
C	9.064	-11.290	-3.670
H	5.048	-11.219	-3.234
H	6.134	-8.950	-1.788
H	6.853	-10.028	-4.384
H	8.587	-10.001	-2.112
H	7.503	-11.193	-2.305
H	8.649	-8.581	-4.607
H	7.268	-7.721	-4.501
H	8.256	-7.839	-3.209
H	9.645	-10.634	-4.143
H	9.617	-11.882	-3.092
H	8.554	-11.834	-4.330
N	4.239	-8.375	-4.317
C	3.482	-7.337	-4.994
C	2.643	-6.541	-3.987
O	1.915	-7.103	-3.189
C	2.601	-7.942	-6.094
C	1.656	-6.931	-6.705
O	3.485	-8.438	-7.104
H	4.163	-9.383	-4.534
H	4.124	-6.701	-5.435
H	2.085	-8.722	-5.711
H	0.711	-7.267	-6.653
H	1.718	-6.059	-6.211
H	1.893	-6.776	-7.668
H	3.788	-9.370	-6.878
N	2.715	-5.217	-4.061
C	1.946	-4.372	-3.165
C	2.750	-3.184	-2.651
O	3.736	-2.770	-3.273
H	3.351	-4.853	-4.794
H	1.122	-4.030	-3.647
H	1.625	-4.921	-2.375
N	2.290	-2.648	-1.518
C	2.805	-1.419	-0.958
C	3.485	-1.770	0.354
O	2.930	-2.460	1.218
C	1.698	-0.367	-0.759
C	2.233	0.920	-0.174
O	1.064	-0.078	-2.008
H	1.518	-3.204	-1.072
H	3.488	-1.042	-1.575

H	0.993	-0.748	-0.124
H	3.228	0.857	-0.054
H	2.029	1.689	-0.787
H	1.809	1.096	0.719
H	1.735	-0.109	-2.757
N	4.712	-1.255	0.492
C	5.539	-1.486	1.655
C	5.963	-0.154	2.257
O	6.234	0.801	1.540
C	6.745	-2.294	1.205
C	6.404	-3.661	0.703
C	6.160	-4.020	-0.586
C	6.427	-4.866	1.462
C	6.126	-5.913	0.581
C	6.617	-5.160	2.810
N	6.013	-5.374	-0.671
C	6.034	-7.234	1.005
C	6.511	-6.459	3.235
C	6.230	-7.488	2.338
H	5.016	-0.668	-0.324
H	4.986	-1.964	2.342
H	7.201	-1.813	0.463
H	7.366	-2.409	1.974
H	6.097	-3.387	-1.357
H	5.849	-5.894	-1.511
H	6.831	-4.429	3.465
H	5.830	-7.966	0.347
H	6.637	-6.670	4.203
H	6.172	-8.427	2.679
N	6.073	-0.141	3.577
C	6.435	1.038	4.327
C	7.645	0.739	5.195
O	7.725	-0.299	5.836
C	5.256	1.462	5.220
C	3.967	1.637	4.471
C	3.742	2.774	3.717
C	2.956	0.685	4.527
C	2.584	2.936	2.975
C	1.773	0.844	3.812
C	1.574	1.990	3.068
O	0.444	2.180	2.328
H	5.872	-1.069	4.030
H	6.686	1.766	3.682
H	5.130	0.759	5.914
H	5.494	2.328	5.650
H	4.435	3.505	3.705
H	3.080	-0.131	5.091
H	2.475	3.729	2.374
H	1.073	0.131	3.839
H	0.375	1.463	1.634
N	8.593	1.685	5.246
C	9.743	1.451	6.089
C	9.536	2.222	7.380
O	8.510	2.868	7.577
C	11.069	1.739	5.383
C	11.326	3.206	5.132
H	8.430	2.510	4.679
H	9.752	0.483	6.325
H	11.811	1.376	5.951
H	11.065	1.259	4.503
N	12.400	3.498	4.403
O	10.568	4.054	5.580
H	12.981	2.764	4.050
H	12.627	4.452	4.207
N	10.515	2.087	8.263
C	10.509	2.715	9.568
C	10.484	4.243	9.423
O	9.990	4.896	10.311
C	11.680	2.143	10.387
C	13.097	2.573	9.960
C	13.731	1.992	8.698
H	11.307	1.476	7.929
H	9.669	2.446	10.047
H	11.559	2.413	11.353
H	11.642	1.134	10.341
H	13.096	3.600	9.868
H	13.731	2.381	10.750
N	14.809	2.641	8.268
O	13.305	0.987	8.111
H	15.305	2.308	7.433
H	15.130	3.458	8.768
N	10.976	4.814	8.309
C	10.989	6.267	8.094
C	9.650	6.808	7.568
O	9.513	8.021	7.418
C	12.077	6.612	7.067
C	13.519	6.316	7.478
C	14.461	6.541	6.300
C	13.917	7.166	8.671
H	11.348	4.133	7.611
H	11.185	6.719	8.979
H	11.889	6.088	6.239
H	12.014	7.588	6.875
H	13.585	5.366	7.777
H	14.396	7.492	5.999
H	15.400	6.344	6.582
H	14.204	5.934	5.548
H	13.312	6.966	9.444
H	14.863	6.962	8.929
H	13.841	8.136	8.434
N	8.733	5.912	7.158
C	7.458	6.292	6.561
C	7.533	6.424	5.038
O	6.579	6.832	4.387
H	9.022	4.917	7.308
H	6.756	5.600	6.797
H	7.152	7.175	6.953
N	8.663	6.043	4.442
C	8.739	6.004	2.992
C	7.838	4.876	2.478
O	7.673	3.888	3.179
C	10.185	5.831	2.522
O	11.026	6.852	3.044
H	9.433	5.793	5.073
H	8.382	6.864	2.632
H	10.521	4.946	2.833
H	10.207	5.874	1.527
H	11.108	6.739	4.033
N	7.393	5.011	1.234
C	6.441	4.117	0.586
C	7.029	3.535	-0.698
O	7.363	4.270	-1.617
C	5.134	4.851	0.243
C	4.073	3.922	-0.307
O	4.660	5.475	1.437
H	7.802	5.853	0.733
H	6.238	3.356	1.212
H	5.334	5.574	-0.439
H	3.164	4.218	-0.001
H	4.096	3.929	-1.311
H	4.235	2.985	0.016
H	5.432	5.735	2.029
N	7.074	2.203	-0.763
C	7.576	1.411	-1.881
C	6.389	0.659	-2.514
O	5.805	-0.204	-1.849
C	8.669	0.526	-1.264
C	9.169	-0.674	-2.008
C	9.534	-0.593	-3.335
C	9.431	-1.853	-1.323
C	10.093	-1.686	-3.983
C	9.894	-2.968	-1.989
C	10.259	-2.879	-3.312
H	6.690	1.738	0.121
H	7.906	2.047	-2.571
H	9.467	1.126	-1.078
H	8.320	0.207	-0.365
H	9.400	0.255	-3.841
H	9.281	-1.890	-0.333
H	10.374	-1.596	-4.949
H	9.962	-3.855	-1.495
H	10.643	-3.671	-3.788
N	6.030	1.002	-3.760
C	4.984	0.317	-4.505
C	5.611	-0.545	-5.606
O	6.253	-0.070	-6.529
C	3.953	1.320	-5.069
C	3.348	2.175	-3.947
C	2.873	0.605	-5.877
C	2.495	3.327	-4.434
H	6.574	1.810	-4.147
H	4.546	-0.316	-3.866
H	4.430	1.928	-5.696
H	2.792	1.581	-3.365
H	4.100	2.542	-3.397
H	2.265	1.281	-6.289
H	3.302	0.061	-6.595
H	2.349	0.008	-5.274
H	1.734	2.970	-4.974
H	2.140	3.831	-3.648
H	3.051	3.935	-4.999
N	5.422	-1.852	-5.513
C	6.190	-2.741	-6.363
C	5.282	-3.793	-6.971
O	4.337	-4.251	-6.327
C	7.357	-3.388	-5.599
C	6.866	-4.374	-4.555
C	8.325	-4.045	-6.570
H	4.726	-2.158	-4.827
H	6.584	-2.200	-7.112
H	7.840	-2.661	-5.111
H	7.411	-5.212	-4.601
H	6.955	-3.971	-3.644
H	5.905	-4.595	-4.727
H	8.174	-3.677	-7.487
H	9.263	-3.854	-6.282
H	8.169	-5.033	-6.575
N	5.603	-4.155	-8.216
C	5.034	-5.324	-8.870
C	6.139	-6.343	-9.097
O	7.122	-6.054	-9.758
C	4.346	-4.982	-10.207
C	3.674	-6.179	-10.856
O	3.369	-3.970	-9.956
H	6.299	-3.523	-8.677
H	4.349	-5.729	-8.265
H	5.040	-4.607	-10.845
H	2.687	-6.154	-10.680
H	3.830	-6.163	-11.847
H	4.052	-7.029	-10.480
H	3.812	-3.127	-9.630
N	5.947	-7.550	-8.563
C	6.844	-8.647	-8.864
C	6.336	-9.394	-10.104
O	5.237	-9.948	-10.090
C	6.913	-9.526	-7.653
H	5.127	-7.624	-7.937
H	7.755	-8.285	-9.067
H	6.130	-9.347	-7.050
H	6.897	-10.492	-7.925
H	7.760	-9.348	-7.144
N	7.122	-9.389	-11.180
C	6.790	-10.153	-12.378
C	7.109	-11.634	-12.198
O	7.879	-12.005	-11.312
H	7.976	-8.804	-11.092
H	5.809	-10.048	-12.582
H	7.313	-9.794	-13.161
N	6.541	-12.484	-13.065
C	6.700	-13.931	-12.926
C	8.140	-14.391	-13.240
O	8.554	-15.466	-12.836
C	5.670	-14.621	-13.802
H	5.996	-12.035	-13.819
H	6.488	-14.194	-11.974
H	4.778	-14.605	-13.349
H	5.603	-14.146	-14.680
H	5.944	-15.570	-13.960
N	8.880	-13.560	-13.972
C	10.205	-13.809	-14.519
C	11.337	-13.303	-13.608
O	12.492	-13.347	-13.998
C	10.327	-12.967	-15.796
C	10.283	-11.445	-15.555
O	9.305	-10.938	-14.899

O	11.249	-10.749	-16.017
H	8.380	-12.619	-14.138
H	10.320	-14.814	-14.591
H	11.196	-13.186	-16.231
H	9.573	-13.207	-16.401
N	10.993	-12.672	-12.489
C	11.965	-12.159	-11.541
C	12.118	-10.635	-11.563
O	12.985	-10.138	-10.846
H	9.957	-12.578	-12.353
H	11.704	-12.447	-10.598
H	12.875	-12.581	-11.726
N	11.282	-9.908	-12.343
C	11.329	-8.443	-12.432
C	10.634	-7.751	-11.255
O	9.596	-8.211	-10.768
C	10.716	-7.988	-13.743
H	10.598	-10.490	-12.878
H	12.300	-8.153	-12.446
H	10.072	-7.242	-13.572
H	11.437	-7.668	-14.359
H	10.231	-8.752	-14.171
N	11.247	-6.660	-10.768
C	10.600	-5.723	-9.852
C	10.517	-4.383	-10.564
O	11.513	-3.908	-11.124
C	11.369	-5.579	-8.522
C	11.600	-6.820	-7.667
C	12.477	-6.452	-6.472
C	10.301	-7.443	-7.153
H	12.231	-6.542	-11.104
H	9.679	-6.043	-9.666
H	12.273	-5.192	-8.742
H	10.867	-4.913	-7.959
H	12.061	-7.513	-8.211
H	12.019	-5.750	-5.928
H	12.630	-7.264	-5.910
H	13.354	-6.102	-6.800
H	9.732	-7.710	-7.930
H	10.516	-8.249	-6.602
H	9.812	-6.774	-6.594
N	9.303	-3.802	-10.547
C	9.004	-2.518	-11.161
C	8.018	-1.798	-10.243
O	7.236	-2.444	-9.565
C	8.421	-2.708	-12.575
C	9.217	-3.663	-13.437
O	7.102	-3.224	-12.415
H	8.578	-4.375	-10.039
H	9.837	-1.981	-11.207
H	8.362	-1.805	-13.026
H	9.671	-3.161	-14.178
H	9.915	-4.126	-12.884
H	8.609	-4.354	-13.838
H	7.095	-3.974	-11.743
N	8.123	-0.478	-10.142
C	7.209	0.279	-9.324
C	7.759	1.658	-9.013
O	8.454	2.247	-9.832
H	8.898	-0.060	-10.689
H	6.323	0.376	-9.801
H	7.038	-0.213	-8.457
N	7.385	2.176	-7.845
C	7.714	3.537	-7.495
C	8.067	3.582	-6.021
O	7.609	2.774	-5.215
C	6.565	4.525	-7.796
C	6.184	4.545	-9.256
O	5.429	4.146	-7.013
H	6.854	1.529	-7.234
H	8.515	3.824	-8.020
H	6.854	5.456	-7.513
H	5.292	4.992	-9.375
H	6.872	5.050	-9.787
H	6.126	3.607	-9.612
H	5.721	3.645	-6.191
N	8.844	4.597	-5.693
C	9.337	4.808	-4.353
C	9.052	6.259	-4.024
O	9.335	7.136	-4.837
C	10.853	4.583	-4.250
C	11.338	4.316	-2.843
C	11.632	5.352	-1.975
C	11.470	3.021	-2.367
C	12.070	5.108	-0.685
C	11.878	2.759	-1.065
C	12.186	3.803	-0.231
O	12.573	3.516	1.049
H	9.072	5.239	-6.494
H	8.820	4.239	-3.727
H	11.089	3.799	-4.818
H	11.311	5.400	-4.589
H	11.527	6.303	-2.284
H	11.265	2.250	-2.976
H	12.302	5.872	-0.078
H	11.945	1.813	-0.744
H	12.216	4.215	1.668
N	8.480	6.481	-2.852
C	8.347	7.834	-2.349
C	9.075	7.906	-1.021
O	8.685	7.194	-0.102
C	6.869	8.168	-2.188
C	6.641	9.508	-1.516
C	5.191	9.781	-1.149
O	4.938	10.128	0.041
O	4.323	9.637	-2.047
H	8.152	5.642	-2.351
H	8.753	8.479	-2.990
H	6.471	8.211	-3.082
H	6.464	7.478	-1.624
H	7.166	9.529	-0.679
H	6.926	10.222	-2.137
N	10.143	8.720	-0.954
C	10.932	8.881	0.269
C	10.342	9.999	1.130
O	10.085	11.099	0.648
C	12.410	9.153	-0.010
O	13.162	9.337	1.207
H	10.355	9.220	-1.842
H	10.875	8.028	0.791
H	12.798	8.380	-0.503
H	12.493	9.982	-0.556
H	13.454	10.287	1.263
N	10.186	9.701	2.421
C	9.736	10.667	3.409
C	10.899	11.585	3.818
O	10.666	12.608	4.471
C	9.148	9.917	4.593
H	10.418	8.706	2.658
H	9.013	11.236	3.009
H	8.766	9.047	4.282
H	9.864	9.742	5.268
H	8.425	10.466	5.012
N	12.151	11.209	3.473
C	13.329	11.982	3.873
C	14.200	12.319	2.650
O	14.063	11.755	1.546
C	14.174	11.295	4.980
C	13.355	10.960	6.226
C	14.921	10.056	4.474
H	12.195	10.340	2.911
H	13.016	12.848	4.273
H	14.883	11.944	5.262
H	12.402	11.219	6.075
H	13.411	9.979	6.405
H	13.720	11.464	7.007
H	15.631	9.814	5.132
H	14.273	9.303	4.379
H	15.334	10.264	3.589
N	15.078	13.306	2.872
C	16.088	13.696	1.906
C	15.562	14.654	0.836
O	14.486	15.248	0.985
H	14.967	13.768	3.806
H	16.866	14.139	2.388
H	16.459	12.867	1.449
N	16.366	14.770	-0.237
C	16.112	15.615	-1.399
C	15.259	14.801	-2.385
O	15.686	14.429	-3.488
C	17.451	16.178	-1.910
C	17.363	16.999	-3.185
H	17.238	14.166	-0.159
H	15.552	16.378	-1.098
H	17.842	16.756	-1.185
H	18.072	15.403	-2.072
N	18.523	17.310	-3.747
O	16.276	17.331	-3.669
H	19.384	17.007	-3.342
H	18.539	17.856	-4.590
N	14.018	14.540	-1.948
C	13.073	13.680	-2.639
C	11.697	14.332	-2.573
O	11.292	14.806	-1.512
C	13.053	12.312	-1.982
H	13.788	15.026	-1.039
H	13.342	13.600	-3.596
H	13.841	11.779	-2.292
H	13.094	12.416	-0.988
H	12.211	11.832	-2.230
N	10.968	14.327	-3.689
C	9.607	14.855	-3.699
C	8.722	14.046	-4.652
O	8.912	14.035	-5.876
C	9.621	16.344	-4.061
C	8.240	16.957	-4.264
C	8.241	18.263	-5.043
O	9.312	18.921	-5.109
O	7.184	18.595	-5.622
H	11.437	13.929	-4.518
H	9.220	14.816	-2.773
H	10.055	16.828	-3.321
H	10.117	16.443	-4.906
H	7.688	16.311	-4.769
H	7.851	17.143	-3.374
N	7.703	13.417	-4.072
C	6.810	12.616	-4.874
C	7.515	11.284	-5.164
O	8.380	10.847	-4.393
C	6.401	13.404	-6.130
O	5.224	12.887	-6.735
H	7.621	13.551	-3.057
H	5.994	12.413	-4.343
H	6.241	14.356	-5.870
H	7.151	13.360	-6.791
H	5.347	12.855	-7.725
N	7.110	10.662	-6.278
C	7.428	9.286	-6.599
C	8.584	9.275	-7.596
O	8.726	10.160	-8.440
C	6.174	8.548	-7.111
C	4.908	8.848	-6.306
C	3.816	7.787	-6.285
N	4.327	6.513	-5.770
C	4.626	6.216	-4.493
N	4.113	6.925	-3.494
N	5.397	5.154	-4.233
H	6.529	11.277	-6.909
H	7.719	8.812	-5.770
H	6.015	8.826	-8.050
H	6.348	7.572	-7.059
H	5.173	9.039	-5.337
H	4.492	9.712	-6.660
H	3.084	8.108	-5.698
H	3.492	7.659	-7.214
H	4.474	5.764	-6.453
H	3.482	7.676	-3.676
H	4.361	6.703	-2.547
H	5.724	4.584	-4.985
H	5.640	4.935	-3.291
N	9.390	8.219	-7.495
C	10.529	7.994	-8.361
C	10.422	6.573	-8.867
O	9.903	5.718	-8.139
C	11.824	8.136	-7.572

C 12.071 9.532 -7.078
C 11.502 9.992 -5.902
C 12.879 10.395 -7.805
C 11.716 11.285 -5.457
C 13.107 11.690 -7.366
C 12.506 12.138 -6.203
O 12.693 13.427 -5.769
H 9.121 7.557 -6.721
H 10.465 8.610 -9.138
H 11.779 7.526 -6.785
H 12.582 7.874 -8.164
H 10.921 9.377 -5.359
H 13.301 10.081 -8.651
H 11.303 11.599 -4.601
H 13.705 12.300 -7.889
H 13.611 13.515 -5.385
N 10.903 6.351 -10.092
C 10.886 5.027 -10.680
C 11.838 4.109 -9.908
O 12.945 4.496 -9.575
C 11.228 5.083 -12.177
C 11.446 3.705 -12.771
C 10.142 5.807 -12.973
H 11.279 7.192 -10.568
H 9.962 4.650 -10.592
H 12.092 5.584 -12.273
H 11.365 3.012 -12.053
H 10.759 3.530 -13.478
H 12.359 3.654 -13.178
H 10.329 5.705 -13.949
H 9.254 5.405 -12.754
H 10.147 6.775 -12.726
N 11.404 2.871 -9.660
C 12.341 1.848 -9.243
C 12.264 0.658 -10.168
O 11.234 0.360 -10.770
C 12.117 1.460 -7.792
C 10.935 0.585 -7.428
C 11.183 -0.914 -7.686
C 10.606 0.832 -5.962
H 10.396 2.724 -9.787
H 13.272 2.228 -9.284
H 12.952 0.983 -7.471
H 12.032 2.315 -7.257
H 10.127 0.872 -7.964
H 11.962 -1.213 -7.140
H 10.369 -1.427 -7.426
H 11.375 -1.049 -8.655
H 10.377 1.797 -5.829
H 9.826 0.265 -5.694
H 11.398 0.597 -5.398
N 13.416 0.016 -10.274
C 13.541 -1.243 -10.956
C 14.432 -2.180 -10.144
O 15.392 -1.754 -9.494
C 14.037 -0.984 -12.378
C 15.487 -0.586 -12.490
O 13.868 -2.229 -13.044
H 14.223 0.508 -9.817
H 12.635 -1.668 -11.034
H 13.443 -0.303 -12.814
H 16.058 -1.397 -12.648
H 15.613 0.052 -13.256
H 15.790 -0.138 -11.643
H 13.199 -2.804 -12.558
N 14.132 -3.467 -10.201
C 14.980 -4.445 -9.529
C 14.732 -5.870 -9.995
O 14.082 -6.105 -11.012
H 13.288 -3.708 -10.736
H 15.955 -4.207 -9.688
H 14.824 -4.390 -8.526
N 15.206 -6.807 -9.185
C 15.096 -8.232 -9.475
C 14.880 -8.994 -8.178
O 15.318 -8.567 -7.120
C 16.375 -8.740 -10.154
C 16.779 -8.059 -11.462
C 15.958 -8.394 -12.682
N 16.048 -9.812 -12.984
C 15.182 -10.463 -13.750
N 14.145 -9.814 -14.239
N 15.293 -11.768 -13.920
H 15.666 -6.437 -8.320
H 14.307 -8.382 -10.073
H 17.136 -8.631 -9.505
H 16.258 -9.721 -10.344
H 16.746 -7.049 -11.326
H 17.751 -8.292 -11.662
H 15.012 -8.155 -12.498
H 16.310 -7.870 -13.449
H 16.813 -10.334 -12.589
H 13.994 -8.845 -14.073
H 13.469 -10.316 -14.810
H 16.049 -12.264 -13.482
H 14.633 -12.265 -14.481
N 14.271 -10.179 -8.277
C 14.107 -11.044 -7.133
C 14.298 -12.477 -7.631
O 14.158 -12.749 -8.846
C 12.774 -10.772 -6.442
C 11.559 -11.342 -7.153
C 11.001 -10.731 -8.268
C 10.956 -12.496 -6.683
C 9.906 -11.285 -8.935
C 9.838 -13.033 -7.311
C 9.326 -12.445 -8.448
O 8.216 -13.012 -9.040
H 13.936 -10.410 -9.234
H 14.824 -10.851 -6.475
H 12.808 -11.168 -5.524
H 12.651 -9.781 -6.371
H 11.389 -9.869 -8.608
H 11.330 -12.956 -5.877
H 9.547 -10.846 -9.758
H 9.402 -13.856 -6.930
H 8.109 -12.636 -9.958
N 14.593 -13.341 -6.680
C 14.674 -14.777 -6.911
C 13.262 -15.334 -7.083
O 12.526 -15.465 -6.100
C 15.383 -15.506 -5.777
C 15.546 -17.037 -5.918
O 15.121 -17.626 -6.957
O 16.100 -17.656 -4.955
H 14.765 -12.909 -5.739
H 15.150 -14.915 -7.779
H 16.307 -15.110 -5.675
H 14.875 -15.332 -4.922
N 12.902 -15.678 -8.336
C 11.538 -16.127 -8.589
C 11.367 -17.647 -8.391
O 10.244 -18.141 -8.525
C 11.090 -15.670 -9.941
O 12.042 -16.025 -10.922
H 13.633 -15.597 -9.041
H 10.925 -15.667 -7.929
H 10.221 -16.101 -10.169
H 10.987 -14.678 -9.942
H 12.099 -15.303 -11.612
N 12.436 -18.393 -8.040
C 12.310 -19.819 -7.708
C 13.078 -20.115 -6.427
O 14.080 -20.833 -6.437
C 12.826 -20.685 -8.832
H 13.333 -17.878 -8.027
H 11.342 -20.029 -7.561
H 12.123 -21.344 -9.104
H 13.062 -20.114 -9.621
H 13.642 -21.182 -8.531
N 12.637 -19.564 -5.288
C 13.434 -19.606 -4.060
C 13.506 -21.004 -3.449
O 12.841 -21.921 -3.925
C 12.695 -18.659 -3.116
C 11.269 -18.716 -3.588
C 11.325 -18.920 -5.092
H 14.367 -19.243 -4.228
H 12.767 -18.972 -2.169
H 13.055 -17.730 -3.188
H 10.782 -19.477 -3.146
H 10.791 -17.859 -3.365
H 10.589 -19.516 -5.402
H 11.283 -18.048 -5.575
N 14.328 -21.133 -2.406
C 14.520 -22.394 -1.714
C 13.254 -22.675 -0.924
O 12.499 -21.751 -0.644
C 15.745 -22.335 -0.828
H 14.817 -20.249 -2.136
H 14.647 -23.118 -2.384
H 15.714 -23.077 -0.157
H 16.571 -22.431 -1.385
H 15.773 -21.457 -0.347
N 12.993 -23.953 -0.629
C 11.849 -24.310 0.198
C 12.381 -24.975 1.460
O 12.203 -26.173 1.664
C 10.866 -25.237 -0.532
C 10.072 -24.562 -1.634
O 11.678 -26.302 -1.027
H 13.655 -24.637 -1.028
H 11.369 -23.478 0.458
H 10.216 -25.612 0.133
H 10.688 -24.041 -2.230
H 9.593 -25.252 -2.183
H 9.397 -23.936 -1.235
H 12.404 -25.943 -1.624
N 13.088 -24.186 2.267
C 13.660 -24.648 3.519
C 13.358 -23.627 4.617
O 13.907 -23.734 5.712
C 15.159 -24.932 3.339
C 15.967 -23.740 2.825
O 15.402 -22.626 2.776
O 17.144 -23.936 2.442
H 13.194 -23.204 1.917
H 13.190 -25.494 3.773
H 15.528 -25.205 4.225
H 15.254 -25.682 2.688
N 12.477 -22.647 4.335
C 12.179 -21.571 5.279
C 13.104 -20.348 5.140
O 13.110 -19.462 6.003
H 12.037 -22.728 3.397
H 11.217 -21.268 5.151
H 12.250 -21.927 6.229
N 13.896 -20.339 4.066
C 14.755 -19.224 3.687
C 13.935 -18.067 3.145
O 12.986 -18.285 2.394
C 15.694 -19.628 2.577
O 16.759 -20.374 3.066
H 13.845 -21.231 3.501
H 15.235 -18.905 4.513
H 15.187 -20.172 1.907
H 16.044 -18.801 2.133
H 16.519 -21.348 3.088
N 14.416 -16.843 3.391
C 13.909 -15.684 2.687
C 14.214 -15.721 1.194
O 15.033 -16.504 0.689
H 15.161 -16.808 4.110
H 12.904 -15.620 2.816
H 14.312 -14.841 3.085
N 13.528 -14.819 0.489
C 13.672 -14.645 -0.945
C 14.550 -13.420 -1.214
O 14.149 -12.309 -0.887
C 12.290 -14.459 -1.580
C 12.332 -14.265 -3.079
O 11.543 -15.622 -1.215
H 12.869 -14.242 1.071
H 14.132 -15.447 -1.325
H 11.846 -13.654 -1.172

H	12.968	-14.922	-3.494
H	11.418	-14.406	-3.471
H	12.639	-13.334	-3.298
H	10.672	-15.652	-1.717
N	15.698	-13.641	-1.847
C	16.629	-12.560	-2.136
C	16.086	-11.608	-3.209
O	15.519	-12.011	-4.222
C	17.955	-13.164	-2.519
H	15.867	-14.631	-2.111
H	16.786	-12.028	-1.291
H	18.577	-12.447	-2.841
H	18.365	-13.619	-1.726
H	17.826	-13.837	-3.251
N	16.337	-10.298	-3.023
C	15.885	-9.303	-3.976
C	16.735	-8.045	-3.837
O	17.518	-7.914	-2.891
C	14.380	-9.017	-3.804
C	13.863	-8.642	-2.414
C	14.483	-7.394	-1.881
C	12.330	-8.483	-2.472
H	16.867	-10.082	-2.161
H	16.004	-9.662	-4.903
H	14.146	-8.261	-4.424
H	13.883	-9.839	-4.097
H	14.050	-9.415	-1.787
H	14.288	-6.619	-2.491
H	14.117	-7.184	-0.969
H	15.480	-7.502	-1.809
H	11.925	-9.347	-2.763
H	11.995	-8.239	-1.565
H	12.103	-7.763	-3.124
N	16.597	-7.172	-4.822
C	17.206	-5.859	-4.754
C	16.558	-4.907	-5.733
O	15.921	-5.341	-6.700
H	16.029	-7.503	-5.625
H	17.113	-5.492	-3.818
H	18.192	-5.933	-4.960
N	16.699	-3.604	-5.469
C	16.192	-2.639	-6.429
C	16.987	-1.358	-6.318
O	17.745	-1.188	-5.387
C	14.687	-2.411	-6.241
C	14.320	-1.725	-4.966
C	14.213	-0.384	-4.797
C	13.981	-2.334	-3.703
C	13.688	-1.276	-2.827
C	13.953	-3.651	-3.212
N	13.825	-0.110	-3.526
C	13.376	-1.487	-1.490
C	13.608	-3.871	-1.900
C	13.347	-2.794	-1.049
H	17.163	-3.373	-4.593
H	16.339	-3.013	-7.347
H	14.354	-1.846	-6.991
H	14.227	-3.295	-6.248
H	14.394	0.301	-5.505
H	13.661	0.809	-3.147
H	14.181	-4.418	-3.813
H	13.183	-0.718	-0.880
H	13.545	-4.800	-1.556
H	13.131	-2.981	-0.086
N	16.827	-0.502	-7.324
C	17.520	0.762	-7.430
C	16.479	1.862	-7.682
O	15.511	1.644	-8.411
C	18.497	0.778	-8.612
C	19.228	2.102	-8.770
O	19.413	-0.317	-8.497
H	16.135	-0.836	-8.051
H	17.975	0.961	-6.565
H	17.970	0.661	-9.479
H	20.216	1.959	-8.677
H	19.037	2.491	-9.674
H	18.922	2.746	-8.065
H	20.115	-0.263	-9.215
N	16.726	3.011	-7.059
C	16.063	4.270	-7.370
C	17.154	5.303	-7.698
O	18.057	5.525	-6.912
C	15.180	4.767	-6.212
C	14.578	6.137	-6.527
C	14.070	3.802	-5.826
H	17.460	2.931	-6.303
H	15.510	4.145	-8.186
H	15.763	4.886	-5.412
H	14.585	6.282	-7.515
H	13.639	6.170	-6.190
H	15.120	6.847	-6.080
H	13.291	3.932	-6.439
H	14.406	2.863	-5.904
H	13.789	3.981	-4.883
N	17.067	5.951	-8.858
C	17.819	7.170	-9.130
C	16.929	8.357	-8.786
O	15.759	8.374	-9.165
C	18.253	7.226	-10.579
H	16.419	5.513	-9.549
H	18.624	7.193	-8.544
H	17.459	7.398	-11.164
H	18.919	7.962	-10.704
H	18.672	6.355	-10.841
N	17.458	9.293	-7.996
C	16.650	10.325	-7.367
C	16.408	11.526	-8.291
O	16.652	12.663	-7.891
C	17.293	10.748	-6.064
C	17.312	9.593	-5.117
C	18.404	8.874	-4.738
C	16.182	9.029	-4.425
C	16.672	7.943	-3.670
C	14.816	9.295	-4.389
N	18.041	7.913	-3.848
C	15.841	7.159	-2.876
C	13.991	8.505	-3.610
C	14.498	7.461	-2.851
H	18.491	9.217	-7.873
H	15.745	9.926	-7.154
H	18.222	11.010	-6.228
H	16.760	11.458	-5.652
H	19.336	9.032	-5.068
H	18.660	7.273	-3.384
H	14.427	10.052	-4.920
H	16.221	6.396	-2.341
H	13.010	8.698	-3.601
H	13.882	6.923	-2.280
N	15.912	11.224	-9.480
C	15.491	12.207	-10.475
C	14.089	11.820	-10.947
O	13.836	10.660	-11.292
C	16.462	12.257	-11.654
C	16.041	13.215	-12.775
C	16.750	12.969	-14.067
C	16.201	13.790	-15.222
N	14.765	13.493	-15.474
H	15.843	10.182	-9.658
H	15.449	13.105	-10.050
H	17.353	12.551	-11.317
H	16.534	11.340	-12.043
H	15.044	13.118	-12.913
H	16.220	14.160	-12.465
H	17.724	13.195	-13.961
H	16.671	11.997	-14.312
H	16.303	14.764	-15.006
H	16.730	13.583	-16.049
H	14.359	14.240	-15.999
H	14.688	12.640	-15.990
H	14.287	13.400	-14.601
N	13.155	12.777	-10.844
C	11.833	12.651	-11.441
C	11.560	13.967	-12.177
O	12.500	14.706	-12.464
C	10.784	12.244	-10.401
C	10.631	13.250	-9.278
H	13.462	13.613	-10.300
H	11.882	11.942	-12.130
H	9.905	12.147	-10.864
H	11.056	11.366	-10.009
N	9.978	12.873	-8.193
O	11.064	14.388	-9.411
H	9.612	11.934	-8.148
H	9.847	13.497	-7.432
N	10.290	14.266	-12.474
C	9.936	15.478	-13.211
C	10.114	16.724	-12.341
O	10.273	17.829	-12.847
C	8.504	15.392	-13.747
C	8.360	14.396	-14.880
H	9.593	13.573	-12.140
H	10.583	15.561	-13.977
H	7.906	15.114	-13.000
H	8.239	16.294	-14.079
N	7.127	14.103	-15.257
O	9.345	13.902	-15.424
H	6.346	14.538	-14.802
H	6.967	13.448	-15.996
N	10.090	16.528	-11.026
C	10.047	17.606	-10.059
C	11.434	17.979	-9.533
O	11.625	19.103	-9.073
C	9.168	17.165	-8.883
C	7.691	17.043	-9.173
C	7.167	17.307	-10.436
C	6.808	16.687	-8.162
C	5.807	17.221	-10.684
C	5.446	16.599	-8.394
C	4.946	16.870	-9.657
O	3.600	16.782	-9.905
H	10.105	15.513	-10.749
H	9.657	18.427	-10.509
H	9.500	16.271	-8.566
H	9.286	17.832	-8.140
H	7.787	17.565	-11.182
H	7.164	16.489	-7.247
H	5.447	17.410	-11.599
H	4.824	16.341	-7.654
H	3.119	16.592	-9.050
N	12.375	17.020	-9.501
C	13.603	17.210	-8.745
C	14.692	16.285	-9.252
O	14.419	15.213	-9.788
C	13.460	16.849	-7.265
C	12.526	17.730	-6.463
C	12.898	17.683	-5.002
N	11.896	18.379	-4.215
C	11.989	18.608	-2.915
N	10.964	19.140	-2.270
N	13.089	18.266	-2.260
H	12.149	16.173	-10.042
H	13.909	18.163	-8.857
H	13.124	15.905	-7.208
H	14.370	16.897	-6.845
H	12.602	18.668	-6.778
H	11.594	17.402	-6.561
H	12.940	16.735	-4.720
H	13.775	18.128	-4.889
H	11.069	18.712	-4.692
H	10.109	19.358	-2.730
H	11.047	19.332	-1.279
H	13.840	17.816	-2.749
H	13.173	18.453	-1.283
N	15.932	16.681	-8.999
C	17.062	15.816	-9.294
C	18.116	16.013	-8.208
O	18.724	17.064	-8.120
C	17.597	16.054	-10.702
C	18.670	15.055	-11.079
H	16.019	17.625	-8.588
H	16.745	14.873	-9.223
H	16.843	15.969	-11.345
H	17.986	16.969	-10.742
N	19.137	15.125	-12.317
O	19.050	14.207	-10.252

H	18.782	15.820	-12.947
H	19.843	14.488	-12.626
N	18.319	14.985	-7.377
C	19.291	15.028	-6.299
C	20.650	14.446	-6.728
O	21.545	14.337	-5.906
C	18.730	14.287	-5.115
H	17.717	14.154	-7.575
H	19.436	15.989	-6.022
H	17.855	14.692	-4.841
H	18.583	13.324	-5.352
H	19.369	14.339	-4.344
N	20.807	14.055	-7.983
C	22.083	13.549	-8.503
C	22.656	12.426	-7.626
O	23.840	12.413	-7.297
C	23.132	14.662	-8.604
C	22.635	15.841	-9.406
H	19.950	14.135	-8.575
H	21.923	13.174	-9.426
H	23.361	14.983	-7.687
H	23.949	14.304	-9.051
C	22.487	17.152	-9.107
N	22.236	15.691	-10.717
C	21.865	16.892	-11.201
N	21.996	17.783	-10.238
H	22.220	14.842	-11.230
H	22.692	17.591	-8.237
H	21.551	17.062	-12.125
N	21.809	11.434	-7.366
C	22.179	10.360	-6.464
C	21.332	9.153	-6.843
O	20.309	9.304	-7.543
C	22.018	10.805	-5.034
O	20.717	11.227	-4.744
H	20.897	11.498	-7.848
H	23.131	10.128	-6.623
H	22.257	10.040	-4.425
H	22.655	11.564	-4.855
H	20.501	12.056	-5.265
N	21.739	7.994	-6.314
C	20.990	6.754	-6.507
C	21.149	5.912	-5.250
O	22.250	5.843	-4.702
C	21.476	6.009	-7.727
H	22.617	8.054	-5.765
H	20.027	6.974	-6.627
H	22.411	5.683	-7.576
H	20.880	5.224	-7.906
H	21.465	6.617	-8.523
N	20.071	5.205	-4.903
C	20.127	4.221	-3.838
C	19.817	2.841	-4.395
O	18.894	2.679	-5.213
C	19.139	4.570	-2.734
C	19.276	3.632	-1.547
O	19.389	5.924	-2.320
H	19.217	5.419	-5.450
H	21.057	4.208	-3.459
H	18.193	4.525	-3.102
H	18.507	2.988	-1.530
H	20.133	3.116	-1.617
H	19.280	4.159	-0.694
H	20.376	6.113	-2.329
N	20.556	1.857	-3.890
C	20.225	0.456	-4.067
C	19.984	-0.174	-2.709
O	20.762	0.042	-1.773
C	21.313	-0.370	-4.772
C	21.667	0.174	-6.143
O	22.494	-0.446	-3.938
H	21.395	2.184	-3.355
H	19.381	0.389	-4.602
H	20.971	-1.321	-4.914
H	21.114	-0.282	-6.845
H	21.489	1.161	-6.176
H	22.638	0.011	-6.336
H	22.619	0.417	-3.443
N	18.951	-1.012	-2.678
C	18.560	-1.857	-1.556
C	18.812	-3.314	-1.926
O	18.325	-3.777	-2.964
C	17.067	-1.674	-1.233
C	16.657	-0.350	-0.662
C	16.384	-0.076	0.647
C	16.376	0.876	-1.382
C	15.960	1.834	-0.430
C	16.404	1.265	-2.728
N	15.957	1.221	0.795
C	15.582	3.129	-0.784
C	16.026	2.546	-3.076
C	15.592	3.458	-2.119
H	18.399	-1.015	-3.596
H	19.152	-1.635	-0.783
H	16.540	-1.808	-2.078
H	16.796	-2.380	-0.570
H	16.481	-0.731	1.401
H	15.687	1.650	1.657
H	16.698	0.618	-3.436
H	15.313	3.790	-0.076
H	16.066	2.818	-4.037
H	15.285	4.362	-2.409
N	19.458	-4.030	-1.014
C	19.722	-5.437	-1.178
C	19.142	-6.115	0.062
O	19.409	-5.681	1.180
C	21.204	-5.670	-1.329
O	21.483	-7.054	-1.464
H	19.755	-3.481	-0.172
H	19.221	-5.768	-1.964
H	21.532	-5.198	-2.142
H	21.676	-5.327	-0.521
H	22.371	-7.173	-1.906
N	18.316	-7.141	-0.144
C	17.725	-7.773	1.015
C	16.902	-8.999	0.653
O	17.171	-9.713	-0.330
H	18.159	-7.408	-1.108
H	18.457	-8.045	1.664
H	17.131	-7.107	1.499
N	16.021	-9.347	1.590
C	15.246	-10.563	1.400
C	13.839	-10.371	1.962
O	13.638	-9.658	2.944
C	15.995	-11.773	1.960
C	16.196	-11.751	3.451
C	16.886	-13.008	3.933
H	15.948	-8.726	2.392
H	15.160	-10.730	0.416
H	15.480	-12.604	1.720
H	16.899	-11.820	1.518
H	16.761	-10.967	3.693
H	15.309	-11.684	3.900
N	17.896	-12.825	4.785
O	16.508	-14.120	3.544
H	18.403	-13.624	5.166
H	18.152	-11.879	5.047
N	12.896	-11.002	1.250
C	11.486	-11.031	1.589
C	11.204	-12.258	2.461
O	11.650	-13.374	2.166
C	10.668	-11.013	0.296
C	9.256	-11.514	0.459
C	8.255	-10.699	0.972
C	8.949	-12.837	0.163
C	6.958	-11.177	1.153
C	7.672	-13.334	0.370
C	6.661	-12.491	0.813
O	5.373	-12.990	1.004
H	13.285	-11.491	0.394
H	11.267	-10.216	2.126
H	10.632	-10.073	-0.036
H	11.131	-11.591	-0.373
H	8.463	-9.751	1.218
H	9.657	-13.436	-0.204
H	6.249	-10.579	1.523
H	7.475	-14.302	0.201
H	4.749	-12.498	0.405
N	10.518	-11.998	3.579
C	10.074	-13.018	4.516
C	8.550	-12.932	4.565
O	7.996	-11.914	5.000
C	10.699	-12.762	5.906
C	10.392	-13.905	6.871
C	12.205	-12.518	5.820
H	10.325	-10.971	3.722
H	10.318	-13.907	4.154
H	10.274	-11.942	6.282
H	11.243	-14.366	7.120
H	9.958	-13.538	7.692
H	9.776	-14.556	6.429
H	12.455	-11.789	6.455
H	12.687	-13.358	6.063
H	12.440	-12.250	4.887
N	7.868	-13.936	4.020
C	6.412	-13.913	3.926
C	5.724	-14.326	5.238
O	6.382	-14.745	6.190
H	8.452	-14.722	3.674
H	6.104	-12.981	3.676
H	6.111	-14.539	3.189
N	4.385	-14.212	5.279
C	3.640	-14.454	6.510
C	2.645	-13.339	6.830
O	2.480	-12.370	6.068
H	3.948	-13.944	4.381
H	3.138	-15.332	6.432
H	4.290	-14.549	7.283
N	1.985	-13.485	7.991
C	0.897	-12.586	8.363
C	1.465	-11.197	8.648
O	0.793	-10.206	8.335
C	0.113	-13.126	9.539
H	2.312	-14.271	8.578
H	0.256	-12.518	7.598
H	-0.859	-12.913	9.425
H	0.228	-14.119	9.595
H	0.444	-12.709	10.387
N	2.690	-11.157	9.225
C	3.493	-9.941	9.300
C	4.735	-10.105	8.404
O	5.853	-10.343	8.849
C	3.819	-9.563	10.750
C	2.582	-9.206	11.579
C	2.198	-10.270	12.588
O	1.101	-10.859	12.461
O	3.012	-10.514	13.499
H	3.001	-12.077	9.608
H	2.962	-9.159	8.983
H	4.244	-10.338	11.174
H	4.399	-8.774	10.732
H	2.779	-8.378	12.076
H	1.823	-9.091	10.960
N	4.486	-9.995	7.108
C	5.509	-10.085	6.091
C	6.433	-8.880	6.215
O	6.059	-7.791	6.692
C	4.845	-10.182	4.735
H	3.469	-9.835	6.883
H	6.048	-10.915	6.237
H	4.616	-11.136	4.537
H	4.007	-9.635	4.729
H	5.466	-9.840	4.028
N	7.676	-9.100	5.803
C	8.734	-8.124	5.999
C	9.675	-8.213	4.796
O	9.946	-9.321	4.318
C	9.484	-8.505	7.284
C	10.292	-7.420	7.964
C	10.685	-7.896	9.352
N	9.626	-7.626	10.310
C	9.401	-6.445	10.868
N	10.262	-5.459	10.715
N	8.328	-6.253	11.609
H	7.822	-10.019	5.335

H	8.340	-7.212	6.026
H	8.803	-8.849	7.946
H	10.109	-9.266	7.060
H	11.116	-7.245	7.451
H	9.743	-6.606	8.060
H	10.852	-8.873	9.307
H	11.509	-7.412	9.621
H	9.019	-8.391	10.570
H	11.087	-5.570	10.163
H	10.090	-4.569	11.161
H	7.668	-7.001	11.735
H	8.163	-5.370	12.047
N	10.237	-7.062	4.397
C	11.458	-7.043	3.570
C	12.575	-6.369	4.371
O	12.455	-5.196	4.691
C	11.218	-6.354	2.221
C	10.035	-6.981	1.472
C	12.490	-6.356	1.367
C	9.596	-6.216	0.267
H	9.750	-6.207	4.713
H	11.725	-7.993	3.451
H	10.995	-5.396	2.396
H	10.298	-7.910	1.201
H	9.269	-7.051	2.116
H	12.328	-5.831	0.535
H	13.234	-5.942	1.885
H	12.726	-7.295	1.131
H	10.351	-6.150	-0.389
H	8.823	-6.682	-0.170
H	9.313	-5.292	0.534
N	13.612	-7.146	4.712
C	14.777	-6.714	5.480
C	15.920	-6.356	4.525
O	16.284	-7.196	3.695
C	15.272	-7.823	6.391
C	14.260	-8.150	7.469
H	13.509	-8.144	4.361
H	14.524	-5.873	5.959
H	15.428	-8.638	5.846
H	16.114	-7.526	6.827
N	14.017	-9.433	7.675
O	13.637	-7.244	8.017
H	14.495	-10.130	7.137
H	13.353	-9.715	8.371
N	16.340	-5.087	4.529
C	17.311	-4.611	3.544
C	18.477	-3.854	4.183
O	18.376	-3.335	5.295
C	16.685	-3.679	2.500
C	15.474	-4.240	1.802
O	16.355	-2.431	3.117
H	15.930	-4.493	5.267
H	17.688	-5.413	3.053
H	17.369	-3.499	1.764
H	14.639	-3.997	2.306
H	15.407	-3.870	0.870
H	15.537	-5.242	1.747
H	15.928	-2.586	4.015
N	19.591	-3.821	3.427
C	20.682	-2.897	3.574
C	20.751	-2.092	2.290
O	20.319	-2.565	1.233
C	21.968	-3.694	3.844
C	21.823	-4.469	5.160
C	23.075	-4.934	5.860
H	19.583	-4.582	2.675
H	20.470	-2.269	4.315
H	22.102	-4.341	3.120
H	22.719	-3.069	3.937
H	21.289	-3.882	5.808
H	21.232	-5.286	4.975
N	23.246	-6.237	5.881
O	23.822	-4.145	6.446
H	24.086	-6.648	6.328
H	22.554	-6.846	5.458
N	21.157	-0.836	2.406
C	21.128	0.027	1.226
C	22.445	0.801	1.126
O	23.153	0.998	2.125
C	19.877	0.924	1.219
C	19.669	1.811	2.413
C	18.701	1.649	3.367
C	20.353	3.041	2.747
C	19.746	3.547	3.914
C	21.411	3.779	2.189
N	18.782	2.651	4.294
C	20.179	4.718	4.547
C	21.832	4.940	2.811
C	21.220	5.414	3.975
H	21.471	-0.543	3.330
H	21.062	-0.559	0.419
H	19.918	1.522	0.411
H	19.064	0.337	1.142
H	18.033	0.906	3.382
H	18.224	2.723	5.125
H	21.858	3.467	1.349
H	19.733	5.029	5.393
H	22.595	5.452	2.413
H	21.542	6.258	4.387
N	22.764	1.205	-0.111
C	23.893	2.059	-0.430
C	23.308	3.226	-1.203
O	22.718	2.983	-2.245
C	24.919	1.341	-1.324
C	25.623	0.140	-0.692
C	26.444	-0.646	-1.707
C	26.443	0.535	0.504
H	22.102	0.836	-0.850
H	24.287	2.400	0.408
H	24.440	1.016	-2.132
H	25.617	2.001	-1.573
H	24.930	-0.467	-0.286
H	27.142	-0.050	-2.104
H	26.885	-1.419	-1.252
H	25.843	-0.984	-2.431
H	25.854	0.951	1.203
H	26.892	-0.273	0.896
H	27.146	1.199	0.235
N	23.642	4.433	-0.778
C	23.250	5.658	-1.463
C	24.513	6.360	-1.955
O	25.370	6.722	-1.152
C	22.492	6.526	-0.462
C	21.732	7.744	-0.992
C	21.097	8.513	0.168
C	22.545	8.644	-1.873
H	24.218	4.436	0.101
H	22.693	5.421	-2.254
H	21.821	5.940	0.005
H	23.153	6.862	0.215
H	21.016	7.437	-1.614
H	21.874	8.850	0.878
H	20.604	9.305	-0.191
H	20.461	7.914	0.653
H	22.872	8.138	-2.677
H	21.987	9.419	-2.184
H	23.415	9.023	-1.306
N	24.649	6.450	-3.275
C	25.816	7.084	-3.864
C	25.401	8.420	-4.471
O	24.398	8.498	-5.174
C	26.467	6.259	-4.977
C	27.676	6.940	-5.571
O	26.881	4.996	-4.466
H	23.874	6.041	-3.824
H	26.486	7.261	-3.143
H	25.777	6.095	-5.715
H	27.453	7.888	-5.819
H	28.430	6.949	-4.907
H	27.978	6.452	-6.396
H	26.152	4.311	-4.581
N	26.138	9.459	-4.085
C	25.997	10.788	-4.654
C	27.109	11.006	-5.680
O	28.289	10.682	-5.442
C	25.986	11.893	-3.558
C	24.949	11.615	-2.434
C	25.659	13.259	-4.185
C	25.508	10.831	-1.251
H	27.022	9.290	-3.559
H	25.139	10.791	-5.163
H	27.004	11.966	-3.113
H	24.592	12.572	-2.010
H	24.061	11.090	-2.840
H	25.681	14.062	-3.419
H	26.410	13.534	-4.947
H	24.659	13.244	-4.671
H	26.463	11.275	-0.899
H	24.784	10.863	-0.409
H	25.656	9.769	-1.527
N	26.742	11.615	-6.821
C	27.739	12.059	-7.781
C	28.695	13.062	-7.131
O	28.261	13.988	-6.455
H	25.725	11.731	-6.941
H	28.266	11.271	-8.113
H	27.287	12.497	-8.563
N	29.997	12.809	-7.257
C	31.030	13.630	-6.650
C	32.157	13.812	-7.670
O	32.292	13.024	-8.616
C	31.638	13.023	-5.359
C	30.631	12.714	-4.272
O	32.378	11.851	-5.708
H	30.210	11.960	-7.837
H	30.651	14.528	-6.442
H	32.332	13.769	-4.925
H	29.921	13.557	-4.145
H	30.079	11.807	-4.540
H	31.162	12.529	-3.316
H	33.288	12.101	-6.056
N	33.030	14.789	-7.389
C	34.336	14.826	-8.034
C	35.160	13.625	-7.583
O	34.908	12.995	-6.543
C	35.088	16.122	-7.709
C	34.339	17.377	-8.106
O	35.309	16.174	-6.320
H	32.829	15.436	-6.597
H	34.207	14.765	-9.025
H	35.999	16.091	-8.138
H	33.235	17.262	-8.078
H	34.630	18.248	-7.483
H	34.648	17.592	-9.117
H	36.029	16.849	-6.179
N	36.216	13.354	-8.335
C	37.122	12.262	-7.985
C	37.800	12.516	-6.628
O	37.947	11.581	-5.859
C	38.105	12.026	-9.144
C	37.383	11.758	-10.469
C	38.285	11.590	-11.677
O	38.121	12.342	-12.655
O	39.147	10.709	-11.630
H	36.341	13.959	-9.162
H	36.611	11.396	-7.941
H	38.635	12.836	-9.256
H	38.634	11.236	-8.931
H	36.861	10.921	-10.365
H	36.782	12.526	-10.649
N	38.142	13.772	-6.282
C	38.737	14.108	-4.986
C	37.791	13.803	-3.815
O	38.251	13.531	-2.707
C	39.030	15.577	-4.893
H	37.944	14.483	-7.021
H	39.579	13.562	-4.864
H	40.054	15.683	-4.662
H	38.975	15.984	-5.849
H	38.394	16.167	-4.196
N	36.464	14.039	-3.997
C	35.424	13.850	-2.973
C	34.778	12.473	-3.085
O	33.852	12.169	-2.285

C	34.372	14.969	-3.121
C	34.902	16.321	-2.761
H	36.217	14.139	-5.017
H	35.870	13.902	-1.954
H	34.012	14.984	-4.168
H	33.505	14.765	-2.460
N	34.704	17.331	-3.523
O	35.367	16.526	-1.614
H	34.221	17.231	-4.442
H	35.051	18.264	-3.223
N	35.351	11.456	-3.914
C	34.749	10.129	-4.067
C	34.769	9.308	-2.767
O	33.877	8.472	-2.571
C	35.393	9.359	-5.208
H	36.162	11.788	-4.457
H	33.784	10.242	-4.355
H	36.157	8.817	-4.858
H	34.718	8.749	-5.624
H	35.730	10.000	-5.897
N	35.805	9.413	-1.877
C	35.835	8.709	-0.571
C	34.611	9.015	0.274
O	33.986	8.080	0.840
C	37.124	9.047	0.210
C	37.197	10.500	0.604
C	37.712	11.495	-0.131
C	36.756	11.068	1.786
C	37.008	12.407	1.681
C	36.180	10.530	2.941
N	37.593	12.686	0.504
C	36.718	13.283	2.728
C	35.890	11.378	3.957
C	36.172	12.765	3.855
H	36.631	9.969	-2.191
H	35.839	7.611	-0.759
H	37.187	8.413	1.121
H	38.007	8.790	-0.413
H	38.199	11.377	-1.099
H	37.958	13.606	0.165
H	35.985	9.466	3.023
H	36.961	14.331	2.647
H	35.441	10.991	4.864
H	35.957	13.408	4.701
N	34.075	10.344	0.316
C	32.800	10.708	0.973
C	31.675	10.787	-0.031
O	30.748	11.626	0.145
C	32.958	12.013	1.791
C	33.450	13.200	0.946
C	33.520	14.502	1.750
C	34.224	15.578	0.912
N	34.388	16.833	1.654
H	34.555	11.022	-0.311
H	32.507	9.904	1.682
H	31.989	12.271	2.274
H	33.679	11.827	2.606
H	34.469	12.983	0.569
H	32.781	13.351	0.072
H	32.491	14.831	2.002
H	34.079	14.333	2.695
H	35.226	15.199	0.590
H	33.623	15.757	-0.012
H	34.957	17.497	1.074
H	33.459	17.273	1.845
H	34.882	16.671	2.562
N	31.486	9.712	-0.956
C	30.336	9.649	-1.874
C	29.223	8.789	-1.332
O	28.048	9.218	-1.425
C	30.741	9.258	-3.313
O	31.247	7.967	-3.453
H	32.148	8.909	-0.901
H	29.905	10.670	-1.976
H	29.868	9.277	-3.968
H	31.470	10.002	-3.704
H	32.193	7.998	-3.127
N	29.461	7.670	-0.468
C	28.470	6.572	-0.390
C	27.927	6.413	1.004
O	28.639	5.872	1.875
C	29.056	5.230	-0.915
C	28.039	4.111	-0.904
O	29.466	5.451	-2.265
H	30.410	7.522	-0.060
H	27.628	6.784	-1.060
H	29.872	4.993	-0.370
H	28.498	3.227	-1.029
H	27.547	4.101	-0.029
H	27.376	4.240	-1.647
H	29.986	6.309	-2.339
N	26.683	6.849	1.351
C	26.020	6.432	2.600
C	25.613	4.943	2.607
O	25.233	4.339	1.565
C	24.833	7.365	2.927
C	25.241	8.852	3.113
C	24.046	9.769	2.864
C	25.801	9.127	4.506
H	26.220	7.528	0.718
H	26.742	6.558	3.422
H	24.106	7.290	2.102
H	24.314	7.004	3.843
H	26.021	9.141	2.376
H	23.179	9.450	3.480
H	24.310	10.819	3.100
H	23.774	9.722	1.791
H	26.783	8.632	4.616
H	25.959	10.211	4.672
H	25.125	8.732	5.292
N	25.600	4.276	3.792
C	25.108	2.940	4.018
C	24.078	2.981	5.143
O	24.191	3.791	6.079
C	26.251	1.950	4.326
C	27.025	2.279	5.601
C	25.771	0.507	4.301
H	25.895	4.821	4.627
H	24.643	2.624	3.191
H	26.910	2.000	3.566
H	27.008	3.266	5.753
H	26.600	1.813	6.376
H	27.971	1.972	5.503
H	25.304	0.329	3.435
H	26.557	-0.104	4.391
H	25.139	0.356	5.061
N	23.034	2.180	4.971
C	22.011	2.090	6.000
C	21.219	0.807	5.857
O	21.608	-0.097	5.127
H	23.016	1.655	4.088
H	22.445	2.117	6.909
H	21.389	2.878	5.925
N	20.174	0.701	6.674
C	19.318	-0.477	6.690
C	17.879	0.003	6.835
O	17.638	0.994	7.511
C	19.726	-1.451	7.784
C	19.938	-0.792	9.091
C	19.164	-0.618	10.197
N	21.173	-0.249	9.373
C	21.144	0.269	10.602
N	19.927	0.084	11.115
H	20.025	1.526	7.294
H	19.405	-0.948	5.819
H	19.008	-2.147	7.887
H	20.579	-1.908	7.512
H	21.958	-0.241	8.763
H	18.233	-0.932	10.325
H	21.902	0.717	11.060
N	16.974	-0.624	6.077
C	15.551	-0.336	6.076
C	14.800	-1.662	6.270
O	15.093	-2.663	5.614
C	15.088	0.335	4.785
C	15.399	1.819	4.589
O	15.838	2.463	5.569
O	15.213	2.325	3.424
H	17.401	-1.373	5.458
H	15.354	0.224	6.875
H	15.498	-0.175	4.007
H	14.081	0.216	4.717
N	13.790	-1.668	7.158
C	12.871	-2.808	7.234
C	11.502	-2.338	6.745
O	10.986	-1.354	7.247
C	12.732	-3.386	8.647
C	11.975	-4.698	8.666
O	14.022	-3.642	9.177
H	13.726	-0.833	7.753
H	13.197	-3.520	6.622
H	12.269	-2.695	9.240
H	11.006	-4.533	8.870
H	12.047	-5.149	7.772
H	12.357	-5.305	9.368
H	14.664	-2.906	8.926
N	10.927	-3.045	5.768
C	9.664	-2.701	5.169
C	8.578	-3.654	5.670
O	8.829	-4.854	5.771
C	9.740	-2.796	3.655
C	10.647	-1.759	3.024
C	12.000	-2.016	2.862
C	10.156	-0.515	2.670
C	12.818	-1.056	2.287
C	10.983	0.449	2.113
C	12.317	0.168	1.933
H	11.493	-3.887	5.476
H	9.411	-1.787	5.497
H	10.095	-3.687	3.417
H	8.832	-2.660	3.289
H	12.384	-2.880	3.153
H	9.186	-0.305	2.817
H	13.798	-1.276	2.134
H	10.599	1.345	1.847
H	12.929	0.865	1.539
N	7.403	-3.110	6.012
C	6.265	-3.965	6.299
C	5.049	-3.452	5.532
O	5.065	-2.365	4.959
C	5.983	-4.047	7.808
C	7.156	-4.535	8.621
O	5.602	-2.761	8.281
H	7.388	-2.085	6.049
H	6.468	-4.892	5.981
H	5.207	-4.690	7.961
H	7.223	-5.536	8.567
H	8.009	-4.135	8.273
H	7.044	-4.270	9.584
H	6.189	-2.047	7.880
N	3.937	-4.197	5.635
C	2.684	-3.858	4.962
C	1.858	-2.749	5.628
O	0.979	-2.216	4.964
C	1.796	-5.103	4.879
C	2.399	-6.251	4.100
C	2.753	-5.876	2.679
C	1.812	-6.475	1.657
N	2.217	-6.082	0.284
H	4.049	-5.042	6.241
H	2.899	-3.564	4.004
H	1.612	-5.414	5.812
H	0.935	-4.839	4.442
H	3.234	-6.564	4.571
H	1.743	-7.016	4.081
H	2.725	-4.877	2.591
H	3.683	-6.195	2.481
H	1.835	-7.471	1.735
H	0.884	-6.147	1.833
H	1.566	-6.457	-0.376
H	2.227	-5.084	0.212
H	3.131	-6.439	0.091
N	2.145	-2.377	6.887
C	1.328	-1.448	7.660
C	2.104	-0.155	7.942

O	3.313	-0.181	8.175
C	0.871	-2.135	8.965
C	0.287	-3.520	8.684
C	1.990	-2.227	9.999
H	3.018	-2.820	7.266
H	0.521	-1.207	7.114
H	0.137	-1.586	9.356
H	0.957	-4.218	8.932
H	-0.543	-3.645	9.226
H	0.068	-3.595	7.712
H	1.950	-3.119	10.447
H	2.871	-2.118	9.540
H	1.870	-1.503	10.677
N	1.422	1.002	7.900
C	2.111	2.265	8.147
C	2.606	2.277	9.593
O	1.930	1.764	10.480
C	1.247	3.477	7.769
C	1.183	3.734	6.267
C	1.267	5.186	5.837
C	-0.080	5.782	5.486
N	0.021	7.236	5.230
H	0.423	0.915	7.689
H	2.899	2.333	7.538
H	0.323	3.314	8.099
H	1.633	4.282	8.209
H	1.941	3.218	5.819
H	0.315	3.338	5.908
H	1.667	5.721	6.585
H	1.863	5.251	5.033
H	-0.435	5.325	4.666
H	-0.715	5.622	6.247
H	0.981	7.488	5.099
H	-0.498	7.469	4.406
H	-0.351	7.741	6.010
N	3.817	2.812	9.880
C	4.450	2.615	11.181
C	4.100	3.745	12.162
O	3.421	3.440	13.130
C	5.944	2.588	10.815
C	6.057	3.403	9.515
C	4.647	3.650	9.001
O	4.379	4.915	12.156
H	4.198	1.720	11.574
H	6.498	3.012	11.531
H	6.260	1.653	10.659
H	6.530	4.272	9.704
H	6.601	2.887	8.842
H	4.398	4.612	9.088
H	4.558	3.364	8.048
C	25.394	16.360	2.911
N	23.336	14.476	1.147
O	19.040	12.852	-0.115
S	17.457	6.316	0.468
C	24.881	15.989	4.282
N	20.375	11.667	-1.730
O	13.423	5.614	2.564
C	26.049	15.511	1.962
N	15.150	7.315	2.499
O	23.986	18.389	-1.499
C	26.294	14.018	2.072
N	15.765	5.094	3.060
O	24.740	16.756	-3.067
C	26.408	16.274	0.886
N	23.993	16.049	-0.938
C	27.101	15.741	-0.350
C	25.990	17.619	1.132
C	26.261	18.841	0.283
C	25.375	17.652	2.379
C	24.753	18.877	3.007
C	23.275	14.870	-1.267
C	22.937	14.013	-0.138
C	22.713	14.581	-2.589
C	22.069	12.844	-0.290
C	21.809	13.438	-2.741
C	21.473	12.563	-1.608
C	21.707	12.087	0.914
C	22.937	13.790	2.337
C	22.139	12.566	2.228
C	19.106	12.076	-1.261
C	17.861	11.632	-1.959
C	16.903	10.838	-1.058
C	17.628	9.740	-0.268
C	16.645	8.997	0.634
C	17.349	7.892	1.477
C	16.564	7.365	2.692
C	17.920	5.387	1.822
C	17.025	5.820	3.071
C	14.649	5.989	2.696
C	24.232	17.081	-1.865
C	24.947	17.570	-4.155
C	23.636	18.205	-4.606
C	25.988	18.638	-3.838
C	25.474	16.684	-5.275
Ir	24.226	16.329	1.071
H	25.135	14.948	4.563
H	23.789	16.132	4.331
H	25.313	16.665	5.040
H	20.381	10.972	-2.502
H	14.987	7.538	1.537
H	25.851	13.496	1.194
H	25.835	13.574	2.975
H	27.380	13.793	2.074
H	15.835	4.415	2.329
H	27.228	16.515	-1.134
H	26.490	14.931	-0.807
H	28.099	15.327	-0.101
H	26.533	18.579	-0.758
H	25.356	19.486	0.228
H	27.086	19.439	0.722
H	23.983	19.294	2.318
H	25.525	19.655	3.184
H	24.242	18.641	3.962
H	22.921	15.207	-3.445
H	21.300	13.321	-3.670
H	21.030	11.250	0.852
H	23.195	14.166	3.309
H	21.830	12.032	3.109
H	17.343	12.528	-2.356
H	18.132	11.006	-2.832
H	16.115	10.372	-1.696
H	16.376	11.524	-0.362
H	18.104	9.021	-0.974
H	18.413	10.193	0.373
H	15.865	8.570	-0.038
H	16.155	9.724	1.321
H	18.264	8.371	1.753
H	16.791	8.065	3.470
H	17.674	4.370	1.601
H	18.929	5.656	2.053
H	17.552	5.674	3.991
H	23.795	18.789	-5.538
H	22.878	17.419	-4.805
H	23.242	18.897	-3.837
H	26.258	19.197	-4.761
H	26.906	18.164	-3.439
H	25.599	19.368	-3.104
H	25.671	17.272	-6.198
H	24.744	15.883	-5.515
H	26.424	16.196	-4.972
N	22.408	17.269	1.137
C	21.375	16.832	2.000
O	21.674	16.339	3.264
C	19.963	16.845	1.531
H	19.578	15.810	1.630
H	19.945	17.117	0.453
C	19.099	17.836	2.327
H	19.465	18.872	2.137
H	19.190	17.631	3.416
C	17.627	17.718	1.904
H	17.511	18.025	0.842
H	17.327	16.651	1.977
C	16.682	18.559	2.788
C	16.810	20.066	2.472
C	15.226	18.100	2.575
H	16.938	18.394	3.860
C	15.827	20.894	3.311
H	16.611	20.248	1.393
H	17.838	20.422	2.694
C	14.247	18.935	3.410
H	14.952	18.180	1.499
H	15.116	17.035	2.873
C	14.387	20.424	3.083
H	15.912	21.967	3.029
H	16.083	20.807	4.391
H	13.205	18.606	3.197
H	14.436	18.769	4.494
H	13.700	21.011	3.733
H	14.093	20.609	2.026
N	25.294	11.144	-20.758
C	26.171	9.959	-20.534
C	25.509	8.678	-21.019
O	26.019	7.599	-20.749
C	26.476	9.693	-19.056
C	26.762	10.932	-18.238
O	27.754	11.629	-18.546
O	25.971	11.203	-17.327
H	24.387	11.074	-21.178
H	26.994	10.106	-21.093
H	25.684	9.222	-18.662
H	27.272	9.087	-19.013
N	24.357	8.783	-21.685
C	23.702	7.593	-22.199
C	24.720	6.846	-23.042
O	24.889	5.636	-22.918
C	22.470	7.985	-23.013
C	21.889	6.861	-23.829
C	20.566	7.246	-24.466
O	19.907	6.355	-25.001
O	20.211	8.450	-24.425
H	23.997	9.739	-21.793
H	23.401	7.017	-21.447
H	21.779	8.289	-22.378
H	22.739	8.703	-23.632
H	22.512	6.644	-24.555
H	21.718	6.097	-23.238
N	25.428	7.634	-23.860
C	26.397	7.130	-24.817
C	27.623	6.539	-24.115
O	28.090	5.446	-24.468
C	26.767	8.277	-25.745
H	25.216	8.657	-23.745
H	25.967	6.402	-25.369
H	27.118	7.911	-26.606
H	25.957	8.835	-25.927
H	27.470	8.843	-25.314
N	28.130	7.254	-23.094
C	29.311	6.820	-22.375
C	29.052	5.573	-21.524
O	29.959	4.775	-21.334
H	27.604	8.123	-22.880
H	30.056	6.618	-23.032
H	29.636	7.566	-21.771
N	27.834	5.419	-20.975
C	27.530	4.251	-20.137
C	27.242	2.996	-20.978
O	27.793	1.928	-20.716
C	26.401	4.587	-19.142
C	26.892	5.647	-18.152
C	25.903	3.331	-18.436
C	25.805	6.296	-17.358
H	27.161	6.166	-21.188
H	28.366	4.056	-19.613
H	25.646	4.971	-19.655
H	27.545	5.211	-17.529
H	27.383	6.350	-18.674
H	25.144	3.568	-17.832
H	25.593	2.669	-19.115
H	26.645	2.935	-17.899
H	25.315	5.605	-16.823
H	26.198	6.975	-16.734
H	25.159	6.752	-17.974
N	26.427	3.147	-22.033
C	26.046	2.047	-22.901
C	27.277	1.350	-23.448

O	28.166	1.981	-24.003
C	25.148	2.514	-24.063
C	24.779	1.362	-24.972
O	23.962	3.127	-23.545
H	26.093	4.130	-22.170
H	25.516	1.381	-22.362
H	25.655	3.212	-24.610
H	25.593	0.810	-25.176
H	24.094	0.778	-24.527
H	24.399	1.711	-25.833
H	24.148	4.078	-23.277
N	27.299	0.028	-23.333
C	28.411	-0.750	-23.820
C	28.802	-1.884	-22.898
O	28.023	-2.309	-22.031
H	26.462	-0.381	-22.871
H	28.182	-1.132	-24.738
H	29.217	-0.140	-23.958
N	30.020	-2.365	-23.129
C	30.567	-3.493	-22.411
C	31.625	-2.991	-21.429
O	32.532	-2.243	-21.771
C	31.052	-4.568	-23.391
C	31.678	-5.733	-22.664
O	29.914	-5.046	-24.138
H	30.545	-1.851	-23.878
H	29.838	-3.931	-21.885
H	31.704	-4.151	-24.047
H	31.273	-6.596	-22.980
H	32.667	-5.757	-22.836
H	31.524	-5.647	-21.675
H	29.115	-5.126	-23.535
N	31.477	-3.401	-20.170
C	32.404	-3.060	-19.112
C	32.959	-4.300	-18.441
O	32.336	-5.349	-18.410
C	31.653	-2.226	-18.103
C	31.157	-0.926	-18.607
C	29.978	-0.675	-19.225
C	31.836	0.325	-18.496
C	30.991	1.288	-19.046
C	33.047	0.712	-17.934
N	29.860	0.653	-19.458
C	31.309	2.639	-19.057
C	33.375	2.043	-17.946
C	32.525	2.988	-18.527
H	30.624	-3.995	-20.016
H	33.180	-2.565	-19.528
H	30.857	-2.744	-17.779
H	32.256	-2.034	-17.324
H	29.299	-1.368	-19.471
H	29.062	1.109	-19.872
H	33.664	0.039	-17.530
H	30.676	3.313	-19.435
H	34.233	2.342	-17.535
H	32.819	3.948	-18.553
N	34.176	-4.195	-17.907
C	34.826	-5.312	-17.253
C	35.328	-4.887	-15.880
O	35.853	-3.791	-15.747
C	36.042	-5.755	-18.086
C	35.716	-6.041	-19.530
C	35.712	-5.012	-20.473
C	35.325	-7.314	-19.943
C	35.382	-5.259	-21.804
C	34.977	-7.575	-21.267
C	35.024	-6.545	-22.200
O	34.665	-6.714	-23.524
H	34.607	-3.251	-18.003
H	34.152	-6.045	-17.124
H	36.719	-5.026	-18.052
H	36.410	-6.584	-17.675
H	35.949	-4.082	-20.191
H	35.294	-8.058	-19.275
H	35.403	-4.516	-22.473
H	34.697	-8.490	-21.542
H	34.217	-5.884	-23.847
N	35.259	-5.762	-14.881
C	35.757	-5.385	-13.565
C	37.104	-6.038	-13.335
O	37.661	-6.678	-14.230
C	34.779	-5.571	-12.397
C	34.457	-6.985	-12.002
H	34.848	-6.670	-15.109
H	35.905	-4.395	-13.577
H	35.166	-5.090	-11.592
H	33.915	-5.098	-12.641
N	33.427	-7.113	-11.175
O	35.131	-7.938	-12.400
H	32.915	-6.322	-10.857
H	33.154	-8.033	-10.861
N	37.630	-5.779	-12.135
C	38.951	-6.230	-11.753
C	38.972	-7.761	-11.637
O	40.041	-8.338	-11.764
C	39.383	-5.425	-10.515
C	38.793	-5.883	-9.169
C	37.379	-5.441	-8.832
H	37.004	-5.227	-11.506
H	39.597	-5.976	-12.478
H	40.387	-5.468	-10.438
H	39.120	-4.460	-10.650
H	38.824	-6.902	-9.146
H	39.416	-5.559	-8.430
N	36.927	-5.838	-7.649
O	36.699	-4.746	-9.584
H	35.987	-5.571	-7.339
H	37.520	-6.408	-7.056
N	37.824	-8.427	-11.386
C	37.782	-9.888	-11.231
C	37.693	-10.636	-12.569
O	37.633	-11.862	-12.542
C	36.541	-10.305	-10.417
C	36.518	-9.904	-8.941
C	35.155	-10.219	-8.313
C	37.656	-10.586	-8.184
H	36.986	-7.819	-11.314
H	38.630	-10.183	-10.761
H	35.744	-9.892	-10.852
H	36.474	-11.299	-10.460
H	36.694	-8.923	-8.865
H	34.978	-11.199	-8.386
H	35.166	-9.949	-7.352
H	34.445	-9.712	-8.798
H	38.531	-10.311	-8.582
H	37.626	-10.313	-7.223
H	37.553	-11.578	-8.254
N	37.472	-9.935	-13.688
C	37.156	-10.602	-14.942
C	35.660	-10.652	-15.292
O	35.299	-11.094	-16.386
H	37.545	-8.910	-13.579
H	37.653	-10.138	-15.707
H	37.516	-11.559	-14.916
N	34.765	-10.159	-14.426
C	33.349	-10.143	-14.775
C	33.063	-9.169	-15.920
O	33.802	-8.202	-16.104
C	32.544	-9.830	-13.526
O	32.791	-10.840	-12.557
H	35.150	-9.813	-13.542
H	33.092	-11.067	-15.087
H	32.832	-8.959	-13.162
H	31.582	-9.829	-13.750
H	33.710	-10.725	-12.185
N	31.963	-9.426	-16.646
C	31.486	-8.650	-17.777
C	30.079	-8.125	-17.538
O	29.137	-8.875	-17.256
C	31.355	-9.477	-19.077
C	31.466	-8.631	-20.317
O	32.339	-10.498	-19.122
H	31.446	-10.295	-16.300
H	32.101	-7.859	-17.907
H	30.429	-9.916	-19.088
H	30.650	-8.053	-20.418
H	32.276	-8.038	-20.263
H	31.549	-9.215	-21.131
H	32.967	-10.423	-18.337
N	29.946	-6.816	-17.714
C	28.729	-6.053	-17.498
C	28.354	-5.487	-18.868
O	29.105	-4.671	-19.396
C	29.100	-5.010	-16.420
C	28.116	-3.922	-16.158
C	26.782	-4.219	-15.930
C	28.551	-2.612	-15.972
C	25.871	-3.207	-15.645
C	27.645	-1.601	-15.706
C	26.301	-1.897	-15.542
H	30.849	-6.350	-18.042
H	28.019	-6.693	-17.253
H	29.248	-5.513	-15.565
H	29.965	-4.590	-16.706
H	26.466	-5.161	-15.968
H	29.527	-2.405	-16.031
H	24.899	-3.440	-15.517
H	27.969	-0.642	-15.633
H	25.644	-1.169	-15.350
N	27.243	-5.929	-19.464
C	26.784	-5.409	-20.747
C	25.501	-4.627	-20.516
O	24.535	-5.141	-19.972
C	26.606	-6.573	-21.737
C	27.901	-7.386	-21.813
C	26.133	-6.047	-23.086
C	27.792	-8.672	-22.624
H	26.739	-6.672	-18.934
H	27.471	-4.752	-21.046
H	25.891	-7.165	-21.390
H	28.613	-6.812	-22.217
H	28.178	-7.623	-20.881
H	25.966	-6.814	-23.703
H	25.288	-5.529	-22.964
H	26.834	-5.453	-23.476
H	27.531	-8.446	-23.560
H	28.675	-9.135	-22.621
H	27.100	-9.259	-22.212
N	25.520	-3.334	-20.789
C	24.415	-2.474	-20.390
C	23.998	-1.532	-21.519
O	24.859	-1.085	-22.282
C	24.808	-1.697	-19.105
C	25.931	-0.694	-19.339
C	23.599	-1.041	-18.455
H	26.357	-2.998	-21.292
H	23.623	-3.059	-20.169
H	25.147	-2.368	-18.442
H	25.541	0.215	-19.483
H	26.533	-0.676	-18.541
H	26.455	-0.963	-20.147
H	23.236	-0.338	-19.066
H	22.898	-1.734	-18.286
H	23.875	-0.624	-17.589
N	22.698	-1.198	-21.536
C	22.123	-0.096	-22.295
C	21.507	0.918	-21.348
O	20.698	0.572	-20.479
C	21.074	-0.590	-23.306
C	20.475	0.522	-24.136
O	21.729	-1.516	-24.177
H	22.105	-1.833	-20.924
H	22.857	0.355	-22.810
H	20.344	-1.082	-22.807
H	20.206	1.288	-23.544
H	21.145	0.851	-24.807
H	19.663	0.189	-24.625
H	22.201	-2.227	-23.643
N	21.923	2.177	-21.499
C	21.301	3.285	-20.790
C	20.255	3.898	-21.709
O	20.595	4.316	-22.819
C	22.325	4.308	-20.371
H	22.719	2.291	-22.159
H	20.847	2.937	-19.975

H	21.906	5.218	-20.340
H	22.682	4.082	-19.462
H	23.084	4.320	-21.026
N	19.003	3.912	-21.245
C	17.930	4.628	-21.904
C	17.927	6.118	-21.585
O	18.539	6.589	-20.616
H	18.871	3.360	-20.365
H	18.008	4.506	-22.910
H	17.037	4.230	-21.628
N	17.176	6.867	-22.405
C	17.134	8.320	-22.303
C	16.422	8.762	-21.017
O	16.635	9.874	-20.526
C	16.456	8.883	-23.542
H	16.636	6.323	-23.108
H	18.076	8.674	-22.280
H	16.409	9.880	-23.475
H	16.980	8.630	-24.355
H	15.530	8.512	-23.615
N	15.593	7.853	-20.488
C	14.683	8.030	-19.367
C	15.341	7.741	-18.003
O	14.719	8.004	-16.978
C	13.520	7.043	-19.557
C	13.885	5.551	-19.432
O	14.751	5.020	-20.236
O	13.303	4.900	-18.504
H	15.663	6.907	-21.003
H	14.406	9.005	-19.351
H	12.822	7.250	-18.867
H	13.133	7.193	-20.469
N	16.562	7.180	-17.999
C	17.263	6.774	-16.778
C	17.334	5.257	-16.533
O	17.846	4.841	-15.484
H	16.972	7.058	-18.956
H	18.216	7.146	-16.801
H	16.812	7.214	-15.972
N	16.854	4.423	-17.463
C	16.894	2.965	-17.313
C	18.261	2.373	-17.645
O	18.984	2.858	-18.517
C	15.809	2.308	-18.147
H	16.451	4.898	-18.299
H	16.668	2.735	-16.352
H	14.973	2.223	-17.604
H	15.623	2.866	-18.956
H	16.110	1.399	-18.435
N	18.638	1.323	-16.892
C	19.756	0.462	-17.249
C	19.179	-0.930	-17.418
O	18.375	-1.380	-16.612
C	20.859	0.405	-16.181
C	21.494	1.740	-15.796
C	22.427	1.600	-14.599
C	22.233	2.363	-16.983
H	18.057	1.187	-16.036
H	20.127	0.760	-18.116
H	20.461	0.014	-15.356
H	21.583	-0.184	-16.526
H	20.766	2.395	-15.572
H	23.160	0.957	-14.821
H	22.822	2.492	-14.378
H	21.911	1.260	-13.813
H	21.587	2.519	-17.729
H	22.637	3.233	-16.701
H	22.954	1.741	-17.287
N	19.577	-1.597	-18.489
C	19.059	-2.913	-18.779
C	20.219	-3.651	-19.414
O	21.028	-3.041	-20.099
C	17.789	-2.878	-19.644
C	16.696	-1.980	-19.108
O	18.169	-2.416	-20.935
H	20.272	-1.104	-19.080
H	18.825	-3.363	-17.933
H	17.428	-3.820	-19.732
H	15.801	-2.290	-19.442
H	16.693	-2.002	-18.104
H	16.847	-1.035	-19.412
H	18.825	-1.655	-20.861
N	20.368	-4.942	-19.139
C	21.401	-5.697	-19.810
C	21.646	-7.018	-19.111
O	20.705	-7.633	-18.618
H	19.717	-5.327	-18.446
H	21.128	-5.871	-20.766
H	22.258	-5.164	-19.824
N	22.897	-7.449	-19.120
C	23.272	-8.763	-18.642
C	24.610	-8.675	-17.933
O	25.457	-7.826	-18.212
C	23.359	-9.816	-19.760
C	22.078	-9.996	-20.550
O	24.402	-9.386	-20.620
H	23.588	-6.760	-19.501
H	22.584	-9.076	-17.983
H	23.618	-10.701	-19.352
H	22.287	-10.084	-21.528
H	21.600	-10.823	-20.244
H	21.477	-9.203	-20.415
H	24.514	-8.385	-20.572
N	24.754	-9.547	-16.969
C	25.927	-9.581	-16.141
C	26.470	-10.998	-16.152
O	25.736	-11.976	-15.997
C	25.612	-9.168	-14.705
C	26.838	-8.805	-13.919
C	27.625	-9.758	-13.315
C	27.227	-7.472	-13.817
C	28.761	-9.406	-12.593
C	28.371	-7.114	-13.132
C	29.140	-8.074	-12.514
O	30.234	-7.682	-11.808
H	23.950	-10.210	-16.855
H	26.621	-8.980	-16.535
H	25.009	-8.378	-14.734
H	25.162	-9.931	-14.254
H	27.383	-10.731	-13.390
H	26.667	-6.766	-14.249
H	29.303	-10.112	-12.134
H	28.644	-6.146	-13.084
H	30.869	-7.200	-12.413
N	27.776	-11.092	-16.326
C	28.437	-12.380	-16.321
C	29.550	-12.331	-15.293
O	30.523	-11.601	-15.501
C	28.969	-12.690	-17.714
C	29.676	-14.025	-17.739
C	29.723	-14.660	-19.120
O	30.099	-15.845	-19.186
O	29.373	-13.956	-20.119
H	28.269	-10.190	-16.462
H	27.788	-13.097	-16.079
H	28.209	-12.741	-18.320
H	29.616	-12.002	-17.948
H	30.608	-13.892	-17.446
H	29.193	-14.649	-17.147
N	29.385	-13.091	-14.195
C	30.354	-13.064	-13.115
C	31.420	-14.132	-13.334
O	31.091	-15.259	-13.683
C	29.723	-13.241	-11.769
O	30.710	-13.489	-10.772
H	28.532	-13.673	-14.199
H	30.811	-12.169	-13.122
H	29.225	-12.413	-11.518
H	29.097	-14.018	-11.790
H	30.600	-14.418	-10.424
N	32.678	-13.781	-13.037
C	33.797	-14.708	-13.164
C	34.004	-15.525	-11.881
O	34.833	-16.424	-11.882
C	35.043	-13.932	-13.527
H	32.782	-12.797	-12.711
H	33.607	-15.349	-13.920
H	34.834	-13.275	-14.253
H	35.380	-13.437	-12.724
H	35.753	-14.561	-13.847
N	33.246	-15.249	-10.794
C	33.423	-15.933	-9.517
C	32.056	-16.281	-8.939
O	31.024	-15.718	-9.351
C	34.204	-15.124	-8.451
C	35.672	-14.967	-8.815
C	33.540	-13.770	-8.153
H	32.532	-14.508	-10.958
H	33.915	-16.788	-9.685
H	34.166	-15.642	-7.596
H	35.852	-14.019	-9.078
H	36.240	-15.207	-8.027
H	35.894	-15.571	-9.581
H	33.055	-13.833	-7.284
H	34.247	-13.069	-8.100
H	32.900	-13.556	-8.887
N	32.101	-17.161	-7.929
C	30.918	-17.516	-7.165
C	30.050	-18.532	-7.908
O	30.461	-19.116	-8.924
H	33.046	-17.557	-7.749
H	31.193	-17.907	-6.275
H	30.370	-16.687	-6.982
N	28.830	-18.694	-7.388
C	27.864	-19.659	-7.899
C	27.035	-18.959	-8.971
O	25.923	-18.487	-8.722
C	27.036	-20.262	-6.755
C	25.935	-21.180	-7.243
H	28.628	-18.062	-6.575
H	28.376	-20.381	-8.352
H	27.650	-20.783	-6.167
H	26.625	-19.513	-6.240
N	25.052	-21.566	-6.334
O	25.832	-21.461	-8.438
H	25.129	-21.249	-5.388
H	24.301	-22.178	-6.591
N	27.628	-18.918	-10.166
C	27.136	-18.174	-11.305
C	27.675	-18.819	-12.582
O	28.873	-19.088	-12.644
C	27.613	-16.743	-11.192
H	28.517	-19.496	-10.205
H	26.138	-18.220	-11.325
H	27.373	-16.376	-10.292
H	28.607	-16.707	-11.307
H	27.180	-16.184	-11.901
N	26.797	-19.007	-13.581
C	27.169	-19.476	-14.914
C	26.430	-18.679	-15.983
O	25.225	-18.484	-15.887
C	26.850	-20.963	-15.088
C	26.827	-21.409	-16.538
C	26.762	-22.915	-16.756
O	26.878	-23.337	-17.957
O	26.598	-23.660	-15.732
H	25.805	-18.783	-13.314
H	28.160	-19.373	-15.044
H	27.551	-21.474	-14.628
H	25.959	-21.123	-14.710
H	26.026	-21.015	-16.972
H	27.656	-21.085	-16.978
N	27.180	-18.230	-17.003
C	26.610	-17.514	-18.124
C	26.109	-16.134	-17.640
O	26.625	-15.575	-16.656
C	25.524	-18.376	-18.820
O	25.344	-17.984	-20.175
H	28.196	-18.445	-16.908
H	27.326	-17.348	-18.799
H	25.812	-19.326	-18.790
H	24.669	-18.256	-18.329
H	25.517	-17.005	-20.262
N	25.134	-15.590	-18.382
C	24.644	-14.245	-18.201

C	23.375	-14.284	-17.365
O	22.534	-15.180	-17.531
C	24.381	-13.612	-19.571
C	25.644	-13.383	-20.386
C	25.328	-12.901	-21.783
N	25.610	-13.876	-22.829
C	25.057	-15.081	-22.941
N	24.377	-15.612	-21.941
N	25.227	-15.777	-24.050
H	24.757	-16.246	-19.116
H	25.332	-13.704	-17.712
H	23.787	-14.219	-20.085
H	23.942	-12.733	-19.428
H	26.208	-12.694	-19.935
H	26.151	-14.240	-20.457
H	24.352	-12.658	-21.816
H	25.869	-12.069	-21.955
H	26.292	-13.614	-23.539
H	24.247	-15.110	-21.086
H	23.982	-16.532	-22.036
H	25.754	-15.391	-24.812
H	24.831	-16.693	-24.138
N	23.248	-13.255	-16.527
C	22.077	-12.977	-15.717
C	21.493	-11.644	-16.128
O	22.248	-10.720	-16.455
C	22.479	-12.917	-14.235
C	23.038	-14.217	-13.755
C	24.339	-14.607	-14.015
C	22.219	-15.108	-13.093
C	24.794	-15.852	-13.637
C	22.688	-16.323	-12.652
C	23.970	-16.717	-12.948
O	24.366	-17.957	-12.509
H	24.111	-12.634	-16.504
H	21.393	-13.689	-15.892
H	23.168	-12.213	-14.134
H	21.669	-12.702	-13.708
H	24.961	-13.976	-14.486
H	21.258	-14.863	-12.929
H	25.737	-16.133	-13.864
H	22.090	-16.927	-12.111
H	24.585	-17.907	-11.536
N	20.163	-11.533	-16.032
C	19.465	-10.305	-16.291
C	19.878	-9.277	-15.232
O	19.935	-9.615	-14.043
C	17.941	-10.508	-16.331
C	17.213	-9.172	-16.354
C	17.535	-11.352	-17.545
H	19.687	-12.425	-15.751
H	19.749	-9.950	-17.181
H	17.674	-10.971	-15.489
H	16.235	-9.327	-16.492
H	17.356	-8.699	-15.485
H	17.569	-8.610	-17.101
H	16.539	-11.368	-17.611
H	17.925	-10.944	-18.368
H	17.883	-12.280	-17.426
N	20.160	-8.040	-15.674
C	20.260	-6.928	-14.726
C	19.344	-5.801	-15.138
O	18.997	-5.611	-16.316
C	21.699	-6.442	-14.596
C	22.295	-5.622	-15.734
C	21.967	-4.116	-15.652
C	23.787	-5.851	-15.702
H	20.292	-7.953	-16.685
H	19.980	-7.260	-13.820
H	21.757	-5.879	-13.762
H	22.285	-7.251	-14.473
H	21.950	-5.978	-16.616
H	22.328	-3.752	-14.797
H	22.389	-3.650	-16.426
H	20.978	-3.998	-15.682
H	23.985	-6.826	-15.828
H	24.227	-5.329	-16.436
H	24.159	-5.553	-14.820
N	18.911	-5.073	-14.122
C	18.178	-3.855	-14.346
C	18.640	-2.841	-13.315
O	18.917	-3.206	-12.160
C	16.681	-4.134	-14.265
C	16.238	-4.559	-12.897
O	15.972	-2.957	-14.634
H	19.142	-5.450	-13.184
H	18.397	-3.502	-15.253
H	16.444	-4.872	-14.929
H	16.454	-3.846	-12.221
H	15.245	-4.721	-12.884
H	16.704	-5.409	-12.625
H	16.551	-2.357	-15.199
N	18.631	-1.579	-13.714
C	18.916	-0.498	-12.779
C	18.511	0.862	-13.341
O	17.685	0.939	-14.247
H	18.412	-1.431	-14.713
H	18.418	-0.660	-11.915
H	19.904	-0.488	-12.567
N	19.009	1.918	-12.692
C	18.669	3.300	-12.970
C	19.946	4.136	-12.905
O	20.884	3.831	-12.155
C	17.675	3.863	-11.945
C	16.368	3.106	-11.743
C	15.466	3.213	-12.973
N	14.987	4.585	-13.132
C	14.473	5.063	-14.271
N	14.239	4.268	-15.290
N	14.174	6.334	-14.366
H	19.698	1.646	-11.930
H	18.292	3.368	-13.891
H	18.145	3.910	-11.052
H	17.444	4.806	-12.223
H	16.562	2.143	-11.586
H	15.877	3.491	-10.968
H	15.997	2.954	-13.765
H	14.696	2.607	-12.840
H	15.046	5.203	-12.347
H	14.420	3.285	-15.232
H	13.869	4.645	-16.150
H	14.309	6.950	-13.582
H	13.805	6.709	-15.222
N	19.968	5.238	-13.661
C	21.056	6.203	-13.592
C	20.458	7.598	-13.704
O	19.307	7.749	-14.126
C	22.128	5.951	-14.657
C	21.741	6.388	-16.064
C	20.822	5.671	-16.825
C	22.263	7.552	-16.626
C	20.434	6.097	-18.099
C	21.920	7.969	-17.908
C	20.990	7.252	-18.645
O	20.610	7.706	-19.918
H	19.148	5.341	-14.294
H	21.496	6.122	-12.701
H	22.953	6.449	-14.396
H	22.321	4.971	-14.683
H	20.425	4.826	-16.456
H	22.905	8.107	-16.089
H	19.757	5.573	-18.617
H	22.346	8.787	-18.299
H	19.772	7.241	-20.180
N	21.251	8.591	-13.287
C	20.876	10.003	-13.337
C	21.041	10.494	-14.773
O	22.164	10.619	-15.240
C	21.762	10.847	-12.427
C	21.431	12.343	-12.438
O	20.553	12.757	-13.218
O	22.057	13.069	-11.688
H	22.178	8.263	-12.916
H	19.905	10.061	-13.117
H	21.659	10.512	-11.492
H	22.710	10.735	-12.721
N	19.929	10.718	-15.485
C	20.020	11.036	-16.904
C	20.250	12.534	-17.136
O	20.380	12.945	-18.280
C	18.816	10.521	-17.667
O	17.594	10.808	-16.983
H	19.050	10.647	-14.960
H	20.812	10.543	-17.288
H	18.784	10.955	-18.565
H	18.892	9.531	-17.774
H	16.966	10.042	-17.100
N	20.328	13.350	-16.081
C	20.666	14.773	-16.210
C	21.651	15.160	-15.109
O	21.278	15.787	-14.109
C	19.413	15.622	-16.140
H	20.130	12.894	-15.166
H	21.102	14.920	-17.097
H	18.654	15.075	-15.785
H	19.568	16.403	-15.534
H	19.180	15.956	-17.054
N	22.914	14.691	-15.192
C	23.869	14.905	-14.102
C	24.288	16.360	-13.926
O	23.865	17.231	-14.684
C	25.054	14.014	-14.485
C	24.943	13.815	-15.971
C	23.463	13.874	-16.288
H	23.498	14.533	-13.228
H	25.925	14.460	-14.251
H	25.007	13.133	-14.004
H	25.440	14.538	-16.460
H	25.328	12.925	-16.235
H	23.300	14.312	-17.171
H	23.056	12.961	-16.286
N	25.076	16.622	-12.885
C	25.649	17.945	-12.684
C	26.808	18.166	-13.666
O	27.356	17.222	-14.234
C	26.069	18.115	-11.242
H	25.240	15.821	-12.249
H	24.947	18.638	-12.864
H	26.844	17.513	-11.041
H	26.340	19.065	-11.077
H	25.306	17.882	-10.637
N	27.169	19.441	-13.880
C	28.145	19.810	-14.902
C	29.384	20.444	-14.275
O	30.107	21.184	-14.937
C	27.518	20.753	-15.938
C	26.843	19.985	-17.058
O	26.609	21.617	-15.239
H	26.697	20.130	-13.261
H	28.429	18.975	-15.389
H	28.246	21.325	-16.348
H	27.508	19.387	-17.512
H	26.098	19.423	-16.688
H	26.465	20.624	-17.733
H	25.784	21.110	-14.971
N	29.633	20.121	-13.004
C	30.730	20.688	-12.243
C	31.884	19.689	-12.188
O	32.733	19.808	-11.305
C	30.266	21.066	-10.834
C	29.713	19.913	-10.004
O	29.595	18.782	-10.530
O	29.421	20.149	-8.824
H	28.962	19.419	-12.607
H	31.063	21.490	-12.744
H	31.049	21.459	-10.347
H	29.552	21.764	-10.921
N	31.878	18.700	-13.102
C	32.855	17.624	-13.121
C	32.514	16.447	-12.191
O	33.326	15.545	-12.026
H	31.099	18.782	-13.800
H	32.951	17.269	-14.072
H	33.769	17.990	-12.858
N	31.315	16.447	-11.603

C	30.853	15.344	-10.767
C	30.583	14.111	-11.617
O	30.119	14.202	-12.757
C	29.614	15.711	-10.014
O	29.908	16.619	-8.968
H	30.744	17.299	-11.793
H	31.584	15.118	-10.116
H	28.960	16.142	-10.633
H	29.208	14.890	-9.615
H	29.862	17.555	-9.316
N	30.733	12.931	-10.996
C	30.303	11.697	-11.622
C	28.775	11.580	-11.734
O	27.999	12.375	-11.191
H	31.170	12.989	-10.061
H	30.702	11.628	-12.553
H	30.651	10.904	-11.091
N	28.346	10.529	-12.450
C	26.941	10.270	-12.707
C	26.508	9.086	-11.842
O	26.966	7.981	-12.093
C	26.735	9.911	-14.181
C	25.311	9.542	-14.525
O	27.200	11.080	-14.876
H	29.118	9.917	-12.803
H	26.409	11.067	-12.433
H	27.329	9.146	-14.420
H	24.674	10.008	-13.904
H	25.102	9.815	-15.469
H	25.181	8.550	-14.439
H	26.767	11.142	-15.781
N	25.635	9.335	-10.874
C	25.159	8.277	-9.977
C	24.361	7.233	-10.743
O	23.536	7.568	-11.579
C	24.332	8.891	-8.891
H	25.323	10.321	-10.806
H	25.957	7.830	-9.556
H	23.791	9.654	-9.256
H	23.706	8.208	-8.504
H	24.924	9.238	-8.158
N	24.529	5.965	-10.345
C	23.777	4.873	-10.904
C	23.784	3.683	-9.952
O	24.492	3.675	-8.951
C	24.360	4.528	-12.277
C	25.873	4.341	-12.368
C	26.344	3.064	-11.725
C	26.301	4.358	-13.835
H	25.252	5.851	-9.597
H	22.822	5.162	-11.036
H	23.935	3.674	-12.582
H	24.112	5.264	-12.907
H	26.322	5.130	-11.922
H	25.915	2.276	-12.174
H	27.340	2.985	-11.809
H	26.098	3.059	-10.753
H	26.047	5.234	-14.244
H	27.291	4.235	-13.893
H	25.843	3.615	-14.323
N	22.977	2.685	-10.294
C	22.977	1.423	-9.596
C	22.205	0.392	-10.388
O	21.419	0.750	-11.268
H	22.352	2.898	-11.104
H	23.923	1.105	-9.467
H	22.553	1.534	-8.690
N	22.433	-0.869	-10.053
C	21.730	-1.951	-10.709
C	21.719	-3.167	-9.791
O	22.469	-3.219	-8.809
C	22.343	-2.313	-12.080
C	23.714	-2.920	-12.022
C	24.018	-4.247	-12.070
C	24.978	-2.235	-12.084
C	25.985	-3.209	-12.067
C	25.361	-0.892	-12.115
N	25.371	-4.419	-12.035
C	27.358	-2.902	-12.089
C	26.704	-0.581	-12.157
C	27.687	-1.570	-12.111
H	23.136	-1.005	-9.305
H	20.780	-1.669	-10.845
H	21.744	-2.971	-12.530
H	22.408	-1.482	-12.627
H	23.347	-4.987	-12.123
H	25.843	-5.309	-11.992
H	24.675	-0.165	-12.108
H	28.038	-3.628	-12.087
H	26.976	0.381	-12.221
H	28.649	-1.296	-12.094
N	20.852	-4.117	-10.160
C	20.633	-5.358	-9.443
C	20.725	-6.525	-10.413
O	20.188	-6.425	-11.511
C	19.235	-5.444	-8.839
C	19.006	-6.730	-8.060
O	19.059	-4.299	-7.992
H	20.326	-3.873	-11.048
H	21.352	-5.464	-8.753
H	18.546	-5.395	-9.581
H	18.672	-6.515	-7.139
H	18.327	-7.297	-8.531
H	19.866	-7.241	-7.986
H	18.204	-4.380	-7.469
N	21.365	-7.588	-9.930
C	21.297	-8.935	-10.479
C	20.733	-9.877	-9.422
O	21.255	-9.923	-8.302
C	22.681	-9.411	-10.954
C	22.594	-10.879	-11.359
C	23.231	-8.511	-12.059
H	21.955	-7.361	-9.073
H	20.666	-8.928	-11.255
H	23.300	-9.360	-10.184
H	23.150	-11.429	-10.737
H	21.642	-11.181	-11.312
H	22.933	-10.987	-12.293
H	23.727	-9.071	-12.721
H	22.471	-8.046	-12.511
H	23.849	-7.838	-11.655
N	19.665	-10.626	-9.771
C	19.296	-11.830	-9.021
C	19.964	-13.036	-9.694
O	19.892	-13.158	-10.908
C	17.807	-12.009	-8.961
H	19.147	-10.278	-10.595
H	19.643	-11.752	-8.092
H	17.349	-11.123	-9.069
H	17.504	-12.622	-9.695
H	17.543	-12.405	-8.078
N	20.624	-13.882	-8.904
C	21.550	-14.891	-9.408
C	20.799	-16.170	-9.769
O	21.185	-17.257	-9.298
C	22.661	-15.140	-8.389
C	23.513	-13.921	-8.227
C	23.515	-13.059	-7.173
C	24.412	-13.382	-9.207
C	24.971	-12.211	-8.645
C	24.857	-13.798	-10.468
N	24.405	-12.052	-7.397
C	25.949	-11.459	-9.315
C	25.816	-13.053	-11.125
C	26.348	-11.898	-10.558
H	20.419	-13.759	-7.882
H	21.963	-14.535	-10.251
H	22.258	-15.339	-7.517
H	23.238	-15.865	-8.713
H	22.946	-13.152	-6.355
H	24.623	-11.307	-6.763
H	24.486	-14.627	-10.890
H	26.331	-10.636	-8.891
H	26.130	-13.353	-12.025
H	27.035	-11.378	-11.067
N	19.735	-15.978	-10.550
C	18.969	-17.063	-11.171
C	18.969	-16.837	-12.680
O	18.667	-15.761	-13.180
C	17.524	-17.142	-10.636
C	16.621	-18.159	-11.346
C	15.149	-18.050	-10.978
C	14.258	-19.029	-11.718
N	14.178	-18.688	-13.156
H	19.486	-14.964	-10.686
H	19.417	-17.929	-10.974
H	17.566	-17.387	-9.667
H	17.109	-16.236	-10.732
H	16.718	-18.031	-12.344
H	16.946	-19.089	-11.121
H	15.052	-18.214	-9.991
H	14.835	-17.117	-11.181
H	14.633	-19.951	-11.615
H	13.340	-19.000	-11.318
H	14.219	-17.694	-13.268
H	14.944	-19.109	-13.644
H	13.316	-19.029	-13.534
N	19.236	-17.933	-13.407
C	19.104	-17.953	-14.839
C	18.774	-19.400	-15.236
O	18.320	-20.183	-14.406
C	20.371	-17.402	-15.503
C	21.594	-18.289	-15.379
H	19.543	-18.752	-12.837
H	18.312	-17.407	-15.084
H	20.177	-17.262	-16.480
H	20.580	-16.511	-15.086
N	22.727	-17.812	-15.893
O	21.567	-19.362	-14.761
H	22.734	-16.909	-16.325
H	23.564	-18.353	-15.846
N	19.077	-19.753	-16.487
C	18.681	-21.054	-17.011
C	19.740	-22.110	-16.774
O	19.623	-23.213	-17.317
C	18.321	-20.963	-18.490
C	17.052	-20.162	-18.693
H	19.598	-19.046	-17.028
H	17.855	-21.340	-16.502
H	19.061	-20.512	-18.969
H	18.178	-21.880	-18.837
N	16.847	-19.688	-19.910
O	16.257	-19.985	-17.764
H	17.508	-19.869	-20.641
H	16.028	-19.145	-20.108
N	20.720	-21.795	-15.928
C	21.761	-22.711	-15.554
C	21.795	-22.967	-14.059
O	22.094	-24.096	-13.656
C	23.096	-22.174	-16.054
C	23.166	-22.200	-17.551
C	23.431	-23.379	-18.237
C	22.853	-21.070	-18.287
C	23.468	-23.406	-19.624
C	22.890	-21.074	-19.665
C	23.215	-22.241	-20.329
O	23.183	-22.180	-21.680
H	20.656	-20.810	-15.561
H	21.594	-23.599	-16.018
H	23.191	-21.239	-15.748
H	23.817	-22.746	-15.694
H	23.599	-24.223	-17.725
H	22.592	-20.229	-17.805
H	23.673	-24.255	-20.108
H	22.685	-20.243	-20.181
H	24.081	-21.904	-22.029
N	21.571	-21.913	-13.252
C	21.880	-21.956	-11.833
C	20.838	-21.119	-11.102
O	20.224	-20.211	-11.659
C	23.218	-21.298	-11.462
C	24.438	-21.692	-12.265
C	25.246	-22.806	-11.649
N	26.440	-22.952	-12.468
C	27.678	-22.691	-12.061

N	28.668	-22.707	-12.940
N	27.911	-22.392	-10.790
H	21.166	-21.087	-13.733
H	21.817	-22.896	-11.512
H	23.099	-20.297	-11.543
H	23.402	-21.507	-10.490
H	24.149	-21.986	-13.187
H	25.041	-20.889	-12.370
H	25.499	-22.545	-10.743
H	24.720	-23.629	-11.684
H	26.321	-23.276	-13.420
H	28.512	-22.889	-13.905
H	29.614	-22.527	-12.624
H	27.144	-22.345	-10.144
H	28.840	-22.213	-10.476
N	20.654	-21.432	-9.839
C	19.986	-20.493	-8.943
C	20.738	-20.491	-7.625
O	20.598	-21.431	-6.844
C	18.489	-20.786	-8.796
C	17.744	-19.733	-8.009
H	21.004	-22.354	-9.545
H	20.084	-19.585	-9.341
H	18.091	-20.844	-9.712
H	18.388	-21.666	-8.331
N	16.428	-19.893	-7.859
O	18.379	-18.777	-7.562
H	15.990	-20.701	-8.265
H	15.894	-19.227	-7.356
N	21.443	-19.379	-7.320
C	22.180	-19.296	-6.078
C	21.363	-18.663	-4.953
O	21.930	-18.429	-3.886
C	23.463	-18.527	-6.288
H	21.400	-18.636	-8.031
H	22.446	-20.232	-5.788
H	24.173	-19.140	-6.639
H	23.312	-17.788	-6.947
H	23.770	-18.134	-5.419
N	20.080	-18.352	-5.188
C	19.192	-17.842	-4.156
C	19.783	-16.590	-3.493
O	19.830	-16.453	-2.261
C	18.886	-18.915	-3.105
C	18.259	-20.133	-3.713
H	19.781	-18.510	-6.177
H	18.317	-17.583	-4.580
H	19.736	-19.186	-2.658
H	18.255	-18.538	-2.429
C	18.674	-21.426	-3.715
N	17.123	-20.082	-4.496
C	16.842	-21.329	-4.920
N	17.772	-22.157	-4.463
H	16.599	-19.273	-4.715
H	19.484	-21.787	-3.258
H	16.062	-21.578	-5.482
N	20.229	-15.673	-4.328
C	20.927	-14.503	-3.833
C	20.751	-13.388	-4.866
O	20.322	-13.645	-6.002
C	22.377	-14.831	-3.546
O	23.010	-15.279	-4.730
H	20.044	-15.854	-5.329
H	20.492	-14.206	-2.990
H	22.839	-14.015	-3.222
H	22.420	-15.552	-2.866
H	22.640	-16.170	-4.990
N	21.039	-12.151	-4.444
C	20.962	-10.991	-5.327
C	22.060	-10.005	-4.912
O	22.354	-9.856	-3.729
C	19.585	-10.358	-5.277
H	21.319	-12.093	-3.444
H	21.135	-11.281	-6.263
H	18.899	-11.059	-5.079
H	19.561	-9.660	-4.560
H	19.377	-9.933	-6.159
N	22.669	-9.335	-5.888
C	23.660	-8.297	-5.649
C	23.140	-6.968	-6.192
O	22.523	-6.904	-7.262
C	24.988	-8.623	-6.350
C	26.046	-7.556	-6.157
O	25.461	-9.852	-5.814
H	22.369	-9.624	-6.853
H	23.795	-8.201	-4.662
H	24.812	-8.748	-7.340
H	26.945	-7.983	-6.026
H	26.084	-6.960	-6.964
H	25.829	-6.999	-5.350
H	25.210	-9.936	-4.842
N	23.342	-5.891	-5.427
C	23.111	-4.550	-5.935
C	24.447	-3.816	-5.920
O	25.223	-3.939	-4.977
C	22.082	-3.760	-5.116
C	20.728	-4.401	-5.122
O	22.638	-3.600	-3.802
H	23.669	-6.098	-4.466
H	22.797	-4.611	-6.877
H	21.967	-2.844	-5.525
H	20.810	-5.395	-5.258
H	20.257	-4.237	-4.248
H	20.163	-4.023	-5.864
H	23.083	-4.451	-3.506
N	24.719	-3.078	-7.005
C	25.859	-2.202	-7.130
C	25.362	-0.762	-7.156
O	24.432	-0.437	-7.898
C	26.615	-2.458	-8.453
C	27.324	-3.759	-8.626
C	28.669	-3.941	-8.531
C	26.784	-5.052	-8.953
C	27.882	-5.935	-9.080
C	25.501	-5.543	-9.196
N	29.005	-5.234	-8.812
C	27.748	-7.287	-9.415
C	25.372	-6.870	-9.581
C	26.470	-7.719	-9.672
H	24.004	-3.202	-7.776
H	26.427	-2.307	-6.315
H	25.952	-2.375	-9.214
H	27.303	-1.724	-8.567
H	29.327	-3.221	-8.287
H	29.941	-5.602	-8.817
H	24.699	-4.967	-9.098
H	28.540	-7.883	-9.460
H	24.457	-7.217	-9.798
H	26.319	-8.679	-9.934
N	26.001	0.063	-6.333
C	25.725	1.472	-6.234
C	27.028	2.203	-6.541
O	28.082	1.949	-5.938
C	25.176	1.755	-4.855
O	24.908	3.134	-4.692
H	26.741	-0.419	-5.750
H	25.067	1.721	-6.935
H	24.328	1.246	-4.728
H	25.846	1.475	-4.171
H	24.218	3.259	-3.979
N	26.966	3.169	-7.465
C	28.191	3.821	-7.822
C	28.029	5.053	-8.725
O	26.998	5.711	-8.717
H	26.048	3.366	-7.844
H	28.692	4.109	-6.975
H	28.814	3.158	-8.295
N	29.154	5.378	-9.369
C	29.165	6.507	-10.287
C	30.032	6.195	-11.500
O	31.086	5.578	-11.406
C	29.501	7.807	-9.551
C	30.851	7.916	-8.881
C	30.983	9.235	-8.148
H	29.966	4.786	-9.164
H	28.226	6.655	-10.611
H	29.417	8.578	-10.215
H	28.784	7.963	-8.840
H	30.948	7.177	-8.224
H	31.562	7.864	-9.573
N	31.529	9.174	-6.943
O	30.615	10.299	-8.661
H	31.666	10.023	-6.389
H	31.812	8.271	-6.571
N	29.503	6.687	-12.630
C	30.196	6.734	-13.902
C	31.020	8.014	-13.955
O	30.476	9.078	-13.710
C	29.151	6.709	-15.006
C	29.695	7.095	-16.357
C	30.380	6.186	-17.130
C	29.559	8.395	-16.810
C	30.908	6.544	-18.366
C	30.043	8.760	-18.057
C	30.720	7.833	-18.831
O	31.194	8.214	-20.088
H	28.516	7.043	-12.503
H	30.823	5.959	-13.955
H	28.782	5.785	-15.070
H	28.425	7.350	-14.768
H	30.507	5.244	-16.804
H	29.110	9.076	-16.239
H	31.414	5.877	-18.909
H	29.904	9.691	-18.397
H	30.731	7.670	-20.780
N	32.305	7.867	-14.293
C	33.238	8.957	-14.511
C	33.751	8.887	-15.954
O	34.421	7.915	-16.305
C	34.408	8.848	-13.522
C	35.349	10.041	-13.674
C	33.919	8.669	-12.081
H	32.591	6.849	-14.391
H	32.750	9.821	-14.404
H	34.932	8.036	-13.757
H	34.811	10.874	-13.799
H	35.911	10.126	-12.853
H	35.937	9.900	-14.469
H	32.977	8.991	-12.015
H	33.965	7.702	-11.840
H	34.504	9.200	-11.471
N	33.439	9.909	-16.762
C	33.756	9.923	-18.188
C	35.221	10.269	-18.476
O	36.038	10.340	-17.570
H	32.948	10.697	-16.279
H	33.548	9.013	-18.587
H	33.160	10.597	-18.658
N	35.546	10.458	-19.763
C	36.856	10.936	-20.174
C	37.710	9.821	-20.770
O	37.198	8.754	-21.115
H	34.779	10.232	-20.433
H	36.748	11.672	-20.860
H	37.337	11.330	-19.377
N	39.020	10.095	-20.848
C	39.983	9.257	-21.551
C	39.798	7.791	-21.170
O	39.489	6.959	-22.021
C	41.384	9.727	-21.221
H	39.293	10.981	-20.350
H	39.835	9.351	-22.542
H	41.987	8.937	-21.105
H	41.732	10.301	-21.964
H	41.371	10.258	-20.373
N	39.988	7.527	-19.873
C	39.884	6.213	-19.263
C	38.594	6.161	-18.424
O	38.645	6.296	-17.207
C	41.102	5.969	-18.359
C	42.457	6.117	-19.039
C	42.853	4.955	-19.932
O	41.957	4.232	-20.391
O	44.062	4.758	-20.135
H	40.230	8.395	-19.309

H	39.845	5.507	-19.966
H	41.063	6.629	-17.624
H	41.039	5.044	-18.017
H	42.430	6.931	-19.604
H	43.149	6.199	-18.334
N	37.437	5.980	-19.075
C	36.166	6.019	-18.365
C	36.107	4.860	-17.365
O	36.675	3.787	-17.574
C	35.003	5.996	-19.325
H	37.530	5.817	-20.089
H	36.100	6.879	-17.857
H	34.143	6.104	-18.822
H	35.091	6.744	-19.986
H	34.985	5.124	-19.819
N	35.382	5.084	-16.267
C	35.340	4.102	-15.197
C	33.955	4.164	-14.567
O	33.317	5.211	-14.566
C	36.452	4.362	-14.168
C	37.696	3.519	-14.372
C	38.887	3.944	-13.537
N	39.996	3.009	-13.657
C	40.712	2.802	-14.755
N	41.697	1.922	-14.731
N	40.444	3.459	-15.874
H	34.876	5.982	-16.251
H	35.477	3.194	-15.580
H	36.711	5.322	-14.231
H	36.086	4.165	-13.263
H	37.486	2.556	-14.148
H	37.969	3.558	-15.344
H	39.179	4.845	-13.849
H	38.596	3.991	-12.584
H	40.244	2.469	-12.832
H	41.911	1.390	-13.918
H	42.256	1.780	-15.565
H	39.674	4.107	-15.887
H	40.993	3.316	-16.690
N	33.497	3.010	-14.046
C	32.350	2.975	-13.142
C	32.862	2.390	-11.820
O	33.340	1.270	-11.773
C	31.160	2.177	-13.716
C	30.639	2.775	-15.032
C	30.050	2.059	-12.669
C	29.603	1.933	-15.748
H	34.024	2.168	-14.341
H	32.109	3.915	-12.957
H	31.477	1.247	-13.906
H	30.246	3.675	-14.833
H	31.423	2.908	-15.643
H	29.328	1.468	-13.020
H	30.427	1.668	-11.832
H	29.679	2.965	-12.479
H	28.810	1.809	-15.154
H	29.323	2.396	-16.587
H	29.998	1.043	-15.971
N	32.821	3.213	-10.771
C	33.350	2.857	-9.478
C	32.159	2.557	-8.568
O	31.249	3.376	-8.461
C	34.149	4.007	-8.868
C	35.391	4.364	-9.642
H	32.370	4.137	-10.977
H	33.855	2.006	-9.578
H	33.554	4.813	-8.824
H	34.414	3.744	-7.937
N	35.549	5.654	-9.892
O	36.140	3.479	-10.058
H	34.885	6.327	-9.576
H	36.355	5.967	-10.411
N	32.155	1.379	-7.960
C	30.953	0.907	-7.284
C	31.310	0.274	-5.939
O	32.436	-0.156	-5.712
C	30.148	-0.154	-8.057
C	29.839	0.223	-9.494
O	30.847	-1.396	-8.056
H	33.037	0.851	-8.005
H	30.351	1.693	-7.111
H	29.250	-0.290	-7.587
H	29.744	-0.605	-10.052
H	28.983	0.744	-9.534
H	30.581	0.785	-9.867
H	31.809	-1.260	-8.319
N	30.275	0.239	-5.095
C	30.260	-0.663	-3.963
C	29.056	-1.540	-4.148
O	28.089	-1.162	-4.827
C	30.197	0.172	-2.681
C	31.499	0.959	-2.508
C	31.701	1.636	-1.180
H	29.509	0.899	-5.323
H	31.071	-1.232	-4.009
H	29.457	0.815	-2.751
H	30.097	-0.429	-1.909
H	32.273	0.320	-2.669
H	31.538	1.661	-3.242
N	31.528	2.934	-1.139
O	32.008	0.995	-0.183
H	31.633	3.464	-0.271
H	31.281	3.433	-2.000
N	29.113	-2.729	-3.566
C	28.038	-3.648	-3.782
C	27.623	-4.351	-2.480
O	28.439	-4.448	-1.562
C	28.361	-4.650	-4.887
C	29.605	-5.439	-4.777
C	30.719	-5.263	-5.535
C	29.835	-6.593	-3.958
C	31.138	-7.032	-4.250
C	29.073	-7.310	-3.029
N	31.671	-6.141	-5.147
C	31.740	-8.101	-3.588
C	29.666	-8.365	-2.365
C	30.966	-8.772	-2.655
H	29.941	-2.906	-2.992
H	27.231	-3.131	-4.104
H	27.591	-5.309	-4.953
H	28.399	-4.148	-5.769
H	30.814	-4.587	-6.266
H	32.629	-6.152	-5.460
H	28.124	-7.067	-2.848
H	32.681	-8.363	-3.783
H	29.144	-8.846	-1.659
H	31.349	-9.563	-2.181
N	26.348	-4.780	-2.475
C	25.810	-5.608	-1.409
C	25.246	-6.886	-2.018
O	24.378	-6.817	-2.899
C	24.692	-4.845	-0.693
C	25.070	-3.580	0.092
C	23.819	-2.834	0.592
C	25.913	-3.901	1.290
H	25.792	-4.465	-3.298
H	26.550	-5.855	-0.786
H	24.017	-4.571	-1.384
H	24.258	-5.475	-0.045
H	25.595	-2.971	-0.503
H	23.297	-3.438	1.190
H	24.104	-2.021	1.095
H	23.263	-2.570	-0.192
H	26.763	-4.354	1.004
H	26.146	-3.058	1.785
H	25.413	-4.513	1.910
N	25.625	-8.030	-1.455
C	25.185	-9.331	-1.956
C	24.430	-10.007	-0.820
O	25.045	-10.262	0.218
C	26.403	-10.156	-2.372
C	26.139	-11.517	-3.039
C	27.459	-12.158	-3.480
C	25.385	-12.430	-2.119
H	26.257	-7.920	-0.633
H	24.561	-9.188	-2.711
H	26.937	-9.612	-3.020
H	26.950	-10.332	-1.554
H	25.557	-11.379	-3.836
H	28.043	-12.292	-2.681
H	27.270	-13.039	-3.910
H	27.915	-11.556	-4.133
H	24.501	-12.021	-1.874
H	25.220	-13.314	-2.567
H	25.913	-12.587	-1.279
N	23.110	-10.112	-0.986
C	22.283	-10.796	0.006
C	21.951	-12.220	-0.447
O	21.469	-12.452	-1.570
C	20.971	-10.048	0.275
C	20.141	-10.673	1.389
O	21.310	-8.714	0.659
H	22.740	-9.682	-1.847
H	22.798	-10.853	0.868
H	20.434	-10.007	-0.583
H	20.728	-11.239	1.973
H	19.721	-9.952	1.945
H	19.420	-11.246	0.994
H	21.377	-8.121	-0.151
N	22.068	-13.148	0.501
C	21.696	-14.539	0.282
C	20.450	-14.825	1.111
O	20.333	-14.336	2.237
C	22.844	-15.513	0.623
C	24.128	-15.225	-0.159
C	22.394	-16.952	0.409
C	25.231	-14.641	0.693
H	22.447	-12.793	1.407
H	21.426	-14.612	-0.672
H	23.049	-15.414	1.601
H	24.447	-16.077	-0.566
H	23.910	-14.581	-0.889
H	23.109	-17.575	0.722
H	21.557	-17.120	0.926
H	22.221	-17.106	-0.562
H	25.461	-15.283	1.424
H	26.037	-14.477	0.126
H	24.922	-13.778	1.091
N	19.472	-15.521	0.509
C	18.291	-15.959	1.234
C	18.692	-16.829	2.431
O	19.567	-17.672	2.293
H	19.638	-15.710	-0.497
H	17.780	-15.162	1.566
H	17.697	-16.493	0.626
N	18.194	-16.491	3.627
C	18.563	-17.221	4.840
C	17.303	-17.412	5.684
O	16.299	-16.739	5.492
C	19.641	-16.525	5.710
C	20.939	-16.226	4.988
O	19.106	-15.324	6.270
H	17.542	-15.688	3.615
H	18.907	-18.118	4.580
H	19.894	-17.152	6.474
H	20.832	-16.396	4.005
H	21.196	-15.266	5.127
H	21.672	-16.813	5.342
H	18.597	-15.526	7.114
N	17.380	-18.360	6.631
C	16.488	-18.401	7.781
C	16.727	-17.176	8.664
O	17.765	-16.497	8.581
C	16.728	-19.684	8.598
C	16.668	-20.937	7.747
O	18.031	-19.555	9.160
H	18.137	-19.062	6.466
H	15.541	-18.373	7.456
H	16.056	-19.722	9.348
H	17.058	-21.713	8.251
H	15.715	-21.147	7.512
H	17.190	-20.802	6.901
H	18.444	-18.675	8.897
N	15.772	-16.927	9.565
C	15.818	-15.751	10.425

C	17.016	-15.892	11.357	N	21.949	-12.643	10.194	C	23.162	-12.926	4.977	C	29.801	-5.036	2.043	H	33.285	-4.119	-9.719
O	17.715	-14.903	11.601	C	23.138	-11.850	10.512	C	23.793	-11.964	3.963	C	30.831	-5.248	3.159	N	35.524	-2.013	-9.566
C	14.431	-15.531	11.067	C	24.278	-12.128	9.534	O	23.506	-12.132	2.788	C	29.964	-3.668	1.426	C	35.763	-0.927	-10.500
C	13.364	-15.320	9.978	O	25.176	-11.300	9.361	C	21.689	-12.615	5.118	H	30.264	-7.990	2.068	C	35.706	-1.488	-11.929
C	11.920	-15.019	10.349	C	23.585	-12.061	11.986	O	21.456	-11.271	5.568	H	29.119	-5.911	0.301	O	36.363	-2.488	-12.181
O	11.063	-15.886	10.092	C	24.138	-13.427	12.249	H	23.532	-12.270	7.053	H	28.896	-5.057	2.483	C	37.134	-0.270	-10.274
O	11.633	-13.885	10.795	C	23.439	-14.559	12.577	H	23.244	-13.864	4.600	H	30.354	-5.448	4.013	C	37.313	0.935	-11.165
H	15.011	-17.632	9.595	C	25.499	-13.844	12.067	H	21.237	-12.729	4.233	H	31.424	-6.014	2.917	O	37.238	0.176	-8.925
H	15.919	-14.929	9.872	C	25.554	-15.226	12.384	H	21.278	-13.240	5.783	H	31.377	-4.419	3.264	H	36.152	-2.820	-9.438
H	14.199	-16.333	11.553	C	26.690	-13.173	11.786	H	21.502	-11.250	6.563	H	29.697	-2.966	2.089	H	35.037	-0.250	-10.405
H	14.473	-14.724	11.596	N	24.288	-15.630	12.682	N	24.596	-10.975	4.373	H	30.921	-3.530	1.163	H	37.863	-0.967	-10.439
H	13.685	-14.557	9.389	C	26.740	-15.947	12.364	C	24.928	-9.898	3.434	H	29.383	-3.594	0.613	H	36.477	1.099	-11.698
H	13.354	-16.156	9.401	C	27.863	-13.889	11.736	C	26.431	-9.677	3.349	N	31.209	-5.426	-0.847	H	37.508	1.750	-10.610
N	17.326	-17.142	11.756	C	27.889	-15.255	12.043	O	27.068	-9.318	4.337	C	32.466	-5.297	-1.554	H	38.077	0.786	-11.800
C	18.469	-17.440	12.612	H	21.549	-13.356	10.836	C	24.212	-8.575	3.732	C	32.488	-4.091	-2.477	H	37.066	-0.584	-8.287
C	19.815	-17.064	11.968	H	22.890	-10.877	10.420	C	24.382	-7.539	2.617	O	31.651	-3.194	-2.371	N	34.918	-0.840	-12.794
O	20.763	-16.707	12.678	H	24.294	-11.403	12.193	O	22.813	-8.814	3.858	H	30.330	-4.983	-1.131	C	34.666	-1.310	-14.154
C	18.460	-18.913	12.941	H	22.797	-11.936	12.571	H	24.931	-11.035	5.330	H	33.224	-5.217	-0.885	C	35.372	-0.392	-15.147
H	16.673	-17.873	11.39	H	22.450	-14.597	12.719	H	24.591	-10.161	2.520	H	32.636	-6.135	-2.099	O	35.298	0.805	-15.001
H	18.377	-16.917	13.476	H	24.019	-16.561	12.937	H	24.556	-8.207	4.620	N	33.531	-4.028	-3.320	C	33.176	-1.339	-14.449
H	19.298	-19.343	12.600	H	26.693	-12.185	11.622	H	23.566	-7.533	2.038	C	33.654	-2.911	-4.238	C	32.376	-2.317	-13.623
H	18.407	-19.040	13.933	H	26.752	-16.925	12.575	H	24.510	-6.632	3.019	C	34.200	-3.424	-5.569	C	31.826	-1.949	-12.393
H	17.669	-19.353	12.512	H	28.708	-13.428	11.478	H	25.182	-7.772	2.062	O	35.044	-4.304	-5.571	C	32.197	-3.626	-14.057
N	19.924	-17.211	10.641	H	28.766	-15.737	12.027	H	22.643	-9.637	4.412	C	34.551	-1.814	-3.666	C	31.165	-2.908	-11.636
C	21.184	-16.973	9.939	N	24.317	-13.304	8.921	N	26.988	-9.888	2.149	C	35.793	-2.326	-3.044	C	31.518	-4.573	-13.290
C	21.244	-15.589	9.271	C	25.395	-13.610	7.993	C	28.365	-9.507	1.894	C	37.081	-2.377	-3.458	C	30.970	-4.184	-12.094
O	22.177	-15.340	8.528	C	24.852	-13.688	6.559	C	28.412	-8.100	1.311	N	35.788	-2.793	-1.750	H	34.496	0.038	-12.408
C	21.466	-18.069	8.914	O	25.433	-14.308	5.684	O	27.504	-7.712	0.566	C	37.037	-3.145	-1.404	H	35.094	-2.210	-14.237
C	21.752	-19.396	9.586	C	26.159	-14.847	8.457	C	28.973	-10.498	0.892	N	37.840	-2.910	-2.435	H	32.809	-0.430	-14.277
H	19.049	-17.504	10.166	C	25.368	-16.147	8.484	C	29.199	-11.932	1.357	H	34.195	-4.807	-3.252	H	33.055	-1.585	-15.405
H	21.921	-16.993	10.632	C	26.230	-17.352	8.858	C	29.476	-12.838	0.156	H	32.747	-2.525	-4.401	H	31.904	-1.025	-12.067
H	20.668	-18.171	8.334	C	25.395	-18.533	9.311	C	30.363	-11.995	2.318	H	34.799	-1.179	-4.408	H	32.568	-3.901	-14.953
H	22.259	-17.805	8.380	N	26.201	-19.634	9.899	H	26.367	-10.330	1.454	H	34.031	-1.300	-2.973	H	30.820	-2.639	-10.710
N	21.361	-20.493	8.963	H	23.550	-13.951	9.151	H	28.868	-9.506	2.762	H	34.992	-2.863	-1.157	H	31.442	-5.520	-13.621
O	22.283	-19.419	10.689	H	26.082	-12.880	8.032	H	28.363	-10.534	0.095	H	37.428	-2.082	-4.349	H	30.425	-4.833	-11.553
H	20.898	-20.413	8.072	H	26.948	-14.975	7.846	H	29.861	-10.131	0.606	H	37.307	-3.515	-0.525	N	36.065	-0.975	-16.128
H	21.519	-21.391	9.363	H	26.503	-14.671	9.387	H	28.383	-12.257	1.831	N	33.680	-2.909	-6.673	C	36.676	-0.196	-17.193
N	20.270	-14.711	9.516	H	24.625	-16.065	9.155	H	30.292	-12.515	-0.321	C	34.124	-3.299	-8.004	C	36.175	-0.729	-18.536
C	20.126	-13.467	8.757	H	24.969	-16.308	7.577	H	29.622	-13.774	0.475	C	34.430	-2.019	-8.792	O	35.573	-1.790	-18.577
C	21.285	-12.503	9.038	H	26.762	-17.622	8.059	H	28.693	-12.813	-0.464	O	33.648	-1.078	-8.727	C	38.212	-0.227	-17.091
O	21.590	-11.642	8.211	H	26.842	-17.088	9.600	H	30.175	-11.421	3.118	C	33.049	-4.106	-8.733	C	38.731	0.335	-15.788
C	18.790	-12.819	9.055	H	24.724	-18.214	9.997	H	30.505	-12.941	2.619	C	32.814	-5.556	-8.287	O	38.667	-1.572	-17.145
H	19.626	-14.985	10.288	H	24.878	-18.891	8.519	H	31.195	-11.666	1.866	O	33.609	-6.100	-7.493	H	36.125	-2.008	-16.061
H	20.125	-13.686	7.773	H	25.865	-20.512	9.557	N	29.566	-7.451	1.499	O	31.770	-6.119	-8.682	H	36.385	0.755	-17.106
H	18.717	-12.630	10.035	H	27.160	-19.519	9.640	C	29.829	-6.128	0.968	H	32.927	-2.202	-6.508	H	38.602	0.279	-17.888
H	18.710	-11.960	8.547	H	26.119	-19.611	10.895	C	31.156	-6.165	0.254	H	34.985	-3.788	-7.903	H	39.545	0.900	-15.955
H	18.048	-13.433	8.782	N	23.824	-12.901	6.277	O	32.135	-6.799	0.675	H	32.171	-3.607	-8.641	H	38.027	0.903	-15.352

H	38.978	-0.412	-15.164
H	37.951	-2.203	-16.821
N	36.435	0.030	-19.619
C	36.005	-0.344	-20.958
C	36.966	-1.361	-21.558
O	36.663	-1.933	-22.589
C	35.985	0.857	-21.905
C	35.003	1.972	-21.582
C	33.568	1.537	-21.600
C	33.054	1.160	-22.972
N	31.637	0.723	-22.898
H	36.968	0.900	-19.406
H	35.090	-0.757	-20.905
H	36.914	1.254	-21.923
H	35.778	0.516	-22.834
H	35.227	2.341	-20.666
H	35.133	2.719	-22.252
H	33.458	0.739	-20.992
H	32.992	2.284	-21.242
H	33.123	1.953	-23.577
H	33.610	0.413	-23.337
H	31.423	0.150	-23.689
H	31.493	0.199	-22.058
H	31.040	1.526	-22.897
N	38.110	-1.591	-20.918
C	39.095	-2.549	-21.405
C	39.334	-3.613	-20.335
O	39.170	-3.311	-19.156
C	40.412	-1.836	-21.782
C	40.161	-0.570	-22.604
C	41.271	-1.517	-20.564
H	38.238	-1.032	-20.048
H	38.720	-3.004	-22.213
H	40.938	-2.457	-22.361
H	40.830	0.126	-22.350
H	40.251	-0.784	-23.575
H	39.240	-0.233	-22.418
H	41.812	-0.697	-20.749
H	40.678	-1.360	-19.775
H	41.880	-2.288	-20.380
N	39.694	-4.841	-20.752
C	40.275	-5.870	-19.885
C	39.356	-7.094	-19.819
O	39.153	-7.712	-20.877
C	40.546	-5.371	-18.458
C	40.911	-6.444	-17.439
C	42.003	-7.373	-17.918
C	42.661	-8.144	-16.794
N	43.896	-8.822	-17.256
O	38.762	-7.601	-18.900
H	39.517	-5.000	-21.778
H	41.160	-6.153	-20.265
H	41.300	-4.709	-18.500
H	39.720	-4.898	-18.134
H	41.214	-5.992	-16.590
H	40.087	-6.986	-17.231
H	41.609	-8.030	-18.566
H	42.706	-6.833	-18.387
H	42.890	-7.509	-16.053
H	42.017	-8.831	-16.448
H	43.923	-9.753	-16.890
H	43.904	-8.857	-18.256
H	44.696	-8.315	-16.934
N	29.761	11.650	28.317
C	28.947	10.417	28.505
C	29.881	9.226	28.675
O	29.662	8.164	28.096
C	28.020	10.122	27.316
C	27.894	11.232	26.284
O	27.255	12.267	26.589
O	28.449	11.054	25.187
H	30.754	11.681	28.306
H	28.449	10.534	29.360
H	28.367	9.297	26.857
H	27.106	9.923	27.684
N	30.950	9.420	29.447
C	31.796	8.323	29.899
C	30.989	7.299	30.710
O	31.133	6.083	30.540
C	32.894	8.918	30.778
C	33.880	7.907	31.294
C	35.048	8.577	31.992
O	35.944	7.851	32.477
O	35.060	9.825	32.041
H	31.125	10.418	29.696
H	32.211	7.862	29.106
H	33.387	9.574	30.232
H	32.457	9.342	31.553
H	33.432	7.337	31.953
H	34.242	7.402	30.537
N	30.203	7.832	31.656
C	29.293	7.069	32.499
C	28.133	6.511	31.673
O	27.693	5.385	31.931
C	28.777	7.956	33.610
H	30.306	8.881	31.737
H	29.794	6.297	32.906
H	28.067	8.566	33.255
H	28.390	7.393	34.341
H	29.527	8.506	33.981
N	27.636	7.320	30.713
C	26.574	6.912	29.802
C	26.962	5.658	29.024
O	26.165	4.728	28.913
H	28.085	8.263	30.687
H	25.732	6.729	30.326
H	26.382	7.659	29.152
N	28.206	5.624	28.505
C	28.658	4.478	27.724
C	29.166	3.337	28.614
O	28.958	2.189	28.307
C	29.750	4.893	26.715
C	29.181	5.821	25.646
C	30.384	3.643	26.135
C	30.243	6.498	24.787
H	28.781	6.454	28.707
H	27.846	4.123	27.243
H	30.454	5.389	27.216
H	28.579	5.293	25.051
H	28.642	6.531	26.097
H	31.134	3.898	25.525
H	30.739	3.072	26.874
H	29.700	3.128	25.619
H	30.779	5.798	24.321
H	29.794	7.084	24.117
H	30.836	7.045	25.373
N	29.842	3.638	29.725
C	30.455	2.603	30.532
C	29.334	1.708	31.069
O	28.286	2.218	31.481
C	31.319	3.174	31.674
C	31.703	2.107	32.672
O	32.542	3.752	31.199
H	29.884	4.649	29.946
H	31.046	2.047	29.957
H	30.784	3.914	32.153
H	32.210	2.516	33.437
H	30.880	1.658	33.032
H	32.283	1.415	32.232
H	33.196	3.856	31.955
N	29.541	0.393	30.969
C	28.609	-0.574	31.520
C	28.364	-1.745	30.593
O	29.216	-2.101	29.779
H	30.415	0.132	30.469
H	28.967	-0.918	32.407
H	27.725	-0.113	31.718
N	27.185	-2.353	30.760
C	26.883	-3.615	30.111
C	25.739	-3.374	29.125
O	24.732	-2.738	29.462
C	26.618	-4.744	31.128
C	26.113	-5.993	30.447
O	27.806	-5.140	31.832
H	26.524	-1.849	31.382
H	27.680	-3.914	29.595
H	25.936	-4.407	31.824
H	26.885	-6.580	30.183
H	25.513	-6.507	31.068
H	25.594	-5.751	29.622
H	28.580	-5.214	31.195
N	25.935	-3.876	27.890
C	25.032	-3.639	26.781
C	24.658	-4.960	26.147
O	25.473	-5.880	26.095
C	25.698	-2.730	25.736
C	25.946	-1.337	26.190
C	27.056	-0.887	26.829
C	25.054	-0.219	26.077
C	25.697	0.872	26.689
C	23.771	-0.039	25.548
N	26.921	0.440	27.104
C	25.173	2.154	26.673
C	23.231	1.222	25.576
C	23.910	2.294	26.157
H	26.806	-4.456	27.811
H	24.195	-3.228	27.148
H	26.579	-3.128	25.486
H	25.107	-2.686	24.933
H	27.852	-1.445	27.063
H	27.617	1.018	27.547
H	23.266	-0.804	25.162
H	25.697	2.930	27.023
H	22.328	1.376	25.170
H	23.456	3.189	26.195
N	23.444	-5.011	25.582
C	22.946	-6.216	24.945
C	22.401	-5.884	23.561
O	21.706	-4.886	23.424
C	21.836	-6.816	25.836
C	22.106	-6.843	27.336
C	22.819	-7.887	27.909
C	21.604	-5.865	28.192
C	23.060	-7.955	29.275
C	21.848	-5.901	29.567
C	22.566	-6.961	30.116
O	22.846	-7.057	31.472
H	22.906	-4.124	25.643
H	23.710	-6.858	24.833
H	20.996	-6.285	25.682
H	21.676	-7.762	25.534
H	23.175	-8.623	27.317
H	21.056	-5.117	27.816
H	23.588	-8.715	29.657
H	21.508	-5.169	30.155
H	23.335	-6.237	31.758
N	22.634	-6.750	22.564
C	22.114	-6.502	21.222
C	20.868	-7.349	20.972
O	20.398	-8.070	21.860
C	23.184	-6.578	20.113
C	23.705	-7.946	19.701
H	23.193	-7.575	22.825
H	21.827	-5.541	21.187
H	22.802	-6.121	19.285
H	23.978	-6.013	20.413
N	24.792	-7.954	18.938
O	23.167	-8.987	20.060
H	25.224	-7.092	18.672
H	25.181	-8.822	18.629
N	20.311	-7.213	19.760
C	19.074	-7.895	19.395
C	19.277	-9.419	19.372
O	18.309	-10.154	19.498
C	18.517	-7.288	18.093
C	19.333	-7.529	16.806
C	20.651	-6.788	16.632
H	20.834	-6.587	19.116
H	18.385	-7.693	20.098
H	17.589	-7.658	17.937
H	18.432	-6.287	18.215
H	19.520	-8.532	16.741
H	18.731	-7.303	16.011
N	21.381	-7.167	15.591
O	21.035	-5.898	17.400

H	22.276	-6.708	15.395
H	21.045	-7.914	14.993
N	20.522	-9.905	19.202
C	20.802	-11.323	19.029
C	21.146	-11.989	20.366
O	21.555	-13.147	20.368
C	22.002	-11.508	18.092
C	21.922	-10.914	16.681
C	23.239	-11.169	15.931
C	20.713	-11.454	15.913
H	21.264	-9.168	19.206
H	19.981	-11.773	18.657
H	22.804	-11.098	18.540
H	22.159	-12.495	17.990
H	21.769	-9.933	16.747
H	23.395	-12.153	15.869
H	23.175	-10.778	15.015
H	23.988	-10.739	16.430
H	19.876	-11.217	16.405
H	20.693	-11.047	15.001
H	20.788	-12.447	15.836
N	21.124	-11.223	21.460
C	21.522	-11.717	22.763
C	23.036	-11.782	22.981
O	23.524	-12.563	23.793
H	20.797	-10.246	21.289
H	21.113	-11.126	23.490
H	21.131	-12.651	22.906
N	23.810	-10.999	22.231
C	25.219	-10.833	22.537
C	25.362	-9.795	23.655
O	24.515	-8.893	23.790
C	26.016	-10.428	21.280
O	25.990	-11.435	20.263
H	23.330	-10.538	21.440
H	25.574	-11.700	22.882
H	25.622	-9.588	20.909
H	26.966	-10.265	21.544
H	26.702	-11.240	19.593
N	26.462	-9.928	24.420
C	26.810	-9.068	25.546
C	28.102	-8.272	25.307
O	29.165	-8.868	25.113
C	27.077	-9.917	26.802
C	27.212	-9.077	28.047
O	26.009	-10.858	26.902
H	27.073	-10.742	24.120
H	26.058	-8.405	25.690
H	27.942	-10.424	26.675
H	27.884	-8.344	27.900
H	26.328	-8.662	28.283
H	27.517	-9.645	28.817
H	26.033	-11.502	26.129
N	28.006	-6.950	25.490
C	29.098	-6.002	25.298
C	29.359	-5.298	26.628
O	28.536	-4.505	27.063
C	28.636	-5.076	24.165
C	29.407	-3.819	23.886
C	30.777	-3.857	23.657
C	28.724	-2.628	23.694
C	31.472	-2.698	23.334
C	29.412	-1.472	23.392
C	30.787	-1.505	23.211
H	27.039	-6.643	25.795
H	29.915	-6.533	25.089
H	28.628	-5.625	23.315
H	27.681	-4.811	24.367
H	31.268	-4.719	23.723
H	27.728	-2.611	23.775
H	32.466	-2.738	23.194
H	28.909	-0.594	23.304
H	31.284	-0.664	22.990
N	30.522	-5.549	27.233
C	30.908	-4.931	28.491
C	32.058	-3.971	28.216
O	33.070	-4.392	27.663
C	31.339	-6.021	29.487
C	30.282	-7.131	29.614
C	31.690	-5.403	30.839
C	30.867	-8.461	30.093
H	31.130	-6.230	26.720
H	30.136	-4.374	28.782
H	32.180	-6.439	29.145
H	29.589	-6.836	30.260
H	29.868	-7.272	28.722
H	32.052	-6.110	31.442
H	32.376	-4.691	30.709
H	30.869	-5.006	31.245
H	31.282	-8.327	30.989
H	30.134	-9.133	30.153
H	31.555	-8.765	29.440
N	31.872	-2.702	28.548
C	32.788	-1.675	28.085
C	33.071	-0.693	29.212
O	32.170	-0.291	29.927
C	32.231	-0.993	26.802
C	31.017	-0.119	27.029
C	33.280	-0.194	26.056
H	31.048	-2.516	29.143
H	33.654	-2.114	27.832
H	31.954	-1.733	26.171
H	31.302	0.766	27.402
H	30.539	0.028	26.161
H	30.395	-0.564	27.676
H	33.818	-0.808	25.478
H	32.831	0.494	25.485
H	33.883	0.260	26.713
N	34.328	-0.255	29.288
C	34.679	0.954	30.000
C	35.103	2.041	29.009
O	35.907	1.788	28.115
C	35.795	0.703	31.023
C	36.123	1.950	31.820
O	35.397	-0.357	31.895
H	35.022	-0.857	28.794
H	33.873	1.286	30.499
H	36.636	0.410	30.528
H	36.644	2.593	31.252
H	35.277	2.395	32.124
H	36.671	1.708	32.625
H	34.959	-1.097	31.372
N	34.532	3.232	29.176
C	34.935	4.418	28.438
C	35.887	5.254	29.285
O	35.450	5.871	30.256
C	33.729	5.228	28.121
H	33.763	3.239	29.890
H	35.414	4.136	27.603
H	33.201	4.798	27.381
H	33.137	5.309	28.930
H	33.994	6.153	27.827
N	37.155	5.333	28.874
C	38.117	6.191	29.545
C	37.929	7.674	29.226
O	37.325	8.075	28.215
H	37.382	4.741	28.054
H	38.044	6.059	30.550
H	39.059	5.915	29.280
N	38.496	8.518	30.104
C	38.411	9.967	29.953
C	39.093	10.425	28.659
O	38.750	11.465	28.102
C	39.043	10.648	31.155
H	38.993	8.049	30.888
H	37.441	10.235	29.912
H	40.012	10.405	31.207
H	38.954	11.640	31.064
H	38.582	10.350	31.991
N	40.083	9.636	28.231
C	40.944	9.920	27.100
C	40.319	9.484	25.771
O	40.960	9.624	24.738
C	42.256	9.141	27.258
C	42.120	7.612	27.327
O	41.050	7.103	27.775
O	43.091	6.921	26.914
H	40.185	8.753	28.814
H	41.060	10.924	27.050
H	42.846	9.366	26.477
H	42.702	9.453	28.101
N	39.109	8.915	25.790
C	38.454	8.471	24.561
C	38.651	6.982	24.282
O	38.212	6.482	23.249
H	38.688	8.820	26.732
H	37.460	8.668	24.622
H	38.815	9.005	23.778
N	39.268	6.245	25.209
C	39.451	4.816	25.028
C	38.146	4.082	25.326
O	37.345	4.516	26.146
C	40.554	4.291	25.891
H	39.591	6.771	26.039
H	39.707	4.636	24.074
H	41.114	3.634	25.378
H	41.143	5.044	26.199
H	40.174	3.829	26.698
N	37.942	3.015	24.573
C	37.011	1.950	24.940
C	37.808	0.686	25.136
O	38.638	0.300	24.301
C	35.970	1.681	23.827
C	35.060	2.819	23.402
C	34.170	2.359	22.271
C	34.221	3.397	24.545
H	38.504	2.994	23.689
H	36.574	2.195	25.801
H	36.480	1.377	23.015
H	35.389	0.926	24.144
H	35.618	3.588	23.097
H	33.611	1.584	22.572
H	33.567	3.108	21.987
H	34.731	2.075	21.491
H	34.829	3.750	25.255
H	33.650	4.137	24.193
H	33.642	2.677	24.926
N	37.482	-0.028	26.224
C	38.140	-1.273	26.562
C	37.067	-2.227	27.098
O	36.089	-1.777	27.673
C	39.267	-1.006	27.583
C	40.378	-0.131	27.043
O	38.657	-0.384	28.712
H	36.718	0.396	26.795
H	38.525	-1.673	25.738
H	39.657	-1.891	27.873
H	41.271	-0.538	27.256
H	40.291	-0.040	26.047
H	40.329	0.782	27.458
H	37.872	0.177	28.425
N	37.201	-3.531	26.864
C	36.249	-4.453	27.457
C	36.265	-5.826	26.821
O	37.313	-6.346	26.395
H	37.984	-3.801	26.268
H	36.445	-4.543	28.453
H	35.312	-4.060	27.383
N	35.062	-6.422	26.854
C	34.835	-7.767	26.402
C	33.502	-7.837	25.673
O	32.521	-7.181	26.029
C	34.827	-8.792	27.562
C	36.081	-8.728	28.415
O	33.667	-8.479	28.330
H	34.305	-5.804	27.244
H	35.558	-8.024	25.755
H	34.721	-9.712	27.176
H	36.276	-7.776	28.665
H	35.953	-9.266	29.252
H	36.861	-9.098	27.904
H	33.432	-7.505	28.234
N	33.503	-8.724	24.691
C	32.325	-9.048	23.920
C	32.075	-10.559	24.015

O	32.967	-11.356	23.766
C	32.523	-8.636	22.456
C	31.255	-8.484	21.655
C	30.687	-9.547	20.976
C	30.647	-7.258	21.563
C	29.501	-9.400	20.277
C	29.470	-7.088	20.871
C	28.913	-8.156	20.198
O	27.727	-7.896	19.575
H	34.439	-9.173	24.522
H	31.536	-8.583	24.320
H	33.010	-7.760	22.444
H	33.092	-9.332	22.013
H	31.140	-10.443	20.987
H	31.069	-6.462	22.012
H	29.075	-10.191	19.835
H	29.017	-6.194	20.853
H	27.105	-8.668	19.708
N	30.810	-10.916	24.230
C	30.343	-12.287	24.074
C	29.263	-12.388	22.993
O	28.173	-11.861	23.217
C	29.766	-12.723	25.416
C	28.991	-14.029	25.344
C	29.252	-14.876	26.575
O	29.431	-16.101	26.408
O	29.372	-14.286	27.685
H	30.185	-10.129	24.519
H	31.116	-12.885	23.827
H	30.515	-12.856	26.037
H	29.145	-12.026	25.719
H	28.039	-13.821	25.323
H	29.301	-14.530	24.568
N	29.565	-13.120	21.897
C	28.706	-13.285	20.719
C	27.760	-14.473	20.917
O	28.210	-15.572	21.242
C	29.511	-13.480	19.441
O	28.665	-13.767	18.313
H	30.517	-13.583	21.968
H	28.147	-12.459	20.624
H	30.023	-12.648	19.244
H	30.140	-14.245	19.562
H	28.789	-14.720	18.051
N	26.461	-14.259	20.663
C	25.473	-15.329	20.750
C	25.394	-16.139	19.447
O	24.701	-17.152	19.414
C	24.139	-14.759	21.126
H	26.229	-13.281	20.404
H	25.735	-15.956	21.496
H	24.097	-14.600	22.116
H	23.990	-13.887	20.653
H	23.406	-15.395	20.870
N	26.123	-15.757	18.387
C	26.120	-16.545	17.155
C	27.537	-16.684	16.599
O	28.484	-16.026	17.054
C	25.147	-15.970	16.098
C	23.698	-16.340	16.393
C	25.296	-14.459	15.922
H	26.663	-14.891	18.518
H	25.782	-17.466	17.373
H	25.381	-16.383	15.218
H	23.092	-15.647	16.003
H	23.492	-17.230	15.987
H	23.561	-16.385	17.382
H	25.786	-14.276	15.071
H	24.389	-14.043	15.887
H	25.809	-14.089	16.695
N	27.657	-17.603	15.628
C	28.857	-17.768	14.821
C	29.940	-18.611	15.496
O	29.696	-19.285	16.502
H	26.799	-18.196	15.504
H	28.610	-18.200	13.933
H	29.243	-16.852	14.600
N	31.152	-18.571	14.914
C	32.299	-19.321	15.422
C	32.926	-18.551	16.599
O	34.026	-18.001	16.500
C	33.253	-19.665	14.268
C	34.385	-20.610	14.641
H	31.197	-17.958	14.071
H	31.954	-20.181	15.793
H	32.716	-20.088	13.534
H	33.653	-18.809	13.928
N	35.148	-21.023	13.645
O	34.589	-20.952	15.808
H	34.974	-20.712	12.709
H	35.910	-21.653	13.821
N	32.188	-18.492	17.717
C	32.572	-17.717	18.890
C	32.160	-18.477	20.152
O	31.082	-19.068	20.178
C	31.914	-16.354	18.860
H	31.306	-19.057	17.669
H	33.566	-17.605	18.897
H	31.168	-16.324	19.527
H	32.587	-15.648	19.087
H	31.546	-16.176	17.946
N	32.995	-18.445	21.197
C	32.566	-18.954	22.497
C	33.336	-18.287	23.636
O	34.570	-18.259	23.667
C	32.647	-20.489	22.600
C	34.033	-21.061	22.882
C	34.085	-22.577	23.020
O	33.861	-23.070	24.142
O	34.324	-23.265	21.997
H	33.923	-18.047	21.008
H	31.579	-18.786	22.618
H	32.053	-20.768	23.341
H	32.340	-20.861	21.736
H	34.627	-20.812	22.130
H	34.359	-20.676	23.734
N	32.542	-17.841	24.610
C	33.012	-17.082	25.746
C	33.338	-15.665	25.260
O	32.797	-15.177	24.256
C	34.173	-17.797	26.464
O	34.253	-17.414	27.843
H	31.527	-18.097	24.472
H	32.275	-17.009	26.411
H	34.025	-18.779	26.413
H	35.026	-17.552	26.015
H	33.655	-16.632	27.998
N	34.177	-14.988	26.031
C	34.359	-13.561	25.895
C	35.589	-13.334	25.028
O	36.525	-14.153	24.994
C	34.479	-12.892	27.270
C	33.480	-13.379	28.323
C	33.333	-12.506	29.603
N	32.233	-11.976	28.786
C	30.951	-11.970	29.164
N	30.015	-11.576	28.327
N	30.603	-12.384	30.372
H	34.685	-15.570	26.733
H	33.568	-13.168	25.434
H	35.399	-13.062	27.621
H	34.343	-11.909	27.152
H	32.558	-13.449	27.905
H	33.742	-14.310	28.629
H	32.998	-13.039	30.231
H	34.010	-11.929	29.589
H	32.460	-11.594	27.885
H	30.233	-11.295	27.394
H	29.050	-11.556	28.627
H	31.314	-12.723	30.998
H	29.651	-12.363	30.661
N	35.556	-12.190	24.331
C	36.681	-11.746	23.529
C	37.025	-10.311	23.919
O	36.147	-9.532	24.295
C	36.350	-11.880	22.039
C	36.068	-13.292	21.579
C	34.846	-13.908	21.815
C	37.029	-14.008	20.895
C	34.591	-15.197	21.375
C	36.806	-15.319	20.502
C	35.577	-15.911	20.732
O	35.404	-17.213	20.267
H	34.664	-11.662	24.419
H	37.471	-12.321	23.738
H	35.543	-11.321	21.848
H	37.127	-11.531	21.515
H	34.131	-13.411	22.315
H	37.906	-13.576	20.675
H	33.686	-15.607	21.525
H	37.536	-15.838	20.051
H	35.322	-17.189	19.275
N	38.320	-10.007	23.839
C	38.821	-8.669	24.061
C	38.281	-7.740	22.964
O	38.317	-8.107	21.787
C	40.359	-8.638	24.093
C	40.871	-7.190	24.111
C	40.916	-9.444	25.289
H	38.934	-10.821	23.602
H	38.472	-8.335	24.937
H	40.692	-9.047	23.250
H	41.070	-6.902	23.177
H	40.169	-6.600	24.504
H	41.700	-7.143	24.663
H	41.505	-8.848	25.827
H	40.151	-9.764	25.841
H	41.436	-10.217	24.937
N	37.790	-6.574	23.387
C	37.518	-5.479	22.458
C	38.290	-4.231	22.863
O	38.648	-4.000	24.021
C	36.015	-5.200	22.384
C	35.397	-4.395	23.508
C	35.481	-2.883	23.280
C	33.968	-4.846	23.698
H	37.623	-6.515	24.407
H	37.819	-5.756	21.540
H	35.843	-4.706	21.527
H	35.546	-6.085	22.350
H	35.866	-4.618	24.375
H	34.999	-2.649	22.437
H	35.059	-2.409	24.051
H	36.440	-2.614	23.204
H	33.947	-5.821	23.930
H	33.542	-4.323	24.440
H	33.449	-4.699	22.853
N	38.617	-3.452	21.834
C	39.158	-2.131	22.018
C	38.536	-1.213	20.962
O	38.225	-1.643	19.848
C	40.686	-2.183	21.982
C	41.282	-2.537	20.633
O	41.107	-0.880	22.344
H	38.446	-3.885	20.899
H	38.895	-1.788	22.917
H	41.008	-2.833	22.679
H	40.961	-1.890	19.936
H	42.283	-2.495	20.679
H	41.006	-3.465	20.369
H	40.430	-0.440	22.948
N	38.392	0.040	21.332
C	37.896	1.069	20.435
C	38.144	2.484	20.957
O	38.977	2.710	21.850
H	38.664	0.236	22.325
H	38.341	0.971	19.528
H	36.899	0.942	20.292
N	37.408	3.423	20.370
C	37.535	4.839	20.618
C	36.144	5.443	20.577
O	35.237	4.998	19.853
C	38.466	5.504	19.590
C	39.888	4.967	19.495
C	40.796	5.337	20.645

N	40.945	6.794	20.756	H	35.217	11.293	20.840	H	28.453	18.015	17.983	C	26.196	11.368	19.433	H	30.457	3.705	21.903
C	41.369	7.423	21.846	H	33.612	11.428	19.087	N	26.502	19.327	21.443	O	26.873	11.890	18.501	N	33.425	3.530	17.974
N	41.868	6.707	22.841	H	32.402	11.365	20.181	C	25.488	19.658	22.444	H	23.727	12.256	17.661	C	33.665	2.288	17.261
N	41.269	8.746	21.936	N	34.976	11.819	23.195	C	24.321	20.304	21.707	H	24.272	11.095	20.222	C	34.548	1.355	18.072
H	36.700	3.026	19.685	C	34.842	12.208	24.596	O	24.018	21.477	21.897	H	24.468	10.385	18.766	O	35.291	1.804	18.962
H	37.920	4.983	21.527	C	34.448	13.673	24.781	C	26.002	20.629	23.527	N	26.765	10.470	20.230	H	34.030	3.867	18.757
H	38.039	5.411	18.682	O	34.331	14.120	25.928	C	27.016	20.026	24.480	C	28.206	10.363	20.420	H	32.786	1.832	17.071
H	38.521	6.485	19.813	C	36.115	11.930	25.311	O	26.578	21.759	22.862	C	28.798	9.289	19.517	H	34.112	2.486	16.379
H	39.854	3.956	19.434	O	37.188	12.603	24.674	H	26.712	20.059	20.727	O	28.580	8.106	19.775	N	34.482	0.054	17.757
H	40.311	5.307	18.639	H	35.897	11.807	22.696	H	25.170	18.818	22.864	C	28.529	9.995	21.865	C	35.342	-0.902	18.415
H	40.399	4.987	21.486	H	34.111	11.638	25.010	H	25.215	20.949	24.078	C	29.994	9.922	22.177	C	35.554	-2.078	17.487
H	41.689	4.932	20.484	H	36.055	12.253	26.251	H	27.637	20.739	24.816	O	27.909	11.024	22.620	O	34.832	-2.231	16.503
H	40.709	7.349	19.950	H	36.306	10.952	25.298	H	26.545	19.608	25.261	H	26.081	9.837	20.716	C	34.803	-1.258	19.809
H	41.956	5.720	22.796	H	36.859	13.037	23.836	H	27.553	19.322	24.008	H	28.627	11.246	20.180	C	33.527	-2.065	19.804
H	42.175	7.183	23.685	N	34.202	14.425	23.698	H	27.405	21.485	22.359	H	28.090	9.123	22.087	C	33.456	-3.419	19.764
H	40.889	9.264	21.165	C	33.563	15.734	23.843	N	23.742	19.538	20.788	H	30.391	10.846	22.218	C	32.167	-1.605	19.832
H	41.569	9.218	22.759	C	32.520	15.966	22.756	C	22.656	19.992	19.939	H	30.142	9.472	23.065	C	31.340	-2.755	19.828
N	35.975	6.518	21.344	O	32.735	16.775	21.860	C	21.644	18.850	19.804	H	30.478	9.397	21.468	C	31.542	-0.375	19.864
C	34.754	7.279	21.271	C	34.609	16.841	23.854	O	20.792	18.864	18.911	H	28.155	10.949	23.595	N	32.166	-3.836	19.829
C	35.098	8.759	21.369	H	34.490	14.017	22.803	C	23.209	20.469	18.577	N	29.665	9.689	18.563	C	29.953	-2.695	19.846
O	36.176	9.100	21.836	H	33.106	15.772	24.741	C	23.787	19.350	17.749	C	30.287	8.697	17.672	C	30.164	-0.313	19.899
C	33.758	6.803	22.332	H	35.505	16.456	23.635	O	24.566	18.505	18.267	C	31.293	7.806	18.398	C	29.376	-1.461	19.914
C	34.081	7.315	23.719	H	34.369	17.533	23.174	O	23.557	19.274	16.512	O	32.061	8.272	19.245	H	33.788	-0.182	17.032
C	35.149	6.835	24.442	H	34.640	17.262	24.760	H	24.143	18.567	20.730	C	30.947	9.414	16.499	H	36.231	-0.469	18.565
C	33.314	8.324	24.273	N	31.336	15.298	22.809	H	22.190	20.735	20.414	H	29.920	10.699	18.491	H	35.487	-1.797	20.291
C	35.434	7.330	25.715	C	30.362	15.352	21.704	H	22.399	20.978	18.009	H	29.556	8.111	17.287	H	34.614	-0.416	20.306
C	33.581	8.821	25.539	C	29.715	16.657	21.492	H	24.006	21.223	18.757	H	31.810	10.017	16.857	H	34.254	-4.031	19.695
C	34.635	8.313	26.255	O	29.752	17.564	22.361	N	21.775	17.857	20.686	H	31.293	8.675	15.746	H	31.862	-4.795	19.872
O	34.827	8.840	27.501	C	29.334	14.276	22.091	C	20.985	16.641	20.634	H	30.209	10.083	16.011	H	32.077	0.477	19.863
H	36.768	6.740	21.966	C	29.430	14.235	23.594	C	21.626	15.504	19.826	N	31.333	6.509	18.035	H	29.408	-3.540	19.809
H	34.325	7.120	20.382	C	30.899	14.425	23.902	O	21.037	14.436	19.750	C	32.237	5.535	18.606	H	29.722	0.588	19.913
H	32.855	7.130	22.081	H	30.865	15.042	20.769	H	22.502	18.037	21.421	C	32.412	4.332	17.680	H	28.386	-1.371	19.975
H	33.777	5.810	22.355	H	28.419	14.542	21.802	H	20.811	16.307	21.583	O	31.721	4.219	16.666	N	36.585	-2.851	17.830
H	35.737	6.119	24.059	H	29.578	13.396	21.696	H	20.069	16.847	20.234	C	31.719	5.077	19.977	C	37.004	-4.048	17.153
H	32.549	8.706	23.750	H	28.883	14.970	23.999	N	22.801	15.727	19.224	C	30.258	4.623	20.045	C	37.073	-5.210	18.136
H	36.216	6.970	26.226	H	29.104	13.353	23.940	C	23.425	14.676	18.424	C	29.998	3.381	19.240	O	37.618	-5.061	19.236
H	33.006	9.545	25.924	H	31.028	14.868	24.785	C	23.954	13.541	19.268	C	29.869	4.407	21.499	C	38.395	-3.891	16.540
H	35.735	8.585	27.833	H	31.387	13.556	23.881	O	24.292	13.682	20.430	H	30.631	6.272	17.284	C	38.808	-5.077	15.696
N	34.171	9.596	20.909	N	28.953	16.814	20.301	C	24.516	15.216	17.493	H	33.141	5.963	18.736	O	38.422	-2.726	15.714
C	34.266	11.040	21.006	C	28.134	17.995	20.135	O	23.923	15.845	16.384	H	32.283	4.309	20.273	H	37.096	-2.480	18.697
C	33.944	11.451	22.445	C	27.021	18.052	21.166	H	23.320	16.617	19.368	H	31.824	5.839	20.611	H	36.328	-4.280	16.444
O	32.794	11.427	22.851	O	26.772	17.109	21.961	H	22.637	14.250	17.764	H	29.674	5.376	19.708	H	39.077	-3.765	17.290
C	33.331	11.699	20.010	C	27.611	18.064	18.701	H	25.193	15.897	18.050	H	30.565	2.626	19.580	H	38.104	-5.792	15.739
C	33.307	13.224	20.071	H	28.637	15.935	19.834	H	25.143	14.381	17.112	H	29.032	3.120	19.313	H	38.927	-4.795	14.740
O	34.110	13.810	20.810	H	28.781	18.886	20.295	H	23.892	16.823	16.609	H	30.219	3.544	18.275	H	39.675	-5.454	16.033
O	32.679	13.882	19.203	H	26.928	17.218	18.501	N	24.025	12.366	18.655	H	29.978	5.263	22.006	H	39.272	-2.696	15.176
H	33.349	9.108	20.461	H	27.065	19.015	18.528	C	24.673	11.238	19.292	H	28.915	4.110	21.550	N	36.579	-6.354	17.670

C	36.920	-7.662	18.227
C	37.577	-8.529	17.168
O	37.028	-8.725	16.079
C	35.650	-8.361	18.721
C	35.926	-9.811	19.110
C	35.022	-7.572	19.835
H	35.919	-6.237	16.863
H	37.580	-7.521	18.970
H	34.998	-8.386	17.965
H	35.774	-10.402	18.318
H	36.873	-9.898	19.415
H	35.310	-10.079	19.850
H	35.081	-8.089	20.692
H	35.502	-6.698	19.944
H	34.058	-7.394	19.624
N	38.754	-9.074	17.495
C	39.309	-10.179	16.740
C	38.861	-11.468	17.443
O	38.925	-11.551	18.665
C	40.801	-10.085	16.673
H	39.216	-8.641	18.316
H	38.933	-10.177	15.823
H	41.106	-9.183	16.990
H	41.219	-10.789	17.254
H	41.115	-10.218	15.729
N	38.410	-12.444	16.647
C	37.682	-13.605	17.119
C	38.661	-14.733	17.458
O	38.552	-15.858	16.971
C	36.625	-14.021	16.101
C	35.558	-12.974	15.968
C	35.406	-12.129	14.917
C	34.553	-12.617	16.920
C	33.829	-11.535	16.375
C	34.188	-13.068	18.194
N	34.360	-11.281	15.137
C	32.755	-10.946	17.039
C	33.150	-12.460	18.859
C	32.423	-11.422	18.286
H	38.637	-12.288	15.628
H	37.211	-13.355	17.968
H	37.047	-14.124	15.219
H	36.189	-14.848	16.406
H	35.980	-12.126	14.094
H	34.028	-10.582	14.500
H	34.681	-13.833	18.620
H	32.244	-10.192	16.607
H	32.914	-12.776	19.782
H	31.659	-11.024	18.783
N	39.609	-14.383	18.319
C	40.544	-15.319	18.900
C	40.537	-15.110	20.408
O	40.565	-13.989	20.912
C	41.940	-15.098	18.322
C	43.016	-16.012	18.882
C	44.392	-15.772	18.323
C	45.467	-16.547	19.062
N	45.467	-16.246	20.521
H	39.618	-13.353	18.542
H	40.247	-16.250	18.705
H	41.894	-15.239	17.331
H	42.207	-14.149	18.505
H	43.045	-15.896	19.890
H	42.750	-16.974	18.700
H	44.409	-16.051	17.361
H	44.608	-14.796	18.387
H	45.310	-17.530	18.930
H	46.364	-16.309	18.678
H	45.440	-17.102	21.035
H	46.297	-15.740	20.754
H	44.664	-15.695	20.745
N	40.419	-16.222	21.150
C	40.626	-16.196	22.589
C	41.336	-17.513	22.960
O	41.916	-18.193	22.101
C	39.307	-15.924	23.342
C	38.226	-16.993	23.184
H	40.174	-17.073	20.614
H	41.263	-15.465	22.790
H	39.520	-15.833	24.321
H	38.936	-15.049	23.013
N	37.030	-16.698	23.675
O	38.455	-18.057	22.597
H	36.874	-15.811	24.114
H	36.282	-17.358	23.608
N	41.268	-17.893	24.237
C	41.893	-19.134	24.697
C	41.184	-20.402	24.207
O	41.795	-21.462	24.128
C	42.046	-19.101	26.213
C	43.151	-18.142	26.576
H	40.748	-17.249	24.858
H	42.832	-19.156	24.310
H	41.203	-18.783	26.607
H	42.288	-20.002	26.524
N	42.932	-17.328	27.592
O	44.180	-18.115	25.904
H	42.058	-17.374	28.087
H	43.627	-16.668	27.873
N	39.916	-20.296	23.826
C	39.122	-21.454	23.472
C	39.046	-21.636	21.956
O	39.023	-22.773	21.482
C	37.770	-21.309	24.170
C	37.914	-21.076	25.657
C	38.542	-22.016	26.464
C	37.455	-19.909	26.257
C	38.699	-21.811	27.827
C	37.603	-19.689	27.616
C	38.232	-20.642	28.404
O	38.396	-20.435	29.753
H	39.551	-19.319	23.806
H	39.560	-22.277	23.860
H	37.296	-20.533	23.777
H	37.257	-22.145	24.035
H	38.891	-22.865	26.054
H	37.008	-19.212	25.693
H	39.150	-22.506	28.392
H	37.258	-18.846	28.031
H	37.675	-19.827	30.082
N	39.029	-20.543	21.174
C	38.680	-20.646	19.760
C	39.406	-19.606	18.908
O	39.835	-18.577	19.415
C	37.164	-20.479	19.623
C	36.420	-21.791	19.808
C	35.278	-21.967	18.836
N	33.985	-21.882	19.484
C	32.851	-22.257	18.913
N	31.736	-22.328	19.622
N	32.853	-22.600	17.637
H	39.275	-19.664	21.648
H	38.936	-21.567	19.428
H	36.857	-19.848	20.319
H	36.970	-20.138	18.716
H	37.061	-22.555	19.683
H	36.048	-21.831	20.740
H	35.348	-21.252	18.140
H	35.374	-22.864	18.403
H	33.945	-21.518	20.420
H	31.722	-22.083	20.589
H	30.883	-22.634	19.180
H	33.698	-22.558	17.101
H	32.004	-22.904	17.196
N	39.609	-19.918	17.633
C	40.128	-18.928	16.699
C	39.348	-19.030	15.393
O	39.450	-20.017	14.664
C	41.644	-19.001	16.500
C	42.165	-17.796	15.738
H	39.375	-20.889	17.373
H	39.938	-18.026	17.083
H	42.077	-19.025	17.391
H	41.854	-19.820	15.982
N	43.481	-17.697	15.623
O	41.374	-16.972	15.249
H	44.074	-18.398	16.022
H	43.887	-16.922	15.136
N	38.552	-17.991	15.095
C	37.740	-18.009	13.895
C	38.409	-17.292	12.731
O	37.782	-17.153	11.680
C	36.398	-17.418	14.202
H	38.576	-17.217	15.774
H	37.576	-18.973	13.625
H	35.797	-17.490	13.402
H	35.973	-17.905	14.970
H	36.494	-16.449	14.447
N	39.648	-16.818	12.927
C	40.444	-16.200	11.880
C	39.671	-15.067	11.191
O	39.600	-14.999	9.953
C	40.940	-17.257	10.887
C	41.736	-18.340	11.55
H	39.991	-16.936	13.913
H	41.272	-15.802	12.294
H	40.152	-17.676	10.435
H	41.519	-16.814	10.202
C	41.582	-19.684	11.550
N	42.812	-18.077	12.376
C	43.283	-19.232	12.852
N	42.545	-20.208	12.381
H	43.178	-17.177	12.584
H	40.899	-20.202	11.041
H	44.066	-19.324	13.461
N	39.099	-14.197	12.027
C	38.219	-13.130	11.554
C	38.160	-12.016	12.600
O	38.563	-12.203	13.758
C	36.852	-13.678	11.198
O	36.189	-14.158	12.346
H	39.332	-14.344	13.030
H	38.614	-12.746	10.719
H	36.306	-12.951	10.788
H	36.961	-14.428	10.550
H	36.621	-13.782	13.166
N	37.635	-10.851	12.187
C	37.529	-9.692	13.064
C	36.313	-8.848	12.684
O	35.985	-8.766	11.505
C	38.802	-8.878	12.969
H	37.316	-10.855	11.196
H	37.414	-10.008	14.011
H	39.436	-9.314	12.329
H	38.590	-7.955	12.645
H	39.234	-8.819	13.870
N	35.599	-8.307	13.679
C	34.453	-7.442	13.453
C	34.776	-6.045	13.963
O	35.333	-5.898	15.054
C	33.189	-7.982	14.145
C	31.963	-7.118	13.949
O	32.987	-9.270	13.556
H	35.943	-8.565	14.634
H	34.280	-7.387	12.469
H	33.379	-8.103	15.120
H	32.231	-6.154	13.862
H	31.475	-7.396	13.117
H	31.345	-7.213	14.735
H	33.251	-9.257	12.585
N	34.423	-5.026	13.178
C	34.362	-3.660	13.680
C	32.922	-3.180	13.659
O	32.149	-3.405	12.720
C	35.247	-2.687	12.879
C	36.706	-3.046	12.942
O	34.783	-2.639	11.526
H	34.201	-5.289	12.199
H	34.686	-3.653	14.628
H	35.157	-1.755	13.281
H	36.818	-4.045	12.980
H	37.186	-2.699	12.129
H	37.129	-2.644	13.761
H	34.426	-3.537	11.246
N	32.563	-2.492	14.749

C	31.324	-1.792	14.919
C	31.598	-0.299	14.872
O	32.424	0.184	15.619
C	30.646	-2.129	16.246
C	30.109	-3.530	16.369
C	28.796	-3.910	16.304
C	30.834	-4.705	16.736
C	29.890	-5.755	16.859
C	32.174	-4.981	17.019
N	28.680	-5.245	16.547
C	30.259	-7.058	17.177
C	32.539	-6.268	17.360
C	31.583	-7.283	17.446
H	33.316	-2.516	15.506
H	30.727	-2.011	14.146
H	31.306	-2.004	16.989
H	29.873	-1.507	16.383
H	28.034	-3.292	16.106
H	27.819	-5.770	16.500
H	32.865	-4.257	16.975
H	29.575	-7.789	17.205
H	33.495	-6.471	17.547
H	31.884	-8.204	17.714
N	30.824	0.394	14.035
C	30.877	1.818	13.888
C	29.496	2.350	14.198
O	28.487	1.927	13.618
C	31.291	2.193	12.494
O	31.556	3.583	12.459
H	30.159	-0.220	13.484
H	31.507	2.188	14.565
H	32.115	1.704	12.249
H	30.555	1.993	11.864
H	32.151	3.790	11.683
N	29.407	3.291	15.137
C	28.093	3.800	15.418
C	28.065	4.974	16.392
O	28.980	5.799	16.436
H	30.267	3.575	15.579
H	27.642	4.090	14.545
H	27.507	3.050	15.796
N	26.892	5.150	16.997
C	26.723	6.262	17.911
C	25.868	5.846	19.108
O	24.924	5.076	18.981
C	26.204	7.513	17.203
C	24.788	7.509	16.688
C	24.462	8.747	15.867
H	26.166	4.465	16.768
H	27.628	6.524	18.262
H	26.304	8.304	17.844
H	26.825	7.711	16.414
H	24.652	6.705	16.109
H	24.157	7.471	17.463
N	23.942	8.549	14.653
O	24.610	9.880	16.326
H	23.584	9.386	14.148
H	23.805	7.589	14.335
N	26.250	6.420	20.252
C	25.504	6.327	21.503
C	24.497	7.469	21.532
O	24.847	8.631	21.265
C	26.526	6.418	22.629
C	25.909	6.808	23.948
C	25.271	5.856	24.729
C	25.903	8.127	24.368
C	24.665	6.200	25.924
C	25.347	8.478	25.593
C	24.716	7.513	26.359
O	24.147	7.801	27.583
H	27.154	6.956	20.171
H	25.002	5.468	21.508
H	26.956	5.529	22.735
H	27.201	7.105	22.386
H	25.247	4.900	24.423
H	26.301	8.834	23.790
H	24.194	5.506	26.471
H	25.403	9.421	25.917
H	24.720	7.421	28.306
N	23.248	7.136	21.897
C	22.153	8.074	22.013
C	21.616	7.909	23.438
O	21.083	6.860	23.778
C	21.056	7.787	20.963
C	19.948	8.822	20.994
C	21.607	7.666	19.549
H	23.140	6.102	22.095
H	22.507	8.992	21.929
H	20.643	6.905	21.195
H	19.065	8.375	20.842
H	19.938	9.278	21.885
H	20.102	9.502	20.276
H	22.241	6.894	19.507
H	20.851	7.516	18.912
H	22.087	8.509	19.309
N	21.768	8.941	24.255
C	21.415	8.852	25.665
C	19.927	9.108	25.873
O	19.201	9.365	24.926
H	22.151	9.798	23.813
H	21.646	7.935	26.013
H	21.945	9.530	26.189
N	19.501	9.099	27.135
C	18.118	9.368	27.482
C	17.541	8.174	28.222
O	18.288	7.286	28.639
H	20.240	8.885	27.838
H	18.067	10.179	28.068
H	17.586	9.532	26.649
N	16.215	8.140	28.359
C	15.603	7.207	29.296
C	15.821	5.790	28.795
O	16.052	4.877	29.600
C	14.140	7.515	29.466
H	15.692	8.800	27.772
H	16.046	7.305	30.188
H	13.596	6.684	29.327
H	13.968	7.865	30.390
H	13.855	8.206	28.798
N	15.753	5.649	27.459
C	15.951	4.383	26.783
C	17.199	4.502	25.907
O	17.096	4.633	24.691
C	14.714	4.017	25.960
C	13.423	4.022	26.774
C	12.607	2.749	26.654
O	12.429	2.267	25.521
O	12.148	2.247	27.698
H	15.543	6.545	26.956
H	16.083	3.653	27.448
H	14.618	4.685	25.249
H	14.841	3.109	25.612
H	13.662	4.135	27.726
H	12.860	4.768	26.453
N	18.359	4.430	26.570
C	19.655	4.657	25.955
C	19.981	3.520	24.984
O	19.687	2.344	25.216
C	20.679	4.797	27.042
H	18.244	4.191	27.589
H	19.628	5.517	25.438
H	20.593	5.694	27.484
H	20.549	4.082	27.733
H	21.603	4.713	26.659
N	20.658	3.870	23.878
C	20.924	2.909	22.827
C	22.311	3.191	22.249
O	22.745	4.358	22.232
C	19.873	3.074	21.710
C	18.682	2.125	21.778
C	17.633	2.459	20.732
N	16.689	1.370	20.535
C	15.881	0.862	21.457
N	15.080	-0.138	21.137
N	15.857	1.346	22.688
H	20.959	4.859	23.854
H	20.923	1.988	23.212
H	19.525	4.012	21.752
H	20.333	2.932	20.833
H	18.995	1.191	21.620
H	18.257	2.193	22.678
H	17.142	3.273	21.036
H	18.104	2.649	19.873
H	16.642	0.957	19.603
H	15.074	-0.541	20.228
H	14.449	-0.512	21.838
H	16.481	2.101	22.925
H	15.238	0.974	23.369
N	22.955	2.143	21.764
C	24.073	2.304	20.828
C	23.635	1.684	19.510
O	23.340	0.499	19.435
C	25.371	1.694	21.371
C	25.767	2.356	22.703
C	26.499	1.792	20.345
C	26.918	1.693	23.386
H	22.615	1.222	22.090
H	24.164	3.277	20.671
H	25.211	0.728	21.549
H	25.993	3.312	22.515
H	24.966	2.332	23.303
H	27.310	1.332	20.700
H	26.212	1.355	19.495
H	26.707	2.752	20.173
H	27.726	1.718	22.794
H	27.126	2.169	24.243
H	26.690	0.738	23.589
N	23.565	2.509	18.474
C	23.137	2.065	17.170
C	24.380	1.909	16.292
O	25.105	2.892	16.125
C	22.208	3.096	16.535
C	20.867	3.228	17.232
H	23.844	3.493	18.687
H	22.742	1.155	17.271
H	22.664	3.988	16.555
H	22.045	2.830	15.582
N	20.432	4.457	17.399
O	20.266	2.231	17.623
H	20.960	5.243	17.076
H	19.550	4.622	17.860
N	24.568	0.728	15.698
C	25.809	0.405	15.004
C	25.535	-0.208	13.639
O	24.472	-0.784	13.372
C	26.760	-0.532	15.750
C	27.097	0.019	17.114
O	26.249	-1.880	15.821
H	23.765	0.073	15.774
H	26.332	1.257	14.870
H	27.660	-0.568	15.249
H	26.256	0.182	17.639
H	27.672	-0.632	17.618
H	27.595	0.887	17.024
H	25.277	-1.867	16.069
N	26.537	-0.010	12.779
C	26.751	-0.910	11.669
C	28.071	-1.603	11.870
O	28.973	-1.058	12.492
C	26.743	-0.058	10.392
C	25.329	0.495	10.198
C	24.978	1.118	8.869
H	27.122	0.820	12.979
H	26.030	-1.602	11.684
H	27.355	0.694	10.505
H	26.959	-0.626	9.627
H	24.668	-0.267	10.381
H	25.162	1.189	10.934
N	24.799	2.417	8.883
O	24.768	0.440	7.870
H	24.570	2.928	8.015
H	24.886	2.930	9.758
N	28.201	-2.807	11.329
C	29.410	-3.555	11.519
C	29.916	-4.172	10.209

O	29.124	-4.429	9.291
C	29.243	-4.613	12.617
C	28.111	-5.570	12.491
C	26.947	-5.541	13.209
C	28.067	-6.771	11.698
C	26.828	-7.390	11.951
C	28.920	-7.347	10.753
N	26.167	-6.609	12.856
C	26.419	-8.554	11.299
C	28.542	-8.527	10.152
C	27.307	-9.108	10.405
H	27.386	-3.134	10.786
H	30.130	-2.923	11.843
H	30.093	-5.159	12.663
H	29.135	-4.137	13.503
H	26.706	-4.850	13.883
H	25.242	-6.794	13.210
H	29.787	-6.913	10.515
H	25.524	-8.955	11.482
H	29.178	-8.972	9.519
H	27.057	-9.952	9.924
N	31.226	-4.412	10.206
C	31.938	-5.179	9.199
C	32.704	-6.325	9.852
O	33.540	-6.091	10.722
C	32.932	-4.280	8.475
C	32.372	-3.110	7.664
C	33.499	-2.147	7.255
C	31.654	-3.619	6.418
H	31.726	-3.975	11.039
H	31.271	-5.570	8.558
H	33.553	-3.892	9.162
H	33.461	-4.851	7.843
H	31.707	-2.614	8.219
H	34.164	-2.641	6.699
H	33.110	-1.394	6.728
H	33.940	-1.793	8.077
H	30.900	-4.217	6.689
H	31.295	-2.842	5.900
H	32.298	-4.131	5.849
N	32.473	-7.524	9.311
C	33.130	-8.752	9.725
C	33.963	-9.292	8.575
O	33.406	-9.710	7.552
C	32.045	-9.747	10.161
C	32.519	-11.086	10.734
C	31.314	-11.939	11.099
C	33.417	-11.818	9.796
H	31.753	-7.499	8.537
H	33.741	-8.549	10.492
H	31.491	-9.303	10.864
H	31.479	-9.948	9.364
H	33.064	-10.914	11.549
H	30.761	-12.105	10.282
H	31.624	-12.814	11.473
H	30.761	-11.462	11.783
H	34.235	-11.268	9.594
H	33.710	-12.691	10.201
H	32.939	-12.010	8.932
N	35.294	-9.217	8.742
C	36.248	-9.682	7.753
C	36.808	-11.031	8.206
O	37.229	-11.223	9.354
C	37.390	-8.693	7.476
C	38.323	-9.174	6.387
O	36.846	-7.431	7.059
H	35.577	-8.789	9.658
H	35.775	-9.817	6.884
H	37.914	-8.542	8.339
H	38.434	-10.170	6.444
H	37.949	-8.940	5.485
H	39.223	-8.741	6.488
H	36.686	-6.841	7.856
N	36.820	-11.963	7.251
C	37.397	-13.291	7.438
C	38.683	-13.332	6.627
O	38.705	-12.866	5.477
C	36.387	-14.364	6.985
C	35.091	-14.308	7.798
C	36.997	-15.753	6.980
C	33.881	-13.847	6.989
H	36.377	-11.657	6.352
H	37.659	-13.350	8.392
H	36.159	-14.186	6.028
H	34.904	-15.216	8.163
H	35.222	-13.674	8.557
H	36.351	-16.402	6.579
H	37.838	-15.749	6.441
H	37.206	-16.031	7.917
H	33.736	-14.480	6.233
H	33.080	-13.836	7.582
H	34.056	-12.931	6.637
N	39.768	-13.820	7.244
C	41.000	-14.084	6.520
C	40.749	-15.007	5.325
O	40.181	-16.066	5.499
H	39.646	-13.986	8.260
H	41.392	-13.218	6.188
H	41.669	-14.518	7.135
N	41.083	-14.568	4.109
C	40.804	-15.362	2.917
C	42.069	-15.404	2.062
O	42.997	-14.633	2.281
C	39.642	-14.841	2.041
C	38.334	-14.610	2.771
O	40.052	-13.634	1.403
H	41.543	-13.643	4.090
H	40.582	-16.292	3.196
H	39.441	-15.534	1.323
H	37.566	-14.679	2.129
H	38.218	-15.298	3.493
H	38.330	-13.697	3.187
H	40.556	-13.836	0.555
N	42.087	-16.282	1.054
C	43.037	-16.156	-0.038
C	42.649	-14.945	-0.888
O	41.500	-14.479	-0.878
C	43.079	-17.449	-0.874
C	43.222	-18.690	-0.020
O	41.857	-17.529	-1.611
H	41.381	-17.036	1.124
H	43.946	-15.993	0.340
H	43.847	-17.388	-1.532
H	43.635	-18.454	0.864
H	42.321	-19.102	0.142
H	43.808	-19.361	-0.483
H	42.000	-18.023	-2.476
N	43.601	-14.490	-1.698
C	43.383	-13.375	-2.606
C	42.254	-13.724	-3.584
O	41.454	-12.856	-3.916
C	44.708	-13.002	-3.288
C	45.792	-12.582	-2.292
C	47.225	-12.534	-2.806
O	48.047	-13.372	-2.360
O	47.533	-11.637	-3.599
H	44.509	-14.999	-1.624
H	43.130	-12.556	-2.090
H	45.033	-13.793	-3.762
H	44.538	-12.241	-3.877
H	45.565	-11.667	-1.962
H	45.773	-13.227	-1.528
N	42.148	-15.008	-3.975
C	41.128	-15.496	-4.898
C	39.708	-15.296	-4.351
O	38.813	-14.949	-5.122
C	41.371	-16.963	-5.187
H	42.877	-15.634	-3.548
H	41.206	-14.988	-5.769
H	40.695	-17.294	-5.847
H	42.289	-17.085	-5.566
H	41.291	-17.492	-4.341
N	39.531	-15.572	-3.047
C	38.264	-15.470	-2.322
C	38.066	-14.123	-1.590
O	37.082	-13.964	-0.888
C	38.138	-16.604	-1.303
C	38.024	-17.954	-1.978
H	40.428	-15.883	-2.570
H	37.518	-15.536	-3.008
H	38.948	-16.601	-0.729
H	37.322	-16.448	-0.760
N	38.347	-19.002	-1.247
O	37.628	-18.036	-3.137
H	38.643	-18.886	-0.296
H	38.298	-19.926	-1.637
N	38.911	-13.119	-1.823
C	38.825	-11.844	-1.108
C	37.484	-11.136	-1.357
O	36.919	-10.507	-0.457
C	39.996	-10.948	-1.493
H	39.630	-13.317	-2.549
H	38.911	-12.020	-0.118
H	39.783	-10.001	-1.256
H	40.814	-11.238	-0.999
H	40.159	-11.017	-2.477
N	36.944	-11.271	-2.568
C	35.654	-10.702	-2.914
C	34.564	-11.147	-1.941
O	33.628	-10.393	-1.721
C	35.276	-11.016	-4.372
C	34.911	-12.448	-4.601
C	35.776	-13.478	-4.866
C	33.604	-13.042	-4.462
C	33.755	-14.425	-4.734
C	32.316	-12.532	-4.221
N	35.087	-14.656	-4.972
C	32.672	-15.308	-4.705
C	31.243	-13.402	-4.217
C	31.420	-14.767	-4.471
H	37.529	-11.819	-3.239
H	35.728	-9.698	-2.844
H	34.491	-10.463	-4.622
H	36.053	-10.808	-4.953
H	36.765	-13.380	-4.967
H	35.486	-15.549	-5.189
H	32.179	-11.558	-4.055
H	32.808	-16.288	-4.849
H	30.330	-13.049	-4.031
H	30.618	-15.365	-4.483
N	34.685	-12.330	-1.317
C	33.679	-12.777	-0.374
C	34.198	-12.835	1.080
O	33.707	-13.622	1.887
C	33.114	-14.112	-0.855
C	34.097	-15.263	-0.911
C	33.401	-16.573	-1.166
C	34.398	-17.703	-1.221
N	33.862	-18.807	-2.042
H	35.531	-12.865	-1.567
H	32.891	-12.138	-0.398
H	32.360	-14.370	-0.240
H	32.736	-13.974	-1.778
H	34.756	-15.092	-1.647
H	34.588	-15.315	-0.038
H	32.759	-16.750	-0.428
H	32.924	-16.526	-2.037
H	35.248	-17.371	-1.625
H	34.572	-18.032	-0.295
H	32.877	-18.681	-2.175
H	34.317	-18.816	-2.934
H	34.024	-19.681	-1.580
N	35.110	-11.920	1.464
C	35.729	-11.940	2.788
C	34.966	-11.100	3.817
O	35.273	-11.209	4.996
C	37.143	-11.442	2.678
O	37.114	-10.071	2.290
H	35.328	-11.208	0.738
H	35.733	-12.899	3.117
H	37.591	-11.512	3.555
H	37.624	-11.955	1.984
H	37.015	-10.011	1.299
N	34.042	-10.206	3.405
C	33.501	-9.215	4.347

C	31.982	-9.229	4.408
O	31.299	-8.984	3.420
C	33.980	-7.785	4.033
C	33.687	-6.785	5.140
O	35.390	-7.855	3.896
H	33.768	-10.281	2.423
H	33.861	-9.430	5.270
H	33.581	-7.497	3.151
H	32.698	-6.638	5.213
H	34.032	-7.134	6.015
H	34.134	-5.911	4.937
H	35.655	-8.646	3.329
N	31.440	-9.503	5.606
C	30.011	-9.379	5.855
C	29.714	-7.999	6.454
O	30.543	-7.443	7.158
C	29.553	-10.453	6.843
C	29.659	-11.900	6.392
C	29.452	-12.860	7.577
C	28.601	-12.160	5.342
H	32.127	-9.807	6.325
H	29.522	-9.454	4.986
H	30.105	-10.355	7.673
H	28.593	-10.274	7.061
H	30.565	-12.062	6.001
H	28.546	-12.707	7.966
H	29.527	-13.800	7.252
H	30.151	-12.683	8.266
H	28.743	-11.551	4.559
H	28.657	-13.112	5.033
H	27.692	-11.992	5.728
N	28.482	-7.531	6.236
C	27.995	-6.271	6.749
C	26.655	-6.505	7.435
O	25.839	-7.292	6.986
C	27.914	-5.210	5.629
C	26.916	-5.572	4.549
C	27.612	-3.848	6.221
H	27.890	-8.177	5.646
H	28.638	-5.936	7.438
H	28.818	-5.164	5.194
H	27.398	-5.749	3.689
H	26.410	-6.393	4.818
H	26.273	-4.816	4.417
H	26.626	-3.680	6.185
H	27.921	-3.822	7.173
H	28.090	-3.143	5.696

N	26.439	-5.800	8.541
C	25.156	-5.846	9.210
C	25.000	-4.670	10.160
O	25.714	-3.674	10.083
H	27.244	-5.237	8.866
H	24.415	-5.821	8.527
H	25.074	-6.705	9.730
N	23.973	-4.767	11.000
C	23.665	-3.677	11.923
C	23.239	-4.276	13.254
O	22.531	-5.273	13.268
C	22.590	-2.767	11.336
C	21.395	-3.483	10.812
C	20.166	-3.728	11.358
N	21.381	-4.061	9.557
C	20.178	-4.598	9.350
N	19.433	-4.415	10.422
H	23.436	-5.643	10.944
H	24.493	-3.141	12.068
H	22.286	-2.119	12.049
H	22.995	-2.229	10.582
H	22.138	-4.077	8.917
H	19.864	-3.455	12.265
H	19.893	-5.061	8.512
N	23.718	-3.704	14.358
C	23.348	-4.197	15.679
C	22.879	-2.985	16.485
O	23.495	-1.914	16.410
C	24.531	-4.825	16.416
C	25.020	-6.202	16.008
O	24.356	-6.855	15.130
O	26.104	-6.608	16.526
H	24.354	-2.906	14.198
H	22.563	-4.791	15.567
H	25.318	-4.174	16.340
H	24.290	-4.856	17.410
N	21.788	-3.143	17.249
C	21.394	-2.106	18.189
C	21.557	-2.634	19.616
O	21.033	-3.704	19.938
C	19.946	-1.655	18.021
C	19.558	-0.546	18.976
O	19.743	-1.203	16.686
H	21.283	-4.027	17.111
H	22.001	-1.319	18.076
H	19.328	-2.452	18.186
H	18.619	-0.686	19.303

H	20.179	-0.538	19.765
H	19.611	0.342	18.511
H	19.795	-0.198	16.641
N	22.239	-1.854	20.455
C	22.521	-2.243	21.836
C	21.655	-1.421	22.791
O	21.506	-0.195	22.667
C	24.009	-2.091	22.152
C	24.915	-2.938	21.310
C	25.317	-2.527	20.042
C	25.385	-4.140	21.772
C	26.155	-3.331	19.299
C	26.230	-4.943	21.026
C	26.587	-4.543	19.762
H	22.552	-0.948	20.043
H	22.235	-3.197	21.936
H	24.253	-1.140	22.006
H	24.144	-2.347	23.101
H	25.003	-1.662	19.678
H	25.107	-4.458	22.696
H	26.455	-3.002	18.376
H	26.572	-5.810	21.415
H	27.157	-5.130	19.186
N	21.083	-2.108	23.802
C	20.319	-1.416	24.834
C	20.844	-1.847	26.225
O	21.593	-2.807	26.337
C	18.812	-1.680	24.671
C	18.251	-1.263	23.328
O	18.534	-3.074	24.769
H	21.234	-3.125	23.773
H	20.465	-0.434	24.744
H	18.311	-1.199	25.425
H	17.257	-1.403	23.312
H	18.446	-0.293	23.160
H	18.668	-1.810	22.596
H	18.355	-3.332	25.726
N	20.481	-1.092	27.266
C	20.990	-1.294	28.619
C	20.151	-2.333	29.365
O	20.556	-2.798	30.426
C	21.001	0.019	29.407
C	22.019	1.067	28.965
C	22.981	1.477	30.067
C	24.045	0.415	30.275
N	24.485	0.282	31.690
H	19.794	-0.337	27.024

H	21.938	-1.626	28.566
H	20.083	0.430	29.342
H	21.179	-0.200	30.375
H	22.550	0.696	28.192
H	21.524	1.884	28.644
H	23.425	2.326	29.807
H	22.474	1.589	30.913
H	23.683	-0.475	29.959
H	24.847	0.640	29.702
H	24.420	-0.676	31.969
H	25.432	0.591	31.773
H	23.898	0.842	32.274
N	19.005	-2.693	28.778
C	18.141	-3.773	29.231
C	18.005	-4.774	28.081
O	17.968	-4.352	26.934
C	16.772	-3.202	29.663
C	16.907	-2.218	30.830
C	16.017	-2.559	28.495
H	18.774	-2.104	27.927
H	18.586	-4.238	29.989
H	16.215	-3.963	29.991
H	16.556	-1.326	30.551
H	16.383	-2.557	31.609
H	17.869	-2.137	31.082
H	15.056	-2.458	28.746
H	16.415	-1.664	28.304
H	16.099	-3.146	27.692
N	17.914	-6.081	28.365
C	17.869	-7.090	27.303
C	16.539	-6.996	26.547
O	16.538	-7.431	25.380
C	18.010	-8.527	27.821
C	18.771	-8.658	29.130
C	19.370	-10.015	29.362
C	20.114	-10.075	30.679
N	20.924	-11.313	30.782
O	15.463	-6.574	26.861
H	17.879	-6.306	29.373
H	18.617	-6.918	26.659
H	17.091	-8.900	27.952
H	18.488	-9.062	27.123
H	19.510	-7.963	29.143
H	18.140	-8.439	29.893
H	18.642	-10.697	29.379
H	20.012	-10.222	28.627
H	20.718	-9.278	30.750

H	19.451	-10.051	31.431
H	20.355	-12.104	30.556
H	21.693	-11.263	30.144
H	21.272	-11.408	31.715

Ir(*R*)-*pro-S* with Sav S112I

N	52.818	-12.396	12.098
C	52.905	-11.169	11.250
C	52.919	-9.893	12.086
O	52.623	-8.824	11.554
C	51.707	-10.963	10.328
C	51.102	-12.223	9.754
O	51.779	-12.882	8.929
O	49.953	-12.514	10.138
H	52.775	-12.346	13.100
H	53.786	-11.223	10.764
H	50.998	-10.476	10.851
H	52.002	-10.373	9.568
N	53.236	-10.020	13.380
C	53.502	-8.890	14.250
C	54.491	-7.902	13.619
O	54.220	-6.702	13.633
C	54.010	-9.428	15.582
C	54.372	-8.362	16.577
C	54.579	-8.947	17.960
O	54.985	-8.196	18.868
O	54.329	-10.164	18.116
H	53.274	-11.017	13.707
H	52.640	-8.404	14.457
H	53.291	-9.979	15.974
H	54.819	-9.962	15.399
H	55.216	-7.949	16.304
H	53.635	-7.721	16.638
N	55.624	-8.387	13.075
C	56.634	-7.502	12.493
C	56.199	-7.009	11.109
O	56.467	-5.856	10.742
C	57.981	-8.188	12.423
H	55.710	-9.417	13.107
H	56.750	-6.703	13.094
H	58.166	-8.659	13.287
H	57.982	-8.856	11.677
H	58.698	-7.509	12.258
N	55.545	-7.901	10.344
C	54.923	-7.544	9.082
C	53.898	-6.402	9.208

O	53.882	-5.502	8.374
H	55.529	-8.866	10.743
H	55.637	-7.265	8.416
H	54.455	-8.356	8.690
N	53.029	-6.446	10.227
C	51.989	-5.426	10.388
C	52.565	-4.145	10.992
O	52.186	-3.043	10.594
C	50.802	-5.929	11.230
C	50.040	-7.043	10.517
C	49.856	-4.782	11.600
C	49.166	-7.842	11.460
H	53.155	-7.244	10.872
H	51.679	-5.192	9.462
H	51.167	-6.300	12.083
H	49.470	-6.637	9.812
H	50.701	-7.656	10.097
H	49.142	-5.127	12.205
H	50.373	-4.067	12.064
H	49.444	-4.415	10.770
H	48.498	-7.234	11.886
H	48.691	-8.555	10.947
H	49.736	-8.260	12.165
N	53.436	-4.278	11.998
C	54.129	-3.130	12.569
C	54.811	-2.322	11.463
O	55.483	-2.865	10.588
C	55.097	-3.610	13.657
C	55.887	-2.483	14.296
O	54.251	-4.275	14.599
H	53.570	-5.253	12.327
H	53.455	-2.538	13.019
H	55.725	-4.281	13.263
H	55.326	-1.652	14.336
H	56.157	-2.740	15.227
H	56.712	-2.295	13.757
H	54.164	-5.250	14.365
N	54.581	-1.011	11.451
C	55.228	-0.162	10.468
C	54.389	1.069	10.141
O	53.380	1.353	10.805
H	53.929	-0.672	12.173
H	56.129	0.136	10.817
H	55.388	-0.687	9.619
N	54.826	1.773	9.092
C	54.168	2.951	8.580
C	53.554	2.601	7.230
O	54.230	2.144	6.313
C	55.109	4.154	8.472
C	54.417	5.325	7.819
O	55.552	4.516	9.784
H	55.710	1.380	8.668
H	53.431	3.210	9.200
H	55.925	3.889	7.923
H	53.435	5.137	7.722
H	54.534	6.152	8.377
H	54.805	5.493	6.908
H	54.797	4.437	10.443
N	52.237	2.826	7.132
C	51.454	2.462	5.967
C	50.778	3.706	5.433
O	50.520	4.634	6.205
C	50.394	1.423	6.313
C	50.851	0.121	6.862
C	51.169	-0.170	8.163
C	51.022	-1.082	6.123
C	51.442	-2.062	7.043
C	50.832	-1.433	4.781
N	51.514	-1.477	8.270
C	51.662	-3.382	6.662
C	51.094	-2.727	4.402
C	51.497	-3.686	5.330
H	51.826	3.291	7.978
H	52.083	2.131	5.264
H	49.760	1.826	6.992
H	49.857	1.222	5.478
H	51.147	0.489	8.913
H	51.782	-1.946	9.119
H	50.514	-0.761	4.118
H	51.929	-4.072	7.336
H	50.994	-2.984	3.441
H	51.670	-4.623	5.014
N	50.518	3.702	4.121
C	49.794	4.758	3.441
C	48.658	4.155	2.603
O	48.779	3.118	1.955
C	50.734	5.535	2.482
C	52.018	5.999	3.115
C	52.065	7.214	3.782
C	53.170	5.215	3.102
C	53.214	7.646	4.413
C	54.338	5.640	3.727
C	54.355	6.861	4.370
O	55.431	7.398	4.979
H	50.892	2.852	3.623
H	49.381	5.361	4.127
H	50.952	4.931	1.719
H	50.234	6.332	2.151
H	51.243	7.795	3.806
H	53.157	4.332	2.637
H	53.226	8.521	4.901
H	55.154	5.066	3.708
H	55.697	6.832	5.763
N	47.532	4.859	2.540
C	46.474	4.424	1.644
C	46.528	5.230	0.351
O	47.371	6.121	0.199
C	45.092	4.462	2.318
C	44.592	5.838	2.657
H	47.484	5.683	3.143
H	46.660	3.469	1.401
H	44.435	4.020	1.697
H	45.148	3.918	3.163
N	43.493	5.908	3.419
O	45.111	6.825	2.133
H	43.060	5.063	3.732
H	43.116	6.793	3.669
N	45.554	4.962	-0.531
C	45.460	5.559	-1.861
C	45.114	7.047	-1.803
O	45.287	7.763	-2.794
C	44.483	4.743	-2.726
C	42.984	4.847	-2.354
C	42.543	4.298	-1.003
H	44.846	4.267	-0.171
H	46.364	5.472	-2.306
H	44.576	5.039	-3.686
H	44.739	3.768	-2.672
H	42.722	5.831	-2.401
H	42.452	4.372	-3.083
N	41.440	4.843	-0.477
O	43.123	3.361	-0.450
H	41.080	4.518	0.413
H	40.974	5.587	-0.987
N	44.668	7.544	-0.645
C	44.414	8.965	-0.447
C	45.660	9.683	0.052
O	45.621	10.903	0.201
C	43.308	9.146	0.597
C	41.930	8.594	0.238
C	40.999	8.691	1.446
C	41.372	9.322	-0.968
H	44.519	6.827	0.099
H	44.146	9.370	-1.330
H	43.603	8.689	1.435
H	43.204	10.124	0.764
H	42.020	7.639	-0.032
H	40.910	9.648	1.720
H	40.101	8.328	1.202
H	41.381	8.160	2.202
H	41.986	9.200	-1.750
H	40.469	8.954	-1.197
H	41.288	10.299	-0.764
N	46.672	8.920	0.444
C	47.876	9.487	1.035
C	47.785	9.698	2.550
O	48.630	10.360	3.148
H	46.533	7.900	0.295
H	48.668	8.878	0.836
H	48.081	10.381	0.592
N	46.796	9.075	3.198
C	46.729	9.077	4.653
C	47.850	8.223	5.214
O	48.216	7.254	4.566
C	45.365	8.560	5.097
O	44.351	9.401	4.559
H	46.104	8.606	2.589
H	46.866	10.018	4.970
H	45.238	7.643	4.753
H	45.314	8.587	6.083
H	44.309	9.276	3.570
N	48.262	8.512	6.459
C	49.386	7.830	7.092
C	48.897	7.069	8.325
O	48.239	7.661	9.164
C	50.479	8.814	7.531
C	51.702	8.075	8.036
O	50.808	9.628	6.399
H	47.709	9.262	6.926
H	49.766	7.167	6.444
H	50.109	9.415	8.258
H	52.384	8.730	8.371
H	51.445	7.459	8.786
H	52.107	7.534	7.294
H	50.055	9.621	5.732
N	49.149	5.764	8.335
C	48.728	4.832	9.377
C	50.002	4.269	10.006
O	50.679	3.443	9.386
C	47.872	3.763	8.699
C	47.483	2.534	9.467
C	47.073	2.612	10.782
C	47.483	1.294	8.854
C	46.658	1.480	11.469
C	47.099	0.158	9.543
C	46.675	0.249	10.850
H	49.705	5.441	7.488
H	48.274	5.363	10.079
H	47.018	4.212	8.390
H	48.370	3.459	7.871
H	47.071	3.493	11.253
H	47.765	1.217	7.893
H	46.346	1.570	12.426
H	47.134	-0.745	9.075
H	46.382	-0.566	11.347
N	50.306	4.699	11.234
C	51.496	4.232	11.931
C	51.040	3.280	13.024
O	50.340	3.697	13.935
C	52.303	5.404	12.520
C	52.753	6.388	11.440
C	53.478	4.911	13.368
C	53.410	7.627	11.984
H	49.633	5.379	11.634
H	52.011	3.690	11.277
H	51.709	5.895	13.158
H	53.392	5.917	10.833
H	51.948	6.654	10.908
H	53.912	5.690	13.814
H	53.142	4.274	14.058
H	54.139	4.451	12.780
H	54.222	7.373	12.510
H	53.678	8.224	11.228
H	52.769	8.113	12.579
N	51.398	2.015	12.898
C	50.845	1.010	13.786
C	51.927	0.111	14.378
O	52.934	-0.214	13.746
C	49.783	0.174	13.059
C	50.370	-0.675	11.944
C	48.978	-0.665	14.052
H	52.074	1.815	12.147
H	50.384	1.482	14.553
H	49.134	0.810	12.631
H	50.212	-0.228	11.063

H	51.354	-0.785	12.088
H	49.933	-1.575	11.942
H	48.756	-1.544	13.632
H	49.525	-0.811	14.875
H	48.138	-0.178	14.285
N	51.637	-0.324	15.602
C	52.376	-1.355	16.314
C	51.425	-2.508	16.658
O	50.411	-2.306	17.320
C	52.966	-0.793	17.613
C	53.672	-1.834	18.457
O	53.882	0.218	17.214
H	50.794	0.161	16.027
H	53.093	-1.712	15.716
H	52.228	-0.366	18.149
H	54.458	-1.421	18.926
H	53.041	-2.205	19.144
H	53.997	-2.585	17.875
H	53.848	0.355	16.216
N	51.754	-3.701	16.176
C	51.083	-4.921	16.573
C	51.803	-5.531	17.783
O	53.021	-5.663	17.753
C	51.082	-5.840	15.408
H	52.542	-3.679	15.484
H	50.142	-4.710	16.842
H	50.227	-5.747	14.885
H	51.853	-5.634	14.795
H	51.165	-6.796	15.712
N	51.059	-5.826	18.848
C	51.582	-6.494	20.033
C	51.378	-8.005	19.956
O	50.669	-8.501	19.076
H	50.054	-5.533	18.758
H	52.567	-6.297	20.124
H	51.116	-6.139	20.854
N	52.030	-8.746	20.865
C	52.022	-10.207	20.826
C	50.621	-10.758	21.119
O	50.269	-11.855	20.692
C	53.032	-10.740	21.825
H	52.533	-8.194	21.587
H	52.296	-10.510	19.906
H	52.757	-11.653	22.127
H	53.934	-10.792	21.396
H	53.077	-10.130	22.616
N	49.845	-9.947	21.853
C	48.563	-10.268	22.464
C	47.367	-9.939	21.557
O	46.240	-9.923	22.046
C	48.430	-9.428	23.743
C	48.324	-7.919	23.519
O	49.009	-7.381	22.603
O	47.549	-7.282	24.268
H	50.283	-8.968	21.959
H	48.543	-11.270	22.617
H	47.608	-9.733	24.227
H	49.233	-9.607	24.313
N	47.621	-9.605	20.280
C	46.563	-9.256	19.341
C	46.193	-7.768	19.328
O	45.273	-7.395	18.626
H	48.632	-9.619	20.030
H	46.844	-9.531	18.399
H	45.727	-9.799	19.558
N	46.898	-6.901	20.066
C	46.575	-5.475	20.110
C	47.160	-4.746	18.906
O	48.247	-5.067	18.460
C	47.110	-4.835	21.358
H	47.684	-7.327	20.597
H	45.574	-5.370	20.094
H	47.998	-5.236	21.598
H	47.228	-3.849	21.216
H	46.473	-4.979	22.120
N	46.434	-3.741	18.397
C	46.984	-2.748	17.477
C	46.916	-1.399	18.190
O	45.907	-1.074	18.801
C	46.165	-2.702	16.172
C	46.093	-3.993	15.360
C	45.229	-3.809	14.122
C	47.477	-4.444	14.909
H	45.438	-3.732	18.718
H	47.934	-2.964	17.302
H	45.231	-2.446	16.414
H	46.569	-2.001	15.589
H	45.703	-4.717	15.932
H	45.618	-3.089	13.546
H	45.196	-4.666	13.606
H	44.302	-3.550	14.396
H	48.053	-4.605	15.711
H	47.397	-5.290	14.381
H	47.890	-3.733	14.339
N	47.993	-0.616	18.141
C	47.994	0.721	18.721
C	48.772	1.629	17.789
O	49.658	1.177	17.065
C	48.651	0.696	20.112
C	47.919	-0.183	21.098
O	49.983	0.201	19.938
H	48.814	-1.035	17.663
H	47.055	1.047	18.773
H	48.705	1.641	20.466
H	48.549	-0.860	21.492
H	47.537	0.375	21.841
H	47.169	-0.668	20.638
H	50.015	-0.460	19.181
N	48.480	2.915	17.801
C	49.261	3.804	16.958
C	48.546	5.105	16.650
O	47.756	5.589	17.446
H	47.717	3.206	18.407
H	50.146	4.009	17.415
H	49.484	3.331	16.086
N	48.874	5.673	15.484
C	48.353	6.972	15.107
C	47.919	6.935	13.641
O	48.444	6.204	12.786
C	49.339	8.133	15.347
C	49.787	8.291	16.787
O	50.454	7.996	14.466
H	49.516	5.111	14.896
H	47.547	7.174	15.665
H	48.867	9.011	15.134
H	49.761	9.259	17.050
H	49.179	7.772	17.394
H	50.724	7.949	16.894
H	50.400	8.671	13.721
N	46.934	7.767	13.363
C	46.378	7.884	12.025
C	46.301	9.360	11.659
O	45.825	10.189	12.434
C	44.967	7.287	11.945
C	44.518	6.967	10.541
C	44.021	7.943	9.689
C	44.616	5.678	10.066
C	43.616	7.636	8.395
C	44.251	5.354	8.776
C	43.734	6.334	7.953
O	43.416	6.059	6.676
H	46.592	8.327	14.174
H	46.997	7.435	11.382
H	44.956	6.445	12.479
H	44.329	7.946	12.336
H	43.941	8.966	10.037
H	44.963	4.954	10.672
H	43.224	8.404	7.742
H	44.359	4.419	8.438
H	43.708	6.812	6.080
N	46.751	9.668	10.449
C	46.693	11.025	9.937
C	45.981	10.998	8.597
O	46.452	10.366	7.659
C	48.090	11.602	9.781
C	48.073	12.985	9.174
C	49.412	13.707	9.205
O	49.449	14.842	8.697
O	50.399	13.148	9.742
H	47.140	8.867	9.912
H	46.192	11.608	10.572
H	48.489	11.670	10.672
H	48.593	11.016	9.180
H	47.804	12.907	8.223
H	47.428	13.543	9.679
N	44.780	11.747	8.496
C	43.863	11.633	7.379
C	44.153	12.733	6.407
O	43.947	13.934	6.727
C	42.433	11.703	7.884
O	41.557	11.698	6.798
H	44.688	12.573	9.132
H	43.991	10.646	6.888
H	42.233	10.805	8.502
H	42.294	12.598	8.537
H	41.031	12.535	6.867
N	44.631	12.408	5.107
C	44.898	13.418	4.081
C	43.620	14.105	3.588
O	43.715	15.049	2.816
C	45.653	12.787	2.929
H	44.539	11.394	4.898
H	45.508	14.124	4.473
H	46.123	13.498	2.404
H	46.328	12.138	3.283
H	45.013	12.304	2.330
N	42.367	13.747	4.034
C	41.113	14.385	3.559
C	40.179	14.788	4.682
O	40.440	14.491	5.873
C	40.380	13.457	2.564
C	41.180	13.310	1.274
C	40.061	12.081	3.156
H	42.280	12.937	4.678
H	41.352	15.337	3.036
H	39.418	13.933	2.282
H	41.542	14.210	1.028
H	41.941	12.668	1.386
H	40.580	12.985	0.541
H	39.524	11.464	2.411
H	40.985	11.531	3.431
H	39.417	12.182	4.052
N	39.037	15.499	4.414
C	37.995	15.744	5.431
C	37.475	17.157	5.389
O	36.339	17.370	4.879
H	38.876	15.812	3.433
H	37.148	15.056	5.246
H	38.349	15.528	6.461
N	38.174	18.212	5.907
C	39.588	18.159	6.330
C	39.720	17.862	7.821
O	39.118	18.593	8.656
C	40.277	19.492	5.949
C	41.771	19.408	5.980
H	37.659	19.118	5.980
H	40.110	17.352	5.777
H	39.989	19.774	4.916
H	39.949	20.297	6.639
N	42.490	20.445	5.765
O	42.340	18.317	6.178
H	42.072	21.359	5.511
H	43.525	20.307	5.743
N	40.527	16.771	8.280
C	40.931	16.613	9.688
C	42.189	17.431	9.985
O	42.437	18.439	9.269
C	41.129	15.115	9.956
H	41.137	16.309	7.564
H	40.124	16.975	10.360
H	42.086	14.766	9.510
H	41.129	14.904	11.043
H	40.297	14.535	9.509
N	42.994	17.169	11.065
C	44.294	17.844	11.257

C	45.268	17.046	12.154
O	44.914	16.802	13.311
C	44.084	19.260	11.813
C	45.355	20.094	11.646
C	45.192	21.525	12.135
O	46.244	22.189	12.269
O	44.101	21.958	12.605
H	42.680	16.486	11.780
H	44.750	17.965	10.249
H	43.255	19.759	11.273
H	43.809	19.201	12.887
H	46.059	19.669	12.208
H	45.655	20.140	10.698
N	46.461	16.693	11.641
C	47.503	16.055	12.419
C	47.080	14.610	12.768
O	46.272	14.000	12.060
C	47.849	16.906	13.634
O	49.075	16.488	14.218
H	46.561	16.925	10.623
H	48.331	15.995	11.861
H	47.936	17.857	13.351
H	47.124	16.815	14.311
H	48.902	16.141	15.138
N	47.670	14.053	13.826
C	47.549	12.633	14.147
C	46.443	12.378	15.171
O	46.229	13.154	16.110
C	48.873	12.129	14.726
C	49.999	12.061	13.717
C	51.325	11.787	14.415
N	52.372	11.637	13.427
C	53.629	11.278	13.694
N	54.438	10.941	12.700
N	54.042	11.188	14.951
H	48.227	14.722	14.407
H	47.326	12.127	13.303
H	49.146	12.747	15.458
H	48.724	11.214	15.089
H	49.831	11.323	13.074
H	50.076	12.929	13.241
H	51.525	12.556	15.003
H	51.229	10.951	14.933
H	52.140	11.817	12.457
H	54.140	10.967	11.752
H	55.384	10.651	12.909
H	53.408	11.411	15.697
H	54.975	10.903	15.156
N	45.791	11.220	15.033
C	44.735	10.772	15.933
C	45.135	9.401	16.491
O	45.743	8.586	15.790
C	43.402	10.671	15.190
C	42.901	12.027	14.776
C	43.341	12.621	13.577
C	42.014	12.692	15.547
C	42.841	13.820	13.210
C	41.542	13.895	15.153
C	41.930	14.448	13.983
O	41.372	15.648	13.546
H	46.111	10.652	14.206
H	44.674	11.409	16.696
H	43.509	10.018	14.296
H	42.658	10.194	15.863
H	44.067	12.120	12.945
H	41.662	12.252	16.475
H	43.176	14.277	12.291
H	40.840	14.422	15.772
H	40.684	16.023	14.192
N	44.755	9.129	17.747
C	44.974	7.824	18.346
C	44.115	6.785	17.621
O	42.969	7.072	17.320
C	44.640	7.838	19.845
C	44.763	6.437	20.420
C	45.537	8.819	20.616
H	44.300	9.922	18.239
H	45.933	7.571	18.216
H	43.689	8.110	19.942
H	44.008	5.871	20.088
H	45.632	6.032	20.134
H	44.731	6.480	21.419
H	45.376	8.704	21.593
H	46.491	8.621	20.401
H	45.310	9.750	20.339
N	44.695	5.610	17.364
C	43.961	4.448	16.906
C	44.208	3.277	17.849
O	45.268	3.171	18.452
C	44.336	4.052	15.477
C	45.701	3.404	15.250
C	45.691	1.865	15.362
C	46.209	3.811	13.879
H	45.732	5.607	17.525
H	42.980	4.658	16.915
H	43.642	3.405	15.148
H	44.302	4.880	14.912
H	46.358	3.771	15.927
H	45.067	1.495	14.679
H	46.613	1.523	15.202
H	45.387	1.609	16.276
H	46.295	4.808	13.834
H	47.104	3.394	13.714
H	45.566	3.502	13.176
N	43.186	2.427	17.987
C	43.371	1.154	18.659
C	42.614	0.105	17.860
O	41.610	0.442	17.224
C	42.960	1.288	20.123
C	41.485	1.489	20.315
O	43.335	0.101	20.818
H	42.292	2.750	17.587
H	44.338	0.921	18.648
H	43.461	2.073	20.539
H	41.314	2.110	21.088
H	41.075	1.888	19.487
H	41.033	0.610	20.504
H	44.083	-0.369	20.336
N	43.070	-1.151	17.937
C	42.339	-2.259	17.355
C	42.908	-3.609	17.742
O	43.613	-3.712	18.749
H	43.972	-1.251	18.440
H	41.366	-2.211	17.646
H	42.346	-2.172	16.342
N	42.556	-4.613	16.929
C	42.869	-6.013	17.174
C	43.241	-6.673	15.861
O	42.712	-6.287	14.822
C	41.660	-6.730	17.784
C	41.144	-6.133	19.079
C	42.040	-6.214	20.325
N	42.267	-7.599	20.690
C	43.223	-8.050	21.499
N	44.023	-7.218	22.133
N	43.366	-9.348	21.677
H	42.021	-4.291	16.078
H	43.650	-6.067	17.797
H	40.918	-6.709	17.114
H	41.920	-7.679	17.961
H	40.927	-5.142	18.930
H	40.253	-6.577	19.323
H	42.899	-5.777	20.105
H	41.574	-5.742	21.059
H	41.638	-8.298	20.292
H	43.917	-6.228	22.036
H	44.754	-7.576	22.727
H	42.746	-9.993	21.222
H	44.094	-9.701	22.269
N	44.104	-7.704	15.920
C	44.380	-8.573	14.793
C	44.472	-10.013	15.326
O	44.679	-10.219	16.533
C	45.669	-8.157	14.062
C	46.946	-8.517	14.790
C	47.359	-7.840	15.931
C	47.765	-9.531	14.299
C	48.509	-8.213	16.612
C	48.951	-9.869	14.926
C	49.314	-9.224	16.093
O	50.488	-9.591	16.666
H	44.556	-7.816	16.859
H	43.616	-8.533	14.158
H	45.678	-8.607	13.171
H	45.648	-7.166	13.939
H	46.818	-7.067	16.270
H	47.486	-10.026	13.474
H	48.760	-7.763	17.468
H	49.543	-10.576	14.537
H	50.553	-9.196	17.584
N	44.303	-10.946	14.413
C	44.489	-12.380	14.630
C	45.985	-12.694	14.714
O	46.681	-12.738	13.692
C	43.819	-13.170	13.507
C	43.860	-14.692	13.643
O	44.353	-15.177	14.678
O	43.446	-15.356	12.661
H	44.008	-10.563	13.465
H	44.094	-12.600	15.522
H	42.855	-12.886	13.460
H	44.267	-12.920	12.642
N	46.443	-12.928	15.953
C	47.855	-13.174	16.245
C	48.274	-14.629	15.987
O	49.450	-14.939	16.070
C	48.191	-12.728	17.651
O	47.310	-13.283	18.606
H	45.701	-12.919	16.689
H	48.408	-12.584	15.635
H	49.125	-13.013	17.871
H	48.131	-11.729	17.700
H	46.571	-13.768	18.139
N	47.341	-15.510	15.615
C	47.674	-16.877	15.248
C	46.910	-17.280	13.987
O	46.062	-18.164	13.999
C	47.344	-17.783	16.400
H	46.371	-15.133	15.612
H	48.657	-16.935	15.056
H	48.085	-18.444	16.541
H	47.225	-17.245	17.238
H	46.495	-18.283	16.212
N	47.182	-16.656	12.837
C	46.347	-16.885	11.649
C	46.567	-18.242	10.962
O	47.527	-18.944	11.266
C	46.779	-15.741	10.729
C	48.241	-15.524	11.104
C	48.319	-15.760	12.603
H	45.359	-16.765	11.881
H	46.704	-16.001	9.772
H	46.252	-14.917	10.906
H	48.821	-16.172	10.609
H	48.517	-14.590	10.874
H	49.177	-16.199	12.851
H	48.211	-14.907	13.106
N	45.680	-18.572	10.009
C	45.725	-19.819	9.259
C	46.976	-19.865	8.387
O	47.517	-18.837	7.972
C	44.469	-19.958	8.427
H	44.943	-17.838	9.854
H	45.758	-20.588	9.905
H	44.673	-20.464	7.587
H	43.771	-20.453	8.947
H	44.119	-19.051	8.187
N	47.419	-21.086	8.086
C	48.684	-21.300	7.398
C	48.452	-21.741	5.948
O	49.325	-22.375	5.357
C	49.502	-22.331	8.191
C	49.987	-21.832	9.538
O	48.611	-23.438	8.347
H	46.789	-21.859	8.383

H	49.194	-20.433	7.382
H	50.282	-22.624	7.637
H	49.542	-20.960	9.761
H	49.767	-22.502	10.251
H	50.981	-21.695	9.515
H	47.802	-23.324	7.759
N	47.303	-21.368	5.360
C	46.861	-21.858	4.059
C	46.978	-20.781	2.967
O	46.443	-20.941	1.867
C	45.437	-22.407	4.173
C	44.406	-21.355	4.560
O	44.807	-20.319	5.157
O	43.219	-21.551	4.225
H	46.745	-20.683	5.930
H	47.481	-22.609	3.792
H	45.182	-22.790	3.289
H	45.436	-23.122	4.867
N	47.700	-19.685	3.251
C	47.831	-18.575	2.315
C	46.742	-17.510	2.507
O	46.689	-16.558	1.742
H	48.151	-19.703	4.189
H	48.738	-18.140	2.431
H	47.782	-18.925	1.366
N	45.878	-17.695	3.513
C	44.876	-16.723	3.941
C	45.526	-15.471	4.516
O	46.602	-15.523	5.092
C	43.938	-17.316	4.966
O	42.944	-18.093	4.327
H	45.988	-18.637	3.986
H	44.340	-16.450	3.131
H	44.454	-17.899	5.585
H	43.496	-16.581	5.470
H	43.176	-19.062	4.404
N	44.828	-14.334	4.424
C	45.295	-13.137	5.081
C	45.004	-13.164	6.579
O	44.335	-14.066	7.077
H	43.965	-14.397	3.864
H	46.292	-13.036	4.943
H	44.847	-12.326	4.675
N	45.539	-12.150	7.268
C	45.388	-11.968	8.702
C	44.297	-10.930	8.956
O	44.499	-9.770	8.571
C	46.722	-11.549	9.331
C	46.624	-11.325	10.816
O	47.614	-12.619	8.965
H	46.091	-11.482	6.665
H	45.098	-12.833	9.109
H	47.042	-10.711	8.895
H	45.921	-10.635	11.019
H	46.378	-12.181	11.282
H	47.505	-11.003	11.179
H	48.475	-12.542	9.476
N	43.222	-11.378	9.603
C	42.056	-10.544	9.902
C	42.403	-9.527	10.979
O	43.156	-9.813	11.906
C	40.873	-11.381	10.291
H	43.281	-12.387	9.883
H	41.789	-10.053	9.068
H	40.972	-11.700	11.237
H	40.030	-10.842	10.218
H	40.801	-12.178	9.686
N	41.949	-8.278	10.772
C	42.239	-7.220	11.719
C	41.218	-6.097	11.562
O	40.455	-6.030	10.593
C	43.683	-6.719	11.534
C	44.114	-6.243	10.144
C	43.345	-5.010	9.715
C	45.609	-5.953	10.142
H	41.398	-8.157	9.909
H	42.164	-7.585	12.649
H	43.818	-5.951	12.167
H	44.298	-7.468	11.797
H	43.954	-6.984	9.485
H	43.508	-4.267	10.366
H	43.648	-4.723	8.805
H	42.365	-5.216	9.687
H	46.111	-6.786	10.378
H	45.888	-5.642	9.233
H	45.812	-5.240	10.814
N	41.232	-5.210	12.538
C	40.458	-3.988	12.424
C	40.962	-2.969	13.418
O	41.708	-3.317	14.326
H	41.815	-5.451	13.353
H	40.543	-3.621	11.493
H	39.490	-4.185	12.605
N	40.591	-1.706	13.206
C	40.967	-0.628	14.109
C	39.965	0.497	13.996
O	39.204	0.584	13.020
C	42.417	-0.178	13.888
C	42.670	0.656	12.659
C	42.581	2.002	12.492
C	43.037	0.118	11.379
C	43.143	1.193	10.490
C	43.244	-1.186	10.913
N	42.860	2.330	11.190
C	43.475	1.013	9.145
C	43.618	-1.353	9.602
C	43.692	-0.263	8.732
H	40.017	-1.563	12.349
H	40.925	-0.985	15.045
H	42.709	0.367	14.676
H	42.999	-0.990	13.815
H	42.347	2.658	13.208
H	42.857	3.259	10.812
H	43.124	-1.969	11.515
H	43.543	1.802	8.532
H	43.839	-2.266	9.268
H	43.916	-0.443	7.764
N	39.957	1.352	15.024
C	39.129	2.527	15.114
C	39.938	3.805	15.359
O	40.902	3.805	16.137
C	38.157	2.388	16.297
C	37.178	3.536	16.418
O	37.431	1.166	16.186
H	40.640	1.074	15.791
H	38.625	2.644	14.240
H	38.697	2.363	17.165
H	37.668	4.385	16.636
H	36.684	3.659	15.553
H	36.515	3.347	17.147
H	36.710	1.119	16.887
N	39.474	4.886	14.712
C	39.870	6.246	15.002
C	38.662	7.003	15.299
O	37.791	7.225	14.434
C	40.600	6.917	13.817
C	40.897	8.396	14.092
C	41.910	6.200	13.554
H	38.776	4.642	13.954
H	40.470	6.237	15.802
H	39.973	6.862	12.899
H	41.579	8.814	13.324
H	39.965	8.993	14.039
H	41.361	8.508	15.092
H	42.415	6.079	14.411
H	41.723	5.303	13.149
H	42.460	6.744	12.918
N	38.554	7.567	16.558
C	37.753	8.776	16.773
C	38.474	9.972	16.186
O	39.692	10.150	16.470
C	37.494	8.965	18.272
H	39.399	7.451	17.158
H	36.761	8.663	16.284
H	36.866	9.868	18.441
H	36.940	8.090	18.674
H	38.447	9.081	18.829
N	37.814	10.897	15.429
C	38.443	12.113	14.880
C	38.385	13.246	15.876
O	38.279	14.437	15.468
C	37.762	12.511	13.560
C	37.826	11.396	12.572
C	36.796	10.638	12.184
C	38.949	10.914	11.931
C	38.525	9.854	11.188
C	40.289	11.310	11.932
N	37.202	9.682	11.319
C	39.413	9.100	10.435
C	41.159	10.588	11.186
C	40.715	9.465	10.435
H	36.798	10.758	15.277
H	39.516	11.917	14.673
H	36.698	12.760	13.762
H	38.254	13.406	13.119
H	35.773	10.754	12.524
H	36.617	8.968	10.841
H	40.620	12.156	12.518
H	39.065	8.239	9.887
H	42.210	10.855	11.180
H	41.427	8.889	9.860
N	38.810	13.013	17.221
C	39.111	14.071	18.180
C	40.581	14.036	18.565
O	41.073	12.983	18.953
C	38.180	13.906	19.384
C	38.444	14.976	20.432
C	37.541	14.807	21.643
C	37.941	15.746	22.794
N	39.262	15.434	23.368
H	38.854	12.038	17.574
H	38.874	15.059	17.730
H	37.130	13.996	19.035
H	38.313	12.894	19.829
H	39.471	14.839	20.766
H	38.340	15.987	19.990
H	36.493	15.028	21.343
H	37.588	13.750	21.965
H	37.912	16.805	22.442
H	37.241	15.669	23.501
H	39.974	15.574	22.676
H	39.441	16.033	24.151
H	39.282	14.480	23.673
N	41.291	15.172	18.428
C	42.614	15.339	19.017
C	42.666	16.759	19.609
O	41.649	17.411	19.742
C	43.705	15.003	17.987
C	43.755	15.951	16.798
H	40.861	15.970	17.907
H	42.685	14.710	19.779
H	44.592	15.030	18.452
H	43.538	14.075	17.647
N	44.481	15.561	15.761
O	42.812	16.733	16.574
H	44.956	14.678	15.787
H	44.557	16.143	14.951
N	43.867	17.255	19.886
C	44.049	18.608	20.402
C	43.777	19.709	19.375
O	43.628	20.864	19.750
C	45.467	18.797	20.932
C	45.703	17.996	22.188
H	44.659	16.602	19.705
H	43.374	18.735	21.153
H	46.106	18.495	20.234
H	45.600	19.760	21.136
N	46.902	17.451	22.323
O	44.792	17.833	22.999
H	47.598	17.586	21.615
H	47.118	16.903	23.133
N	43.487	19.451	18.066
C	43.420	20.488	17.008
C	41.998	20.694	16.528
O	41.440	21.818	16.639

C	44.333	20.101	15.816
C	45.796	20.061	16.189
C	46.270	19.113	17.085
C	46.686	21.006	15.701
C	47.599	19.075	17.461
C	48.027	20.975	16.057
C	48.472	20.018	16.956
O	49.773	19.941	17.353
H	43.389	18.475	17.785
H	43.772	21.472	17.396
H	44.049	19.106	15.411
H	44.176	20.837	14.997
H	45.631	18.438	17.471
H	46.359	21.719	15.083
H	47.927	18.370	18.092
H	48.667	21.636	15.670
H	50.267	19.304	16.760
N	41.295	19.630	15.878
C	40.198	19.845	14.919
C	38.992	19.045	15.355
O	38.675	17.993	14.741
C	40.732	19.482	13.514
C	39.793	19.819	12.345
C	39.763	21.312	11.960
N	41.095	21.848	11.631
C	41.675	21.853	10.417
N	41.222	21.102	9.426
N	42.722	22.626	10.185
H	41.741	18.690	15.912
H	39.894	20.914	14.918
H	41.699	20.003	13.361
H	40.962	18.395	13.482
H	40.123	19.225	11.470
H	38.760	19.486	12.577
H	39.065	21.457	11.106
H	39.335	21.896	12.805
H	41.653	22.278	12.405
H	40.518	20.370	9.612
H	41.857	20.904	8.631
H	43.134	23.193	10.956
H	43.276	22.546	9.316
N	38.334	19.370	16.585
C	37.360	18.470	17.236
C	36.184	18.195	16.314
O	35.373	19.122	16.059
C	36.882	19.091	18.576
C	36.219	18.096	19.476
H	38.704	20.204	17.094
H	37.873	17.507	17.455
H	37.761	19.493	19.124
H	36.189	19.941	18.400
N	35.098	17.554	19.178
O	36.777	17.766	20.543
H	34.527	17.881	18.376
H	34.718	16.818	19.820
N	35.906	16.879	15.833
C	35.028	16.660	14.672
C	33.781	15.853	14.999
O	32.951	15.654	14.071
C	35.838	16.000	13.557
H	36.524	16.099	16.154
H	34.662	17.634	14.276
H	36.209	15.012	13.890
H	35.209	15.864	12.654
H	36.710	16.637	13.294
N	33.552	15.304	16.239
C	32.307	14.591	16.610
C	31.991	13.466	15.641
O	30.911	13.462	15.001
C	31.136	15.585	16.758
C	31.440	16.660	17.761
H	34.280	15.400	16.983
H	32.452	14.097	17.595
H	30.909	16.042	15.771
H	30.217	15.049	17.085
C	31.615	17.945	17.499
N	31.526	16.470	19.091
C	31.742	17.705	19.589
N	31.804	18.598	18.658
H	31.433	15.575	19.629
H	31.598	18.415	16.527
H	31.839	17.934	20.649
N	32.968	12.468	15.357
C	32.780	11.401	14.343
C	33.750	10.253	14.556
O	34.601	10.329	15.476
C	32.891	11.968	12.923
O	34.126	12.596	12.710
H	33.868	12.548	15.883
H	31.757	10.990	14.435
H	32.768	11.149	12.186
H	32.060	12.687	12.743
H	34.081	13.454	13.215
N	33.694	9.049	13.781
C	34.559	7.904	14.115
C	34.628	6.891	12.983
O	33.573	6.313	12.612
C	34.052	7.231	15.400
H	32.823	8.866	13.244
H	35.583	8.280	14.300
H	33.014	6.861	15.259
H	34.705	6.373	15.670
H	34.055	7.945	16.251
N	35.814	6.580	12.372
C	35.965	5.466	11.414
C	36.358	4.154	12.080
O	37.327	4.056	12.896
C	36.937	5.819	10.277
C	37.027	4.714	9.221
O	36.515	7.005	9.661
H	36.637	7.188	12.544
H	34.988	5.314	10.921
H	37.939	6.000	10.711
H	37.496	3.808	9.640
H	36.015	4.457	8.846
H	37.652	5.055	8.371
H	35.792	6.768	9.025
N	35.792	3.018	11.598
C	36.395	1.688	11.772
C	36.767	1.097	10.416
O	35.998	1.178	9.468
C	35.460	0.716	12.505
C	35.046	1.301	13.830
O	34.306	0.461	11.679
H	35.029	3.121	10.905
H	37.232	1.776	12.313
H	35.944	-0.168	12.648
H	33.955	1.412	13.898
H	35.315	0.688	14.580
H	35.487	2.193	13.969
H	34.173	1.139	10.979
N	37.962	0.529	10.390
C	38.476	-0.258	9.302
C	38.453	-1.718	9.706
O	39.006	-2.077	10.733
C	39.898	0.170	8.926
C	40.066	1.552	8.366
C	40.303	1.819	7.046
C	40.191	2.818	9.049
C	40.433	3.799	8.063
C	40.032	3.261	10.368
N	40.498	3.158	6.870
C	40.577	5.158	8.366
C	40.202	4.592	10.678
C	40.504	5.530	9.663
H	38.527	0.713	11.275
H	37.861	-0.152	8.519
H	40.478	0.113	9.746
H	40.256	-0.472	8.238
H	40.329	1.133	6.321
H	40.666	3.606	5.979
H	39.791	2.604	11.094
H	40.820	5.870	7.593
H	40.116	4.882	11.632
H	40.686	6.559	9.934
N	37.946	-2.551	8.792
C	37.927	-3.999	8.925
C	38.548	-4.585	7.657
O	38.188	-4.212	6.539
C	36.495	-4.439	9.121
O	36.416	-5.835	9.273
H	37.552	-2.053	7.947
H	38.505	-4.253	9.692
H	36.128	-4.002	9.939
H	35.959	-4.168	8.324
H	35.552	-6.079	9.715
N	39.552	-5.453	7.820
C	40.261	-5.989	6.668
C	41.249	-7.090	7.021
O	41.120	-7.798	8.030
H	39.766	-5.696	8.798
H	39.586	-6.351	5.997
H	40.758	-5.234	6.199
N	42.214	-7.260	6.122
C	43.178	-8.320	6.300
C	44.521	-7.869	5.737
O	44.614	-7.132	4.754
C	42.623	-9.644	5.754
C	42.310	-9.702	4.276
C	41.746	-11.042	3.855
H	42.206	-6.601	5.334
H	43.299	-8.479	7.281
H	43.294	-10.377	5.964
H	41.773	-9.864	6.265
H	41.639	-8.993	4.059
H	43.150	-9.534	3.760
N	40.735	-11.006	3.000
O	42.220	-12.103	4.277
H	40.308	-11.869	2.657
H	40.384	-10.104	2.686
N	45.557	-8.288	6.462
C	46.941	-8.134	6.065
C	47.325	-9.311	5.156
O	47.067	-10.462	5.486
C	47.779	-8.063	7.346
C	49.250	-8.284	7.128
C	50.050	-7.236	6.699
C	49.841	-9.523	7.343
C	51.407	-7.415	6.489
C	51.203	-9.714	7.155
C	51.985	-8.651	6.726
O	53.341	-8.741	6.504
H	45.272	-8.751	7.369
H	47.034	-7.295	5.532
H	47.651	-7.159	7.750
H	47.444	-8.763	7.974
H	49.643	-6.335	6.537
H	49.275	-10.292	7.640
H	51.972	-6.650	6.167
H	51.616	-10.605	7.326
H	53.789	-7.934	6.884
N	47.941	-9.001	4.011
C	48.430	-9.982	3.060
C	49.932	-9.726	2.897
O	50.336	-8.685	2.362
C	47.669	-9.877	1.725
C	48.098	-10.972	0.769
C	46.152	-9.892	1.914
H	48.041	-7.961	3.859
H	48.318	-10.886	3.449
H	47.915	-9.007	1.305
H	48.690	-11.623	1.246
H	47.291	-11.452	0.422
H	48.599	-10.572	0.001
H	45.937	-10.296	2.802
H	45.809	-8.955	1.876
H	45.737	-10.435	1.186
N	50.746	-10.626	3.458
C	52.194	-10.448	3.500
C	52.849	-10.766	2.157
O	52.166	-10.905	1.148
H	50.259	-11.456	3.857
H	52.408	-9.494	3.755
H	52.586	-11.051	4.210
N	54.182	-10.889	2.164

C	54.929	-11.373	1.014
C	55.445	-10.230	0.144
O	55.742	-9.135	0.638
H	54.631	-10.608	3.064
H	55.714	-11.928	1.330
H	54.337	-11.973	0.454
N	55.582	-10.518	-1.159
C	55.875	-9.496	-2.151
C	54.755	-8.455	-2.110
O	53.583	-8.827	-2.196
C	56.009	-10.131	-3.523
H	55.460	-11.526	-1.388
H	56.733	-9.047	-1.916
H	55.257	-10.774	-3.671
H	55.980	-9.421	-4.227
H	56.879	-10.621	-3.583
N	55.119	-7.168	-1.941
C	54.151	-6.074	-1.961
C	53.085	-6.368	-0.898
O	52.007	-6.873	-1.206
C	53.530	-5.897	-3.358
C	54.541	-5.767	-4.499
C	54.621	-4.401	-5.159
O	53.579	-3.930	-5.635
O	55.726	-3.821	-5.218
H	56.140	-7.031	-1.797
H	54.603	-5.215	-1.748
H	52.977	-6.692	-3.541
H	52.992	-5.072	-3.343
H	55.442	-5.975	-4.133
H	54.294	-6.424	-5.203
N	53.471	-6.157	0.363
C	52.592	-6.360	1.501
C	51.505	-5.283	1.440
O	51.792	-4.122	1.120
C	53.394	-6.287	2.779
H	54.463	-5.833	0.455
H	52.157	-7.251	1.427
H	54.190	-6.892	2.716
H	53.707	-5.348	2.931
H	52.826	-6.572	3.553
N	50.276	-5.704	1.764
C	49.115	-4.837	1.696
C	48.247	-5.070	2.925
O	48.247	-6.154	3.509
C	48.319	-5.123	0.421
C	48.120	-3.768	-0.453
C	47.982	-4.029	-1.940
N	49.083	-3.461	-2.703
C	50.280	-4.021	-2.829
N	51.078	-4.119	-1.781
N	50.673	-4.478	-4.005
H	50.234	-6.702	2.066
H	49.417	-3.883	1.694
H	48.812	-5.734	-0.153
H	47.422	-5.423	0.645
H	47.299	-3.325	-0.110
H	48.918	-3.200	-0.283
H	47.957	-5.017	-2.081
H	47.122	-3.623	-2.247
H	48.927	-2.579	-3.170
H	50.798	-3.799	-0.880
H	51.996	-4.529	-1.890
H	50.058	-4.419	-4.796
H	51.580	-4.884	-4.112
N	47.519	-4.027	3.314
C	46.357	-4.213	4.168
C	45.146	-3.739	3.365
O	45.086	-2.582	2.966
C	46.521	-3.487	5.505
C	47.766	-3.956	6.275
C	45.246	-3.650	6.339
C	47.967	-3.203	7.554
H	47.842	-3.111	2.968
H	46.248	-5.189	4.290
H	46.630	-2.509	5.319
H	47.661	-4.925	6.471
H	48.557	-3.823	5.686
H	45.326	-3.107	7.171
H	44.463	-3.336	5.807
H	45.128	-4.611	6.575
H	47.173	-3.330	8.151
H	48.786	-3.540	8.021
H	48.078	-2.227	7.359
N	44.216	-4.654	3.075
C	43.034	-4.322	2.298
C	41.838	-4.199	3.230
O	41.632	-5.092	4.047
C	42.709	-5.410	1.277
C	43.765	-5.527	0.201
H	44.414	-5.603	3.449
H	43.185	-3.419	1.894
H	42.643	-6.281	1.756
H	41.838	-5.188	0.847
N	44.128	-6.755	-0.100
O	44.269	-4.527	-0.317
H	43.721	-7.546	0.355
H	44.834	-6.912	-0.806
N	41.157	-3.047	3.190
C	40.134	-2.774	4.193
C	38.860	-2.253	3.545
O	38.877	-1.667	2.451
C	40.525	-1.723	5.256
C	41.817	-2.034	5.968
O	40.620	-0.438	4.658
H	41.410	-2.411	2.429
H	39.918	-3.634	4.672
H	39.793	-1.696	5.969
H	42.151	-1.219	6.452
H	41.676	-2.771	6.636
H	42.516	-2.326	5.307
H	41.100	-0.492	3.773
N	37.768	-2.427	4.317
C	36.548	-1.676	4.158
C	36.349	-0.869	5.425
O	36.854	-1.258	6.480
C	35.424	-2.646	3.851
C	35.630	-3.235	2.441
C	34.454	-3.922	1.794
H	37.899	-3.171	5.051
H	36.666	-1.037	3.404
H	35.443	-3.390	4.491
H	34.565	-2.170	3.853
H	35.939	-2.477	1.833
H	36.406	-3.895	2.494
N	34.511	-5.243	1.803
O	33.546	-3.292	1.240
H	33.741	-5.799	1.393
H	35.311	-5.709	2.211
N	35.674	0.275	5.302
C	35.517	1.127	6.476
C	34.109	1.694	6.552
O	33.381	1.808	5.553
C	36.603	2.222	6.531
C	36.709	3.111	5.328
C	37.703	3.065	4.396
C	35.876	4.222	4.970
C	36.404	4.782	3.786
C	34.758	4.804	5.568
N	37.505	4.045	3.444
C	35.825	5.882	3.168
C	34.186	5.869	4.953
C	34.718	6.407	3.748
H	35.305	0.483	4.377
H	35.660	0.554	7.291
H	36.419	2.811	7.321
H	37.494	1.779	6.653
H	38.465	2.421	4.396
H	38.075	4.190	2.637
H	34.359	4.411	6.487
H	36.232	6.287	2.252
H	33.300	6.318	5.380
H	34.221	7.249	3.282
N	33.625	2.132	7.757
C	32.338	2.799	8.017
C	32.590	4.097	8.787
O	33.199	4.022	9.882
C	31.470	1.878	8.913
C	30.946	0.570	8.287
C	30.319	-0.298	9.377
C	29.901	0.846	7.224
H	34.223	2.015	8.583
H	31.819	3.023	7.071
H	32.099	1.586	9.777
H	30.600	2.458	9.296
H	31.767	-0.016	7.840
H	29.540	0.271	9.926
H	29.855	-1.206	8.936
H	31.097	-0.627	10.098
H	30.341	1.435	6.398
H	29.526	-0.108	6.798
H	29.056	1.409	7.669
N	32.203	5.330	8.332
C	32.404	6.579	9.112
C	31.082	7.071	9.671
O	30.372	7.852	8.985
C	33.098	7.640	8.223
C	33.687	8.863	8.971
C	34.545	9.670	8.003
C	32.622	9.794	9.550
H	31.715	5.373	7.414
H	33.074	6.393	9.977
H	33.922	7.132	7.680
H	32.399	8.008	7.453
H	34.332	8.517	9.806
H	33.950	9.978	7.118
H	34.927	10.580	8.505
H	35.411	9.064	7.665
H	32.159	9.338	10.447
H	33.074	10.753	9.877
H	31.837	10.013	8.797
N	30.694	6.767	10.944
C	29.483	7.365	11.549
C	29.724	8.796	11.953
O	30.542	9.078	12.865
C	28.917	6.514	12.696
C	27.685	7.170	13.312
O	28.503	5.279	12.173
H	31.328	6.179	11.528
H	28.681	7.379	10.799
H	29.697	6.381	13.482
H	27.979	8.029	13.951
H	26.985	7.518	12.524
H	27.153	6.435	13.945
H	29.241	4.633	12.341
N	29.120	9.853	11.206
C	28.918	11.205	11.757
C	28.084	11.118	13.016
O	27.082	10.362	13.042
C	28.226	12.121	10.705
C	29.029	12.190	9.376
C	28.019	13.539	11.263
C	28.159	12.586	8.180
H	28.511	9.547	10.421
H	29.906	11.637	12.019
H	27.224	11.689	10.490
H	29.864	12.911	9.468
H	29.485	11.208	9.131
H	27.567	14.207	10.505
H	27.312	13.527	12.117
H	28.979	13.976	11.609
H	27.735	13.601	8.310
H	28.776	12.582	7.257
H	27.329	11.861	8.048
N	28.380	11.927	14.151
C	27.456	12.008	15.289
C	26.254	12.836	14.936
O	26.431	14.004	14.492
H	28.977	12.759	13.973
H	27.146	10.986	15.594
H	27.964	12.483	16.153
N	24.953	12.247	14.878
C	23.783	12.933	14.283
C	22.619	12.963	15.225
O	22.423	11.987	15.988

C	23.325	12.249	12.977
C	24.295	12.535	11.842
O	23.217	10.861	13.130
H	24.836	11.285	15.278
H	24.033	13.993	14.067
H	22.338	12.653	12.661
H	24.426	13.628	11.706
H	25.278	12.077	12.068
H	23.902	12.104	10.898
H	22.562	10.703	13.867
N	21.536	13.857	14.964
C	20.223	13.712	15.629
C	19.484	12.448	15.151
O	19.913	11.797	14.159
C	19.342	14.983	15.429
C	20.010	16.268	15.867
O	19.049	15.084	14.057
H	21.695	14.609	14.262
H	20.394	13.595	16.722
H	18.466	14.910	15.935
H	19.353	17.141	15.669
H	20.135	16.184	16.940
H	20.984	16.439	15.373
H	18.282	15.717	13.991
N	18.419	12.046	15.851
C	17.622	10.895	15.477
C	17.045	11.104	14.080
O	17.028	10.171	13.263
C	16.554	10.665	16.556
C	17.160	10.194	17.875
C	16.861	11.073	19.080
O	17.757	11.843	19.480
O	15.750	10.977	19.625
H	18.216	12.638	16.691
H	18.188	10.068	15.482
H	16.109	11.517	16.721
H	15.954	9.965	16.238
H	16.802	9.289	18.072
H	18.146	10.164	17.768
N	16.621	12.344	13.798
C	15.987	12.653	12.525
C	16.975	12.446	11.374
O	16.565	12.153	10.252
C	15.469	14.066	12.541
H	16.783	13.037	14.548
H	15.207	12.034	12.388
H	16.166	14.682	12.915
H	15.242	14.358	11.609
H	14.645	14.123	13.110
N	18.270	12.621	11.653
C	19.335	12.645	10.674
C	20.181	11.400	10.768
O	21.222	11.283	10.070
C	20.178	13.910	10.889
C	19.370	15.087	10.530
H	18.482	12.769	12.657
H	18.805	12.659	9.719
H	20.509	13.982	11.945
H	21.071	13.895	10.232
N	19.468	16.248	11.197
O	18.709	15.051	9.472
H	20.029	16.306	12.074
H	18.830	17.023	10.928
N	19.714	10.354	11.491
C	20.448	9.098	11.720
C	20.578	8.295	10.445
O	21.681	7.763	10.152
C	19.747	8.282	12.808
H	18.777	10.541	11.913
H	21.470	9.334	12.083
H	18.710	8.024	12.504
H	20.311	7.347	13.013
H	19.703	8.874	13.748
N	19.556	8.264	9.539
C	19.692	7.669	8.192
C	20.847	8.257	7.406
O	21.568	7.504	6.701
C	18.377	7.810	7.411
C	18.121	9.186	6.885
C	17.558	10.223	7.570
C	18.531	9.714	5.608
C	18.135	11.070	5.582
C	19.119	9.168	4.466
N	17.560	11.350	6.791
C	18.336	11.889	4.473
C	19.336	9.980	3.375
C	18.940	11.343	3.394
H	18.635	8.641	9.857
H	19.901	6.581	8.309
H	18.403	7.195	6.636
H	17.635	7.583	8.025
H	17.198	10.170	8.499
H	17.196	12.245	7.063
H	19.381	8.203	4.437
H	18.055	12.848	4.487
H	19.792	9.601	2.570
H	19.133	11.953	2.527
N	21.116	9.599	7.466
C	22.263	10.242	6.794
C	23.397	10.500	7.752
O	24.083	11.554	7.624
C	21.814	11.484	6.001
C	21.150	12.562	6.868
C	20.894	13.848	6.073
C	20.019	14.791	6.903
N	19.666	16.013	6.172
H	20.506	10.159	8.094
H	22.701	9.536	6.072
H	22.693	11.911	5.491
H	21.110	11.163	5.209
H	20.183	12.176	7.247
H	21.791	12.802	7.743
H	21.867	14.334	5.844
H	20.388	13.605	5.116
H	19.085	14.250	7.195
H	20.571	15.052	7.839
H	19.126	15.783	5.306
H	19.057	16.605	6.788
H	20.522	16.563	5.930
N	23.876	9.459	8.492
C	25.063	9.539	9.358
C	26.164	8.645	8.844
O	27.258	9.160	8.490
C	24.693	9.207	10.812
O	24.718	7.824	11.057
H	23.357	8.555	8.446
H	25.457	10.577	9.363
H	25.423	9.686	11.486
H	23.696	9.647	11.040
H	23.779	7.536	11.217
N	25.964	7.305	8.668
C	27.063	6.365	8.373
C	27.494	6.473	6.942
O	26.628	6.465	6.038
C	26.674	4.908	8.714
C	27.891	3.992	8.821
O	25.965	4.846	9.925
H	25.001	6.922	8.797
H	27.926	6.626	8.995
H	26.011	4.517	7.914
H	28.622	4.381	9.560
H	27.570	2.981	9.142
H	28.380	3.891	7.839
H	26.553	5.238	10.629
N	28.816	6.543	6.616
C	29.345	6.372	5.256
C	29.927	4.984	5.104
O	30.024	4.229	6.104
C	30.411	7.458	4.977
C	29.815	8.872	4.805
C	30.865	9.936	5.128
C	29.325	9.090	3.372
H	29.493	6.447	7.394
H	28.544	6.450	4.508
H	31.117	7.448	5.823
H	31.014	7.215	4.076
H	28.963	9.017	5.504
H	31.761	9.808	4.484
H	30.440	10.949	4.977
H	31.165	9.848	6.194
H	28.565	8.331	3.106
H	28.860	10.089	3.275
H	30.164	9.009	2.649
N	30.442	4.591	3.915
C	31.175	3.331	3.689
C	32.161	3.536	2.539
O	31.857	4.282	1.605
C	30.227	2.163	3.396
C	29.413	2.376	2.138
C	30.927	0.798	3.412
H	30.398	5.276	3.139
H	31.706	3.103	4.626
H	29.501	2.158	4.240
H	28.445	2.201	2.328
H	29.521	3.320	1.821
H	29.727	1.750	1.422
H	30.693	0.304	2.577
H	31.914	0.940	3.459
H	30.618	0.285	4.210
N	33.342	2.952	2.701
C	34.363	2.981	1.667
C	35.353	1.846	1.819
O	35.129	0.903	2.561
H	33.470	2.479	3.620
H	33.922	2.922	0.758
H	34.856	3.864	1.707
N	36.431	1.933	1.030
C	37.445	0.902	0.964
C	38.799	1.603	0.828
O	38.904	2.568	0.063
C	37.116	-0.119	-0.130
C	36.804	0.523	-1.407
H	36.470	2.816	0.467
H	37.446	0.392	1.816
H	37.906	-0.726	-0.246
H	36.328	-0.662	0.170
C	35.736	1.139	-1.946
N	37.836	0.662	-2.313
C	37.371	1.253	-3.415
N	36.120	1.599	-3.198
H	38.776	0.369	-2.171
H	34.837	1.252	-1.537
H	37.887	1.403	-4.250
N	39.744	1.218	1.687
C	41.107	1.719	1.686
C	42.066	0.538	1.503
O	41.916	-0.502	2.152
C	41.454	2.480	2.978
C	40.885	3.880	3.134
O	40.277	4.366	2.163
O	41.027	4.461	4.262
H	39.404	0.499	2.385
H	41.214	2.304	0.884
H	41.135	1.917	3.756
H	42.462	2.536	3.035
N	43.065	0.716	0.613
C	44.175	-0.221	0.492
C	45.477	0.447	0.931
O	45.832	1.510	0.446
C	44.418	-0.763	-0.932
C	45.453	-1.867	-0.944
O	43.202	-1.285	-1.449
H	42.966	1.573	0.032
H	44.003	-1.003	1.097
H	44.718	0.010	-1.526
H	45.001	-2.763	-0.910
H	46.004	-1.813	-1.781
H	46.060	-1.779	-0.149
H	42.785	-1.929	-0.796
N	46.138	-0.196	1.905
C	47.336	0.338	2.538
C	48.570	-0.395	2.038
O	48.593	-1.635	1.984
C	47.248	0.217	4.049
C	46.172	1.083	4.653

C	44.870	0.616	4.778
C	46.442	2.370	5.050
C	43.883	1.444	5.287
C	45.458	3.182	5.614
C	44.183	2.705	5.722
H	45.713	-1.116	2.167
H	47.424	1.294	2.241
H	47.040	-0.721	4.272
H	48.112	0.500	4.432
H	44.644	-0.310	4.505
H	47.370	2.745	4.936
H	42.928	1.097	5.330
H	45.704	4.109	5.931
H	43.459	3.280	6.123
N	49.604	0.380	1.705
C	50.904	-0.242	1.452
C	51.984	0.593	2.125
O	51.719	1.689	2.596
C	51.247	-0.437	-0.032
C	50.143	-1.072	-0.851
O	51.611	0.806	-0.622
H	49.417	1.379	1.647
H	50.905	-1.152	1.861
H	52.047	-1.073	-0.104
H	49.251	-0.886	-0.429
H	50.146	-0.697	-1.782
H	50.279	-2.065	-0.900
H	50.838	1.451	-0.596
N	53.214	0.061	2.160
C	54.304	0.746	2.827
C	54.929	1.822	1.926
O	55.660	2.669	2.415
C	55.260	-0.313	3.387
C	54.663	-1.091	4.549
C	55.662	-1.626	5.537
C	54.991	-2.212	6.757
N	55.813	-3.285	7.369
H	53.304	-0.851	1.682
H	53.954	1.197	3.660
H	55.477	-0.952	2.659
H	56.081	0.147	3.704
H	54.007	-0.486	5.038
H	54.122	-1.866	4.173
H	56.205	-2.342	5.099
H	56.263	-0.883	5.831
H	54.844	-1.484	7.433
H	54.100	-2.591	6.492
H	56.378	-2.898	8.099
H	55.213	-3.989	7.750
H	56.403	-3.692	6.672
N	54.572	1.870	0.647
C	55.040	2.919	-0.260
C	54.037	4.089	-0.295
O	52.904	3.889	-0.703
C	55.180	2.316	-1.674
C	55.711	3.329	-2.686
C	56.014	1.042	-1.706
H	53.934	1.102	0.345
H	55.909	3.275	0.091
H	54.265	2.050	-1.982
H	55.441	3.051	-3.607
H	55.330	4.230	-2.484
H	56.708	3.366	-2.627
H	55.833	0.551	-2.558
H	56.983	1.281	-1.654
H	55.767	0.467	-0.927
N	54.449	5.307	0.077
C	53.608	6.496	-0.043
C	53.416	6.846	-1.513
O	54.403	6.919	-2.244
C	54.261	7.686	0.667
C	53.444	8.971	0.724
C	54.169	10.084	1.481
C	53.524	11.447	1.327
N	52.051	11.377	1.491
H	55.420	5.331	0.459
H	52.712	6.303	0.363
H	54.471	7.407	1.612
H	55.130	7.888	0.198
H	53.263	9.285	-0.210
H	52.573	8.786	1.183
H	54.186	9.845	2.456
H	55.113	10.136	1.143
H	53.904	12.068	2.018
H	53.738	11.808	0.415
H	51.714	12.246	1.855
H	51.624	11.200	0.604
H	51.820	10.638	2.125
N	52.168	7.090	-1.990
C	51.890	7.182	-3.428
C	52.088	8.590	-4.018
O	52.571	9.446	-3.291
C	50.417	6.749	-3.480
C	49.833	7.344	-2.203
C	50.955	7.307	-1.180
O	51.843	9.027	-5.118
H	52.450	6.504	-3.944
H	49.961	7.128	-4.277
H	50.334	5.760	-3.469
H	49.532	8.284	-2.371
H	49.052	6.797	-1.898
H	51.015	8.171	-0.679
H	50.825	6.556	-0.529
S	38.208	7.982	7.286
O	42.350	7.884	5.288
N	40.384	9.303	5.294
N	40.127	7.009	4.747
C	37.296	13.282	9.698
C	38.225	12.640	8.679
C	37.568	11.462	7.940
C	38.616	10.775	7.066
C	38.099	9.547	6.260
C	38.983	9.135	5.069
C	37.924	6.982	5.934
C	38.772	7.535	4.699
C	41.085	8.067	5.124
H	40.488	9.557	6.256
H	40.144	6.334	5.485
H	37.896	13.668	10.533
H	36.633	12.526	10.137
H	39.120	12.258	9.224
H	38.591	13.397	7.947
H	37.198	10.721	8.681
H	36.722	11.826	7.317
H	39.433	10.472	7.761
H	39.003	11.524	6.342
H	37.128	9.879	5.958
H	38.671	9.785	4.278
H	38.316	6.016	6.173
H	36.891	7.092	5.677
H	38.295	7.302	3.770
C	30.007	18.525	7.296
N	31.665	17.042	9.814
O	35.829	14.325	8.054
C	30.876	17.867	6.264
N	36.299	15.473	9.979
C	28.906	17.890	8.022
O	31.506	21.652	9.998
C	28.476	16.461	7.905
O	33.687	21.410	9.402
C	28.241	18.904	8.778
N	32.359	19.557	9.635
C	27.008	18.754	9.610
C	28.911	20.173	8.549
C	28.464	21.499	9.081
C	29.955	19.941	7.588
C	30.792	20.996	6.940
C	33.425	18.647	9.677
C	33.016	17.284	9.794
C	34.799	18.890	9.663
C	33.952	16.219	9.938
C	35.729	17.833	9.729
C	35.345	16.514	9.850
C	33.440	14.932	10.221
C	31.209	15.819	10.085
C	32.082	14.741	10.313
C	36.419	14.387	9.119
C	32.466	20.944	9.717
C	34.035	22.857	9.505
C	33.192	23.675	8.524
C	33.869	23.334	10.947
C	35.509	22.868	9.094
Ir	30.465	18.730	9.451
H	31.058	16.820	6.517
H	31.842	18.371	6.189
H	30.392	17.905	5.283
H	36.922	15.520	10.774
H	28.086	16.111	8.864
H	29.302	15.809	7.615
H	27.689	16.365	7.151
H	27.107	19.301	10.553
H	26.799	17.716	9.858
H	26.154	19.186	9.077
H	27.907	21.377	10.014
H	29.315	22.146	9.289
H	27.800	21.998	8.365
H	30.852	21.887	7.566
H	31.806	20.649	6.734
H	30.331	21.282	5.987
H	35.167	19.900	9.592
H	36.787	18.071	9.689
H	34.123	14.112	10.409
H	30.136	15.717	10.177
H	31.673	13.777	10.586
H	33.564	24.704	8.505
H	33.271	23.270	7.511
H	32.145	23.696	8.827
H	34.216	24.369	11.030
H	34.472	22.724	11.626
H	32.826	23.296	11.264
H	35.887	23.894	9.114
H	35.637	22.472	8.084
H	36.111	22.273	9.787
N	29.754	18.532	11.300
C	29.047	17.515	11.856
O	28.553	16.633	11.129
C	28.792	17.516	13.360
H	28.764	16.469	13.681
H	27.775	17.902	13.520
C	29.814	18.341	14.142
H	30.774	17.818	14.158
H	29.478	18.458	15.181
C	29.963	19.691	13.473
H	30.108	19.337	12.271
H	28.998	20.208	13.394
C	31.080	20.651	13.823
C	30.757	21.309	15.200
C	32.495	20.048	13.802
H	31.053	21.459	13.077
C	31.843	22.311	15.622
H	30.664	20.531	15.967
H	29.781	21.808	15.148
C	33.545	21.091	14.210
H	32.560	19.197	14.491
H	32.712	19.655	12.801
C	33.241	21.680	15.594
H	31.610	22.690	16.623
H	31.819	23.176	14.946
H	34.545	20.644	14.191
H	33.552	21.903	13.473
H	33.998	22.423	15.867
H	33.301	20.885	16.351
N	5.895	-16.409	-4.446
C	6.072	-15.132	-3.679
C	5.712	-13.892	-4.493
O	5.793	-12.789	-3.964
C	7.514	-14.873	-3.249
C	8.108	-15.913	-2.322
O	7.335	-16.676	-1.661
O	9.339	-15.996	-2.319
H	5.569	-16.397	-5.399
H	5.431	-15.182	-2.900
H	8.078	-14.825	-4.079
H	7.542	-13.981	-2.789
N	5.345	-14.072	-5.767

C	4.885	-12.989	-6.622
C	3.762	-12.202	-5.939
O	3.828	-10.976	-5.871
C	4.443	-13.595	-7.957
C	4.312	-12.585	-9.060
C	3.729	-13.160	-10.341
O	3.587	-12.393	-11.295
O	3.389	-14.366	-10.364
H	5.420	-15.066	-6.092
H	5.647	-12.366	-6.827
H	5.126	-14.252	-8.222
H	3.563	-14.011	-7.815
H	3.705	-11.865	-8.762
H	5.208	-12.235	-9.281
N	2.751	-12.917	-5.408
C	1.622	-12.318	-4.708
C	2.053	-11.659	-3.399
O	1.514	-10.616	-2.996
C	0.602	-13.396	-4.424
H	2.855	-13.952	-5.547
H	1.209	-11.611	-5.300
H	-0.319	-13.049	-4.609
H	0.777	-14.191	-5.008
H	0.661	-13.675	-3.464
N	2.977	-12.322	-2.694
C	3.450	-11.867	-1.404
C	4.236	-10.561	-1.519
O	4.150	-9.691	-0.644
H	3.321	-13.193	-3.161
H	2.666	-11.723	-0.783
H	4.044	-12.571	-0.990
N	5.056	-10.439	-2.573
C	5.892	-9.238	-2.719
C	5.104	-8.074	-3.344
O	5.341	-6.896	-3.037
C	7.151	-9.571	-3.552
C	8.070	-10.525	-2.787
C	7.896	-8.319	-4.007
C	9.064	-11.290	-3.670
H	5.048	-11.219	-3.234
H	6.134	-8.950	-1.788
H	6.853	-10.028	-4.384
H	8.587	-10.001	-2.112
H	7.503	-11.193	-2.305
H	8.649	-8.581	-4.607
H	7.268	-7.721	-4.501
H	8.256	-7.839	-3.209
H	9.645	-10.634	-4.143
H	9.617	-11.882	-3.092
H	8.554	-11.834	-4.330
N	4.239	-8.375	-4.317
C	3.482	-7.337	-4.994
C	2.643	-6.541	-3.987
O	1.915	-7.103	-3.189
C	2.601	-7.942	-6.094
C	1.656	-6.931	-6.705
O	3.485	-8.438	-7.104
H	4.163	-9.383	-4.534
H	4.124	-6.701	-5.435
H	2.085	-8.722	-5.711
H	0.711	-7.267	-6.653
H	1.718	-6.059	-6.211
H	1.893	-6.776	-7.668
H	3.788	-9.370	-6.878
N	2.715	-5.217	-4.061
C	1.946	-4.372	-3.165
C	2.750	-3.184	-2.651
O	3.736	-2.770	-3.273
H	3.351	-4.853	-4.794
H	1.122	-4.030	-3.647
H	1.625	-4.921	-2.375
N	2.290	-2.648	-1.518
C	2.805	-1.419	-0.958
C	3.485	-1.770	0.354
O	2.930	-2.460	1.218
C	1.698	-0.367	-0.759
C	2.233	0.920	-0.174
O	1.064	-0.078	-2.008
H	1.518	-3.204	-1.072
H	3.488	-1.042	-1.575
H	0.993	-0.748	-0.124
H	3.228	0.857	-0.054
H	2.029	1.689	-0.787
H	1.809	1.096	0.719
H	1.735	-0.109	-2.757
N	4.712	-1.255	0.492
C	5.539	-1.486	1.655
C	5.963	-0.154	2.257
O	6.234	0.801	1.540
C	6.745	-2.294	1.205
C	6.404	-3.661	0.703
C	6.160	-4.020	-0.586
C	6.427	-4.866	1.462
C	6.126	-5.913	0.581
C	6.617	-5.160	2.810
N	6.013	-5.374	-0.671
C	6.034	-7.234	1.005
C	6.511	-6.459	3.235
C	6.230	-7.488	2.338
H	5.016	-0.668	-0.324
H	4.986	-1.964	2.342
H	7.201	-1.813	0.463
H	7.366	-2.409	1.974
H	6.097	-3.387	-1.357
H	5.849	-5.894	-1.511
H	6.831	-4.429	3.465
H	5.830	-7.966	0.347
H	6.637	-6.670	4.203
H	6.172	-8.427	2.679
N	6.073	-0.141	3.577
C	6.435	1.038	4.327
C	7.645	0.739	5.195
O	7.725	-0.299	5.836
C	5.256	1.462	5.220
C	3.967	1.637	4.471
C	3.742	2.774	3.717
C	2.956	0.685	4.527
C	2.584	2.936	2.975
C	1.773	0.844	3.812
C	1.574	1.990	3.068
O	0.444	2.180	2.328
H	5.872	-1.069	4.030
H	6.686	1.766	3.682
H	5.130	0.759	5.914
H	5.494	2.328	5.650
H	4.435	3.505	3.705
H	3.080	-0.131	5.091
H	2.475	3.729	2.374
H	1.073	0.131	3.839
H	0.375	1.463	1.634
N	8.593	1.685	5.246
C	9.743	1.451	6.089
C	9.536	2.222	7.380
O	8.510	2.868	7.577
C	11.069	1.739	5.383
C	11.326	3.206	5.132
H	8.430	2.510	4.679
H	9.752	0.483	6.325
H	11.811	1.376	5.951
H	11.065	1.259	4.503
N	12.400	3.498	4.403
O	10.568	4.054	5.580
H	12.981	2.764	4.050
H	12.627	4.452	4.207
N	10.515	2.087	8.263
C	10.509	2.715	9.568
C	10.484	4.243	9.423
O	9.990	4.896	10.311
C	11.680	2.143	10.387
C	13.097	2.573	9.960
C	13.731	1.992	8.698
H	11.307	1.476	7.929
H	9.669	2.446	10.047
H	11.559	2.413	11.353
H	11.642	1.134	10.341
H	13.096	3.600	9.868
H	13.731	2.381	10.750
N	14.809	2.641	8.268
O	13.305	0.987	8.111
H	15.305	2.308	7.433
H	15.130	3.458	8.768
N	10.976	4.814	8.309
C	10.989	6.267	8.094
C	9.650	6.808	7.568
O	9.513	8.021	7.418
C	12.077	6.612	7.067
C	13.519	6.316	7.478
C	14.461	6.541	6.300
C	13.917	7.166	8.671
H	11.348	4.133	7.611
H	11.185	6.719	8.979
H	11.889	6.088	6.239
H	12.014	7.588	6.875
H	13.585	5.366	7.777
H	14.396	7.492	5.999
H	15.400	6.344	6.582
H	14.204	5.934	5.548
H	13.312	6.966	9.444
H	14.863	6.962	8.929
H	13.841	8.136	8.434
N	8.733	5.912	7.158
C	7.458	6.292	6.561
C	7.533	6.424	5.038
O	6.579	6.832	4.387
H	9.022	4.917	7.308
H	6.756	5.600	6.797
H	7.152	7.175	6.953
N	8.663	6.043	4.442
C	8.739	6.004	2.992
C	7.838	4.876	2.478
O	7.673	3.888	3.179
C	10.185	5.831	2.522
O	11.026	6.852	3.044
H	9.433	5.793	5.073
H	8.382	6.864	2.632
H	10.521	4.946	2.833
H	10.207	5.874	1.527
H	11.108	6.739	4.033
N	7.393	5.011	1.234
C	6.441	4.117	0.586
C	7.029	3.535	-0.698
O	7.363	4.270	-1.617
C	5.134	4.851	0.243
C	4.073	3.922	-0.307
O	4.660	5.475	1.437
H	7.802	5.853	0.733
H	6.238	3.356	1.212
H	5.334	5.574	-0.439
H	3.164	4.218	-0.001
H	4.096	3.929	-1.311
H	4.235	2.985	0.016
H	5.432	5.735	2.029
N	7.074	2.203	-0.763
C	7.576	1.411	-1.881
C	6.389	0.659	-2.514
O	5.805	-0.204	-1.849
C	8.669	0.526	-1.264
C	9.169	-0.674	-2.008
C	9.534	-0.593	-3.335
C	9.431	-1.853	-1.323
C	10.093	-1.686	-3.983
C	9.894	-2.968	-1.989
C	10.259	-2.879	-3.312
H	6.690	1.738	0.121
H	7.906	2.047	-2.571
H	9.467	1.126	-1.078
H	8.320	0.207	-0.365
H	9.400	0.255	-3.841
H	9.281	-1.890	-0.333
H	10.374	-1.596	-4.949
H	9.962	-3.855	-1.495
H	10.643	-3.671	-3.788
N	6.030	1.002	-3.760
C	4.984	0.317	-4.505
C	5.611	-0.545	-5.606

O	6.253	-0.070	-6.529
C	3.953	1.320	-5.069
C	3.348	2.175	-3.947
C	2.873	0.605	-5.877
C	2.495	3.327	-4.434
H	6.574	1.810	-4.147
H	4.546	-0.316	-3.866
H	4.430	1.928	-5.696
H	2.792	1.581	-3.365
H	4.100	2.542	-3.397
H	2.265	1.281	-6.289
H	3.302	0.061	-6.595
H	2.349	0.008	-5.274
H	1.734	2.970	-4.974
H	2.140	3.831	-3.648
H	3.051	3.935	-4.999
N	5.422	-1.852	-5.513
C	6.190	-2.741	-6.363
C	5.282	-3.793	-6.971
O	4.337	-4.251	-6.327
C	7.357	-3.388	-5.599
C	6.866	-4.374	-4.555
C	8.325	-4.045	-6.570
H	4.726	-2.158	-4.827
H	6.584	-2.200	-7.112
H	7.840	-2.661	-5.111
H	7.411	-5.212	-4.601
H	6.955	-3.971	-3.644
H	5.905	-4.595	-4.727
H	8.174	-3.677	-7.487
H	9.263	-3.854	-6.282
H	8.169	-5.033	-6.575
N	5.603	-4.155	-8.216
C	5.034	-5.324	-8.870
C	6.139	-6.343	-9.097
O	7.122	-6.054	-9.758
C	4.346	-4.982	-10.207
C	3.674	-6.179	-10.856
O	3.369	-3.970	-9.956
H	6.299	-3.523	-8.677
H	4.349	-5.729	-8.265
H	5.040	-4.607	-10.845
H	2.687	-6.154	-10.680
H	3.830	-6.163	-11.847
H	4.052	-7.029	-10.480
H	3.812	-3.127	-9.630
N	5.947	-7.550	-8.563
C	6.844	-8.647	-8.864
C	6.336	-9.394	-10.104
O	5.237	-9.948	-10.090
C	6.913	-9.526	-7.653
H	5.127	-7.624	-7.937
H	7.755	-8.285	-9.067
H	6.130	-9.347	-7.050
H	6.897	-10.492	-7.925
H	7.760	-9.348	-7.144
N	7.122	-9.389	-11.180
C	6.790	-10.153	-12.378
C	7.109	-11.634	-12.198
O	7.879	-12.005	-11.312
H	7.976	-8.804	-11.092
H	5.809	-10.048	-12.582
H	7.313	-9.794	-13.161
N	6.541	-12.484	-13.065
C	6.700	-13.931	-12.926
C	8.140	-14.391	-13.240
O	8.554	-15.466	-12.836
C	5.670	-14.621	-13.802
H	5.996	-12.035	-13.819
H	6.488	-14.194	-11.974
H	4.778	-14.605	-13.349
H	5.603	-14.146	-14.680
H	5.944	-15.570	-13.960
N	8.880	-13.560	-13.972
C	10.205	-13.809	-14.519
C	11.337	-13.303	-13.608
O	12.492	-13.347	-13.998
C	10.327	-12.967	-15.796
C	10.283	-11.445	-15.555
O	9.305	-10.938	-14.899
O	11.249	-10.749	-16.017
H	8.380	-12.619	-14.138
H	10.320	-14.814	-14.591
H	11.196	-13.186	-16.231
H	9.573	-13.207	-16.401
N	10.993	-12.672	-12.489
C	11.965	-12.159	-11.541
C	12.118	-10.635	-11.563
O	12.985	-10.138	-10.846
H	9.957	-12.578	-12.353
H	11.704	-12.447	-10.598
H	12.875	-12.581	-11.726
N	11.282	-9.908	-12.343
C	11.329	-8.443	-12.432
C	10.634	-7.751	-11.255
O	9.596	-8.211	-10.768
C	10.716	-7.988	-13.743
H	10.598	-10.490	-12.878
H	12.300	-8.153	-12.446
H	10.072	-7.242	-13.572
H	11.437	-7.668	-14.359
H	10.231	-8.752	-14.171
N	11.247	-6.660	-10.768
C	10.600	-5.723	-9.852
C	10.517	-4.383	-10.564
O	11.513	-3.908	-11.124
C	11.369	-5.579	-8.522
C	11.600	-6.820	-7.667
C	12.477	-6.452	-6.472
C	10.301	-7.443	-7.153
H	12.231	-6.542	-11.104
H	9.679	-6.043	-9.666
H	12.273	-5.192	-8.742
H	10.867	-4.913	-7.959
H	12.061	-7.513	-8.211
H	12.019	-5.750	-5.928
H	12.630	-7.264	-5.910
H	13.354	-6.102	-6.800
H	9.732	-7.710	-7.930
H	10.516	-8.249	-6.602
H	9.812	-6.774	-6.594
N	9.303	-3.802	-10.547
C	9.004	-2.518	-11.161
C	8.018	-1.798	-10.243
O	7.236	-2.444	-9.565
C	8.421	-2.708	-12.575
C	9.217	-3.663	-13.437
O	7.102	-3.224	-12.415
H	8.578	-4.375	-10.039
H	9.837	-1.981	-11.207
H	8.362	-1.805	-13.026
H	9.671	-3.161	-14.178
H	9.915	-4.126	-12.884
H	8.609	-4.354	-13.838
H	7.095	-3.974	-11.743
N	8.123	-0.478	-10.142
C	7.209	0.279	-9.324
C	7.759	1.658	-9.013
O	8.454	2.247	-9.832
H	8.898	-0.060	-10.689
H	6.323	0.376	-9.801
H	7.038	-0.213	-8.457
N	7.385	2.176	-7.845
C	7.714	3.537	-7.495
C	8.067	3.582	-6.021
O	7.609	2.774	-5.215
C	6.565	4.525	-7.796
C	6.184	4.545	-9.256
O	5.429	4.146	-7.013
H	6.854	1.529	-7.234
H	8.515	3.824	-8.020
H	6.854	5.456	-7.513
H	5.292	4.992	-9.375
H	6.872	5.050	-9.787
H	6.126	3.607	-9.612
H	5.721	3.645	-6.191
N	8.844	4.597	-5.693
C	9.337	4.808	-4.353
C	9.052	6.259	-4.024
O	9.335	7.136	-4.837
C	10.853	4.583	-4.250
C	11.338	4.316	-2.843
C	11.632	5.352	-1.975
C	11.470	3.021	-2.367
C	12.070	5.108	-0.685
C	11.878	2.759	-1.065
C	12.186	3.803	-0.231
O	12.573	3.516	1.049
H	9.072	5.239	-6.494
H	8.820	4.239	-3.727
H	11.089	3.799	-4.818
H	11.311	5.400	-4.589
H	11.527	6.303	-2.284
H	11.265	2.250	-2.976
H	12.302	5.872	-0.078
H	11.945	1.813	-0.744
H	12.216	4.215	1.668
N	8.480	6.481	-2.852
C	8.347	7.834	-2.349
C	9.075	7.906	-1.021
O	8.685	7.194	-0.102
C	6.869	8.168	-2.188
C	6.641	9.508	-1.516
C	5.191	9.781	-1.149
O	4.938	10.128	0.041
O	4.323	9.637	-2.047
H	8.152	5.642	-2.351
H	8.753	8.479	-2.990
H	6.471	8.211	-3.082
H	6.464	7.478	-1.624
H	7.166	9.529	-0.679
H	6.926	10.222	-2.137
N	10.143	8.720	-0.954
C	10.932	8.881	0.269
C	10.342	9.999	1.130
O	10.085	11.099	0.648
C	12.410	9.153	-0.010
O	13.162	9.337	1.207
H	10.355	9.220	-1.842
H	10.875	8.028	0.791
H	12.798	8.380	-0.503
H	12.493	9.982	-0.556
H	13.454	10.287	1.263
N	10.186	9.701	2.421
C	9.736	10.667	3.409
C	10.899	11.585	3.818
O	10.666	12.608	4.471
C	9.148	9.917	4.593
H	10.418	8.706	2.658
H	9.013	11.236	3.009
H	8.766	9.047	4.282
H	9.864	9.742	5.268
H	8.425	10.466	5.012
N	12.151	11.209	3.473
C	13.329	11.982	3.873
C	14.200	12.319	2.650
O	14.063	11.755	1.546
C	14.174	11.295	4.980
C	13.355	10.960	6.226
C	14.921	10.056	4.474
H	12.195	10.340	2.911
H	13.016	12.848	4.273
H	14.883	11.944	5.262
H	12.402	11.219	6.075
H	13.411	9.979	6.405
H	13.720	11.464	7.007
H	15.631	9.814	5.132
H	14.273	9.303	4.379
H	15.334	10.264	3.589
N	15.078	13.306	2.872
C	16.088	13.696	1.906
C	15.562	14.654	0.836
O	14.486	15.248	0.985
H	14.967	13.768	3.806
H	16.866	14.139	2.388

H	16.459	12.867	1.449
N	16.366	14.770	-0.237
C	16.112	15.615	-1.399
C	15.259	14.801	-2.385
O	15.686	14.429	-3.488
C	17.451	16.178	-1.910
C	17.363	16.999	-3.185
H	17.238	14.166	-0.159
H	15.552	16.378	-1.098
H	17.842	16.756	-1.185
H	18.072	15.403	-2.072
N	18.523	17.310	-3.747
O	16.276	17.331	-3.669
H	19.384	17.007	-3.342
H	18.539	17.856	-4.590
N	14.018	14.540	-1.948
C	13.073	13.680	-2.639
C	11.697	14.332	-2.573
O	11.292	14.806	-1.512
C	13.053	12.312	-1.982
H	13.788	15.026	-1.039
H	13.342	13.600	-3.596
H	13.841	11.779	-2.292
H	13.094	12.416	-0.988
H	12.211	11.832	-2.230
N	10.968	14.327	-3.689
C	9.607	14.855	-3.699
C	8.722	14.046	-4.652
O	8.912	14.035	-5.876
C	9.621	16.344	-4.061
C	8.240	16.957	-4.264
C	8.241	18.263	-5.043
O	9.312	18.921	-5.109
O	7.184	18.595	-5.622
H	11.437	13.929	-4.518
H	9.220	14.816	-2.773
H	10.055	16.828	-3.321
H	10.117	16.443	-4.906
H	7.688	16.311	-4.769
H	7.851	17.143	-3.374
N	7.703	13.417	-4.072
C	6.810	12.616	-4.874
C	7.515	11.284	-5.164
O	8.380	10.847	-4.393
C	6.401	13.404	-6.130
O	5.224	12.887	-6.735
H	7.621	13.551	-3.057
H	5.994	12.413	-4.343
H	6.241	14.356	-5.870
H	7.151	13.360	-6.791
H	5.347	12.855	-7.725
N	7.110	10.662	-6.278
C	7.428	9.286	-6.599
C	8.584	9.275	-7.596
O	8.726	10.160	-8.440
C	6.174	8.548	-7.111
C	4.908	8.848	-6.306
C	3.816	7.787	-6.285
N	4.327	6.513	-5.770
C	4.626	6.216	-4.493
N	4.113	6.925	-3.494
N	5.397	5.154	-4.233
H	6.529	11.277	-6.909
H	7.719	8.812	-5.770
H	6.015	8.826	-8.050
H	6.348	7.572	-7.059
H	5.173	9.039	-5.337
H	4.492	9.712	-6.660
H	3.084	8.108	-5.698
H	3.492	7.659	-7.214
H	4.474	5.764	-6.453
H	3.482	7.676	-3.676
H	4.361	6.703	-2.547
H	5.724	4.584	-4.985
H	5.640	4.935	-3.291
N	9.390	8.219	-7.495
C	10.529	7.994	-8.361
C	10.422	6.573	-8.867
O	9.903	5.718	-8.139
C	11.824	8.136	-7.572
C	12.071	9.532	-7.078
C	11.502	9.992	-5.902
C	12.879	10.395	-7.805
C	11.716	11.285	-5.457
C	13.107	11.690	-7.366
C	12.506	12.138	-6.203
O	12.693	13.427	-5.769
H	9.121	7.557	-6.721
H	10.465	8.610	-9.138
H	11.779	7.526	-6.785
H	12.582	7.874	-8.164
H	10.921	9.377	-5.359
H	13.301	10.081	-8.651
H	11.303	11.599	-4.601
H	13.705	12.300	-7.889
H	13.611	13.515	-5.385
N	10.903	6.351	-10.092
C	10.886	5.027	-10.680
C	11.838	4.109	-9.908
O	12.945	4.496	-9.575
C	11.228	5.083	-12.177
C	11.446	3.705	-12.771
C	10.142	5.807	-12.973
H	11.279	7.192	-10.568
H	9.962	4.650	-10.592
H	12.092	5.584	-12.273
H	11.365	3.012	-12.053
H	10.759	3.530	-13.478
H	12.359	3.654	-13.178
H	10.329	5.705	-13.949
H	9.254	5.405	-12.754
H	10.147	6.775	-12.726
N	11.404	2.871	-9.660
C	12.341	1.848	-9.243
C	12.264	0.658	-10.168
O	11.234	0.360	-10.770
C	12.117	1.460	-7.792
C	10.935	0.585	-7.428
C	11.183	-0.914	-7.686
C	10.606	0.832	-5.962
H	10.396	2.724	-9.787
H	13.272	2.228	-9.284
H	12.952	0.983	-7.471
H	12.032	2.315	-7.257
H	10.127	0.872	-7.964
H	11.962	-1.213	-7.140
H	10.369	-1.427	-7.426
H	11.375	-1.049	-8.655
H	10.377	1.797	-5.829
H	9.826	0.265	-5.694
H	11.398	0.597	-5.398
N	13.416	0.016	-10.274
C	13.541	-1.243	-10.956
C	14.432	-2.180	-10.144
O	15.392	-1.754	-9.494
C	14.037	-0.984	-12.378
C	15.487	-0.586	-12.490
O	13.868	-2.229	-13.044
H	14.223	0.508	-9.817
H	12.635	-1.668	-11.034
H	13.443	-0.303	-12.814
H	16.058	-1.397	-12.648
H	15.613	0.052	-13.256
H	15.790	-0.138	-11.643
H	13.199	-2.804	-12.558
N	14.132	-3.467	-10.201
C	14.980	-4.445	-9.529
C	14.732	-5.870	-9.995
O	14.082	-6.105	-11.012
H	13.288	-3.708	-10.736
H	15.955	-4.207	-9.688
H	14.824	-4.390	-8.526
N	15.206	-6.807	-9.185
C	15.096	-8.232	-9.475
C	14.880	-8.994	-8.178
O	15.318	-8.567	-7.120
C	16.375	-8.740	-10.154
C	16.779	-8.059	-11.462
C	15.958	-8.394	-12.682
N	16.048	-9.812	-12.984
C	15.182	-10.463	-13.750
N	14.145	-9.814	-14.239
N	15.293	-11.768	-13.920
H	15.666	-6.437	-8.320
H	14.307	-8.382	-10.073
H	17.136	-8.631	-9.505
H	16.258	-9.721	-10.344
H	16.746	-7.049	-11.326
H	17.751	-8.292	-11.662
H	15.012	-8.155	-12.498
H	16.310	-7.870	-13.449
H	16.813	-10.334	-12.589
H	13.994	-8.845	-14.073
H	13.469	-10.316	-14.810
H	16.049	-12.264	-13.482
H	14.633	-12.265	-14.481
N	14.271	-10.179	-8.277
C	14.107	-11.044	-7.133
C	14.298	-12.477	-7.631
O	14.158	-12.749	-8.846
C	12.774	-10.772	-6.442
C	11.559	-11.342	-7.153
C	11.001	-10.731	-8.268
C	10.956	-12.496	-6.683
C	9.906	-11.285	-8.935
C	9.838	-13.033	-7.311
C	9.326	-12.445	-8.448
O	8.216	-13.012	-9.040
H	13.936	-10.410	-9.234
H	14.824	-10.851	-6.475
H	12.808	-11.168	-5.524
H	12.651	-9.781	-6.371
H	11.389	-9.869	-8.608
H	11.330	-12.956	-5.877
H	9.547	-10.846	-9.758
H	9.402	-13.856	-6.930
H	8.109	-12.636	-9.958
N	14.593	-13.341	-6.680
C	14.674	-14.777	-6.911
C	13.262	-15.334	-7.083
O	12.526	-15.465	-6.100
C	15.383	-15.506	-5.777
C	15.546	-17.037	-5.918
O	15.121	-17.626	-6.957
O	16.100	-17.656	-4.955
H	14.765	-12.909	-5.739
H	15.150	-14.915	-7.779
H	16.307	-15.110	-5.675
H	14.875	-15.332	-4.922
N	12.902	-15.678	-8.336
C	11.538	-16.127	-8.589
C	11.367	-17.647	-8.391
O	10.244	-18.141	-8.525
C	11.090	-15.670	-9.941
O	12.042	-16.025	-10.922
H	13.633	-15.597	-9.041
H	10.925	-15.667	-7.929
H	10.221	-16.101	-10.169
H	10.987	-14.678	-9.942
H	12.099	-15.303	-11.612
N	12.436	-18.393	-8.040
C	12.310	-19.819	-7.708
C	13.078	-20.115	-6.427
O	14.080	-20.833	-6.437
C	12.826	-20.685	-8.832
H	13.333	-17.878	-8.027
H	11.342	-20.029	-7.561
H	12.123	-21.344	-9.104
H	13.062	-20.114	-9.621
H	13.642	-21.182	-8.531
N	12.637	-19.564	-5.288
C	13.434	-19.606	-4.060
C	13.506	-21.004	-3.449
O	12.841	-21.921	-3.925
C	12.695	-18.659	-3.116

C	11.269	-18.716	-3.588
C	11.325	-18.920	-5.092
H	14.367	-19.243	-4.228
H	12.767	-18.972	-2.169
H	13.055	-17.730	-3.188
H	10.782	-19.477	-3.146
H	10.791	-17.859	-3.365
H	10.589	-19.516	-5.402
H	11.283	-18.048	-5.575
N	14.328	-21.133	-2.406
C	14.520	-22.394	-1.714
C	13.254	-22.675	-0.924
O	12.499	-21.751	-0.644
C	15.745	-22.335	-0.828
H	14.817	-20.249	-2.136
H	14.647	-23.118	-2.384
H	15.714	-23.077	-0.157
H	16.571	-22.431	-1.385
H	15.773	-21.457	-0.347
N	12.993	-23.953	-0.629
C	11.849	-24.310	0.198
C	12.381	-24.975	1.460
O	12.203	-26.173	1.664
C	10.866	-25.237	-0.532
C	10.072	-24.562	-1.634
O	11.678	-26.302	-1.027
H	13.655	-24.637	-1.028
H	11.369	-23.478	0.458
H	10.216	-25.612	0.133
H	10.688	-24.041	-2.230
H	9.593	-25.252	-2.183
H	9.397	-23.936	-1.235
H	12.404	-25.943	-1.624
N	13.088	-24.186	2.267
C	13.660	-24.648	3.519
C	13.358	-23.627	4.617
O	13.907	-23.734	5.712
C	15.159	-24.932	3.339
C	15.967	-23.740	2.825
O	15.402	-22.626	2.776
O	17.144	-23.936	2.442
H	13.194	-23.204	1.917
H	13.190	-25.494	3.773
H	15.528	-25.205	4.225
H	15.254	-25.682	2.688
N	12.477	-22.647	4.335
C	12.179	-21.571	5.279
C	13.104	-20.348	5.140
O	13.110	-19.462	6.003
H	12.037	-22.728	3.397
H	11.217	-21.268	5.151
H	12.250	-21.927	6.229
N	13.896	-20.339	4.066
C	14.755	-19.224	3.687
C	13.935	-18.067	3.145
O	12.986	-18.285	2.394
C	15.694	-19.628	2.577
O	16.759	-20.374	3.066
H	13.845	-21.231	3.501
H	15.235	-18.905	4.513
H	15.187	-20.172	1.907
H	16.044	-18.801	2.133
H	16.519	-21.348	3.088
N	14.416	-16.843	3.391
C	13.909	-15.684	2.687
C	14.214	-15.721	1.194
O	15.033	-16.504	0.689
H	15.161	-16.808	4.110
H	12.904	-15.620	2.816
H	14.312	-14.841	3.085
N	13.528	-14.819	0.489
C	13.672	-14.645	-0.945
C	14.550	-13.420	-1.214
O	14.149	-12.309	-0.887
C	12.290	-14.459	-1.580
C	12.332	-14.265	-3.079
O	11.543	-15.622	-1.215
H	12.869	-14.242	1.071
H	14.132	-15.447	-1.325
H	11.846	-13.654	-1.172
H	12.968	-14.922	-3.494
H	11.418	-14.406	-3.471
H	12.639	-13.334	-3.298
H	10.672	-15.652	-1.717
N	15.698	-13.641	-1.847
C	16.629	-12.560	-2.136
C	16.086	-11.608	-3.209
O	15.519	-12.011	-4.222
C	17.955	-13.164	-2.519
H	15.867	-14.631	-2.111
H	16.786	-12.028	-1.291
H	18.577	-12.447	-2.841
H	18.365	-13.619	-1.726
H	17.826	-13.837	-3.251
N	16.337	-10.298	-3.023
C	15.885	-9.303	-3.976
C	16.735	-8.045	-3.837
O	17.518	-7.914	-2.891
C	14.380	-9.017	-3.804
C	13.863	-8.642	-2.414
C	14.483	-7.394	-1.881
C	12.330	-8.483	-2.472
H	16.867	-10.082	-2.161
H	16.004	-9.662	-4.903
H	14.146	-8.261	-4.424
H	13.883	-9.839	-4.097
H	14.050	-9.415	-1.787
H	14.288	-6.619	-2.491
H	14.117	-7.184	-0.969
H	15.480	-7.502	-1.809
H	11.925	-9.347	-2.763
H	11.995	-8.239	-1.565
H	12.103	-7.763	-3.124
N	16.597	-7.172	-4.822
C	17.206	-5.859	-4.754
C	16.558	-4.907	-5.733
O	15.921	-5.341	-6.700
H	16.029	-7.503	-5.625
H	17.113	-5.492	-3.818
H	18.192	-5.933	-4.960
N	16.699	-3.604	-5.469
C	16.192	-2.639	-6.429
C	16.987	-1.358	-6.318
O	17.745	-1.188	-5.387
C	14.687	-2.411	-6.241
C	14.320	-1.725	-4.966
C	14.213	-0.384	-4.797
C	13.981	-2.334	-3.703
C	13.688	-1.276	-2.827
C	13.953	-3.651	-3.212
N	13.825	-0.110	-3.526
C	13.376	-1.487	-1.490
C	13.608	-3.871	-1.900
C	13.347	-2.794	-1.049
H	17.163	-3.373	-4.593
H	16.339	-3.013	-7.347
H	14.354	-1.846	-6.991
H	14.227	-3.295	-6.248
H	14.394	0.301	-5.505
H	13.661	0.809	-3.147
H	14.181	-4.418	-3.813
H	13.183	-0.718	-0.880
H	13.545	-4.800	-1.556
H	13.131	-2.981	-0.086
N	16.827	-0.502	-7.324
C	17.520	0.762	-7.430
C	16.479	1.862	-7.682
O	15.511	1.644	-8.411
C	18.497	0.778	-8.612
C	19.228	2.102	-8.770
O	19.413	-0.317	-8.497
H	16.135	-0.836	-8.051
H	17.975	0.961	-6.565
H	17.970	0.661	-9.479
H	20.216	1.959	-8.677
H	19.037	2.491	-9.674
H	18.922	2.746	-8.065
H	20.115	-0.263	-9.215
N	16.726	3.011	-7.059
C	16.063	4.270	-7.370
C	17.154	5.303	-7.698
O	18.057	5.525	-6.912
C	15.180	4.767	-6.212
C	14.578	6.137	-6.527
C	14.070	3.802	-5.826
H	17.460	2.931	-6.303
H	15.510	4.145	-8.186
H	15.763	4.886	-5.412
H	14.585	6.282	-7.515
H	13.639	6.170	-6.190
H	15.120	6.847	-6.080
H	13.291	3.932	-6.439
H	14.406	2.863	-5.904
H	13.789	3.981	-4.883
N	17.067	5.951	-8.858
C	17.819	7.170	-9.130
C	16.929	8.357	-8.786
O	15.759	8.374	-9.165
C	18.253	7.226	-10.579
H	16.419	5.513	-9.549
H	18.624	7.193	-8.544
H	17.459	7.398	-11.164
H	18.919	7.962	-10.704
H	18.672	6.355	-10.841
N	17.458	9.293	-7.996
C	16.650	10.325	-7.367
C	16.408	11.526	-8.291
O	16.652	12.663	-7.891
C	17.293	10.748	-6.064
C	17.312	9.593	-5.117
C	18.404	8.874	-4.738
C	16.182	9.029	-4.425
C	16.672	7.943	-3.670
C	14.816	9.295	-4.389
N	18.041	7.913	-3.848
C	15.841	7.159	-2.876
C	13.991	8.505	-3.610
C	14.498	7.461	-2.851
H	18.491	9.217	-7.873
H	15.745	9.926	-7.154
H	18.222	11.010	-6.228
H	16.760	11.458	-5.652
H	19.336	9.032	-5.068
H	18.660	7.273	-3.384
H	14.427	10.052	-4.920
H	16.221	6.396	-2.341
H	13.010	8.698	-3.601
H	13.882	6.923	-2.280
N	15.912	11.224	-9.480
C	15.491	12.207	-10.475
C	14.089	11.820	-10.947
O	13.836	10.660	-11.292
C	16.462	12.257	-11.654
C	16.041	13.215	-12.775
C	16.750	12.969	-14.067
C	16.201	13.790	-15.222
N	14.765	13.493	-15.474
H	15.843	10.182	-9.658
H	15.449	13.105	-10.050
H	17.353	12.551	-11.317
H	16.534	11.340	-12.043
H	15.044	13.118	-12.913
H	16.220	14.160	-12.465
H	17.724	13.195	-13.961
H	16.671	11.997	-14.312
H	16.303	14.764	-15.006
H	16.730	13.583	-16.049
H	14.359	14.240	-15.999
H	14.688	12.640	-15.990
H	14.287	13.400	-14.601
N	13.155	12.777	-10.844
C	11.833	12.651	-11.441
C	11.560	13.967	-12.177
O	12.500	14.706	-12.464
C	10.784	12.244	-10.401

C	10.631	13.250	-9.278
H	13.462	13.613	-10.300
H	11.882	11.942	-12.130
H	9.905	12.147	-10.864
H	11.056	11.366	-10.009
N	9.978	12.873	-8.193
O	11.064	14.388	-9.411
H	9.612	11.934	-8.148
H	9.847	13.497	-7.432
N	10.290	14.266	-12.474
C	9.936	15.478	-13.211
C	10.114	16.724	-12.341
O	10.273	17.829	-12.847
C	8.504	15.392	-13.747
C	8.360	14.396	-14.880
H	9.593	13.573	-12.140
H	10.583	15.561	-13.977
H	7.906	15.114	-13.000
H	8.239	16.294	-14.079
N	7.127	14.103	-15.257
O	9.345	13.902	-15.424
H	6.346	14.538	-14.802
H	6.967	13.448	-15.996
N	10.090	16.528	-11.026
C	10.047	17.606	-10.059
C	11.434	17.979	-9.533
O	11.625	19.103	-9.073
C	9.168	17.165	-8.883
C	7.691	17.043	-9.173
C	7.167	17.307	-10.436
C	6.808	16.687	-8.162
C	5.807	17.221	-10.684
C	5.446	16.599	-8.394
C	4.946	16.870	-9.657
O	3.600	16.782	-9.905
H	10.105	15.513	-10.749
H	9.657	18.427	-10.509
H	9.500	16.271	-8.566
H	9.286	17.832	-8.140
H	7.787	17.565	-11.182
H	7.164	16.489	-7.247
H	5.447	17.410	-11.599
H	4.824	16.341	-7.654
H	3.119	16.592	-9.050
N	12.375	17.020	-9.501
C	13.603	17.210	-8.745
C	14.692	16.285	-9.252
O	14.419	15.213	-9.788
C	13.460	16.849	-7.265
C	12.526	17.730	-6.463
C	12.898	17.683	-5.002
N	11.896	18.379	-4.215
C	11.989	18.608	-2.915
N	10.964	19.140	-2.270
N	13.089	18.266	-2.260
H	12.149	16.173	-10.042
H	13.909	18.163	-8.857
H	13.124	15.905	-7.208
H	14.370	16.897	-6.845
H	12.602	18.668	-6.778
H	11.594	17.402	-6.561
H	12.940	16.735	-4.720
H	13.775	18.128	-4.889
H	11.069	18.712	-4.692
H	10.109	19.358	-2.730
H	11.047	19.332	-1.279
H	13.840	17.816	-2.749
H	13.173	18.453	-1.283
N	15.932	16.681	-8.999
C	17.062	15.816	-9.294
C	18.116	16.013	-8.208
O	18.724	17.064	-8.120
C	17.597	16.054	-10.702
C	18.670	15.055	-11.079
H	16.019	17.625	-8.588
H	16.745	14.873	-9.223
H	16.843	15.969	-11.345
H	17.986	16.969	-10.742
N	19.137	15.125	-12.317
O	19.050	14.207	-10.252
H	18.782	15.820	-12.947
H	19.843	14.488	-12.626
N	18.319	14.985	-7.377
C	19.291	15.028	-6.299
C	20.650	14.446	-6.728
O	21.545	14.337	-5.906
C	18.730	14.287	-5.115
H	17.717	14.154	-7.575
H	19.436	15.989	-6.022
H	17.855	14.692	-4.841
H	18.583	13.324	-5.352
H	19.369	14.339	-4.344
N	20.807	14.055	-7.983
C	22.083	13.549	-8.503
C	22.656	12.426	-7.626
O	23.840	12.413	-7.297
C	23.132	14.662	-8.604
C	22.635	15.841	-9.406
H	19.950	14.135	-8.575
H	21.923	13.174	-9.426
H	23.361	14.983	-7.687
H	23.949	14.304	-9.051
C	22.487	17.152	-9.107
N	22.236	15.691	-10.717
C	21.865	16.892	-11.201
N	21.996	17.783	-10.238
H	22.220	14.842	-11.230
H	22.692	17.591	-8.237
H	21.551	17.062	-12.125
N	21.809	11.434	-7.366
C	22.179	10.360	-6.464
C	21.332	9.153	-6.843
O	20.309	9.304	-7.543
C	22.018	10.805	-5.034
O	20.717	11.227	-4.744
H	20.897	11.498	-7.848
H	23.131	10.128	-6.623
H	22.257	10.040	-4.425
H	22.655	11.564	-4.855
H	20.501	12.056	-5.265
N	21.739	7.994	-6.314
C	20.990	6.754	-6.507
C	21.149	5.912	-5.250
O	22.250	5.843	-4.702
C	21.476	6.009	-7.727
H	22.617	8.054	-5.765
H	20.027	6.974	-6.627
H	22.411	5.683	-7.576
H	20.880	5.224	-7.906
H	21.465	6.617	-8.523
N	20.071	5.205	-4.903
C	20.127	4.221	-3.838
C	19.817	2.841	-4.395
O	18.894	2.679	-5.213
C	19.139	4.570	-2.734
C	19.276	3.632	-1.547
O	19.389	5.924	-2.320
H	19.217	5.419	-5.450
H	21.057	4.208	-3.459
H	18.193	4.525	-3.102
H	18.507	2.988	-1.530
H	20.133	3.116	-1.617
H	19.280	4.159	-0.694
H	20.376	6.113	-2.329
N	20.556	1.857	-3.890
C	20.225	0.456	-4.067
C	19.984	-0.174	-2.709
O	20.762	0.042	-1.773
C	21.313	-0.370	-4.772
C	21.667	0.174	-6.143
O	22.494	-0.446	-3.938
H	21.395	2.184	-3.355
H	19.381	0.389	-4.602
H	20.971	-1.321	-4.914
H	21.114	-0.282	-6.845
H	21.489	1.161	-6.176
H	22.638	0.011	-6.336
H	22.619	0.417	-3.443
N	18.951	-1.012	-2.678
C	18.560	-1.857	-1.556
C	18.812	-3.314	-1.926
O	18.325	-3.777	-2.964
C	17.067	-1.674	-1.233
C	16.657	-0.350	-0.662
C	16.384	-0.076	0.647
C	16.376	0.876	-1.382
C	15.960	1.834	-0.430
C	16.404	1.265	-2.728
N	15.957	1.221	0.795
C	15.582	3.129	-0.784
C	16.026	2.546	-3.076
C	15.592	3.458	-2.119
H	18.399	-1.015	-3.596
H	19.152	-1.635	-0.783
H	16.540	-1.808	-2.078
H	16.796	-2.380	-0.570
H	16.481	-0.731	1.401
H	15.687	1.650	1.657
H	16.698	0.618	-3.436
H	15.313	3.790	-0.076
H	16.066	2.818	-4.037
H	15.285	4.362	-2.409
N	19.458	-4.030	-1.014
C	19.722	-5.437	-1.178
C	19.142	-6.115	0.062
O	19.409	-5.681	1.180
C	21.204	-5.670	-1.329
O	21.483	-7.054	-1.464
H	19.755	-3.481	-0.172
H	19.221	-5.768	-1.964
H	21.532	-5.198	-2.142
H	21.676	-5.327	-0.521
H	22.371	-7.173	-1.906
N	18.316	-7.141	-0.144
C	17.725	-7.773	1.015
C	16.902	-8.999	0.653
O	17.171	-9.713	-0.330
H	18.159	-7.408	-1.108
H	18.457	-8.045	1.664
H	17.131	-7.107	1.499
N	16.021	-9.347	1.590
C	15.246	-10.563	1.400
C	13.839	-10.371	1.962
O	13.638	-9.658	2.944
C	15.995	-11.773	1.960
C	16.196	-11.751	3.451
C	16.886	-13.008	3.933
H	15.948	-8.726	2.392
H	15.160	-10.730	0.416
H	15.480	-12.604	1.720
H	16.899	-11.820	1.518
H	16.761	-10.967	3.693
H	15.309	-11.684	3.900
N	17.896	-12.825	4.785
O	16.508	-14.120	3.544
H	18.403	-13.624	5.166
H	18.152	-11.879	5.047
N	12.896	-11.002	1.250
C	11.486	-11.031	1.589
C	11.204	-12.258	2.461
O	11.650	-13.374	2.166
C	10.668	-11.013	0.296
C	9.256	-11.514	0.459
C	8.255	-10.699	0.972
C	8.949	-12.837	0.163
C	6.958	-11.177	1.153
C	7.672	-13.334	0.370
C	6.661	-12.491	0.813
O	5.373	-12.990	1.004
H	13.285	-11.491	0.394
H	11.267	-10.216	2.126
H	10.632	-10.073	-0.036
H	11.131	-11.591	-0.373
H	8.463	-9.751	1.218
H	9.657	-13.436	-0.204

H	6.249	-10.579	1.523
H	7.475	-14.302	0.201
H	4.749	-12.498	0.405
N	10.518	-11.998	3.579
C	10.074	-13.018	4.516
C	8.550	-12.932	4.565
O	7.996	-11.914	5.000
C	10.699	-12.762	5.906
C	10.392	-13.905	6.871
C	12.205	-12.518	5.820
H	10.325	-10.971	3.722
H	10.318	-13.907	4.154
H	10.274	-11.942	6.282
H	11.243	-14.366	7.120
H	9.958	-13.538	7.692
H	9.776	-14.556	6.429
H	12.455	-11.789	6.455
H	12.687	-13.358	6.063
H	12.440	-12.250	4.887
N	7.868	-13.936	4.020
C	6.412	-13.913	3.926
C	5.724	-14.326	5.238
O	6.382	-14.745	6.190
H	8.452	-14.722	3.674
H	6.104	-12.981	3.676
H	6.111	-14.539	3.189
N	4.385	-14.212	5.279
C	3.640	-14.454	6.510
C	2.645	-13.339	6.830
O	2.480	-12.370	6.068
H	3.948	-13.944	4.381
H	3.138	-15.332	6.432
H	4.290	-14.549	7.283
N	1.985	-13.485	7.991
C	0.897	-12.586	8.363
C	1.465	-11.197	8.648
O	0.793	-10.206	8.335
C	0.113	-13.126	9.539
H	2.312	-14.271	8.578
H	0.256	-12.518	7.598
H	-0.859	-12.913	9.425
H	0.228	-14.119	9.595
H	0.444	-12.709	10.387
N	2.690	-11.157	9.225
C	3.493	-9.941	9.300
C	4.735	-10.105	8.404
O	5.853	-10.343	8.849
C	3.819	-9.563	10.750
C	2.582	-9.206	11.579
C	2.198	-10.270	12.588
O	1.101	-10.859	12.461
O	3.012	-10.514	13.499
H	3.001	-12.077	9.608
H	2.962	-9.159	8.983
H	4.244	-10.338	11.174
H	4.399	-8.774	10.732
H	2.779	-8.378	12.076
H	1.823	-9.091	10.960
N	4.486	-9.995	7.108
C	5.509	-10.085	6.091
C	6.433	-8.880	6.215
O	6.059	-7.791	6.692
C	4.845	-10.182	4.735
H	3.469	-9.835	6.883
H	6.048	-10.915	6.237
H	4.616	-11.136	4.537
H	4.007	-9.635	4.729
H	5.466	-9.840	4.028
N	7.676	-9.100	5.803
C	8.734	-8.124	5.999
C	9.675	-8.213	4.796
O	9.946	-9.321	4.318
C	9.484	-8.505	7.284
C	10.292	-7.420	7.964
C	10.685	-7.896	9.352
N	9.626	-7.626	10.310
C	9.401	-6.445	10.868
N	10.262	-5.459	10.715
N	8.328	-6.253	11.609
H	7.822	-10.019	5.335
H	8.340	-7.212	6.026
H	8.803	-8.849	7.946
H	10.109	-9.266	7.060
H	11.116	-7.245	7.451
H	9.743	-6.606	8.060
H	10.852	-8.873	9.307
H	11.509	-7.412	9.621
H	9.019	-8.391	10.570
H	11.087	-5.570	10.163
H	10.090	-4.569	11.161
H	7.668	-7.001	11.735
H	8.163	-5.370	12.047
N	10.237	-7.062	4.397
C	11.458	-7.043	3.570
C	12.575	-6.369	4.371
O	12.455	-5.196	4.691
C	11.218	-6.354	2.221
C	10.035	-6.981	1.472
C	12.490	-6.356	1.367
C	9.596	-6.216	0.267
H	9.750	-6.207	4.713
H	11.725	-7.993	3.451
H	10.995	-5.396	2.396
H	10.298	-7.910	1.201
H	9.269	-7.051	2.116
H	12.328	-5.831	0.535
H	13.234	-5.942	1.885
H	12.726	-7.295	1.131
H	10.351	-6.150	-0.389
H	8.823	-6.682	-0.170
H	9.313	-5.292	0.534
N	13.612	-7.146	4.712
C	14.777	-6.714	5.480
C	15.920	-6.356	4.525
O	16.284	-7.196	3.695
C	15.272	-7.823	6.391
C	14.260	-8.150	7.469
H	13.509	-8.144	4.361
H	14.524	-5.873	5.959
H	15.428	-8.638	5.846
H	16.114	-7.526	6.827
N	14.017	-9.433	7.675
O	13.637	-7.244	8.017
H	14.495	-10.130	7.137
H	13.353	-9.715	8.371
N	16.340	-5.087	4.529
C	17.311	-4.611	3.544
C	18.477	-3.854	4.183
O	18.376	-3.335	5.295
C	16.685	-3.679	2.500
C	15.474	-4.240	1.802
O	16.355	-2.431	3.117
H	15.930	-4.493	5.267
H	17.688	-5.413	3.053
H	17.369	-3.499	1.764
H	14.639	-3.997	2.306
H	15.407	-3.870	0.870
H	15.537	-5.242	1.747
H	15.928	-2.586	4.015
N	19.591	-3.821	3.427
C	20.682	-2.897	3.574
C	20.751	-2.092	2.290
O	20.319	-2.565	1.233
C	21.968	-3.694	3.844
C	21.823	-4.469	5.160
C	23.075	-4.934	5.860
H	19.583	-4.582	2.675
H	20.470	-2.269	4.315
H	22.102	-4.341	3.120
H	22.719	-3.069	3.937
H	21.289	-3.882	5.808
H	21.232	-5.286	4.975
N	23.246	-6.237	5.881
O	23.822	-4.145	6.446
H	24.086	-6.648	6.328
H	22.554	-6.846	5.458
N	21.157	-0.836	2.406
C	21.128	0.027	1.226
C	22.445	0.801	1.126
O	23.153	0.998	2.125
C	19.877	0.924	1.219
C	19.669	1.811	2.413
C	18.701	1.649	3.367
C	20.353	3.041	2.747
C	19.746	3.547	3.914
C	21.411	3.779	2.189
N	18.782	2.651	4.294
C	20.179	4.718	4.547
C	21.832	4.940	2.811
C	21.220	5.414	3.975
H	21.471	-0.543	3.330
H	21.062	-0.559	0.419
H	19.918	1.522	0.411
H	19.064	0.337	1.142
H	18.033	0.906	3.382
H	18.224	2.723	5.125
H	21.858	3.467	1.349
H	19.733	5.029	5.393
H	22.595	5.452	2.413
H	21.587	6.319	4.445
N	22.764	1.205	-0.111
C	23.893	2.059	-0.430
C	23.308	3.226	-1.203
O	22.718	2.983	-2.245
C	24.919	1.341	-1.324
C	25.623	0.140	-0.692
C	26.444	-0.646	-1.707
C	26.443	0.535	0.504
H	22.102	0.836	-0.850
H	24.287	2.400	0.408
H	24.440	1.016	-2.132
H	25.617	2.001	-1.573
H	24.930	-0.467	-0.286
H	27.142	-0.050	-2.104
H	26.885	-1.419	-1.252
H	25.843	-0.984	-2.431
H	25.854	0.951	1.203
H	26.892	-0.273	0.896
H	27.146	1.199	0.235
N	23.642	4.433	-0.778
C	23.250	5.658	-1.463
C	24.513	6.360	-1.955
O	25.370	6.722	-1.152
C	22.492	6.526	-0.462
C	21.732	7.744	-0.992
C	21.097	8.513	0.168
C	22.545	8.644	-1.873
H	24.218	4.436	0.101
H	22.693	5.421	-2.254
H	21.821	5.940	0.005
H	23.153	6.862	0.215
H	21.016	7.437	-1.614
H	21.870	8.853	0.863
H	20.604	9.305	-0.191
H	20.461	7.914	0.653
H	22.872	8.138	-2.677
H	21.987	9.419	-2.184
H	23.428	9.024	-1.322
N	24.649	6.450	-3.275
C	25.816	7.084	-3.864
C	25.401	8.420	-4.471
O	24.398	8.498	-5.174
C	26.467	6.259	-4.977
C	27.676	6.940	-5.571
O	26.881	4.996	-4.466
H	23.874	6.041	-3.824
H	26.486	7.261	-3.143
H	25.777	6.095	-5.715
H	27.453	7.888	-5.819
H	28.430	6.949	-4.907
H	27.978	6.452	-6.396
H	26.152	4.311	-4.581
N	26.138	9.459	-4.085
C	25.997	10.788	-4.654
C	27.109	11.006	-5.680

O	28.289	10.682	-5.442
C	26.147	11.876	-3.549
C	25.302	11.600	-2.279
C	25.828	13.264	-4.094
C	26.046	10.697	-1.291
H	27.071	9.267	-3.678
H	25.139	10.791	-5.163
H	27.220	11.922	-3.251
H	25.088	12.552	-1.745
H	24.323	11.150	-2.551
H	26.529	13.550	-4.896
H	25.928	14.023	-3.301
H	24.819	13.287	-4.505
H	27.129	10.931	-1.277
H	25.724	10.861	-0.260
H	25.857	9.631	-1.526
N	26.742	11.615	-6.821
C	27.739	12.059	-7.781
C	28.695	13.062	-7.131
O	28.261	13.988	-6.455
H	25.725	11.731	-6.941
H	28.266	11.271	-8.113
H	27.287	12.497	-8.563
N	29.997	12.809	-7.257
C	31.030	13.630	-6.650
C	32.157	13.812	-7.670
O	32.292	13.024	-8.616
C	31.691	13.031	-5.394
C	30.705	12.767	-4.272
O	32.378	11.851	-5.708
H	30.210	11.960	-7.837
H	30.651	14.528	-6.442
H	32.412	13.777	-4.986
H	30.068	13.658	-4.094
H	30.065	11.914	-4.537
H	31.255	12.518	-3.341
H	33.288	12.101	-6.056
N	33.030	14.789	-7.389
C	34.336	14.826	-8.034
C	35.160	13.625	-7.583
O	34.908	12.995	-6.543
C	35.088	16.122	-7.709
C	34.355	17.354	-8.186
O	35.258	16.133	-6.288
H	32.702	15.482	-6.702
H	34.207	14.765	-9.025
H	35.999	16.091	-8.138
H	33.365	17.187	-8.191
H	34.550	18.129	-7.577
H	34.648	17.592	-9.117
H	34.838	15.316	-5.878
N	36.216	13.354	-8.335
C	37.122	12.262	-7.985
C	37.800	12.516	-6.628
O	37.947	11.581	-5.859
C	38.105	12.026	-9.144
C	37.383	11.758	-10.469
C	38.285	11.590	-11.677
O	38.121	12.342	-12.655
O	39.147	10.709	-11.630
H	36.341	13.959	-9.162
H	36.611	11.396	-7.941
H	38.635	12.836	-9.256
H	38.634	11.236	-8.931
H	36.861	10.921	-10.365
H	36.782	12.526	-10.649
N	38.142	13.772	-6.282
C	38.737	14.108	-4.986
C	37.791	13.803	-3.815
O	38.251	13.531	-2.707
C	39.099	15.578	-4.942
H	37.944	14.483	-7.021
H	39.579	13.562	-4.864
H	40.054	15.683	-4.662
H	38.975	15.984	-5.849
H	38.510	16.052	-4.286
N	36.471	13.915	-4.038
C	35.484	13.734	-2.954
C	34.783	12.410	-3.119
O	33.856	12.099	-2.322
C	34.523	14.939	-2.879
C	35.223	16.159	-2.371
H	36.217	14.139	-5.017
H	35.988	13.653	-1.962
H	34.054	15.139	-3.856
H	33.697	14.717	-2.173
N	35.422	17.234	-3.151
O	35.527	16.243	-1.157
H	35.179	17.236	-4.162
H	35.923	18.050	-2.749
N	35.351	11.456	-3.914
C	34.749	10.129	-4.067
C	34.769	9.308	-2.767
O	33.877	8.472	-2.571
C	35.393	9.359	-5.208
H	36.162	11.788	-4.457
H	33.784	10.242	-4.355
H	36.157	8.817	-4.858
H	34.718	8.749	-5.624
H	35.730	10.000	-5.897
N	35.802	9.421	-1.876
C	35.848	8.699	-0.583
C	34.625	8.979	0.280
O	34.046	8.020	0.853
C	37.152	9.017	0.180
C	37.220	10.451	0.631
C	37.709	11.485	-0.068
C	36.766	10.965	1.832
C	36.996	12.311	1.782
C	36.171	10.373	2.948
N	37.566	12.648	0.615
C	36.681	13.138	2.861
C	35.841	11.175	3.987
C	36.115	12.567	3.953
H	36.620	9.991	-2.183
H	35.852	7.606	-0.793
H	37.240	8.349	1.064
H	38.025	8.793	-0.471
H	38.181	11.417	-1.047
H	37.877	13.595	0.298
H	35.979	9.306	2.980
H	36.905	14.192	2.829
H	35.353	10.751	4.857
H	35.860	13.166	4.819
N	34.072	10.234	0.336
C	32.781	10.542	1.001
C	31.659	10.642	-0.006
O	30.747	11.498	0.163
C	32.894	11.800	1.897
C	33.389	13.048	1.147
C	33.448	14.281	2.058
C	34.231	15.405	1.364
N	34.493	16.530	2.271
H	34.534	10.960	-0.252
H	32.493	9.703	1.671
H	31.907	12.014	2.364
H	33.587	11.574	2.728
H	34.410	12.857	0.763
H	32.729	13.265	0.280
H	32.414	14.620	2.287
H	33.946	14.013	3.014
H	35.203	15.000	0.989
H	33.651	15.751	0.473
H	35.072	17.245	1.768
H	33.602	16.983	2.575
H	35.030	16.218	3.114
N	31.499	9.654	-0.936
C	30.343	9.592	-1.849
C	29.275	8.655	-1.353
O	28.130	8.802	-1.815
C	30.745	9.257	-3.309
O	31.247	7.967	-3.453
H	32.179	8.867	-0.924
H	29.863	10.595	-1.905
H	29.868	9.277	-3.968
H	31.472	10.008	-3.687
H	32.193	7.998	-3.127
N	29.483	7.656	-0.438
C	28.500	6.544	-0.326
C	27.945	6.401	1.062
O	28.620	5.807	1.929
C	29.056	5.230	-0.915
C	28.039	4.111	-0.904
O	29.466	5.451	-2.265
H	30.419	7.539	0.007
H	27.649	6.723	-0.954
H	29.872	4.993	-0.370
H	28.498	3.227	-1.029
H	27.547	4.101	-0.029
H	27.376	4.240	-1.647
H	29.986	6.309	-2.339
N	26.712	6.888	1.403
C	26.021	6.428	2.619
C	25.699	4.928	2.601
O	25.233	4.339	1.565
C	24.751	7.241	2.940
C	24.956	8.725	3.313
C	23.585	9.378	3.454
C	25.689	8.917	4.636
H	26.240	7.542	0.750
H	26.703	6.587	3.463
H	24.089	7.195	2.066
H	24.219	6.746	3.784
H	25.518	9.248	2.509
H	22.933	8.757	4.101
H	23.658	10.408	3.849
H	23.084	9.414	2.488
H	26.718	8.523	4.576
H	25.770	9.987	4.903
H	25.150	8.394	5.455
N	25.733	4.283	3.804
C	25.157	2.966	4.070
C	24.100	3.005	5.111
O	24.201	3.764	6.113
C	26.236	1.938	4.387
C	26.984	2.203	5.690
C	25.771	0.507	4.301
H	26.098	4.793	4.628
H	24.643	2.624	3.191
H	26.910	2.000	3.566
H	27.464	3.201	5.669
H	26.305	2.129	6.563
H	27.775	1.443	5.793
H	25.304	0.329	3.435
H	26.557	-0.104	4.391
H	25.139	0.356	5.061
N	23.034	2.180	4.971
C	22.011	2.090	6.000
C	21.219	0.807	5.857
O	21.608	-0.097	5.127
H	23.016	1.655	4.088
H	22.459	2.121	7.011
H	21.389	2.878	5.925
N	20.174	0.701	6.674
C	19.318	-0.477	6.690
C	17.879	0.003	6.835
O	17.638	0.994	7.511
C	19.726	-1.451	7.784
C	19.938	-0.792	9.091
C	19.164	-0.618	10.197
N	21.173	-0.249	9.373
C	21.144	0.269	10.602
N	19.927	0.084	11.115
H	20.025	1.526	7.294
H	19.405	-0.948	5.819
H	19.008	-2.147	7.887
H	20.579	-1.908	7.512
H	21.958	-0.241	8.763
H	18.233	-0.932	10.325
H	21.956	0.788	11.106
N	16.974	-0.624	6.077
C	15.551	-0.336	6.076
C	14.800	-1.662	6.270
O	15.093	-2.663	5.614
C	15.088	0.335	4.785

C	15.399	1.819	4.589
O	15.838	2.463	5.569
O	15.213	2.325	3.424
H	17.401	-1.373	5.458
H	15.354	0.224	6.875
H	15.498	-0.175	4.007
H	14.081	0.216	4.717
N	13.790	-1.668	7.158
C	12.871	-2.808	7.234
C	11.502	-2.338	6.745
O	10.986	-1.354	7.247
C	12.732	-3.386	8.647
C	11.975	-4.698	8.666
O	14.022	-3.642	9.177
H	13.726	-0.833	7.753
H	13.197	-3.520	6.622
H	12.269	-2.695	9.240
H	11.006	-4.533	8.870
H	12.047	-5.149	7.772
H	12.357	-5.305	9.368
H	14.664	-2.906	8.926
N	10.927	-3.045	5.768
C	9.664	-2.701	5.169
C	8.578	-3.654	5.670
O	8.829	-4.854	5.771
C	9.740	-2.796	3.655
C	10.647	-1.759	3.024
C	12.000	-2.016	2.862
C	10.156	-0.515	2.670
C	12.818	-1.056	2.287
C	10.983	0.449	2.113
C	12.317	0.168	1.933
H	11.493	-3.887	5.476
H	9.411	-1.787	5.497
H	10.095	-3.687	3.417
H	8.832	-2.660	3.289
H	12.384	-2.880	3.153
H	9.186	-0.305	2.817
H	13.798	-1.276	2.134
H	10.599	1.345	1.847
H	12.929	0.865	1.539
N	7.403	-3.110	6.012
C	6.265	-3.965	6.299
C	5.049	-3.452	5.532
O	5.065	-2.365	4.959
C	5.983	-4.047	7.808
C	7.156	-4.535	8.621
O	5.602	-2.761	8.281
H	7.388	-2.085	6.049
H	6.468	-4.892	5.981
H	5.207	-4.690	7.961
H	7.223	-5.536	8.567
H	8.009	-4.135	8.273
H	7.044	-4.270	9.584
H	6.189	-2.047	7.880
N	3.937	-4.197	5.635
C	2.684	-3.858	4.962
C	1.858	-2.749	5.628
O	0.979	-2.216	4.964
C	1.796	-5.103	4.879
C	2.399	-6.251	4.100
C	2.753	-5.876	2.679
C	1.812	-6.475	1.657
N	2.217	-6.082	0.284
H	4.049	-5.042	6.241
H	2.899	-3.564	4.004
H	1.612	-5.414	5.812
H	0.935	-4.839	4.442
H	3.234	-6.564	4.571
H	1.743	-7.016	4.081
H	2.725	-4.877	2.591
H	3.683	-6.195	2.481
H	1.835	-7.471	1.735
H	0.884	-6.147	1.833
H	1.566	-6.457	-0.376
H	2.227	-5.084	0.212
H	3.131	-6.439	0.091
N	2.145	-2.377	6.887
C	1.328	-1.448	7.660
C	2.104	-0.155	7.942
O	3.313	-0.181	8.175
C	0.871	-2.135	8.965
C	0.287	-3.520	8.684
C	1.990	-2.227	9.999
H	3.018	-2.820	7.266
H	0.521	-1.207	7.114
H	0.137	-1.586	9.356
H	0.957	-4.218	8.932
H	-0.543	-3.645	9.226
H	0.068	-3.595	7.712
H	1.950	-3.119	10.447
H	2.871	-2.118	9.540
H	1.870	-1.503	10.677
N	1.422	1.002	7.900
C	2.111	2.265	8.147
C	2.606	2.277	9.593
O	1.930	1.764	10.480
C	1.247	3.477	7.769
C	1.183	3.734	6.267
C	1.267	5.186	5.837
C	-0.080	5.782	5.486
N	0.021	7.236	5.230
H	0.423	0.915	7.689
H	2.899	2.333	7.538
H	0.323	3.314	8.099
H	1.633	4.282	8.209
H	1.941	3.218	5.819
H	0.315	3.338	5.908
H	1.667	5.721	6.585
H	1.863	5.251	5.033
H	-0.435	5.325	4.666
H	-0.715	5.622	6.247
H	0.981	7.488	5.099
H	-0.498	7.469	4.406
H	-0.351	7.741	6.010
N	3.817	2.812	9.880
C	4.450	2.615	11.181
C	4.100	3.745	12.162
O	3.421	3.440	13.130
C	5.944	2.588	10.815
C	6.057	3.403	9.515
C	4.647	3.650	9.001
O	4.379	4.915	12.156
H	4.198	1.720	11.574
H	6.498	3.012	11.531
H	6.260	1.653	10.659
H	6.530	4.272	9.704
H	6.601	2.887	8.842
H	4.398	4.612	9.088
H	4.558	3.364	8.048
C	25.772	14.246	4.016
N	24.127	13.744	1.495
O	18.970	12.659	0.279
S	17.373	6.368	0.497
C	24.876	13.178	4.579
N	20.211	13.265	-1.694
O	13.318	5.630	2.540
C	26.931	14.050	3.226
N	15.036	7.340	2.509
O	24.440	18.527	2.536
C	27.592	12.753	2.800
N	15.656	5.117	3.059
O	23.954	18.430	0.201
C	27.470	15.298	3.001
N	23.923	16.428	1.498
C	28.765	15.539	2.262
C	26.645	16.268	3.582
C	26.985	17.740	3.696
C	25.557	15.593	4.201
C	24.390	16.194	4.948
C	22.963	15.737	0.697
C	23.052	14.286	0.732
C	21.879	16.370	-0.076
C	22.146	13.436	-0.060
C	20.993	15.530	-0.910
C	21.109	14.060	-0.905
C	22.358	11.988	-0.039
C	24.393	12.338	1.451
C	23.469	11.442	0.739
C	19.191	12.484	-1.075
C	18.293	11.602	-1.900
C	17.163	10.880	-1.113
C	17.711	9.752	-0.225
C	16.622	9.076	0.648
C	17.245	7.935	1.518
C	16.449	7.395	2.719
C	17.824	5.431	1.849
C	16.912	5.849	3.092
C	14.540	6.010	2.690
C	24.144	17.817	1.386
C	24.058	19.772	-0.080
C	23.056	20.578	0.744
C	25.480	20.265	0.164
C	23.723	19.946	-1.555
Ir	25.483	15.204	2.055
H	25.229	12.164	4.334
H	23.856	13.304	4.165
H	24.845	13.249	5.689
H	20.452	13.067	-2.690
H	14.884	7.570	1.547
H	27.602	12.670	1.688
H	27.107	11.858	3.235
H	28.647	12.731	3.148
H	15.738	4.444	2.324
H	28.958	16.622	2.122
H	28.709	15.073	1.253
H	29.609	15.084	2.819
H	27.023	18.215	2.697
H	26.242	18.301	4.295
H	27.972	17.862	4.183
H	24.359	17.296	4.848
H	23.447	15.803	4.513
H	24.451	15.921	6.023
H	21.748	17.442	-0.066
H	20.254	15.999	-1.529
H	21.758	11.335	-0.656
H	25.284	11.920	1.884
H	23.711	10.395	0.636
H	17.826	12.239	-2.680
H	18.921	10.851	-2.426
H	16.447	10.433	-1.841
H	16.582	11.604	-0.504
H	18.185	8.979	-0.874
H	18.490	10.174	0.442
H	15.830	8.688	-0.032
H	16.162	9.838	1.315
H	18.155	8.416	1.809
H	16.663	8.089	3.505
H	17.586	4.414	1.617
H	18.829	5.702	2.095
H	17.429	5.698	4.016
H	23.035	21.634	0.403
H	22.040	20.147	0.633
H	23.328	20.578	1.817
H	25.584	21.326	-0.145
H	26.200	19.655	-0.419
H	25.750	20.186	1.234
H	23.790	21.012	-1.858
H	22.698	19.577	-1.761
H	24.426	19.363	-2.183
N	26.151	15.213	0.126
C	26.721	16.403	-0.384
O	27.187	17.356	0.501
C	26.586	16.747	-1.820
H	26.010	17.688	-1.867
H	25.993	15.956	-2.302
C	27.946	16.904	-2.523
H	28.343	15.905	-2.821
H	28.680	17.350	-1.814
C	27.878	17.832	-3.752
H	27.386	18.796	-3.495
H	28.930	18.092	-4.009
C	27.211	17.197	-4.998
C	25.679	17.396	-4.997
C	27.795	17.825	-6.283

H	27.438	16.106	-5.023
C	25.053	16.752	-6.237
H	25.439	18.482	-4.982
H	25.215	16.932	-4.111
C	27.152	17.237	-7.544
H	27.649	18.929	-6.280
H	28.890	17.634	-6.340
C	25.630	17.374	-7.513
H	23.953	16.897	-6.213
H	25.260	15.657	-6.234
H	27.436	16.167	-7.647
H	27.548	17.774	-8.436
H	25.217	16.854	-8.402
H	25.354	18.449	-7.577
N	25.294	11.144	-20.758
C	26.171	9.959	-20.534
C	25.509	8.678	-21.019
O	26.019	7.599	-20.749
C	26.476	9.693	-19.056
C	26.762	10.932	-18.238
O	27.754	11.629	-18.546
O	25.971	11.203	-17.327
H	24.387	11.074	-21.178
H	26.994	10.106	-21.093
H	25.684	9.222	-18.662
H	27.272	9.087	-19.013
N	24.357	8.783	-21.685
C	23.702	7.593	-22.199
C	24.720	6.846	-23.042
O	24.889	5.636	-22.918
C	22.470	7.985	-23.013
C	21.889	6.861	-23.829
C	20.566	7.246	-24.466
O	19.907	6.355	-25.001
O	20.211	8.450	-24.425
H	23.997	9.739	-21.793
H	23.401	7.017	-21.447
H	21.779	8.289	-22.378
H	22.739	8.703	-23.632
H	22.512	6.644	-24.555
H	21.718	6.097	-23.238
N	25.428	7.634	-23.860
C	26.397	7.130	-24.817
C	27.623	6.539	-24.115
O	28.090	5.446	-24.468
C	26.767	8.277	-25.745
H	25.216	8.657	-23.745
H	25.967	6.402	-25.369
H	27.118	7.911	-26.606
H	25.957	8.835	-25.927
H	27.470	8.843	-25.314
N	28.130	7.254	-23.094
C	29.311	6.820	-22.375
C	29.052	5.573	-21.524
O	29.959	4.775	-21.334
H	27.604	8.123	-22.880
H	30.056	6.618	-23.032
H	29.636	7.566	-21.771
N	27.834	5.419	-20.975
C	27.530	4.251	-20.137
C	27.242	2.996	-20.978
O	27.793	1.928	-20.716
C	26.401	4.587	-19.142
C	26.892	5.647	-18.152
C	25.903	3.331	-18.436
C	25.805	6.296	-17.358
H	27.161	6.166	-21.188
H	28.366	4.056	-19.613
H	25.646	4.971	-19.655
H	27.545	5.211	-17.529
H	27.383	6.350	-18.674
H	25.144	3.568	-17.832
H	25.593	2.669	-19.115
H	26.645	2.935	-17.899
H	25.315	5.605	-16.823
H	26.198	6.975	-16.734
H	25.159	6.752	-17.974
N	26.427	3.147	-22.033
C	26.046	2.047	-22.901
C	27.277	1.350	-23.448
O	28.166	1.981	-24.003
C	25.148	2.514	-24.063
C	24.779	1.362	-24.972
O	23.962	3.127	-23.545
H	26.093	4.130	-22.170
H	25.516	1.381	-22.362
H	25.655	3.212	-24.610
H	25.593	0.810	-25.176
H	24.094	0.778	-24.527
H	24.399	1.711	-25.833
H	24.148	4.078	-23.277
N	27.299	0.028	-23.333
C	28.411	-0.750	-23.820
C	28.802	-1.884	-22.898
O	28.023	-2.309	-22.031
H	26.462	-0.381	-22.871
H	28.182	-1.132	-24.738
H	29.217	-0.140	-23.958
N	30.020	-2.365	-23.129
C	30.567	-3.493	-22.411
C	31.625	-2.991	-21.429
O	32.532	-2.243	-21.771
C	31.052	-4.568	-23.391
C	31.678	-5.733	-22.664
O	29.914	-5.046	-24.138
H	30.545	-1.851	-23.878
H	29.838	-3.931	-21.885
H	31.704	-4.151	-24.047
H	31.273	-6.596	-22.980
H	32.667	-5.757	-22.836
H	31.524	-5.647	-21.675
H	29.115	-5.126	-23.535
N	31.477	-3.401	-20.170
C	32.404	-3.060	-19.112
C	32.959	-4.300	-18.441
O	32.336	-5.349	-18.410
C	31.653	-2.226	-18.103
C	31.157	-0.926	-18.607
C	29.978	-0.675	-19.225
C	31.836	0.325	-18.496
C	30.991	1.288	-19.046
C	33.047	0.712	-17.934
N	29.860	0.653	-19.458
C	31.309	2.639	-19.057
C	33.375	2.043	-17.946
C	32.525	2.988	-18.527
H	30.624	-3.995	-20.016
H	33.180	-2.565	-19.528
H	30.857	-2.744	-17.779
H	32.256	-2.034	-17.324
H	29.299	-1.368	-19.471
H	29.062	1.109	-19.872
H	33.664	0.039	-17.530
H	30.676	3.313	-19.435
H	34.233	2.342	-17.535
H	32.819	3.948	-18.553
N	34.176	-4.195	-17.907
C	34.826	-5.312	-17.253
C	35.328	-4.887	-15.880
O	35.853	-3.791	-15.747
C	36.042	-5.755	-18.086
C	35.716	-6.041	-19.530
C	35.712	-5.012	-20.473
C	35.325	-7.314	-19.943
C	35.382	-5.259	-21.804
C	34.977	-7.575	-21.267
C	35.024	-6.545	-22.200
O	34.665	-6.714	-23.524
H	34.607	-3.251	-18.003
H	34.152	-6.045	-17.124
H	36.719	-5.026	-18.052
H	36.410	-6.584	-17.675
H	35.949	-4.082	-20.191
H	35.294	-8.058	-19.275
H	35.403	-4.516	-22.473
H	34.697	-8.490	-21.542
H	34.217	-5.884	-23.847
N	35.259	-5.762	-14.881
C	35.757	-5.385	-13.565
C	37.104	-6.038	-13.335
O	37.661	-6.678	-14.230
C	34.779	-5.571	-12.397
C	34.457	-6.985	-12.002
H	34.848	-6.670	-15.109
H	35.905	-4.395	-13.577
H	35.166	-5.090	-11.592
H	33.915	-5.098	-12.641
N	33.427	-7.113	-11.175
O	35.131	-7.938	-12.400
H	32.915	-6.322	-10.857
H	33.154	-8.033	-10.861
N	37.630	-5.779	-12.135
C	38.951	-6.230	-11.753
C	38.972	-7.761	-11.637
O	40.041	-8.338	-11.764
C	39.383	-5.425	-10.515
C	38.793	-5.883	-9.169
C	37.379	-5.441	-8.832
H	37.004	-5.227	-11.506
H	39.597	-5.976	-12.478
H	40.387	-5.468	-10.438
H	39.120	-4.460	-10.650
H	38.824	-6.902	-9.146
H	39.416	-5.559	-8.430
N	36.927	-5.838	-7.649
O	36.699	-4.746	-9.584
H	35.987	-5.571	-7.339
H	37.520	-6.408	-7.056
N	37.824	-8.427	-11.386
C	37.782	-9.888	-11.231
C	37.693	-10.636	-12.569
O	37.633	-11.862	-12.542
C	36.541	-10.305	-10.417
C	36.518	-9.904	-8.941
C	35.155	-10.219	-8.313
C	37.656	-10.586	-8.184
H	36.986	-7.819	-11.314
H	38.630	-10.183	-10.761
H	35.744	-9.892	-10.852
H	36.474	-11.299	-10.460
H	36.694	-8.923	-8.865
H	34.978	-11.199	-8.386
H	35.166	-9.949	-7.352
H	34.445	-9.712	-8.798
H	38.531	-10.311	-8.582
H	37.626	-10.313	-7.223
H	37.553	-11.578	-8.254
N	37.472	-9.935	-13.688
C	37.156	-10.602	-14.942
C	35.660	-10.652	-15.292
O	35.299	-11.094	-16.386
H	37.545	-8.910	-13.579
H	37.653	-10.138	-15.707
H	37.516	-11.559	-14.916
N	34.765	-10.159	-14.426
C	33.349	-10.143	-14.775
C	33.063	-9.169	-15.920
O	33.802	-8.202	-16.104
C	32.544	-9.830	-13.526
O	32.791	-10.840	-12.557
H	35.150	-9.813	-13.542
H	33.092	-11.067	-15.087
H	32.832	-8.959	-13.162
H	31.582	-9.829	-13.750
H	33.710	-10.725	-12.185
N	31.963	-9.426	-16.646
C	31.486	-8.650	-17.777
C	30.079	-8.125	-17.538
O	29.137	-8.875	-17.256
C	31.355	-9.477	-19.077
C	31.466	-8.631	-20.317
O	32.339	-10.498	-19.122
H	31.446	-10.295	-16.300
H	32.101	-7.859	-17.907
H	30.429	-9.916	-19.088

H	30.650	-8.053	-20.418
H	32.276	-8.038	-20.263
H	31.549	-9.215	-21.131
H	32.967	-10.423	-18.337
N	29.946	-6.816	-17.714
C	28.729	-6.053	-17.498
C	28.354	-5.487	-18.868
O	29.105	-4.671	-19.396
C	29.100	-5.010	-16.420
C	28.116	-3.922	-16.158
C	26.782	-4.219	-15.930
C	28.551	-2.612	-15.972
C	25.871	-3.207	-15.645
C	27.645	-1.601	-15.706
C	26.301	-1.897	-15.542
H	30.849	-6.350	-18.042
H	28.019	-6.693	-17.253
H	29.248	-5.513	-15.565
H	29.965	-4.590	-16.706
H	26.466	-5.161	-15.968
H	29.527	-2.405	-16.031
H	24.899	-3.440	-15.517
H	27.969	-0.642	-15.633
H	25.644	-1.169	-15.350
N	27.243	-5.929	-19.464
C	26.784	-5.409	-20.747
C	25.501	-4.627	-20.516
O	24.535	-5.141	-19.972
C	26.606	-6.573	-21.737
C	27.901	-7.386	-21.813
C	26.133	-6.047	-23.086
C	27.792	-8.672	-22.624
H	26.739	-6.672	-18.934
H	27.471	-4.752	-21.046
H	25.891	-7.165	-21.390
H	28.613	-6.812	-22.217
H	28.178	-7.623	-20.881
H	25.966	-6.814	-23.703
H	25.288	-5.529	-22.964
H	26.834	-5.453	-23.476
H	27.531	-8.446	-23.560
H	28.675	-9.135	-22.621
H	27.100	-9.259	-22.212
N	25.520	-3.334	-20.789
C	24.415	-2.474	-20.390
C	23.998	-1.532	-21.519
O	24.859	-1.085	-22.282
C	24.808	-1.697	-19.105
C	25.931	-0.694	-19.339
C	23.599	-1.041	-18.455
H	26.357	-2.998	-21.292
H	23.623	-3.059	-20.169
H	25.147	-2.368	-18.442
H	25.541	0.215	-19.483
H	26.533	-0.676	-18.541
H	26.455	-0.963	-20.147
H	23.236	-0.338	-19.066
H	22.898	-1.734	-18.286
H	23.875	-0.624	-17.589
N	22.698	-1.198	-21.536
C	22.123	-0.096	-22.295
C	21.507	0.918	-21.348
O	20.698	0.572	-20.479
C	21.074	-0.590	-23.306
C	20.475	0.522	-24.136
O	21.729	-1.516	-24.177
H	22.105	-1.833	-20.924
H	22.857	0.355	-22.810
H	20.344	-1.082	-22.807
H	20.206	1.288	-23.544
H	21.145	0.851	-24.807
H	19.663	0.189	-24.625
H	22.201	-2.227	-23.643
N	21.923	2.177	-21.499
C	21.301	3.285	-20.790
C	20.255	3.898	-21.709
O	20.595	4.316	-22.819
C	22.325	4.308	-20.371
H	22.719	2.291	-22.159
H	20.847	2.937	-19.975
H	21.906	5.218	-20.340
H	22.682	4.082	-19.462
H	23.084	4.320	-21.026
N	19.003	3.912	-21.245
C	17.930	4.628	-21.904
C	17.927	6.118	-21.585
O	18.539	6.589	-20.616
H	18.871	3.360	-20.365
H	18.008	4.506	-22.910
H	17.037	4.230	-21.628
N	17.176	6.867	-22.405
C	17.134	8.320	-22.303
C	16.422	8.762	-21.017
O	16.635	9.874	-20.526
C	16.456	8.883	-23.542
H	16.636	6.323	-23.108
H	18.076	8.674	-22.280
H	16.409	9.880	-23.475
H	16.980	8.630	-24.355
H	15.530	8.512	-23.615
N	15.593	7.853	-20.488
C	14.683	8.030	-19.367
C	15.341	7.741	-18.003
O	14.719	8.004	-16.978
C	13.520	7.043	-19.557
C	13.885	5.551	-19.432
O	14.751	5.020	-20.236
O	13.303	4.900	-18.504
H	15.663	6.907	-21.003
H	14.406	9.005	-19.351
H	12.822	7.250	-18.867
H	13.133	7.193	-20.469
N	16.562	7.180	-17.999
C	17.263	6.774	-16.778
C	17.334	5.257	-16.533
O	17.846	4.841	-15.484
H	16.972	7.058	-18.956
H	18.216	7.146	-16.801
H	16.812	7.214	-15.972
N	16.854	4.423	-17.463
C	16.894	2.965	-17.313
C	18.261	2.373	-17.645
O	18.984	2.858	-18.517
C	15.809	2.308	-18.147
H	16.451	4.898	-18.299
H	16.668	2.735	-16.352
H	14.973	2.223	-17.604
H	15.623	2.866	-18.956
H	16.110	1.399	-18.435
N	18.638	1.323	-16.892
C	19.756	0.462	-17.249
C	19.179	-0.930	-17.418
O	18.375	-1.380	-16.612
C	20.859	0.405	-16.181
C	21.494	1.740	-15.796
C	22.427	1.600	-14.599
C	22.233	2.363	-16.983
H	18.057	1.187	-16.036
H	20.127	0.760	-18.116
H	20.461	0.014	-15.356
H	21.583	-0.184	-16.526
H	20.766	2.395	-15.572
H	23.160	0.957	-14.821
H	22.822	2.492	-14.378
H	21.911	1.260	-13.813
H	21.587	2.519	-17.729
H	22.637	3.233	-16.701
H	22.954	1.741	-17.287
N	19.577	-1.597	-18.489
C	19.059	-2.913	-18.779
C	20.219	-3.651	-19.414
O	21.028	-3.041	-20.099
C	17.789	-2.878	-19.644
C	16.696	-1.980	-19.108
O	18.169	-2.416	-20.935
H	20.272	-1.104	-19.080
H	18.825	-3.363	-17.933
H	17.428	-3.820	-19.732
H	15.801	-2.290	-19.442
H	16.693	-2.002	-18.104
H	16.847	-1.035	-19.412
H	18.825	-1.655	-20.861
N	20.368	-4.942	-19.139
C	21.401	-5.697	-19.810
C	21.646	-7.018	-19.111
O	20.705	-7.633	-18.618
H	19.717	-5.327	-18.446
H	21.128	-5.871	-20.766
H	22.258	-5.164	-19.824
N	22.897	-7.449	-19.120
C	23.272	-8.763	-18.642
C	24.610	-8.675	-17.933
O	25.457	-7.826	-18.212
C	23.359	-9.816	-19.760
C	22.078	-9.996	-20.550
O	24.402	-9.386	-20.620
H	23.588	-6.760	-19.501
H	22.584	-9.076	-17.983
H	23.618	-10.701	-19.352
H	22.287	-10.084	-21.528
H	21.600	-10.823	-20.244
H	21.477	-9.203	-20.415
H	24.514	-8.385	-20.572
N	24.754	-9.547	-16.969
C	25.927	-9.581	-16.141
C	26.470	-10.998	-16.152
O	25.736	-11.976	-15.997
C	25.612	-9.168	-14.705
C	26.838	-8.805	-13.919
C	27.625	-9.758	-13.315
C	27.227	-7.472	-13.817
C	28.761	-9.406	-12.593
C	28.371	-7.114	-13.132
C	29.140	-8.074	-12.514
O	30.234	-7.682	-11.808
H	23.950	-10.210	-16.855
H	26.621	-8.980	-16.535
H	25.009	-8.378	-14.734
H	25.162	-9.931	-14.254
H	27.383	-10.731	-13.390
H	26.667	-6.766	-14.249
H	29.303	-10.112	-12.134
H	28.644	-6.146	-13.084
H	30.869	-7.200	-12.413
N	27.776	-11.092	-16.326
C	28.437	-12.380	-16.321
C	29.550	-12.331	-15.293
O	30.523	-11.601	-15.501
C	28.969	-12.690	-17.714
C	29.676	-14.025	-17.739
C	29.723	-14.660	-19.120
O	30.099	-15.845	-19.186
O	29.373	-13.956	-20.119
H	28.269	-10.190	-16.462
H	27.788	-13.097	-16.079
H	28.209	-12.741	-18.320
H	29.616	-12.002	-17.948
H	30.608	-13.892	-17.446
H	29.193	-14.649	-17.147
N	29.385	-13.091	-14.195
C	30.354	-13.064	-13.115
C	31.420	-14.132	-13.334
O	31.091	-15.259	-13.683
C	29.723	-13.241	-11.769
O	30.710	-13.489	-10.772
H	28.532	-13.673	-14.199
H	30.811	-12.169	-13.122
H	29.225	-12.413	-11.518
H	29.097	-14.018	-11.790
H	30.600	-14.418	-10.424
N	32.678	-13.781	-13.037
C	33.797	-14.708	-13.164
C	34.004	-15.525	-11.881
O	34.833	-16.424	-11.882
C	35.043	-13.932	-13.527

H	32.782	-12.797	-12.711
H	33.607	-15.349	-13.920
H	34.834	-13.275	-14.253
H	35.380	-13.437	-12.724
H	35.753	-14.561	-13.847
N	33.246	-15.249	-10.794
C	33.423	-15.933	-9.517
C	32.056	-16.281	-8.939
O	31.024	-15.718	-9.351
C	34.204	-15.124	-8.451
C	35.672	-14.967	-8.815
C	33.540	-13.770	-8.153
H	32.532	-14.508	-10.958
H	33.915	-16.788	-9.685
H	34.166	-15.642	-7.596
H	35.852	-14.019	-9.078
H	36.240	-15.207	-8.027
H	35.894	-15.571	-9.581
H	33.055	-13.833	-7.284
H	34.247	-13.069	-8.100
H	32.900	-13.556	-8.887
N	32.101	-17.161	-7.929
C	30.918	-17.516	-7.165
C	30.050	-18.532	-7.908
O	30.461	-19.116	-8.924
H	33.046	-17.557	-7.749
H	31.193	-17.907	-6.275
H	30.370	-16.687	-6.982
N	28.830	-18.694	-7.388
C	27.864	-19.659	-7.899
C	27.035	-18.959	-8.971
O	25.923	-18.487	-8.722
C	27.036	-20.262	-6.755
C	25.935	-21.180	-7.243
H	28.628	-18.062	-6.575
H	28.376	-20.381	-8.352
H	27.650	-20.783	-6.167
H	26.625	-19.513	-6.240
N	25.052	-21.566	-6.334
O	25.832	-21.461	-8.438
H	25.129	-21.249	-5.388
H	24.301	-22.178	-6.591
N	27.628	-18.918	-10.166
C	27.136	-18.174	-11.305
C	27.675	-18.819	-12.582
O	28.873	-19.088	-12.644
C	27.613	-16.743	-11.192
H	28.517	-19.496	-10.205
H	26.138	-18.220	-11.325
H	27.373	-16.376	-10.292
H	28.607	-16.707	-11.307
H	27.180	-16.184	-11.901
N	26.797	-19.007	-13.581
C	27.169	-19.476	-14.914
C	26.430	-18.679	-15.983
O	25.225	-18.484	-15.887
C	26.850	-20.963	-15.088
C	26.827	-21.409	-16.538
C	26.762	-22.915	-16.756
O	26.878	-23.337	-17.957
O	26.598	-23.660	-15.732
H	25.805	-18.783	-13.314
H	28.160	-19.373	-15.044
H	27.551	-21.474	-14.628
H	25.959	-21.123	-14.710
H	26.026	-21.015	-16.972
H	27.656	-21.085	-16.978
N	27.180	-18.230	-17.003
C	26.610	-17.514	-18.124
C	26.109	-16.134	-17.640
O	26.625	-15.575	-16.656
C	25.524	-18.376	-18.820
O	25.344	-17.984	-20.175
H	28.196	-18.445	-16.908
H	27.326	-17.348	-18.799
H	25.812	-19.326	-18.790
H	24.669	-18.256	-18.329
H	25.517	-17.005	-20.262
N	25.134	-15.590	-18.382
C	24.644	-14.245	-18.201
C	23.375	-14.284	-17.365
O	22.534	-15.180	-17.531
C	24.381	-13.612	-19.571
C	25.644	-13.383	-20.386
C	25.328	-12.901	-21.783
N	25.610	-13.876	-22.829
C	25.057	-15.081	-22.941
N	24.377	-15.612	-21.941
N	25.227	-15.777	-24.050
H	24.757	-16.246	-19.116
H	25.332	-13.704	-17.712
H	23.787	-14.219	-20.085
H	23.942	-12.733	-19.428
H	26.208	-12.694	-19.935
H	26.151	-14.240	-20.457
H	24.352	-12.658	-21.816
H	25.869	-12.069	-21.955
H	26.292	-13.614	-23.539
H	24.247	-15.110	-21.086
H	23.982	-16.532	-22.036
H	25.754	-15.391	-24.812
H	24.831	-16.693	-24.138
N	23.248	-13.255	-16.527
C	22.077	-12.977	-15.717
C	21.493	-11.644	-16.128
O	22.248	-10.720	-16.455
C	22.479	-12.917	-14.235
C	23.038	-14.217	-13.755
C	24.339	-14.607	-14.015
C	22.219	-15.108	-13.093
C	24.794	-15.852	-13.637
C	22.688	-16.323	-12.652
C	23.970	-16.717	-12.948
O	24.366	-17.957	-12.509
H	24.111	-12.634	-16.504
H	21.393	-13.689	-15.892
H	23.168	-12.213	-14.134
H	21.669	-12.702	-13.708
H	24.961	-13.976	-14.486
H	21.258	-14.863	-12.929
H	25.737	-16.133	-13.864
H	22.090	-16.927	-12.111
H	24.585	-17.907	-11.536
N	20.163	-11.533	-16.032
C	19.465	-10.305	-16.291
C	19.878	-9.277	-15.232
O	19.935	-9.615	-14.043
C	17.941	-10.508	-16.331
C	17.213	-9.172	-16.354
C	17.535	-11.352	-17.545
H	19.687	-12.425	-15.751
H	19.749	-9.950	-17.181
H	17.674	-10.971	-15.489
H	16.235	-9.327	-16.492
H	17.356	-8.699	-15.485
H	17.569	-8.610	-17.101
H	16.539	-11.368	-17.611
H	17.925	-10.944	-18.368
H	17.883	-12.280	-17.426
N	20.160	-8.040	-15.674
C	20.260	-6.928	-14.726
C	19.344	-5.801	-15.138
O	18.997	-5.611	-16.316
C	21.699	-6.442	-14.596
C	22.295	-5.622	-15.734
C	21.967	-4.116	-15.652
C	23.787	-5.851	-15.702
H	20.292	-7.953	-16.685
H	19.980	-7.260	-13.820
H	21.757	-5.879	-13.762
H	22.285	-7.251	-14.473
H	21.950	-5.978	-16.616
H	22.328	-3.752	-14.797
H	22.389	-3.650	-16.426
H	20.978	-3.998	-15.682
H	23.985	-6.826	-15.828
H	24.227	-5.329	-16.436
H	24.159	-5.553	-14.820
N	18.911	-5.073	-14.122
C	18.178	-3.855	-14.346
C	18.640	-2.841	-13.315
O	18.917	-3.206	-12.160
C	16.681	-4.134	-14.265
C	16.238	-4.559	-12.897
O	15.972	-2.957	-14.634
H	19.142	-5.450	-13.184
H	18.397	-3.502	-15.253
H	16.444	-4.872	-14.929
H	16.454	-3.846	-12.221
H	15.245	-4.721	-12.884
H	16.704	-5.409	-12.625
H	16.551	-2.357	-15.199
N	18.631	-1.579	-13.714
C	18.916	-0.498	-12.779
C	18.511	0.862	-13.341
O	17.685	0.939	-14.247
H	18.412	-1.431	-14.713
H	18.418	-0.660	-11.915
H	19.904	-0.488	-12.567
N	19.009	1.918	-12.692
C	18.669	3.300	-12.970
C	19.946	4.136	-12.905
O	20.884	3.831	-12.155
C	17.675	3.863	-11.945
C	16.368	3.106	-11.743
C	15.466	3.213	-12.973
N	14.987	4.585	-13.132
C	14.473	5.063	-14.271
N	14.239	4.268	-15.290
N	14.174	6.334	-14.366
H	19.698	1.646	-11.930
H	18.292	3.368	-13.891
H	18.145	3.910	-11.052
H	17.444	4.806	-12.223
H	16.562	2.143	-11.586
H	15.877	3.491	-10.968
H	15.997	2.954	-13.765
H	14.696	2.607	-12.840
H	15.046	5.203	-12.347
H	14.420	3.285	-15.232
H	13.869	4.645	-16.150
H	14.309	6.950	-13.582
H	13.805	6.709	-15.222
N	19.968	5.238	-13.661
C	21.056	6.203	-13.592
C	20.458	7.598	-13.704
O	19.307	7.749	-14.126
C	22.128	5.951	-14.657
C	21.741	6.388	-16.064
C	20.822	5.671	-16.825
C	22.263	7.552	-16.626
C	20.434	6.097	-18.099
C	21.920	7.969	-17.908
C	20.990	7.252	-18.645
O	20.610	7.706	-19.918
H	19.148	5.341	-14.294
H	21.496	6.122	-12.701
H	22.953	6.449	-14.396
H	22.321	4.971	-14.683
H	20.425	4.826	-16.456
H	22.905	8.107	-16.089
H	19.757	5.573	-18.617
H	22.346	8.787	-18.299
H	19.772	7.241	-20.180
N	21.251	8.591	-13.287
C	20.876	10.003	-13.337
C	21.041	10.494	-14.773
O	22.164	10.619	-15.240
C	21.762	10.847	-12.427
C	21.431	12.343	-12.438
O	20.553	12.757	-13.218
O	22.057	13.069	-11.688
H	22.178	8.263	-12.916
H	19.905	10.061	-13.117
H	21.659	10.512	-11.492

H	22.710	10.735	-12.721
N	19.929	10.718	-15.485
C	20.020	11.036	-16.904
C	20.250	12.534	-17.136
O	20.380	12.945	-18.280
C	18.816	10.521	-17.667
O	17.594	10.808	-16.983
H	19.050	10.647	-14.960
H	20.812	10.543	-17.288
H	18.784	10.955	-18.565
H	18.892	9.531	-17.774
H	16.966	10.042	-17.100
N	20.328	13.350	-16.081
C	20.666	14.773	-16.210
C	21.651	15.160	-15.109
O	21.278	15.787	-14.109
C	19.413	15.622	-16.140
H	20.130	12.894	-15.166
H	21.102	14.920	-17.097
H	18.654	15.075	-15.785
H	19.568	16.403	-15.534
H	19.180	15.956	-17.054
N	22.914	14.691	-15.192
C	23.869	14.905	-14.102
C	24.288	16.360	-13.926
O	23.865	17.231	-14.684
C	25.054	14.014	-14.485
C	24.943	13.815	-15.971
C	23.463	13.874	-16.288
H	23.498	14.533	-13.228
H	25.925	14.460	-14.251
H	25.007	13.133	-14.004
H	25.440	14.538	-16.460
H	25.328	12.925	-16.235
H	23.300	14.312	-17.171
H	23.056	12.961	-16.286
N	25.076	16.622	-12.885
C	25.649	17.945	-12.684
C	26.808	18.166	-13.666
O	27.356	17.222	-14.234
C	26.069	18.115	-11.242
H	25.240	15.821	-12.249
H	24.947	18.638	-12.864
H	26.844	17.513	-11.041
H	26.340	19.065	-11.077
H	25.306	17.882	-10.637
N	27.169	19.441	-13.880
C	28.145	19.810	-14.902
C	29.384	20.444	-14.275
O	30.107	21.184	-14.937
C	27.518	20.753	-15.938
C	26.843	19.985	-17.058
O	26.609	21.617	-15.239
H	26.697	20.130	-13.261
H	28.429	18.975	-15.389
H	28.246	21.325	-16.348
H	27.508	19.387	-17.512
H	26.098	19.423	-16.688
H	26.465	20.624	-17.733
H	25.784	21.110	-14.971
N	29.633	20.121	-13.004
C	30.730	20.688	-12.243
C	31.884	19.689	-12.188
O	32.733	19.808	-11.305
C	30.266	21.066	-10.834
C	29.713	19.913	-10.004
O	29.595	18.782	-10.530
O	29.421	20.149	-8.824
H	28.962	19.419	-12.607
H	31.063	21.490	-12.744
H	31.049	21.459	-10.347
H	29.552	21.764	-10.921
N	31.878	18.700	-13.102
C	32.855	17.624	-13.121
C	32.514	16.447	-12.191
O	33.326	15.545	-12.026
H	31.099	18.782	-13.800
H	32.951	17.269	-14.072
H	33.769	17.990	-12.858
N	31.315	16.447	-11.603
C	30.853	15.344	-10.767
C	30.583	14.111	-11.617
O	30.119	14.202	-12.757
C	29.614	15.711	-10.014
O	29.908	16.619	-8.968
H	30.744	17.299	-11.793
H	31.584	15.118	-10.116
H	28.960	16.142	-10.633
H	29.208	14.890	-9.615
H	29.862	17.555	-9.316
N	30.733	12.931	-10.996
C	30.303	11.697	-11.622
C	28.775	11.580	-11.734
O	27.999	12.375	-11.191
H	31.170	12.989	-10.061
H	30.702	11.628	-12.553
H	30.651	10.904	-11.091
N	28.346	10.529	-12.450
C	26.941	10.270	-12.707
C	26.508	9.086	-11.842
O	26.966	7.981	-12.093
C	26.735	9.911	-14.181
C	25.311	9.542	-14.525
O	27.200	11.080	-14.876
H	29.118	9.917	-12.803
H	26.409	11.067	-12.433
H	27.329	9.146	-14.420
H	24.674	10.008	-13.904
H	25.102	9.815	-15.469
H	25.181	8.550	-14.439
H	26.767	11.142	-15.781
N	25.635	9.335	-10.874
C	25.159	8.277	-9.977
C	24.361	7.233	-10.743
O	23.536	7.568	-11.579
C	24.332	8.891	-8.891
H	25.323	10.321	-10.806
H	25.957	7.830	-9.556
H	23.791	9.654	-9.256
H	23.706	8.208	-8.504
H	24.924	9.238	-8.158
N	24.529	5.965	-10.345
C	23.777	4.873	-10.904
C	23.784	3.683	-9.952
O	24.492	3.675	-8.951
C	24.360	4.528	-12.277
C	25.873	4.341	-12.368
C	26.344	3.064	-11.725
C	26.301	4.358	-13.835
H	25.252	5.851	-9.597
H	22.822	5.162	-11.036
H	23.935	3.674	-12.582
H	24.112	5.264	-12.907
H	26.322	5.130	-11.922
H	25.915	2.276	-12.174
H	27.340	2.985	-11.809
H	26.098	3.059	-10.753
H	26.047	5.234	-14.244
H	27.291	4.235	-13.893
H	25.843	3.615	-14.323
N	22.977	2.685	-10.294
C	22.977	1.423	-9.596
C	22.205	0.392	-10.388
O	21.419	0.750	-11.268
H	22.352	2.898	-11.104
H	23.923	1.105	-9.467
H	22.553	1.534	-8.690
N	22.433	-0.869	-10.053
C	21.730	-1.951	-10.709
C	21.719	-3.167	-9.791
O	22.469	-3.219	-8.809
C	22.343	-2.313	-12.080
C	23.714	-2.920	-12.022
C	24.018	-4.247	-12.070
C	24.978	-2.235	-12.084
C	25.985	-3.209	-12.067
C	25.361	-0.892	-12.115
N	25.371	-4.419	-12.035
C	27.358	-2.902	-12.089
C	26.704	-0.581	-12.157
C	27.687	-1.570	-12.111
H	23.136	-1.005	-9.305
H	20.780	-1.669	-10.845
H	21.744	-2.971	-12.530
H	22.408	-1.482	-12.627
H	23.347	-4.987	-12.123
H	25.843	-5.309	-11.992
H	24.675	-0.165	-12.108
H	28.038	-3.628	-12.087
H	26.976	0.381	-12.221
H	28.649	-1.296	-12.094
N	20.852	-4.117	-10.160
C	20.633	-5.358	-9.443
C	20.725	-6.525	-10.413
O	20.188	-6.425	-11.511
C	19.235	-5.444	-8.839
C	19.006	-6.730	-8.060
O	19.059	-4.299	-7.992
H	20.326	-3.873	-11.048
H	21.352	-5.464	-8.753
H	18.546	-5.395	-9.581
H	18.672	-6.515	-7.139
H	18.327	-7.297	-8.531
H	19.866	-7.241	-7.986
H	18.204	-4.380	-7.469
N	21.365	-7.588	-9.930
C	21.297	-8.935	-10.479
C	20.733	-9.877	-9.422
O	21.255	-9.923	-8.302
C	22.681	-9.411	-10.954
C	22.594	-10.879	-11.359
C	23.231	-8.511	-12.059
H	21.955	-7.361	-9.073
H	20.666	-8.928	-11.255
H	23.300	-9.360	-10.184
H	23.150	-11.429	-10.737
H	21.642	-11.181	-11.312
H	22.933	-10.987	-12.293
H	23.727	-9.071	-12.721
H	22.471	-8.046	-12.511
H	23.849	-7.838	-11.655
N	19.665	-10.626	-9.771
C	19.296	-11.830	-9.021
C	19.964	-13.036	-9.694
O	19.892	-13.158	-10.908
C	17.807	-12.009	-8.961
H	19.147	-10.278	-10.595
H	19.643	-11.752	-8.092
H	17.349	-11.123	-9.069
H	17.504	-12.622	-9.695
H	17.543	-12.405	-8.078
N	20.624	-13.882	-8.904
C	21.550	-14.891	-9.408
C	20.799	-16.170	-9.769
O	21.185	-17.257	-9.298
C	22.661	-15.140	-8.389
C	23.513	-13.921	-8.227
C	23.515	-13.059	-7.173
C	24.412	-13.382	-9.207
C	24.971	-12.211	-8.645
C	24.857	-13.798	-10.468
N	24.405	-12.052	-7.397
C	25.949	-11.459	-9.315
C	25.816	-13.053	-11.125
C	26.348	-11.898	-10.558
H	20.419	-13.759	-7.882
H	21.963	-14.535	-10.251
H	22.258	-15.339	-7.517
H	23.238	-15.865	-8.713
H	22.946	-13.152	-6.355
H	24.623	-11.307	-6.763
H	24.486	-14.627	-10.890
H	26.331	-10.636	-8.891
H	26.130	-13.353	-12.025
H	27.035	-11.378	-11.067

N	19.735	-15.978	-10.550
C	18.969	-17.063	-11.171
C	18.969	-16.837	-12.680
O	18.667	-15.761	-13.180
C	17.524	-17.142	-10.636
C	16.621	-18.159	-11.346
C	15.149	-18.050	-10.978
C	14.258	-19.029	-11.718
N	14.178	-18.688	-13.156
H	19.486	-14.964	-10.686
H	19.417	-17.929	-10.974
H	17.566	-17.387	-9.667
H	17.109	-16.236	-10.732
H	16.718	-18.031	-12.344
H	16.946	-19.089	-11.121
H	15.052	-18.214	-9.991
H	14.835	-17.117	-11.181
H	14.633	-19.951	-11.615
H	13.340	-19.000	-11.318
H	14.219	-17.694	-13.268
H	14.944	-19.109	-13.644
H	13.316	-19.029	-13.534
N	19.236	-17.933	-13.407
C	19.104	-17.953	-14.839
C	18.774	-19.400	-15.236
O	18.320	-20.183	-14.406
C	20.371	-17.402	-15.503
C	21.594	-18.289	-15.379
H	19.543	-18.752	-12.837
H	18.312	-17.407	-15.084
H	20.177	-17.262	-16.480
H	20.580	-16.511	-15.086
N	22.727	-17.812	-15.893
O	21.567	-19.362	-14.761
H	22.734	-16.909	-16.325
H	23.564	-18.353	-15.846
N	19.077	-19.753	-16.487
C	18.681	-21.054	-17.011
C	19.740	-22.110	-16.774
O	19.623	-23.213	-17.317
C	18.321	-20.963	-18.490
C	17.052	-20.162	-18.693
H	19.598	-19.046	-17.028
H	17.855	-21.340	-16.502
H	19.061	-20.512	-18.969
H	18.178	-21.880	-18.837
N	16.847	-19.688	-19.910
O	16.257	-19.985	-17.764
H	17.508	-19.869	-20.641
H	16.028	-19.145	-20.108
N	20.720	-21.795	-15.928
C	21.761	-22.711	-15.554
C	21.795	-22.967	-14.059
O	22.094	-24.096	-13.656
C	23.096	-22.174	-16.054
C	23.166	-22.200	-17.551
C	23.431	-23.379	-18.237
C	22.853	-21.070	-18.287
C	23.468	-23.406	-19.624
C	22.890	-21.074	-19.665
C	23.215	-22.241	-20.329
O	23.183	-22.180	-21.680
H	20.656	-20.810	-15.561
H	21.594	-23.599	-16.018
H	23.191	-21.239	-15.748
H	23.817	-22.746	-15.694
H	23.599	-24.223	-17.725
H	22.592	-20.229	-17.805
H	23.673	-24.255	-20.108
H	22.685	-20.243	-20.181
H	24.081	-21.904	-22.029
N	21.571	-21.913	-13.252
C	21.880	-21.956	-11.833
C	20.838	-21.119	-11.102
O	20.224	-20.211	-11.659
C	23.218	-21.298	-11.462
C	24.438	-21.692	-12.265
C	25.246	-22.806	-11.649
N	26.440	-22.952	-12.468
C	27.678	-22.691	-12.061
N	28.668	-22.707	-12.940
N	27.911	-22.392	-10.790
H	21.166	-21.087	-13.733
H	21.817	-22.896	-11.512
H	23.099	-20.297	-11.543
H	23.402	-21.507	-10.490
H	24.149	-21.986	-13.187
H	25.041	-20.889	-12.370
H	25.499	-22.545	-10.743
H	24.720	-23.629	-11.684
H	26.321	-23.276	-13.420
H	28.512	-22.889	-13.905
H	29.614	-22.527	-12.624
H	27.144	-22.345	-10.144
H	28.840	-22.213	-10.476
N	20.654	-21.432	-9.839
C	19.986	-20.493	-8.943
C	20.738	-20.491	-7.625
O	20.598	-21.431	-6.844
C	18.489	-20.786	-8.796
C	17.744	-19.733	-8.009
H	21.004	-22.354	-9.545
H	20.084	-19.585	-9.341
H	18.091	-20.844	-9.712
H	18.388	-21.666	-8.331
N	16.428	-19.893	-7.859
O	18.379	-18.777	-7.562
H	15.990	-20.701	-8.265
H	15.894	-19.227	-7.356
N	21.443	-19.379	-7.320
C	22.180	-19.296	-6.078
C	21.363	-18.663	-4.953
O	21.930	-18.429	-3.886
C	23.463	-18.527	-6.288
H	21.400	-18.636	-8.031
H	22.446	-20.232	-5.788
H	24.173	-19.140	-6.639
H	23.312	-17.788	-6.947
H	23.770	-18.134	-5.419
N	20.080	-18.352	-5.188
C	19.192	-17.842	-4.156
C	19.783	-16.590	-3.493
O	19.830	-16.453	-2.261
C	18.886	-18.915	-3.105
C	18.259	-20.133	-3.713
H	19.781	-18.510	-6.177
H	18.317	-17.583	-4.580
H	19.736	-19.186	-2.658
H	18.255	-18.538	-2.429
C	18.674	-21.426	-3.715
N	17.123	-20.082	-4.496
C	16.842	-21.329	-4.920
N	17.772	-22.157	-4.463
H	16.599	-19.273	-4.715
H	19.484	-21.787	-3.258
H	16.062	-21.578	-5.482
N	20.229	-15.673	-4.328
C	20.927	-14.503	-3.833
C	20.751	-13.388	-4.866
O	20.322	-13.645	-6.002
C	22.377	-14.831	-3.546
O	23.010	-15.279	-4.730
H	20.044	-15.854	-5.329
H	20.492	-14.206	-2.990
H	22.839	-14.015	-3.222
H	22.420	-15.552	-2.866
H	22.640	-16.170	-4.990
N	21.039	-12.151	-4.444
C	20.962	-10.991	-5.327
C	22.060	-10.005	-4.912
O	22.354	-9.856	-3.729
C	19.585	-10.358	-5.277
H	21.319	-12.093	-3.444
H	21.135	-11.281	-6.263
H	18.899	-11.059	-5.079
H	19.561	-9.660	-4.560
H	19.377	-9.933	-6.159
N	22.669	-9.335	-5.888
C	23.660	-8.297	-5.649
C	23.140	-6.968	-6.192
O	22.523	-6.904	-7.262
C	24.988	-8.623	-6.350
C	26.046	-7.556	-6.157
O	25.461	-9.852	-5.814
H	22.369	-9.624	-6.853
H	23.795	-8.201	-4.662
H	24.812	-8.748	-7.340
H	26.945	-7.983	-6.026
H	26.084	-6.960	-6.964
H	25.829	-6.999	-5.350
H	25.210	-9.936	-4.842
N	23.342	-5.891	-5.427
C	23.111	-4.550	-5.935
C	24.447	-3.816	-5.920
O	25.223	-3.939	-4.977
C	22.082	-3.760	-5.116
C	20.728	-4.401	-5.122
O	22.638	-3.600	-3.802
H	23.669	-6.098	-4.466
H	22.797	-4.611	-6.877
H	21.967	-2.844	-5.525
H	20.810	-5.395	-5.258
H	20.257	-4.237	-4.248
H	20.163	-4.023	-5.864
H	23.083	-4.451	-3.506
N	24.719	-3.078	-7.005
C	25.859	-2.202	-7.130
C	25.362	-0.762	-7.156
O	24.432	-0.437	-7.898
C	26.615	-2.458	-8.453
C	27.324	-3.759	-8.626
C	28.669	-3.941	-8.531
C	26.784	-5.052	-8.953
C	27.882	-5.935	-9.080
C	25.501	-5.543	-9.196
N	29.005	-5.234	-8.812
C	27.748	-7.287	-9.415
C	25.372	-6.870	-9.581
C	26.470	-7.719	-9.672
H	24.004	-3.202	-7.776
H	26.427	-2.307	-6.315
H	25.952	-2.375	-9.214
H	27.303	-1.724	-8.567
H	29.327	-3.221	-8.287
H	29.941	-5.602	-8.817
H	24.699	-4.967	-9.098
H	28.540	-7.883	-9.460
H	24.457	-7.217	-9.798
H	26.319	-8.679	-9.934
N	26.001	0.063	-6.333
C	25.725	1.472	-6.234
C	27.028	2.203	-6.541
O	28.082	1.949	-5.938
C	25.176	1.755	-4.855
O	24.908	3.134	-4.692
H	26.741	-0.419	-5.750
H	25.067	1.721	-6.935
H	24.328	1.246	-4.728
H	25.846	1.475	-4.171
H	24.218	3.259	-3.979
N	26.966	3.169	-7.465
C	28.191	3.821	-7.822
C	28.029	5.053	-8.725
O	26.998	5.711	-8.717
H	26.048	3.366	-7.844
H	28.692	4.109	-6.975
H	28.814	3.158	-8.295
N	29.154	5.378	-9.369
C	29.165	6.507	-10.287
C	30.032	6.195	-11.500
O	31.086	5.578	-11.406
C	29.501	7.807	-9.551
C	30.851	7.916	-8.881
C	30.983	9.235	-8.148
H	29.966	4.786	-9.164

H	28.226	6.655	-10.611
H	29.417	8.578	-10.215
H	28.784	7.963	-8.840
H	30.948	7.177	-8.224
H	31.562	7.864	-9.573
N	31.529	9.174	-6.943
O	30.615	10.299	-8.661
H	31.666	10.023	-6.389
H	31.812	8.271	-6.571
N	29.503	6.687	-12.630
C	30.196	6.734	-13.902
C	31.020	8.014	-13.955
O	30.476	9.078	-13.710
C	29.151	6.709	-15.006
C	29.695	7.095	-16.357
C	30.380	6.186	-17.130
C	29.559	8.395	-16.810
C	30.908	6.544	-18.366
C	30.043	8.760	-18.057
C	30.720	7.833	-18.831
O	31.194	8.214	-20.088
H	28.516	7.043	-12.503
H	30.823	5.959	-13.955
H	28.782	5.785	-15.070
H	28.425	7.350	-14.768
H	30.507	5.244	-16.804
H	29.110	9.076	-16.239
H	31.414	5.877	-18.909
H	29.904	9.691	-18.397
H	30.731	7.670	-20.780
N	32.305	7.867	-14.293
C	33.238	8.957	-14.511
C	33.751	8.887	-15.954
O	34.421	7.915	-16.305
C	34.408	8.848	-13.522
C	35.349	10.041	-13.674
C	33.919	8.669	-12.081
H	32.591	6.849	-14.391
H	32.750	9.821	-14.404
H	34.932	8.036	-13.757
H	34.811	10.874	-13.799
H	35.911	10.126	-12.853
H	35.937	9.900	-14.469
H	32.977	8.991	-12.015
H	33.965	7.702	-11.840
H	34.504	9.200	-11.471
N	33.439	9.909	-16.762
C	33.756	9.923	-18.188
C	35.221	10.269	-18.476
O	36.038	10.340	-17.570
H	32.948	10.697	-16.279
H	33.548	9.013	-18.587
H	33.160	10.597	-18.658
N	35.546	10.458	-19.763
C	36.856	10.936	-20.174
C	37.710	9.821	-20.770
O	37.198	8.754	-21.115
H	34.779	10.232	-20.433
H	36.748	11.672	-20.860
H	37.337	11.330	-19.377
N	39.020	10.095	-20.848
C	39.983	9.257	-21.551
C	39.798	7.791	-21.170
O	39.489	6.959	-22.021
C	41.384	9.727	-21.221
H	39.293	10.981	-20.350
H	39.835	9.351	-22.542
H	41.987	8.937	-21.105
H	41.732	10.301	-21.964
H	41.371	10.258	-20.373
N	39.988	7.527	-19.873
C	39.884	6.213	-19.263
C	38.594	6.161	-18.424
O	38.645	6.296	-17.207
C	41.102	5.969	-18.359
C	42.457	6.117	-19.039
C	42.853	4.955	-19.932
O	41.957	4.232	-20.391
O	44.062	4.758	-20.135
H	40.230	8.395	-19.309
H	39.845	5.507	-19.966
H	41.063	6.629	-17.624
H	41.039	5.044	-18.017
H	42.430	6.931	-19.604
H	43.149	6.199	-18.334
N	37.437	5.980	-19.075
C	36.166	6.019	-18.365
C	36.107	4.860	-17.365
O	36.675	3.787	-17.574
C	35.003	5.996	-19.325
H	37.530	5.817	-20.089
H	36.100	6.879	-17.857
H	34.143	6.104	-18.822
H	35.091	6.744	-19.986
H	34.985	5.124	-19.819
N	35.382	5.084	-16.267
C	35.340	4.102	-15.197
C	33.955	4.164	-14.567
O	33.317	5.211	-14.566
C	36.452	4.362	-14.168
C	37.696	3.519	-14.372
C	38.887	3.944	-13.537
N	39.996	3.009	-13.657
C	40.712	2.802	-14.755
N	41.697	1.922	-14.731
N	40.444	3.459	-15.874
H	34.876	5.982	-16.251
H	35.477	3.194	-15.580
H	36.711	5.322	-14.231
H	36.086	4.165	-13.263
H	37.486	2.556	-14.148
H	37.969	3.558	-15.344
H	39.179	4.845	-13.849
H	38.596	3.991	-12.584
H	40.244	2.469	-12.832
H	41.911	1.390	-13.918
H	42.256	1.780	-15.565
H	39.674	4.107	-15.887
H	40.993	3.316	-16.690
N	33.497	3.010	-14.046
C	32.350	2.975	-13.142
C	32.862	2.390	-11.820
O	33.340	1.270	-11.773
C	31.160	2.177	-13.716
C	30.639	2.775	-15.032
C	30.050	2.059	-12.669
C	29.603	1.933	-15.748
H	34.024	2.168	-14.341
H	32.109	3.915	-12.957
H	31.477	1.247	-13.906
H	30.246	3.675	-14.833
H	31.423	2.908	-15.643
H	29.328	1.468	-13.020
H	30.427	1.668	-11.832
H	29.679	2.965	-12.479
H	28.810	1.809	-15.154
H	29.323	2.396	-16.587
H	29.998	1.043	-15.971
N	32.821	3.213	-10.771
C	33.350	2.857	-9.478
C	32.159	2.557	-8.568
O	31.249	3.376	-8.461
C	34.149	4.007	-8.868
C	35.391	4.364	-9.642
H	32.370	4.137	-10.977
H	33.855	2.006	-9.578
H	33.554	4.813	-8.824
H	34.414	3.744	-7.937
N	35.549	5.654	-9.892
O	36.140	3.479	-10.058
H	34.885	6.327	-9.576
H	36.355	5.967	-10.411
N	32.155	1.379	-7.960
C	30.953	0.907	-7.284
C	31.310	0.274	-5.939
O	32.436	-0.156	-5.712
C	30.148	-0.154	-8.057
C	29.839	0.223	-9.494
O	30.847	-1.396	-8.056
H	33.037	0.851	-8.005
H	30.351	1.693	-7.111
H	29.250	-0.290	-7.587
H	29.744	-0.605	-10.052
H	28.983	0.744	-9.534
H	30.581	0.785	-9.867
H	31.809	-1.260	-8.319
N	30.275	0.239	-5.095
C	30.260	-0.663	-3.963
C	29.056	-1.540	-4.148
O	28.089	-1.162	-4.827
C	30.197	0.172	-2.681
C	31.499	0.959	-2.508
C	31.701	1.636	-1.180
H	29.509	0.899	-5.323
H	31.071	-1.232	-4.009
H	29.457	0.815	-2.751
H	30.097	-0.429	-1.909
H	32.273	0.320	-2.669
H	31.538	1.661	-3.242
N	31.528	2.934	-1.139
O	32.008	0.995	-0.183
H	31.633	3.464	-0.271
H	31.281	3.433	-2.000
N	29.113	-2.729	-3.566
C	28.038	-3.648	-3.782
C	27.623	-4.351	-2.480
O	28.439	-4.448	-1.562
C	28.361	-4.650	-4.887
C	29.605	-5.439	-4.777
C	30.719	-5.263	-5.535
C	29.835	-6.593	-3.958
C	31.138	-7.032	-4.250
C	29.073	-7.310	-3.029
N	31.671	-6.141	-5.147
C	31.740	-8.101	-3.588
C	29.666	-8.365	-2.365
C	30.966	-8.772	-2.655
H	29.941	-2.906	-2.992
H	27.231	-3.131	-4.104
H	27.591	-5.309	-4.953
H	28.399	-4.148	-5.769
H	30.814	-4.587	-6.266
H	32.629	-6.152	-5.460
H	28.124	-7.067	-2.848
H	32.681	-8.363	-3.783
H	29.144	-8.846	-1.659
H	31.349	-9.563	-2.181
N	26.348	-4.780	-2.475
C	25.810	-5.608	-1.409
C	25.246	-6.886	-2.018
O	24.378	-6.817	-2.899
C	24.692	-4.845	-0.693
C	25.070	-3.580	0.092
C	23.819	-2.834	0.592
C	25.913	-3.901	1.290
H	25.792	-4.465	-3.298
H	26.550	-5.855	-0.786
H	24.017	-4.571	-1.384
H	24.258	-5.475	-0.045
H	25.595	-2.971	-0.503
H	23.297	-3.438	1.190
H	24.104	-2.021	1.095
H	23.263	-2.570	-0.192
H	26.763	-4.354	1.004
H	26.146	-3.058	1.785
H	25.413	-4.513	1.910
N	25.625	-8.030	-1.455
C	25.185	-9.331	-1.956
C	24.430	-10.007	-0.820
O	25.045	-10.262	0.218
C	26.403	-10.156	-2.372
C	26.139	-11.517	-3.039
C	27.459	-12.158	-3.480
C	25.385	-12.430	-2.119
H	26.257	-7.920	-0.633

H	24.561	-9.188	-2.711
H	26.937	-9.612	-3.020
H	26.950	-10.332	-1.554
H	25.557	-11.379	-3.836
H	28.043	-12.292	-2.681
H	27.270	-13.039	-3.910
H	27.915	-11.556	-4.133
H	24.501	-12.021	-1.874
H	25.220	-13.314	-2.567
H	25.913	-12.587	-1.279
N	23.110	-10.112	-0.986
C	22.283	-10.796	0.006
C	21.951	-12.220	-0.447
O	21.469	-12.452	-1.570
C	20.971	-10.048	0.275
C	20.141	-10.673	1.389
O	21.310	-8.714	0.659
H	22.740	-9.682	-1.847
H	22.798	-10.853	0.868
H	20.434	-10.007	-0.583
H	20.728	-11.239	1.973
H	19.721	-9.952	1.945
H	19.420	-11.246	0.994
H	21.377	-8.121	-0.151
N	22.068	-13.148	0.501
C	21.696	-14.539	0.282
C	20.450	-14.825	1.111
O	20.333	-14.336	2.237
C	22.844	-15.513	0.623
C	24.128	-15.225	-0.159
C	22.394	-16.952	0.409
C	25.231	-14.641	0.693
H	22.447	-12.793	1.407
H	21.426	-14.612	-0.672
H	23.049	-15.414	1.601
H	24.447	-16.077	-0.566
H	23.910	-14.581	-0.889
H	23.109	-17.575	0.722
H	21.557	-17.120	0.926
H	22.221	-17.106	-0.562
H	25.461	-15.283	1.424
H	26.037	-14.477	0.126
H	24.922	-13.778	1.091
N	19.472	-15.521	0.509
C	18.291	-15.959	1.234
C	18.692	-16.829	2.431
O	19.567	-17.672	2.293
H	19.638	-15.710	-0.497
H	17.780	-15.162	1.566
H	17.697	-16.493	0.626
N	18.194	-16.491	3.627
C	18.563	-17.221	4.840
C	17.303	-17.412	5.684
O	16.299	-16.739	5.492
C	19.641	-16.525	5.710
C	20.939	-16.226	4.988
O	19.106	-15.324	6.270
H	17.542	-15.688	3.615
H	18.907	-18.118	4.580
H	19.894	-17.152	6.474
H	20.832	-16.396	4.005
H	21.196	-15.266	5.127
H	21.672	-16.813	5.342
H	18.597	-15.526	7.114
N	17.380	-18.360	6.631
C	16.488	-18.401	7.781
C	16.727	-17.176	8.664
O	17.765	-16.497	8.581
C	16.728	-19.684	8.598
C	16.668	-20.937	7.747
O	18.031	-19.555	9.160
H	18.137	-19.062	6.466
H	15.541	-18.373	7.456
H	16.056	-19.722	9.348
H	17.058	-21.713	8.251
H	15.715	-21.147	7.512
H	17.190	-20.802	6.901
H	18.444	-18.675	8.897
N	15.772	-16.927	9.565
C	15.818	-15.751	10.425
C	17.016	-15.892	11.357
O	17.715	-14.903	11.601
C	14.431	-15.531	11.067
C	13.364	-15.320	9.978
C	11.920	-15.019	10.349
O	11.063	-15.886	10.092
O	11.633	-13.885	10.795
H	15.011	-17.632	9.595
H	15.919	-14.929	9.872
H	14.199	-16.333	11.553
H	14.473	-14.724	11.596
H	13.685	-14.557	9.389
H	13.354	-16.156	9.401
N	17.326	-17.142	11.756
C	18.469	-17.440	12.612
C	19.815	-17.064	11.968
O	20.763	-16.707	12.678
C	18.460	-18.913	12.941
H	16.673	-17.873	11.395
H	18.377	-16.917	13.476
H	19.298	-19.343	12.600
H	18.407	-19.040	13.933
H	17.669	-19.353	12.512
N	19.924	-17.211	10.641
C	21.184	-16.973	9.939
C	21.244	-15.589	9.271
O	22.177	-15.340	8.528
C	21.466	-18.069	8.914
C	21.752	-19.396	9.586
H	19.049	-17.504	10.166
H	21.921	-16.993	10.632
H	20.668	-18.171	8.334
H	22.259	-17.805	8.380
N	21.361	-20.493	8.963
O	22.283	-19.419	10.689
H	20.898	-20.413	8.072
H	21.519	-21.391	9.363
N	20.270	-14.711	9.516
C	20.126	-13.467	8.757
C	21.285	-12.503	9.038
O	21.590	-11.642	8.211
C	18.790	-12.819	9.055
H	19.626	-14.985	10.288
H	20.125	-13.686	7.773
H	18.717	-12.630	10.035
H	18.710	-11.960	8.547
H	18.048	-13.433	8.782
N	21.949	-12.643	10.194
C	23.138	-11.850	10.512
C	24.278	-12.128	9.534
O	25.176	-11.300	9.361
C	23.585	-12.061	11.986
C	24.138	-13.427	12.249
C	23.439	-14.559	12.577
C	25.499	-13.844	12.067
C	25.554	-15.226	12.384
C	26.690	-13.173	11.786
N	24.288	-15.630	12.682
C	26.740	-15.947	12.364
C	27.863	-13.889	11.736
C	27.889	-15.255	12.043
H	21.549	-13.356	10.836
H	22.890	-10.877	10.420
H	24.294	-11.403	12.193
H	22.797	-11.936	12.571
H	22.450	-14.597	12.719
H	24.019	-16.561	12.937
H	26.693	-12.185	11.622
H	26.752	-16.925	12.575
H	28.708	-13.428	11.478
H	28.766	-15.737	12.027
N	24.317	-13.304	8.921
C	25.395	-13.610	7.993
C	24.852	-13.688	6.559
O	25.433	-14.308	5.684
C	26.159	-14.847	8.457
C	25.368	-16.147	8.484
C	26.230	-17.352	8.858
C	25.395	-18.533	9.311
N	26.201	-19.634	9.899
H	23.550	-13.951	9.151
H	26.082	-12.880	8.032
H	26.948	-14.975	7.846
H	26.503	-14.671	9.387
H	24.625	-16.065	9.155
H	24.969	-16.308	7.577
H	26.762	-17.622	8.059
H	26.842	-17.088	9.600
H	24.724	-18.214	9.997
H	24.878	-18.891	8.519
H	25.865	-20.512	9.557
H	27.160	-19.519	9.640
H	26.119	-19.611	10.895
N	23.824	-12.901	6.277
C	23.162	-12.926	4.977
C	23.793	-11.964	3.963
O	23.506	-12.132	2.788
C	21.689	-12.615	5.118
O	21.456	-11.271	5.568
H	23.532	-12.270	7.053
H	23.244	-13.864	4.600
H	21.237	-12.729	4.233
H	21.278	-13.240	5.783
H	21.502	-11.250	6.563
N	24.596	-10.975	4.373
C	24.928	-9.898	3.434
C	26.431	-9.677	3.349
O	27.068	-9.318	4.337
C	24.212	-8.575	3.732
C	24.382	-7.539	2.617
O	22.813	-8.814	3.858
H	24.931	-11.035	5.330
H	24.591	-10.161	2.520
H	24.556	-8.207	4.620
H	23.566	-7.533	2.038
H	24.510	-6.632	3.019
H	25.182	-7.772	2.062
H	22.643	-9.637	4.412
N	26.988	-9.888	2.149
C	28.365	-9.507	1.894
C	28.412	-8.100	1.311
O	27.504	-7.712	0.566
C	28.973	-10.498	0.892
C	29.199	-11.932	1.357
C	29.476	-12.838	0.156
C	30.363	-11.995	2.318
H	26.367	-10.330	1.454
H	28.868	-9.506	2.762
H	28.363	-10.534	0.095
H	29.861	-10.131	0.606
H	28.383	-12.257	1.831
H	30.292	-12.515	-0.321
H	29.622	-13.774	0.475
H	28.693	-12.813	-0.464
H	30.175	-11.421	3.118
H	30.505	-12.941	2.619
H	31.195	-11.666	1.866
N	29.566	-7.451	1.499
C	29.829	-6.128	0.968
C	31.156	-6.165	0.254
O	32.135	-6.799	0.675
C	29.801	-5.036	2.043
C	30.831	-5.248	3.159
C	29.964	-3.668	1.426
H	30.264	-7.990	2.068
H	29.119	-5.911	0.301
H	28.896	-5.057	2.483
H	30.354	-5.448	4.013
H	31.424	-6.014	2.917
H	31.377	-4.419	3.264
H	29.697	-2.966	2.089
H	30.921	-3.530	1.163
H	29.383	-3.594	0.613
N	31.209	-5.426	-0.847
C	32.466	-5.297	-1.554

C	32.488	-4.091	-2.477
O	31.651	-3.194	-2.371
H	30.330	-4.983	-1.131
H	33.224	-5.217	-0.885
H	32.636	-6.135	-2.099
N	33.531	-4.028	-3.320
C	33.654	-2.911	-4.238
C	34.200	-3.424	-5.569
O	35.044	-4.304	-5.571
C	34.551	-1.814	-3.666
C	35.793	-2.326	-3.044
C	37.081	-2.377	-3.458
N	35.788	-2.793	-1.750
C	37.037	-3.145	-1.404
N	37.840	-2.910	-2.435
H	34.195	-4.807	-3.252
H	32.747	-2.525	-4.401
H	34.799	-1.179	-4.408
H	34.031	-1.300	-2.973
H	34.992	-2.863	-1.157
H	37.428	-2.082	-4.349
H	37.307	-3.515	-0.525
N	33.680	-2.909	-6.673
C	34.124	-3.299	-8.004
C	34.430	-2.019	-8.792
O	33.648	-1.078	-8.727
C	33.049	-4.106	-8.733
C	32.814	-5.556	-8.287
O	33.609	-6.100	-7.493
O	31.770	-6.119	-8.682
H	32.927	-2.202	-6.508
H	34.985	-3.788	-7.903
H	32.171	-3.607	-8.641
H	33.285	-4.119	-9.719
N	35.524	-2.013	-9.566
C	35.763	-0.927	-10.500
C	35.706	-1.488	-11.929
O	36.363	-2.488	-12.181
C	37.134	-0.270	-10.274
C	37.313	0.935	-11.165
O	37.238	0.176	-8.925
H	36.152	-2.820	-9.438
H	35.037	-0.250	-10.405
H	37.863	-0.967	-10.439
H	36.477	1.099	-11.698
H	37.508	1.750	-10.610
H	38.077	0.786	-11.800
H	37.066	-0.584	-8.287
N	34.918	-0.840	-12.794
C	34.666	-1.310	-14.154
C	35.372	-0.392	-15.147
O	35.298	0.805	-15.001
C	33.176	-1.339	-14.449
C	32.376	-2.317	-13.623
C	31.826	-1.949	-12.393
C	32.197	-3.626	-14.057
C	31.165	-2.908	-11.636
C	31.518	-4.573	-13.290
C	30.970	-4.184	-12.094
H	34.496	0.038	-12.408
H	35.094	-2.210	-14.237
H	32.809	-0.430	-14.277
H	33.055	-1.585	-15.405
H	31.904	-1.025	-12.067
H	32.568	-3.901	-14.953
H	30.820	-2.639	-10.710
H	31.442	-5.520	-13.621
H	30.425	-4.833	-11.553
N	36.065	-0.975	-16.128
C	36.676	-0.196	-17.193
C	36.175	-0.729	-18.536
O	35.573	-1.790	-18.577
C	38.212	-0.227	-17.091
C	38.731	0.335	-15.788
O	38.667	-1.572	-17.145
H	36.125	-2.008	-16.061
H	36.385	0.755	-17.106
H	38.602	0.279	-17.888
H	39.545	0.900	-15.955
H	38.027	0.903	-15.352
H	38.978	-0.412	-15.164
H	37.951	-2.203	-16.821
N	36.435	0.030	-19.619
C	36.005	-0.344	-20.958
C	36.966	-1.361	-21.558
O	36.663	-1.933	-22.589
C	35.985	0.857	-21.905
C	35.003	1.972	-21.582
C	33.568	1.537	-21.600
C	33.054	1.160	-22.972
N	31.637	0.723	-22.898
H	36.968	0.900	-19.406
H	35.090	-0.757	-20.905
H	36.914	1.254	-21.923
H	35.778	0.516	-22.834
H	35.227	2.341	-20.666
H	35.133	2.719	-22.252
H	33.458	0.739	-20.992
H	32.992	2.284	-21.242
H	33.123	1.953	-23.577
H	33.610	0.413	-23.337
H	31.423	0.150	-23.689
H	31.493	0.199	-22.058
H	31.040	1.526	-22.897
N	38.110	-1.591	-20.918
C	39.095	-2.549	-21.405
C	39.334	-3.613	-20.335
O	39.170	-3.311	-19.156
C	40.412	-1.836	-21.782
C	40.161	-0.570	-22.604
C	41.271	-1.517	-20.564
H	38.238	-1.032	-20.048
H	38.720	-3.004	-22.213
H	40.938	-2.457	-22.361
H	40.830	0.126	-22.350
H	40.251	-0.784	-23.575
H	39.240	-0.233	-22.418
H	41.812	-0.697	-20.749
H	40.678	-1.360	-19.775
H	41.880	-2.288	-20.380
N	39.694	-4.841	-20.752
C	40.275	-5.870	-19.885
C	39.356	-7.094	-19.819
O	39.153	-7.712	-20.877
C	40.546	-5.371	-18.458
C	40.911	-6.444	-17.439
C	42.003	-7.373	-17.918
C	42.661	-8.144	-16.794
N	43.896	-8.822	-17.256
O	38.762	-7.601	-18.900
H	39.517	-5.000	-21.778
H	41.160	-6.153	-20.265
H	41.300	-4.709	-18.500
H	39.720	-4.898	-18.134
H	41.214	-5.992	-16.590
H	40.087	-6.986	-17.231
H	41.609	-8.030	-18.566
H	42.706	-6.833	-18.387
H	42.890	-7.509	-16.053
H	42.017	-8.831	-16.448
H	43.923	-9.753	-16.890
H	43.904	-8.857	-18.256
H	44.696	-8.315	-16.934
N	29.761	11.650	28.317
C	28.947	10.417	28.505
C	29.881	9.226	28.675
O	29.662	8.164	28.096
C	28.020	10.122	27.316
C	27.894	11.232	26.284
O	27.255	12.267	26.589
O	28.449	11.054	25.187
H	30.754	11.681	28.306
H	28.449	10.534	29.360
H	28.367	9.297	26.857
H	27.106	9.923	27.684
N	30.950	9.420	29.447
C	31.796	8.323	29.899
C	30.989	7.299	30.710
O	31.133	6.083	30.540
C	32.894	8.918	30.778
C	33.880	7.907	31.294
C	35.048	8.577	31.992
O	35.944	7.851	32.477
O	35.060	9.825	32.041
H	31.125	10.418	29.696
H	32.211	7.862	29.106
H	33.387	9.574	30.232
H	32.457	9.342	31.553
H	33.432	7.337	31.953
H	34.242	7.402	30.537
N	30.203	7.832	31.656
C	29.293	7.069	32.499
C	28.133	6.511	31.673
O	27.693	5.385	31.931
C	28.777	7.956	33.610
H	30.306	8.881	31.737
H	29.794	6.297	32.906
H	28.067	8.566	33.255
H	28.390	7.393	34.341
H	29.527	8.506	33.981
N	27.636	7.320	30.713
C	26.574	6.912	29.802
C	26.962	5.658	29.024
O	26.165	4.728	28.913
H	28.085	8.263	30.687
H	25.732	6.729	30.326
H	26.382	7.659	29.152
N	28.206	5.624	28.505
C	28.658	4.478	27.724
C	29.166	3.337	28.614
O	28.958	2.189	28.307
C	29.750	4.893	26.715
C	29.181	5.821	25.646
C	30.384	3.643	26.135
C	30.243	6.498	24.787
H	28.781	6.454	28.707
H	27.846	4.123	27.243
H	30.454	5.389	27.216
H	28.579	5.293	25.051
H	28.642	6.531	26.097
H	31.134	3.898	25.525
H	30.739	3.072	26.874
H	29.700	3.128	25.619
H	30.779	5.798	24.321
H	29.794	7.084	24.117
H	30.836	7.045	25.373
N	29.842	3.638	29.725
C	30.455	2.603	30.532
C	29.334	1.708	31.069
O	28.286	2.218	31.481
C	31.319	3.174	31.674
C	31.703	2.107	32.672
O	32.542	3.752	31.199
H	29.884	4.649	29.946
H	31.046	2.047	29.957
H	30.784	3.914	32.153
H	32.210	2.516	33.437
H	30.880	1.658	33.032
H	32.283	1.415	32.232
H	33.196	3.856	31.955
N	29.541	0.393	30.969
C	28.609	-0.574	31.520
C	28.364	-1.745	30.593
O	29.216	-2.101	29.779
H	30.415	0.132	30.469
H	28.967	-0.918	32.407
H	27.725	-0.113	31.718
N	27.185	-2.353	30.760
C	26.883	-3.615	30.111
C	25.739	-3.374	29.125
O	24.732	-2.738	29.462
C	26.618	-4.744	31.128
C	26.113	-5.993	30.447
O	27.806	-5.140	31.832
H	26.524	-1.849	31.382

H	27.680	-3.914	29.595
H	25.936	-4.407	31.824
H	26.885	-6.580	30.183
H	25.513	-6.507	31.068
H	25.594	-5.751	29.622
H	28.580	-5.214	31.195
N	25.935	-3.876	27.890
C	25.032	-3.639	26.781
C	24.658	-4.960	26.147
O	25.473	-5.880	26.095
C	25.698	-2.730	25.736
C	25.946	-1.337	26.190
C	27.056	-0.887	26.829
C	25.054	-0.219	26.077
C	25.697	0.872	26.689
C	23.771	-0.039	25.548
N	26.921	0.440	27.104
C	25.173	2.154	26.673
C	23.231	1.222	25.576
C	23.910	2.294	26.157
H	26.806	-4.456	27.811
H	24.195	-3.228	27.148
H	26.579	-3.128	25.486
H	25.107	-2.686	24.933
H	27.852	-1.445	27.063
H	27.617	1.018	27.547
H	23.266	-0.804	25.162
H	25.697	2.930	27.023
H	22.328	1.376	25.170
H	23.456	3.189	26.195
N	23.444	-5.011	25.582
C	22.946	-6.216	24.945
C	22.401	-5.884	23.561
O	21.706	-4.886	23.424
C	21.836	-6.816	25.836
C	22.106	-6.843	27.336
C	22.819	-7.887	27.909
C	21.604	-5.865	28.192
C	23.060	-7.955	29.275
C	21.848	-5.901	29.567
C	22.566	-6.961	30.116
O	22.846	-7.057	31.472
H	22.906	-4.124	25.643
H	23.710	-6.858	24.833
H	20.996	-6.285	25.682
H	21.676	-7.762	25.534
H	23.175	-8.623	27.317
H	21.056	-5.117	27.816
H	23.588	-8.715	29.657
H	21.508	-5.169	30.155
H	23.335	-6.237	31.758
N	22.634	-6.750	22.564
C	22.114	-6.502	21.222
C	20.868	-7.349	20.972
O	20.398	-8.070	21.860
C	23.184	-6.578	20.113
C	23.705	-7.946	19.701
H	23.193	-7.575	22.825
H	21.827	-5.541	21.187
H	22.802	-6.121	19.285
H	23.978	-6.013	20.413
N	24.792	-7.954	18.938
O	23.167	-8.987	20.060
H	25.224	-7.092	18.672
H	25.181	-8.822	18.629
N	20.311	-7.213	19.760
C	19.074	-7.895	19.395
C	19.277	-9.419	19.372
O	18.309	-10.154	19.498
C	18.517	-7.288	18.093
C	19.333	-7.529	16.806
C	20.651	-6.788	16.632
H	20.834	-6.587	19.116
H	18.385	-7.693	20.098
H	17.589	-7.658	17.937
H	18.432	-6.287	18.215
H	19.520	-8.532	16.741
H	18.731	-7.303	16.011
N	21.381	-7.167	15.591
O	21.035	-5.898	17.400
H	22.276	-6.708	15.395
H	21.045	-7.914	14.993
N	20.522	-9.905	19.202
C	20.802	-11.323	19.029
C	21.146	-11.989	20.366
O	21.555	-13.147	20.368
C	22.002	-11.508	18.092
C	21.922	-10.914	16.681
C	23.239	-11.169	15.931
C	20.713	-11.454	15.913
H	21.264	-9.168	19.206
H	19.981	-11.773	18.657
H	22.804	-11.098	18.540
H	22.159	-12.495	17.990
H	21.769	-9.933	16.747
H	23.395	-12.153	15.869
H	23.175	-10.778	15.015
H	23.988	-10.739	16.430
H	19.876	-11.217	16.405
H	20.693	-11.047	15.001
H	20.788	-12.447	15.836
N	21.124	-11.223	21.460
C	21.522	-11.717	22.763
C	23.036	-11.782	22.981
O	23.524	-12.563	23.793
H	20.797	-10.246	21.289
H	21.113	-11.126	23.490
H	21.131	-12.651	22.906
N	23.810	-10.999	22.231
C	25.219	-10.833	22.537
C	25.362	-9.795	23.655
O	24.515	-8.893	23.790
C	26.016	-10.428	21.280
O	25.990	-11.435	20.263
H	23.330	-10.538	21.440
H	25.574	-11.700	22.882
H	25.622	-9.588	20.909
H	26.966	-10.265	21.544
H	26.702	-11.240	19.593
N	26.462	-9.928	24.420
C	26.810	-9.068	25.546
C	28.102	-8.272	25.307
O	29.165	-8.868	25.113
C	27.077	-9.917	26.802
C	27.212	-9.077	28.047
O	26.009	-10.858	26.902
H	27.073	-10.742	24.120
H	26.058	-8.405	25.690
H	27.942	-10.424	26.675
H	27.884	-8.344	27.900
H	26.328	-8.662	28.283
H	27.517	-9.645	28.817
H	26.033	-11.502	26.129
N	28.006	-6.950	25.490
C	29.098	-6.002	25.298
C	29.359	-5.298	26.628
O	28.536	-4.505	27.063
C	28.636	-5.076	24.165
C	29.407	-3.819	23.886
C	30.777	-3.857	23.657
C	28.724	-2.628	23.694
C	31.472	-2.698	23.334
C	29.412	-1.472	23.392
C	30.787	-1.505	23.211
H	27.039	-6.643	25.795
H	29.915	-6.533	25.089
H	28.628	-5.625	23.315
H	27.681	-4.811	24.367
H	31.268	-4.719	23.723
H	27.728	-2.611	23.775
H	32.466	-2.738	23.194
H	28.909	-0.594	23.304
H	31.284	-0.664	22.990
N	30.522	-5.549	27.233
C	30.908	-4.931	28.491
C	32.058	-3.971	28.216
O	33.070	-4.392	27.663
C	31.339	-6.021	29.487
C	30.282	-7.131	29.614
C	31.690	-5.403	30.839
C	30.867	-8.461	30.093
H	31.130	-6.230	26.720
H	30.136	-4.374	28.782
H	32.180	-6.439	29.145
H	29.589	-6.836	30.260
H	29.868	-7.272	28.722
H	32.052	-6.110	31.442
H	32.376	-4.691	30.709
H	30.869	-5.006	31.245
H	31.282	-8.327	30.989
H	30.134	-9.133	30.153
H	31.555	-8.765	29.440
N	31.872	-2.702	28.548
C	32.788	-1.675	28.085
C	33.071	-0.693	29.212
O	32.170	-0.291	29.927
C	32.231	-0.993	26.802
C	31.017	-0.119	27.029
C	33.280	-0.194	26.056
H	31.048	-2.516	29.143
H	33.654	-2.114	27.832
H	31.954	-1.733	26.171
H	31.302	0.766	27.402
H	30.539	0.028	26.161
H	30.395	-0.564	27.676
H	33.818	-0.808	25.478
H	32.831	0.494	25.485
H	33.883	0.260	26.713
N	34.328	-0.255	29.288
C	34.679	0.954	30.000
C	35.103	2.041	29.009
O	35.907	1.788	28.115
C	35.795	0.703	31.023
C	36.123	1.950	31.820
O	35.397	-0.357	31.895
H	35.022	-0.857	28.794
H	33.873	1.286	30.499
H	36.636	0.410	30.528
H	36.644	2.593	31.252
H	35.277	2.395	32.124
H	36.671	1.708	32.625
H	34.959	-1.097	31.372
N	34.532	3.232	29.176
C	34.935	4.418	28.438
C	35.887	5.254	29.285
O	35.450	5.871	30.256
C	33.729	5.228	28.121
H	33.763	3.239	29.890
H	35.414	4.136	27.603
H	33.201	4.798	27.381
H	33.137	5.309	28.930
H	33.994	6.153	27.827
N	37.155	5.333	28.874
C	38.117	6.191	29.545
C	37.929	7.674	29.226
O	37.325	8.075	28.215
H	37.382	4.741	28.054
H	38.044	6.059	30.550
H	39.059	5.915	29.280
N	38.496	8.518	30.104
C	38.411	9.967	29.953
C	39.093	10.425	28.659
O	38.750	11.465	28.102
C	39.043	10.648	31.155
H	38.993	8.049	30.888
H	37.441	10.235	29.912
H	40.012	10.405	31.207
H	38.954	11.640	31.064
H	38.582	10.350	31.991
N	40.083	9.636	28.231
C	40.944	9.920	27.100
C	40.319	9.484	25.771
O	40.960	9.624	24.738
C	42.256	9.141	27.258
C	42.120	7.612	27.327

O	41.050	7.103	27.775
O	43.091	6.921	26.914
H	40.185	8.753	28.814
H	41.060	10.924	27.050
H	42.846	9.366	26.477
H	42.702	9.453	28.101
N	39.109	8.915	25.790
C	38.454	8.471	24.561
C	38.651	6.982	24.282
O	38.212	6.482	23.249
H	38.688	8.820	26.732
H	37.460	8.668	24.622
H	38.815	9.005	23.778
N	39.268	6.245	25.209
C	39.451	4.816	25.028
C	38.146	4.082	25.326
O	37.345	4.516	26.146
C	40.554	4.291	25.891
H	39.591	6.771	26.039
H	39.707	4.636	24.074
H	41.114	3.634	25.378
H	41.143	5.044	26.199
H	40.174	3.829	26.698
N	37.942	3.015	24.573
C	37.011	1.950	24.940
C	37.808	0.686	25.136
O	38.638	0.300	24.301
C	35.970	1.681	23.827
C	35.060	2.819	23.402
C	34.170	2.359	22.271
C	34.221	3.397	24.545
H	38.504	2.994	23.689
H	36.574	2.195	25.801
H	36.480	1.377	23.015
H	35.389	0.926	24.144
H	35.618	3.588	23.097
H	33.611	1.584	22.572
H	33.567	3.108	21.987
H	34.731	2.075	21.491
H	34.829	3.750	25.255
H	33.650	4.137	24.193
H	33.642	2.677	24.926
N	37.482	-0.028	26.224
C	38.140	-1.273	26.562
C	37.067	-2.227	27.098
O	36.089	-1.777	27.673
C	39.267	-1.006	27.583
C	40.378	-0.131	27.043
O	38.657	-0.384	28.712
H	36.718	0.396	26.795
H	38.525	-1.673	25.738
H	39.657	-1.891	27.873
H	41.271	-0.538	27.256
H	40.291	-0.040	26.047
H	40.329	0.782	27.458
H	37.872	0.177	28.425
N	37.201	-3.531	26.864
C	36.249	-4.453	27.457
C	36.265	-5.826	26.821
O	37.313	-6.346	26.395
H	37.984	-3.801	26.268
H	36.445	-4.543	28.453
H	35.312	-4.060	27.383
N	35.062	-6.422	26.854
C	34.835	-7.767	26.402
C	33.502	-7.837	25.673
O	32.521	-7.181	26.029
C	34.827	-8.792	27.562
C	36.081	-8.728	28.415
O	33.667	-8.479	28.330
H	34.305	-5.804	27.244
H	35.558	-8.024	25.755
H	34.721	-9.712	27.176
H	36.276	-7.776	28.665
H	35.953	-9.266	29.252
H	36.861	-9.098	27.904
H	33.432	-7.505	28.234
N	33.503	-8.724	24.691
C	32.325	-9.048	23.920
C	32.075	-10.559	24.015
O	32.967	-11.356	23.766
C	32.523	-8.636	22.456
C	31.255	-8.484	21.655
C	30.687	-9.547	20.976
C	30.647	-7.258	21.563
C	29.501	-9.400	20.277
C	29.470	-7.088	20.871
C	28.913	-8.156	20.198
O	27.727	-7.896	19.575
H	34.439	-9.173	24.522
H	31.536	-8.583	24.320
H	33.010	-7.760	22.444
H	33.092	-9.332	22.013
H	31.140	-10.443	20.987
H	31.069	-6.462	22.012
H	29.075	-10.191	19.835
H	29.017	-6.194	20.853
H	27.105	-8.668	19.708
N	30.810	-10.916	24.230
C	30.343	-12.287	24.074
C	29.263	-12.388	22.993
O	28.173	-11.861	23.217
C	29.766	-12.723	25.416
C	28.991	-14.029	25.344
C	29.252	-14.876	26.575
O	29.431	-16.101	26.408
O	29.372	-14.286	27.685
H	30.185	-10.129	24.519
H	31.116	-12.885	23.827
H	30.515	-12.856	26.037
H	29.145	-12.026	25.719
H	28.039	-13.821	25.323
H	29.301	-14.530	24.568
N	29.565	-13.120	21.897
C	28.706	-13.285	20.719
C	27.760	-14.473	20.917
O	28.210	-15.572	21.242
C	29.511	-13.480	19.441
O	28.665	-13.767	18.313
H	30.517	-13.583	21.968
H	28.147	-12.459	20.624
H	30.023	-12.648	19.244
H	30.140	-14.245	19.562
H	28.789	-14.720	18.051
N	26.461	-14.259	20.663
C	25.473	-15.329	20.750
C	25.394	-16.139	19.447
O	24.701	-17.152	19.414
C	24.139	-14.759	21.126
H	26.229	-13.281	20.404
H	25.735	-15.956	21.496
H	24.097	-14.600	22.116
H	23.990	-13.887	20.653
H	23.406	-15.395	20.870
N	26.123	-15.757	18.387
C	26.120	-16.545	17.155
C	27.537	-16.684	16.599
O	28.484	-16.026	17.054
C	25.147	-15.970	16.098
C	23.698	-16.340	16.393
C	25.296	-14.459	15.922
H	26.663	-14.891	18.518
H	25.782	-17.466	17.373
H	25.381	-16.383	15.218
H	23.092	-15.647	16.003
H	23.492	-17.230	15.987
H	23.561	-16.385	17.382
H	25.786	-14.276	15.071
H	24.389	-14.043	15.887
H	25.809	-14.089	16.695
N	27.657	-17.603	15.628
C	28.857	-17.768	14.821
C	29.940	-18.611	15.496
O	29.696	-19.285	16.502
H	26.799	-18.196	15.504
H	28.610	-18.200	13.933
H	29.243	-16.852	14.600
N	31.152	-18.571	14.914
C	32.299	-19.321	15.422
C	32.926	-18.551	16.599
O	34.026	-18.001	16.500
C	33.253	-19.665	14.268
C	34.385	-20.610	14.641
H	31.197	-17.958	14.071
H	31.954	-20.181	15.793
H	32.716	-20.088	13.534
H	33.653	-18.809	13.928
N	35.148	-21.023	13.645
O	34.589	-20.952	15.808
H	34.974	-20.712	12.709
H	35.910	-21.653	13.821
N	32.188	-18.492	17.717
C	32.572	-17.717	18.890
C	32.160	-18.477	20.152
O	31.082	-19.068	20.178
C	31.914	-16.354	18.860
H	31.306	-19.057	17.669
H	33.566	-17.605	18.897
H	31.168	-16.324	19.527
H	32.587	-15.648	19.087
H	31.546	-16.176	17.946
N	32.995	-18.445	21.197
C	32.566	-18.954	22.497
C	33.336	-18.287	23.636
O	34.570	-18.259	23.667
C	32.647	-20.489	22.600
C	34.033	-21.061	22.882
C	34.085	-22.577	23.020
O	33.861	-23.070	24.142
O	34.324	-23.265	21.997
H	33.923	-18.047	21.008
H	31.579	-18.786	22.618
H	32.053	-20.768	23.341
H	32.340	-20.861	21.736
H	34.627	-20.812	22.130
H	34.359	-20.676	23.734
N	32.542	-17.841	24.610
C	33.012	-17.082	25.746
C	33.338	-15.665	25.260
O	32.797	-15.177	24.256
C	34.173	-17.797	26.464
O	34.253	-17.414	27.843
H	31.527	-18.097	24.472
H	32.275	-17.009	26.411
H	34.025	-18.779	26.413
H	35.026	-17.552	26.015
H	33.655	-16.632	27.998
N	34.177	-14.988	26.031
C	34.359	-13.561	25.895
C	35.589	-13.334	25.028
O	36.525	-14.153	24.994
C	34.479	-12.892	27.270
C	33.480	-13.379	28.323
C	33.333	-12.506	29.603
N	32.233	-11.976	28.786
C	30.951	-11.970	29.164
N	30.015	-11.576	28.327
N	30.603	-12.384	30.372
H	34.685	-15.570	26.733
H	33.568	-13.168	25.434
H	35.399	-13.062	27.621
H	34.343	-11.909	27.152
H	32.558	-13.449	27.905
H	33.742	-14.310	28.629
H	32.998	-13.039	30.231
H	34.010	-11.929	29.589
H	32.460	-11.594	27.885
H	30.233	-11.295	27.394
H	29.050	-11.556	28.627
H	31.314	-12.723	30.998
H	29.651	-12.363	30.661
N	35.556	-12.190	24.331
C	36.681	-11.746	23.529
C	37.025	-10.311	23.919
O	36.147	-9.532	24.295

C	36.350	-11.880	22.039
C	36.068	-13.292	21.579
C	34.846	-13.908	21.815
C	37.029	-14.008	20.895
C	34.591	-15.197	21.375
C	36.806	-15.319	20.502
C	35.577	-15.911	20.732
O	35.404	-17.213	20.267
H	34.664	-11.662	24.419
H	37.471	-12.321	23.738
H	35.543	-11.321	21.848
H	37.127	-11.531	21.515
H	34.131	-13.411	22.315
H	37.906	-13.576	20.675
H	33.686	-15.607	21.525
H	37.536	-15.838	20.051
H	35.322	-17.189	19.275
N	38.320	-10.007	23.839
C	38.821	-8.669	24.061
C	38.281	-7.740	22.964
O	38.317	-8.107	21.787
C	40.359	-8.638	24.093
C	40.871	-7.190	24.111
C	40.916	-9.444	25.289
H	38.934	-10.821	23.602
H	38.472	-8.335	24.937
H	40.692	-9.047	23.250
H	41.070	-6.902	23.177
H	40.169	-6.600	24.504
H	41.700	-7.143	24.663
H	41.505	-8.848	25.827
H	40.151	-9.764	25.841
H	41.436	-10.217	24.937
N	37.790	-6.574	23.387
C	37.518	-5.479	22.458
C	38.290	-4.231	22.863
O	38.648	-4.000	24.021
C	36.015	-5.200	22.384
C	35.397	-4.395	23.508
C	35.481	-2.883	23.280
C	33.968	-4.846	23.698
H	37.623	-6.515	24.407
H	37.819	-5.756	21.540
H	35.843	-4.706	21.527
H	35.546	-6.085	22.350
H	35.866	-4.618	24.375
H	34.999	-2.649	22.437
H	35.059	-2.409	24.051
H	36.440	-2.614	23.204
H	33.947	-5.821	23.930
H	33.542	-4.323	24.440
H	33.449	-4.699	22.853
N	38.617	-3.452	21.834
C	39.158	-2.131	22.018
C	38.536	-1.213	20.962
O	38.225	-1.643	19.848
C	40.686	-2.183	21.982
C	41.282	-2.537	20.633
O	41.107	-0.880	22.344
H	38.446	-3.885	20.899
H	38.895	-1.788	22.917
H	41.008	-2.833	22.679
H	40.961	-1.890	19.936
H	42.283	-2.495	20.679
H	41.006	-3.465	20.369
H	40.430	-0.440	22.948
N	38.392	0.040	21.332
C	37.896	1.069	20.435
C	38.144	2.484	20.957
O	38.977	2.710	21.850
H	38.664	0.236	22.325
H	38.341	0.971	19.528
H	36.899	0.942	20.292
N	37.408	3.423	20.370
C	37.535	4.839	20.618
C	36.144	5.443	20.577
O	35.237	4.998	19.853
C	38.466	5.504	19.590
C	39.888	4.967	19.495
C	40.796	5.337	20.645
N	40.945	6.794	20.756
C	41.369	7.423	21.846
N	41.868	6.707	22.841
N	41.269	8.746	21.936
H	36.700	3.026	19.685
H	37.920	4.983	21.527
H	38.039	5.411	18.682
H	38.521	6.485	19.813
H	39.854	3.956	19.434
H	40.311	5.307	18.639
H	40.399	4.987	21.486
H	41.689	4.932	20.484
H	40.709	7.349	19.950
H	41.956	5.720	22.796
H	42.175	7.183	23.685
H	40.889	9.264	21.165
H	41.569	9.218	22.759
N	35.975	6.518	21.344
C	34.754	7.279	21.271
C	35.052	8.774	21.307
O	36.238	9.162	21.517
C	33.758	6.803	22.332
C	34.081	7.315	23.719
C	35.149	6.835	24.442
C	33.314	8.324	24.273
C	35.434	7.330	25.715
C	33.581	8.821	25.539
C	34.635	8.313	26.255
O	34.827	8.840	27.501
H	36.768	6.740	21.966
H	34.274	7.136	20.281
H	32.855	7.130	22.081
H	33.777	5.810	22.355
H	35.737	6.119	24.059
H	32.549	8.706	23.750
H	36.216	6.970	26.226
H	33.006	9.545	25.924
H	35.735	8.585	27.833
N	34.110	9.695	20.935
C	34.405	11.139	20.853
C	34.080	11.810	22.180
O	32.990	12.402	22.369
C	33.718	11.781	19.626
C	34.451	13.018	19.167
O	34.651	13.983	19.951
O	34.864	13.121	17.979
H	33.130	9.372	20.773
H	35.497	11.277	20.686
H	33.688	11.038	18.798
H	32.668	12.056	19.850
N	35.004	11.749	23.185
C	34.842	12.208	24.596
C	34.448	13.673	24.781
O	34.331	14.120	25.928
C	36.115	11.930	25.311
O	37.188	12.603	24.674
H	35.779	11.072	22.996
H	34.111	11.638	25.010
H	36.055	12.253	26.251
H	36.306	10.952	25.298
H	36.859	13.037	23.836
N	34.199	14.550	23.761
C	33.335	15.729	24.000
C	32.124	15.661	23.122
O	32.248	15.813	21.873
C	34.130	17.021	23.799
H	34.400	14.292	22.772
H	33.004	15.776	25.056
H	34.863	16.919	22.971
H	33.449	17.871	23.573
H	34.640	17.262	24.760
N	30.833	15.446	23.693
C	29.609	15.332	22.897
C	29.374	16.500	21.978
O	29.837	17.644	22.245
C	28.476	15.188	23.912
C	29.141	14.636	25.162
C	30.588	15.101	25.091
H	29.677	14.411	22.280
H	28.028	16.178	24.156
H	27.674	14.512	23.544
H	28.637	14.988	26.088
H	29.117	13.529	25.129
H	30.729	15.998	25.732
H	31.259	14.276	25.423
N	28.612	16.315	20.784
C	27.955	17.458	20.142
C	26.780	17.918	20.981
O	25.876	17.098	21.289
C	27.512	17.061	18.732
H	28.139	15.384	20.695
H	28.678	18.297	20.044
H	26.860	16.163	18.777
H	26.947	17.889	18.251
H	28.398	16.823	18.107
N	26.676	19.274	21.432
C	25.594	19.700	22.353
C	24.433	20.298	21.604
O	23.663	21.094	22.202
C	26.092	20.675	23.444
C	27.100	20.044	24.409
O	26.627	21.838	22.863
H	27.247	19.991	20.936
H	25.170	18.818	22.864
H	25.215	20.949	24.078
H	27.765	20.824	24.836
H	26.545	19.608	25.261
H	27.722	19.269	23.912
H	27.563	21.641	22.605
N	23.957	19.647	20.422
C	22.668	19.986	19.794
C	21.644	18.850	19.804
O	20.792	18.864	18.911
C	22.908	20.598	18.391
C	23.504	19.646	17.382
O	24.038	18.562	17.742
O	23.621	20.000	16.177
H	24.524	18.880	20.005
H	22.190	20.735	20.414
H	21.944	20.985	17.993
H	23.586	21.475	18.492
N	21.775	17.857	20.686
C	20.985	16.641	20.634
C	21.638	15.534	19.831
O	21.037	14.436	19.750
H	22.502	18.037	21.421
H	20.811	16.307	21.583
H	20.069	16.847	20.234
N	22.822	15.699	19.155
C	23.347	14.680	18.216
C	24.129	13.592	18.922
O	25.235	13.870	19.461
C	24.222	15.313	17.121
O	23.418	15.868	16.111
H	23.363	16.582	19.290
H	22.493	14.201	17.701
H	24.914	16.069	17.554
H	24.860	14.536	16.654
H	23.295	16.828	16.368
N	23.764	12.219	18.727
C	24.437	11.077	19.365
C	25.936	11.062	19.185
O	26.441	11.083	18.028
H	22.787	12.051	18.400
H	24.183	11.072	20.445
H	24.049	10.137	18.919
N	26.782	10.773	20.300
C	28.254	10.889	20.243
C	28.876	9.714	19.525
O	28.817	8.558	20.025
C	28.826	11.045	21.662
C	30.344	11.124	21.683
O	28.332	12.246	22.179
H	26.313	10.635	21.224
H	28.509	11.820	19.694

H	28.500	10.176	22.285
H	30.683	11.992	21.083
H	30.679	11.268	22.733
H	30.804	10.190	21.304
H	28.295	12.123	23.170
N	29.586	9.904	18.298
C	30.047	8.770	17.478
C	31.080	7.904	18.187
O	32.028	8.444	18.820
C	30.636	9.288	16.172
H	29.476	10.840	17.852
H	29.169	8.145	17.220
H	31.523	9.911	16.407
H	30.940	8.439	15.521
H	29.881	9.892	15.624
N	31.118	6.558	17.944
C	32.127	5.649	18.511
C	32.517	4.526	17.561
O	31.925	4.382	16.459
C	31.656	5.142	19.899
C	30.198	4.671	20.024
C	29.953	3.410	19.200
C	29.869	4.407	21.499
H	30.360	6.152	17.366
H	33.071	6.213	18.655
H	32.283	4.309	20.273
H	31.768	5.994	20.591
H	29.512	5.464	19.671
H	30.725	2.647	19.423
H	28.953	2.993	19.419
H	29.978	3.655	18.124
H	29.978	5.263	22.006
H	28.915	4.110	21.550
H	30.457	3.705	21.903
N	33.495	3.643	17.911
C	33.543	2.277	17.388
C	34.548	1.355	18.072
O	35.291	1.804	18.962
H	34.030	3.867	18.757
H	32.543	1.833	17.559
H	33.753	2.299	16.299
N	34.482	0.054	17.757
C	35.342	-0.902	18.415
C	35.554	-2.078	17.487
O	34.832	-2.231	16.503
C	34.803	-1.258	19.809
C	33.527	-2.065	19.804
C	33.456	-3.419	19.764
C	32.167	-1.605	19.832
C	31.340	-2.755	19.828
C	31.542	-0.375	19.864
N	32.166	-3.836	19.829
C	29.953	-2.695	19.846
C	30.164	-0.313	19.899
C	29.376	-1.461	19.914
H	33.788	-0.182	17.032
H	36.231	-0.469	18.565
H	35.487	-1.797	20.291
H	34.614	-0.416	20.306
H	34.254	-4.031	19.695
H	31.862	-4.795	19.872
H	32.077	0.477	19.863
H	29.408	-3.540	19.809
H	29.722	0.588	19.913
H	28.386	-1.371	19.975
N	36.585	-2.851	17.830
C	37.004	-4.048	17.153
C	37.073	-5.210	18.136
O	37.618	-5.061	19.236
C	38.395	-3.891	16.540
C	38.808	-5.077	15.696
O	38.422	-2.726	15.714
H	37.096	-2.480	18.697
H	36.328	-4.280	16.444
H	39.077	-3.765	17.290
H	38.104	-5.792	15.739
H	38.927	-4.795	14.740
H	39.675	-5.454	16.033
H	39.272	-2.696	15.176
N	36.579	-6.354	17.670
C	36.920	-7.662	18.227
C	37.577	-8.529	17.168
O	37.028	-8.725	16.079
C	35.650	-8.361	18.721
C	35.926	-9.811	19.110
C	35.022	-7.572	19.835
H	35.919	-6.237	16.863
H	37.580	-7.521	18.970
H	34.998	-8.386	17.965
H	35.774	-10.402	18.318
H	36.873	-9.898	19.415
H	35.310	-10.079	19.850
H	35.081	-8.089	20.692
H	35.502	-6.698	19.944
H	34.058	-7.394	19.624
N	38.754	-9.074	17.495
C	39.309	-10.179	16.740
C	38.861	-11.468	17.443
O	38.925	-11.551	18.665
C	40.801	-10.085	16.673
H	39.216	-8.641	18.316
H	38.933	-10.177	15.823
H	41.106	-9.183	16.990
H	41.219	-10.789	17.254
H	41.115	-10.218	15.729
N	38.410	-12.444	16.647
C	37.682	-13.605	17.119
C	38.661	-14.733	17.458
O	38.552	-15.858	16.971
C	36.625	-14.021	16.101
C	35.558	-12.974	15.968
C	35.406	-12.129	14.917
C	34.553	-12.617	16.920
C	33.829	-11.535	16.375
C	34.188	-13.068	18.194
N	34.360	-11.281	15.137
C	32.755	-10.946	17.039
C	33.150	-12.460	18.859
C	32.423	-11.422	18.286
H	38.637	-12.288	15.628
H	37.211	-13.355	17.968
H	37.047	-14.124	15.219
H	36.189	-14.848	16.406
H	35.980	-12.126	14.094
H	34.028	-10.582	14.500
H	34.681	-13.833	18.620
H	32.244	-10.192	16.607
H	32.914	-12.776	19.782
H	31.659	-11.024	18.783
N	39.609	-14.383	18.319
C	40.544	-15.319	18.900
C	40.537	-15.110	20.408
O	40.565	-13.989	20.912
C	41.940	-15.098	18.322
C	43.016	-16.012	18.882
C	44.392	-15.772	18.323
C	45.467	-16.547	19.062
N	45.467	-16.246	20.521
H	39.618	-13.353	18.542
H	40.247	-16.250	18.705
H	41.894	-15.239	17.331
H	42.207	-14.149	18.505
H	43.045	-15.896	19.890
H	42.750	-16.974	18.700
H	44.409	-16.051	17.361
H	44.608	-14.796	18.387
H	45.310	-17.530	18.930
H	46.364	-16.309	18.678
H	45.440	-17.102	21.035
H	46.297	-15.740	20.754
H	44.664	-15.695	20.745
N	40.419	-16.222	21.150
C	40.626	-16.196	22.589
C	41.336	-17.513	22.960
O	41.916	-18.193	22.101
C	39.307	-15.924	23.342
C	38.226	-16.993	23.184
H	40.174	-17.073	20.614
H	41.263	-15.465	22.790
H	39.520	-15.833	24.321
H	38.936	-15.049	23.013
N	37.030	-16.698	23.675
O	38.455	-18.057	22.597
H	36.874	-15.811	24.114
H	36.282	-17.358	23.608
N	41.268	-17.893	24.237
C	41.893	-19.134	24.697
C	41.184	-20.402	24.207
O	41.795	-21.462	24.128
C	42.046	-19.101	26.213
C	43.151	-18.142	26.576
H	40.748	-17.249	24.858
H	42.832	-19.156	24.310
H	41.203	-18.783	26.607
H	42.288	-20.002	26.524
N	42.932	-17.328	27.592
O	44.180	-18.115	25.904
H	42.058	-17.374	28.087
H	43.627	-16.668	27.873
N	39.916	-20.296	23.826
C	39.122	-21.454	23.472
C	39.046	-21.636	21.956
O	39.023	-22.773	21.482
C	37.770	-21.309	24.170
C	37.914	-21.076	25.657
C	38.542	-22.016	26.464
C	37.455	-19.909	26.257
C	38.699	-21.811	27.827
C	37.603	-19.689	27.616
C	38.232	-20.642	28.404
O	38.396	-20.435	29.753
H	39.551	-19.319	23.806
H	39.560	-22.277	23.860
H	37.296	-20.533	23.777
H	37.257	-22.145	24.035
H	38.891	-22.865	26.054
H	37.008	-19.212	25.693
H	39.150	-22.506	28.392
H	37.258	-18.846	28.031
H	37.675	-19.827	30.082
N	39.029	-20.543	21.174
C	38.680	-20.646	19.760
C	39.406	-19.606	18.908
O	39.835	-18.577	19.415
C	37.164	-20.479	19.623
C	36.420	-21.791	19.808
C	35.278	-21.967	18.836
N	33.985	-21.882	19.484
C	32.851	-22.257	18.913
N	31.736	-22.328	19.622
N	32.853	-22.600	17.637
H	39.275	-19.664	21.648
H	38.936	-21.567	19.428
H	36.857	-19.848	20.319
H	36.970	-20.138	18.716
H	37.061	-22.555	19.683
H	36.048	-21.831	20.740
H	35.348	-21.252	18.140
H	35.374	-22.864	18.403
H	33.945	-21.518	20.420
H	31.722	-22.083	20.589
H	30.883	-22.634	19.180
H	33.698	-22.558	17.101
H	32.004	-22.904	17.196
N	39.609	-19.918	17.633
C	40.128	-18.928	16.699
C	39.348	-19.030	15.393
O	39.450	-20.017	14.664
C	41.644	-19.001	16.500
C	42.165	-17.796	15.738
H	39.375	-20.889	17.373
H	39.938	-18.026	17.083
H	42.077	-19.025	17.391
H	41.854	-19.820	15.982
N	43.481	-17.697	15.623

O	41.374	-16.972	15.249
H	44.074	-18.398	16.022
H	43.887	-16.922	15.136
N	38.552	-17.991	15.095
C	37.740	-18.009	13.895
C	38.409	-17.292	12.731
O	37.782	-17.153	11.680
C	36.398	-17.418	14.202
H	38.576	-17.217	15.774
H	37.576	-18.973	13.625
H	35.797	-17.490	13.402
H	35.973	-17.905	14.970
H	36.494	-16.449	14.447
N	39.648	-16.818	12.927
C	40.444	-16.200	11.880
C	39.671	-15.067	11.191
O	39.600	-14.999	9.953
C	40.940	-17.257	10.887
C	41.736	-18.340	11.552
H	39.991	-16.936	13.913
H	41.272	-15.802	12.294
H	40.152	-17.676	10.435
H	41.519	-16.814	10.202
C	41.582	-19.684	11.550
N	42.812	-18.077	12.376
C	43.283	-19.232	12.852
N	42.545	-20.208	12.381
H	43.178	-17.177	12.584
H	40.899	-20.202	11.041
H	44.066	-19.324	13.461
N	39.099	-14.197	12.027
C	38.219	-13.130	11.554
C	38.160	-12.016	12.600
O	38.563	-12.203	13.758
C	36.852	-13.678	11.198
O	36.189	-14.158	12.346
H	39.332	-14.344	13.030
H	38.614	-12.746	10.719
H	36.306	-12.951	10.788
H	36.961	-14.428	10.550
H	36.621	-13.782	13.166
N	37.635	-10.851	12.187
C	37.529	-9.692	13.064
C	36.313	-8.848	12.684
O	35.985	-8.766	11.505
C	38.802	-8.878	12.969
H	37.316	-10.855	11.196
H	37.414	-10.008	14.011
H	39.436	-9.314	12.329
H	38.590	-7.955	12.645
H	39.234	-8.819	13.870
N	35.599	-8.307	13.679
C	34.453	-7.442	13.453
C	34.776	-6.045	13.963
O	35.333	-5.898	15.054
C	33.189	-7.982	14.145
C	31.963	-7.118	13.949
O	32.987	-9.270	13.556
H	35.943	-8.565	14.634
H	34.280	-7.387	12.469
H	33.379	-8.103	15.120
H	32.231	-6.154	13.862
H	31.475	-7.396	13.117
H	31.345	-7.213	14.735
H	33.251	-9.257	12.585
N	34.423	-5.026	13.178
C	34.362	-3.660	13.680
C	32.922	-3.180	13.659
O	32.149	-3.405	12.720
C	35.247	-2.687	12.879
C	36.706	-3.046	12.942
O	34.783	-2.639	11.526
H	34.201	-5.289	12.199
H	34.686	-3.653	14.628
H	35.157	-1.755	13.281
H	36.818	-4.045	12.980
H	37.186	-2.699	12.129
H	37.129	-2.644	13.761
H	34.426	-3.537	11.246
N	32.563	-2.492	14.749
C	31.324	-1.792	14.919
C	31.553	-0.284	14.852
O	31.760	0.342	15.923
C	30.646	-2.129	16.246
C	30.109	-3.530	16.369
C	28.796	-3.910	16.304
C	30.834	-4.705	16.736
C	29.890	-5.755	16.859
C	32.174	-4.981	17.019
N	28.680	-5.245	16.547
C	30.259	-7.058	17.177
C	32.539	-6.268	17.360
C	31.583	-7.283	17.446
H	33.316	-2.516	15.506
H	30.727	-2.011	14.146
H	31.306	-2.004	16.989
H	29.873	-1.507	16.383
H	28.034	-3.292	16.106
H	27.819	-5.770	16.500
H	32.865	-4.257	16.975
H	29.575	-7.789	17.205
H	33.495	-6.471	17.547
H	31.884	-8.204	17.714
N	31.417	0.437	13.694
C	31.267	1.914	13.737
C	29.835	2.304	3.938
O	29.012	2.148	12.998
C	31.746	2.605	12.465
O	31.582	3.993	12.586
H	31.203	-0.081	12.812
H	31.874	2.332	14.568
H	32.803	2.376	12.250
H	31.132	2.252	11.617
H	32.281	4.311	13.224
N	29.454	2.966	15.061
C	28.109	3.520	15.207
C	28.083	4.755	16.040
O	28.793	5.745	15.741
H	30.115	2.992	15.865
H	27.648	3.760	14.226
H	27.504	2.768	15.718
N	27.180	4.828	17.133
C	26.958	6.039	17.930
C	26.146	5.724	19.161
O	25.381	4.731	19.182
C	26.308	7.173	17.104
C	24.971	6.784	16.459
C	24.462	7.905	15.603
H	26.656	3.959	17.370
H	27.944	6.406	18.281
H	26.155	8.059	17.760
H	27.012	7.490	16.307
H	25.100	5.871	15.844
H	24.216	6.577	17.245
N	24.470	7.833	14.322
O	24.017	8.943	16.135
H	24.211	8.693	13.796
H	24.868	7.020	13.820
N	26.263	6.495	20.258
C	25.504	6.327	21.503
C	24.497	7.469	21.532
O	24.751	8.604	21.061
C	26.526	6.418	22.629
C	25.909	6.808	23.948
C	25.271	5.856	24.729
C	25.903	8.127	24.368
C	24.665	6.200	25.924
C	25.347	8.478	25.593
C	24.716	7.513	26.359
O	24.147	7.801	27.583
H	26.787	7.391	20.138
H	25.002	5.468	21.508
H	26.956	5.529	22.735
H	27.201	7.105	22.386
H	25.247	4.900	24.423
H	26.301	8.834	23.790
H	24.194	5.506	26.471
H	25.403	9.421	25.917
H	24.720	7.421	28.306
N	23.248	7.136	21.897
C	22.153	8.074	22.013
C	21.616	7.909	23.438
O	21.083	6.860	23.778
C	21.056	7.787	20.963
C	19.948	8.822	20.994
C	21.607	7.666	19.549
H	23.140	6.102	22.095
H	22.507	8.992	21.929
H	20.643	6.905	21.195
H	19.065	8.375	20.842
H	19.938	9.278	21.885
H	20.102	9.502	20.276
H	22.305	6.807	19.475
H	20.851	7.516	18.912
H	22.147	8.595	19.265
N	21.768	8.941	24.255
C	21.415	8.852	25.665
C	19.927	9.108	25.873
O	19.201	9.365	24.926
H	22.151	9.798	23.813
H	21.646	7.935	26.013
H	21.945	9.530	26.189
N	19.501	9.099	27.135
C	18.118	9.368	27.482
C	17.541	8.174	28.222
O	18.288	7.286	28.639
H	20.240	8.885	27.838
H	18.067	10.179	28.068
H	17.586	9.532	26.649
N	16.215	8.140	28.359
C	15.603	7.207	29.296
C	15.821	5.790	28.795
O	16.052	4.877	29.600
C	14.140	7.515	29.466
H	15.692	8.800	27.772
H	16.046	7.305	30.188
H	13.596	6.684	29.327
H	13.968	7.865	30.390
H	13.855	8.206	28.798
N	15.753	5.649	27.459
C	15.951	4.383	26.783
C	17.199	4.502	25.907
O	17.096	4.633	24.691
C	14.714	4.017	25.960
C	13.423	4.022	26.774
C	12.607	2.749	26.654
O	12.429	2.267	25.521
O	12.148	2.247	27.698
H	15.543	6.545	26.956
H	16.083	3.653	27.448
H	14.618	4.685	25.249
H	14.841	3.109	25.612
H	13.662	4.135	27.726
H	12.860	4.768	26.453
N	18.359	4.430	26.570
C	19.655	4.657	25.955
C	19.981	3.520	24.984
O	19.687	2.344	25.216
C	20.679	4.797	27.042
H	18.244	4.191	27.589
H	19.628	5.517	25.438
H	20.593	5.694	27.484
H	20.549	4.082	27.733
H	21.603	4.713	26.659
N	20.658	3.870	23.878
C	20.924	2.909	22.827
C	22.311	3.191	22.249
O	22.745	4.358	22.232
C	19.873	3.074	21.710
C	18.682	2.125	21.778
C	17.633	2.459	20.732
N	16.689	1.370	20.535
C	15.881	0.862	21.457
N	15.080	-0.138	21.137
N	15.857	1.346	22.688

H	20.959	4.859	23.854
H	20.923	1.988	23.212
H	19.525	4.012	21.752
H	20.333	2.932	20.833
H	18.995	1.191	21.620
H	18.257	2.193	22.678
H	17.142	3.273	21.036
H	18.104	2.649	19.873
H	16.642	0.957	19.603
H	15.074	-0.541	20.228
H	14.449	-0.512	21.838
H	16.481	2.101	22.925
H	15.238	0.974	23.369
N	22.955	2.143	21.764
C	24.073	2.304	20.828
C	23.635	1.684	19.510
O	23.340	0.499	19.435
C	25.371	1.694	21.371
C	25.767	2.356	22.703
C	26.499	1.792	20.345
C	26.918	1.693	23.386
H	22.615	1.222	22.090
H	24.164	3.277	20.671
H	25.211	0.728	21.549
H	25.993	3.312	22.515
H	24.966	2.332	23.303
H	27.310	1.332	20.700
H	26.212	1.355	19.495
H	26.745	2.854	20.149
H	27.726	1.718	22.794
H	27.126	2.169	24.243
H	26.690	0.738	23.589
N	23.444	2.555	18.460
C	23.124	2.128	17.120
C	24.335	1.960	16.259
O	25.049	2.951	15.957
C	22.099	3.032	16.427
C	20.867	3.228	17.232
H	23.514	3.580	18.624
H	22.742	1.155	17.271
H	22.574	4.008	16.189
H	21.791	2.566	15.468
N	20.432	4.457	17.399
O	20.266	2.231	17.623
H	20.968	5.286	17.075
H	19.550	4.622	17.860
N	24.532	0.763	15.650

C	25.772	0.406	14.941
C	25.524	-0.133	13.574
O	24.448	-0.728	13.293
C	26.689	-0.546	15.754
C	26.878	-0.036	17.165
O	26.249	-1.880	15.821
H	23.765	0.073	15.774
H	26.350	1.321	14.769
H	27.688	-0.550	15.262
H	25.947	-0.172	17.753
H	27.672	-0.632	17.618
H	27.190	1.026	17.179
H	25.277	-1.867	16.069
N	26.433	0.111	12.594
C	26.695	-0.857	11.533
C	28.046	-1.510	11.714
O	28.939	-0.942	12.398
C	26.525	-0.198	10.159
C	25.030	-0.169	9.813
C	24.696	0.812	8.753
H	27.178	0.813	12.773
H	25.964	-1.677	11.610
H	26.959	0.825	10.180
H	27.059	-0.781	9.375
H	24.730	-1.183	9.475
H	24.424	0.092	10.706
N	24.720	2.128	9.021
O	24.034	0.429	7.766
H	24.408	2.788	8.280
H	25.182	2.506	9.872
N	28.201	-2.807	11.329
C	29.410	-3.555	11.519
C	29.916	-4.172	10.209
O	29.124	-4.429	9.291
C	29.243	-4.613	12.617
C	28.111	-5.570	12.491
C	26.947	-5.541	13.209
C	28.067	-6.771	11.698
C	26.828	-7.390	11.951
C	28.920	-7.347	10.753
N	26.167	-6.609	12.856
C	26.419	-8.554	11.299
C	28.542	-8.527	10.152
C	27.307	-9.108	10.405
H	27.386	-3.134	10.786
H	30.130	-2.923	11.843
H	30.093	-5.159	12.663

H	29.135	-4.137	13.503
H	26.706	-4.850	13.883
H	25.242	-6.794	13.210
H	29.787	-6.913	10.515
H	25.524	-8.955	11.482
H	29.178	-8.972	9.519
H	27.057	-9.952	9.924
N	31.226	-4.412	10.206
C	31.938	-5.179	9.199
C	32.704	-6.325	9.852
O	33.540	-6.091	10.722
C	32.932	-4.280	8.475
C	32.372	-3.110	7.664
C	33.499	-2.147	7.255
C	31.654	-3.619	6.418
H	31.726	-3.975	11.039
H	31.271	-5.570	8.558
H	33.553	-3.892	9.162
H	33.461	-4.851	7.843
H	31.707	-2.614	8.219
H	34.164	-2.641	6.699
H	33.110	-1.394	6.728
H	33.940	-1.793	8.077
H	30.900	-4.217	6.689
H	31.295	-2.842	5.900
H	32.298	-4.131	5.849
N	32.473	-7.524	9.311
C	33.130	-8.752	9.725
C	33.963	-9.292	8.575
O	33.406	-9.710	7.552
C	32.045	-9.747	10.161
C	32.519	-11.086	10.734
C	31.314	-11.939	11.099
C	33.417	-11.818	9.796
H	31.753	-7.499	8.537
H	33.741	-8.549	10.492
H	31.491	-9.303	10.864
H	31.479	-9.948	9.364
H	33.064	-10.914	11.549
H	30.761	-12.105	10.282
H	31.624	-12.814	11.473
H	30.761	-11.462	11.783
H	34.235	-11.268	9.594
H	33.710	-12.691	10.201
H	32.939	-12.010	8.932
N	35.294	-9.217	8.742
C	36.248	-9.682	7.753

C	36.808	-11.031	8.206
O	37.229	-11.223	9.354
C	37.390	-8.693	7.476
C	38.323	-9.174	6.387
O	36.846	-7.431	7.059
H	35.577	-8.789	9.658
H	35.775	-9.817	6.884
H	37.914	-8.542	8.339
H	38.434	-10.170	6.444
H	37.949	-8.940	5.485
H	39.223	-8.741	6.488
H	36.686	-6.841	7.856
N	36.820	-11.963	7.251
C	37.397	-13.291	7.438
C	38.683	-13.332	6.627
O	38.705	-12.866	5.477
C	36.387	-14.364	6.985
C	35.091	-14.308	7.798
C	36.997	-15.753	6.980
C	33.881	-13.847	6.989
H	36.377	-11.657	6.352
H	37.659	-13.350	8.392
H	36.159	-14.186	6.028
H	34.904	-15.216	8.163
H	35.222	-13.674	8.557
H	36.351	-16.402	6.579
H	37.838	-15.749	6.441
H	37.206	-16.031	7.917
H	33.736	-14.480	6.233
H	33.080	-13.836	7.582
H	34.056	-12.931	6.637
N	39.768	-13.820	7.244
C	41.000	-14.084	6.520
C	40.749	-15.007	5.325
O	40.181	-16.066	5.499
H	39.646	-13.986	8.260
H	41.392	-13.218	6.188
H	41.669	-14.518	7.135
N	41.083	-14.568	4.109
C	40.804	-15.362	2.917
C	42.069	-15.404	2.062
O	42.997	-14.633	2.281
C	39.642	-14.841	2.041
C	38.334	-14.610	2.771
O	40.052	-13.634	1.403
H	41.543	-13.643	4.090
H	40.582	-16.292	3.196

H	39.441	-15.534	1.323
H	37.566	-14.679	2.129
H	38.218	-15.298	3.493
H	38.330	-13.697	3.187
H	40.556	-13.836	0.555
N	42.087	-16.282	1.054
C	43.037	-16.156	-0.038
C	42.649	-14.945	-0.888
O	41.500	-14.479	-0.878
C	43.079	-17.449	-0.874
C	43.222	-18.690	-0.020
O	41.857	-17.529	-1.611
H	41.381	-17.036	1.124
H	43.946	-15.993	0.340
H	43.847	-17.388	-1.532
H	43.635	-18.454	0.864
H	42.321	-19.102	0.142
H	43.808	-19.361	-0.483
H	42.000	-18.023	-2.476
N	43.601	-14.490	-1.698
C	43.383	-13.375	-2.606
C	42.254	-13.724	-3.584
O	41.454	-12.856	-3.916
C	44.708	-13.002	-3.288
C	45.792	-12.582	-2.292
C	47.225	-12.534	-2.806
O	48.047	-13.372	-2.360
O	47.533	-11.637	-3.599
H	44.509	-14.999	-1.624
H	43.130	-12.556	-2.090
H	45.033	-13.793	-3.762
H	44.538	-12.241	-3.877
H	45.565	-11.667	-1.962
H	45.773	-13.227	-1.528
N	42.148	-15.008	-3.975
C	41.128	-15.496	-4.898
C	39.708	-15.296	-4.351
O	38.813	-14.949	-5.122
C	41.371	-16.963	-5.187
H	42.877	-15.634	-3.548
H	41.206	-14.988	-5.769
H	40.695	-17.294	-5.847
H	42.289	-17.085	-5.566
H	41.291	-17.492	-4.341
N	39.531	-15.572	-3.047
C	38.264	-15.470	-2.322
C	38.066	-14.123	-1.590

O	37.082	-13.964	-0.888
C	38.138	-16.604	-1.303
C	38.024	-17.954	-1.978
H	40.428	-15.883	-2.570
H	37.518	-15.536	-3.008
H	38.948	-16.601	-0.729
H	37.322	-16.448	-0.760
N	38.347	-19.002	-1.247
O	37.628	-18.036	-3.137
H	38.643	-18.886	-0.296
H	38.298	-19.926	-1.637
N	38.911	-13.119	-1.823
C	38.825	-11.844	-1.108
C	37.484	-11.136	-1.357
O	36.919	-10.507	-0.457
C	39.996	-10.948	-1.493
H	39.630	-13.317	-2.549
H	38.911	-12.020	-0.118
H	39.783	-10.001	-1.256
H	40.814	-11.238	-0.999
H	40.159	-11.017	-2.477
N	36.944	-11.271	-2.568
C	35.654	-10.702	-2.914
C	34.564	-11.147	-1.941
O	33.628	-10.393	-1.721
C	35.276	-11.016	-4.372
C	34.911	-12.448	-4.601
C	35.776	-13.478	-4.866
C	33.604	-13.042	-4.462
C	33.755	-14.425	-4.734
C	32.316	-12.532	-4.221
N	35.087	-14.656	-4.972
C	32.672	-15.308	-4.705
C	31.243	-13.402	-4.217
C	31.420	-14.767	-4.471
H	37.529	-11.819	-3.239
H	35.728	-9.698	-2.844
H	34.491	-10.463	-4.622
H	36.053	-10.808	-4.953
H	36.765	-13.380	-4.967
H	35.486	-15.549	-5.189
H	32.179	-11.558	-4.055
H	32.808	-16.288	-4.849
H	30.330	-13.049	-4.031
H	30.618	-15.365	-4.483
N	34.685	-12.330	-1.317
C	33.679	-12.777	-0.374
C	34.198	-12.835	1.080
O	33.707	-13.622	1.887
C	33.114	-14.112	-0.855
C	34.097	-15.263	-0.911
C	33.401	-16.573	-1.166
C	34.398	-17.703	-1.221
N	33.862	-18.807	-2.042
H	35.531	-12.865	-1.567
H	32.891	-12.138	-0.398
H	32.360	-14.370	-0.240
H	32.736	-13.974	-1.778
H	34.756	-15.092	-1.647
H	34.588	-15.315	-0.038
H	32.759	-16.750	-0.428
H	32.924	-16.526	-2.037
H	35.248	-17.371	-1.625
H	34.572	-18.032	-0.295
H	32.877	-18.681	-2.175
H	34.317	-18.816	-2.934
H	34.024	-19.681	-1.580
N	35.110	-11.920	1.464
C	35.729	-11.940	2.788
C	34.966	-11.100	3.817
O	35.273	-11.209	4.996
C	37.143	-11.442	2.678
O	37.114	-10.071	2.290
H	35.328	-11.208	0.738
H	35.733	-12.899	3.117
H	37.591	-11.512	3.555
H	37.624	-11.955	1.984
H	37.015	-10.011	1.299
N	34.042	-10.206	3.405
C	33.501	-9.215	4.347
C	31.982	-9.229	4.408
O	31.299	-8.984	3.420
C	33.980	-7.785	4.033
C	33.687	-6.785	5.140
O	35.390	-7.855	3.896
H	33.768	-10.281	2.423
H	33.861	-9.430	5.270
H	33.581	-7.497	3.151
H	32.698	-6.638	5.213
H	34.032	-7.134	6.015
H	34.134	-5.911	4.937
H	35.655	-8.646	3.329
N	31.440	-9.503	5.606
C	30.011	-9.379	5.855
C	29.714	-7.999	6.454
O	30.543	-7.443	7.158
C	29.553	-10.453	6.843
C	29.659	-11.900	6.392
C	29.452	-12.860	7.577
C	28.601	-12.160	5.342
H	32.127	-9.807	6.325
H	29.522	-9.454	4.986
H	30.105	-10.355	7.673
H	28.593	-10.274	7.061
H	30.565	-12.062	6.001
H	28.546	-12.707	7.966
H	29.527	-13.800	7.252
H	30.151	-12.683	8.266
H	28.743	-11.551	4.559
H	28.657	-13.112	5.033
H	27.692	-11.992	5.728
N	28.482	-7.531	6.236
C	27.995	-6.271	6.749
C	26.655	-6.505	7.435
O	25.839	-7.292	6.986
C	27.914	-5.210	5.629
C	26.916	-5.572	4.549
C	27.612	-3.848	6.221
H	27.890	-8.177	5.646
H	28.638	-5.936	7.438
H	28.818	-5.164	5.194
H	27.398	-5.749	3.689
H	26.410	-6.393	4.818
H	26.273	-4.816	4.417
H	26.626	-3.680	6.185
H	27.921	-3.822	7.173
H	28.090	-3.143	5.696
N	26.439	-5.800	8.541
C	25.156	-5.846	9.210
C	25.000	-4.670	10.160
O	25.714	-3.674	10.083
H	27.244	-5.237	8.866
H	24.415	-5.821	8.527
H	25.074	-6.705	9.730
N	23.973	-4.767	11.000
C	23.665	-3.677	11.923
C	23.239	-4.276	13.254
O	22.531	-5.273	13.268
C	22.590	-2.767	11.336
C	21.395	-3.483	10.812
C	20.166	-3.728	11.358
N	21.381	-4.061	9.557
C	20.178	-4.598	9.350
N	19.433	-4.415	10.422
H	23.436	-5.643	10.944
H	24.493	-3.141	12.068
H	22.286	-2.119	12.049
H	22.995	-2.229	10.582
H	22.138	-4.077	8.917
H	19.864	-3.455	12.265
H	19.893	-5.061	8.512
N	23.718	-3.704	14.358
C	23.348	-4.197	15.679
C	22.879	-2.985	16.485
O	23.495	-1.914	16.410
C	24.531	-4.825	16.416
C	25.020	-6.202	16.008
O	24.356	-6.855	15.130
O	26.104	-6.608	16.526
H	24.354	-2.906	14.198
H	22.563	-4.791	15.567
H	25.318	-4.174	16.340
H	24.290	-4.856	17.410
N	21.788	-3.143	17.249
C	21.394	-2.106	18.189
C	21.557	-2.634	19.616
O	21.033	-3.704	19.938
C	19.946	-1.655	18.021
C	19.558	-0.546	18.976
O	19.743	-1.203	16.686
H	21.283	-4.027	17.111
H	22.001	-1.319	18.076
H	19.328	-2.452	18.186
H	18.619	-0.686	19.303
H	20.179	-0.538	19.765
H	19.611	0.342	18.511
H	19.795	-0.198	16.641
N	22.239	-1.854	20.455
C	22.521	-2.243	21.836
C	21.655	-1.421	22.791
O	21.506	-0.195	22.667
C	24.009	-2.091	22.152
C	24.915	-2.938	21.310
C	25.317	-2.527	20.042
C	25.385	-4.140	21.772
C	26.155	-3.331	19.299
C	26.230	-4.943	21.026
C	26.587	-4.543	19.762
H	22.552	-0.948	20.043
H	22.235	-3.197	21.936
H	24.253	-1.140	22.006
H	24.144	-2.347	23.101
H	25.003	-1.662	19.678
H	25.107	-4.458	22.696
H	26.455	-3.002	18.376
H	26.572	-5.810	21.415
H	27.157	-5.130	19.186
N	21.083	-2.108	23.802
C	20.319	-1.416	24.834
C	20.844	-1.847	26.225
O	21.593	-2.807	26.337
C	18.812	-1.680	24.671
C	18.251	-1.263	23.328
O	18.534	-3.074	24.769
H	21.234	-3.125	23.773
H	20.465	-0.434	24.744
H	18.311	-1.199	25.425
H	17.257	-1.403	23.312
H	18.446	-0.293	23.160
H	18.668	-1.810	22.596
H	18.355	-3.332	25.726
N	20.481	-1.092	27.266
C	20.990	-1.294	28.619
C	20.151	-2.333	29.365
O	20.556	-2.798	30.426
C	21.001	0.019	29.407
C	22.019	1.067	28.965
C	22.981	1.477	30.067
C	24.045	0.415	30.275
N	24.485	0.282	31.690
H	19.794	-0.337	27.024
H	21.938	-1.626	28.566
H	20.083	0.430	29.342
H	21.179	-0.200	30.375
H	22.550	0.696	28.192
H	21.524	1.884	28.644
H	23.425	2.326	29.807
H	22.474	1.589	30.913
H	23.683	-0.475	29.959
H	24.847	0.640	29.702
H	24.420	-0.676	31.969
H	25.432	0.591	31.773
H	23.898	0.842	32.274
N	19.005	-2.693	28.778
C	18.141	-3.773	29.231

C	18.005	-4.774	28.081
O	17.968	-4.352	26.934
C	16.772	-3.202	29.663
C	16.907	-2.218	30.830
C	16.017	-2.559	28.495
H	18.774	-2.104	27.927
H	18.586	-4.238	29.989
H	16.215	-3.963	29.991
H	16.556	-1.326	30.551
H	16.383	-2.557	31.609
H	17.869	-2.137	31.082
H	15.056	-2.458	28.746
H	16.415	-1.664	28.304
H	16.099	-3.146	27.692
N	17.914	-6.081	28.365
C	17.869	-7.090	27.303
C	16.539	-6.996	26.547
O	16.538	-7.431	25.380
C	18.010	-8.527	27.821
C	18.771	-8.658	29.130
C	19.370	-10.015	29.362
C	20.114	-10.075	30.679
N	20.924	-11.313	30.782
O	15.463	-6.574	26.861
H	17.879	-6.306	29.373
H	18.617	-6.918	26.659
H	17.091	-8.900	27.952
H	18.488	-9.062	27.123
H	19.510	-7.963	29.143
H	18.140	-8.439	29.893
H	18.642	-10.697	29.379
H	20.012	-10.222	28.627
H	20.718	-9.278	30.750
H	19.451	-10.051	31.431
H	20.355	-12.104	30.556
H	21.693	-11.263	30.144
H	21.272	-11.408	31.715

Ir$_{(R)}$-*pro-R* with Sav S112I

N	52.818	-12.396	12.098
C	52.905	-11.169	11.250
C	52.919	-9.893	12.086
O	52.623	-8.824	11.554
C	51.707	-10.963	10.328
C	51.102	-12.223	9.754
O	51.779	-12.882	8.929
O	49.953	-12.514	10.138
H	52.775	-12.346	13.100
H	53.786	-11.223	10.764
H	50.998	-10.476	10.851
H	52.002	-10.373	9.568
N	53.236	-10.020	13.380
C	53.502	-8.890	14.250
C	54.491	-7.902	13.619
O	54.220	-6.702	13.633
C	54.010	-9.428	15.582
C	54.372	-8.362	16.577
C	54.579	-8.947	17.960
O	54.985	-8.196	18.868
O	54.329	-10.164	18.116
H	53.274	-11.017	13.707
H	52.640	-8.404	14.457
H	53.291	-9.979	15.974
H	54.819	-9.962	15.399
H	55.216	-7.949	16.304
H	53.635	-7.721	16.638
N	55.624	-8.387	13.075
C	56.634	-7.502	12.493
C	56.199	-7.009	11.109
O	56.467	-5.856	10.742
C	57.981	-8.188	12.423
H	55.710	-9.417	13.107
H	56.750	-6.703	13.094
H	58.166	-8.659	13.287
H	57.982	-8.856	11.677
H	58.698	-7.509	12.258
N	55.545	-7.901	10.344
C	54.923	-7.544	9.082
C	53.898	-6.402	9.208
O	53.882	-5.502	8.374
H	55.529	-8.866	10.743
H	55.637	-7.265	8.416
H	54.455	-8.356	8.690
N	53.029	-6.446	10.227
C	51.989	-5.426	10.388
C	52.565	-4.145	10.992
O	52.186	-3.043	10.594
C	50.802	-5.929	11.230
C	50.040	-7.043	10.517
C	49.856	-4.782	11.600
C	49.166	-7.842	11.460
H	53.155	-7.244	10.872
H	51.679	-5.192	9.462
H	51.167	-6.300	12.083
H	49.470	-6.637	9.812
H	50.701	-7.656	10.097
H	49.142	-5.127	12.205
H	50.373	-4.067	12.064
H	49.444	-4.415	10.770
H	48.498	-7.234	11.886
H	48.691	-8.555	10.947
H	49.736	-8.260	12.165
N	53.436	-4.278	11.998
C	54.129	-3.130	12.569
C	54.811	-2.322	11.463
O	55.483	-2.865	10.588
C	55.097	-3.610	13.657
C	55.887	-2.483	14.296
O	54.251	-4.275	14.599
H	53.570	-5.253	12.327
H	53.455	-2.538	13.019
H	55.725	-4.281	13.263
H	55.326	-1.652	14.336
H	56.157	-2.740	15.227
H	56.712	-2.295	13.757
H	54.164	-5.250	14.365
N	54.581	-1.011	11.451
C	55.228	-0.162	10.468
C	54.389	1.069	10.141
O	53.380	1.353	10.805
H	53.929	-0.672	12.173
H	56.129	0.136	10.817
H	55.388	-0.687	9.619
N	54.826	1.773	9.092
C	54.168	2.951	8.580
C	53.554	2.601	7.230
O	54.230	2.144	6.313
C	55.109	4.154	8.472
C	54.417	5.325	7.819
O	55.552	4.516	9.784
H	55.710	1.380	8.668
H	53.431	3.210	9.200
H	55.925	3.889	7.923
H	53.435	5.137	7.722
H	54.534	6.152	8.377
H	54.805	5.493	6.908
H	54.797	4.437	10.443
N	52.237	2.826	7.132
C	51.454	2.462	5.967
C	50.778	3.706	5.433
O	50.520	4.634	6.205
C	50.394	1.423	6.313
C	50.851	0.121	6.862
C	51.169	-0.170	8.163
C	51.022	-1.082	6.123
C	51.442	-2.062	7.043
C	50.832	-1.433	4.781
N	51.514	-1.477	8.270
C	51.662	-3.382	6.662
C	51.094	-2.727	4.402
C	51.497	-3.686	5.330
H	51.826	3.291	7.978
H	52.083	2.131	5.264
H	49.760	1.826	6.992
H	49.857	1.222	5.478
H	51.147	0.489	8.913
H	51.782	-1.946	9.119
H	50.514	-0.761	4.118
H	51.929	-4.072	7.336
H	50.994	-2.984	3.441
H	51.670	-4.623	5.014
N	50.518	3.702	4.121
C	49.794	4.758	3.441
C	48.658	4.155	2.603
O	48.779	3.118	1.955
C	50.734	5.535	2.482
C	52.018	5.999	3.115
C	52.065	7.214	3.782
C	53.170	5.215	3.102
C	53.214	7.646	4.413
C	54.338	5.640	3.727
C	54.355	6.861	4.370
O	55.431	7.398	4.979
H	50.892	2.852	3.623
H	49.381	5.361	4.127
H	50.952	4.931	1.719
H	50.234	6.332	2.151
H	51.243	7.795	3.806
H	53.157	4.332	2.637
H	53.226	8.521	4.901
H	55.154	5.066	3.708
H	55.697	6.832	5.763
N	47.532	4.859	2.540
C	46.474	4.424	1.644
C	46.528	5.230	0.351
O	47.371	6.121	0.199
C	45.092	4.462	2.318
C	44.592	5.838	2.657
H	47.484	5.683	3.143
H	46.660	3.469	1.401
H	44.435	4.020	1.697
H	45.148	3.918	3.163
N	43.493	5.908	3.419
O	45.111	6.825	2.133
H	43.060	5.063	3.732
H	43.116	6.793	3.669
N	45.554	4.962	-0.531
C	45.460	5.559	-1.861
C	45.114	7.047	-1.803
O	45.287	7.763	-2.794
C	44.483	4.743	-2.726
C	42.984	4.847	-2.354
C	42.543	4.298	-1.003
H	44.846	4.267	-0.171
H	46.364	5.472	-2.306
H	44.576	5.039	-3.686
H	44.739	3.768	-2.672
H	42.722	5.831	-2.401
H	42.452	4.372	-3.083
N	41.440	4.843	-0.477
O	43.123	3.361	-0.450
H	41.080	4.518	0.413
H	40.974	5.587	-0.987
N	44.668	7.544	-0.645
C	44.414	8.965	-0.447
C	45.660	9.683	0.052
O	45.621	10.903	0.201
C	43.308	9.146	0.597
C	41.930	8.594	0.238
C	40.999	8.691	1.446
C	41.372	9.322	-0.968
H	44.519	6.827	0.099
H	44.146	9.370	-1.330
H	43.603	8.689	1.435
H	43.204	10.124	0.764
H	42.020	7.639	-0.032
H	40.910	9.648	1.720
H	40.101	8.328	1.202
H	41.381	8.160	2.202
H	41.986	9.200	-1.750
H	40.469	8.954	-1.197
H	41.288	10.299	-0.764
N	46.672	8.920	0.444
C	47.876	9.487	1.035

C	47.785	9.698	2.550
O	48.630	10.360	3.148
H	46.533	7.900	0.295
H	48.668	8.878	0.836
H	48.081	10.381	0.592
N	46.796	9.075	3.198
C	46.729	9.077	4.653
C	47.850	8.223	5.214
O	48.216	7.254	4.566
C	45.365	8.560	5.097
O	44.351	9.401	4.559
H	46.104	8.606	2.589
H	46.866	10.018	4.970
H	45.238	7.643	4.753
H	45.314	8.587	6.083
H	44.309	9.276	3.570
N	48.262	8.512	6.459
C	49.386	7.830	7.092
C	48.897	7.069	8.325
O	48.239	7.661	9.164
C	50.479	8.814	7.531
C	51.702	8.075	8.036
O	50.808	9.628	6.399
H	47.709	9.262	6.926
H	49.766	7.167	6.444
H	50.109	9.415	8.258
H	52.384	8.730	8.371
H	51.445	7.459	8.786
H	52.107	7.534	7.294
H	50.055	9.621	5.732
N	49.149	5.764	8.335
C	48.728	4.832	9.377
C	50.002	4.269	10.006
O	50.679	3.443	9.386
C	47.872	3.763	8.699
C	47.483	2.534	9.467
C	47.073	2.612	10.782
C	47.483	1.294	8.854
C	46.658	1.480	11.469
C	47.099	0.158	9.543
C	46.675	0.249	10.850
H	49.705	5.441	7.488
H	48.274	5.363	10.079
H	47.018	4.212	8.390
H	48.370	3.459	7.871
H	47.071	3.493	11.253
H	47.765	1.217	7.893
H	46.346	1.570	12.426
H	47.134	-0.745	9.075
H	46.382	-0.566	11.347
N	50.306	4.699	11.234
C	51.496	4.232	11.931
C	51.040	3.280	13.024
O	50.340	3.697	13.935
C	52.303	5.404	12.520
C	52.753	6.388	11.440
C	53.478	4.911	13.368
C	53.410	7.627	11.984
H	49.633	5.379	11.634
H	52.011	3.690	11.277
H	51.709	5.895	13.158
H	53.392	5.917	10.833
H	51.948	6.654	10.908
H	53.912	5.690	13.814
H	53.142	4.274	14.058
H	54.139	4.451	12.780
H	54.222	7.373	12.510
H	53.678	8.224	11.228
H	52.769	8.113	12.579
N	51.398	2.015	12.898
C	50.845	1.010	13.786
C	51.927	0.111	14.378
O	52.934	-0.214	13.746
C	49.783	0.174	13.059
C	50.370	-0.675	11.944
C	48.978	-0.665	14.052
H	52.074	1.815	12.147
H	50.384	1.482	14.553
H	49.134	0.810	12.631
H	50.212	-0.228	11.063
H	51.354	-0.785	12.088
H	49.933	-1.575	11.942
H	48.756	-1.544	13.632
H	49.525	-0.811	14.875
H	48.138	-0.178	14.285
N	51.637	-0.324	15.602
C	52.376	-1.355	16.314
C	51.425	-2.508	16.658
O	50.411	-2.306	17.320
C	52.966	-0.793	17.613
C	53.672	-1.834	18.457
O	53.882	0.218	17.214
H	50.794	0.161	16.027
H	53.093	-1.712	15.716
H	52.228	-0.366	18.149
H	54.458	-1.421	18.926
H	53.041	-2.205	19.144
H	53.997	-2.585	17.875
H	53.848	0.355	16.216
N	51.754	-3.701	16.176
C	51.083	-4.921	16.573
C	51.803	-5.531	17.783
O	53.021	-5.663	17.753
C	51.082	-5.840	15.408
H	52.542	-3.679	15.484
H	50.142	-4.710	16.842
H	50.227	-5.747	14.885
H	51.853	-5.634	14.795
H	51.165	-6.796	15.712
N	51.059	-5.826	18.848
C	51.582	-6.494	20.033
C	51.378	-8.005	19.956
O	50.669	-8.501	19.076
H	50.054	-5.533	18.758
H	52.567	-6.297	20.124
H	51.116	-6.139	20.854
N	52.030	-8.746	20.865
C	52.022	-10.207	20.826
C	50.621	-10.758	21.119
O	50.269	-11.855	20.692
C	53.032	-10.740	21.825
H	52.533	-8.194	21.587
H	52.296	-10.510	19.906
H	52.757	-11.653	22.127
H	53.934	-10.792	21.396
H	53.077	-10.130	22.616
N	49.845	-9.947	21.853
C	48.563	-10.268	22.464
C	47.367	-9.939	21.557
O	46.240	-9.923	22.046
C	48.430	-9.428	23.743
C	48.324	-7.919	23.519
O	49.009	-7.381	22.603
O	47.549	-7.282	24.268
H	50.283	-8.968	21.959
H	48.543	-11.270	22.617
H	47.608	-9.733	24.227
H	49.233	-9.607	24.313
N	47.621	-9.605	20.280
C	46.563	-9.256	19.341
C	46.193	-7.768	19.328
O	45.273	-7.395	18.626
H	48.632	-9.619	20.030
H	46.844	-9.531	18.399
H	45.727	-9.799	19.558
N	46.898	-6.901	20.066
C	46.575	-5.475	20.110
C	47.160	-4.746	18.906
O	48.247	-5.067	18.460
C	47.110	-4.835	21.358
H	47.684	-7.327	20.597
H	45.574	-5.370	20.094
H	47.998	-5.236	21.598
H	47.228	-3.849	21.216
H	46.473	-4.979	22.120
N	46.434	-3.741	18.397
C	46.984	-2.748	17.477
C	46.916	-1.399	18.190
O	45.907	-1.074	18.801
C	46.165	-2.702	16.172
C	46.093	-3.993	15.360
C	45.229	-3.809	14.122
C	47.477	-4.444	14.909
H	45.438	-3.732	18.718
H	47.934	-2.964	17.302
H	45.231	-2.446	16.414
H	46.569	-2.001	15.589
H	45.703	-4.717	15.932
H	45.618	-3.089	13.546
H	45.196	-4.666	13.606
H	44.302	-3.550	14.396
H	48.053	-4.605	15.711
H	47.397	-5.290	14.381
H	47.890	-3.733	14.339
N	47.993	-0.616	18.141
C	47.994	0.721	18.721
C	48.772	1.629	17.789
O	49.658	1.177	17.065
C	48.651	0.696	20.112
C	47.919	-0.183	21.098
O	49.983	0.201	19.938
H	48.814	-1.035	17.663
H	47.055	1.047	18.773
H	48.705	1.641	20.466
H	48.549	-0.860	21.492
H	47.537	0.375	21.841
H	47.169	-0.668	20.638
H	50.015	-0.460	19.181
N	48.480	2.915	17.801
C	49.261	3.804	16.958
C	48.546	5.105	16.650
O	47.756	5.589	17.446
H	47.717	3.206	18.407
H	50.146	4.009	17.415
H	49.484	3.331	16.086
N	48.874	5.673	15.484
C	48.353	6.972	15.107
C	47.919	6.935	13.641
O	48.444	6.204	12.786
C	49.339	8.133	15.347
C	49.787	8.291	16.787
O	50.454	7.996	14.466
H	49.516	5.111	14.896
H	47.547	7.174	15.665
H	48.867	9.011	15.134
H	49.761	9.259	17.050
H	49.179	7.772	17.394
H	50.724	7.949	16.894
H	50.400	8.671	13.721
N	46.934	7.767	13.363
C	46.378	7.884	12.025
C	46.301	9.360	11.659
O	45.825	10.189	12.434
C	44.967	7.287	11.945
C	44.518	6.967	10.541
C	44.021	7.943	9.689
C	44.616	5.678	10.066
C	43.616	7.636	8.395
C	44.251	5.354	8.776
C	43.734	6.334	7.953
O	43.416	6.059	6.676
H	46.592	8.327	14.174
H	46.997	7.435	11.382
H	44.956	6.445	12.479
H	44.329	7.946	12.336
H	43.944	8.967	10.036
H	44.963	4.954	10.672
H	43.228	8.406	7.741
H	44.359	4.419	8.438
H	43.708	6.812	6.080
N	46.751	9.668	10.449
C	46.693	11.025	9.937
C	45.981	10.998	8.597
O	46.452	10.366	7.659
C	48.090	11.602	9.781

C	48.073	12.985	9.174	N	39.076	15.540	4.392	C	47.503	16.055	12.419	H	44.674	11.409	16.696	C	42.960	1.288	20.123
C	49.412	13.707	9.205	C	38.009	15.797	5.376	C	47.080	14.610	12.768	H	43.506	10.032	14.288	C	41.485	1.489	20.315
O	49.449	14.842	8.697	C	37.503	17.216	5.320	O	46.272	14.000	12.060	H	42.659	10.198	15.861	O	43.335	0.101	20.818
O	50.399	13.148	9.742	O	36.367	17.437	4.813	C	47.849	16.906	13.634	H	44.143	12.187	13.003	H	42.292	2.750	17.587
H	47.140	8.867	9.912	H	38.959	15.885	3.415	O	49.075	16.488	14.218	H	41.616	12.223	16.453	H	44.338	0.921	18.648
H	46.192	11.608	10.572	H	37.163	15.116	5.164	H	46.561	16.925	10.623	H	43.321	14.393	12.416	H	43.461	2.073	20.539
H	48.489	11.670	10.672	H	38.340	15.583	6.415	H	48.331	15.995	11.861	H	40.852	14.425	15.810	H	41.314	2.110	21.088
H	48.593	11.016	9.180	N	38.223	18.271	5.810	H	47.936	17.857	13.351	H	40.828	16.122	14.307	H	41.075	1.888	19.487
H	47.804	12.907	8.223	C	39.627	18.193	6.260	H	47.124	16.815	14.311	N	44.755	9.129	17.747	H	41.033	0.610	20.504
H	47.428	13.543	9.679	C	39.722	17.940	7.760	H	48.902	16.141	15.138	C	44.974	7.824	18.346	H	44.083	-0.369	20.336
N	44.793	11.750	8.517	O	39.156	18.736	8.559	N	47.670	14.053	13.826	C	44.115	6.785	17.621	N	43.070	-1.151	17.937
C	44.005	11.700	7.328	C	40.362	19.491	5.848	C	47.549	12.633	14.147	O	42.969	7.072	17.320	C	42.339	-2.259	17.355
C	44.487	12.730	6.389	C	41.853	19.357	5.895	C	46.443	12.378	15.171	C	44.640	7.838	19.845	C	42.908	-3.609	17.742
O	44.425	13.942	6.731	H	37.724	19.186	5.870	O	46.229	13.154	16.110	C	44.763	6.437	20.420	O	43.613	-3.712	18.749
C	42.536	11.877	7.677	H	40.136	17.354	5.744	C	48.873	12.129	14.726	C	45.537	8.819	20.616	H	43.972	-1.251	18.440
O	41.756	11.621	6.550	H	40.092	19.755	4.805	C	49.999	12.061	13.717	H	44.300	9.922	18.239	H	41.366	-2.211	17.646
H	44.685	12.566	9.166	H	40.055	20.326	6.512	C	51.325	11.787	14.415	H	45.933	7.571	18.216	H	42.346	-2.172	16.342
H	44.207	10.739	6.857	N	42.608	20.366	5.670	N	52.372	11.637	13.427	H	43.689	8.110	19.942	N	42.556	-4.613	16.929
H	42.263	11.148	8.465	O	42.382	18.248	6.107	C	53.629	11.278	13.694	H	44.008	5.871	20.088	C	42.869	-6.013	17.174
H	42.362	12.904	8.081	H	42.222	21.291	5.405	N	54.438	10.941	12.700	H	45.632	6.032	20.134	C	43.241	-6.673	15.861
H	40.999	12.259	6.595	H	43.638	20.191	5.657	N	54.042	11.188	14.951	H	44.731	6.480	21.419	O	42.712	-6.287	14.822
N	44.631	12.408	5.107	N	40.445	16.813	8.267	H	48.227	14.722	14.407	H	45.376	8.704	21.593	C	41.660	-6.730	17.784
C	44.898	13.418	4.081	C	40.850	16.700	9.680	H	47.326	12.127	13.303	H	46.491	8.621	20.401	C	41.144	-6.133	19.079
C	43.620	14.105	3.588	C	42.150	17.466	9.931	H	49.146	12.747	15.458	H	45.310	9.750	20.339	C	42.040	-6.214	20.325
O	43.715	15.049	2.816	O	42.434	18.444	9.186	H	48.724	11.214	15.089	N	44.695	5.610	17.364	N	42.267	-7.599	20.690
C	45.653	12.787	2.929	C	40.962	15.207	10.025	H	49.831	11.323	13.074	C	43.961	4.448	16.906	C	43.223	-8.050	21.499
H	44.539	11.394	4.898	H	41.012	16.271	7.573	H	50.076	12.929	13.241	C	44.208	3.277	17.849	N	44.023	-7.218	22.133
H	45.508	14.124	4.473	H	40.067	17.141	10.334	H	51.525	12.556	15.003	O	45.268	3.171	18.452	N	43.366	-9.348	21.677
H	46.123	13.498	2.404	H	41.876	14.770	9.570	H	51.229	10.951	14.933	C	44.336	4.052	15.477	H	42.021	-4.291	16.078
H	46.328	12.138	3.283	H	40.992	15.054	11.123	H	52.140	11.817	12.457	C	45.701	3.404	15.250	H	43.650	-6.067	17.797
H	45.013	12.304	2.330	H	40.076	14.660	9.641	H	54.140	10.967	11.752	C	45.691	1.865	15.362	H	40.918	-6.709	17.114
N	42.373	13.749	4.041	N	42.951	17.202	11.012	H	55.384	10.651	12.909	C	46.209	3.811	13.879	H	41.920	-7.679	17.961
C	41.117	14.375	3.561	C	44.261	17.856	11.202	H	53.408	11.411	15.697	H	45.732	5.607	17.525	H	40.927	-5.142	18.930
C	40.175	14.772	4.673	C	45.207	17.032	12.088	H	54.975	10.903	15.156	H	42.980	4.658	16.915	H	40.253	-6.577	19.323
O	40.365	14.377	5.852	O	44.870	16.749	13.267	N	45.791	11.220	15.033	H	43.642	3.405	15.148	H	42.899	-5.777	20.105
C	40.384	13.447	2.565	C	44.073	19.266	11.786	C	44.735	10.772	15.933	H	44.302	4.880	14.912	H	41.574	-5.742	21.059
C	41.180	13.310	1.274	C	45.355	20.094	11.646	C	45.135	9.401	16.491	H	46.358	3.771	15.927	H	41.638	-8.298	20.292
C	40.070	12.068	3.151	C	45.192	21.525	12.135	O	45.743	8.586	15.790	H	45.067	1.495	14.679	H	43.917	-6.228	22.036
H	42.308	12.946	4.692	O	46.244	22.189	12.269	C	43.403	10.677	15.189	H	46.613	1.523	15.202	H	44.754	-7.576	22.727
H	41.354	15.328	3.040	O	44.095	21.975	12.570	C	42.911	12.040	14.789	H	45.387	1.609	16.276	H	42.746	-9.993	21.222
H	39.420	13.924	2.289	H	42.609	16.555	11.748	C	43.402	12.673	13.630	H	46.295	4.808	13.834	H	44.094	-9.701	22.269
H	41.542	14.210	1.028	H	44.726	17.976	10.198	C	42.003	12.686	15.550	H	47.104	3.394	13.714	N	44.104	-7.704	15.920
H	41.941	12.668	1.386	H	43.254	19.783	11.246	C	42.941	13.897	13.298	H	45.566	3.502	13.176	C	44.380	-8.573	14.793
H	40.580	12.985	0.541	H	43.789	19.191	12.857	C	41.564	13.910	15.187	N	43.186	2.427	17.987	C	44.472	-10.013	15.326
H	39.542	11.446	2.399	H	46.059	19.669	12.208	C	42.016	14.510	14.063	C	43.371	1.154	18.659	O	44.679	-10.219	16.533
H	40.996	11.526	3.436	H	45.655	20.140	10.698	O	41.520	15.749	13.667	C	42.614	0.105	17.860	C	45.669	-8.157	14.062
H	39.417	12.164	4.040	N	46.461	16.693	11.641	H	46.111	10.652	14.206	O	41.610	0.442	17.224	C	46.946	-8.517	14.790

C	47.359	-7.840	15.931
C	47.765	-9.531	14.299
C	48.509	-8.213	16.612
C	48.951	-9.869	14.926
C	49.314	-9.224	16.093
O	50.488	-9.591	16.666
H	44.556	-7.816	16.859
H	43.616	-8.533	14.158
H	45.678	-8.607	13.171
H	45.648	-7.166	13.939
H	46.818	-7.067	16.270
H	47.486	-10.026	13.474
H	48.760	-7.763	17.468
H	49.543	-10.576	14.537
H	50.553	-9.196	17.584
N	44.303	-10.946	14.413
C	44.489	-12.380	14.63
C	45.985	-12.694	14.714
O	46.681	-12.738	13.692
C	43.819	-13.170	13.507
C	43.860	-14.692	13.643
O	44.353	-15.177	14.678
O	43.446	-15.356	12.661
H	44.008	-10.563	13.465
H	44.094	-12.600	15.522
H	42.855	-12.886	13.460
H	44.267	-12.920	12.642
N	46.443	-12.928	15.953
C	47.855	-13.174	16.245
C	48.274	-14.629	15.987
O	49.450	-14.939	16.070
C	48.191	-12.728	17.651
O	47.310	-13.283	18.606
H	45.701	-12.919	16.689
H	48.408	-12.584	15.635
H	49.125	-13.013	17.871
H	48.131	-11.729	17.700
H	46.571	-13.768	18.139
N	47.341	-15.510	15.615
C	47.674	-16.877	15.248
C	46.910	-17.280	13.987
O	46.062	-18.164	13.999
C	47.344	-17.783	16.400
H	46.371	-15.133	15.612
H	48.657	-16.935	15.056
H	48.085	-18.444	16.541
H	47.225	-17.245	17.238
H	46.495	-18.283	16.212
N	47.182	-16.656	12.837
C	46.347	-16.885	11.649
C	46.567	-18.242	10.962
O	47.527	-18.944	11.266
C	46.779	-15.741	10.729
C	48.241	-15.524	11.104
C	48.319	-15.760	12.603
H	45.359	-16.765	11.881
H	46.704	-16.001	9.772
H	46.252	-14.917	10.906
H	48.821	-16.172	10.609
H	48.517	-14.590	10.874
H	49.177	-16.199	12.851
H	48.211	-14.907	13.106
N	45.680	-18.572	10.009
C	45.725	-19.819	9.259
C	46.976	-19.865	8.387
O	47.517	-18.837	7.972
C	44.469	-19.958	8.427
H	44.943	-17.838	9.854
H	45.758	-20.588	9.905
H	44.673	-20.464	7.587
H	43.771	-20.453	8.947
H	44.119	-19.051	8.187
N	47.419	-21.086	8.086
C	48.684	-21.300	7.398
C	48.452	-21.741	5.948
O	49.325	-22.375	5.357
C	49.502	-22.331	8.191
C	49.987	-21.832	9.538
O	48.611	-23.438	8.347
H	46.789	-21.859	8.383
H	49.194	-20.433	7.382
H	50.282	-22.624	7.637
H	49.542	-20.960	9.761
H	49.767	-22.502	10.251
H	50.981	-21.695	9.515
H	47.802	-23.324	7.759
N	47.303	-21.368	5.360
C	46.861	-21.858	4.059
C	46.978	-20.781	2.967
O	46.443	-20.941	1.867
C	45.437	-22.407	4.173
C	44.406	-21.355	4.560
O	44.807	-20.319	5.157
O	43.219	-21.551	4.225
H	46.745	-20.683	5.930
H	47.481	-22.609	3.792
H	45.182	-22.790	3.289
H	45.436	-23.122	4.867
N	47.700	-19.685	3.251
C	47.831	-18.575	2.315
C	46.742	-17.510	2.507
O	46.689	-16.558	1.742
H	48.151	-19.703	4.189
H	48.738	-18.140	2.431
H	47.782	-18.925	1.366
N	45.878	-17.695	3.513
C	44.876	-16.723	3.941
C	45.526	-15.471	4.516
O	46.602	-15.523	5.092
C	43.938	-17.316	4.966
O	42.944	-18.093	4.327
H	45.988	-18.637	3.986
H	44.340	-16.450	3.131
H	44.454	-17.899	5.585
H	43.496	-16.581	5.470
H	43.176	-19.062	4.404
N	44.828	-14.334	4.424
C	45.295	-13.137	5.081
C	45.004	-13.164	6.579
O	44.335	-14.066	7.077
H	43.965	-14.397	3.864
H	46.292	-13.036	4.943
H	44.847	-12.326	4.675
N	45.539	-12.150	7.268
C	45.388	-11.968	8.702
C	44.297	-10.930	8.956
O	44.499	-9.770	8.571
C	46.722	-11.549	9.331
C	46.624	-11.325	10.816
O	47.614	-12.619	8.965
H	46.091	-11.482	6.665
H	45.098	-12.833	9.109
H	47.042	-10.711	8.895
H	45.921	-10.635	11.019
H	46.378	-12.181	11.282
H	47.505	-11.003	11.179
H	48.475	-12.542	9.476
N	43.222	-11.378	9.603
C	42.056	-10.544	9.902
C	42.403	-9.527	10.979
O	43.156	-9.813	11.906
C	40.873	-11.381	10.291
H	43.281	-12.387	9.883
H	41.789	-10.053	9.068
H	40.972	-11.700	11.237
H	40.030	-10.842	10.218
H	40.801	-12.178	9.686
N	41.949	-8.278	10.772
C	42.239	-7.220	11.719
C	41.218	-6.097	11.562
O	40.455	-6.030	10.593
C	43.683	-6.719	11.534
C	44.114	-6.243	10.144
C	43.345	-5.010	9.715
C	45.609	-5.953	10.142
H	41.398	-8.157	9.909
H	42.164	-7.585	12.649
H	43.818	-5.951	12.167
H	44.298	-7.468	11.797
H	43.954	-6.984	9.485
H	43.508	-4.267	10.366
H	43.648	-4.723	8.805
H	42.365	-5.216	9.687
H	46.111	-6.786	10.378
H	45.888	-5.642	9.233
H	45.812	-5.240	10.814
N	41.232	-5.210	12.538
C	40.458	-3.988	12.424
C	40.962	-2.969	13.418
O	41.708	-3.317	14.326
H	41.815	-5.451	13.353
H	40.543	-3.621	11.493
H	39.490	-4.185	12.605
N	40.591	-1.706	13.206
C	40.967	-0.628	14.109
C	39.965	0.497	13.996
O	39.204	0.584	13.020
C	42.417	-0.178	13.888
C	42.670	0.656	12.659
C	42.581	2.002	12.492
C	43.037	0.118	11.379
C	43.143	1.193	10.490
C	43.244	-1.186	10.913
N	42.860	2.330	11.190
C	43.475	1.013	9.145
C	43.618	-1.353	9.602
C	43.692	-0.263	8.732
H	40.017	-1.563	12.349
H	40.925	-0.985	15.045
H	42.709	0.367	14.676
H	42.999	-0.990	13.815
H	42.347	2.658	13.208
H	42.857	3.259	10.812
H	43.124	-1.969	11.515
H	43.543	1.802	8.532
H	43.839	-2.266	9.268
H	43.916	-0.443	7.764
N	39.957	1.352	15.024
C	39.129	2.527	15.114
C	39.938	3.805	15.359
O	40.902	3.805	16.137
C	38.157	2.388	16.297
C	37.178	3.536	16.418
O	37.431	1.166	16.186
H	40.640	1.074	15.791
H	38.625	2.644	14.240
H	38.697	2.363	17.165
H	37.668	4.385	16.636
H	36.684	3.659	15.553
H	36.515	3.347	17.147
H	36.710	1.119	16.887
N	39.474	4.886	14.712
C	39.829	6.270	14.987
C	38.653	7.054	15.466
O	37.757	7.372	14.658
C	40.583	6.940	13.817
C	40.887	8.409	14.125
C	41.910	6.200	13.554
H	38.776	4.642	13.954
H	40.470	6.237	15.802
H	39.955	6.907	12.901
H	41.521	8.851	13.328
H	39.952	9.006	14.158
H	41.409	8.488	15.099
H	42.415	6.079	14.411
H	41.723	5.303	13.149
H	42.460	6.744	12.918
N	38.605	7.567	16.799
C	37.791	8.754	17.078
C	38.499	9.998	16.577
O	39.674	10.245	16.968
C	37.495	8.846	18.572
H	39.438	7.395	17.408
H	36.806	8.660	16.568
H	36.885	9.753	18.779

H	36.902	7.968	18.902
H	38.434	8.908	19.162
N	37.844	10.904	15.791
C	38.455	12.141	15.272
C	38.318	13.294	16.258
O	38.412	14.477	15.829
C	37.831	12.510	13.910
C	37.946	11.389	12.915
C	36.968	10.539	12.579
C	39.050	11.033	12.161
C	38.660	9.971	11.397
C	40.349	11.535	12.084
N	37.379	9.665	11.631
C	39.541	9.334	10.532
C	41.216	10.919	11.242
C	40.807	9.801	10.463
H	36.864	10.690	15.532
H	39.534	11.973	15.099
H	36.759	12.767	14.058
H	38.331	13.409	13.486
H	35.982	10.524	13.018
H	36.824	8.917	11.165
H	40.652	12.377	12.689
H	39.217	8.483	9.950
H	42.239	11.272	11.175
H	41.516	9.310	9.809
N	38.433	13.066	17.673
C	38.752	14.118	18.654
C	40.217	14.066	19.015
O	40.667	13.098	19.684
C	37.862	13.942	19.895
C	38.179	14.950	21.015
C	37.228	14.722	22.189
C	37.544	15.580	23.426
N	38.802	15.204	24.098
H	38.358	12.090	18.025
H	38.516	15.113	18.221
H	36.799	14.058	19.585
H	37.981	12.907	20.291
H	39.227	14.818	21.354
H	38.063	15.987	20.635
H	36.207	14.977	21.836
H	37.220	13.648	22.466
H	37.549	16.659	23.136
H	36.702	15.435	24.139
H	39.287	14.405	23.629
H	39.441	16.033	24.151
H	38.595	14.896	25.080
N	41.037	15.097	18.677
C	42.404	15.266	19.200
C	42.602	16.692	19.716
O	41.588	17.398	19.963
C	43.407	14.827	18.106
C	43.493	15.771	16.950
H	40.640	15.850	18.072
H	42.561	14.604	20.082
H	44.414	14.678	18.543
H	43.104	13.839	17.715
N	44.683	16.072	16.416
O	42.511	16.479	16.634
H	45.468	15.401	16.507
H	44.667	16.698	15.583
N	43.850	17.224	19.901
C	44.049	18.608	20.402
C	43.692	19.680	19.361
O	43.628	20.864	19.750
C	45.467	18.797	20.932
C	45.703	17.996	22.188
H	44.659	16.602	19.705
H	43.374	18.735	21.153
H	46.106	18.495	20.234
H	45.600	19.760	21.136
N	46.902	17.451	22.323
O	44.792	17.833	22.999
H	47.598	17.586	21.615
H	47.118	16.903	23.133
N	43.443	19.407	18.041
C	43.410	20.455	16.991
C	42.002	20.648	16.475
O	41.414	21.754	16.606
C	44.343	20.089	15.807
C	45.796	20.061	16.189
C	46.270	19.113	17.085
C	46.686	21.006	15.701
C	47.599	19.075	17.461
C	48.027	20.975	16.057
C	48.472	20.018	16.956
O	49.773	19.941	17.353
H	43.347	18.419	17.751
H	43.745	21.438	17.396
H	44.082	19.096	15.386
H	44.188	20.830	14.990
H	45.631	18.438	17.471
H	46.359	21.719	15.083
H	47.927	18.370	18.092
H	48.667	21.636	15.670
H	50.267	19.304	16.760
N	41.350	19.586	15.769
C	40.302	19.813	14.760
C	39.173	18.847	15.003
O	38.980	17.887	14.212
C	40.955	19.676	13.366
C	40.032	19.986	12.173
C	39.925	21.484	11.834
N	41.216	22.068	11.436
C	41.824	21.943	10.241
N	41.321	21.190	9.275
N	42.942	22.605	9.994
H	41.818	18.653	15.792
H	39.884	20.838	14.859
H	41.839	20.346	13.328
H	41.340	18.637	13.253
H	40.427	19.437	11.297
H	39.012	19.589	12.352
H	39.170	21.634	11.031
H	39.537	22.027	12.725
H	41.775	22.538	12.184
H	40.488	20.602	9.435
H	41.954	20.871	8.518
H	43.388	23.175	10.745
H	43.493	22.436	9.137
N	38.455	18.891	16.239
C	37.441	17.874	16.554
C	36.307	17.927	15.548
O	35.607	18.970	15.448
C	36.921	18.034	18.002
C	36.025	16.904	18.412
H	38.612	19.714	16.862
H	37.928	16.878	16.479
H	37.780	18.057	18.703
H	36.356	18.985	18.099
N	35.474	16.873	19.635
O	35.808	15.947	17.635
H	35.536	17.674	20.288
H	34.886	16.040	19.876
N	36.061	16.822	14.679
C	35.052	16.846	13.619
C	33.957	15.851	13.934
O	33.818	14.823	13.221
C	35.751	16.596	12.279
H	36.646	15.966	14.807
H	34.564	17.838	13.557
H	36.179	15.571	12.242
H	35.032	16.730	11.449
H	36.577	17.328	12.144
N	33.088	16.075	15.055
C	32.510	15.016	15.920
C	32.164	13.742	15.161
O	31.112	13.705	14.471
C	31.235	15.550	16.619
C	31.483	16.771	17.462
H	33.188	17.024	15.479
H	33.253	14.752	16.705
H	30.470	15.794	15.848
H	30.797	14.761	17.272
C	31.058	17.997	17.196
N	32.085	16.770	18.664
C	31.976	18.044	19.093
N	31.373	18.794	18.232
H	32.484	15.955	19.187
H	30.505	18.314	16.322
H	32.301	18.392	20.070
N	33.080	12.641	15.085
C	32.811	11.422	14.269
C	33.723	10.261	14.617
O	34.625	10.422	15.477
C	32.880	11.711	12.765
O	34.188	11.908	12.303
H	33.883	12.673	15.753
H	31.774	11.100	14.457
H	32.433	10.861	12.213
H	32.263	12.588	12.526
H	34.563	12.658	12.837
N	33.663	9.018	13.904
C	34.595	7.914	14.186
C	34.652	6.912	13.037
O	33.586	6.370	12.644
C	34.169	7.212	15.480
H	32.773	8.792	13.414
H	35.605	8.338	14.336
H	33.139	6.812	15.371
H	34.862	6.376	15.714
H	34.180	7.924	16.333
N	35.840	6.558	12.448
C	35.964	5.474	11.450
C	36.358	4.154	12.080
O	37.327	4.056	12.896
C	36.954	5.836	10.330
C	37.043	4.746	9.248
O	36.566	7.049	9.745
H	36.704	7.064	12.719
H	34.984	5.340	10.955
H	37.953	5.988	10.780
H	37.496	3.808	9.640
H	36.034	4.516	8.847
H	37.682	5.100	8.415
H	35.849	6.845	9.090
N	35.792	3.018	11.598
C	36.395	1.688	11.772
C	36.767	1.097	10.416
O	35.998	1.178	9.468
C	35.460	0.716	12.505
C	35.046	1.301	13.830
O	34.306	0.461	11.679
H	35.029	3.121	10.905
H	37.232	1.776	12.313
H	35.944	-0.168	12.648
H	33.955	1.412	13.898
H	35.315	0.688	14.580
H	35.487	2.193	13.969
H	34.173	1.139	10.979
N	37.962	0.529	10.390
C	38.476	-0.258	9.302
C	38.453	-1.718	9.706
O	39.006	-2.077	10.733
C	39.898	0.170	8.926
C	40.066	1.552	8.366
C	40.303	1.819	7.046
C	40.191	2.818	9.049
C	40.433	3.799	8.063
C	40.032	3.261	10.368
N	40.498	3.158	6.870
C	40.577	5.158	8.366
C	40.202	4.592	10.678
C	40.497	5.533	9.662
H	38.527	0.713	11.275
H	37.861	-0.152	8.519
H	40.478	0.113	9.746
H	40.256	-0.472	8.238
H	40.329	1.133	6.321
H	40.666	3.606	5.979
H	39.791	2.604	11.094
H	40.815	5.871	7.593
H	40.063	4.919	11.694
H	40.654	6.570	9.928

N	37.946	-2.551	8.792
C	37.927	-3.999	8.925
C	38.548	-4.585	7.657
O	38.188	-4.212	6.539
C	36.495	-4.439	9.121
O	36.416	-5.835	9.273
H	37.552	-2.053	7.947
H	38.505	-4.253	9.692
H	36.128	-4.002	9.939
H	35.959	-4.168	8.324
H	35.552	-6.079	9.715
N	39.552	-5.453	7.820
C	40.261	-5.989	6.668
C	41.249	-7.090	7.021
O	41.120	-7.798	8.030
H	39.766	-5.696	8.798
H	39.586	-6.351	5.997
H	40.758	-5.234	6.199
N	42.214	-7.260	6.122
C	43.178	-8.320	6.300
C	44.521	-7.869	5.737
O	44.614	-7.132	4.754
C	42.623	-9.644	5.754
C	42.310	-9.702	4.276
C	41.746	-11.042	3.855
H	42.206	-6.601	5.334
H	43.299	-8.479	7.281
H	43.294	-10.377	5.964
H	41.773	-9.864	6.265
H	41.639	-8.993	4.059
H	43.150	-9.534	3.760
N	40.735	-11.006	3.000
O	42.220	-12.103	4.277
H	40.308	-11.869	2.657
H	40.384	-10.104	2.686
N	45.557	-8.288	6.462
C	46.941	-8.134	6.065
C	47.325	-9.311	5.156
O	47.067	-10.462	5.486
C	47.779	-8.063	7.346
C	49.250	-8.284	7.128
C	50.050	-7.236	6.699
C	49.841	-9.523	7.343
C	51.407	-7.415	6.489
C	51.203	-9.714	7.155
C	51.985	-8.651	6.726
O	53.341	-8.741	6.504
H	45.272	-8.751	7.369
H	47.034	-7.295	5.532
H	47.651	-7.159	7.750
H	47.444	-8.763	7.974
H	49.643	-6.335	6.537
H	49.275	-10.292	7.640
H	51.972	-6.650	6.167
H	51.616	-10.605	7.326
H	53.789	-7.934	6.884
N	47.941	-9.001	4.011
C	48.430	-9.982	3.060
C	49.932	-9.726	2.897
O	50.336	-8.685	2.362
C	47.669	-9.877	1.725
C	48.098	-10.972	0.769
C	46.152	-9.892	1.914
H	48.041	-7.961	3.859
H	48.318	-10.886	3.449
H	47.915	-9.007	1.305
H	48.690	-11.623	1.246
H	47.291	-11.452	0.422
H	48.599	-10.572	0.001
H	45.937	-10.296	2.802
H	45.809	-8.955	1.876
H	45.737	-10.435	1.186
N	50.746	-10.626	3.458
C	52.194	-10.448	3.500
C	52.849	-10.766	2.157
O	52.166	-10.905	1.148
H	50.259	-11.456	3.857
H	52.408	-9.494	3.755
H	52.586	-11.051	4.210
N	54.182	-10.889	2.164
C	54.929	-11.373	1.014
C	55.445	-10.230	0.144
O	55.742	-9.135	0.638
H	54.631	-10.608	3.064
H	55.714	-11.928	1.330
H	54.337	-11.973	0.454
N	55.582	-10.518	-1.159
C	55.875	-9.496	-2.151
C	54.755	-8.455	-2.110
O	53.583	-8.827	-2.196
C	56.009	-10.131	-3.523
H	55.460	-11.526	-1.388
H	56.733	-9.047	-1.916
H	55.257	-10.774	-3.671
H	55.980	-9.421	-4.227
H	56.879	-10.621	-3.583
N	55.119	-7.168	-1.941
C	54.151	-6.074	-1.961
C	53.085	-6.368	-0.898
O	52.007	-6.873	-1.206
C	53.530	-5.897	-3.358
C	54.541	-5.767	-4.499
C	54.621	-4.401	-5.159
O	53.579	-3.930	-5.635
O	55.726	-3.821	-5.218
H	56.140	-7.031	-1.797
H	54.603	-5.215	-1.748
H	52.977	-6.692	-3.541
H	52.992	-5.072	-3.343
H	55.442	-5.975	-4.133
H	54.294	-6.424	-5.203
N	53.471	-6.157	0.363
C	52.592	-6.360	1.501
C	51.505	-5.283	1.440
O	51.792	-4.122	1.120
C	53.394	-6.287	2.779
H	54.463	-5.833	0.455
H	52.157	-7.251	1.427
H	54.190	-6.892	2.716
H	53.707	-5.348	2.931
H	52.826	-6.572	3.553
N	50.276	-5.704	1.764
C	49.115	-4.837	1.696
C	48.247	-5.070	2.925
O	48.247	-6.154	3.509
C	48.319	-5.123	0.421
C	48.120	-3.768	-0.453
C	47.982	-4.029	-1.940
N	49.083	-3.461	-2.703
C	50.280	-4.021	-2.829
N	51.078	-4.119	-1.781
N	50.673	-4.478	-4.005
H	50.234	-6.702	2.066
H	49.417	-3.883	1.694
H	48.812	-5.734	-0.153
H	47.422	-5.423	0.645
H	47.299	-3.325	-0.110
H	48.918	-3.200	-0.283
H	47.957	-5.017	-2.081
H	47.122	-3.623	-2.247
H	48.927	-2.579	-3.170
H	50.798	-3.799	-0.880
H	51.996	-4.529	-1.890
H	50.058	-4.419	-4.796
H	51.580	-4.884	-4.112
N	47.519	-4.027	3.314
C	46.357	-4.213	4.168
C	45.146	-3.739	3.365
O	45.086	-2.582	2.966
C	46.521	-3.487	5.505
C	47.766	-3.956	6.275
C	45.246	-3.650	6.339
C	47.967	-3.203	7.554
H	47.842	-3.111	2.968
H	46.248	-5.189	4.290
H	46.630	-2.509	5.319
H	47.661	-4.925	6.471
H	48.557	-3.823	5.686
H	45.326	-3.107	7.171
H	44.463	-3.336	5.807
H	45.128	-4.611	6.575
H	47.173	-3.330	8.151
H	48.786	-3.540	8.021
H	48.078	-2.227	7.359
N	44.216	-4.654	3.075
C	43.034	-4.322	2.298
C	41.838	-4.199	3.230
O	41.632	-5.092	4.047
C	42.709	-5.410	1.277
C	43.765	-5.527	0.201
H	44.414	-5.603	3.449
H	43.185	-3.419	1.894
H	42.643	-6.281	1.756
H	41.838	-5.188	0.847
N	44.128	-6.755	-0.100
O	44.269	-4.527	-0.317
H	43.721	-7.546	0.355
H	44.834	-6.912	-0.806
N	41.157	-3.047	3.190
C	40.134	-2.774	4.193
C	38.860	-2.253	3.545
O	38.877	-1.667	2.451
C	40.525	-1.723	5.256
C	41.817	-2.034	5.968
O	40.620	-0.438	4.658
H	41.410	-2.411	2.429
H	39.918	-3.634	4.672
H	39.793	-1.696	5.969
H	42.151	-1.219	6.452
H	41.676	-2.771	6.636
H	42.516	-2.326	5.307
H	41.100	-0.492	3.773
N	37.768	-2.427	4.317
C	36.548	-1.676	4.158
C	36.349	-0.869	5.425
O	36.854	-1.258	6.480
C	35.424	-2.646	3.851
C	35.630	-3.235	2.441
C	34.454	-3.922	1.794
H	37.899	-3.171	5.051
H	36.666	-1.037	3.404
H	35.443	-3.390	4.491
H	34.565	-2.170	3.853
H	35.939	-2.477	1.833
H	36.406	-3.895	2.494
N	34.511	-5.243	1.803
O	33.546	-3.292	1.240
H	33.741	-5.799	1.393
H	35.311	-5.709	2.211
N	35.674	0.275	5.302
C	35.517	1.127	6.476
C	34.109	1.694	6.552
O	33.381	1.808	5.553
C	36.603	2.222	6.531
C	36.709	3.111	5.328
C	37.703	3.065	4.396
C	35.876	4.222	4.970
C	36.404	4.782	3.786
C	34.758	4.804	5.568
N	37.505	4.045	3.444
C	35.825	5.882	3.168
C	34.186	5.869	4.953
C	34.718	6.407	3.748
H	35.305	0.483	4.377
H	35.660	0.554	7.291
H	36.419	2.811	7.321
H	37.494	1.779	6.653
H	38.465	2.421	4.396
H	38.075	4.190	2.637
H	34.359	4.411	6.487
H	36.232	6.287	2.252
H	33.300	6.318	5.381
H	34.221	7.249	3.282
N	33.625	2.132	7.757
C	32.338	2.799	8.017

C	32.590	4.097	8.787
O	33.199	4.022	9.882
C	31.470	1.878	8.913
C	30.946	0.570	8.287
C	30.319	-0.298	9.377
C	29.901	0.846	7.224
H	34.223	2.015	8.583
H	31.819	3.023	7.071
H	32.099	1.586	9.777
H	30.600	2.458	9.296
H	31.767	-0.016	7.840
H	29.540	0.271	9.926
H	29.855	-1.206	8.936
H	31.097	-0.627	10.098
H	30.341	1.435	6.398
H	29.526	-0.108	6.798
H	29.056	1.409	7.669
N	32.203	5.330	8.332
C	32.399	6.576	9.117
C	31.074	7.065	9.676
O	30.361	7.841	8.986
C	33.095	7.636	8.229
C	33.664	8.872	8.969
C	34.500	9.688	7.989
C	32.585	9.789	9.541
H	31.715	5.373	7.414
H	33.069	6.390	9.983
H	33.933	7.130	7.704
H	32.402	7.988	7.447
H	34.319	8.543	9.803
H	33.897	9.974	7.102
H	34.856	10.611	8.486
H	35.380	9.098	7.657
H	32.119	9.321	10.427
H	33.028	10.746	9.884
H	31.808	10.008	8.780
N	30.695	6.782	10.958
C	29.484	7.382	11.562
C	29.738	8.806	11.994
O	30.495	9.059	12.964
C	28.910	6.517	12.699
C	27.673	7.162	13.315
O	28.503	5.279	12.173
H	31.337	6.207	11.548
H	28.683	7.420	10.809
H	29.685	6.383	13.489
H	27.959	8.026	13.951
H	26.967	7.497	12.527
H	27.154	6.422	13.954
H	29.241	4.633	12.341
N	29.222	9.888	11.216
C	28.947	11.228	11.777
C	28.082	11.101	13.014
O	27.051	10.389	12.973
C	28.260	12.122	10.698
C	29.211	12.354	9.493
C	27.782	13.475	11.260
C	28.492	12.903	8.258
H	28.787	9.612	10.310
H	29.906	11.698	12.075
H	27.359	11.578	10.339
H	30.029	13.047	9.781
H	29.690	11.400	9.190
H	27.194	14.026	10.501
H	27.103	13.334	12.120
H	28.628	14.097	11.596
H	28.159	13.947	8.424
H	29.190	12.899	7.394
H	27.617	12.267	8.003
N	28.362	11.851	14.195
C	27.385	11.943	15.287
C	26.194	12.770	14.878
O	26.387	13.944	14.454
H	29.094	12.585	14.138
H	27.066	10.921	15.584
H	27.855	12.423	16.171
N	24.939	12.227	14.842
C	23.761	12.926	14.273
C	22.601	12.952	15.229
O	22.377	11.951	15.952
C	23.280	12.254	12.968
C	24.223	12.546	11.813
O	23.185	10.867	13.115
H	24.822	11.240	15.172
H	24.008	13.987	14.061
H	22.285	12.656	12.675
H	24.383	13.638	11.701
H	25.193	12.046	11.996
H	23.791	12.149	10.871
H	22.496	10.705	13.821
N	21.538	13.874	14.989
C	20.200	13.722	15.602
C	19.484	12.448	15.151
O	19.913	11.797	14.159
C	19.342	14.983	15.429
C	20.018	16.274	15.892
O	19.049	15.084	14.057
H	21.705	14.617	14.280
H	20.272	13.636	16.669
H	18.466	14.910	15.935
H	19.353	17.141	15.669
H	20.204	16.235	16.988
H	20.983	16.448	15.378
H	18.282	15.717	13.991
N	18.419	12.046	15.851
C	17.622	10.895	15.477
C	17.045	11.104	14.080
O	17.028	10.171	13.263
C	16.554	10.665	16.556
C	17.160	10.194	17.875
C	16.861	11.073	19.080
O	17.757	11.843	19.480
O	15.750	10.977	19.625
H	18.216	12.638	16.691
H	18.188	10.068	15.482
H	16.109	11.517	16.721
H	15.954	9.965	16.238
H	16.802	9.289	18.072
H	18.146	10.164	17.768
N	16.621	12.344	13.798
C	15.987	12.653	12.525
C	16.975	12.446	11.374
O	16.565	12.153	10.252
C	15.469	14.066	12.541
H	16.783	13.037	14.548
H	15.207	12.034	12.388
H	16.166	14.682	12.915
H	15.242	14.358	11.609
H	14.645	14.123	13.110
N	18.270	12.621	11.653
C	19.335	12.645	10.674
C	20.181	11.400	10.768
O	21.222	11.283	10.070
C	20.178	13.909	10.892
C	19.370	15.087	10.530
H	18.482	12.769	12.657
H	18.805	12.659	9.719
H	20.510	13.975	11.949
H	21.071	13.893	10.234
N	19.420	16.192	11.194
O	18.709	15.051	9.472
H	19.976	16.243	12.074
H	18.830	17.023	10.928
N	19.714	10.354	11.491
C	20.448	9.098	11.720
C	20.578	8.295	10.445
O	21.681	7.763	10.152
C	19.747	8.282	12.808
H	18.777	10.541	11.913
H	21.470	9.334	12.083
H	18.710	8.024	12.504
H	20.311	7.347	13.013
H	19.703	8.874	13.748
N	19.556	8.264	9.539
C	19.692	7.669	8.192
C	20.847	8.257	7.406
O	21.568	7.504	6.701
C	18.377	7.810	7.411
C	18.121	9.186	6.885
C	17.558	10.223	7.570
C	18.531	9.714	5.608
C	18.135	11.070	5.582
C	19.119	9.168	4.466
N	17.560	11.350	6.791
C	18.336	11.889	4.473
C	19.336	9.980	3.375
C	18.940	11.343	3.394
H	18.635	8.641	9.857
H	19.901	6.581	8.309
H	18.403	7.195	6.636
H	17.635	7.583	8.025
H	17.198	10.170	8.499
H	17.196	12.245	7.063
H	19.381	8.203	4.437
H	18.055	12.848	4.487
H	19.792	9.601	2.570
H	19.133	11.953	2.527
N	21.116	9.599	7.466
C	22.263	10.242	6.794
C	23.371	10.545	7.746
O	24.059	11.596	7.611
C	21.814	11.484	6.001
C	21.150	12.562	6.868
C	20.894	13.848	6.073
C	20.019	14.791	6.903
N	19.666	16.013	6.172
H	20.506	10.159	8.094
H	22.701	9.536	6.072
H	22.693	11.911	5.491
H	21.110	11.163	5.209
H	20.183	12.176	7.247
H	21.787	12.806	7.745
H	21.867	14.334	5.844
H	20.388	13.605	5.116
H	19.085	14.250	7.195
H	20.571	15.052	7.839
H	19.126	15.783	5.306
H	19.057	16.605	6.788
H	20.521	16.563	5.928
N	23.876	9.459	8.492
C	25.062	9.541	9.362
C	26.169	8.644	8.865
O	27.277	9.157	8.554
C	24.687	9.207	10.812
O	24.718	7.824	11.057
H	23.357	8.555	8.446
H	25.451	10.581	9.363
H	25.408	9.689	11.487
H	23.687	9.643	11.035
H	23.779	7.536	11.217
N	25.964	7.305	8.668
C	27.063	6.365	8.373
C	27.494	6.473	6.942
O	26.628	6.465	6.038
C	26.674	4.908	8.714
C	27.891	3.992	8.821
O	25.965	4.846	9.925
H	25.001	6.922	8.797
H	27.926	6.626	8.995
H	26.011	4.517	7.914
H	28.622	4.381	9.560
H	27.570	2.981	9.142
H	28.380	3.891	7.839
H	26.553	5.238	10.629
N	28.816	6.543	6.616
C	29.345	6.372	5.256
C	29.927	4.984	5.104
O	30.024	4.229	6.104
C	30.410	7.458	4.975
C	29.816	8.873	4.804
C	30.869	9.935	5.123
C	29.324	9.090	3.371
H	29.493	6.447	7.394
H	28.544	6.450	4.508
H	31.116	7.448	5.820

H	31.014	7.215	4.076
H	28.967	9.019	5.504
H	31.763	9.805	4.477
H	30.446	10.949	4.972
H	31.173	9.847	6.188
H	28.565	8.331	3.106
H	28.860	10.090	3.274
H	30.164	9.009	2.649
N	30.442	4.591	3.915
C	31.175	3.331	3.689
C	32.161	3.536	2.539
O	31.857	4.282	1.605
C	30.227	2.163	3.396
C	29.413	2.376	2.138
C	30.927	0.798	3.412
H	30.398	5.276	3.139
H	31.706	3.103	4.626
H	29.501	2.158	4.240
H	28.445	2.201	2.328
H	29.521	3.320	1.821
H	29.727	1.750	1.422
H	30.693	0.304	2.577
H	31.914	0.940	3.459
H	30.618	0.285	4.210
N	33.342	2.952	2.701
C	34.363	2.981	1.667
C	35.353	1.846	1.819
O	35.129	0.903	2.561
H	33.470	2.479	3.620
H	33.922	2.922	0.758
H	34.856	3.864	1.707
N	36.431	1.933	1.030
C	37.445	0.902	0.964
C	38.799	1.603	0.828
O	38.904	2.568	0.063
C	37.116	-0.119	-0.130
C	36.804	0.523	-1.407
H	36.470	2.816	0.467
H	37.446	0.392	1.816
H	37.906	-0.726	-0.246
H	36.328	-0.662	0.170
C	35.736	1.139	-1.946
N	37.836	0.662	-2.313
C	37.371	1.253	-3.415
N	36.120	1.599	-3.198
H	38.776	0.369	-2.171
H	34.837	1.252	-1.537
H	37.887	1.403	-4.250
N	39.744	1.218	1.687
C	41.107	1.719	1.686
C	42.066	0.538	1.503
O	41.916	-0.502	2.152
C	41.454	2.480	2.978
C	40.885	3.880	3.134
O	40.277	4.366	2.163
O	41.027	4.461	4.262
H	39.404	0.499	2.385
H	41.214	2.304	0.884
H	41.135	1.917	3.756
H	42.462	2.536	3.035
N	43.065	0.716	0.613
C	44.175	-0.221	0.492
C	45.477	0.447	0.931
O	45.832	1.510	0.446
C	44.418	-0.763	-0.932
C	45.453	-1.867	-0.944
O	43.202	-1.285	-1.449
H	42.966	1.573	0.032
H	44.003	-1.003	1.097
H	44.718	0.010	-1.526
H	45.001	-2.763	-0.910
H	46.004	-1.813	-1.781
H	46.060	-1.779	-0.149
H	42.785	-1.929	-0.796
N	46.138	-0.196	1.905
C	47.336	0.338	2.538
C	48.570	-0.395	2.038
O	48.593	-1.635	1.984
C	47.248	0.217	4.049
C	46.172	1.083	4.653
C	44.870	0.616	4.778
C	46.442	2.370	5.050
C	43.883	1.444	5.287
C	45.458	3.182	5.614
C	44.183	2.705	5.722
H	45.713	-1.116	2.167
H	47.424	1.294	2.241
H	47.040	-0.721	4.272
H	48.112	0.500	4.432
H	44.644	-0.310	4.505
H	47.370	2.745	4.936
H	42.928	1.097	5.330
H	45.704	4.109	5.931
H	43.459	3.280	6.123
N	49.604	0.380	1.705
C	50.904	-0.242	1.452
C	51.984	0.593	2.125
O	51.719	1.689	2.596
C	51.247	-0.437	-0.032
C	50.143	-1.072	-0.851
O	51.611	0.806	-0.622
H	49.417	1.379	1.647
H	50.905	-1.152	1.861
H	52.047	-1.073	-0.104
H	49.251	-0.886	-0.429
H	50.146	-0.697	-1.782
H	50.279	-2.065	-0.900
H	50.838	1.451	-0.596
N	53.214	0.061	2.160
C	54.304	0.746	2.827
C	54.929	1.822	1.926
O	55.660	2.669	2.415
C	55.260	-0.313	3.387
C	54.663	-1.091	4.549
C	55.662	-1.626	5.537
C	54.991	-2.212	6.757
N	55.813	-3.285	7.369
H	53.304	-0.851	1.682
H	53.954	1.197	3.660
H	55.477	-0.952	2.659
H	56.081	0.147	3.704
H	54.007	-0.486	5.038
H	54.122	-1.866	4.173
H	56.205	-2.342	5.099
H	56.263	-0.883	5.831
H	54.844	-1.484	7.433
H	54.100	-2.591	6.492
H	56.378	-2.898	8.099
H	55.213	-3.989	7.750
H	56.403	-3.692	6.672
N	54.572	1.870	0.647
C	55.040	2.919	-0.260
C	54.037	4.089	-0.295
O	52.904	3.889	-0.703
C	55.180	2.316	-1.674
C	55.711	3.329	-2.686
C	56.014	1.042	-1.706
H	53.934	1.102	0.345
H	55.909	3.275	0.091
H	54.265	2.050	-1.982
H	55.441	3.051	-3.607
H	55.330	4.230	-2.484
H	56.708	3.366	-2.627
H	55.833	0.551	-2.558
H	56.983	1.281	-1.654
H	55.767	0.467	-0.927
N	54.449	5.307	0.077
C	53.608	6.496	-0.043
C	53.416	6.846	-1.513
O	54.403	6.919	-2.244
C	54.261	7.686	0.667
C	53.444	8.971	0.724
C	54.169	10.084	1.481
C	53.524	11.447	1.327
N	52.051	11.377	1.491
H	55.420	5.331	0.459
H	52.712	6.303	0.363
H	54.471	7.407	1.612
H	55.130	7.888	0.198
H	53.263	9.285	-0.210
H	52.573	8.786	1.183
H	54.186	9.845	2.456
H	55.113	10.136	1.143
H	53.904	12.068	2.018
H	53.738	11.808	0.415
H	51.714	12.246	1.855
H	51.624	11.200	0.604
H	51.820	10.638	2.125
N	52.168	7.090	-1.990
C	51.890	7.182	-3.428
C	52.088	8.590	-4.018
O	52.571	9.446	-3.291
C	50.417	6.749	-3.480
C	49.833	7.344	-2.203
C	50.955	7.307	-1.180
O	51.843	9.027	-5.118
H	52.450	6.504	-3.944
H	49.961	7.128	-4.277
H	50.334	5.760	-3.469
H	49.532	8.284	-2.371
H	49.052	6.797	-1.898
H	51.015	8.171	-0.679
H	50.825	6.556	-0.529
S	38.208	7.982	7.286
O	42.350	7.884	5.288
N	40.384	9.303	5.294
N	40.127	7.009	4.747
C	36.847	13.326	9.463
C	37.963	12.665	8.658
C	37.463	11.413	7.925
C	38.576	10.791	7.071
C	38.099	9.547	6.260
C	38.983	9.135	5.069
C	37.924	6.982	5.934
C	38.772	7.535	4.699
C	41.085	8.067	5.124
H	40.488	9.557	6.256
H	40.144	6.334	5.485
H	37.305	13.978	10.222
H	36.283	12.571	10.029
H	38.777	12.359	9.357
H	38.408	13.389	7.936
H	37.138	10.661	8.677
H	36.605	11.693	7.274
H	39.411	10.537	7.763
H	38.911	11.559	6.344
H	37.128	9.879	5.958
H	38.671	9.785	4.278
H	38.316	6.016	6.173
H	36.891	7.092	5.677
H	38.295	7.302	3.770
C	28.039	18.439	10.376
N	31.094	17.283	10.917
O	36.143	14.618	7.502
C	27.644	17.169	9.681
N	34.779	14.609	9.338
C	28.135	18.591	11.825
O	30.863	21.408	8.818
C	27.815	17.559	12.866
O	32.366	20.378	7.465
C	28.363	19.970	12.108
N	31.490	19.246	9.253
C	28.443	20.555	13.480
C	28.427	20.699	10.859
C	28.519	22.187	10.736
C	28.176	19.755	9.797
C	28.003	20.106	8.352
C	32.352	18.143	9.097
C	32.084	17.078	9.997
C	33.419	17.936	8.214
C	32.791	15.839	9.951
C	34.219	16.772	8.249
C	33.954	15.728	9.124
C	32.269	14.777	10.711
C	30.723	16.299	11.734

C	31.232	15.008	11.586
C	35.917	14.217	8.629
C	31.538	20.430	8.521
C	32.632	21.581	6.624
C	31.349	22.010	5.909
C	33.225	22.699	7.482
C	33.666	21.057	5.624
Ir	30.126	19.114	10.824
H	28.179	16.314	10.104
H	27.851	17.205	8.612
H	26.573	16.983	9.825
H	34.687	14.205	10.264
H	28.534	17.568	13.690
H	27.753	16.547	12.462
H	26.833	17.780	13.309
H	28.983	21.502	13.497
H	28.914	19.868	14.184
H	27.423	20.748	13.845
H	28.911	22.487	9.767
H	27.526	22.632	10.881
H	29.174	22.601	11.507
H	26.976	20.460	8.201
H	28.687	20.905	8.066
H	28.167	19.245	7.704
H	33.680	18.705	7.507
H	35.068	16.699	7.583
H	32.682	13.780	10.602
H	30.016	16.533	12.513
H	30.807	14.221	12.186
H	31.585	22.799	5.188
H	30.910	21.171	5.362
H	30.616	22.402	6.616
H	33.550	23.519	6.834
H	34.100	22.341	8.034
H	32.494	23.091	8.190
H	33.935	21.855	4.926
H	33.265	20.217	5.053
H	34.576	20.736	6.139
N	31.307	19.895	12.184
C	31.764	19.405	13.368
O	31.224	18.425	13.903
C	32.818	20.244	14.091
H	33.222	19.632	14.900
H	32.270	21.064	14.571
C	33.926	20.810	13.185
H	34.669	20.035	12.984
H	34.461	21.596	13.733
C	33.381	21.349	11.858
H	33.959	20.990	11.001
H	32.330	20.790	11.736
C	33.077	22.839	11.715
C	32.236	23.432	12.859
C	34.393	23.643	11.541
H	32.502	22.957	10.784
C	31.955	24.928	12.644
H	32.769	23.308	13.813
H	31.293	22.877	12.954
C	34.114	25.140	11.331
H	35.015	23.512	12.438
H	34.968	23.241	10.698
C	33.257	25.722	12.464
H	31.382	25.324	13.490
H	31.324	25.054	11.753
H	35.062	25.683	11.253
H	33.592	25.280	10.374
H	33.034	26.776	12.269
H	33.828	25.694	13.403
N	5.895	-16.409	-4.446
C	6.072	-15.132	-3.679
C	5.712	-13.892	-4.493
O	5.793	-12.789	-3.964
C	7.514	-14.873	-3.249
C	8.108	-15.913	-2.322
O	7.335	-16.676	-1.661
O	9.339	-15.996	-2.319
H	5.569	-16.397	-5.399
H	5.431	-15.182	-2.900
H	8.078	-14.825	-4.079
H	7.542	-13.981	-2.789
N	5.345	-14.072	-5.767
C	4.885	-12.989	-6.622
C	3.762	-12.202	-5.939
O	3.828	-10.976	-5.871
C	4.443	-13.595	-7.957
C	4.312	-12.585	-9.060
C	3.729	-13.160	-10.341
O	3.587	-12.393	-11.295
O	3.389	-14.366	-10.364
H	5.420	-15.066	-6.092
H	5.647	-12.366	-6.827
H	5.126	-14.252	-8.222
H	3.563	-14.011	-7.815
H	3.705	-11.865	-8.762
H	5.208	-12.235	-9.281
N	2.751	-12.917	-5.408
C	1.622	-12.318	-4.708
C	2.053	-11.659	-3.399
O	1.514	-10.616	-2.996
C	0.602	-13.396	-4.424
H	2.855	-13.952	-5.547
H	1.209	-11.611	-5.300
H	-0.319	-13.049	-4.609
H	0.777	-14.191	-5.008
H	0.661	-13.675	-3.464
N	2.977	-12.322	-2.694
C	3.450	-11.867	-1.404
C	4.236	-10.561	-1.519
O	4.150	-9.691	-0.644
H	3.321	-13.193	-3.161
H	2.666	-11.723	-0.783
H	4.044	-12.571	-0.990
N	5.056	-10.439	-2.573
C	5.892	-9.238	-2.719
C	5.104	-8.074	-3.344
O	5.341	-6.896	-3.037
C	7.151	-9.571	-3.552
C	8.070	-10.525	-2.787
C	7.896	-8.319	-4.007
C	9.064	-11.290	-3.670
H	5.048	-11.219	-3.234
H	6.134	-8.950	-1.788
H	6.853	-10.028	-4.384
H	8.587	-10.001	-2.112
H	7.503	-11.193	-2.305
H	8.649	-8.581	-4.607
H	7.268	-7.721	-4.501
H	8.256	-7.839	-3.209
H	9.645	-10.634	-4.143
H	9.617	-11.882	-3.092
H	8.554	-11.834	-4.330
N	4.239	-8.375	-4.317
C	3.482	-7.337	-4.994
C	2.643	-6.541	-3.987
O	1.915	-7.103	-3.189
C	2.601	-7.942	-6.094
C	1.656	-6.931	-6.705
O	3.485	-8.438	-7.104
H	4.163	-9.383	-4.534
H	4.124	-6.701	-5.435
H	2.085	-8.722	-5.711
H	0.711	-7.267	-6.653
H	1.718	-6.059	-6.211
H	1.893	-6.776	-7.668
H	3.788	-9.370	-6.878
N	2.715	-5.217	-4.061
C	1.946	-4.372	-3.165
C	2.750	-3.184	-2.651
O	3.736	-2.770	-3.273
H	3.351	-4.853	-4.794
H	1.122	-4.030	-3.647
H	1.625	-4.921	-2.375
N	2.290	-2.648	-1.518
C	2.805	-1.419	-0.958
C	3.485	-1.770	0.354
O	2.930	-2.460	1.218
C	1.698	-0.367	-0.759
C	2.233	0.920	-0.174
O	1.064	-0.078	-2.008
H	1.518	-3.204	-1.072
H	3.488	-1.042	-1.575
H	0.993	-0.748	-0.124
H	3.228	0.857	-0.054
H	2.029	1.689	-0.787
H	1.809	1.096	0.719
H	1.735	-0.109	-2.757
N	4.712	-1.255	0.492
C	5.539	-1.486	1.655
C	5.963	-0.154	2.257
O	6.234	0.801	1.540
C	6.745	-2.294	1.205
C	6.404	-3.661	0.703
C	6.160	-4.020	-0.586
C	6.427	-4.866	1.462
C	6.126	-5.913	0.581
C	6.617	-5.160	2.810
N	6.013	-5.374	-0.671
C	6.034	-7.234	1.005
C	6.511	-6.459	3.235
C	6.230	-7.488	2.338
H	5.016	-0.668	-0.324
H	4.986	-1.964	2.342
H	7.201	-1.813	0.463
H	7.366	-2.409	1.974
H	6.097	-3.387	-1.357
H	5.849	-5.894	-1.511
H	6.831	-4.429	3.465
H	5.830	-7.966	0.347
H	6.637	-6.670	4.203
H	6.172	-8.427	2.679
N	6.073	-0.141	3.577
C	6.435	1.038	4.327
C	7.645	0.739	5.195
O	7.725	-0.299	5.836
C	5.256	1.462	5.220
C	3.967	1.637	4.471
C	3.742	2.774	3.717
C	2.956	0.685	4.527
C	2.584	2.936	2.975
C	1.773	0.844	3.812
C	1.574	1.990	3.068
O	0.444	2.180	2.328
H	5.872	-1.069	4.030
H	6.686	1.766	3.682
H	5.130	0.759	5.914
H	5.494	2.328	5.650
H	4.435	3.505	3.705
H	3.080	-0.131	5.091
H	2.475	3.729	2.374
H	1.073	0.131	3.839
H	0.375	1.463	1.634
N	8.593	1.685	5.246
C	9.743	1.451	6.089
C	9.536	2.222	7.380
O	8.510	2.868	7.577
C	11.069	1.739	5.383
C	11.326	3.206	5.132
H	8.430	2.510	4.679
H	9.752	0.483	6.325
H	11.811	1.376	5.951
H	11.065	1.259	4.503
N	12.400	3.498	4.403
O	10.568	4.054	5.580
H	12.981	2.764	4.050
H	12.627	4.452	4.207
N	10.515	2.087	8.263
C	10.509	2.715	9.568
C	10.484	4.243	9.423
O	9.990	4.896	10.311
C	11.680	2.143	10.387
C	13.097	2.573	9.960
C	13.731	1.992	8.698
H	11.307	1.476	7.929
H	9.669	2.446	10.047
H	11.559	2.413	11.353
H	11.642	1.134	10.341

H	13.096	3.600	9.868
H	13.731	2.381	10.750
N	14.809	2.641	8.268
O	13.305	0.987	8.111
H	15.305	2.308	7.433
H	15.130	3.458	8.768
N	10.976	4.814	8.309
C	10.989	6.267	8.094
C	9.650	6.808	7.568
O	9.513	8.021	7.418
C	12.077	6.612	7.067
C	13.519	6.316	7.478
C	14.461	6.541	6.300
C	13.917	7.166	8.671
H	11.348	4.133	7.611
H	11.185	6.719	8.979
H	11.889	6.088	6.239
H	12.014	7.588	6.875
H	13.585	5.366	7.777
H	14.396	7.492	5.999
H	15.400	6.344	6.582
H	14.204	5.934	5.548
H	13.312	6.966	9.444
H	14.863	6.962	8.929
H	13.841	8.136	8.434
N	8.733	5.912	7.158
C	7.458	6.292	6.561
C	7.533	6.424	5.038
O	6.579	6.832	4.387
H	9.022	4.917	7.308
H	6.756	5.600	6.797
H	7.152	7.175	6.953
N	8.663	6.043	4.442
C	8.739	6.004	2.992
C	7.838	4.876	2.478
O	7.673	3.888	3.179
C	10.185	5.831	2.522
O	11.026	6.852	3.044
H	9.433	5.793	5.073
H	8.382	6.864	2.632
H	10.521	4.946	2.833
H	10.207	5.874	1.527
H	11.108	6.739	4.033
N	7.393	5.011	1.234
C	6.441	4.117	0.586
C	7.029	3.535	-0.698
O	7.363	4.270	-1.617
C	5.134	4.851	0.243
C	4.073	3.922	-0.307
O	4.660	5.475	1.437
H	7.802	5.853	0.733
H	6.238	3.356	1.212
H	5.334	5.574	-0.439
H	3.164	4.218	-0.001
H	4.096	3.929	-1.311
H	4.235	2.985	0.016
H	5.432	5.735	2.029
N	7.074	2.203	-0.763
C	7.576	1.411	-1.881
C	6.389	0.659	-2.514
O	5.805	-0.204	-1.849
C	8.669	0.526	-1.264
C	9.169	-0.674	-2.008
C	9.534	-0.593	-3.335
C	9.431	-1.853	-1.323
C	10.093	-1.686	-3.983
C	9.894	-2.968	-1.989
C	10.259	-2.879	-3.312
H	6.690	1.738	0.121
H	7.906	2.047	-2.571
H	9.467	1.126	-1.078
H	8.320	0.207	-0.365
H	9.400	0.255	-3.841
H	9.281	-1.890	-0.333
H	10.374	-1.596	-4.949
H	9.962	-3.855	-1.495
H	10.643	-3.671	-3.788
N	6.030	1.002	-3.760
C	4.984	0.317	-4.505
C	5.611	-0.545	-5.606
O	6.253	-0.070	-6.529
C	3.953	1.320	-5.069
C	3.348	2.175	-3.947
C	2.873	0.605	-5.877
C	2.495	3.327	-4.434
H	6.574	1.810	-4.147
H	4.546	-0.316	-3.866
H	4.430	1.928	-5.696
H	2.792	1.581	-3.365
H	4.100	2.542	-3.397
H	2.265	1.281	-6.289
H	3.302	0.061	-6.595
H	2.349	0.008	-5.274
H	1.734	2.970	-4.974
H	2.140	3.831	-3.648
H	3.051	3.935	-4.999
N	5.422	-1.852	-5.513
C	6.190	-2.741	-6.363
C	5.282	-3.793	-6.971
O	4.337	-4.251	-6.327
C	7.357	-3.388	-5.599
C	6.866	-4.374	-4.555
C	8.325	-4.045	-6.570
H	4.726	-2.158	-4.827
H	6.584	-2.200	-7.112
H	7.840	-2.661	-5.111
H	7.411	-5.212	-4.601
H	6.955	-3.971	-3.644
H	5.905	-4.595	-4.727
H	8.174	-3.677	-7.487
H	9.263	-3.854	-6.282
H	8.169	-5.033	-6.575
N	5.603	-4.155	-8.216
C	5.034	-5.324	-8.870
C	6.139	-6.343	-9.097
O	7.122	-6.054	-9.758
C	4.346	-4.982	-10.207
C	3.674	-6.179	-10.856
O	3.369	-3.970	-9.956
H	6.299	-3.523	-8.677
H	4.349	-5.729	-8.265
H	5.040	-4.607	-10.845
H	2.687	-6.154	-10.680
H	3.830	-6.163	-11.847
H	4.052	-7.029	-10.480
H	3.812	-3.127	-9.630
N	5.947	-7.550	-8.563
C	6.844	-8.647	-8.864
C	6.336	-9.394	-10.104
O	5.237	-9.948	-10.090
C	6.913	-9.526	-7.653
H	5.127	-7.624	-7.937
H	7.755	-8.285	-9.067
H	6.130	-9.347	-7.050
H	6.897	-10.492	-7.925
H	7.760	-9.348	-7.144
N	7.122	-9.389	-11.180
C	6.790	-10.153	-12.378
C	7.109	-11.634	-12.198
O	7.879	-12.005	-11.312
H	7.976	-8.804	-11.092
H	5.809	-10.048	-12.582
H	7.313	-9.794	-13.161
N	6.541	-12.484	-13.065
C	6.700	-13.931	-12.926
C	8.140	-14.391	-13.240
O	8.554	-15.466	-12.836
C	5.670	-14.621	-13.802
H	5.996	-12.035	-13.819
H	6.488	-14.194	-11.974
H	4.778	-14.605	-13.349
H	5.603	-14.146	-14.680
H	5.944	-15.570	-13.960
N	8.880	-13.560	-13.972
C	10.205	-13.809	-14.519
C	11.337	-13.303	-13.608
O	12.492	-13.347	-13.998
C	10.327	-12.967	-15.796
C	10.283	-11.445	-15.555
O	9.305	-10.938	-14.899
O	11.249	-10.749	-16.017
H	8.380	-12.619	-14.138
H	10.320	-14.814	-14.591
H	11.196	-13.186	-16.231
H	9.573	-13.207	-16.401
N	10.993	-12.672	-12.489
C	11.965	-12.159	-11.541
C	12.118	-10.635	-11.563
O	12.985	-10.138	-10.846
H	9.957	-12.578	-12.353
H	11.704	-12.447	-10.598
H	12.875	-12.581	-11.726
N	11.282	-9.908	-12.343
C	11.329	-8.443	-12.432
C	10.634	-7.751	-11.255
O	9.596	-8.211	-10.768
C	10.716	-7.988	-13.743
H	10.598	-10.490	-12.878
H	12.300	-8.153	-12.446
H	10.072	-7.242	-13.572
H	11.437	-7.668	-14.359
H	10.231	-8.752	-14.171
N	11.247	-6.660	-10.768
C	10.600	-5.723	-9.852
C	10.517	-4.383	-10.564
O	11.513	-3.908	-11.124
C	11.369	-5.579	-8.522
C	11.600	-6.820	-7.667
C	12.477	-6.452	-6.472
C	10.301	-7.443	-7.153
H	12.231	-6.542	-11.104
H	9.679	-6.043	-9.666
H	12.273	-5.192	-8.742
H	10.867	-4.913	-7.959
H	12.061	-7.513	-8.211
H	12.019	-5.750	-5.928
H	12.630	-7.264	-5.910
H	13.354	-6.102	-6.800
H	9.732	-7.710	-7.930
H	10.516	-8.249	-6.602
H	9.812	-6.774	-6.594
N	9.303	-3.802	-10.547
C	9.004	-2.518	-11.161
C	8.018	-1.798	-10.243
O	7.236	-2.444	-9.565
C	8.421	-2.708	-12.575
C	9.217	-3.663	-13.437
O	7.102	-3.224	-12.415
H	8.578	-4.375	-10.039
H	9.837	-1.981	-11.207
H	8.362	-1.805	-13.026
H	9.671	-3.161	-14.178
H	9.915	-4.126	-12.884
H	8.609	-4.354	-13.838
H	7.095	-3.974	-11.743
N	8.123	-0.478	-10.142
C	7.209	0.279	-9.324
C	7.759	1.658	-9.013
O	8.454	2.247	-9.832
H	8.898	-0.060	-10.689
H	6.323	0.376	-9.801
H	7.038	-0.213	-8.457
N	7.385	2.176	-7.845
C	7.714	3.537	-7.495
C	8.067	3.582	-6.021
O	7.609	2.774	-5.215
C	6.565	4.525	-7.796
C	6.184	4.545	-9.256
O	5.429	4.146	-7.013
H	6.854	1.529	-7.234
H	8.515	3.824	-8.020
H	6.854	5.456	-7.513
H	5.292	4.992	-9.375
H	6.872	5.050	-9.787
H	6.126	3.607	-9.612

H 5.721 3.645 -6.191
N 8.844 4.597 -5.693
C 9.337 4.808 -4.353
C 9.052 6.259 -4.024
O 9.335 7.136 -4.837
C 10.853 4.583 -4.250
C 11.338 4.316 -2.843
C 11.632 5.352 -1.975
C 11.470 3.021 -2.367
C 12.070 5.108 -0.685
C 11.878 2.759 -1.065
C 12.186 3.803 -0.231
O 12.573 3.516 1.049
H 9.072 5.239 -6.494
H 8.820 4.239 -3.727
H 11.089 3.799 -4.818
H 11.311 5.400 -4.589
H 11.527 6.303 -2.284
H 11.265 2.250 -2.976
H 12.302 5.872 -0.078
H 11.945 1.813 -0.744
H 12.216 4.215 1.668
N 8.480 6.481 -2.852
C 8.347 7.834 -2.349
C 9.075 7.906 -1.021
O 8.685 7.194 -0.102
C 6.869 8.168 -2.188
C 6.641 9.508 -1.516
C 5.191 9.781 -1.149
O 4.938 10.128 0.041
O 4.323 9.637 -2.047
H 8.152 5.642 -2.351
H 8.753 8.479 -2.990
H 6.471 8.211 -3.082
H 6.464 7.478 -1.624
H 7.166 9.529 -0.679
H 6.926 10.222 -2.137
N 10.143 8.720 -0.954
C 10.932 8.881 0.269
C 10.342 9.999 1.130
O 10.085 11.099 0.648
C 12.410 9.153 -0.010
O 13.162 9.337 1.207
H 10.355 9.220 -1.842
H 10.875 8.028 0.791
H 12.798 8.380 -0.503
H 12.493 9.982 -0.556
H 13.454 10.287 1.263
N 10.186 9.701 2.421
C 9.736 10.667 3.409
C 10.899 11.585 3.818
O 10.666 12.608 4.471
C 9.148 9.917 4.593
H 10.418 8.706 2.658
H 9.013 11.236 3.009
H 8.766 9.047 4.282
H 9.864 9.742 5.268
H 8.425 10.466 5.012
N 12.151 11.209 3.473
C 13.329 11.982 3.873
C 14.200 12.319 2.650
O 14.063 11.755 1.546
C 14.174 11.295 4.980
C 13.355 10.960 6.226
C 14.921 10.056 4.474
H 12.195 10.340 2.911
H 13.016 12.848 4.273
H 14.883 11.944 5.262
H 12.402 11.219 6.075
H 13.411 9.979 6.405
H 13.720 11.464 7.007
H 15.631 9.814 5.132
H 14.273 9.303 4.379
H 15.334 10.264 3.589
N 15.078 13.306 2.872
C 16.088 13.696 1.906
C 15.562 14.654 0.836
O 14.486 15.248 0.985
H 14.967 13.768 3.806
H 16.866 14.139 2.388
H 16.459 12.867 1.449
N 16.366 14.770 -0.237
C 16.112 15.615 -1.399
C 15.259 14.801 -2.385
O 15.686 14.429 -3.488
C 17.451 16.178 -1.910
C 17.363 16.999 -3.185
H 17.238 14.166 -0.159
H 15.552 16.378 -1.098
H 17.842 16.756 -1.185
H 18.072 15.403 -2.072
N 18.523 17.310 -3.747
O 16.276 17.331 -3.669
H 19.384 17.007 -3.342
H 18.539 17.856 -4.590
N 14.018 14.540 -1.948
C 13.073 13.680 -2.639
C 11.697 14.332 -2.573
O 11.292 14.806 -1.512
C 13.053 12.312 -1.982
H 13.788 15.026 -1.039
H 13.342 13.600 -3.596
H 13.841 11.779 -2.292
H 13.094 12.416 -0.988
H 12.211 11.832 -2.230
N 10.968 14.327 -3.689
C 9.607 14.855 -3.699
C 8.722 14.046 -4.652
O 8.912 14.035 -5.876
C 9.621 16.344 -4.061
C 8.240 16.957 -4.264
C 8.241 18.263 -5.043
O 9.312 18.921 -5.109
O 7.184 18.595 -5.622
H 11.437 13.929 -4.518
H 9.220 14.816 -2.773
H 10.055 16.828 -3.321
H 10.117 16.443 -4.906
H 7.688 16.311 -4.769
H 7.851 17.143 -3.374
N 7.703 13.417 -4.072
C 6.810 12.616 -4.874
C 7.515 11.284 -5.164
O 8.380 10.847 -4.393
C 6.401 13.404 -6.130
O 5.224 12.887 -6.735
H 7.621 13.551 -3.057
H 5.994 12.413 -4.343
H 6.241 14.356 -5.870
H 7.151 13.360 -6.791
H 5.347 12.855 -7.725
N 7.110 10.662 -6.278
C 7.428 9.286 -6.599
C 8.584 9.275 -7.596
O 8.726 10.160 -8.440
C 6.174 8.548 -7.111
C 4.908 8.848 -6.306
C 3.816 7.787 -6.285
N 4.327 6.513 -5.770
C 4.626 6.216 -4.493
N 4.113 6.925 -3.494
N 5.397 5.154 -4.233
H 6.529 11.277 -6.909
H 7.719 8.812 -5.770
H 6.015 8.826 -8.050
H 6.348 7.572 -7.059
H 5.173 9.039 -5.337
H 4.492 9.712 -6.660
H 3.084 8.108 -5.698
H 3.492 7.659 -7.214
H 4.474 5.764 -6.453
H 3.482 7.676 -3.676
H 4.361 6.703 -2.547
H 5.724 4.584 -4.985
H 5.640 4.935 -3.291
N 9.390 8.219 -7.495
C 10.529 7.994 -8.361
C 10.422 6.573 -8.867
O 9.903 5.718 -8.139
C 11.824 8.136 -7.572
C 12.071 9.532 -7.078
C 11.502 9.992 -5.902
C 12.879 10.395 -7.805
C 11.716 11.285 -5.457
C 13.107 11.690 -7.366
C 12.506 12.138 -6.203
O 12.693 13.427 -5.769
H 9.121 7.557 -6.721
H 10.465 8.610 -9.138
H 11.779 7.526 -6.785
H 12.582 7.874 -8.164
H 10.921 9.377 -5.359
H 13.301 10.081 -8.651
H 11.303 11.599 -4.601
H 13.705 12.300 -7.889
H 13.611 13.515 -5.385
N 10.903 6.351 -10.092
C 10.886 5.027 -10.680
C 11.838 4.109 -9.908
O 12.945 4.496 -9.575
C 11.228 5.083 -12.177
C 11.446 3.705 -12.771
C 10.142 5.807 -12.973
H 11.279 7.192 -10.568
H 9.962 4.650 -10.592
H 12.092 5.584 -12.273
H 11.365 3.012 -12.053
H 10.759 3.530 -13.478
H 12.359 3.654 -13.178
H 10.329 5.705 -13.949
H 9.254 5.405 -12.754
H 10.147 6.775 -12.726
N 11.404 2.871 -9.660
C 12.341 1.848 -9.243
C 12.264 0.658 -10.168
O 11.234 0.360 -10.770
C 12.117 1.460 -7.792
C 10.935 0.585 -7.428
C 11.183 -0.914 -7.686
C 10.606 0.832 -5.962
H 10.396 2.724 -9.787
H 13.272 2.228 -9.284
H 12.952 0.983 -7.471
H 12.032 2.315 -7.257
H 10.127 0.872 -7.964
H 11.962 -1.213 -7.140
H 10.369 -1.427 -7.426
H 11.375 -1.049 -8.655
H 10.377 1.797 -5.829
H 9.826 0.265 -5.694
H 11.398 0.597 -5.398
N 13.416 0.016 -10.274
C 13.541 -1.243 -10.956
C 14.432 -2.180 -10.144
O 15.392 -1.754 -9.494
C 14.037 -0.984 -12.378
C 15.487 -0.586 -12.490
O 13.868 -2.229 -13.044
H 14.223 0.508 -9.817
H 12.635 -1.668 -11.034
H 13.443 -0.303 -12.814
H 16.058 -1.397 -12.648
H 15.613 0.052 -13.256
H 15.790 -0.138 -11.643
H 13.199 -2.804 -12.558
N 14.132 -3.467 -10.201
C 14.980 -4.445 -9.529
C 14.732 -5.870 -9.995
O 14.082 -6.105 -11.012
H 13.288 -3.708 -10.736
H 15.955 -4.207 -9.688
H 14.824 -4.390 -8.526
N 15.206 -6.807 -9.185
C 15.096 -8.232 -9.475
C 14.880 -8.994 -8.178

O	15.318	-8.567	-7.120
C	16.375	-8.740	-10.154
C	16.779	-8.059	-11.462
C	15.958	-8.394	-12.682
N	16.048	-9.812	-12.984
C	15.182	-10.463	-13.750
N	14.145	-9.814	-14.239
N	15.293	-11.768	-13.920
H	15.666	-6.437	-8.320
H	14.307	-8.382	-10.073
H	17.136	-8.631	-9.505
H	16.258	-9.721	-10.344
H	16.746	-7.049	-11.326
H	17.751	-8.292	-11.662
H	15.012	-8.155	-12.498
H	16.310	-7.870	-13.449
H	16.813	-10.334	-12.589
H	13.994	-8.845	-14.073
H	13.469	-10.316	-14.810
H	16.049	-12.264	-13.482
H	14.633	-12.265	-14.481
N	14.271	-10.179	-8.277
C	14.107	-11.044	-7.133
C	14.298	-12.477	-7.631
O	14.158	-12.749	-8.846
C	12.774	-10.772	-6.442
C	11.559	-11.342	-7.153
C	11.001	-10.731	-8.268
C	10.956	-12.496	-6.683
C	9.906	-11.285	-8.935
C	9.838	-13.033	-7.311
C	9.326	-12.445	-8.448
O	8.216	-13.012	-9.040
H	13.936	-10.410	-9.234
H	14.824	-10.851	-6.475
H	12.808	-11.168	-5.524
H	12.651	-9.781	-6.371
H	11.389	-9.869	-8.608
H	11.330	-12.956	-5.877
H	9.547	-10.846	-9.758
H	9.402	-13.856	-6.930
H	8.109	-12.636	-9.958
N	14.593	-13.341	-6.680
C	14.674	-14.777	-6.911
C	13.262	-15.334	-7.083
O	12.526	-15.465	-6.100
C	15.383	-15.506	-5.777
C	15.546	-17.037	-5.918
O	15.121	-17.626	-6.957
O	16.100	-17.656	-4.955
H	14.765	-12.909	-5.739
H	15.150	-14.915	-7.779
H	16.307	-15.110	-5.675
H	14.875	-15.332	-4.922
N	12.902	-15.678	-8.336
C	11.538	-16.127	-8.589
C	11.367	-17.647	-8.391
O	10.244	-18.141	-8.525
C	11.090	-15.670	-9.941
O	12.042	-16.025	-10.922
H	13.633	-15.597	-9.041
H	10.925	-15.667	-7.929
H	10.221	-16.101	-10.169
H	10.987	-14.678	-9.942
H	12.099	-15.303	-11.612
N	12.436	-18.393	-8.040
C	12.310	-19.819	-7.708
C	13.078	-20.115	-6.427
O	14.080	-20.833	-6.437
C	12.826	-20.685	-8.832
H	13.333	-17.878	-8.027
H	11.342	-20.029	-7.561
H	12.123	-21.344	-9.104
H	13.062	-20.114	-9.621
H	13.642	-21.182	-8.531
N	12.637	-19.564	-5.288
C	13.434	-19.606	-4.060
C	13.506	-21.004	-3.449
O	12.841	-21.921	-3.925
C	12.695	-18.659	-3.116
C	11.269	-18.716	-3.588
C	11.325	-18.920	-5.092
H	14.367	-19.243	-4.228
H	12.767	-18.972	-2.169
H	13.055	-17.730	-3.188
H	10.782	-19.477	-3.146
H	10.791	-17.859	-3.365
H	10.589	-19.516	-5.402
H	11.283	-18.048	-5.575
N	14.328	-21.133	-2.406
C	14.520	-22.394	-1.714
C	13.254	-22.675	-0.924
O	12.499	-21.751	-0.644
C	15.745	-22.335	-0.828
H	14.817	-20.249	-2.136
H	14.647	-23.118	-2.384
H	15.714	-23.077	-0.157
H	16.571	-22.431	-1.385
H	15.773	-21.457	-0.347
N	12.993	-23.953	-0.629
C	11.849	-24.310	0.198
C	12.381	-24.975	1.460
O	12.203	-26.173	1.664
C	10.866	-25.237	-0.532
C	10.072	-24.562	-1.634
O	11.678	-26.302	-1.027
H	13.655	-24.637	-1.028
H	11.369	-23.478	0.458
H	10.216	-25.612	0.133
H	10.688	-24.041	-2.230
H	9.593	-25.252	-2.183
H	9.397	-23.936	-1.235
H	12.404	-25.943	-1.624
N	13.088	-24.186	2.267
C	13.660	-24.648	3.519
C	13.358	-23.627	4.617
O	13.907	-23.734	5.712
C	15.159	-24.932	3.339
C	15.967	-23.740	2.825
O	15.402	-22.626	2.776
O	17.144	-23.936	2.442
H	13.194	-23.204	1.917
H	13.190	-25.494	3.773
H	15.528	-25.205	4.225
H	15.254	-25.682	2.688
N	12.477	-22.647	4.335
C	12.179	-21.571	5.279
C	13.104	-20.348	5.140
O	13.110	-19.462	6.003
H	12.037	-22.728	3.397
H	11.217	-21.268	5.151
H	12.250	-21.927	6.229
N	13.896	-20.339	4.066
C	14.755	-19.224	3.687
C	13.935	-18.067	3.145
O	12.986	-18.285	2.394
C	15.694	-19.628	2.577
O	16.759	-20.374	3.066
H	13.845	-21.231	3.501
H	15.235	-18.905	4.513
H	15.187	-20.172	1.907
H	16.044	-18.801	2.133
H	16.519	-21.348	3.088
N	14.416	-16.843	3.391
C	13.909	-15.684	2.687
C	14.214	-15.721	1.194
O	15.033	-16.504	0.689
H	15.161	-16.808	4.110
H	12.904	-15.620	2.816
H	14.312	-14.841	3.085
N	13.528	-14.819	0.489
C	13.672	-14.645	-0.945
C	14.550	-13.420	-1.214
O	14.149	-12.309	-0.887
C	12.290	-14.459	-1.580
C	12.332	-14.265	-3.079
O	11.543	-15.622	-1.215
H	12.869	-14.242	1.071
H	14.132	-15.447	-1.325
H	11.846	-13.654	-1.172
H	12.968	-14.922	-3.494
H	11.418	-14.406	-3.471
H	12.639	-13.334	-3.298
H	10.672	-15.652	-1.717
N	15.698	-13.641	-1.847
C	16.629	-12.560	-2.136
C	16.086	-11.608	-3.209
O	15.519	-12.011	-4.222
C	17.955	-13.164	-2.519
H	15.867	-14.631	-2.111
H	16.786	-12.028	-1.291
H	18.577	-12.447	-2.841
H	18.365	-13.619	-1.726
H	17.826	-13.837	-3.251
N	16.337	-10.298	-3.023
C	15.885	-9.303	-3.976
C	16.735	-8.045	-3.837
O	17.518	-7.914	-2.891
C	14.380	-9.017	-3.804
C	13.863	-8.642	-2.414
C	14.483	-7.394	-1.881
C	12.330	-8.483	-2.472
H	16.867	-10.082	-2.161
H	16.004	-9.662	-4.903
H	14.146	-8.261	-4.424
H	13.883	-9.839	-4.097
H	14.050	-9.415	-1.787
H	14.288	-6.619	-2.491
H	14.117	-7.184	-0.969
H	15.480	-7.502	-1.809
H	11.925	-9.347	-2.763
H	11.995	-8.239	-1.565
H	12.103	-7.763	-3.124
N	16.597	-7.172	-4.822
C	17.206	-5.859	-4.754
C	16.558	-4.907	-5.733
O	15.921	-5.341	-6.700
H	16.029	-7.503	-5.625
H	17.113	-5.492	-3.818
H	18.192	-5.933	-4.960
N	16.699	-3.604	-5.469
C	16.192	-2.639	-6.429
C	16.987	-1.358	-6.318
O	17.745	-1.188	-5.387
C	14.687	-2.411	-6.241
C	14.320	-1.725	-4.966
C	14.213	-0.384	-4.797
C	13.981	-2.334	-3.703
C	13.688	-1.276	-2.827
C	13.953	-3.651	-3.212
N	13.825	-0.110	-3.526
C	13.376	-1.487	-1.490
C	13.608	-3.871	-1.900
C	13.347	-2.794	-1.049
H	17.163	-3.373	-4.593
H	16.339	-3.013	-7.347
H	14.354	-1.846	-6.991
H	14.227	-3.295	-6.248
H	14.394	0.301	-5.505
H	13.661	0.809	-3.147
H	14.181	-4.418	-3.813
H	13.183	-0.718	-0.880
H	13.545	-4.800	-1.556
H	13.131	-2.981	-0.086
N	16.827	-0.502	-7.324
C	17.520	0.762	-7.430
C	16.479	1.862	-7.682
O	15.511	1.644	-8.411
C	18.497	0.778	-8.612
C	19.228	2.102	-8.770
O	19.413	-0.317	-8.497
H	16.135	-0.836	-8.051
H	17.975	0.961	-6.565
H	17.970	0.661	-9.479
H	20.216	1.959	-8.677

H 19.037 2.491 -9.674
H 18.922 2.746 -8.065
H 20.115 -0.263 -9.215
N 16.726 3.011 -7.059
C 16.063 4.270 -7.370
C 17.154 5.303 -7.698
O 18.057 5.525 -6.912
C 15.180 4.767 -6.212
C 14.578 6.137 -6.527
C 14.070 3.802 -5.826
H 17.460 2.931 -6.303
H 15.510 4.145 -8.186
H 15.763 4.886 -5.412
H 14.585 6.282 -7.515
H 13.639 6.170 -6.190
H 15.120 6.847 -6.080
H 13.291 3.932 -6.439
H 14.406 2.863 -5.904
H 13.789 3.981 -4.883
N 17.067 5.951 -8.858
C 17.819 7.170 -9.130
C 16.929 8.357 -8.786
O 15.759 8.374 -9.165
C 18.253 7.226 -10.579
H 16.419 5.513 -9.549
H 18.624 7.193 -8.544
H 17.459 7.398 -11.164
H 18.919 7.962 -10.704
H 18.672 6.355 -10.841
N 17.458 9.293 -7.996
C 16.650 10.325 -7.367
C 16.408 11.526 -8.291
O 16.652 12.663 -7.891
C 17.293 10.748 -6.064
C 17.312 9.593 -5.117
C 18.404 8.874 -4.738
C 16.182 9.029 -4.425
C 16.672 7.943 -3.670
C 14.816 9.295 -4.389
N 18.041 7.913 -3.848
C 15.841 7.159 -2.876
C 13.991 8.505 -3.610
C 14.498 7.461 -2.851
H 18.491 9.217 -7.873
H 15.745 9.926 -7.154
H 18.222 11.010 -6.228
H 16.760 11.458 -5.652
H 19.336 9.032 -5.068
H 18.660 7.273 -3.384
H 14.427 10.052 -4.920
H 16.221 6.396 -2.341
H 13.010 8.698 -3.601
H 13.882 6.923 -2.280
N 15.912 11.224 -9.480
C 15.491 12.207 -10.475
C 14.089 11.820 -10.947
O 13.836 10.660 -11.292
C 16.462 12.257 -11.654
C 16.041 13.215 -12.775
C 16.750 12.969 -14.067
C 16.201 13.790 -15.222
N 14.765 13.493 -15.474
H 15.843 10.182 -9.658
H 15.449 13.105 -10.050
H 17.353 12.551 -11.317
H 16.534 11.340 -12.043
H 15.044 13.118 -12.913
H 16.220 14.160 -12.465
H 17.724 13.195 -13.961
H 16.671 11.997 -14.312
H 16.303 14.764 -15.006
H 16.730 13.583 -16.049
H 14.359 14.240 -15.999
H 14.688 12.640 -15.990
H 14.287 13.400 -14.601
N 13.155 12.777 -10.844
C 11.833 12.651 -11.441
C 11.560 13.967 -12.177
O 12.500 14.706 -12.464
C 10.784 12.244 -10.401
C 10.631 13.250 -9.278
H 13.462 13.613 -10.300
H 11.882 11.942 -12.130
H 9.905 12.147 -10.864
H 11.056 11.366 -10.009
N 9.978 12.873 -8.193
O 11.064 14.388 -9.411
H 9.612 11.934 -8.148
H 9.847 13.497 -7.432
N 10.290 14.266 -12.474
C 9.936 15.478 -13.211
C 10.114 16.724 -12.341
O 10.273 17.829 -12.847
C 8.504 15.392 -13.747
C 8.360 14.396 -14.880
H 9.593 13.573 -12.140
H 10.583 15.561 -13.977
H 7.906 15.114 -13.000
H 8.239 16.294 -14.079
N 7.127 14.103 -15.257
O 9.345 13.902 -15.424
H 6.346 14.538 -14.802
H 6.967 13.448 -15.996
N 10.090 16.528 -11.026
C 10.047 17.606 -10.059
C 11.434 17.979 -9.533
O 11.625 19.103 -9.073
C 9.168 17.165 -8.883
C 7.691 17.043 -9.173
C 7.167 17.307 -10.436
C 6.808 16.687 -8.162
C 5.807 17.221 -10.684
C 5.446 16.599 -8.394
C 4.946 16.870 -9.657
O 3.600 16.782 -9.905
H 10.105 15.513 -10.749
H 9.657 18.427 -10.509
H 9.500 16.271 -8.566
H 9.286 17.832 -8.140
H 7.787 17.565 -11.182
H 7.164 16.489 -7.247
H 5.447 17.410 -11.599
H 4.824 16.341 -7.654
H 3.119 16.592 -9.050
N 12.375 17.020 -9.501
C 13.603 17.210 -8.745
C 14.692 16.285 -9.252
O 14.419 15.213 -9.788
C 13.460 16.849 -7.265
C 12.526 17.730 -6.463
C 12.898 17.683 -5.002
N 11.896 18.379 -4.215
C 11.989 18.608 -2.915
N 10.964 19.140 -2.270
N 13.089 18.266 -2.260
H 12.149 16.173 -10.042
H 13.909 18.163 -8.857
H 13.124 15.905 -7.208
H 14.370 16.897 -6.845
H 12.602 18.668 -6.778
H 11.594 17.402 -6.561
H 12.940 16.735 -4.720
H 13.775 18.128 -4.889
H 11.069 18.712 -4.692
H 10.109 19.358 -2.730
H 11.047 19.332 -1.279
H 13.840 17.816 -2.749
H 13.173 18.453 -1.283
N 15.932 16.681 -8.999
C 17.062 15.816 -9.294
C 18.116 16.013 -8.208
O 18.724 17.064 -8.120
C 17.597 16.054 -10.702
C 18.670 15.055 -11.079
H 16.019 17.625 -8.588
H 16.745 14.873 -9.223
H 16.843 15.969 -11.345
H 17.986 16.969 -10.742
N 19.137 15.125 -12.317
O 19.050 14.207 -10.252
H 18.782 15.820 -12.947
H 19.843 14.488 -12.626
N 18.319 14.985 -7.377
C 19.291 15.028 -6.299
C 20.650 14.446 -6.728
O 21.545 14.337 -5.906
C 18.730 14.287 -5.115
H 17.717 14.154 -7.575
H 19.436 15.989 -6.022
H 17.855 14.692 -4.841
H 18.583 13.324 -5.352
H 19.369 14.339 -4.344
N 20.807 14.055 -7.983
C 22.083 13.549 -8.503
C 22.656 12.426 -7.626
O 23.840 12.413 -7.297
C 23.132 14.662 -8.604
C 22.635 15.841 -9.406
H 19.950 14.135 -8.575
H 21.923 13.174 -9.426
H 23.361 14.983 -7.687
H 23.949 14.304 -9.051
C 22.487 17.152 -9.107
N 22.236 15.691 -10.717
C 21.865 16.892 -11.201
N 21.996 17.783 -10.238
H 22.220 14.842 -11.230
H 22.692 17.591 -8.237
H 21.551 17.062 -12.125
N 21.809 11.434 -7.366
C 22.179 10.360 -6.464
C 21.332 9.153 -6.843
O 20.309 9.304 -7.543
C 22.018 10.805 -5.034
O 20.717 11.227 -4.744
H 20.897 11.498 -7.848
H 23.131 10.128 -6.623
H 22.257 10.040 -4.425
H 22.655 11.564 -4.855
H 20.501 12.056 -5.265
N 21.739 7.994 -6.314
C 20.990 6.754 -6.507
C 21.149 5.912 -5.250
O 22.250 5.843 -4.702
C 21.476 6.009 -7.727
H 22.617 8.054 -5.765
H 20.027 6.974 -6.627
H 22.411 5.683 -7.576
H 20.880 5.224 -7.906
H 21.465 6.617 -8.523
N 20.071 5.205 -4.903
C 20.127 4.221 -3.838
C 19.817 2.841 -4.395
O 18.894 2.679 -5.213
C 19.139 4.570 -2.734
C 19.276 3.632 -1.547
O 19.389 5.924 -2.320
H 19.217 5.419 -5.450
H 21.057 4.208 -3.459
H 18.193 4.525 -3.102
H 18.507 2.988 -1.530
H 20.133 3.116 -1.617
H 19.280 4.159 -0.694
H 20.376 6.113 -2.329
N 20.556 1.857 -3.890
C 20.225 0.456 -4.067
C 19.984 -0.174 -2.709
O 20.762 0.042 -1.773
C 21.313 -0.370 -4.772
C 21.667 0.174 -6.143
O 22.494 -0.446 -3.938
H 21.395 2.184 -3.355
H 19.381 0.389 -4.602
H 20.971 -1.321 -4.914
H 21.114 -0.282 -6.845

H	21.489	1.161	-6.176
H	22.638	0.011	-6.336
H	22.619	0.417	-3.443
N	18.951	-1.012	-2.678
C	18.560	-1.857	-1.556
C	18.812	-3.314	-1.926
O	18.325	-3.777	-2.964
C	17.067	-1.674	-1.233
C	16.657	-0.350	-0.662
C	16.384	-0.076	0.647
C	16.376	0.876	-1.382
C	15.960	1.834	-0.430
C	16.404	1.265	-2.728
N	15.957	1.221	0.795
C	15.582	3.129	-0.784
C	16.026	2.546	-3.076
C	15.592	3.458	-2.119
H	18.399	-1.015	-3.596
H	19.152	-1.635	-0.783
H	16.540	-1.808	-2.078
H	16.796	-2.380	-0.570
H	16.481	-0.731	1.401
H	15.687	1.650	1.657
H	16.698	0.618	-3.436
H	15.313	3.790	-0.076
H	16.066	2.818	-4.037
H	15.285	4.362	-2.409
N	19.458	-4.030	-1.014
C	19.722	-5.437	-1.178
C	19.142	-6.115	0.062
O	19.409	-5.681	1.180
C	21.204	-5.670	-1.329
O	21.483	-7.054	-1.464
H	19.755	-3.481	-0.172
H	19.221	-5.768	-1.964
H	21.532	-5.198	-2.142
H	21.676	-5.327	-0.521
H	22.371	-7.173	-1.906
N	18.316	-7.141	-0.144
C	17.725	-7.773	1.015
C	16.902	-8.999	0.653
O	17.171	-9.713	-0.330
H	18.159	-7.408	-1.108
H	18.457	-8.045	1.664
H	17.131	-7.107	1.499
N	16.021	-9.347	1.590
C	15.246	-10.563	1.400
C	13.839	-10.371	1.962
O	13.638	-9.658	2.944
C	15.995	-11.773	1.960
C	16.196	-11.751	3.451
C	16.886	-13.008	3.933
H	15.948	-8.726	2.392
H	15.160	-10.730	0.416
H	15.480	-12.604	1.720
H	16.899	-11.820	1.518
H	16.761	-10.967	3.693
H	15.309	-11.684	3.900
N	17.896	-12.825	4.785
O	16.508	-14.120	3.544
H	18.403	-13.624	5.166
H	18.152	-11.879	5.047
N	12.896	-11.002	1.250
C	11.486	-11.031	1.589
C	11.204	-12.258	2.461
O	11.650	-13.374	2.166
C	10.668	-11.013	0.296
C	9.256	-11.514	0.459
C	8.255	-10.699	0.972
C	8.949	-12.837	0.163
C	6.958	-11.177	1.153
C	7.672	-13.334	0.370
C	6.661	-12.491	0.813
O	5.373	-12.990	1.004
H	13.285	-11.491	0.394
H	11.267	-10.216	2.126
H	10.632	-10.073	-0.036
H	11.131	-11.591	-0.373
H	8.463	-9.751	1.218
H	9.657	-13.436	-0.204
H	6.249	-10.579	1.523
H	7.475	-14.302	0.201
H	4.749	-12.498	0.405
N	10.518	-11.998	3.579
C	10.074	-13.018	4.516
C	8.550	-12.932	4.565
O	7.996	-11.914	5.000
C	10.699	-12.762	5.906
C	10.392	-13.905	6.871
C	12.205	-12.518	5.820
H	10.325	-10.971	3.722
H	10.318	-13.907	4.154
H	10.274	-11.942	6.282
H	11.243	-14.366	7.120
H	9.958	-13.538	7.692
H	9.776	-14.556	6.429
H	12.455	-11.789	6.455
H	12.687	-13.358	6.063
H	12.440	-12.250	4.887
N	7.868	-13.936	4.020
C	6.412	-13.913	3.926
C	5.724	-14.326	5.238
O	6.382	-14.745	6.190
H	8.452	-14.722	3.674
H	6.104	-12.981	3.676
H	6.111	-14.539	3.189
N	4.385	-14.212	5.279
C	3.640	-14.454	6.510
C	2.645	-13.339	6.830
O	2.480	-12.370	6.068
H	3.948	-13.944	4.381
H	3.138	-15.332	6.432
H	4.290	-14.549	7.283
N	1.985	-13.485	7.991
C	0.897	-12.586	8.363
C	1.465	-11.197	8.648
O	0.793	-10.206	8.335
C	0.113	-13.126	9.539
H	2.312	-14.271	8.578
H	0.256	-12.518	7.598
H	-0.859	-12.913	9.425
H	0.228	-14.119	9.595
H	0.444	-12.709	10.387
N	2.690	-11.157	9.225
C	3.493	-9.941	9.300
C	4.735	-10.105	8.404
O	5.853	-10.343	8.849
C	3.819	-9.563	10.750
C	2.582	-9.206	11.579
C	2.198	-10.270	12.588
O	1.101	-10.859	12.461
O	3.012	-10.514	13.499
H	3.001	-12.077	9.608
H	2.962	-9.159	8.983
H	4.244	-10.338	11.174
H	4.399	-8.774	10.732
H	2.779	-8.378	12.076
H	1.823	-9.091	10.960
N	4.486	-9.995	7.108
C	5.509	-10.085	6.091
C	6.433	-8.880	6.215
O	6.059	-7.791	6.692
C	4.845	-10.182	4.735
H	3.469	-9.835	6.883
H	6.048	-10.915	6.237
H	4.616	-11.136	4.537
H	4.007	-9.635	4.729
H	5.466	-9.840	4.028
N	7.676	-9.100	5.803
C	8.734	-8.124	5.999
C	9.675	-8.213	4.796
O	9.946	-9.321	4.318
C	9.484	-8.505	7.284
C	10.292	-7.420	7.964
C	10.685	-7.896	9.352
N	9.626	-7.626	10.310
C	9.401	-6.445	10.868
N	10.262	-5.459	10.715
N	8.328	-6.253	11.609
H	7.822	-10.019	5.335
H	8.340	-7.212	6.026
H	8.803	-8.849	7.946
H	10.109	-9.266	7.060
H	11.116	-7.245	7.451
H	9.743	-6.606	8.060
H	10.852	-8.873	9.307
H	11.509	-7.412	9.621
H	9.019	-8.391	10.570
H	11.087	-5.570	10.163
H	10.090	-4.569	11.161
H	7.668	-7.001	11.735
H	8.163	-5.370	12.047
N	10.237	-7.062	4.397
C	11.458	-7.043	3.570
C	12.575	-6.369	4.371
O	12.455	-5.196	4.691
C	11.218	-6.354	2.221
C	10.035	-6.981	1.472
C	12.490	-6.356	1.367
C	9.596	-6.216	0.267
H	9.750	-6.207	4.713
H	11.725	-7.993	3.451
H	10.995	-5.396	2.396
H	10.298	-7.910	1.201
H	9.269	-7.051	2.116
H	12.328	-5.831	0.535
H	13.234	-5.942	1.885
H	12.726	-7.295	1.131
H	10.351	-6.150	-0.389
H	8.823	-6.682	-0.170
H	9.313	-5.292	0.534
N	13.612	-7.146	4.712
C	14.777	-6.714	5.480
C	15.920	-6.356	4.525
O	16.284	-7.196	3.695
C	15.272	-7.823	6.391
C	14.260	-8.150	7.469
H	13.509	-8.144	4.361
H	14.524	-5.873	5.959
H	15.428	-8.638	5.846
H	16.114	-7.526	6.827
N	14.017	-9.433	7.675
O	13.637	-7.244	8.017
H	14.495	-10.130	7.137
H	13.353	-9.715	8.371
N	16.340	-5.087	4.529
C	17.311	-4.611	3.544
C	18.477	-3.854	4.183
O	18.376	-3.335	5.295
C	16.685	-3.679	2.500
C	15.474	-4.240	1.802
O	16.355	-2.431	3.117
H	15.930	-4.493	5.267
H	17.688	-5.413	3.053
H	17.369	-3.499	1.764
H	14.639	-3.997	2.306
H	15.407	-3.870	0.870
H	15.537	-5.242	1.747
H	15.928	-2.586	4.015
N	19.591	-3.821	3.427
C	20.682	-2.897	3.574
C	20.751	-2.092	2.290
O	20.319	-2.565	1.233
C	21.968	-3.694	3.844
C	21.823	-4.469	5.160
C	23.075	-4.934	5.860
H	19.583	-4.582	2.675
H	20.470	-2.269	4.315
H	22.102	-4.341	3.120
H	22.719	-3.069	3.937
H	21.289	-3.882	5.808
H	21.232	-5.286	4.975
N	23.246	-6.237	5.881
O	23.822	-4.145	6.446
H	24.086	-6.648	6.328

H	22.554	-6.846	5.458
N	21.157	-0.836	2.406
C	21.128	0.027	1.226
C	22.445	0.801	1.126
O	23.153	0.998	2.125
C	19.877	0.924	1.219
C	19.669	1.811	2.413
C	18.701	1.649	3.367
C	20.353	3.041	2.747
C	19.746	3.547	3.914
C	21.411	3.779	2.189
N	18.782	2.651	4.294
C	20.179	4.718	4.547
C	21.832	4.940	2.811
C	21.220	5.414	3.975
H	21.471	-0.543	3.330
H	21.062	-0.559	0.419
H	19.918	1.522	0.411
H	19.064	0.337	1.142
H	18.033	0.906	3.382
H	18.224	2.723	5.125
H	21.858	3.467	1.349
H	19.733	5.029	5.393
H	22.595	5.452	2.413
H	21.587	6.319	4.445
N	22.764	1.205	-0.111
C	23.893	2.059	-0.430
C	23.308	3.226	-1.203
O	22.718	2.983	-2.245
C	24.919	1.341	-1.324
C	25.623	0.140	-0.692
C	26.444	-0.646	-1.707
C	26.443	0.535	0.504
H	22.102	0.836	-0.850
H	24.287	2.400	0.408
H	24.440	1.016	-2.132
H	25.617	2.001	-1.573
H	24.930	-0.467	-0.286
H	27.142	-0.050	-2.104
H	26.885	-1.419	-1.252
H	25.843	-0.984	-2.431
H	25.854	0.951	1.203
H	26.892	-0.273	0.896
H	27.146	1.199	0.235
N	23.642	4.433	-0.778
C	23.250	5.658	-1.463
C	24.513	6.360	-1.955
O	25.370	6.722	-1.152
C	22.492	6.526	-0.462
C	21.732	7.744	-0.992
C	21.097	8.513	0.168
C	22.545	8.644	-1.873
H	24.218	4.436	0.101
H	22.693	5.421	-2.254
H	21.821	5.940	0.005
H	23.153	6.862	0.215
H	21.016	7.437	-1.614
H	21.870	8.853	0.863
H	20.604	9.305	-0.191
H	20.461	7.914	0.653
H	22.872	8.138	-2.677
H	21.987	9.419	-2.184
H	23.428	9.024	-1.322
N	24.649	6.450	-3.275
C	25.816	7.084	-3.864
C	25.401	8.420	-4.471
O	24.398	8.498	-5.174
C	26.467	6.259	-4.977
C	27.676	6.940	-5.571
O	26.881	4.996	-4.466
H	23.874	6.041	-3.824
H	26.486	7.261	-3.143
H	25.777	6.095	-5.715
H	27.453	7.888	-5.819
H	28.430	6.949	-4.907
H	27.978	6.452	-6.396
H	26.152	4.311	-4.581
N	26.138	9.459	-4.085
C	25.997	10.788	-4.654
C	27.109	11.006	-5.680
O	28.289	10.682	-5.442
C	26.147	11.876	-3.549
C	25.302	11.600	-2.279
C	25.828	13.264	-4.094
C	26.046	10.697	-1.291
H	27.071	9.267	-3.678
H	25.139	10.791	-5.163
H	27.220	11.922	-3.251
H	25.088	12.552	-1.745
H	24.323	11.150	-2.551
H	26.529	13.550	-4.896
H	25.928	14.023	-3.301
H	24.819	13.287	-4.505
H	27.129	10.931	-1.277
H	25.724	10.861	-0.260
H	25.857	9.631	-1.526
N	26.742	11.615	-6.821
C	27.739	12.059	-7.781
C	28.695	13.062	-7.131
O	28.261	13.988	-6.455
H	25.725	11.731	-6.941
H	28.266	11.271	-8.113
H	27.287	12.497	-8.563
N	29.997	12.809	-7.257
C	31.030	13.630	-6.650
C	32.157	13.812	-7.670
O	32.292	13.024	-8.616
C	31.691	13.031	-5.394
C	30.705	12.767	-4.272
O	32.378	11.851	-5.708
H	30.210	11.960	-7.837
H	30.651	14.528	-6.442
H	32.412	13.777	-4.986
H	30.068	13.658	-4.094
H	30.065	11.914	-4.537
H	31.255	12.518	-3.341
H	33.288	12.101	-6.056
N	33.030	14.789	-7.389
C	34.336	14.826	-8.034
C	35.160	13.625	-7.583
O	34.908	12.995	-6.543
C	35.088	16.122	-7.709
C	34.355	17.354	-8.186
O	35.258	16.133	-6.288
H	32.702	15.482	-6.702
H	34.207	14.765	-9.025
H	35.999	16.091	-8.138
H	33.365	17.187	-8.191
H	34.550	18.129	-7.577
H	34.648	17.592	-9.117
H	34.838	15.316	-5.878
N	36.216	13.354	-8.335
C	37.122	12.262	-7.985
C	37.800	12.516	-6.628
O	37.947	11.581	-5.859
C	38.105	12.026	-9.144
C	37.383	11.758	-10.469
C	38.285	11.590	-11.677
O	38.121	12.342	-12.655
O	39.147	10.709	-11.630
H	36.341	13.959	-9.162
H	36.611	11.396	-7.941
H	38.635	12.836	-9.256
H	38.634	11.236	-8.931
H	36.861	10.921	-10.365
H	36.782	12.526	-10.649
N	38.142	13.772	-6.282
C	38.737	14.108	-4.986
C	37.791	13.803	-3.815
O	38.251	13.531	-2.707
C	39.099	15.578	-4.942
H	37.944	14.483	-7.021
H	39.579	13.562	-4.864
H	40.054	15.683	-4.662
H	38.975	15.984	-5.849
H	38.510	16.052	-4.286
N	36.471	13.915	-4.038
C	35.484	13.734	-2.954
C	34.783	12.410	-3.119
O	33.856	12.099	-2.322
C	34.523	14.939	-2.879
C	35.223	16.159	-2.371
H	36.217	14.139	-5.017
H	35.988	13.653	-1.962
H	34.054	15.139	-3.856
H	33.697	14.717	-2.173
N	35.422	17.234	-3.151
O	35.531	16.242	-1.158
H	35.179	17.236	-4.162
H	35.923	18.050	-2.749
N	35.351	11.456	-3.914
C	34.749	10.129	-4.067
C	34.769	9.308	-2.767
O	33.877	8.472	-2.571
C	35.393	9.359	-5.208
H	36.162	11.788	-4.457
H	33.784	10.242	-4.355
H	36.157	8.817	-4.858
H	34.718	8.749	-5.624
H	35.730	10.000	-5.897
N	35.802	9.421	-1.876
C	35.848	8.699	-0.583
C	34.625	8.979	0.280
O	34.046	8.020	0.853
C	37.152	9.017	0.180
C	37.220	10.451	0.631
C	37.709	11.485	-0.068
C	36.766	10.966	1.833
C	36.995	12.312	1.783
C	36.171	10.376	2.950
N	37.564	12.648	0.615
C	36.682	13.141	2.860
C	35.842	11.181	3.989
C	36.117	12.572	3.953
H	36.620	9.991	-2.183
H	35.852	7.606	-0.793
H	37.240	8.349	1.064
H	38.025	8.793	-0.471
H	38.181	11.417	-1.047
H	37.877	13.595	0.298
H	35.978	9.310	2.984
H	36.908	14.194	2.826
H	35.354	10.758	4.860
H	35.869	13.174	4.820
N	34.072	10.234	0.336
C	32.781	10.542	1.001
C	31.659	10.642	-0.006
O	30.747	11.498	0.163
C	32.893	11.800	1.895
C	33.384	13.052	1.146
C	33.410	14.285	2.060
C	34.198	15.424	1.396
N	34.440	16.532	2.330
H	34.534	10.960	-0.252
H	32.493	9.703	1.671
H	31.907	12.010	2.365
H	33.588	11.577	2.725
H	34.415	12.878	0.786
H	32.738	13.250	0.261
H	32.368	14.612	2.267
H	33.890	14.016	3.026
H	35.178	15.032	1.028
H	33.632	15.784	0.502
H	35.021	17.264	1.853
H	33.543	16.970	2.635
H	34.970	16.206	3.173
N	31.499	9.654	-0.936
C	30.343	9.592	-1.849
C	29.275	8.655	-1.353
O	28.130	8.802	-1.815
C	30.745	9.257	-3.309
O	31.247	7.967	-3.453
H	32.179	8.867	-0.924
H	29.863	10.595	-1.905
H	29.868	9.277	-3.968

H	31.472	10.008	-3.687
H	32.193	7.998	-3.127
N	29.483	7.656	-0.438
C	28.500	6.544	-0.326
C	27.945	6.401	1.062
O	28.620	5.807	1.929
C	29.056	5.230	-0.915
C	28.039	4.111	-0.904
O	29.466	5.451	-2.265
H	30.419	7.539	0.007
H	27.649	6.723	-0.954
H	29.872	4.993	-0.370
H	28.498	3.227	-1.029
H	27.547	4.101	-0.029
H	27.376	4.240	-1.647
H	29.986	6.309	-2.339
N	26.712	6.888	1.403
C	26.021	6.428	2.619
C	25.699	4.928	2.601
O	25.233	4.339	1.565
C	24.751	7.241	2.940
C	24.956	8.725	3.313
C	23.585	9.378	3.454
C	25.689	8.917	4.636
H	26.240	7.542	0.750
H	26.703	6.587	3.463
H	24.089	7.195	2.066
H	24.219	6.746	3.784
H	25.518	9.248	2.509
H	22.933	8.757	4.101
H	23.658	10.408	3.849
H	23.084	9.414	2.488
H	26.718	8.523	4.576
H	25.770	9.987	4.903
H	25.150	8.394	5.455
N	25.733	4.283	3.804
C	25.157	2.966	4.070
C	24.100	3.005	5.111
O	24.201	3.764	6.113
C	26.236	1.938	4.387
C	26.984	2.203	5.690
C	25.771	0.507	4.301
H	26.098	4.793	4.628
H	24.643	2.624	3.191
H	26.910	2.000	3.566
H	27.464	3.201	5.669
H	26.305	2.129	6.563
H	27.775	1.443	5.793
H	25.304	0.329	3.435
H	26.557	-0.104	4.391
H	25.139	0.356	5.061
N	23.034	2.180	4.971
C	22.011	2.090	6.000
C	21.219	0.807	5.857
O	21.608	-0.097	5.127
H	23.016	1.655	4.088
H	22.459	2.121	7.011
H	21.389	2.878	5.925
N	20.174	0.701	6.674
C	19.318	-0.477	6.690
C	17.879	0.003	6.835
O	17.638	0.994	7.511
C	19.726	-1.451	7.784
C	19.938	-0.792	9.091
C	19.164	-0.618	10.197
N	21.173	-0.249	9.373
C	21.144	0.269	10.602
N	19.927	0.084	11.115
H	20.025	1.526	7.294
H	19.405	-0.948	5.819
H	19.008	-2.147	7.887
H	20.579	-1.908	7.512
H	21.958	-0.241	8.763
H	18.233	-0.932	10.325
H	21.956	0.788	11.106
N	16.974	-0.624	6.077
C	15.551	-0.336	6.076
C	14.800	-1.662	6.270
O	15.093	-2.663	5.614
C	15.088	0.335	4.785
C	15.399	1.819	4.589
O	15.838	2.463	5.569
O	15.213	2.325	3.424
H	17.401	-1.373	5.458
H	15.354	0.224	6.875
H	15.498	-0.175	4.007
H	14.081	0.216	4.717
N	13.790	-1.668	7.158
C	12.871	-2.808	7.234
C	11.502	-2.338	6.745
O	10.986	-1.354	7.247
C	12.732	-3.386	8.647
C	11.975	-4.698	8.666
O	14.022	-3.642	9.177
H	13.726	-0.833	7.753
H	13.197	-3.520	6.622
H	12.269	-2.695	9.240
H	11.006	-4.533	8.870
H	12.047	-5.149	7.772
H	12.357	-5.305	9.368
H	14.664	-2.906	8.926
N	10.927	-3.045	5.768
C	9.664	-2.701	5.169
C	8.578	-3.654	5.670
O	8.829	-4.854	5.771
C	9.740	-2.796	3.655
C	10.647	-1.759	3.024
C	12.000	-2.016	2.862
C	10.156	-0.515	2.670
C	12.818	-1.056	2.287
C	10.983	0.449	2.113
C	12.317	0.168	1.933
H	11.493	-3.887	5.476
H	9.411	-1.787	5.497
H	10.095	-3.687	3.417
H	8.832	-2.660	3.289
H	12.384	-2.880	3.153
H	9.186	-0.305	2.817
H	13.798	-1.276	2.134
H	10.599	1.345	1.847
H	12.929	0.865	1.539
N	7.403	-3.110	6.012
C	6.265	-3.965	6.299
C	5.049	-3.452	5.532
O	5.065	-2.365	4.959
C	5.983	-4.047	7.808
C	7.156	-4.535	8.621
O	5.602	-2.761	8.281
H	7.388	-2.085	6.049
H	6.468	-4.892	5.981
H	5.207	-4.690	7.961
H	7.223	-5.536	8.567
H	8.009	-4.135	8.273
H	7.044	-4.270	9.584
H	6.189	-2.047	7.880
N	3.937	-4.197	5.635
C	2.684	-3.858	4.962
C	1.858	-2.749	5.628
O	0.979	-2.216	4.964
C	1.796	-5.103	4.879
C	2.399	-6.251	4.100
C	2.753	-5.876	2.679
C	1.812	-6.475	1.657
N	2.217	-6.082	0.284
H	4.049	-5.042	6.241
H	2.899	-3.564	4.004
H	1.612	-5.414	5.812
H	0.935	-4.839	4.442
H	3.234	-6.564	4.571
H	1.743	-7.016	4.081
H	2.725	-4.877	2.591
H	3.683	-6.195	2.481
H	1.835	-7.471	1.735
H	0.884	-6.147	1.833
H	1.566	-6.457	-0.376
H	2.227	-5.084	0.212
H	3.131	-6.439	0.091
N	2.145	-2.377	6.887
C	1.328	-1.448	7.660
C	2.104	-0.155	7.942
O	3.313	-0.181	8.175
C	0.871	-2.135	8.965
C	0.287	-3.520	8.684
C	1.990	-2.227	9.999
H	3.018	-2.820	7.266
H	0.521	-1.207	7.114
H	0.137	-1.586	9.356
H	0.957	-4.218	8.932
H	-0.543	-3.645	9.226
H	0.068	-3.595	7.712
H	1.950	-3.119	10.447
H	2.871	-2.118	9.540
H	1.870	-1.503	10.677
N	1.422	1.002	7.900
C	2.111	2.265	8.147
C	2.606	2.277	9.593
O	1.930	1.764	10.480
C	1.247	3.477	7.769
C	1.183	3.734	6.267
C	1.267	5.186	5.837
C	-0.080	5.782	5.486
N	0.021	7.236	5.230
H	0.423	0.915	7.689
H	2.899	2.333	7.538
H	0.323	3.314	8.099
H	1.633	4.282	8.209
H	1.941	3.218	5.819
H	0.315	3.338	5.908
H	1.667	5.721	6.585
H	1.863	5.251	5.033
H	-0.435	5.325	4.666
H	-0.715	5.622	6.247
H	0.981	7.488	5.099
H	-0.498	7.469	4.406
H	-0.351	7.741	6.010
N	3.817	2.812	9.880
C	4.450	2.615	11.181
C	4.100	3.745	12.162
O	3.421	3.440	13.130
C	5.944	2.588	10.815
C	6.057	3.403	9.515
C	4.647	3.650	9.001
O	4.379	4.915	12.156
H	4.198	1.720	11.574
H	6.498	3.012	11.531
H	6.260	1.653	10.659
H	6.530	4.272	9.704
H	6.601	2.887	8.842
H	4.398	4.612	9.088
H	4.558	3.364	8.048
C	25.778	14.248	4.011
N	24.126	13.744	1.497
O	18.970	12.659	0.279
S	17.373	6.368	0.497
C	24.884	13.180	4.576
N	20.211	13.265	-1.694
O	13.318	5.630	2.540
C	26.936	14.052	3.219
N	15.036	7.340	2.509
O	24.428	18.528	2.539
C	27.596	12.756	2.791
N	15.656	5.117	3.059
O	23.954	18.430	0.201
C	27.473	15.301	2.992
N	23.922	16.428	1.498
C	28.767	15.543	2.251
C	26.649	16.271	3.574
C	26.987	17.745	3.684
C	25.563	15.595	4.196
C	24.395	16.196	4.943
C	22.963	15.737	0.697
C	23.052	14.286	0.732
C	21.879	16.370	-0.076
C	22.146	13.436	-0.060
C	20.993	15.530	-0.910

C	21.109	14.060	-0.905
C	22.358	11.988	-0.039
C	24.393	12.338	1.451
C	23.469	11.442	0.739
C	19.191	12.484	-1.075
C	18.293	11.602	-1.900
C	17.163	10.880	-1.113
C	17.711	9.752	-0.225
C	16.622	9.076	0.648
C	17.245	7.935	1.518
C	16.449	7.395	2.719
C	17.824	5.431	1.849
C	16.912	5.849	3.092
C	14.540	6.010	2.690
C	24.143	17.817	1.387
C	24.058	19.773	-0.080
C	23.056	20.578	0.744
C	25.480	20.266	0.165
C	23.723	19.946	-1.555
Ir	25.485	15.205	2.051
H	25.237	12.166	4.330
H	23.862	13.306	4.165
H	24.855	13.252	5.686
H	20.452	13.067	-2.690
H	14.884	7.570	1.547
H	27.604	12.674	1.679
H	27.113	11.861	3.226
H	28.652	12.736	3.136
H	15.738	4.444	2.324
H	28.959	16.626	2.112
H	28.712	15.076	1.243
H	29.612	15.092	2.809
H	27.013	18.221	2.686
H	26.252	18.304	4.293
H	27.982	17.867	4.158
H	24.366	17.298	4.843
H	23.453	15.806	4.506
H	24.457	15.922	6.017
H	21.748	17.442	-0.066
H	20.254	15.999	-1.529
H	21.758	11.335	-0.656
H	25.284	11.920	1.884
H	23.711	10.395	0.636
H	17.826	12.239	-2.680
H	18.921	10.851	-2.426
H	16.447	10.433	-1.841
H	16.582	11.604	-0.504
H	18.185	8.979	-0.874
H	18.490	10.174	0.442
H	15.830	8.688	-0.032
H	16.162	9.838	1.315
H	18.155	8.416	1.809
H	16.663	8.089	3.505
H	17.586	4.414	1.617
H	18.829	5.702	2.095
H	17.429	5.698	4.016
H	23.035	21.634	0.403
H	22.040	20.147	0.633
H	23.328	20.578	1.816
H	25.584	21.327	-0.141
H	26.200	19.658	-0.420
H	25.751	20.183	1.234
H	23.790	21.012	-1.858
H	22.698	19.577	-1.761
H	24.426	19.363	-2.183
N	26.146	15.212	0.121
C	26.720	16.400	-0.386
O	27.177	17.356	0.500
C	26.586	16.747	-1.820
H	26.010	17.688	-1.867
H	25.993	15.956	-2.302
C	27.946	16.904	-2.523
H	28.343	15.905	-2.821
H	28.680	17.350	-1.813
C	27.878	17.832	-3.752
H	27.386	18.796	-3.495
H	28.930	18.092	-4.009
C	27.211	17.197	-4.998
C	25.679	17.396	-4.997
C	27.795	17.825	-6.283
H	27.438	16.106	-5.023
C	25.053	16.752	-6.237
H	25.439	18.482	-4.982
H	25.215	16.932	-4.111
C	27.152	17.237	-7.544
H	27.649	18.929	-6.280
H	28.890	17.634	-6.340
C	25.630	17.374	-7.513
H	23.953	16.897	-6.213
H	25.260	15.657	-6.234
H	27.436	16.167	-7.647
H	27.548	17.774	-8.436
H	25.217	16.854	-8.402
H	25.354	18.449	-7.577
N	25.294	11.144	-20.758
C	26.171	9.959	-20.534
C	25.509	8.678	-21.019
O	26.019	7.599	-20.749
C	26.476	9.693	-19.056
C	26.762	10.932	-18.238
O	27.754	11.629	-18.546
O	25.971	11.203	-17.327
H	24.387	11.074	-21.178
H	26.994	10.106	-21.093
H	25.684	9.222	-18.662
H	27.272	9.087	-19.013
N	24.357	8.783	-21.685
C	23.702	7.593	-22.199
C	24.720	6.846	-23.042
O	24.889	5.636	-22.918
C	22.470	7.985	-23.013
C	21.889	6.861	-23.829
C	20.566	7.246	-24.466
O	19.907	6.355	-25.001
O	20.211	8.450	-24.425
H	23.997	9.739	-21.793
H	23.401	7.017	-21.447
H	21.779	8.289	-22.378
H	22.739	8.703	-23.632
H	22.512	6.644	-24.555
H	21.718	6.097	-23.238
N	25.428	7.634	-23.860
C	26.397	7.130	-24.817
C	27.623	6.539	-24.115
O	28.090	5.446	-24.468
C	26.767	8.277	-25.745
H	25.216	8.657	-23.745
H	25.967	6.402	-25.369
H	27.118	7.911	-26.606
H	25.957	8.835	-25.927
H	27.470	8.843	-25.314
N	28.130	7.254	-23.094
C	29.311	6.820	-22.375
C	29.052	5.573	-21.524
O	29.959	4.775	-21.334
H	27.604	8.123	-22.880
H	30.056	6.618	-23.032
H	29.636	7.566	-21.771
N	27.834	5.419	-20.975
C	27.530	4.251	-20.137
C	27.242	2.996	-20.978
O	27.793	1.928	-20.716
C	26.401	4.587	-19.142
C	26.892	5.647	-18.152
C	25.903	3.331	-18.436
C	25.805	6.296	-17.358
H	27.161	6.166	-21.188
H	28.366	4.056	-19.613
H	25.646	4.971	-19.655
H	27.545	5.211	-17.529
H	27.383	6.350	-18.674
H	25.144	3.568	-17.832
H	25.593	2.669	-19.115
H	26.645	2.935	-17.899
H	25.315	5.605	-16.823
H	26.198	6.975	-16.734
H	25.159	6.752	-17.974
N	26.427	3.147	-22.033
C	26.046	2.047	-22.901
C	27.277	1.350	-23.448
O	28.166	1.981	-24.003
C	25.148	2.514	-24.063
C	24.779	1.362	-24.972
O	23.962	3.127	-23.545
H	26.093	4.130	-22.170
H	25.516	1.381	-22.362
H	25.655	3.212	-24.610
H	25.593	0.810	-25.176
H	24.094	0.778	-24.527
H	24.399	1.711	-25.833
H	24.148	4.078	-23.277
N	27.299	0.028	-23.333
C	28.411	-0.750	-23.820
C	28.802	-1.884	-22.898
O	28.023	-2.309	-22.031
H	26.462	-0.381	-22.871
H	28.182	-1.132	-24.738
H	29.217	-0.140	-23.958
N	30.020	-2.365	-23.129
C	30.567	-3.493	-22.411
C	31.625	-2.991	-21.429
O	32.532	-2.243	-21.771
C	31.052	-4.568	-23.391
C	31.678	-5.733	-22.664
O	29.914	-5.046	-24.138
H	30.545	-1.851	-23.878
H	29.838	-3.931	-21.885
H	31.704	-4.151	-24.047
H	31.273	-6.596	-22.980
H	32.667	-5.757	-22.836
H	31.524	-5.647	-21.675
H	29.115	-5.126	-23.535
N	31.477	-3.401	-20.170
C	32.404	-3.060	-19.112
C	32.959	-4.300	-18.441
O	32.336	-5.349	-18.410
C	31.653	-2.226	-18.103
C	31.157	-0.926	-18.607
C	29.978	-0.675	-19.225
C	31.836	0.325	-18.496
C	30.991	1.288	-19.046
C	33.047	0.712	-17.934
N	29.860	0.653	-19.458
C	31.309	2.639	-19.057
C	33.375	2.043	-17.946
C	32.525	2.988	-18.527
H	30.624	-3.995	-20.016
H	33.180	-2.565	-19.528
H	30.857	-2.744	-17.779
H	32.256	-2.034	-17.324
H	29.299	-1.368	-19.471
H	29.062	1.109	-19.872
H	33.664	0.039	-17.530
H	30.676	3.313	-19.435
H	34.233	2.342	-17.535
H	32.819	3.948	-18.553
N	34.176	-4.195	-17.907
C	34.826	-5.312	-17.253
C	35.328	-4.887	-15.880
O	35.853	-3.791	-15.747
C	36.042	-5.755	-18.086
C	35.716	-6.041	-19.530
C	35.712	-5.012	-20.473
C	35.325	-7.314	-19.943
C	35.382	-5.259	-21.804
C	34.977	-7.575	-21.267
C	35.024	-6.545	-22.200
O	34.665	-6.714	-23.524
H	34.607	-3.251	-18.003
H	34.152	-6.045	-17.124
H	36.719	-5.026	-18.052
H	36.410	-6.584	-17.675
H	35.949	-4.082	-20.191
H	35.294	-8.058	-19.275
H	35.403	-4.516	-22.473

H	34.697	-8.490	-21.542
H	34.217	-5.884	-23.847
N	35.259	-5.762	-14.881
C	35.757	-5.385	-13.565
C	37.104	-6.038	-13.335
O	37.661	-6.678	-14.230
C	34.779	-5.571	-12.397
C	34.457	-6.985	-12.002
H	34.848	-6.670	-15.109
H	35.905	-4.395	-13.577
H	35.166	-5.090	-11.592
H	33.915	-5.098	-12.641
N	33.427	-7.113	-11.175
O	35.131	-7.938	-12.400
H	32.915	-6.322	-10.857
H	33.154	-8.033	-10.861
N	37.630	-5.779	-12.135
C	38.951	-6.230	-11.753
C	38.972	-7.761	-11.637
O	40.041	-8.338	-11.764
C	39.383	-5.425	-10.515
C	38.793	-5.883	-9.169
C	37.379	-5.441	-8.832
H	37.004	-5.227	-11.506
H	39.597	-5.976	-12.478
H	40.387	-5.468	-10.438
H	39.120	-4.460	-10.650
H	38.824	-6.902	-9.146
H	39.416	-5.559	-8.430
N	36.927	-5.838	-7.649
O	36.699	-4.746	-9.584
H	35.987	-5.571	-7.339
H	37.520	-6.408	-7.056
N	37.824	-8.427	-11.386
C	37.782	-9.888	-11.231
C	37.693	-10.636	-12.569
O	37.633	-11.862	-12.542
C	36.541	-10.305	-10.417
C	36.518	-9.904	-8.941
C	35.155	-10.219	-8.313
C	37.656	-10.586	-8.184
H	36.986	-7.819	-11.314
H	38.630	-10.183	-10.761
H	35.744	-9.892	-10.852
H	36.474	-11.299	-10.460
H	36.694	-8.923	-8.865
H	34.978	-11.199	-8.386
H	35.166	-9.949	-7.352
H	34.445	-9.712	-8.798
H	38.531	-10.311	-8.582
H	37.626	-10.313	-7.223
H	37.553	-11.578	-8.254
N	37.472	-9.935	-13.688
C	37.156	-10.602	-14.942
C	35.660	-10.652	-15.292
O	35.299	-11.094	-16.386
H	37.545	-8.910	-13.579
H	37.653	-10.138	-15.707
H	37.516	-11.559	-14.916
N	34.765	-10.159	-14.426
C	33.349	-10.143	-14.775
C	33.063	-9.169	-15.920
O	33.802	-8.202	-16.104
C	32.544	-9.830	-13.526
O	32.791	-10.840	-12.557
H	35.150	-9.813	-13.542
H	33.092	-11.067	-15.087
H	32.832	-8.959	-13.162
H	31.582	-9.829	-13.750
H	33.710	-10.725	-12.185
N	31.963	-9.426	-16.646
C	31.486	-8.650	-17.777
C	30.079	-8.125	-17.538
O	29.137	-8.875	-17.256
C	31.355	-9.477	-19.077
C	31.466	-8.631	-20.317
O	32.339	-10.498	-19.122
H	31.446	-10.295	-16.300
H	32.101	-7.859	-17.907
H	30.429	-9.916	-19.088
H	30.650	-8.053	-20.418
H	32.276	-8.038	-20.263
H	31.549	-9.215	-21.131
H	32.967	-10.423	-18.337
N	29.946	-6.816	-17.714
C	28.729	-6.053	-17.498
C	28.354	-5.487	-18.868
O	29.105	-4.671	-19.396
C	29.100	-5.010	-16.420
C	28.116	-3.922	-16.158
C	26.782	-4.219	-15.930
C	28.551	-2.612	-15.972
C	25.871	-3.207	-15.645
C	27.645	-1.601	-15.706
C	26.301	-1.897	-15.542
H	30.849	-6.350	-18.042
H	28.019	-6.693	-17.253
H	29.248	-5.513	-15.565
H	29.965	-4.590	-16.706
H	26.466	-5.161	-15.968
H	29.527	-2.405	-16.031
H	24.899	-3.440	-15.517
H	27.969	-0.642	-15.633
H	25.644	-1.169	-15.350
N	27.243	-5.929	-19.464
C	26.784	-5.409	-20.747
C	25.501	-4.627	-20.516
O	24.535	-5.141	-19.972
C	26.606	-6.573	-21.737
C	27.901	-7.386	-21.813
C	26.133	-6.047	-23.086
C	27.792	-8.672	-22.624
H	26.739	-6.672	-18.934
H	27.471	-4.752	-21.046
H	25.891	-7.165	-21.390
H	28.613	-6.812	-22.217
H	28.178	-7.623	-20.881
H	25.966	-6.814	-23.703
H	25.288	-5.529	-22.964
H	26.834	-5.453	-23.476
H	27.531	-8.446	-23.560
H	28.675	-9.135	-22.621
H	27.100	-9.259	-22.212
N	25.520	-3.334	-20.789
C	24.415	-2.474	-20.390
C	23.998	-1.532	-21.519
O	24.859	-1.085	-22.282
C	24.808	-1.697	-19.105
C	25.931	-0.694	-19.339
C	23.599	-1.041	-18.455
H	26.357	-2.998	-21.292
H	23.623	-3.059	-20.169
H	25.147	-2.368	-18.442
H	25.541	0.215	-19.483
H	26.533	-0.676	-18.541
H	26.455	-0.963	-20.147
H	23.236	-0.338	-19.066
H	22.898	-1.734	-18.286
H	23.875	-0.624	-17.589
N	22.698	-1.198	-21.536
C	22.123	-0.096	-22.295
C	21.507	0.918	-21.348
O	20.698	0.572	-20.479
C	21.074	-0.590	-23.306
C	20.475	0.522	-24.136
O	21.729	-1.516	-24.177
H	22.105	-1.833	-20.924
H	22.857	0.355	-22.810
H	20.344	-1.082	-22.807
H	20.206	1.288	-23.544
H	21.145	0.851	-24.807
H	19.663	0.189	-24.625
H	22.201	-2.227	-23.643
N	21.923	2.177	-21.499
C	21.301	3.285	-20.790
C	20.255	3.898	-21.709
O	20.595	4.316	-22.819
C	22.325	4.308	-20.371
H	22.719	2.291	-22.159
H	20.847	2.937	-19.975
H	21.906	5.218	-20.340
H	22.682	4.082	-19.462
H	23.084	4.320	-21.026
N	19.003	3.912	-21.245
C	17.930	4.628	-21.904
C	17.927	6.118	-21.585
O	18.539	6.589	-20.616
H	18.871	3.360	-20.365
H	18.008	4.506	-22.910
H	17.037	4.230	-21.628
N	17.176	6.867	-22.405
C	17.134	8.320	-22.303
C	16.422	8.762	-21.017
O	16.635	9.874	-20.526
C	16.456	8.883	-23.542
H	16.636	6.323	-23.108
H	18.076	8.674	-22.280
H	16.409	9.880	-23.475
H	16.980	8.630	-24.355
H	15.530	8.512	-23.615
N	15.593	7.853	-20.488
C	14.683	8.030	-19.367
C	15.341	7.741	-18.003
O	14.719	8.004	-16.978
C	13.520	7.043	-19.557
C	13.885	5.551	-19.432
O	14.751	5.020	-20.236
O	13.303	4.900	-18.504
H	15.663	6.907	-21.003
H	14.406	9.005	-19.351
H	12.822	7.250	-18.867
H	13.133	7.193	-20.469
N	16.562	7.180	-17.999
C	17.263	6.774	-16.778
C	17.334	5.257	-16.533
O	17.846	4.841	-15.484
H	16.972	7.058	-18.956
H	18.216	7.146	-16.801
H	16.812	7.214	-15.972
N	16.854	4.423	-17.463
C	16.894	2.965	-17.313
C	18.261	2.373	-17.645
O	18.984	2.858	-18.517
C	15.809	2.308	-18.147
H	16.451	4.898	-18.299
H	16.668	2.735	-16.352
H	14.973	2.223	-17.604
H	15.623	2.866	-18.956
H	16.110	1.399	-18.435
N	18.638	1.323	-16.892
C	19.756	0.462	-17.249
C	19.179	-0.930	-17.418
O	18.375	-1.380	-16.612
C	20.859	0.405	-16.181
C	21.494	1.740	-15.796
C	22.427	1.600	-14.599
C	22.233	2.363	-16.983
H	18.057	1.187	-16.036
H	20.127	0.760	-18.116
H	20.461	0.014	-15.356
H	21.583	-0.184	-16.526
H	20.766	2.395	-15.572
H	23.160	0.957	-14.821
H	22.822	2.492	-14.378
H	21.911	1.260	-13.813
H	21.587	2.519	-17.729
H	22.637	3.233	-16.701
H	22.954	1.741	-17.287
N	19.577	-1.597	-18.489
C	19.059	-2.913	-18.779
C	20.219	-3.651	-19.414
O	21.028	-3.041	-20.099
C	17.789	-2.878	-19.644
C	16.696	-1.980	-19.108
O	18.169	-2.416	-20.935

H 20.272 -1.104 -19.080
H 18.825 -3.363 -17.933
H 17.428 -3.820 -19.732
H 15.801 -2.290 -19.442
H 16.693 -2.002 -18.104
H 16.847 -1.035 -19.412
H 18.825 -1.655 -20.861
N 20.368 -4.942 -19.139
C 21.401 -5.697 -19.810
C 21.646 -7.018 -19.111
O 20.705 -7.633 -18.618
H 19.717 -5.327 -18.446
H 21.128 -5.871 -20.766
H 22.258 -5.164 -19.824
N 22.897 -7.449 -19.120
C 23.272 -8.763 -18.642
C 24.610 -8.675 -17.933
O 25.457 -7.826 -18.212
C 23.359 -9.816 -19.760
C 22.078 -9.996 -20.550
O 24.402 -9.386 -20.620
H 23.588 -6.760 -19.501
H 22.584 -9.076 -17.983
H 23.618 -10.701 -19.352
H 22.287 -10.084 -21.528
H 21.600 -10.823 -20.244
H 21.477 -9.203 -20.415
H 24.514 -8.385 -20.572
N 24.754 -9.547 -16.969
C 25.927 -9.581 -16.141
C 26.470 -10.998 -16.152
O 25.736 -11.976 -15.997
C 25.612 -9.168 -14.705
C 26.838 -8.805 -13.919
C 27.625 -9.758 -13.315
C 27.227 -7.472 -13.817
C 28.761 -9.406 -12.593
C 28.371 -7.114 -13.132
C 29.140 -8.074 -12.514
O 30.234 -7.682 -11.808
H 23.950 -10.210 -16.855
H 26.621 -8.980 -16.535
H 25.009 -8.378 -14.734
H 25.162 -9.931 -14.254
H 27.383 -10.731 -13.390
H 26.667 -6.766 -14.249
H 29.303 -10.112 -12.134
H 28.644 -6.146 -13.084
H 30.869 -7.200 -12.413
N 27.776 -11.092 -16.326
C 28.437 -12.380 -16.321
C 29.550 -12.331 -15.293
O 30.523 -11.601 -15.501
C 28.969 -12.690 -17.714
C 29.676 -14.025 -17.739
C 29.723 -14.660 -19.120
O 30.099 -15.845 -19.186
O 29.373 -13.956 -20.119
H 28.269 -10.190 -16.462
H 27.788 -13.097 -16.079
H 28.209 -12.741 -18.320
H 29.616 -12.002 -17.948
H 30.608 -13.892 -17.446
H 29.193 -14.649 -17.147
N 29.385 -13.091 -14.195
C 30.354 -13.064 -13.115
C 31.420 -14.132 -13.334
O 31.091 -15.259 -13.683
C 29.723 -13.241 -11.769
O 30.710 -13.489 -10.772
H 28.532 -13.673 -14.199
H 30.811 -12.169 -13.122
H 29.225 -12.413 -11.518
H 29.097 -14.018 -11.790
H 30.600 -14.418 -10.424
N 32.678 -13.781 -13.037
C 33.797 -14.708 -13.164
C 34.004 -15.525 -11.881
O 34.833 -16.424 -11.882
C 35.043 -13.932 -13.527
H 32.782 -12.797 -12.711
H 33.607 -15.349 -13.920
H 34.834 -13.275 -14.253
H 35.380 -13.437 -12.724
H 35.753 -14.561 -13.847
N 33.246 -15.249 -10.794
C 33.423 -15.933 -9.517
C 32.056 -16.281 -8.939
O 31.024 -15.718 -9.351
C 34.204 -15.124 -8.451
C 35.672 -14.967 -8.815
C 33.540 -13.770 -8.153
H 32.532 -14.508 -10.958
H 33.915 -16.788 -9.685
H 34.166 -15.642 -7.596
H 35.852 -14.019 -9.078
H 36.240 -15.207 -8.027
H 35.894 -15.571 -9.581
H 33.055 -13.833 -7.284
H 34.247 -13.069 -8.100
H 32.900 -13.556 -8.887
N 32.101 -17.161 -7.929
C 30.918 -17.516 -7.165
C 30.050 -18.532 -7.908
O 30.461 -19.116 -8.924
H 33.046 -17.557 -7.749
H 31.193 -17.907 -6.275
H 30.370 -16.687 -6.982
N 28.830 -18.694 -7.388
C 27.864 -19.659 -7.899
C 27.035 -18.959 -8.971
O 25.923 -18.487 -8.722
C 27.036 -20.262 -6.755
C 25.935 -21.180 -7.243
H 28.628 -18.062 -6.575
H 28.376 -20.381 -8.352
H 27.650 -20.783 -6.167
H 26.625 -19.513 -6.240
N 25.052 -21.566 -6.334
O 25.832 -21.461 -8.438
H 25.129 -21.249 -5.388
H 24.301 -22.178 -6.591
N 27.628 -18.918 -10.166
C 27.136 -18.174 -11.305
C 27.675 -18.819 -12.582
O 28.873 -19.088 -12.644
C 27.613 -16.743 -11.192
H 28.517 -19.496 -10.205
H 26.138 -18.220 -11.325
H 27.373 -16.376 -10.292
H 28.607 -16.707 -11.307
H 27.180 -16.184 -11.901
N 26.797 -19.007 -13.581
C 27.169 -19.476 -14.914
C 26.430 -18.679 -15.983
O 25.225 -18.484 -15.887
C 26.850 -20.963 -15.088
C 26.827 -21.409 -16.538
C 26.762 -22.915 -16.756
O 26.878 -23.337 -17.957
O 26.598 -23.660 -15.732
H 25.805 -18.783 -13.314
H 28.160 -19.373 -15.044
H 27.551 -21.474 -14.628
H 25.959 -21.123 -14.710
H 26.026 -21.015 -16.972
H 27.656 -21.085 -16.978
N 27.180 -18.230 -17.003
C 26.610 -17.514 -18.124
C 26.109 -16.134 -17.640
O 26.625 -15.575 -16.656
C 25.524 -18.376 -18.820
O 25.344 -17.984 -20.175
H 28.196 -18.445 -16.908
H 27.326 -17.348 -18.799
H 25.812 -19.326 -18.790
H 24.669 -18.256 -18.329
H 25.517 -17.005 -20.262
N 25.134 -15.590 -18.382
C 24.644 -14.245 -18.201
C 23.375 -14.284 -17.365
O 22.534 -15.180 -17.531
C 24.381 -13.612 -19.571
C 25.644 -13.383 -20.386
C 25.328 -12.901 -21.783
N 25.610 -13.876 -22.829
C 25.057 -15.081 -22.941
N 24.377 -15.612 -21.941
N 25.227 -15.777 -24.050
H 24.757 -16.246 -19.116
H 25.332 -13.704 -17.712
H 23.787 -14.219 -20.085
H 23.942 -12.733 -19.428
H 26.208 -12.694 -19.935
H 26.151 -14.240 -20.457
H 24.352 -12.658 -21.816
H 25.869 -12.069 -21.955
H 26.292 -13.614 -23.539
H 24.247 -15.110 -21.086
H 23.982 -16.532 -22.036
H 25.754 -15.391 -24.812
H 24.831 -16.693 -24.138
N 23.248 -13.255 -16.527
C 22.077 -12.977 -15.717
C 21.493 -11.644 -16.128
O 22.248 -10.720 -16.455
C 22.479 -12.917 -14.235
C 23.038 -14.217 -13.755
C 24.339 -14.607 -14.015
C 22.219 -15.108 -13.093
C 24.794 -15.852 -13.637
C 22.688 -16.323 -12.652
C 23.970 -16.717 -12.948
O 24.366 -17.957 -12.509
H 24.111 -12.634 -16.504
H 21.393 -13.689 -15.892
H 23.168 -12.213 -14.134
H 21.669 -12.702 -13.708
H 24.961 -13.976 -14.486
H 21.258 -14.863 -12.929
H 25.737 -16.133 -13.864
H 22.090 -16.927 -12.111
H 24.585 -17.907 -11.536
N 20.163 -11.533 -16.032
C 19.465 -10.305 -16.291
C 19.878 -9.277 -15.232
O 19.935 -9.615 -14.043
C 17.941 -10.508 -16.331
C 17.213 -9.172 -16.354
C 17.535 -11.352 -17.545
H 19.687 -12.425 -15.751
H 19.749 -9.950 -17.181
H 17.674 -10.971 -15.489
H 16.235 -9.327 -16.492
H 17.356 -8.699 -15.485
H 17.569 -8.610 -17.101
H 16.539 -11.368 -17.611
H 17.925 -10.944 -18.368
H 17.883 -12.280 -17.426
N 20.160 -8.040 -15.674
C 20.260 -6.928 -14.726
C 19.344 -5.801 -15.138
O 18.997 -5.611 -16.316
C 21.699 -6.442 -14.596
C 22.295 -5.622 -15.734
C 21.967 -4.116 -15.652
C 23.787 -5.851 -15.702
H 20.292 -7.953 -16.685
H 19.980 -7.260 -13.820
H 21.757 -5.879 -13.762
H 22.285 -7.251 -14.473
H 21.950 -5.978 -16.616
H 22.328 -3.752 -14.797
H 22.389 -3.650 -16.426
H 20.978 -3.998 -15.682

H	23.985	-6.826	-15.828
H	24.227	-5.329	-16.436
H	24.159	-5.553	-14.820
N	18.911	-5.073	-14.122
C	18.178	-3.855	-14.346
C	18.640	-2.841	-13.315
O	18.917	-3.206	-12.160
C	16.681	-4.134	-14.265
C	16.238	-4.559	-12.897
O	15.972	-2.957	-14.634
H	19.142	-5.450	-13.184
H	18.397	-3.502	-15.253
H	16.444	-4.872	-14.929
H	16.454	-3.846	-12.221
H	15.245	-4.721	-12.884
H	16.704	-5.409	-12.625
H	16.551	-2.357	-15.199
N	18.631	-1.579	-13.714
C	18.916	-0.498	-12.779
C	18.511	0.862	-13.341
O	17.685	0.939	-14.247
H	18.412	-1.431	-14.713
H	18.418	-0.660	-11.915
H	19.904	-0.488	-12.567
N	19.009	1.918	-12.692
C	18.669	3.300	-12.970
C	19.946	4.136	-12.905
O	20.884	3.831	-12.155
C	17.675	3.863	-11.945
C	16.368	3.106	-11.743
C	15.466	3.213	-12.973
N	14.987	4.585	-13.132
C	14.473	5.063	-14.271
N	14.239	4.268	-15.290
N	14.174	6.334	-14.366
H	19.698	1.646	-11.930
H	18.292	3.368	-13.891
H	18.145	3.910	-11.052
H	17.444	4.806	-12.223
H	16.562	2.143	-11.586
H	15.877	3.491	-10.968
H	15.997	2.954	-13.765
H	14.696	2.607	-12.840
H	15.046	5.203	-12.347
H	14.420	3.285	-15.232
H	13.869	4.645	-16.150
H	14.309	6.950	-13.582
H	13.805	6.709	-15.222
N	19.968	5.238	-13.661
C	21.056	6.203	-13.592
C	20.458	7.598	-13.704
O	19.307	7.749	-14.126
C	22.128	5.951	-14.657
C	21.741	6.388	-16.064
C	20.822	5.671	-16.825
C	22.263	7.552	-16.626
C	20.434	6.097	-18.099
C	21.920	7.969	-17.908
C	20.990	7.252	-18.645
O	20.610	7.706	-19.918
H	19.148	5.341	-14.294
H	21.496	6.122	-12.701
H	22.953	6.449	-14.396
H	22.321	4.971	-14.683
H	20.425	4.826	-16.456
H	22.905	8.107	-16.089
H	19.757	5.573	-18.617
H	22.346	8.787	-18.299
H	19.772	7.241	-20.180
N	21.251	8.591	-13.287
C	20.876	10.003	-13.337
C	21.041	10.494	-14.773
O	22.164	10.619	-15.240
C	21.762	10.847	-12.427
C	21.431	12.343	-12.438
O	20.553	12.757	-13.218
O	22.057	13.069	-11.688
H	22.178	8.263	-12.916
H	19.905	10.061	-13.117
H	21.659	10.512	-11.492
H	22.710	10.735	-12.721
N	19.929	10.718	-15.485
C	20.020	11.036	-16.904
C	20.250	12.534	-17.136
O	20.380	12.945	-18.280
C	18.816	10.521	-17.667
O	17.594	10.808	-16.983
H	19.050	10.647	-14.960
H	20.812	10.543	-17.288
H	18.784	10.955	-18.565
H	18.892	9.531	-17.774
H	16.966	10.042	-17.100
N	20.328	13.350	-16.081
C	20.666	14.773	-16.210
C	21.651	15.160	-15.109
O	21.278	15.787	-14.109
C	19.413	15.622	-16.140
H	20.130	12.894	-15.166
H	21.102	14.920	-17.097
H	18.654	15.075	-15.785
H	19.568	16.403	-15.534
H	19.180	15.956	-17.054
N	22.914	14.691	-15.192
C	23.869	14.905	-14.102
C	24.288	16.360	-13.926
O	23.865	17.231	-14.684
C	25.054	14.014	-14.485
C	24.943	13.815	-15.971
C	23.463	13.874	-16.288
H	23.498	14.533	-13.228
H	25.925	14.460	-14.251
H	25.007	13.133	-14.004
H	25.440	14.538	-16.460
H	25.328	12.925	-16.235
H	23.300	14.312	-17.171
H	23.056	12.961	-16.286
N	25.076	16.622	-12.885
C	25.649	17.945	-12.684
C	26.808	18.166	-13.666
O	27.356	17.222	-14.234
C	26.069	18.115	-11.242
H	25.240	15.821	-12.249
H	24.947	18.638	-12.864
H	26.844	17.513	-11.041
H	26.340	19.065	-11.077
H	25.306	17.882	-10.637
N	27.169	19.441	-13.880
C	28.145	19.810	-14.902
C	29.384	20.444	-14.275
O	30.107	21.184	-14.937
C	27.518	20.753	-15.938
C	26.843	19.985	-17.058
O	26.609	21.617	-15.239
H	26.697	20.130	-13.261
H	28.429	18.975	-15.389
H	28.246	21.325	-16.348
H	27.508	19.387	-17.512
H	26.098	19.423	-16.688
H	26.465	20.624	-17.733
H	25.784	21.110	-14.971
N	29.633	20.121	-13.004
C	30.730	20.688	-12.243
C	31.884	19.689	-12.188
O	32.733	19.808	-11.305
C	30.266	21.066	-10.834
C	29.713	19.913	-10.004
O	29.595	18.782	-10.530
O	29.421	20.149	-8.824
H	28.962	19.419	-12.607
H	31.063	21.490	-12.744
H	31.049	21.459	-10.347
H	29.552	21.764	-10.921
N	31.878	18.700	-13.102
C	32.855	17.624	-13.121
C	32.514	16.447	-12.191
O	33.326	15.545	-12.026
H	31.099	18.782	-13.800
H	32.951	17.269	-14.072
H	33.769	17.990	-12.858
N	31.315	16.447	-11.603
C	30.853	15.344	-10.767
C	30.583	14.111	-11.617
O	30.119	14.202	-12.757
C	29.614	15.711	-10.014
O	29.908	16.619	-8.968
H	30.744	17.299	-11.793
H	31.584	15.118	-10.116
H	28.960	16.142	-10.633
H	29.208	14.890	-9.615
H	29.862	17.555	-9.316
N	30.733	12.931	-10.996
C	30.303	11.697	-11.622
C	28.775	11.580	-11.734
O	27.999	12.375	-11.191
H	31.170	12.989	-10.061
H	30.702	11.628	-12.553
H	30.651	10.904	-11.091
N	28.346	10.529	-12.450
C	26.941	10.270	-12.707
C	26.508	9.086	-11.842
O	26.966	7.981	-12.093
C	26.735	9.911	-14.181
C	25.311	9.542	-14.525
O	27.200	11.080	-14.876
H	29.118	9.917	-12.803
H	26.409	11.067	-12.433
H	27.329	9.146	-14.420
H	24.674	10.008	-13.904
H	25.102	9.815	-15.469
H	25.181	8.550	-14.439
H	26.767	11.142	-15.781
N	25.635	9.335	-10.874
C	25.159	8.277	-9.977
C	24.361	7.233	-10.743
O	23.536	7.568	-11.579
C	24.332	8.891	-8.891
H	25.323	10.321	-10.806
H	25.957	7.830	-9.556
H	23.791	9.654	-9.256
H	23.706	8.208	-8.504
H	24.924	9.238	-8.158
N	24.529	5.965	-10.345
C	23.777	4.873	-10.904
C	23.784	3.683	-9.952
O	24.492	3.675	-8.951
C	24.360	4.528	-12.277
C	25.873	4.341	-12.368
C	26.344	3.064	-11.725
C	26.301	4.358	-13.835
H	25.252	5.851	-9.597
H	22.822	5.162	-11.036
H	23.935	3.674	-12.582
H	24.112	5.264	-12.907
H	26.322	5.130	-11.922
H	25.915	2.276	-12.174
H	27.340	2.985	-11.809
H	26.098	3.059	-10.753
H	26.047	5.234	-14.244
H	27.291	4.235	-13.893
H	25.843	3.615	-14.323
N	22.977	2.685	-10.294
C	22.977	1.423	-9.596
C	22.205	0.392	-10.388
O	21.419	0.750	-11.268
H	22.352	2.898	-11.104
H	23.923	1.105	-9.467
H	22.553	1.534	-8.690
N	22.433	-0.869	-10.053
C	21.730	-1.951	-10.709
C	21.719	-3.167	-9.791
O	22.469	-3.219	-8.809
C	22.343	-2.313	-12.080
C	23.714	-2.920	-12.022
C	24.018	-4.247	-12.070
C	24.978	-2.235	-12.084

C	25.985	-3.209	-12.067
C	25.361	-0.892	-12.115
N	25.371	-4.419	-12.035
C	27.358	-2.902	-12.089
C	26.704	-0.581	-12.157
C	27.687	-1.570	-12.111
H	23.136	-1.005	-9.305
H	20.780	-1.669	-10.845
H	21.744	-2.971	-12.530
H	22.408	-1.482	-12.627
H	23.347	-4.987	-12.123
H	25.843	-5.309	-11.992
H	24.675	-0.165	-12.108
H	28.038	-3.628	-12.087
H	26.976	0.381	-12.221
H	28.649	-1.296	-12.094
N	20.852	-4.117	-10.160
C	20.633	-5.358	-9.443
C	20.725	-6.525	-10.413
O	20.188	-6.425	-11.511
C	19.235	-5.444	-8.839
C	19.006	-6.730	-8.060
O	19.059	-4.299	-7.992
H	20.326	-3.873	-11.048
H	21.352	-5.464	-8.753
H	18.546	-5.395	-9.581
H	18.672	-6.515	-7.139
H	18.327	-7.297	-8.531
H	19.866	-7.241	-7.986
H	18.204	-4.380	-7.469
N	21.365	-7.588	-9.930
C	21.297	-8.935	-10.479
C	20.733	-9.877	-9.422
O	21.255	-9.923	-8.302
C	22.681	-9.411	-10.954
C	22.594	-10.879	-11.359
C	23.231	-8.511	-12.059
H	21.955	-7.361	-9.073
H	20.666	-8.928	-11.255
H	23.300	-9.360	-10.184
H	23.150	-11.429	-10.737
H	21.642	-11.181	-11.312
H	22.933	-10.987	-12.293
H	23.727	-9.071	-12.721
H	22.471	-8.046	-12.511
H	23.849	-7.838	-11.655
N	19.665	-10.626	-9.771
C	19.296	-11.830	-9.021
C	19.964	-13.036	-9.694
O	19.892	-13.158	-10.908
C	17.807	-12.009	-8.961
H	19.147	-10.278	-10.595
H	19.643	-11.752	-8.092
H	17.349	-11.123	-9.069
H	17.504	-12.622	-9.695
H	17.543	-12.405	-8.078
N	20.624	-13.882	-8.904
C	21.550	-14.891	-9.408
C	20.799	-16.170	-9.769
O	21.185	-17.257	-9.298
C	22.661	-15.140	-8.389
C	23.513	-13.921	-8.227
C	23.515	-13.059	-7.173
C	24.412	-13.382	-9.207
C	24.971	-12.211	-8.645
C	24.857	-13.798	-10.468
N	24.405	-12.052	-7.397
C	25.949	-11.459	-9.315
C	25.816	-13.053	-11.125
C	26.348	-11.898	-10.558
H	20.419	-13.759	-7.882
H	21.963	-14.535	-10.251
H	22.258	-15.339	-7.517
H	23.238	-15.865	-8.713
H	22.946	-13.152	-6.355
H	24.623	-11.307	-6.763
H	24.486	-14.627	-10.890
H	26.331	-10.636	-8.891
H	26.130	-13.353	-12.025
H	27.035	-11.378	-11.067
N	19.735	-15.978	-10.550
C	18.969	-17.063	-11.171
C	18.969	-16.837	-12.680
O	18.667	-15.761	-13.180
C	17.524	-17.142	-10.636
C	16.621	-18.159	-11.346
C	15.149	-18.050	-10.978
C	14.258	-19.029	-11.718
N	14.178	-18.688	-13.156
H	19.486	-14.964	-10.686
H	19.417	-17.929	-10.974
H	17.566	-17.387	-9.667
H	17.109	-16.236	-10.732
H	16.718	-18.031	-12.344
H	16.946	-19.089	-11.121
H	15.052	-18.214	-9.991
H	14.835	-17.117	-11.181
H	14.633	-19.951	-11.615
H	13.340	-19.000	-11.318
H	14.219	-17.694	-13.268
H	14.944	-19.109	-13.644
H	13.316	-19.029	-13.534
N	19.236	-17.933	-13.407
C	19.104	-17.953	-14.839
C	18.774	-19.400	-15.236
O	18.320	-20.183	-14.406
C	20.371	-17.402	-15.503
C	21.594	-18.289	-15.379
H	19.543	-18.752	-12.837
H	18.312	-17.407	-15.084
H	20.177	-17.262	-16.480
H	20.580	-16.511	-15.086
N	22.727	-17.812	-15.893
O	21.567	-19.362	-14.761
H	22.734	-16.909	-16.325
H	23.564	-18.353	-15.846
N	19.077	-19.753	-16.487
C	18.681	-21.054	-17.011
C	19.740	-22.110	-16.774
O	19.623	-23.213	-17.317
C	18.321	-20.963	-18.490
C	17.052	-20.162	-18.693
H	19.598	-19.046	-17.028
H	17.855	-21.340	-16.502
H	19.061	-20.512	-18.969
H	18.178	-21.880	-18.837
N	16.847	-19.688	-19.910
O	16.257	-19.985	-17.764
H	17.508	-19.869	-20.641
H	16.028	-19.145	-20.108
N	20.720	-21.795	-15.928
C	21.761	-22.711	-15.554
C	21.795	-22.967	-14.059
O	22.094	-24.096	-13.656
C	23.096	-22.174	-16.054
C	23.166	-22.200	-17.551
C	23.431	-23.379	-18.237
C	22.853	-21.070	-18.287
C	23.468	-23.406	-19.624
C	22.890	-21.074	-19.665
C	23.215	-22.241	-20.329
O	23.183	-22.180	-21.680
H	20.656	-20.810	-15.561
H	21.594	-23.599	-16.018
H	23.191	-21.239	-15.748
H	23.817	-22.746	-15.694
H	23.599	-24.223	-17.725
H	22.592	-20.229	-17.805
H	23.673	-24.255	-20.108
H	22.685	-20.243	-20.181
H	24.081	-21.904	-22.029
N	21.571	-21.913	-13.252
C	21.880	-21.956	-11.833
C	20.838	-21.119	-11.102
O	20.224	-20.211	-11.659
C	23.218	-21.298	-11.462
C	24.438	-21.692	-12.265
C	25.246	-22.806	-11.649
N	26.440	-22.952	-12.468
C	27.678	-22.691	-12.061
N	28.668	-22.707	-12.940
N	27.911	-22.392	-10.790
H	21.166	-21.087	-13.733
H	21.817	-22.896	-11.512
H	23.099	-20.297	-11.543
H	23.402	-21.507	-10.490
H	24.149	-21.986	-13.187
H	25.041	-20.889	-12.370
H	25.499	-22.545	-10.743
H	24.720	-23.629	-11.684
H	26.321	-23.276	-13.420
H	28.512	-22.889	-13.905
H	29.614	-22.527	-12.624
H	27.144	-22.345	-10.144
H	28.840	-22.213	-10.476
N	20.654	-21.432	-9.839
C	19.986	-20.493	-8.943
C	20.738	-20.491	-7.625
O	20.598	-21.431	-6.844
C	18.489	-20.786	-8.796
C	17.744	-19.733	-8.009
H	21.004	-22.354	-9.545
H	20.084	-19.585	-9.341
H	18.091	-20.844	-9.712
H	18.388	-21.666	-8.331
N	16.428	-19.893	-7.859
O	18.379	-18.777	-7.562
H	15.990	-20.701	-8.265
H	15.894	-19.227	-7.356
N	21.443	-19.379	-7.320
C	22.180	-19.296	-6.078
C	21.363	-18.663	-4.953
O	21.930	-18.429	-3.886
C	23.463	-18.527	-6.288
H	21.400	-18.636	-8.031
H	22.446	-20.232	-5.788
H	24.173	-19.140	-6.639
H	23.312	-17.788	-6.947
H	23.770	-18.134	-5.419
N	20.080	-18.352	-5.188
C	19.192	-17.842	-4.156
C	19.783	-16.590	-3.493
O	19.830	-16.453	-2.261
C	18.886	-18.915	-3.105
C	18.259	-20.133	-3.713
H	19.781	-18.510	-6.177
H	18.317	-17.583	-4.580
H	19.736	-19.186	-2.658
H	18.255	-18.538	-2.429
C	18.674	-21.426	-3.715
N	17.123	-20.082	-4.496
C	16.842	-21.329	-4.920
N	17.772	-22.157	-4.463
H	16.599	-19.273	-4.715
H	19.484	-21.787	-3.258
H	16.062	-21.578	-5.482
N	20.229	-15.673	-4.328
C	20.927	-14.503	-3.833
C	20.751	-13.388	-4.866
O	20.322	-13.645	-6.002
C	22.377	-14.831	-3.546
O	23.010	-15.279	-4.730
H	20.044	-15.854	-5.329
H	20.492	-14.206	-2.990
H	22.839	-14.015	-3.222
H	22.420	-15.552	-2.866
H	22.640	-16.170	-4.990
N	21.039	-12.151	-4.444
C	20.962	-10.991	-5.327
C	22.060	-10.005	-4.912
O	22.354	-9.856	-3.729
C	19.585	-10.358	-5.277
H	21.319	-12.093	-3.444
H	21.135	-11.281	-6.263
H	18.899	-11.059	-5.079

H	19.561	-9.660	-4.560
H	19.377	-9.933	-6.159
N	22.669	-9.335	-5.888
C	23.660	-8.297	-5.649
C	23.140	-6.968	-6.192
O	22.523	-6.904	-7.262
C	24.988	-8.623	-6.350
C	26.046	-7.556	-6.157
O	25.461	-9.852	-5.814
H	22.369	-9.624	-6.853
H	23.795	-8.201	-4.662
H	24.812	-8.748	-7.340
H	26.945	-7.983	-6.026
H	26.084	-6.960	-6.964
H	25.829	-6.999	-5.350
H	25.210	-9.936	-4.842
N	23.342	-5.891	-5.427
C	23.111	-4.550	-5.935
C	24.447	-3.816	-5.920
O	25.223	-3.939	-4.977
C	22.082	-3.760	-5.116
C	20.728	-4.401	-5.122
O	22.638	-3.600	-3.802
H	23.669	-6.098	-4.466
H	22.797	-4.611	-6.877
H	21.967	-2.844	-5.525
H	20.810	-5.395	-5.258
H	20.257	-4.237	-4.248
H	20.163	-4.023	-5.864
H	23.083	-4.451	-3.506
N	24.719	-3.078	-7.005
C	25.859	-2.202	-7.130
C	25.362	-0.762	-7.156
O	24.432	-0.437	-7.898
C	26.615	-2.458	-8.453
C	27.324	-3.759	-8.626
C	28.669	-3.941	-8.531
C	26.784	-5.052	-8.953
C	27.882	-5.935	-9.080
C	25.501	-5.543	-9.196
N	29.005	-5.234	-8.812
C	27.748	-7.287	-9.415
C	25.372	-6.870	-9.581
C	26.470	-7.719	-9.672
H	24.004	-3.202	-7.776
H	26.427	-2.307	-6.315
H	25.952	-2.375	-9.214
H	27.303	-1.724	-8.567
H	29.327	-3.221	-8.287
H	29.941	-5.602	-8.817
H	24.699	-4.967	-9.098
H	28.540	-7.883	-9.460
H	24.457	-7.217	-9.798
H	26.319	-8.679	-9.934
N	26.001	0.063	-6.333
C	25.725	1.472	-6.234
C	27.028	2.203	-6.541
O	28.082	1.949	-5.938
C	25.176	1.755	-4.855
O	24.908	3.134	-4.692
H	26.741	-0.419	-5.750
H	25.067	1.721	-6.935
H	24.328	1.246	-4.728
H	25.846	1.475	-4.171
H	24.218	3.259	-3.979
N	26.966	3.169	-7.465
C	28.191	3.821	-7.822
C	28.029	5.053	-8.725
O	26.998	5.711	-8.717
H	26.048	3.366	-7.844
H	28.692	4.109	-6.975
H	28.814	3.158	-8.295
N	29.154	5.378	-9.369
C	29.165	6.507	-10.287
C	30.032	6.195	-11.500
O	31.086	5.578	-11.406
C	29.501	7.807	-9.551
C	30.851	7.916	-8.881
C	30.983	9.235	-8.148
H	29.966	4.786	-9.164
H	28.226	6.655	-10.611
H	29.417	8.578	-10.215
H	28.784	7.963	-8.840
H	30.948	7.177	-8.224
H	31.562	7.864	-9.573
N	31.529	9.174	-6.943
O	30.615	10.299	-8.661
H	31.666	10.023	-6.389
H	31.812	8.271	-6.571
N	29.503	6.687	-12.630
C	30.196	6.734	-13.902
C	31.020	8.014	-13.955
O	30.476	9.078	-13.710
C	29.151	6.709	-15.006
C	29.695	7.095	-16.357
C	30.380	6.186	-17.130
C	29.559	8.395	-16.810
C	30.908	6.544	-18.366
C	30.043	8.760	-18.057
C	30.720	7.833	-18.831
O	31.194	8.214	-20.088
H	28.516	7.043	-12.503
H	30.823	5.959	-13.955
H	28.782	5.785	-15.070
H	28.425	7.350	-14.768
H	30.507	5.244	-16.804
H	29.110	9.076	-16.239
H	31.414	5.877	-18.909
H	29.904	9.691	-18.397
H	30.731	7.670	-20.780
N	32.305	7.867	-14.293
C	33.238	8.957	-14.511
C	33.751	8.887	-15.954
O	34.421	7.915	-16.305
C	34.408	8.848	-13.522
C	35.349	10.041	-13.674
C	33.919	8.669	-12.081
H	32.591	6.849	-14.391
H	32.750	9.821	-14.404
H	34.932	8.036	-13.757
H	34.811	10.874	-13.799
H	35.911	10.126	-12.853
H	35.937	9.900	-14.469
H	32.977	8.991	-12.015
H	33.965	7.702	-11.840
H	34.504	9.200	-11.471
N	33.439	9.909	-16.762
C	33.756	9.923	-18.188
C	35.221	10.269	-18.476
O	36.038	10.340	-17.570
H	32.948	10.697	-16.279
H	33.548	9.013	-18.587
H	33.160	10.597	-18.658
N	35.546	10.458	-19.763
C	36.856	10.936	-20.174
C	37.710	9.821	-20.770
O	37.198	8.754	-21.115
H	34.779	10.232	-20.433
H	36.748	11.672	-20.860
H	37.337	11.330	-19.377
N	39.020	10.095	-20.848
C	39.983	9.257	-21.551
C	39.798	7.791	-21.170
O	39.489	6.959	-22.021
C	41.384	9.727	-21.221
H	39.293	10.981	-20.350
H	39.835	9.351	-22.542
H	41.987	8.937	-21.105
H	41.732	10.301	-21.964
H	41.371	10.258	-20.373
N	39.988	7.527	-19.873
C	39.884	6.213	-19.263
C	38.594	6.161	-18.424
O	38.645	6.296	-17.207
C	41.102	5.969	-18.359
C	42.457	6.117	-19.039
C	42.853	4.955	-19.932
O	41.957	4.232	-20.391
O	44.062	4.758	-20.135
H	40.230	8.395	-19.309
H	39.845	5.507	-19.966
H	41.063	6.629	-17.624
H	41.039	5.044	-18.017
H	42.430	6.931	-19.604
H	43.149	6.199	-18.334
N	37.437	5.980	-19.075
C	36.166	6.019	-18.365
C	36.107	4.860	-17.365
O	36.675	3.787	-17.574
C	35.003	5.996	-19.325
H	37.530	5.817	-20.089
H	36.100	6.879	-17.857
H	34.143	6.104	-18.822
H	35.091	6.744	-19.986
H	34.985	5.124	-19.819
N	35.382	5.084	-16.267
C	35.340	4.102	-15.197
C	33.955	4.164	-14.567
O	33.317	5.211	-14.566
C	36.452	4.362	-14.168
C	37.696	3.519	-14.372
C	38.887	3.944	-13.537
N	39.996	3.009	-13.657
C	40.712	2.802	-14.755
N	41.697	1.922	-14.731
N	40.444	3.459	-15.874
H	34.876	5.982	-16.251
H	35.477	3.194	-15.580
H	36.711	5.322	-14.231
H	36.086	4.165	-13.263
H	37.486	2.556	-14.148
H	37.969	3.558	-15.344
H	39.179	4.845	-13.849
H	38.596	3.991	-12.584
H	40.244	2.469	-12.832
H	41.911	1.390	-13.918
H	42.256	1.780	-15.565
H	39.674	4.107	-15.887
H	40.993	3.316	-16.690
N	33.497	3.010	-14.046
C	32.350	2.975	-13.142
C	32.862	2.390	-11.820
O	33.340	1.270	-11.773
C	31.160	2.177	-13.716
C	30.639	2.775	-15.032
C	30.050	2.059	-12.669
C	29.603	1.933	-15.748
H	34.024	2.168	-14.341
H	32.109	3.915	-12.957
H	31.477	1.247	-13.906
H	30.246	3.675	-14.833
H	31.423	2.908	-15.643
H	29.328	1.468	-13.020
H	30.427	1.668	-11.832
H	29.679	2.965	-12.479
H	28.810	1.809	-15.154
H	29.323	2.396	-16.587
H	29.998	1.043	-15.971
N	32.821	3.213	-10.771
C	33.350	2.857	-9.478
C	32.159	2.557	-8.568
O	31.249	3.376	-8.461
C	34.149	4.007	-8.868
C	35.391	4.364	-9.642
H	32.370	4.137	-10.977
H	33.855	2.006	-9.578
H	33.554	4.813	-8.824
H	34.414	3.744	-7.937
N	35.549	5.654	-9.892
O	36.140	3.479	-10.058
H	34.885	6.327	-9.576
H	36.355	5.967	-10.411
N	32.155	1.379	-7.960
C	30.953	0.907	-7.284
C	31.310	0.274	-5.939

O	32.436	-0.156	-5.712
C	30.148	-0.154	-8.057
C	29.839	0.223	-9.494
O	30.847	-1.396	-8.056
H	33.037	0.851	-8.005
H	30.351	1.693	-7.111
H	29.250	-0.290	-7.587
H	29.744	-0.605	-10.052
H	28.983	0.744	-9.534
H	30.581	0.785	-9.867
H	31.809	-1.260	-8.319
N	30.275	0.239	-5.095
C	30.260	-0.663	-3.963
C	29.056	-1.540	-4.148
O	28.089	-1.162	-4.827
C	30.197	0.172	-2.681
C	31.499	0.959	-2.508
C	31.701	1.636	-1.180
H	29.509	0.899	-5.323
H	31.071	-1.232	-4.009
H	29.457	0.815	-2.751
H	30.097	-0.429	-1.909
H	32.273	0.320	-2.669
H	31.538	1.661	-3.242
N	31.528	2.934	-1.139
O	32.008	0.995	-0.183
H	31.633	3.464	-0.271
H	31.281	3.433	-2.000
N	29.113	-2.729	-3.566
C	28.038	-3.648	-3.782
C	27.623	-4.351	-2.480
O	28.439	-4.448	-1.562
C	28.361	-4.650	-4.887
C	29.605	-5.439	-4.777
C	30.719	-5.263	-5.535
C	29.835	-6.593	-3.958
C	31.138	-7.032	-4.250
C	29.073	-7.310	-3.029
N	31.671	-6.141	-5.147
C	31.740	-8.101	-3.588
C	29.666	-8.365	-2.365
C	30.966	-8.772	-2.655
H	29.941	-2.906	-2.992
H	27.231	-3.131	-4.104
H	27.591	-5.309	-4.953
H	28.399	-4.148	-5.769
H	30.814	-4.587	-6.266
H	32.629	-6.152	-5.460
H	28.124	-7.067	-2.848
H	32.681	-8.363	-3.783
H	29.144	-8.846	-1.659
H	31.349	-9.563	-2.181
N	26.348	-4.780	-2.475
C	25.810	-5.608	-1.409
C	25.246	-6.886	-2.018
O	24.378	-6.817	-2.899
C	24.692	-4.845	-0.693
C	25.070	-3.580	0.092
C	23.819	-2.834	0.592
C	25.913	-3.901	1.290
H	25.792	-4.465	-3.298
H	26.550	-5.855	-0.786
H	24.017	-4.571	-1.384
H	24.258	-5.475	-0.045
H	25.595	-2.971	-0.503
H	23.297	-3.438	1.190
H	24.104	-2.021	1.095
H	23.263	-2.570	-0.192
H	26.763	-4.354	1.004
H	26.146	-3.058	1.785
H	25.413	-4.513	1.910
N	25.625	-8.030	-1.455
C	25.185	-9.331	-1.956
C	24.430	-10.007	-0.820
O	25.045	-10.262	0.218
C	26.403	-10.156	-2.372
C	26.139	-11.517	-3.039
C	27.459	-12.158	-3.480
C	25.385	-12.430	-2.119
H	26.257	-7.920	-0.633
H	24.561	-9.188	-2.711
H	26.937	-9.612	-3.020
H	26.950	-10.332	-1.554
H	25.557	-11.379	-3.836
H	28.043	-12.292	-2.681
H	27.270	-13.039	-3.910
H	27.915	-11.556	-4.133
H	24.501	-12.021	-1.874
H	25.220	-13.314	-2.567
H	25.913	-12.587	-1.279
N	23.110	-10.112	-0.986
C	22.283	-10.796	0.006
C	21.951	-12.220	-0.447
O	21.469	-12.452	-1.570
C	20.971	-10.048	0.275
C	20.141	-10.673	1.389
O	21.310	-8.714	0.659
H	22.740	-9.682	-1.847
H	22.798	-10.853	0.868
H	20.434	-10.007	-0.583
H	20.728	-11.239	1.973
H	19.721	-9.952	1.945
H	19.420	-11.246	0.994
H	21.377	-8.121	-0.151
N	22.068	-13.148	0.501
C	21.696	-14.539	0.282
C	20.450	-14.825	1.111
O	20.333	-14.336	2.237
C	22.844	-15.513	0.623
C	24.128	-15.225	-0.159
C	22.394	-16.952	0.409
C	25.231	-14.641	0.693
H	22.447	-12.793	1.407
H	21.426	-14.612	-0.672
H	23.049	-15.414	1.601
H	24.447	-16.077	-0.566
H	23.910	-14.581	-0.889
H	23.109	-17.575	0.722
H	21.557	-17.120	0.926
H	22.221	-17.106	-0.562
H	25.461	-15.283	1.424
H	26.037	-14.477	0.126
H	24.922	-13.778	1.091
N	19.472	-15.521	0.509
C	18.291	-15.959	1.234
C	18.692	-16.829	2.431
O	19.567	-17.672	2.293
H	19.638	-15.710	-0.497
H	17.780	-15.162	1.566
H	17.697	-16.493	0.626
N	18.194	-16.491	3.627
C	18.563	-17.221	4.840
C	17.303	-17.412	5.684
O	16.299	-16.739	5.492
C	19.641	-16.525	5.710
C	20.939	-16.226	4.988
O	19.106	-15.324	6.270
H	17.542	-15.688	3.615
H	18.907	-18.118	4.580
H	19.894	-17.152	6.474
H	20.832	-16.396	4.005
H	21.196	-15.266	5.127
H	21.672	-16.813	5.342
H	18.597	-15.526	7.114
N	17.380	-18.360	6.631
C	16.488	-18.401	7.781
C	16.727	-17.176	8.664
O	17.765	-16.497	8.581
C	16.728	-19.684	8.598
C	16.668	-20.937	7.747
O	18.031	-19.555	9.160
H	18.137	-19.062	6.466
H	15.541	-18.373	7.456
H	16.056	-19.722	9.348
H	17.058	-21.713	8.251
H	15.715	-21.147	7.512
H	17.190	-20.802	6.901
H	18.444	-18.675	8.897
N	15.772	-16.927	9.565
C	15.818	-15.751	10.425
C	17.016	-15.892	11.357
O	17.715	-14.903	11.601
C	14.431	-15.531	11.067
C	13.364	-15.320	9.978
C	11.920	-15.019	10.349
O	11.063	-15.886	10.092
O	11.633	-13.885	10.795
H	15.011	-17.632	9.595
H	15.919	-14.929	9.872
H	14.199	-16.333	11.553
H	14.473	-14.724	11.596
H	13.685	-14.557	9.389
H	13.354	-16.156	9.401
N	17.326	-17.142	11.756
C	18.469	-17.440	12.612
C	19.815	-17.064	11.968
O	20.763	-16.707	12.678
C	18.460	-18.913	12.941
H	16.673	-17.873	11.395
H	18.377	-16.917	13.476
H	19.298	-19.343	12.600
H	18.407	-19.040	13.933
H	17.669	-19.353	12.512
N	19.924	-17.211	10.641
C	21.184	-16.973	9.939
C	21.244	-15.589	9.271
O	22.177	-15.340	8.528
C	21.466	-18.069	8.914
C	21.752	-19.396	9.586
H	19.049	-17.504	10.166
H	21.921	-16.993	10.632
H	20.668	-18.171	8.334
H	22.259	-17.805	8.380
N	21.361	-20.493	8.963
O	22.283	-19.419	10.689
H	20.898	-20.413	8.072
H	21.519	-21.391	9.363
N	20.270	-14.711	9.516
C	20.126	-13.467	8.757
C	21.285	-12.503	9.038
O	21.590	-11.642	8.211
C	18.790	-12.819	9.055
H	19.626	-14.985	10.288
H	20.125	-13.686	7.773
H	18.717	-12.630	10.035
H	18.710	-11.960	8.547
H	18.048	-13.433	8.782
N	21.949	-12.643	10.194
C	23.138	-11.850	10.512
C	24.278	-12.128	9.534
O	25.176	-11.300	9.361
C	23.585	-12.061	11.986
C	24.138	-13.427	12.249
C	23.439	-14.559	12.577
C	25.499	-13.844	12.067
C	25.554	-15.226	12.384
C	26.690	-13.173	11.786
N	24.288	-15.630	12.682
C	26.740	-15.947	12.364
C	27.863	-13.889	11.736
C	27.889	-15.255	12.043
H	21.549	-13.356	10.836
H	22.890	-10.877	10.420
H	24.294	-11.403	12.193
H	22.797	-11.936	12.571
H	22.450	-14.597	12.719
H	24.019	-16.561	12.937
H	26.693	-12.185	11.622
H	26.752	-16.925	12.575
H	28.708	-13.428	11.478
H	28.766	-15.737	12.027
N	24.317	-13.304	8.921
C	25.395	-13.610	7.993
C	24.852	-13.688	6.559
O	25.433	-14.308	5.684

C	26.159	-14.847	8.457
C	25.368	-16.147	8.484
C	26.230	-17.352	8.858
C	25.395	-18.533	9.311
N	26.201	-19.634	9.899
H	23.550	-13.951	9.151
H	26.082	-12.880	8.032
H	26.948	-14.975	7.846
H	26.503	-14.671	9.387
H	24.625	-16.065	9.155
H	24.969	-16.308	7.577
H	26.762	-17.622	8.059
H	26.842	-17.088	9.600
H	24.724	-18.214	9.997
H	24.878	-18.891	8.519
H	25.865	-20.512	9.557
H	27.160	-19.519	9.640
H	26.119	-19.611	10.895
N	23.824	-12.901	6.277
C	23.162	-12.926	4.977
C	23.793	-11.964	3.963
O	23.506	-12.132	2.788
C	21.689	-12.615	5.118
O	21.456	-11.271	5.568
H	23.532	-12.270	7.053
H	23.244	-13.864	4.600
H	21.237	-12.729	4.233
H	21.278	-13.240	5.783
H	21.502	-11.250	6.563
N	24.596	-10.975	4.373
C	24.928	-9.898	3.434
C	26.431	-9.677	3.349
O	27.068	-9.318	4.337
C	24.212	-8.575	3.732
C	24.382	-7.539	2.617
O	22.813	-8.814	3.858
H	24.931	-11.035	5.330
H	24.591	-10.161	2.520
H	24.556	-8.207	4.620
H	23.566	-7.533	2.038
H	24.510	-6.632	3.019
H	25.182	-7.772	2.062
H	22.643	-9.637	4.412
N	26.988	-9.888	2.149
C	28.365	-9.507	1.894
C	28.412	-8.100	1.311
O	27.504	-7.712	0.566
C	28.973	-10.498	0.892
C	29.199	-11.932	1.357
C	29.476	-12.838	0.156
C	30.363	-11.995	2.318
H	26.367	-10.330	1.454
H	28.868	-9.506	2.762
H	28.363	-10.534	0.095
H	29.861	-10.131	0.606
H	28.383	-12.257	1.831
H	30.292	-12.515	-0.321
H	29.622	-13.774	0.475
H	28.693	-12.813	-0.464
H	30.175	-11.421	3.118
H	30.505	-12.941	2.619
H	31.195	-11.666	1.866
N	29.566	-7.451	1.499
C	29.829	-6.128	0.968
C	31.156	-6.165	0.254
O	32.135	-6.799	0.675
C	29.801	-5.036	2.043
C	30.831	-5.248	3.159
C	29.964	-3.668	1.426
H	30.264	-7.990	2.068
H	29.119	-5.911	0.301
H	28.896	-5.057	2.483
H	30.354	-5.448	4.013
H	31.424	-6.014	2.917
H	31.377	-4.419	3.264
H	29.697	-2.966	2.089
H	30.921	-3.530	1.163
H	29.383	-3.594	0.613
N	31.209	-5.426	-0.847
C	32.466	-5.297	-1.554
C	32.488	-4.091	-2.477
O	31.651	-3.194	-2.371
H	30.330	-4.983	-1.131
H	33.224	-5.217	-0.885
H	32.636	-6.135	-2.099
N	33.531	-4.028	-3.320
C	33.654	-2.911	-4.238
C	34.200	-3.424	-5.569
O	35.044	-4.304	-5.571
C	34.551	-1.814	-3.666
C	35.793	-2.326	-3.044
C	37.081	-2.377	-3.458
N	35.788	-2.793	-1.750
C	37.037	-3.145	-1.404
N	37.840	-2.910	-2.435
H	34.195	-4.807	-3.252
H	32.747	-2.525	-4.401
H	34.799	-1.179	-4.408
H	34.031	-1.300	-2.973
H	34.992	-2.863	-1.157
H	37.428	-2.082	-4.349
H	37.307	-3.515	-0.525
N	33.680	-2.909	-6.673
C	34.124	-3.299	-8.004
C	34.430	-2.019	-8.792
O	33.648	-1.078	-8.727
C	33.049	-4.106	-8.733
C	32.814	-5.556	-8.287
O	33.609	-6.100	-7.493
O	31.770	-6.119	-8.682
H	32.927	-2.202	-6.508
H	34.985	-3.788	-7.903
H	32.171	-3.607	-8.641
H	33.285	-4.119	-9.719
N	35.524	-2.013	-9.566
C	35.763	-0.927	-10.500
C	35.706	-1.488	-11.929
O	36.363	-2.488	-12.181
C	37.134	-0.270	-10.274
C	37.313	0.935	-11.165
O	37.238	0.176	-8.925
H	36.152	-2.820	-9.438
H	35.037	-0.250	-10.405
H	37.863	-0.967	-10.439
H	36.477	1.099	-11.698
H	37.508	1.750	-10.610
H	38.077	0.786	-11.800
H	37.066	-0.584	-8.287
N	34.918	-0.840	-12.794
C	34.666	-1.310	-14.154
C	35.372	-0.392	-15.147
O	35.298	0.805	-15.001
C	33.176	-1.339	-14.449
C	32.376	-2.317	-13.623
C	31.826	-1.949	-12.393
C	32.197	-3.626	-14.057
C	31.165	-2.908	-11.636
C	31.518	-4.573	-13.290
C	30.970	-4.184	-12.094
H	34.496	0.038	-12.408
H	35.094	-2.210	-14.237
H	32.809	-0.430	-14.277
H	33.055	-1.585	-15.405
H	31.904	-1.025	-12.067
H	32.568	-3.901	-14.953
H	30.820	-2.639	-10.710
H	31.442	-5.520	-13.621
H	30.425	-4.833	-11.553
N	36.065	-0.975	-16.128
C	36.676	-0.196	-17.193
C	36.175	-0.729	-18.536
O	35.573	-1.790	-18.577
C	38.212	-0.227	-17.091
C	38.731	0.335	-15.788
O	38.667	-1.572	-17.145
H	36.125	-2.008	-16.061
H	36.385	0.755	-17.106
H	38.602	0.279	-17.888
H	39.545	0.900	-15.955
H	38.027	0.903	-15.352
H	38.978	-0.412	-15.164
H	37.951	-2.203	-16.821
N	36.435	0.030	-19.619
C	36.005	-0.344	-20.958
C	36.966	-1.361	-21.558
O	36.663	-1.933	-22.589
C	35.985	0.857	-21.905
C	35.003	1.972	-21.582
C	33.568	1.537	-21.600
C	33.054	1.160	-22.972
N	31.637	0.723	-22.898
H	36.968	0.900	-19.406
H	35.090	-0.757	-20.905
H	36.914	1.254	-21.923
H	35.778	0.516	-22.834
H	35.227	2.341	-20.666
H	35.133	2.719	-22.252
H	33.458	0.739	-20.992
H	32.992	2.284	-21.242
H	33.123	1.953	-23.577
H	33.610	0.413	-23.337
H	31.423	0.150	-23.689
H	31.493	0.199	-22.058
H	31.040	1.526	-22.897
N	38.110	-1.591	-20.918
C	39.095	-2.549	-21.405
C	39.334	-3.613	-20.335
O	39.170	-3.311	-19.156
C	40.412	-1.836	-21.782
C	40.161	-0.570	-22.604
C	41.271	-1.517	-20.564
H	38.238	-1.032	-20.048
H	38.720	-3.004	-22.213
H	40.938	-2.457	-22.361
H	40.830	0.126	-22.350
H	40.251	-0.784	-23.575
H	39.240	-0.233	-22.418
H	41.812	-0.697	-20.749
H	40.678	-1.360	-19.775
H	41.880	-2.288	-20.380
N	39.694	-4.841	-20.752
C	40.275	-5.870	-19.885
C	39.356	-7.094	-19.819
O	39.153	-7.712	-20.877
C	40.546	-5.371	-18.458
C	40.911	-6.444	-17.439
C	42.003	-7.373	-17.918
C	42.661	-8.144	-16.794
N	43.896	-8.822	-17.256
O	38.762	-7.601	-18.900
H	39.517	-5.000	-21.778
H	41.160	-6.153	-20.265
H	41.300	-4.709	-18.500
H	39.720	-4.898	-18.134
H	41.214	-5.992	-16.590
H	40.087	-6.986	-17.231
H	41.609	-8.030	-18.566
H	42.706	-6.833	-18.387
H	42.890	-7.509	-16.053
H	42.017	-8.831	-16.448
H	43.923	-9.753	-16.890
H	43.904	-8.857	-18.256
H	44.696	-8.315	-16.934
N	29.761	11.650	28.317
C	28.947	10.417	28.505
C	29.881	9.226	28.675
O	29.662	8.164	28.096
C	28.020	10.122	27.316
C	27.894	11.232	26.284
O	27.255	12.267	26.589
O	28.449	11.054	25.187
H	30.754	11.681	28.306
H	28.449	10.534	29.360
H	28.367	9.297	26.857
H	27.106	9.923	27.684

N	30.950	9.420	29.447
C	31.796	8.323	29.899
C	30.989	7.299	30.710
O	31.133	6.083	30.540
C	32.894	8.918	30.778
C	33.880	7.907	31.294
C	35.048	8.577	31.992
O	35.944	7.851	32.477
O	35.060	9.825	32.041
H	31.125	10.418	29.696
H	32.211	7.862	29.106
H	33.387	9.574	30.232
H	32.457	9.342	31.553
H	33.432	7.337	31.953
H	34.242	7.402	30.537
N	30.203	7.832	31.656
C	29.293	7.069	32.499
C	28.133	6.511	31.673
O	27.693	5.385	31.931
C	28.777	7.956	33.610
H	30.306	8.881	31.737
H	29.794	6.297	32.906
H	28.067	8.566	33.255
H	28.390	7.393	34.341
H	29.527	8.506	33.981
N	27.636	7.320	30.713
C	26.574	6.912	29.802
C	26.962	5.658	29.024
O	26.165	4.728	28.913
H	28.085	8.263	30.687
H	25.732	6.729	30.326
H	26.382	7.659	29.152
N	28.206	5.624	28.505
C	28.658	4.478	27.724
C	29.166	3.337	28.614
O	28.958	2.189	28.307
C	29.750	4.893	26.715
C	29.181	5.821	25.646
C	30.384	3.643	26.135
C	30.243	6.498	24.787
H	28.781	6.454	28.707
H	27.846	4.123	27.243
H	30.454	5.389	27.216
H	28.579	5.293	25.051
H	28.642	6.531	26.097
H	31.134	3.898	25.525
H	30.739	3.072	26.874
H	29.700	3.128	25.619
H	30.779	5.798	24.321
H	29.794	7.084	24.117
H	30.836	7.045	25.373
N	29.842	3.638	29.725
C	30.455	2.603	30.532
C	29.334	1.708	31.069
O	28.286	2.218	31.481
C	31.319	3.174	31.674
C	31.703	2.107	32.672
O	32.542	3.752	31.199
H	29.884	4.649	29.946
H	31.046	2.047	29.957
H	30.784	3.914	32.153
H	32.210	2.516	33.437
H	30.880	1.658	33.032
H	32.283	1.415	32.232
H	33.196	3.856	31.955
N	29.541	0.393	30.969
C	28.609	-0.574	31.520
C	28.364	-1.745	30.593
O	29.216	-2.101	29.779
H	30.415	0.132	30.469
H	28.967	-0.918	32.407
H	27.725	-0.113	31.718
N	27.185	-2.353	30.760
C	26.883	-3.615	30.111
C	25.739	-3.374	29.125
O	24.732	-2.738	29.462
C	26.618	-4.744	31.128
C	26.113	-5.993	30.447
O	27.806	-5.140	31.832
H	26.524	-1.849	31.382
H	27.680	-3.914	29.595
H	25.936	-4.407	31.824
H	26.885	-6.580	30.183
H	25.513	-6.507	31.068
H	25.594	-5.751	29.622
H	28.580	-5.214	31.195
N	25.935	-3.876	27.890
C	25.032	-3.639	26.781
C	24.658	-4.960	26.147
O	25.473	-5.880	26.095
C	25.698	-2.730	25.736
C	25.946	-1.337	26.190
C	27.056	-0.887	26.829
C	25.054	-0.219	26.077
C	25.697	0.872	26.689
C	23.771	-0.039	25.548
N	26.921	0.440	27.104
C	25.173	2.154	26.673
C	23.231	1.222	25.576
C	23.910	2.294	26.157
H	26.806	-4.456	27.811
H	24.195	-3.228	27.148
H	26.579	-3.128	25.486
H	25.107	-2.686	24.933
H	27.852	-1.445	27.063
H	27.617	1.018	27.547
H	23.266	-0.804	25.162
H	25.697	2.930	27.023
H	22.328	1.376	25.170
H	23.456	3.189	26.195
N	23.444	-5.011	25.582
C	22.946	-6.216	24.945
C	22.401	-5.884	23.561
O	21.706	-4.886	23.424
C	21.836	-6.816	25.836
C	22.106	-6.843	27.336
C	22.819	-7.887	27.909
C	21.604	-5.865	28.192
C	23.060	-7.955	29.275
C	21.848	-5.901	29.567
C	22.566	-6.961	30.116
O	22.846	-7.057	31.472
H	22.906	-4.124	25.643
H	23.710	-6.858	24.833
H	20.996	-6.285	25.682
H	21.676	-7.762	25.534
H	23.175	-8.623	27.317
H	21.056	-5.117	27.816
H	23.588	-8.715	29.657
H	21.508	-5.169	30.155
H	23.335	-6.237	31.758
N	22.634	-6.750	22.564
C	22.114	-6.502	21.222
C	20.868	-7.349	20.972
O	20.398	-8.070	21.860
C	23.184	-6.578	20.113
C	23.705	-7.946	19.701
H	23.193	-7.575	22.825
H	21.827	-5.541	21.187
H	22.802	-6.121	19.285
H	23.978	-6.013	20.413
N	24.792	-7.954	18.938
O	23.167	-8.987	20.060
H	25.224	-7.092	18.672
H	25.181	-8.822	18.629
N	20.311	-7.213	19.760
C	19.074	-7.895	19.395
C	19.277	-9.419	19.372
O	18.309	-10.154	19.498
C	18.517	-7.288	18.093
C	19.333	-7.529	16.806
C	20.651	-6.788	16.632
H	20.834	-6.587	19.116
H	18.385	-7.693	20.098
H	17.589	-7.658	17.937
H	18.432	-6.287	18.215
H	19.520	-8.532	16.741
H	18.731	-7.303	16.011
N	21.381	-7.167	15.591
O	21.035	-5.898	17.400
H	22.276	-6.708	15.395
H	21.045	-7.914	14.993
N	20.522	-9.905	19.202
C	20.802	-11.323	19.029
C	21.146	-11.989	20.366
O	21.555	-13.147	20.368
C	22.002	-11.508	18.092
C	21.922	-10.914	16.681
C	23.239	-11.169	15.931
C	20.713	-11.454	15.913
H	21.264	-9.168	19.206
H	19.981	-11.773	18.657
H	22.804	-11.098	18.540
H	22.159	-12.495	17.990
H	21.769	-9.933	16.747
H	23.395	-12.153	15.869
H	23.175	-10.778	15.015
H	23.988	-10.739	16.430
H	19.876	-11.217	16.405
H	20.693	-11.047	15.001
H	20.788	-12.447	15.836
N	21.124	-11.223	21.460
C	21.522	-11.717	22.763
C	23.036	-11.782	22.981
O	23.524	-12.563	23.793
H	20.797	-10.246	21.289
H	21.113	-11.126	23.490
H	21.131	-12.651	22.906
N	23.810	-10.999	22.231
C	25.219	-10.833	22.537
C	25.362	-9.795	23.655
O	24.515	-8.893	23.790
C	26.016	-10.428	21.280
O	25.990	-11.435	20.263
H	23.330	-10.538	21.440
H	25.574	-11.700	22.882
H	25.622	-9.588	20.909
H	26.966	-10.265	21.544
H	26.702	-11.240	19.593
N	26.462	-9.928	24.420
C	26.810	-9.068	25.546
C	28.102	-8.272	25.307
O	29.165	-8.868	25.113
C	27.077	-9.917	26.802
C	27.212	-9.077	28.047
O	26.009	-10.858	26.902
H	27.073	-10.742	24.120
H	26.058	-8.405	25.690
H	27.942	-10.424	26.675
H	27.884	-8.344	27.900
H	26.328	-8.662	28.283
H	27.517	-9.645	28.817
H	26.033	-11.502	26.129
N	28.006	-6.950	25.490
C	29.098	-6.002	25.298
C	29.359	-5.298	26.628
O	28.536	-4.505	27.063
C	28.636	-5.076	24.165
C	29.407	-3.819	23.886
C	30.777	-3.857	23.657
C	28.724	-2.628	23.694
C	31.472	-2.698	23.334
C	29.412	-1.472	23.392
C	30.787	-1.505	23.211
H	27.039	-6.643	25.795
H	29.915	-6.533	25.089
H	28.628	-5.625	23.315
H	27.681	-4.811	24.367
H	31.268	-4.719	23.723
H	27.728	-2.611	23.775
H	32.466	-2.738	23.194
H	28.909	-0.594	23.304
H	31.284	-0.664	22.990
N	30.522	-5.549	27.233
C	30.908	-4.931	28.491

C	32.058	-3.971	28.216
O	33.070	-4.392	27.663
C	31.339	-6.021	29.487
C	30.282	-7.131	29.614
C	31.690	-5.403	30.839
C	30.867	-8.461	30.093
H	31.130	-6.230	26.720
H	30.136	-4.374	28.782
H	32.180	-6.439	29.145
H	29.589	-6.836	30.260
H	29.868	-7.272	28.722
H	32.052	-6.110	31.442
H	32.376	-4.691	30.709
H	30.869	-5.006	31.245
H	31.282	-8.327	30.989
H	30.134	-9.133	30.153
H	31.555	-8.765	29.440
N	31.872	-2.702	28.548
C	32.788	-1.675	28.085
C	33.071	-0.693	29.212
O	32.170	-0.291	29.927
C	32.231	-0.993	26.802
C	31.017	-0.119	27.029
C	33.280	-0.194	26.056
H	31.048	-2.516	29.143
H	33.654	-2.114	27.832
H	31.954	-1.733	26.171
H	31.302	0.766	27.402
H	30.539	0.028	26.161
H	30.395	-0.564	27.676
H	33.818	-0.808	25.478
H	32.831	0.494	25.485
H	33.883	0.260	26.713
N	34.328	-0.255	29.288
C	34.679	0.954	30.000
C	35.103	2.041	29.009
O	35.907	1.788	28.115
C	35.795	0.703	31.023
C	36.123	1.950	31.820
O	35.397	-0.357	31.895
H	35.022	-0.857	28.794
H	33.873	1.286	30.499
H	36.636	0.410	30.528
H	36.644	2.593	31.252
H	35.277	2.395	32.124
H	36.671	1.708	32.625
H	34.959	-1.097	31.372
N	34.532	3.232	29.176
C	34.935	4.418	28.438
C	35.887	5.254	29.285
O	35.450	5.871	30.256
C	33.729	5.228	28.121
H	33.763	3.239	29.890
H	35.414	4.136	27.603
H	33.201	4.798	27.381
H	33.137	5.309	28.930
H	33.994	6.153	27.827
N	37.155	5.333	28.874
C	38.117	6.191	29.545
C	37.929	7.674	29.226
O	37.325	8.075	28.215
H	37.382	4.741	28.054
H	38.044	6.059	30.550
H	39.059	5.915	29.280
N	38.496	8.518	30.104
C	38.411	9.967	29.953
C	39.093	10.425	28.659
O	38.750	11.465	28.102
C	39.043	10.648	31.155
H	38.993	8.049	30.888
H	37.441	10.235	29.912
H	40.012	10.405	31.207
H	38.954	11.640	31.064
H	38.582	10.350	31.991
N	40.083	9.636	28.231
C	40.944	9.920	27.100
C	40.319	9.484	25.771
O	40.960	9.624	24.738
C	42.256	9.141	27.258
C	42.120	7.612	27.327
O	41.050	7.103	27.775
O	43.091	6.921	26.914
H	40.185	8.753	28.814
H	41.060	10.924	27.050
H	42.846	9.366	26.477
H	42.702	9.453	28.101
N	39.109	8.915	25.790
C	38.454	8.471	24.561
C	38.651	6.982	24.282
O	38.212	6.482	23.249
H	38.688	8.820	26.732
H	37.460	8.668	24.622
H	38.815	9.005	23.778
N	39.268	6.245	25.209
C	39.451	4.816	25.028
C	38.146	4.082	25.326
O	37.345	4.516	26.146
C	40.554	4.291	25.891
H	39.591	6.771	26.039
H	39.707	4.636	24.074
H	41.114	3.634	25.378
H	41.143	5.044	26.199
H	40.174	3.829	26.698
N	37.942	3.015	24.573
C	37.011	1.950	24.940
C	37.808	0.686	25.136
O	38.638	0.300	24.301
C	35.970	1.681	23.827
C	35.060	2.819	23.402
C	34.170	2.359	22.271
C	34.221	3.397	24.545
H	38.504	2.994	23.689
H	36.574	2.195	25.801
H	36.480	1.377	23.015
H	35.389	0.926	24.144
H	35.618	3.588	23.097
H	33.611	1.584	22.572
H	33.567	3.108	21.987
H	34.731	2.075	21.491
H	34.829	3.750	25.255
H	33.650	4.137	24.193
H	33.642	2.677	24.926
N	37.482	-0.028	26.224
C	38.140	-1.273	26.562
C	37.067	-2.227	27.098
O	36.089	-1.777	27.673
C	39.267	-1.006	27.583
C	40.378	-0.131	27.043
O	38.657	-0.384	28.712
H	36.718	0.396	26.795
H	38.525	-1.673	25.738
H	39.657	-1.891	27.873
H	41.271	-0.538	27.256
H	40.291	-0.040	26.047
H	40.329	0.782	27.458
H	37.872	0.177	28.425
N	37.201	-3.531	26.864
C	36.249	-4.453	27.457
C	36.265	-5.826	26.821
O	37.313	-6.346	26.395
H	37.984	-3.801	26.268
H	36.445	-4.543	28.453
H	35.312	-4.060	27.383
N	35.062	-6.422	26.854
C	34.835	-7.767	26.402
C	33.502	-7.837	25.673
O	32.521	-7.181	26.029
C	34.827	-8.792	27.562
C	36.081	-8.728	28.415
O	33.667	-8.479	28.330
H	34.305	-5.804	27.244
H	35.558	-8.024	25.755
H	34.721	-9.712	27.176
H	36.276	-7.776	28.665
H	35.953	-9.266	29.252
H	36.861	-9.098	27.904
H	33.432	-7.505	28.234
N	33.503	-8.724	24.691
C	32.325	-9.048	23.920
C	32.075	-10.559	24.015
O	32.967	-11.356	23.766
C	32.523	-8.636	22.456
C	31.255	-8.484	21.655
C	30.687	-9.547	20.976
C	30.647	-7.258	21.563
C	29.501	-9.400	20.277
C	29.470	-7.088	20.871
C	28.913	-8.156	20.198
O	27.727	-7.896	19.575
H	34.439	-9.173	24.522
H	31.536	-8.583	24.320
H	33.010	-7.760	22.444
H	33.092	-9.332	22.013
H	31.140	-10.443	20.987
H	31.069	-6.462	22.012
H	29.075	-10.191	19.835
H	29.017	-6.194	20.853
H	27.105	-8.668	19.708
N	30.810	-10.916	24.230
C	30.343	-12.287	24.074
C	29.263	-12.388	22.993
O	28.173	-11.861	23.217
C	29.766	-12.723	25.416
C	28.991	-14.029	25.344
C	29.252	-14.876	26.575
O	29.431	-16.101	26.408
O	29.372	-14.286	27.685
H	30.185	-10.129	24.519
H	31.116	-12.885	23.827
H	30.515	-12.856	26.037
H	29.145	-12.026	25.719
H	28.039	-13.821	25.323
H	29.301	-14.530	24.568
N	29.565	-13.120	21.897
C	28.706	-13.285	20.719
C	27.760	-14.473	20.917
O	28.210	-15.572	21.242
C	29.511	-13.480	19.441
O	28.665	-13.767	18.313
H	30.517	-13.583	21.968
H	28.147	-12.459	20.624
H	30.023	-12.648	19.244
H	30.140	-14.245	19.562
H	28.789	-14.720	18.051
N	26.461	-14.259	20.663
C	25.473	-15.329	20.750
C	25.394	-16.139	19.447
O	24.701	-17.152	19.414
C	24.139	-14.759	21.126
H	26.229	-13.281	20.404
H	25.735	-15.956	21.496
H	24.097	-14.600	22.116
H	23.990	-13.887	20.653
H	23.406	-15.395	20.870
N	26.123	-15.757	18.387
C	26.120	-16.545	17.155
C	27.537	-16.684	16.599
O	28.484	-16.026	17.054
C	25.147	-15.970	16.098
C	23.698	-16.340	16.393
C	25.296	-14.459	15.922
H	26.663	-14.891	18.518
H	25.782	-17.466	17.373
H	25.381	-16.383	15.218
H	23.092	-15.647	16.003
H	23.492	-17.230	15.987
H	23.561	-16.385	17.382
H	25.786	-14.276	15.071
H	24.389	-14.043	15.887
H	25.809	-14.089	16.695
N	27.657	-17.603	15.628
C	28.857	-17.768	14.821
C	29.940	-18.611	15.496
O	29.696	-19.285	16.502
H	26.799	-18.196	15.504

H	28.610	-18.200	13.933
H	29.243	-16.852	14.600
N	31.152	-18.571	14.914
C	32.299	-19.321	15.422
C	32.926	-18.551	16.599
O	34.026	-18.001	16.500
C	33.253	-19.665	14.268
C	34.385	-20.610	14.641
H	31.197	-17.958	14.071
H	31.954	-20.181	15.793
H	32.716	-20.088	13.534
H	33.653	-18.809	13.928
N	35.148	-21.023	13.645
O	34.589	-20.952	15.808
H	34.974	-20.712	12.709
H	35.910	-21.653	13.821
N	32.188	-18.492	17.717
C	32.572	-17.717	18.890
C	32.160	-18.477	20.152
O	31.082	-19.068	20.178
C	31.914	-16.354	18.860
H	31.306	-19.057	17.669
H	33.566	-17.605	18.897
H	31.168	-16.324	19.527
H	32.587	-15.648	19.087
H	31.546	-16.176	17.946
N	32.995	-18.445	21.197
C	32.566	-18.954	22.497
C	33.336	-18.287	23.636
O	34.570	-18.259	23.667
C	32.647	-20.489	22.600
C	34.033	-21.061	22.882
C	34.085	-22.577	23.020
O	33.861	-23.070	24.142
O	34.324	-23.265	21.997
H	33.923	-18.047	21.008
H	31.579	-18.786	22.618
H	32.053	-20.768	23.341
H	32.340	-20.861	21.736
H	34.627	-20.812	22.130
H	34.359	-20.676	23.734
N	32.542	-17.841	24.610
C	33.012	-17.082	25.746
C	33.338	-15.665	25.260
O	32.797	-15.177	24.256
C	34.173	-17.797	26.464
O	34.253	-17.414	27.843
H	31.527	-18.097	24.472
H	32.275	-17.009	26.411
H	34.025	-18.779	26.413
H	35.026	-17.552	26.015
H	33.655	-16.632	27.998
N	34.177	-14.988	26.031
C	34.359	-13.561	25.895
C	35.589	-13.334	25.028
O	36.525	-14.153	24.994
C	34.479	-12.892	27.270
C	33.480	-13.379	28.323
C	33.333	-12.506	29.603
N	32.233	-11.976	28.786
C	30.951	-11.970	29.164
N	30.015	-11.576	28.327
N	30.603	-12.384	30.372
H	34.685	-15.570	26.733
H	33.568	-13.168	25.434
H	35.399	-13.062	27.621
H	34.343	-11.909	27.152
H	32.558	-13.449	27.905
H	33.742	-14.310	28.629
H	32.998	-13.039	30.231
H	34.010	-11.929	29.589
H	32.460	-11.594	27.885
H	30.233	-11.295	27.394
H	29.050	-11.556	28.627
H	31.314	-12.723	30.998
H	29.651	-12.363	30.661
N	35.556	-12.190	24.331
C	36.681	-11.746	23.529
C	37.025	-10.311	23.919
O	36.147	-9.532	24.295
C	36.350	-11.880	22.039
C	36.068	-13.292	21.579
C	34.846	-13.908	21.815
C	37.029	-14.008	20.895
C	34.591	-15.197	21.375
C	36.806	-15.319	20.502
C	35.577	-15.911	20.732
O	35.404	-17.213	20.267
H	34.664	-11.662	24.419
H	37.471	-12.321	23.738
H	35.543	-11.321	21.848
H	37.127	-11.531	21.515
H	34.131	-13.411	22.315
H	37.906	-13.576	20.675
H	33.686	-15.607	21.525
H	37.536	-15.838	20.051
H	35.322	-17.189	19.275
N	38.320	-10.007	23.839
C	38.821	-8.669	24.061
C	38.281	-7.740	22.964
O	38.317	-8.107	21.787
C	40.359	-8.638	24.093
C	40.871	-7.190	24.111
C	40.916	-9.444	25.289
H	38.934	-10.821	23.602
H	38.472	-8.335	24.937
H	40.692	-9.047	23.250
H	41.070	-6.902	23.177
H	40.169	-6.600	24.504
H	41.700	-7.143	24.663
H	41.505	-8.848	25.827
H	40.151	-9.764	25.841
H	41.436	-10.217	24.937
N	37.790	-6.574	23.387
C	37.518	-5.479	22.458
C	38.290	-4.231	22.863
O	38.648	-4.000	24.021
C	36.015	-5.200	22.384
C	35.397	-4.395	23.508
C	35.481	-2.883	23.280
C	33.968	-4.846	23.698
H	37.623	-6.515	24.407
H	37.819	-5.756	21.540
H	35.843	-4.706	21.527
H	35.546	-6.085	22.350
H	35.866	-4.618	24.375
H	34.999	-2.649	22.437
H	35.059	-2.409	24.051
H	36.440	-2.614	23.204
H	33.947	-5.821	23.930
H	33.542	-4.323	24.440
H	33.449	-4.699	22.853
N	38.617	-3.452	21.834
C	39.158	-2.131	22.018
C	38.536	-1.213	20.962
O	38.225	-1.643	19.848
C	40.686	-2.183	21.982
C	41.282	-2.537	20.633
O	41.107	-0.880	22.344
H	38.446	-3.885	20.899
H	38.895	-1.788	22.917
H	41.008	-2.833	22.679
H	40.961	-1.890	19.936
H	42.283	-2.495	20.679
H	41.006	-3.465	20.369
H	40.430	-0.440	22.948
N	38.392	0.040	21.332
C	37.896	1.069	20.435
C	38.144	2.484	20.957
O	38.977	2.710	21.850
H	38.664	0.236	22.325
H	38.341	0.971	19.528
H	36.899	0.942	20.292
N	37.408	3.423	20.370
C	37.535	4.839	20.618
C	36.144	5.443	20.577
O	35.237	4.998	19.853
C	38.466	5.504	19.590
C	39.888	4.967	19.495
C	40.796	5.337	20.645
N	40.945	6.794	20.756
C	41.369	7.423	21.846
N	41.868	6.707	22.841
N	41.269	8.746	21.936
H	36.700	3.026	19.685
H	37.920	4.983	21.527
H	38.039	5.411	18.682
H	38.521	6.485	19.813
H	39.854	3.956	19.434
H	40.311	5.307	18.639
H	40.399	4.987	21.486
H	41.689	4.932	20.484
H	40.709	7.349	19.950
H	41.956	5.720	22.796
H	42.175	7.183	23.685
H	40.889	9.264	21.165
H	41.569	9.218	22.759
N	35.975	6.518	21.344
C	34.754	7.279	21.271
C	34.987	8.774	21.297
O	36.144	9.241	21.499
C	33.758	6.803	22.332
C	34.081	7.315	23.719
C	35.149	6.835	24.442
C	33.314	8.324	24.273
C	35.434	7.330	25.715
C	33.581	8.821	25.539
C	34.635	8.313	26.255
O	34.827	8.840	27.501
H	36.768	6.740	21.966
H	34.325	7.120	20.382
H	32.855	7.130	22.081
H	33.777	5.810	22.355
H	35.737	6.119	24.059
H	32.549	8.706	23.750
H	36.216	6.970	26.226
H	33.006	9.545	25.924
H	35.735	8.585	27.833
N	33.970	9.609	20.942
C	34.091	11.069	20.950
C	33.895	11.594	22.358
O	32.739	11.794	22.803
C	33.074	11.661	19.957
C	33.207	13.141	19.783
O	34.182	13.778	20.276
O	32.533	13.703	18.879
H	33.024	9.194	20.778
H	35.105	11.360	20.595
H	33.222	11.182	18.964
H	32.038	11.428	20.288
N	34.971	11.801	23.173
C	34.842	12.208	24.596
C	34.448	13.673	24.781
O	34.331	14.120	25.928
C	36.115	11.930	25.311
O	37.188	12.603	24.674
H	35.906	11.542	22.789
H	34.111	11.638	25.010
H	36.055	12.253	26.251
H	36.306	10.952	25.298
H	37.995	12.256	25.152
N	34.242	14.518	23.717
C	33.527	15.808	23.818
C	32.528	15.947	22.671
O	32.890	16.538	21.609
C	34.511	16.981	23.845
H	34.352	14.126	22.759
H	32.970	15.870	24.777
H	35.143	16.979	22.935
H	33.949	17.939	23.875
H	35.132	16.933	24.763
N	31.312	15.311	22.715
C	30.357	15.257	21.604
C	29.769	16.608	21.263
O	30.170	17.658	21.837

C	29.266	14.293	22.082
C	29.430	14.235	23.594
C	30.868	14.477	23.821
H	30.848	14.838	20.703
H	28.231	14.597	21.808
H	29.457	13.282	21.656
H	28.883	14.970	23.999
H	29.104	13.353	23.940
H	31.028	14.868	24.785
H	31.387	13.556	23.881
N	28.838	16.722	20.184
C	28.061	17.951	19.990
C	26.966	18.085	21.035
O	26.238	17.101	21.320
C	27.484	17.979	18.575
H	28.451	15.831	19.802
H	28.741	18.826	20.086
H	26.807	17.118	18.426
H	26.907	18.914	18.405
H	28.299	17.922	17.826
N	26.578	19.387	21.472
C	25.488	19.658	22.444
C	24.321	20.304	21.707
O	24.018	21.477	21.897
C	26.002	20.629	23.527
C	27.016	20.026	24.480
O	26.578	21.759	22.862
H	26.921	20.183	20.890
H	25.170	18.818	22.864
H	25.215	20.949	24.078
H	27.637	20.739	24.816
H	26.545	19.608	25.261
H	27.553	19.322	24.008
H	27.526	21.555	22.659
N	23.888	19.693	20.563
C	22.664	20.047	19.812
C	21.697	18.859	19.747
O	20.766	18.847	18.896
C	23.042	20.578	18.407
C	23.776	19.602	17.517
O	24.125	18.465	17.934
O	24.124	19.946	16.354
H	24.392	18.829	20.260
H	22.117	20.862	20.335
H	22.118	20.912	17.887
H	23.685	21.477	18.532
N	21.775	17.857	20.686
C	20.985	16.641	20.634
C	21.626	15.504	19.826
O	21.037	14.436	19.750
H	22.502	18.037	21.421
H	20.811	16.307	21.583
H	20.069	16.847	20.234
N	22.803	15.692	19.165
C	23.387	14.626	18.356
C	24.000	13.542	19.202
O	24.292	13.682	20.430
C	24.395	15.172	17.336
O	23.718	15.744	16.244
H	23.345	16.567	19.323
H	22.570	14.141	17.779
H	25.074	15.903	17.821
H	25.038	14.358	16.945
H	23.531	16.689	16.518
N	24.253	12.315	18.552
C	24.598	11.109	19.260
C	26.084	10.872	19.178
O	26.689	10.946	18.074
H	24.132	12.247	17.523
H	24.246	11.101	20.315
H	24.090	10.266	18.745
N	26.748	10.425	20.271
C	28.216	10.334	20.412
C	28.844	9.321	19.475
O	28.911	8.107	19.806
C	28.516	10.009	21.878
C	29.994	9.922	22.177
O	27.909	11.024	22.620
H	26.181	10.431	21.151
H	28.656	11.332	20.203
H	28.047	9.047	22.140
H	30.391	10.846	22.218
H	30.142	9.472	23.065
H	30.550	9.323	21.430
H	28.155	10.949	23.595
N	29.535	9.750	18.303
C	30.119	8.788	17.354
C	31.196	7.921	17.995
O	32.217	8.463	18.501
C	30.699	9.537	16.172
H	29.378	10.740	18.014
H	29.308	8.135	16.967
H	31.462	10.256	16.542
H	31.169	8.820	15.466
H	29.893	10.084	15.640
N	31.116	6.556	17.937
C	32.133	5.656	18.509
C	32.517	4.526	17.561
O	31.925	4.382	16.459
C	31.656	5.142	19.899
C	30.198	4.671	20.024
C	29.953	3.410	19.200
C	29.869	4.407	21.499
H	30.265	6.125	17.529
H	33.077	6.219	18.665
H	32.283	4.309	20.273
H	31.768	5.994	20.591
H	29.512	5.464	19.671
H	30.725	2.647	19.423
H	28.953	2.993	19.419
H	29.978	3.655	18.124
H	29.978	5.263	22.006
H	28.915	4.110	21.550
H	30.457	3.705	21.903
N	33.495	3.643	17.911
C	33.543	2.277	17.388
C	34.548	1.355	18.072
O	35.291	1.804	18.962
H	34.030	3.867	18.757
H	32.543	1.833	17.559
H	33.753	2.299	16.299
N	34.482	0.054	17.757
C	35.342	-0.902	18.415
C	35.554	-2.078	17.487
O	34.832	-2.231	16.503
C	34.803	-1.258	19.809
C	33.527	-2.065	19.804
C	33.456	-3.419	19.764
C	32.167	-1.605	19.832
C	31.340	-2.755	19.828
C	31.542	-0.375	19.864
N	32.166	-3.836	19.829
C	29.953	-2.695	19.846
C	30.164	-0.313	19.899
C	29.376	-1.461	19.914
H	33.788	-0.182	17.032
H	36.231	-0.469	18.565
H	35.487	-1.797	20.291
H	34.614	-0.416	20.306
H	34.254	-4.031	19.695
H	31.862	-4.795	19.872
H	32.077	0.477	19.863
H	29.408	-3.540	19.809
H	29.722	0.588	19.913
H	28.386	-1.371	19.975
N	36.585	-2.851	17.830
C	37.004	-4.048	17.153
C	37.073	-5.210	18.136
O	37.618	-5.061	19.236
C	38.395	-3.891	16.540
C	38.808	-5.077	15.696
O	38.422	-2.726	15.714
H	37.096	-2.480	18.697
H	36.328	-4.280	16.444
H	39.077	-3.765	17.290
H	38.104	-5.792	15.739
H	38.927	-4.795	14.740
H	39.675	-5.454	16.033
H	39.272	-2.696	15.176
N	36.579	-6.354	17.670
C	36.920	-7.662	18.227
C	37.577	-8.529	17.168
O	37.028	-8.725	16.079
C	35.650	-8.361	18.721
C	35.926	-9.811	19.110
C	35.022	-7.572	19.835
H	35.919	-6.237	16.863
H	37.580	-7.521	18.970
H	34.998	-8.386	17.965
H	35.774	-10.402	18.318
H	36.873	-9.898	19.415
H	35.310	-10.079	19.850
H	35.081	-8.089	20.692
H	35.502	-6.698	19.944
H	34.058	-7.394	19.624
N	38.754	-9.074	17.495
C	39.309	-10.179	16.740
C	38.861	-11.468	17.443
O	38.925	-11.551	18.665
C	40.801	-10.085	16.673
H	39.216	-8.641	18.316
H	38.933	-10.177	15.823
H	41.106	-9.183	16.990
H	41.219	-10.789	17.254
H	41.115	-10.218	15.729
N	38.410	-12.444	16.647
C	37.682	-13.605	17.119
C	38.661	-14.733	17.458
O	38.552	-15.858	16.971
C	36.625	-14.021	16.101
C	35.558	-12.974	15.968
C	35.406	-12.129	14.917
C	34.553	-12.617	16.920
C	33.829	-11.535	16.375
C	34.188	-13.068	18.194
N	34.360	-11.281	15.137
C	32.755	-10.946	17.039
C	33.150	-12.460	18.859
C	32.423	-11.422	18.286
H	38.637	-12.288	15.628
H	37.211	-13.355	17.968
H	37.047	-14.124	15.219
H	36.189	-14.848	16.406
H	35.980	-12.126	14.094
H	34.028	-10.582	14.500
H	34.681	-13.833	18.620
H	32.244	-10.192	16.607
H	32.914	-12.776	19.782
H	31.659	-11.024	18.783
N	39.609	-14.383	18.319
C	40.544	-15.319	18.900
C	40.537	-15.110	20.408
O	40.565	-13.989	20.912
C	41.940	-15.098	18.322
C	43.016	-16.012	18.882
C	44.392	-15.772	18.323
C	45.467	-16.547	19.062
N	45.467	-16.246	20.521
H	39.618	-13.353	18.542
H	40.247	-16.250	18.705
H	41.894	-15.239	17.331
H	42.207	-14.149	18.505
H	43.045	-15.896	19.890
H	42.750	-16.974	18.700
H	44.409	-16.051	17.361
H	44.608	-14.796	18.387
H	45.310	-17.530	18.930
H	46.364	-16.309	18.678
H	45.440	-17.102	21.035
H	46.297	-15.740	20.754
H	44.664	-15.695	20.745
N	40.419	-16.222	21.15
C	40.626	-16.196	22.589
C	41.336	-17.513	22.960
O	41.916	-18.193	22.101

C	39.307	-15.924	23.342
C	38.226	-16.993	23.184
H	40.174	-17.073	20.614
H	41.263	-15.465	22.790
H	39.520	-15.833	24.321
H	38.936	-15.049	23.013
N	37.030	-16.698	23.675
O	38.455	-18.057	22.597
H	36.874	-15.811	24.114
H	36.282	-17.358	23.608
N	41.268	-17.893	24.237
C	41.893	-19.134	24.697
C	41.184	-20.402	24.207
O	41.795	-21.462	24.128
C	42.046	-19.101	26.213
C	43.151	-18.142	26.576
H	40.748	-17.249	24.858
H	42.832	-19.156	24.310
H	41.203	-18.783	26.607
H	42.288	-20.002	26.524
N	42.932	-17.328	27.592
O	44.180	-18.115	25.904
H	42.058	-17.374	28.087
H	43.627	-16.668	27.873
N	39.916	-20.296	23.826
C	39.122	-21.454	23.472
C	39.046	-21.636	21.956
O	39.023	-22.773	21.482
C	37.770	-21.309	24.170
C	37.914	-21.076	25.657
C	38.542	-22.016	26.464
C	37.455	-19.909	26.257
C	38.699	-21.811	27.827
C	37.603	-19.689	27.616
C	38.232	-20.642	28.404
O	38.396	-20.435	29.753
H	39.551	-19.319	23.806
H	39.560	-22.277	23.860
H	37.296	-20.533	23.777
H	37.257	-22.145	24.035
H	38.891	-22.865	26.054
H	37.008	-19.212	25.693
H	39.150	-22.506	28.392
H	37.258	-18.846	28.031
H	37.675	-19.827	30.082
N	39.029	-20.543	21.174
C	38.680	-20.646	19.760
C	39.406	-19.606	18.908
O	39.835	-18.577	19.415
C	37.164	-20.479	19.623
C	36.420	-21.791	19.808
C	35.278	-21.967	18.836
N	33.985	-21.882	19.484
C	32.851	-22.257	18.913
N	31.736	-22.328	19.622
N	32.853	-22.600	17.637
H	39.275	-19.664	21.648
H	38.936	-21.567	19.428
H	36.857	-19.848	20.319
H	36.970	-20.138	18.716
H	37.061	-22.555	19.683
H	36.048	-21.831	20.740
H	35.348	-21.252	18.140
H	35.374	-22.864	18.403
H	33.945	-21.518	20.420
H	31.722	-22.083	20.589
H	30.883	-22.634	19.180
H	33.698	-22.558	17.101
H	32.004	-22.904	17.196
N	39.609	-19.918	17.633
C	40.128	-18.928	16.699
C	39.348	-19.030	15.393
O	39.450	-20.017	14.664
C	41.644	-19.001	16.500
C	42.165	-17.796	15.738
H	39.375	-20.889	17.373
H	39.938	-18.026	17.083
H	42.077	-19.025	17.391
H	41.854	-19.820	15.982
N	43.481	-17.697	15.623
O	41.374	-16.972	15.249
H	44.074	-18.398	16.022
H	43.887	-16.922	15.136
N	38.552	-17.991	15.095
C	37.740	-18.009	13.895
C	38.409	-17.292	12.731
O	37.782	-17.153	11.680
C	36.398	-17.418	14.202
H	38.576	-17.217	15.774
H	37.576	-18.973	13.625
H	35.797	-17.490	13.402
H	35.973	-17.905	14.970
H	36.494	-16.449	14.447
N	39.648	-16.818	12.927
C	40.444	-16.200	11.880
C	39.671	-15.067	11.191
O	39.600	-14.999	9.953
C	40.940	-17.257	10.887
C	41.736	-18.340	11.552
H	39.991	-16.936	13.913
H	41.272	-15.802	12.294
H	40.152	-17.676	10.435
H	41.519	-16.814	10.202
C	41.582	-19.684	11.550
N	42.812	-18.077	12.376
C	43.283	-19.232	12.852
N	42.545	-20.208	12.381
H	43.178	-17.177	12.584
H	40.899	-20.202	11.041
H	44.066	-19.324	13.461
N	39.099	-14.197	12.027
C	38.219	-13.130	11.554
C	38.160	-12.016	12.600
O	38.563	-12.203	13.758
C	36.852	-13.678	11.198
O	36.189	-14.158	12.346
H	39.332	-14.344	13.030
H	38.614	-12.746	10.719
H	36.306	-12.951	10.788
H	36.961	-14.428	10.550
H	36.621	-13.782	13.166
N	37.635	-10.851	12.187
C	37.529	-9.692	13.064
C	36.313	-8.848	12.684
O	35.985	-8.766	11.505
C	38.802	-8.878	12.969
H	37.316	-10.855	11.196
H	37.414	-10.008	14.011
H	39.436	-9.314	12.329
H	38.590	-7.955	12.645
H	39.234	-8.819	13.870
N	35.599	-8.307	13.679
C	34.453	-7.442	13.453
C	34.776	-6.045	13.963
O	35.333	-5.898	15.054
C	33.189	-7.982	14.145
C	31.963	-7.118	13.949
O	32.987	-9.270	13.556
H	35.943	-8.565	14.634
H	34.280	-7.387	12.469
H	33.379	-8.103	15.120
H	32.231	-6.154	13.862
H	31.475	-7.396	13.117
H	31.345	-7.213	14.735
H	33.251	-9.257	12.585
N	34.423	-5.026	13.178
C	34.362	-3.660	13.680
C	32.922	-3.180	13.659
O	32.149	-3.405	12.720
C	35.247	-2.687	12.879
C	36.706	-3.046	12.942
O	34.783	-2.639	11.526
H	34.201	-5.289	12.199
H	34.686	-3.653	14.628
H	35.157	-1.755	13.281
H	36.818	-4.045	12.980
H	37.186	-2.699	12.129
H	37.129	-2.644	13.761
H	34.426	-3.537	11.246
N	32.563	-2.492	14.749
C	31.324	-1.792	14.919
C	31.553	-0.284	14.852
O	31.760	0.342	15.923
C	30.646	-2.129	16.246
C	30.109	-3.530	16.369
C	28.796	-3.910	16.304
C	30.834	-4.705	16.736
C	29.890	-5.755	16.859
C	32.174	-4.981	17.019
N	28.680	-5.245	16.547
C	30.259	-7.058	17.177
C	32.539	-6.268	17.360
C	31.583	-7.283	17.446
H	33.316	-2.516	15.506
H	30.727	-2.011	14.146
H	31.306	-2.004	16.989
H	29.873	-1.507	16.383
H	28.034	-3.292	16.106
H	27.819	-5.770	16.500
H	32.865	-4.257	16.975
H	29.575	-7.789	17.205
H	33.495	-6.471	17.547
H	31.884	-8.204	17.714
N	31.417	0.437	13.694
C	31.267	1.914	13.737
C	29.835	2.304	13.938
O	29.012	2.148	12.998
C	31.746	2.605	12.465
O	31.582	3.993	12.586
H	31.203	-0.081	12.812
H	31.874	2.332	14.568
H	32.803	2.376	12.250
H	31.132	2.252	11.617
H	32.281	4.311	13.224
N	29.454	2.966	15.061
C	28.109	3.520	15.207
C	28.083	4.755	16.040
O	28.811	5.738	15.754
H	30.115	2.992	15.865
H	27.648	3.760	14.226
H	27.504	2.768	15.718
N	27.180	4.828	17.133
C	26.958	6.037	17.930
C	26.146	5.724	19.161
O	25.381	4.731	19.182
C	26.313	7.170	17.106
C	24.975	6.785	16.461
C	24.482	7.912	15.606
H	26.656	3.959	17.370
H	27.944	6.398	18.277
H	26.164	8.056	17.764
H	27.019	7.485	16.309
H	25.100	5.871	15.844
H	24.216	6.577	17.245
N	24.435	7.811	14.264
O	24.171	9.006	16.123
H	24.275	8.698	13.742
H	24.798	6.975	13.772
N	26.263	6.495	20.258
C	25.504	6.327	21.503
C	24.497	7.469	21.532
O	24.751	8.604	21.061
C	26.526	6.418	22.629
C	25.909	6.808	23.948
C	25.271	5.856	24.729
C	25.903	8.127	24.368
C	24.665	6.200	25.924
C	25.347	8.478	25.593
C	24.716	7.513	26.359
O	24.147	7.801	27.583
H	26.781	7.390	20.147
H	25.002	5.468	21.508
H	26.956	5.529	22.735
H	27.201	7.105	22.386
H	25.247	4.900	24.423

H	26.301	8.834	23.790
H	24.194	5.506	26.471
H	25.403	9.421	25.917
H	24.720	7.421	28.306
N	23.248	7.136	21.897
C	22.153	8.074	22.013
C	21.616	7.909	23.438
O	21.083	6.860	23.778
C	21.056	7.787	20.963
C	19.948	8.822	20.994
C	21.607	7.666	19.549
H	23.140	6.102	22.095
H	22.507	8.992	21.929
H	20.643	6.905	21.195
H	19.065	8.375	20.842
H	19.938	9.278	21.885
H	20.102	9.502	20.276
H	22.305	6.807	19.475
H	20.851	7.516	18.912
H	22.147	8.595	19.265
N	21.768	8.941	24.255
C	21.415	8.852	25.665
C	19.927	9.108	25.873
O	19.201	9.365	24.926
H	22.151	9.798	23.813
H	21.646	7.935	26.013
H	21.945	9.530	26.189
N	19.501	9.099	27.135
C	18.118	9.368	27.482
C	17.541	8.174	28.222
O	18.288	7.286	28.639
H	20.240	8.885	27.838
H	18.067	10.179	28.068
H	17.586	9.532	26.649
N	16.215	8.140	28.359
C	15.603	7.207	29.296
C	15.821	5.790	28.795
O	16.052	4.877	29.600
C	14.140	7.515	29.466
H	15.692	8.800	27.772
H	16.046	7.305	30.188
H	13.596	6.684	29.327
H	13.968	7.865	30.390
H	13.855	8.206	28.798
N	15.753	5.649	27.459
C	15.951	4.383	26.783
C	17.199	4.502	25.907
O	17.096	4.633	24.691
C	14.714	4.017	25.960
C	13.423	4.022	26.774
C	12.607	2.749	26.654
O	12.429	2.267	25.521
O	12.148	2.247	27.698
H	15.543	6.545	26.956
H	16.083	3.653	27.448
H	14.618	4.685	25.249
H	14.841	3.109	25.612
H	13.662	4.135	27.726
H	12.860	4.768	26.453
N	18.359	4.430	26.570
C	19.655	4.657	25.955
C	19.981	3.520	24.984
O	19.687	2.344	25.216
C	20.679	4.797	27.042
H	18.244	4.191	27.589
H	19.628	5.517	25.438
H	20.593	5.694	27.484
H	20.549	4.082	27.733
H	21.603	4.713	26.659
N	20.658	3.870	23.878
C	20.924	2.909	22.827
C	22.311	3.191	22.249
O	22.745	4.358	22.232
C	19.873	3.074	21.710
C	18.682	2.125	21.778
C	17.633	2.459	20.732
N	16.689	1.370	20.535
C	15.881	0.862	21.457
N	15.080	-0.138	21.137
N	15.857	1.346	22.688
H	20.959	4.859	23.854
H	20.923	1.988	23.212
H	19.525	4.012	21.752
H	20.333	2.932	20.833
H	18.995	1.191	21.620
H	18.257	2.193	22.678
H	17.142	3.273	21.036
H	18.104	2.649	19.873
H	16.642	0.957	19.603
H	15.074	-0.541	20.228
H	14.449	-0.512	21.838
H	16.481	2.101	22.925
H	15.238	0.974	23.369
N	22.955	2.143	21.764
C	24.073	2.304	20.828
C	23.635	1.684	19.510
O	23.340	0.499	19.435
C	25.371	1.694	21.371
C	25.767	2.356	22.703
C	26.499	1.792	20.345
C	26.918	1.693	23.386
H	22.615	1.222	22.090
H	24.164	3.277	20.671
H	25.211	0.728	21.549
H	25.993	3.312	22.515
H	24.966	2.332	23.303
H	27.310	1.332	20.700
H	26.212	1.355	19.495
H	26.745	2.854	20.149
H	27.726	1.718	22.794
H	27.126	2.169	24.243
H	26.690	0.738	23.589
N	23.444	2.555	18.460
C	23.124	2.128	17.120
C	24.335	1.960	16.259
O	25.049	2.951	15.957
C	22.099	3.032	16.427
C	20.867	3.228	17.232
H	23.514	3.580	18.624
H	22.742	1.155	17.271
H	22.574	4.008	16.189
H	21.791	2.566	15.468
N	20.432	4.457	17.399
O	20.266	2.231	17.623
H	20.968	5.286	17.075
H	19.550	4.622	17.860
N	24.532	0.763	15.650
C	25.772	0.406	14.941
C	25.524	-0.133	13.574
O	24.448	-0.728	13.293
C	26.689	-0.546	15.754
C	26.878	-0.036	17.165
O	26.249	-1.880	15.821
H	23.765	0.073	15.774
H	26.350	1.321	14.769
H	27.688	-0.550	15.262
H	25.947	-0.172	17.753
H	27.672	-0.632	17.618
H	27.190	1.026	17.179
H	25.277	-1.867	16.069
N	26.433	0.111	12.594
C	26.695	-0.857	11.533
C	28.046	-1.510	11.714
O	28.939	-0.942	12.398
C	26.525	-0.198	10.159
C	25.030	-0.169	9.813
C	24.696	0.812	8.753
H	27.178	0.813	12.773
H	25.964	-1.677	11.610
H	26.959	0.825	10.180
H	27.059	-0.781	9.375
H	24.730	-1.183	9.475
H	24.424	0.092	10.706
N	24.720	2.128	9.021
O	24.034	0.429	7.766
H	24.408	2.788	8.280
H	25.182	2.506	9.872
N	28.201	-2.807	11.329
C	29.410	-3.555	11.519
C	29.916	-4.172	10.209
O	29.124	-4.429	9.291
C	29.243	-4.613	12.617
C	28.111	-5.570	12.491
C	26.947	-5.541	13.209
C	28.067	-6.771	11.698
C	26.828	-7.390	11.951
C	28.920	-7.347	10.753
N	26.167	-6.609	12.856
C	26.419	-8.554	11.299
C	28.542	-8.527	10.152
C	27.307	-9.108	10.405
H	27.386	-3.134	10.786
H	30.130	-2.923	11.843
H	30.093	-5.159	12.663
H	29.135	-4.137	13.503
H	26.706	-4.850	13.883
H	25.242	-6.794	13.210
H	29.787	-6.913	10.515
H	25.524	-8.955	11.482
H	29.178	-8.972	9.519
H	27.057	-9.952	9.924
N	31.226	-4.412	10.206
C	31.938	-5.179	9.199
C	32.704	-6.325	9.852
O	33.540	-6.091	10.722
C	32.932	-4.280	8.475
C	32.372	-3.110	7.664
C	33.499	-2.147	7.255
C	31.654	-3.619	6.418
H	31.726	-3.975	11.039
H	31.271	-5.570	8.558
H	33.553	-3.892	9.162
H	33.461	-4.851	7.843
H	31.707	-2.614	8.219
H	34.164	-2.641	6.699
H	33.110	-1.394	6.728
H	33.940	-1.793	8.077
H	30.900	-4.217	6.689
H	31.295	-2.842	5.900
H	32.298	-4.131	5.849
N	32.473	-7.524	9.311
C	33.130	-8.752	9.725
C	33.963	-9.292	8.575
O	33.406	-9.710	7.552
C	32.045	-9.747	10.161
C	32.519	-11.086	10.734
C	31.314	-11.939	11.099
C	33.417	-11.818	9.796
H	31.753	-7.499	8.537
H	33.741	-8.549	10.492
H	31.491	-9.303	10.864
H	31.479	-9.948	9.364
H	33.064	-10.914	11.549
H	30.761	-12.105	10.282
H	31.624	-12.814	11.473
H	30.761	-11.462	11.783
H	34.235	-11.268	9.594
H	33.710	-12.691	10.201
H	32.939	-12.010	8.932
N	35.294	-9.217	8.742
C	36.248	-9.682	7.753
C	36.808	-11.031	8.206
O	37.229	-11.223	9.354
C	37.390	-8.693	7.476
C	38.323	-9.174	6.387
O	36.846	-7.431	7.059
H	35.577	-8.789	9.658
H	35.775	-9.817	6.884
H	37.914	-8.542	8.339
H	38.434	-10.170	6.444
H	37.949	-8.940	5.485
H	39.223	-8.741	6.488
H	36.686	-6.841	7.856
N	36.820	-11.963	7.251
C	37.397	-13.291	7.438

C	38.683	-13.332	6.627
O	38.705	-12.866	5.477
C	36.387	-14.364	6.985
C	35.091	-14.308	7.798
C	36.997	-15.753	6.980
C	33.881	-13.847	6.989
H	36.377	-11.657	6.352
H	37.659	-13.350	8.392
H	36.159	-14.186	6.028
H	34.904	-15.216	8.163
H	35.222	-13.674	8.557
H	36.351	-16.402	6.579
H	37.838	-15.749	6.441
H	37.206	-16.031	7.917
H	33.736	-14.480	6.233
H	33.080	-13.836	7.582
H	34.056	-12.931	6.637
N	39.768	-13.820	7.244
C	41.000	-14.084	6.520
C	40.749	-15.007	5.325
O	40.181	-16.066	5.499
H	39.646	-13.986	8.260
H	41.392	-13.218	6.188
H	41.669	-14.518	7.135
N	41.083	-14.568	4.109
C	40.804	-15.362	2.917
C	42.069	-15.404	2.062
O	42.997	-14.633	2.281
C	39.642	-14.841	2.041
C	38.334	-14.610	2.771
O	40.052	-13.634	1.403
H	41.543	-13.643	4.090
H	40.582	-16.292	3.196
H	39.441	-15.534	1.323
H	37.566	-14.679	2.129
H	38.218	-15.298	3.493
H	38.330	-13.697	3.187
H	40.556	-13.836	0.555
N	42.087	-16.282	1.054
C	43.037	-16.156	-0.038
C	42.649	-14.945	-0.888
O	41.500	-14.479	-0.878
C	43.079	-17.449	-0.874
C	43.222	-18.690	-0.020
O	41.857	-17.529	-1.611
H	41.381	-17.036	1.124
H	43.946	-15.993	0.340
H	43.847	-17.388	-1.532
H	43.635	-18.454	0.864
H	42.321	-19.102	0.142
H	43.808	-19.361	-0.483
H	42.000	-18.023	-2.476
N	43.601	-14.490	-1.698
C	43.383	-13.375	-2.606
C	42.254	-13.724	-3.584
O	41.454	-12.856	-3.916
C	44.708	-13.002	-3.288
C	45.792	-12.582	-2.292
C	47.225	-12.534	-2.806
O	48.047	-13.372	-2.360
O	47.533	-11.637	-3.599
H	44.509	-14.999	-1.624
H	43.130	-12.556	-2.090
H	45.033	-13.793	-3.762
H	44.538	-12.241	-3.877
H	45.565	-11.667	-1.962
H	45.773	-13.227	-1.528
N	42.148	-15.008	-3.975
C	41.128	-15.496	-4.898
C	39.708	-15.296	-4.351
O	38.813	-14.949	-5.122
C	41.371	-16.963	-5.187
H	42.877	-15.634	-3.548
H	41.206	-14.988	-5.769
H	40.695	-17.294	-5.847
H	42.289	-17.085	-5.566
H	41.291	-17.492	-4.341
N	39.531	-15.572	-3.047
C	38.264	-15.470	-2.322
C	38.066	-14.123	-1.590
O	37.082	-13.964	-0.888
C	38.138	-16.604	-1.303
C	38.024	-17.954	-1.978
H	40.428	-15.883	-2.570
H	37.518	-15.536	-3.008
H	38.948	-16.601	-0.729
H	37.322	-16.448	-0.760
N	38.347	-19.002	-1.247
O	37.628	-18.036	-3.137
H	38.643	-18.886	-0.296
H	38.298	-19.926	-1.637
N	38.911	-13.119	-1.823
C	38.825	-11.844	-1.108
C	37.484	-11.136	-1.357
O	36.919	-10.507	-0.457
C	39.996	-10.948	-1.493
H	39.630	-13.317	-2.549
H	38.911	-12.020	-0.118
H	39.783	-10.001	-1.256
H	40.814	-11.238	-0.999
H	40.159	-11.017	-2.477
N	36.944	-11.271	-2.568
C	35.654	-10.702	-2.914
C	34.564	-11.147	-1.941
O	33.628	-10.393	-1.721
C	35.276	-11.016	-4.372
C	34.911	-12.448	-4.601
C	35.776	-13.478	-4.866
C	33.604	-13.042	-4.462
C	33.755	-14.425	-4.734
C	32.316	-12.532	-4.221
N	35.087	-14.656	-4.972
C	32.672	-15.308	-4.705
C	31.243	-13.402	-4.217
C	31.420	-14.767	-4.471
H	37.529	-11.819	-3.239
H	35.728	-9.698	-2.844
H	34.491	-10.463	-4.622
H	36.053	-10.808	-4.953
H	36.765	-13.380	-4.967
H	35.486	-15.549	-5.189
H	32.179	-11.558	-4.055
H	32.808	-16.288	-4.849
H	30.330	-13.049	-4.031
H	30.618	-15.365	-4.483
N	34.685	-12.330	-1.317
C	33.679	-12.777	-0.374
C	34.198	-12.835	1.080
O	33.707	-13.622	1.887
C	33.114	-14.112	-0.855
C	34.097	-15.263	-0.911
C	33.401	-16.573	-1.166
C	34.398	-17.703	-1.221
N	33.862	-18.807	-2.042
H	35.531	-12.865	-1.567
H	32.891	-12.138	-0.398
H	32.360	-14.370	-0.240
H	32.736	-13.974	-1.778
H	34.756	-15.092	-1.647
H	34.588	-15.315	-0.038
H	32.759	-16.750	-0.428
H	32.924	-16.526	-2.037
H	35.248	-17.371	-1.625
H	34.572	-18.032	-0.295
H	32.877	-18.681	-2.175
H	34.317	-18.816	-2.934
H	34.024	-19.681	-1.580
N	35.110	-11.920	1.464
C	35.729	-11.940	2.788
C	34.966	-11.100	3.817
O	35.273	-11.209	4.996
C	37.143	-11.442	2.678
O	37.114	-10.071	2.290
H	35.328	-11.208	0.738
H	35.733	-12.899	3.117
H	37.591	-11.512	3.555
H	37.624	-11.955	1.984
H	37.015	-10.011	1.299
N	34.042	-10.206	3.405
C	33.501	-9.215	4.347
C	31.982	-9.229	4.408
O	31.299	-8.984	3.420
C	33.980	-7.785	4.033
C	33.687	-6.785	5.140
O	35.390	-7.855	3.896
H	33.768	-10.281	2.423
H	33.861	-9.430	5.270
H	33.581	-7.497	3.151
H	32.698	-6.638	5.213
H	34.032	-7.134	6.015
H	34.134	-5.911	4.937
H	35.655	-8.646	3.329
N	31.440	-9.503	5.606
C	30.011	-9.379	5.855
C	29.714	-7.999	6.454
O	30.543	-7.443	7.158
C	29.553	-10.453	6.843
C	29.659	-11.900	6.392
C	29.452	-12.860	7.577
C	28.601	-12.160	5.342
H	32.127	-9.807	6.325
H	29.522	-9.454	4.986
H	30.105	-10.355	7.673
H	28.593	-10.274	7.061
H	30.565	-12.062	6.001
H	28.546	-12.707	7.966
H	29.527	-13.800	7.252
H	30.151	-12.683	8.266
H	28.743	-11.551	4.559
H	28.657	-13.112	5.033
H	27.692	-11.992	5.728
N	28.482	-7.531	6.236
C	27.995	-6.271	6.749
C	26.655	-6.505	7.435
O	25.839	-7.292	6.986
C	27.914	-5.210	5.629
C	26.916	-5.572	4.549
C	27.612	-3.848	6.221
H	27.890	-8.177	5.646
H	28.638	-5.936	7.438
H	28.818	-5.164	5.194
H	27.398	-5.749	3.689
H	26.410	-6.393	4.818
H	26.273	-4.816	4.417
H	26.626	-3.680	6.185
H	27.921	-3.822	7.173
H	28.090	-3.143	5.696
N	26.439	-5.800	8.541
C	25.156	-5.846	9.210
C	25.000	-4.670	10.160
O	25.714	-3.674	10.083
H	27.244	-5.237	8.866
H	24.415	-5.821	8.527
H	25.074	-6.705	9.730
N	23.973	-4.767	11.000
C	23.665	-3.677	11.923
C	23.239	-4.276	13.254
O	22.531	-5.273	13.268
C	22.590	-2.767	11.336
C	21.395	-3.483	10.812
C	20.166	-3.728	11.358
N	21.381	-4.061	9.557
C	20.178	-4.598	9.350
N	19.433	-4.415	10.422
H	23.436	-5.643	10.944
H	24.493	-3.141	12.068
H	22.286	-2.119	12.049
H	22.995	-2.229	10.582
H	22.138	-4.077	8.917
H	19.864	-3.455	12.265
H	19.893	-5.061	8.512
N	23.718	-3.704	14.358
C	23.348	-4.197	15.679
C	22.879	-2.985	16.485
O	23.495	-1.914	16.410

C	24.531	-4.825	16.416
C	25.020	-6.202	16.008
O	24.356	-6.855	15.130
O	26.104	-6.608	16.526
H	24.354	-2.906	14.198
H	22.563	-4.791	15.567
H	25.318	-4.174	16.340
H	24.290	-4.856	17.410
N	21.788	-3.143	17.249
C	21.394	-2.106	18.189
C	21.557	-2.634	19.616
O	21.033	-3.704	19.938
C	19.946	-1.655	18.021
C	19.558	-0.546	18.976
O	19.743	-1.203	16.686
H	21.283	-4.027	17.111
H	22.001	-1.319	18.076
H	19.328	-2.452	18.186
H	18.619	-0.686	19.303
H	20.179	-0.538	19.765
H	19.611	0.342	18.511
H	19.795	-0.198	16.641
N	22.239	-1.854	20.455
C	22.521	-2.243	21.836
C	21.655	-1.421	22.791
O	21.506	-0.195	22.667
C	24.009	-2.091	22.152
C	24.915	-2.938	21.310
C	25.317	-2.527	20.042
C	25.385	-4.140	21.772
C	26.155	-3.331	19.299
C	26.230	-4.943	21.026
C	26.587	-4.543	19.762
H	22.552	-0.948	20.043
H	22.235	-3.197	21.936
H	24.253	-1.140	22.006
H	24.144	-2.347	23.101
H	25.003	-1.662	19.678
H	25.107	-4.458	22.696
H	26.455	-3.002	18.376
H	26.572	-5.810	21.415
H	27.157	-5.130	19.186
N	21.083	-2.108	23.802
C	20.319	-1.416	24.834
C	20.844	-1.847	26.225
O	21.593	-2.807	26.337
C	18.812	-1.680	24.671
C	18.251	-1.263	23.328
O	18.534	-3.074	24.769
H	21.234	-3.125	23.773
H	20.465	-0.434	24.744
H	18.311	-1.199	25.425
H	17.257	-1.403	23.312
H	18.446	-0.293	23.160
H	18.668	-1.810	22.596
H	18.355	-3.332	25.726
N	20.481	-1.092	27.266
C	20.990	-1.294	28.619
C	20.151	-2.333	29.365
O	20.556	-2.798	30.426
C	21.001	0.019	29.407
C	22.019	1.067	28.965
C	22.981	1.477	30.067
C	24.045	0.415	30.275
N	24.485	0.282	31.690
H	19.794	-0.337	27.024
H	21.938	-1.626	28.566
H	20.083	0.430	29.342
H	21.179	-0.200	30.375
H	22.550	0.696	28.192
H	21.524	1.884	28.644
H	23.425	2.326	29.807
H	22.474	1.589	30.913
H	23.683	-0.475	29.959
H	24.847	0.640	29.702
H	24.420	-0.676	31.969
H	25.432	0.591	31.773
H	23.898	0.842	32.274
N	19.005	-2.693	28.778
C	18.141	-3.773	29.231
C	18.005	-4.774	28.081
O	17.968	-4.352	26.934
C	16.772	-3.202	29.663
C	16.907	-2.218	30.830
C	16.017	-2.559	28.495
H	18.774	-2.104	27.927
H	18.586	-4.238	29.989
H	16.215	-3.963	29.991
H	16.556	-1.326	30.551
H	16.383	-2.557	31.609
H	17.869	-2.137	31.082
H	15.056	-2.458	28.746
H	16.415	-1.664	28.304
H	16.099	-3.146	27.692
N	17.914	-6.081	28.365
C	17.869	-7.090	27.303
C	16.539	-6.996	26.547
O	16.538	-7.431	25.380
C	18.010	-8.527	27.821
C	18.771	-8.658	29.130
C	19.370	-10.015	29.362
C	20.114	-10.075	30.679
N	20.924	-11.313	30.782
O	15.463	-6.574	26.861
H	17.879	-6.306	29.373
H	18.617	-6.918	26.659
H	17.091	-8.900	27.952
H	18.488	-9.062	27.123
H	19.510	-7.963	29.143
H	18.140	-8.439	29.893
H	18.642	-10.697	29.379
H	20.012	-10.222	28.627
H	20.718	-9.278	30.750
H	19.451	-10.051	31.431
H	20.355	-12.104	30.556
H	21.693	-11.263	30.144
H	21.272	-11.408	31.715

REFERENCES

(1) Hong, S. Y.; Park, Y.; Hwang, Y.; Kim, Y. B.; Baik, M. H.; Chang, S. Selective formation of gamma-lactams via C-H amidation enabled by tailored iridium catalysts. *Science* **2018**, *359*, 1016-1021.

(2) Zimbron, J. M.; Heinisch, T.; Schmid, M.; Hamels, D.; Nogueira, E. S.; Schirmer, T.; Ward, T. R. A Dual Anchoring Strategy for the Localization and Activation of Artificial Metalloenzymes Based on the Biotin-Streptavidin Technology. *J. Am. Chem. Soc.* **2013**, *135*, 5384-5388.

(3) Frisch, M. J. T., G. W.; Schlegel, H. B.; Scuseria, G. E.; Robb, M. A.; Cheeseman, J. R.; Saclmani, G.; Barone, V.; Petersson, G. A.; Nakatsuji, H.; Li, X.; Caricato, M.; Marenich, A.; Bloino, J.; Janesko, B. G.; Gomperts, R.; Mennucci, B.; Hratchian, H. P.; Ortiz, J. V.; Izmaylov, A. F.; Sonnenberg, J. L.; Williams-Young, D.; Ding, F.; Lipparini, F.; Egidi, F.; Goings, J.; Peng, B.; Petrone, A.; Henderson, T.; Ranasinghe, D.; Zakrzewski, V. G.; Gao, J.; Rega, N.; Zheng, G.; Liang, W.; Hada, M.; Ehara, M.; Toyota, K.; Fukuda, R.; Hasegawa, J.; Ishida, M.; Nakajima, T.; Honda, Y.; Kitao, O.; Nakai, H.; Vreven, T.; Throssell, K.; Montgomery, J. A.; Jr., Peralta, J. E.; Ogliaro, F.; Bearpark, M.; Heyd, J. J.; Brothers, E.; Kudin, K. N.; Staroverov, V. N.; Keith, T.; Kobayashi, R.; Normand, J.; Raghavachari, K.; Rendell, A.; Burant, J. C.; Iyengar, S; Romasi, J.; Cossi, M.; Millam, J. M.; Klene, M.; Adamo, C.; Fox, R. . Gaussian 09, Revision E.01. *Gaussian, Inc., Wallingford CT* **2016**.

(4) Park, Y.; Chang, S. Asymmetric formation of γ-lactams via C–H amidation enabled by chiral hydrogen-bond-donor catalysts. *Nat. Catal.* **2019**, *2*, 219-227.

(5) Letondor, C.; Pordea, A.; Humbert, N.; Ivanova, A.; Mazurek, S.; Novic, M.; Ward, T. R. Artificial transfer hydrogenases based on the biotin-(strept)avidin technology: Fine tuning the selectivity by saturation mutagenesis of the host protein. *J. Am. Chem. Soc.* **2006**, *128*, 8320-8328.

(6) Serrano-Plana, J.; Rumo, C.; Rebelein, J. G.; Peterson, R. L.; Barnet, M.; Ward, T. R. Enantioselective Hydroxylation of Benzylic C(sp(3))-H Bonds by an Artificial Iron Hydroxylase Based on the Biotin-Streptavidin Technology. *J. Am. Chem. Soc.* **2020**, *142*, 10617-10623.

(7) Zhu, X. L.; Qiao, L.; Ye, P. P.; Ying, B. B.; Xu, J.; Shen, C.; Zhang, P. F. Copper-catalyzed rapid C-H nitration of 8-aminoquinolines by using sodium nitrite as the nitro source under mild conditions. *RSC Adv.* **2016**, *6*, 89979-89983.

(8) Quinto, T.; Schwizer, F.; Zimbron, J. M.; Morina, A.; Kohler, V.; Ward, T. R. Expanding the Chemical Diversity in Artificial Imine Reductases Based on the Biotin- Streptavidin Technology. *ChemCatChem* **2014**, *6*, 1010-1014.

(9) Facchetti, G.; Rimoldi, I. 8-Amino-5,6,7,8-tetrahydroquinoline in iridium(III) biotinylated Cp* complex as artificial imine reductase. *New J. Chem.* **2018**, *42*, 18773-18776.

(10) Park, Y.; Chang, S. Asymmetric formation of γ-lactams via C–H amidation enabled by chiral hydrogen-bond-donor catalysts. *Nature Catal.* **2019**, *2*, 219-227.

(11) Wang, H.; Park, Y.; Bai, Z.; Chang, S.; He, G.; Chen, G. Iridium-Catalyzed Enantioselective C(sp(3))-H Amidation Controlled by Attractive Noncovalent Interactions. *J. Am. Chem. Soc.* **2019**, *141*, 7194-7201.

(12) Xing, Q.; Chan, C. M.; Yeung, Y. W.; Yu, W. Y. Ruthenium(II)-Catalyzed Enantioselective gamma-Lactams Formation by Intramolecular C-H Amidation of 1,4,2-Dioxazol-5-ones. *J. Am. Chem. Soc.* **2019**, *141*, 3849-3853.

(13) Kweon, J.; Chang, S. Highly Robust Iron Catalyst System for Intramolecular C(sp(3))-H Amidation Leading to gamma-Lactams. *Angew. Chem., Int. Ed.* **2021**, *60*, 2909-2914.

(14) Kada, G.; Kaiser, K.; Falk, H.; Gruber, H. J. Rapid estimation of avidin and streptavidin by fluorescence quenching or fluorescence polarization. *Biochim. Biophys. Acta* **1999**, *1427*, 44-48.
(15) Skander, M.; Humbert, N.; Collot, J.; Gradinaru, J.; Klein, G.; Loosli, A.; Sauser, J.; Zocchi, A.; Gilardoni, F.; Ward, T. R. Artificial metalloenzymes: (Strept)avidin as host for enantioselective hydrogenation by achiral biotinylated rhodium-diphosphine complexes. *J. Am. Chem. Soc.* **2004**, *126*, 14411-14418.
(16) Wang, Z.-X. An Exact Mathematical Expression for Describing Competitive Binding of Two Different Ligands to a Protein Molecule. *FEBS Lett.* **1995**, *360*, 111-114.
(17) Rumo, C.; Stein, A.; Klehr, J.; Tachibana, R.; Prescimone, A.; Haussinger, D.; Ward, T. R. An Artificial Metalloenzyme Based on a Copper Heteroscorpionate Enables sp(3) C-H Functionalization via Intramolecular Carbene Insertion. *J. Am. Chem. Soc.* **2022**, *144*, 11676-11684.
(18) W, K. XDS. *Acta Cryst.* **2010**, *66*, 125-131.
(19) Evans, P. R.; Murshudov, G. N. How good are my data and what is the resolution? *Acta Cryst.* **2013**, *69*, 1204-1214.
(20) Potterton, L.; Agirre, J.; Ballard, C.; Cowtan, K.; Dodson, E.; Evans, P. R.; Jenkins, H. T.; Keegan, R.; Krissinel, E.; Stevenson, K.; et al. CCP4i2: the new graphical user interface to the CCP4 program suite. *Acta Cryst.* **2018**, *74*, 68-84.
(21) Mccoy, A. J.; Grosse-Kunstleve, R. W.; Adams, P. D.; Winn, M. D.; Storoni, L. C.; Read, R. J. Phaser crystallographic software. *J. Appl. Cryst.* **2007**, *40*, 658-674.
(22) Murshudov, G. N.; Skubak, P.; Lebedev, A. A.; Pannu, N. S.; Steiner, R. A.; Nicholls, R. A.; Winn, M. D.; Long, F.; Vagin, A. A. REFMAC5 for the refinement of macromolecular crystal structures. *Acta Cryst.* **2011**, *67*, 355-367.
(23) Emsley, P.; Lohkamp, B.; Scott, W. G.; Cowtan, K. Features and development of Coot. *Acta Cryst.* **2010**, *66*, 486-501.
(24) Cividino, P.; Py, S.; Delair, P.; Greene, A. E. 1-(2,4,6-Triisopropylphenyl)ethylamine: A new chiral auxiliary for the asymmetric synthesis of gamma-amino acid derivatives. *J. Org. Chem.* **2007**, *72*, 485-493.
